# The analysis of gut microbiota in patients with bile acid diarrhoea treated with colesevelam

**DOI:** 10.3389/fmicb.2023.1134105

**Published:** 2023-03-17

**Authors:** Aditi Kumar, Mohammed Nabil Quraishi, Hafid O. Al-Hassi, Mohammed E. El-Asrag, Jonathan P. Segal, Manushri Jain, Helen Steed, Jeffrey Butterworth, Adam Farmer, John Mclaughlin, Andrew Beggs, Matthew J. Brookes

**Affiliations:** ^1^Department of Gastroenterology, The Royal Wolverhampton NHS Trust, Wolverhampton, United Kingdom; ^2^Microbiome Treatment Centre, University of Birmingham Microbiome Treatment Centre, Birmingham, United Kingdom; ^3^Department of Gastroenterology, University Hospitals Birmingham NHS Foundation Trust, Birmingham, United Kingdom; ^4^Faculty of Science and Engineering, University of Wolverhampton, Wolverhampton, United Kingdom; ^5^Institute of Cancer and Genomic Sciences, University of Birmingham, Birmingham, United Kingdom; ^6^Faculty of Science, Benha University, Benha, Egypt; ^7^Department of Gastroenterology, Northern Hospital, Melbourne, VIC, Australia; ^8^Department of Medicine, University of Melbourne, Parkville, VIC, Australia; ^9^School of Medicine and Clinical Practice, Faculty of Sciences and Engineering, University of Wolverhampton, Wolverhampton, United Kingdom; ^10^Department of Gastroenterology, Shrewsbury and Telford Hospital NHS Trust, Shrewsbury, United Kingdom; ^11^Department of Gastroenterology, University Hospitals of North Midlands, Stoke-on-Trent, United Kingdom; ^12^Division of Diabetes, Endocrinology and Gastroenterology, Faculty of Biology Medicine and Health, The University of Manchester, Manchester Academic Health Science Centre, Manchester, United Kingdom; ^13^Department of Gastroenterology, Salford Royal Foundation Trust, Salford, United Kingdom

**Keywords:** microbiome, Crohn’s disease, bile acid diarrhoea, colesevelam, post-cholecystectomy

## Abstract

**Introduction:**

Bile acid diarrhoea (BAD) is a common disorder that results from an increased loss of primary bile acids and can result in a change in microbiome. The aims of this study were to characterise the microbiome in different cohorts of patients with BAD and to determine if treatment with a bile acid sequestrant, colesevelam, can alter the microbiome and improve microbial diversity.

**Materials and methods:**

Patients with symptoms of diarrhoea underwent 75-selenium homocholic acid (^75^SeHCAT) testing and were categorised into four cohorts: idiopathic BAD, post-cholecystectomy BAD, post-operative Crohn’s disease BAD and ^75^SeHCAT negative control group. Patients with a positive ^75^SeHCAT (<15%) were given a trial of treatment with colesevelam. Stool samples were collected pre-treatment, 4-weeks, 8-weeks and 6–12 months post-treatment. Faecal 16S ribosomal RNA gene analysis was undertaken.

**Results:**

A total of 257 samples were analysed from 134 patients. α-diversity was significantly reduced in patients with BAD and more specifically, in the idiopathic BAD cohort and in patients with severe disease (SeHCAT <5%); *p* < 0.05. Colesevelam did not alter bacterial α/β-diversity but patients who clinically responded to treatment had a significantly greater abundance of *Fusobacteria* and *Ruminococcus*, both of which aid in the conversion of primary to secondary bile acids.

**Conclusion:**

This is the first study to examine treatment effects on the microbiome in BAD, which demonstrated a possible association with colesevelam on the microbiome through bile acid modulation in clinical responders. Larger studies are now needed to establish a causal relationship with colesevelam and the inter-crosstalk between bile acids and the microbiome.

## Introduction

Bile acid diarrhoea (BAD) affects 1% of the general population and is often misdiagnosed as functional diarrhoea or diarrhoea-predominant irritable bowel syndrome (D-IBS; Khalid et al., 2010; Fani et al., 2018). BAD may be caused either by malabsorption or overproduction of bile acids and can be classified based on the underlying pathology. Type 1 BAD results from ileal resection or ileal inflammation where the site of bile acid reabsorption is impaired (Pattni and Walters, 2009). This is prevalent in patients with Crohn’s disease, a chronic relapsing–remitting inflammatory condition of the gastrointestinal tract (Lamb et al., 2019). Greater than 90% of patients that have had a terminal ileal resection are eventually diagnosed with BAD and 11–52% are diagnosed in non-resected Crohn’s disease patients (Barkun et al., 2013). Type 2 BAD is known as idiopathic or primary BAD, which is a combination of excessive bile acid production and impaired absorption (Pattni and Walters, 2009; Tiratterra et al., 2018). Type 3 BAD can be from numerous intestinal conditions such as post-cholecystectomy (the most common cause), small intestinal bacterial overgrowth, coeliac disease, post-radiation enteritis or pancreatic insufficiency (Walters and Pattni, 2010). As there is varied aetiology in type 3, the underlying pathophysiological mechanisms will also differ.

A recent study demonstrated that patients with BAD had reduced microbial α-diversity compared to healthy controls and D-IBS, which may be a cause or result of bile acid modulation (Sagar et al., 2020). Bile acids, derived from cholesterol in the liver, undergo conjugation with glycine or taurine derivatives to form two primary bile acids: cholic acid and chenodeoxycholic acid (Hegyi et al., 2018). These primary bile acids circulate through the small intestine before being reabsorbed in the terminal ileum back into the enterohepatic circulation (Ridlon et al., 2014; Hegyi et al., 2018). Unabsorbed primary bile acids will continue into the colon where they undergo biotransformation by the microbiota to form secondary bile acids: lithocholic acid, deoxycholic acid and ursodeoxycholic acid. These will either be reabsorbed or excreted in the faeces. Biotransformation includes deconjugation *via* bile salt hydrolase, epimerisation, oxidation, dihydroxylation and hydroxylation *via* hydroxysteroid dehydrogenase (Doden and Ridlon, 2021). Whilst deconjugation *via* bile salt hydrolase is present in all major bacterial divisions including members of *Lactobacilli, Bifidobacterial, Clostridium* and *Listeria* (Jones et al., 2008; Jia et al., 2018), the most potent deconjugating bacteria are *Firmicutes* (30%), *Bacteroidetes* (14.4%) and *Actinobacteria* (8.9%; Jones et al., 2008; Duboc et al., 2013). The conversion of primary to secondary bile acids through the complex biotransformation process of 7α-dehydroxylation is one of the most quantitatively important processes performed by colonic microflora yet only 0.0001% of colonic bacteria are capable of performing this reaction, specifically only the *Clostridium* genus (Ridlon et al., 2006; Winston and Theriot, 2020).

Intraluminal bile acid binders such as cholestyramine and colestipol are first-line treatment for BAD. However, poor palatability due to texture and taste of the resin powder results in poor treatment compliance (Halilbasic et al., 2013). Colesevelam, an unlicensed bile acid sequestrant, is available in tablet form and is generally better tolerated (Wedlake et al., 2009; DG44, 2021). Colesevelam differs structurally from conventional bile acid sequestrants due to its numerous hydrophobic side chains specifically added to enhance bile acid binding (Donovan et al., 2005). It thus forms nonabsorbable complexes with bile acids in the gastrointestinal tract and are subsequently removed from the enterohepatic circulation (Nwose and Jones, 2013). Importantly, colesevelam is not absorbed systemically and is excreted unchanged from the gastrointestinal tract (Heller et al., 2002). Whether colesevelam has an effect on bile acid receptors and transport/absorption pathways with subsequent microbiome modulation is currently unknown.

This study aims to characterise the microbiome in patients with BAD, to compare the microbial diversity between the different types of BAD and determine if treatment with colesevelam can alter the microbiome and improve microbial diversity.

## Materials and methods

### Ethical approval and good clinical practise

The study was performed in accordance with the recommendations guiding physicians in biomedical research involving human subjects, adopted by the 18th World Medical Assembly, Helsinki, Finland 1964, amended at Edinburgh in 2000. The study was conducted in accordance with the International Conference on Harmonisation Good Clinical Practise (ICH GCP) guidelines. Patient information was anonymised and any collection of patient data was in compliance of the Data Protection Act 1998. The study underwent full ethical approval by London-Stanmore Research Ethics Committee. REC ref.: 16/LO/1325. Written and informed consent was obtained from all participants in the trial. All authors had access to the study data and reviewed and approved the final manuscript.

### Study design

The Bile Acid Diarrhoea study design has been published previously, including eligibility criteria (Kumar et al., 2022a,b). Briefly, patients were recruited, and baseline stool samples collected, if they had a 75-selenium homocholic acid taurine (^75^SeHCAT) scan requested by their gastroenterologist for symptoms of ongoing diarrhoea. Recruitment occurred from two district general hospitals and one tertiary centre. Diarrhoea was defined as the persistent alteration from the patient’s norm with stool consistency between types 5 and 7 on the Bristol stool chart and increased frequency greater than 4-weeks’ duration (Arasaradnam et al., 2018). All patients were seen in secondary care and investigations for diarrhoea were at the discretion of their primary Gastroenterologist. Patients were excluded from the study if they were: pregnant or breast feeding; unable to provide written consent; known established BAD; currently or previously treated with bile acid sequestrants; or recipients of antibiotics within 4 weeks of the initial trial participation.

Recruited study patients were categorised into four groups: Idiopathic BAD (^75^SeHCAT positive), post-operative terminal ileal resected Crohn’s disease, post-cholecystectomy and ^75^SeHCAT negative control group. As per the United Kingdom National Institute for Health and Care Excellence (NICE) guidance, a ^75^SeHCAT result of <5% was considered severe bile acid diarrhoea, 5–10% as moderate, 10–15% as mild and >15% as a negative result (DG44, 2021).

Patients with a positive ^75^SeHCAT result received a therapeutic trial of bile acid sequestrants with colesevelam 625 mg once or twice daily offered as first-line treatment. Patients were reviewed in a research clinic 4- and 8-weeks after treatment commencement and assessment of response was made at each review. Patients were required to complete a 7-day stool chart prior to their appointment where daily stool frequency and consistency (as per the Bristol Stool Form Scale) were documented. Stool samples were also collected at each clinic appointment. An early morning stool collection was advised, however depending on patient’s time and ability, a random stool sample was collected from any point in the day. Samples were immediately stored in −80°C after collection.

Clinical response was defined as patients who had improved bowel frequency by >50% from their initial assessment or <3 bowel movements per day. If patients had a partial response (defined as improved bowel frequency but not >50% or reduced bowel frequency but still >3 bowel movements/day), their colesevelam dose was increased at their clinic appointment and reviewed again in 4 weeks’ time. Any side effects of the treatment were documented, as well as review of their medication history. If patients could not tolerate the medication or no benefit was observed, they were subsequently withdrawn from the study, however their stool samples collected up to that point were still used for analysis. Of the post-operative Crohn’s disease cohort, those patients who had a primary terminal ileal resection and were diagnosed with bile acid diarrhoea within 12 months of their surgery were further reviewed at their 6–12 months colonoscopy appointment where stool samples were collected prior to bowel prep administration.

### DNA extraction and 16S rRNA amplicon sequencing

Microbial DNA was extracted from faecal samples according to the manufacturer’s instruction using the commercially available QIAamp Fast DNA Stool Mini Kit (Qiagen, United Kingdom). The extracted microbial DNA was then used for 16S ribosomal RNA (rRNA) gene amplification and sequencing to determine the mucosal-adherent microbiota as per the Earth Microbiome project protocol (Thompson et al., 2017). Commercially available primers were targeted to the V4 region (515F Parada: GTGYCAGCMGCCGCGGTAA, 806R Apprill: GGACTACNVGGGTWTCTAAT) and the 16S rRNA genes were amplified in triplicate. Each sample was amplified *via* polymerase chain reaction (PCR), with a unique ‘Earth Microbiome Project’ primer (16S Illumina Amplicon Protocol) that had a specific barcode to enable sample identification after sequencing. DNA extraction and 16S rRNA gene PCR were performed *via* paired-end sequencing (2 × 300 base pairs) using the MiSeq v2 Reagent kit and the Illumina MiSeq system (Illumina, San Diego, United States).

### Statistical analysis

Microbial bioinformatic analysis was performed using the Quantitative Insight into Microbial Ecology 2 (QIIME2) pipeline (Bolyen et al., 2019). Forward and reverse reads were assessed for quality using qiime demux summaries and trimmed using DADA2 to remove low-quality reads. Rarefaction plots were used to identify sequence sampling depth and α-and β-diversity was then estimated using the rarefied data. High-quality reads were clustered into amplicon sequence variant (ASVs) and classified using the SILVA 16S rRNA gene database using DADA2’s default parameters.

The Shannon diversity index and the Faith’s phylogenetic diversity was used to assess α-diversity metrics comparing intra-sample variability. Shannon α-diversity metric accounts for both the richness (total number of species within the community) and the evenness (relative abundance of different species), whereas Faith’s phylogenetic diversity is a measure of the biodiversity that incorporates phylogenetic differences between species. Statistical analysis for intra-sample comparisons (including pairwise and longitudinal analysis) was assessed using the non-parametric Kruskal–Wallis test. The Bray–Curtis dissimilarity distance matrix was used to assess β-diversity comparing inter-sample variability. Longitudinal analysis of subjects comparing timepoints following colesevelam treatment was performed using MaAsLin 2 (Microbiome Multivariable Associations with Linear Models; Mallick et al., 2021). Statistical analysis was performed using the permutational multivariate analysis of variance (PERMANOVA).

Comparison of relative abundances of taxa between the different group cohorts was performed using a linear discriminant analysis (LDA) effect size (LEfSe; Segata et al., 2011). Taxa with an LDA score >2 with a *p*-value ≤0.05 was considered statistically significant. Corrected *q*-values to adjust for the false discovery rate was calculated for multiple hypothesis testing between treatment groups and a *q*-value ≤0.05 was considered statistically significant (Storey and Tibshirani, 2003).

## Results

### Study population

A total of 257 samples from 135 patients were analysed in this study. A total of 135 baseline samples were collected pre-treatment, 57 samples from 4-weeks post-treatment, 54 samples from 8-weeks post-treatment and 11 samples from 6 to 12 months post-treatment. A total of 26 samples were excluded as these patients had an indeterminate diagnosis of BAD with a ^75^SeHCAT result between 15 and 20%. Although they were given a trial of treatment, there is currently not enough evidence to confidently state that patients would clinically improve with a borderline test result (DG44, 2021) and thus the decision was made to exclude these patients from the final microbial analysis. A total of 60 samples from 48 patients were not obtained during the study duration (27 from 4-weeks post-treatment, 30 samples from 8-weeks post-treatment and three samples from 6–12 months post-treatment) due to; patient withdrawal from adverse effects (*n* = 8), patient withdrawal, unknown reason (*n* = 34), not attending clinic appointments at specific timepoints in study (*n* = 12) and unable to collect samples due to national SARS-CoV2 virus lockdown restrictions (*n* = 6). Faecal sample collection details from each patient cohort is described in detail in Supplementary Table 1 and patient demographics can be viewed in Supplementary Table 2. Additional information on stool frequency and type as per the Bristol Stool Chart before and after treatment is also documented in Supplementary Table 3.

A total of 30.7 million reads was obtained following quality control with an average of 132,678 reads/sample (standard deviation of 71,210 reads/sample). A subsampling depth of 5,044 reads/sample was chosen following rarefaction.

### Bile acid diarrhoea is associated with changes in microbial diversity

**Figure 1:**
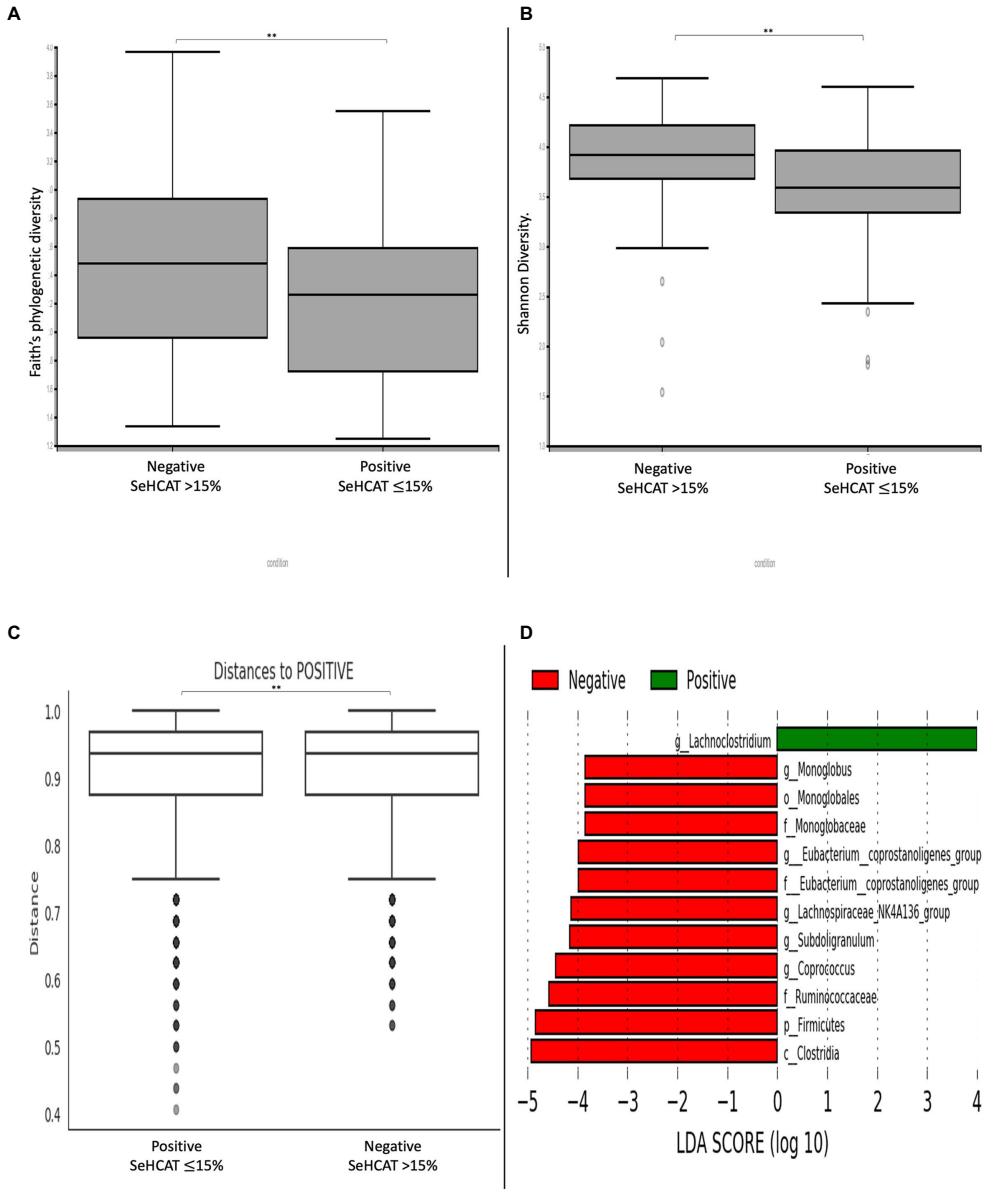
Changes in diversity and microbial composition in patients with and without BAD. α-diversity metrics **(A)** Faith’s phylogenetic diversity. **(B)** Shannon diversity. **(C)** β-diversity demonstrated by Bray-curtis distance matrix shows significantly distinct bacterial clusters between positive and negative BAD patients. **(D)** LEfSE histogram of LDA scores for differentially abundant bacterial taxa between positive and negative patients with BAD. Taxa highlighted in green was more abundant in patients with BAD and red in patients without BAD. Taxa with an LDA  > 2 with a *p*-value  ≤ 0.05 was considered statistically significant. **p-value < 0.01.

Comparison of α-diversity metrics demonstrates that patients with a positive diagnosis of BAD have a significantly lower bacterial diversity (Faith’s phylogenetic diversity, Shannon diversity) relative to those with a negative diagnosis (Figures 1A,B; *p* < 0.01). Consistent with this, the Bray-Curtis distance matrix assessed β-diversity between the two groups and showed that patients with a positive diagnosis of BAD formed significantly different bacterial community clusters from those with a negative diagnosis of BAD (Figure 1C; *p* = 0.007). To rank the greatest differences of abundant genera between positive and negative patients, the linear discriminant analysis effect size (LEfSe) was used (Figure 1D). Patients with a positive diagnosis of BAD showed a greater abundance of the *Lachnoclostridium* genus. Patients with a negative diagnosis of BAD (SeHCAT >15%) showed a greater abundance of the *Firmicutes* phylum; *Clostridia* class; *Monoglobales* order; *Monoglobaceae, Eubacterium coprostanoligenes* and *Ruminococcaceae* families; and *Monoglobus, Eubacterium coprostanoligenes, Lachnospiraceae NK4A136, Subdoligranulum* and *Coprococcus* genera.

### Bile acid disease severity is associated with a reduction in microbial diversity

**Figure 2:**
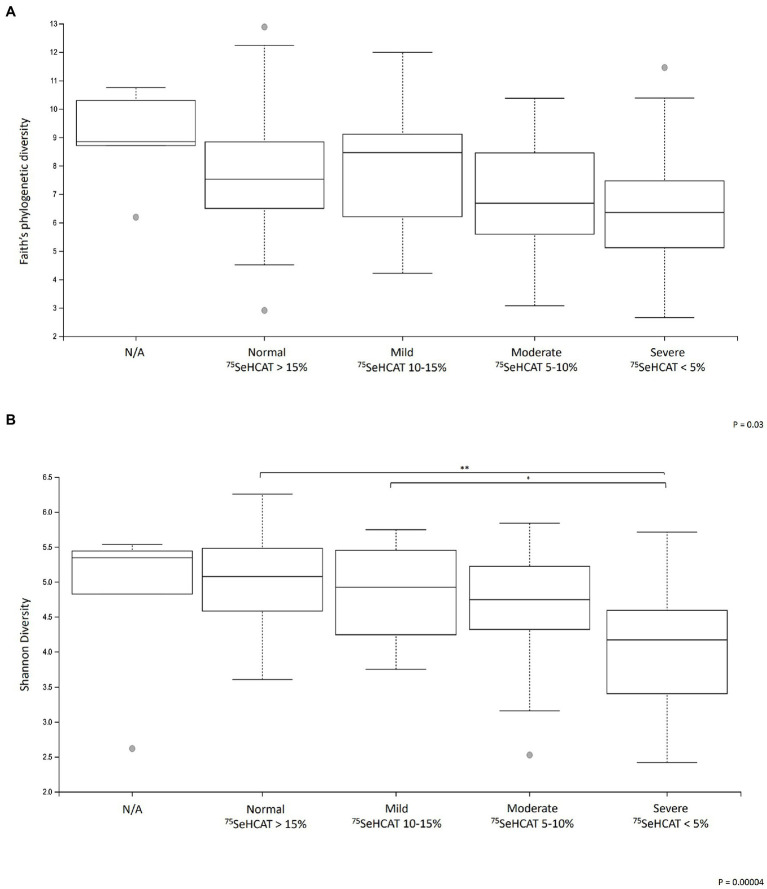
Changes in α-diversity metrics **(A)** Faith’s phylogenetic diversity and **(B)** Shannon diversity in patients with different degrees of severity of BAD. *q < 0.05 and **q < 0.001. N/A: Patients who did not undergo a SeHCAT scan but provided a baseline stool sample (*n* = 12).

A sub-analysis comparing the different degrees of severity of BAD showed that there were significant differences in α-diversity between the groups (Faith’s phylogenetic diversity *p* < 0.05, Shannon diversity *p* < 0.001; Figures 2A,B). Within the groups, Shannon diversity showed that patients with severe BAD (SeHCAT <5%) had significantly lower α-diversity relative to patients with mild BAD (SeHCAT 10–15%; *q* = 0.02) and those with a negative diagnosis (SeHCAT >15%; *q* < 0.01). There were no significant changes in β-diversity or microbial taxa.

### Type 2 BAD is associated with reduced bacterial diversity

**Figure 3:**
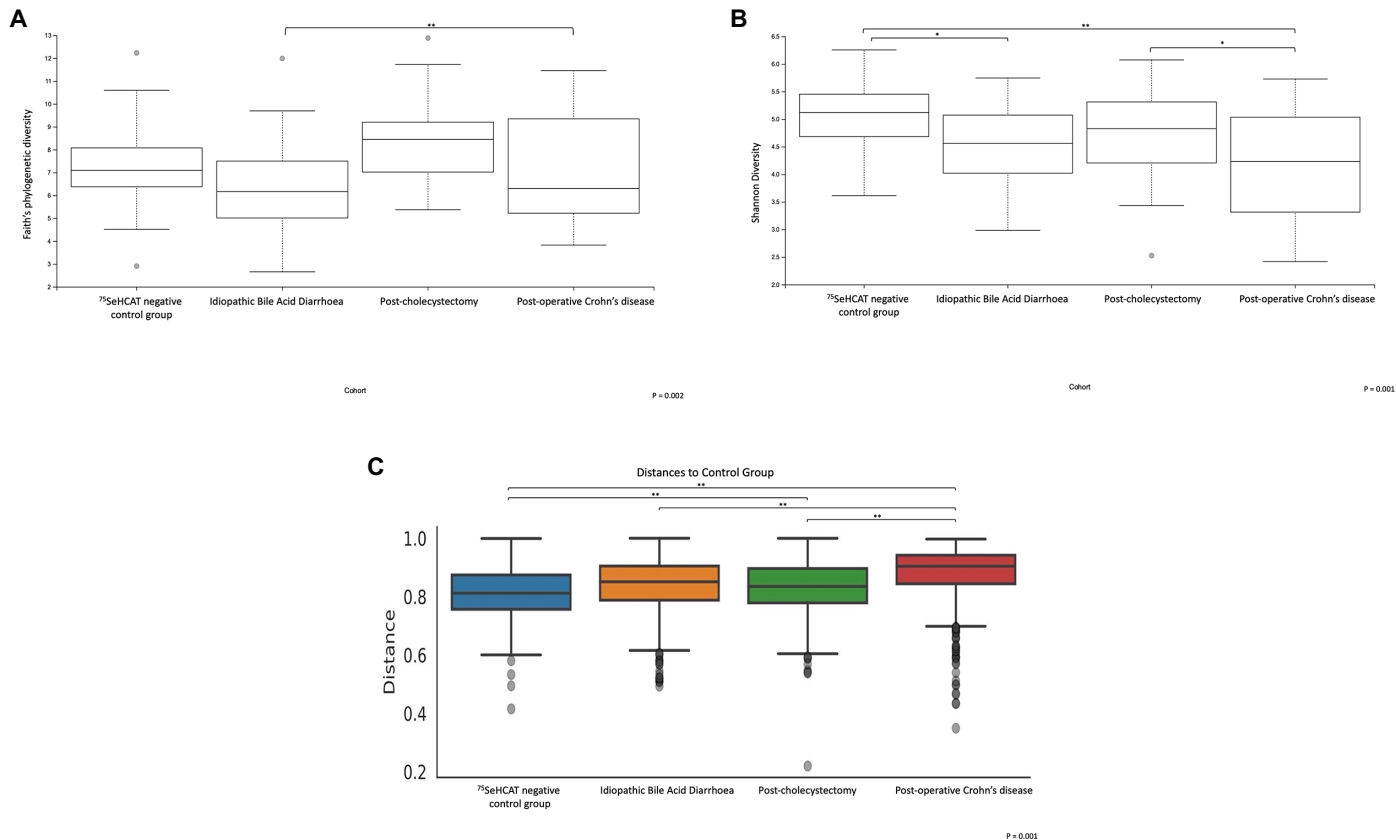
α and β-diversity metrics are significantly different between the 4 group cohorts. α-diversity metrics **(A)** Faith’s phylogenetic diversity, **(B)** Shannon diversity. **(C)** Bray-Curtis β-diversity distance matrix metric. *q < 0.05, **q < 0.001.

A further sub-analysis comparing the different group cohorts demonstrated significant differences in α-diversity (Faith’s phylogenetic diversity *p* = 0.002, Shannon diversity *p* = 0.001; Figures 3A,B). A pairwise permanova comparison between the groups demonstrated that patients with idiopathic Type 2 BAD had significantly lower α-diversity relative to type 1 post-operative Crohn’s disease patients (Faith’s PD, *q* < 0.001) and to the control group (Shannon diversity, *q* = 0.01). The post-operative Crohn’s disease cohort also demonstrated significantly lower α-diversity relative to type 3 post-cholecystectomy (Shannon diversity, *q* = 0.048) and control group cohort (Shannon diversity, *q* = 0.003). Each of the patient cohorts was significantly different based on pairwise β-diversity metrics compared to healthy controls (Figure 3C; *p* = 0.001, *q* < 0.01). There were no significant changes in microbial taxa between the group cohorts.

### Colesevelam does not alter bacterial diversity but affects microbial taxonomic profile

**Figure 4:**
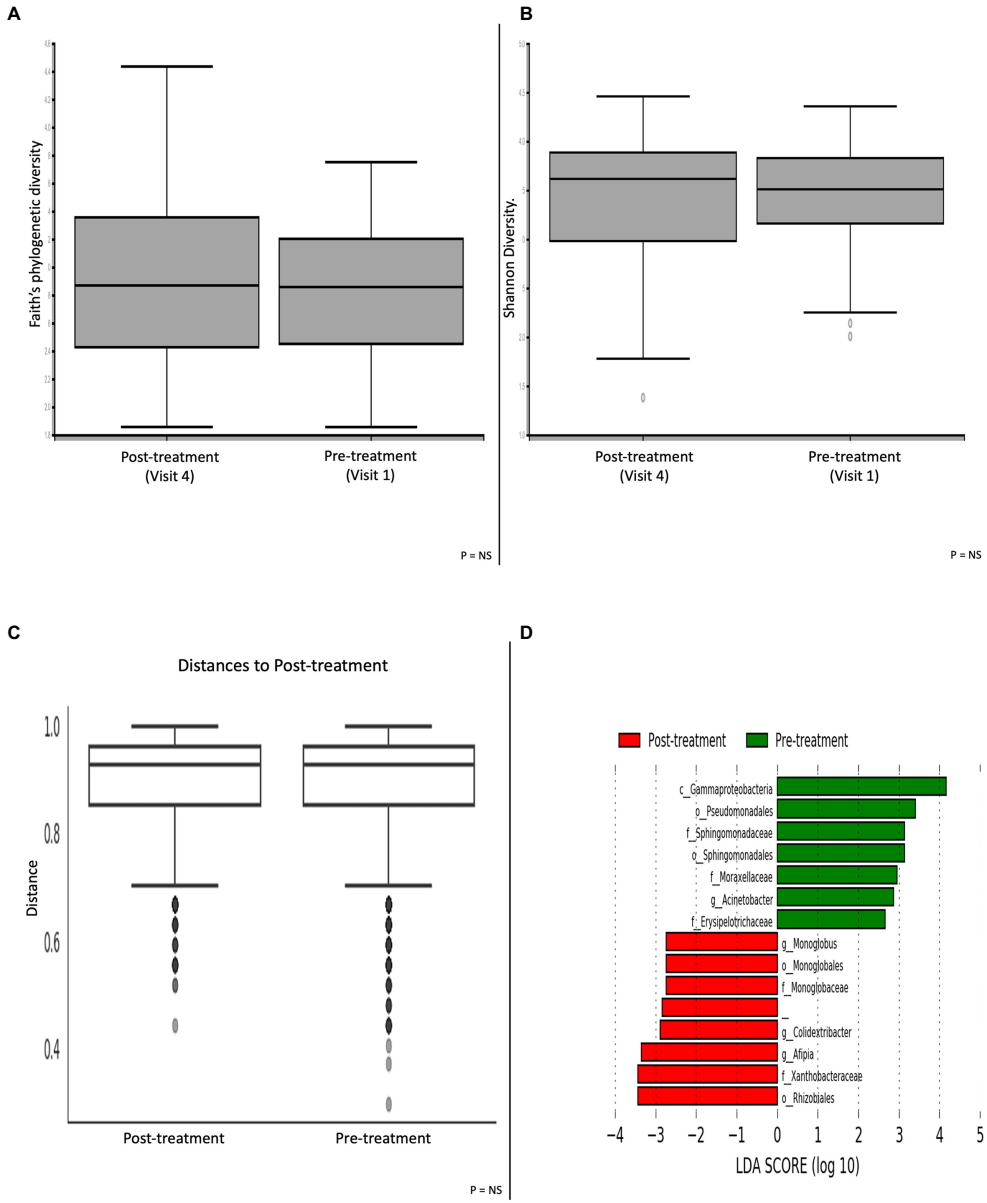
Microbial differences comparing patients pre-and post-treatment with the bile acid sequestrant colesevelam. α-diversity metric **(A)** Faith’s phylogenetic diversity and **(B)** Shannon diversity. **(C)** β-diversity Bray-Curtis distance matrix metric. **(D)** LEfSE histogram of LDA scores for differentially abundant bacterial taxa between pre-and post-treatment patients. Taxa highlighted in green was more significant in pre-treatment patients and red in post-treatment patients. Taxa with an LDA >2 with a *p*-value ≤0.05 was considered statistically significant.

A cross-sectional comparison with paired pre-and post-treatment groups did not show any significant differences in α-and β-diversity metrics (Figures 4A–C). Patients prior to having treatment showed an enrichment of the *Gammaproteobacteria* class; *Pseudomonadales* and *Sphingomonadales* order; *Sphingomonadaceae, Moraxellaceae* and E*rysipelotrichaceae* families and the *Acinetobacter* genus. Following treatment with colesevelam, there was a greater abundance of the *Monoglobales* and *Rhizobiales* orders; *Monoglobaceae* and *Xanthobacteraceae* families; and the *Monoglobus, Colidextribacter* and *Afipia* genera (Figure 4D).

Longitudinal analysis comparing baseline and week 4 of colesevelam treatment did not reveal any significant change in stool microbial profiles in the entire treated cohort as well as within each subgroup. However, longitudinal analysis comparing baseline and week 8 of colesevelam treatment showed a significant change in specific microbial taxa of the colesevelam treated cohort at week 8 compared to baseline (Supplementary Figure 1). These included an increase in abundance of genus *Monoglobus* and *Colidextribacter* and decrease in family *Enterobacteriaceae* (FDR corrected *p* ≤ 0.1). Subgroup analysis demonstrated that these significant taxonomic shifts at week 8 were only observed in the idiopathic BAD cohort with a decrease in *Prevotella* genus and an increase in the *Monoglobus* and *Eubacterium xylanophilum* groups, and genera belonging to family *Oscillospiraceae* (False discovery rate corrected *p* ≤ 0.1; Supplementary Figure 2). No significant stool microbial taxonomic changes were demonstrated at month 6 compared to baseline in the post-operative Crohn’s treated cohort.

### Bacterial diversity predicts treatment response

**Figure 5:**
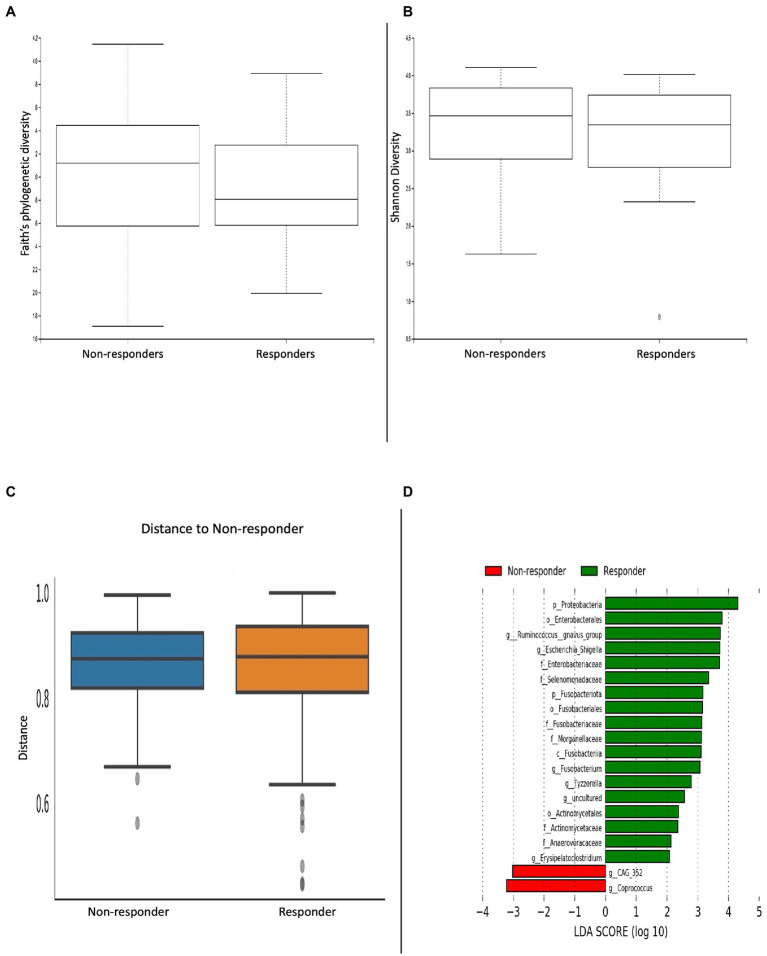
Microbial differences comparing clinical responders versus non-responders. α-diversity metrics **(A)** Faith’s phylogenetic diversity and **(B)** Shannon diversity were non-significant. **(C)** Bray-curtis distance matrix β-diversity metric was non-significant. **(D)** LEfSE histogram of LDA scores for differentially abundant bacterial taxa between responders and non-responders. Taxa highlighted in green was more significant in responders and red in non-responders. Taxa with an LDA >2 with a *p*-value ≤0.05 was considered statistically significant.

Following on from our treatment analysis, we then performed a sub-analysis comparing differences in α/β-diversity metrics between clinical responders (*n* = 81) and non-responders (*n* = 27; Figures 5A–C). Microbial analyses was done on faecal samples following 8-weeks of treatment. There were no significant differences in α/β-diversity metrics. Patients who clinically responded to treatment had a greater abundance of the *Proteobacteria* and F*usobacteria* phyla; *Fusobacteria* class; *Enterobacterales, Fusobacteriales* and *Actinomycetales* orders; *Enterobacteriaceae, Selenmonadaceae, Fusobacteriaceae, Morganellaceae, Actinomycetaceae* and *Anaerovoracaceae* families; *Ruminococcus gnavus, Escherichia/Shigella, Fusobacterium, Tyzzerella* and *Erysipelatoclostridium* genera. Conversely, patients who did not respond to treatment had an abundance of the *CAG 352* and *Coprococcus* genera (Figure 5D).

## Discussion

To the best of our knowledge, this is the first study to explore microbial changes in BAD based on both classification and severity of disease, whilst providing key information on the impact of or response to bile acid sequestrants. Similar to Sagar et al. (2020), we demonstrated a significant reduction in α-and β-diversity in patients with a positive diagnosis of BAD compared to patients who were not diagnosed with BAD. Our study, however, further illustrated that patients with severe BAD had significantly reduced microbial diversity compared to patients with mild disease and a normal ^75^SeHCAT scan. Moreover, out of the four different group cohorts, the idiopathic BAD group was found to have the most significant reduction in α-and β-diversity. Interestingly, Camilleri et al. (2022) also found reduced α-diversity with a different compositional profile based on β-diversity in patients with BAD whilst Jeffery et al. (2020) found a significant reduction in microbiota in patients with severe disease. However, neither study explored treatment response or compared between the sub-group classifications of BAD, which would have been pertinent considering Camilleri’s study included 15 out of 43 patients with a cholecystectomy.

Initially, idiopathic BAD was considered a result of impaired bile acid absorption. However, in the early 1990s, van Tilburg et al. (1992) demonstrated that the mean bile acid pool in idiopathic BAD was larger than in controls. Since then, the fibroblast growth factor-19 (FGF-19) was discovered to play a role in the negative feedback regulation of the enterohepatic circulation and hepatic bile acid synthesis (Inagaki et al., 2005). Recent data has shown that patients with idiopathic BAD have reduced FGF-19 levels compared to controls (Walters et al., 2009). Thus, this disrupted feedback control by FGF-19 in idiopathic BAD results in a triad of excessive bile acid production, incomplete absorption and excess faecal bile acid loss. This mechanism differs from patients with post-operative Crohn’s disease where the main site of bile acid absorption is removed and in post-cholecystectomy patients where the storage and concentration of bile acids are removed, with both processes augmenting an increase in primary bile acids entering the colon (Housset et al., 2016). Whilst even a small fluctuation in bile acids can trigger major alterations in bacterial community structures (Buffie et al., 2015), the significantly reduced diversity seen in the idiopathic BAD group from our study may be a result of either an excess of bile acid content in the colon or potentially the elimination of slower-growing bacteria in response to diarrhoea. Bile acids are known to inhibit the growth of many bacteria *via* their detergent-like actions (Urdaneta and Casadesus, 2017; Tian et al., 2020); thus, the excessive faecal bile content may be detrimental for bacterial growth both directly (antimicrobial) and indirectly (diarrhoea). We are, however, unable to account for why reduced diversity was not seen in equal measure in the post-cholecystectomy and Crohn’s disease cohorts despite a recent study demonstrating reduced diversity in the post-cholecystectomy cohort compared to non-post-cholecystectomy patients and healthy controls (Xu et al., 2022). Further research is needed to explore the relationship between bile acids, bile acid receptors such as the farnesoid X receptor (FXR) and FGF-19 and their interaction with the microbiome to understand the pathophysiology of disease underpinning these different cohorts.

The diagnosis of BAD is associated with an increase in unconjugated primary bile acids due to reduced biotransformation of primary to secondary bile acids (Ridlon et al., 2006; Winston and Theriot, 2020). In patients with a normal ^75^SeHCAT scan, we found an abundance of *Clostridia, Firmicutes* and *Ruminococcus* bacterium, which are crucial in expressing enzymes for 7-α-dehydroxylation to secondary bile acids in the large intestine. It was surprising, however, to find an increase in the *Lachnoclostridium* species in patients with a positive diagnosis of BAD in our study. The *Lachnoclostridium* genus is a relatively newly defined genus under the *Clostridia* class and includes organisms from the *Lachnospiraceae* family and several *Clostridial* clusters including *Clostridium XIVa* (Yutin and Galperin, 2013). This genus, along with *Clostridoides* sp. and *Eggerthella,* is known to be part of the bile acid-inducible (bai) gene cluster for the multistep 7α/β-dehydroxylation pathway, which aids in the conversion of primary to secondary bile acids (Heinken et al., 2019). This increased abundance was also seen by Sagar et al. (2020) who correspondingly found an increase in secondary bile acid concentration in their BAD cohort. Whether this is a consequence of the higher concentrations of primary bile acids entering the colon rather than an outcome of the disease itself is unknown and may indicate that the pathophysiology of BAD can differ depending on the classification of BAD.

Our novel study explored microbial changes following treatment with colesevelam in patients with BAD. Although our results did not show any significant change in stool microbial profiles after 4 weeks of treatment with colesevelam, specific microbial taxonomic shifts were seen in the treated cohort at week 8, signifying that longer treatment durations are needed to demonstrate microbial effect. Our subgroup analysis also showed that these taxonomic shifts were only observed in the idiopathic BAD cohort, which may explain why changes were not seen at 6-months in our post-operative Crohn’s cohort. Specifically, the treated cohort had an abundance of genus belonging to the *Oscillospiraceae* family, including *Monoglobus* and *Colidextribacter*. Information on the role of these bacteria with bile acid modulation is limited and thus would be a target of interest for future research.

Treatment response to BAD with bile acid sequestrants is variable. Colestyramine is the first-line licenced treatment for BAD but is poorly tolerated due to its texture and taste and numerous gastrointestinal related side effects. Response to treatment is estimated to be between 70 and 90% depending on severity of disease (Barkun et al., 2013; Riemsma et al., 2013). Colesevelam is currently unlicensed but is better tolerated and has a greater affinity for binding bile acids (Wedlake et al., 2009). Treatment response with colesevelam is variable with one small study demonstrating 70% of patients improving with treatment (>30% reduction in number of liquid stools/day after 4 weeks; Beigel et al., 2014). A recent study by Vijayvargiya et al. did not show any change in stool frequency, consistency or colonic transit time in colesevelam treated patients with IBS-D with increased bile acid synthesis or faecal excretion. Our recently published small study of 47 patients with established BAD on ^75^SeHCAT demonstrated modest improvement with colesevelam of 55%, with a greater response in patients with Crohn’s disease (82%) and those with severe disease (75%) (Kumar et al., 2022b). As there is such variability in treatment response, we explored whether any alterations in the microbiome was dependent upon response to colesevelam. This could help elucidate alternative targets to predict treatment response or indicate a signal response where colesevelam had not altered the bile acid pool, which may have implications for future disease recurrence. Of interest, we showed that patients who responded to treatment had a greater abundance in *Fusobacteria* and *Ruminococcus*, both of which were found in abundance in patients with a normal ^75^SeHCAT scan. *Ruminococcus* has also been consistently associated with firmer stools and a longer colonic transit time (Falony et al., 2016; Vandeputte et al., 2016; Asnicar et al., 2021; Steenackers et al., 2022). These findings signify a possible association with colesevelam in clinical responders leading to a change in microbiome to reverse the underlying mechanism of BAD.

Our study has several limitations. Firstly, the 16S rRNA sequencing does not provide functional information which is needed to better understand host–microbe interactions relevant to states of health and disease. We also did not control for other confounding factors when analysing the microbiome such as diet, medications, smoking status and past medical and surgical history. Subsequently, we can only form associations rather than cause or consequence. However, Zhernakova et al. (2016) recently demonstrated that microbial diversity is associated with 126 exogenous and intrinsic host factors, including 31 intrinsic factors, 12 diseases, 19 drug groups, four smoking categories and 60 dietary factors. Therefore, it may not be possible to control for each and every confounding factor. A further limitation in our study is the lack of correlation between bile acid composition and microbial changes. Future studies should involve the study of metagenomics, meta-transcriptomics and metabolomics to better understand the complex relationship between bile acids, bile acid receptors and the microbiome. This would include correlating faecal bile acid measurements with microbial analysis. Lastly, this study did not examine the role of other microbial kingdoms such as fungi and viruses, and their function in the bile acid pathway is currently unknown.

## Conclusion

This novel study is the first to explore microbial diversity comparing the different classifications and severity of BAD, demonstrating reduced diversity in patients with severe BAD and in the idiopathic cohort. It is also the first study to examine treatment effects on the microbiome and we were able to demonstrate a possible association with colesevelam on the microbiome, which was most discernible in our idiopathic BAD cohort. Whilst the data pool is small and exploratory only, the results are still noteworthy to consider developing a larger mechanistic study that would accommodate the heterogenous response to bile acid sequestrants and explore the impact of microbiome manipulation on the prevention of disease recurrence.

## Data availability statement

The datasets presented in this study have been deposited in the NCBI repository, accession number PRJNA941862.

## Ethics statement

The studies involving human participants were reviewed and approved by London-Stanmore Research Ethics Committee. REC ref.: 16/LO/1325. The patients/participants provided their written informed consent to participate in this study.

## Author contributions

AK led the study, recruited, collected data, and analysed the results. She wrote the first draft of the manuscript. NQ, ME and AB aided with data analysis. MJ, HS, JB, and AF helped with study recruitment. JM, HA-H, and MB conceptualised and designed the study. AK, NQ, AB, HA-H, JS, and MB were involved with critical revisions of the manuscript. All authors contributed to the article and approved the submitted version.

## Funding

The research department of MB received project funding from Bowel and Cancer Research for part of this work. The research department of MB received project funding from an unrestricted grant from Tillotts Pharma for part of this work.

## Conflict of interest

MB has received grants and travel expenses from Vifor International and Tillotts Pharma, outside of the submitted work. The research department of MB also received funding from Tillotts Pharma to support part of the described work. HS has received travel and conference expenses from Tillotts Pharma, Norgine, MSD, Abbvie and Janssen outside of the submitted work. JS has received speaker fees for Abbvie, Takeda and Janssen outside of the submitted work.

The remaining authors declare that the research was conducted in the absence of any commercial or financial relationships that could be construed as a potential conflict of interest.

## Publisher’s note

All claims expressed in this article are solely those of the authors and do not necessarily represent those of their affiliated organizations, or those of the publisher, the editors and the reviewers. Any product that may be evaluated in this article, or claim that may be made by its manufacturer, is not guaranteed or endorsed by the publisher.
