# Supplementary figures and images for: The analysis of gut microbiota in patients with bile acid diarrhoea treated with colesevelam
Source: Front Microbiol. 2023 Mar 17;14:1134105. doi: 10.3389/fmicb.2023.1134105 (PMC10063896; doi:10.3389/fmicb.2023.1134105)

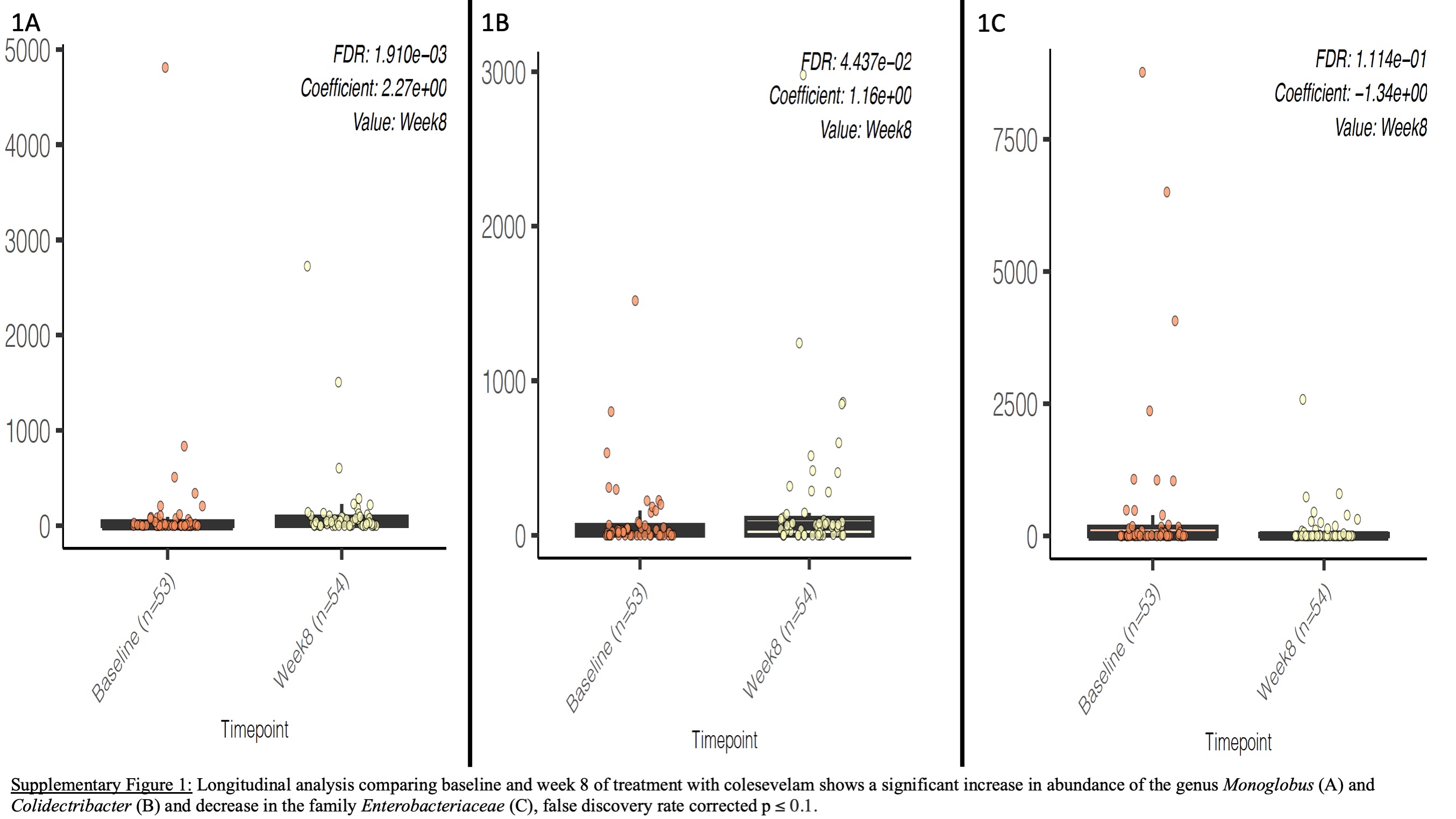

Supplement: Supplementary file 1 [file Image_1.JPEG]

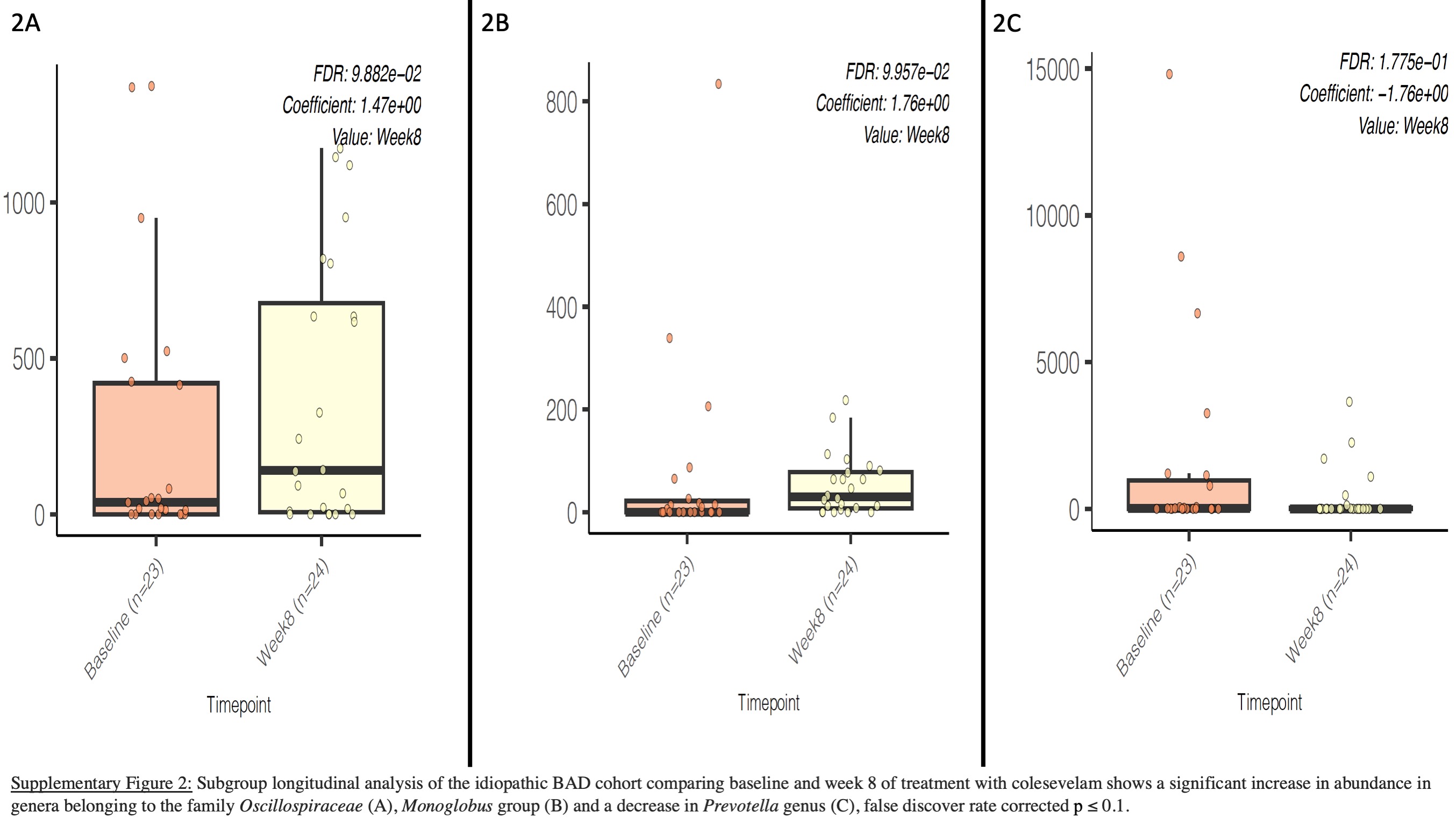

Supplement: Supplementary file 2 [file Image_2.JPEG]
