# Supplementary material for: The analysis of gut microbiota in patients with bile acid diarrhoea treated with colesevelam
Source: Front Microbiol. 2023 Mar 17;14:1134105. doi: 10.3389/fmicb.2023.1134105 (PMC10063896; doi:10.3389/fmicb.2023.1134105)
Supplement: Supplementary file 3 [file Data_Sheet_1.PDF]

	SubjectID1	SubjectID2	Group1	Group2	Distance
0	B66	B27	Control group	Control group	0.8403890160183066
1	B86	B27	Control group	Control group	0.7744088482074752
2	B86	B66	Control group	Control group	0.770976353928299
3	B97	B27	Control group	Control group	0.7242562929061785
4	B97	B66	Control group	Control group	0.6618993135011442
5	B97	B86	Control group	Control group	0.7437070938215103
6	B98	B27	Control group	Control group	0.7057589626239512
7	B98	B66	Control group	Control group	0.7725019069412662
8	B98	B86	Control group	Control group	0.812929061784897
9	B98	B97	Control group	Control group	0.6647597254004577
10	B100	B27	Control group	Control group	0.938977879481312
11	B100	B66	Control group	Control group	0.8115942028985508
12	B100	B86	Control group	Control group	0.9242944317315027
13	B100	B97	Control group	Control group	0.9166666666666666
14	B100	B98	Control group	Control group	0.936880244088482
15	B112	B27	Control group	Control group	0.7581998474446987
16	B112	B66	Control group	Control group	0.8459191456903128
17	B112	B86	Control group	Control group	0.6700991609458429
18	B112	B97	Control group	Control group	0.8032036613272311
19	B112	B98	Control group	Control group	0.801487414187643
20	B112	B100	Control group	Control group	0.9723493516399695
21	B115	B27	Control group	Control group	0.7170099160945843
22	B115	B66	Control group	Control group	0.7841342486651411
23	B115	B86	Control group	Control group	0.8033943554538521
24	B115	B97	Control group	Control group	0.6826849733028223
25	B115	B98	Control group	Control group	0.6996567505720824
26	B115	B100	Control group	Control group	

0.9391685736079328
27	B115	B112	Control group	Control group
0.8573607932875668
28	I1	B27	Control group	Control group
0.708047292143402
29	I1	B66	Control group	Control group
0.697177269260107
30	I1	B86	Control group	Control group
0.7454233409610984
31	I1	B97	Control group	Control group
0.6735316552250191
32	I1	B98	Control group	Control group
0.6003051106025934
33	I1	B100	Control group	Control group
0.9036994660564455
34	I1	B112	Control group	Control group
0.7831807780320366
35	I1	B115	Control group	Control group
0.6937452326468345
36	I3	B27	Control group	Control group
0.8470633104500381
37	I3	B66	Control group	Control group
0.7740274599542334
38	I3	B86	Control group	Control group
0.8314263920671243
39	I3	B97	Control group	Control group
0.6895499618611747
40	I3	B98	Control group	Control group
0.780511060259344
41	I3	B100	Control group	Control group
0.9286803966437833
42	I3	B112	Control group	Control group
0.8659420289855072
43	I3	B115	Control group	Control group
0.8133104500381388
44	I3	I1	Control group	Control group
0.7631578947368421
45	I6	B27	Control group	Control group
0.7601067887109078
46	I6	B66	Control group	Control group
0.6819221967963387
47	I6	B86	Control group	Control group
0.7850877192982456
48	I6	B97	Control group	Control group
0.6348207475209764
49	I6	B98	Control group	Control group
0.6800152555301296
50	I6	B100	Control group	Control group
0.948512585812357
51	I6	B112	Control group	Control group
0.8281845919145691
52	I6	B115	Control group	Control group
0.6399694889397407
53	I6	I1	Control group	Control group

					0.6683829138062548
54	I6	I3	Control group	Control group	0.7143401983218917
55	I8	B27	Control group	Control group	0.7911899313501144
56	I8	B66	Control group	Control group	0.8585049580472921
57	I8	B86	Control group	Control group	0.7757437070938215
58	I8	B97	Control group	Control group	0.7379862700228833
59	I8	B98	Control group	Control group	0.7942410373760488
60	I8	B100	Control group	Control group	0.9500381388253242
61	I8	B112	Control group	Control group	0.8331426392067124
62	I8	B115	Control group	Control group	0.7158657513348589
63	I8	I1	Control group	Control group	0.6893592677345538
64	I8	I3	Control group	Control group	0.8342868039664378
65	I8	I6	Control group	Control group	0.7719298245614035
66	I10	B27	Control group	Control group	0.8768115942028986
67	I10	B66	Control group	Control group	0.8264683447749809
68	I10	B86	Control group	Control group	0.7761250953470633
69	I10	B97	Control group	Control group	0.7440884820747521
70	I10	B98	Control group	Control group	0.7936689549961862
71	I10	B100	Control group	Control group	0.9464149504195271
72	I10	B112	Control group	Control group	0.8983600305110603
73	I10	B115	Control group	Control group	0.7757437070938215
74	I10	I1	Control group	Control group	0.7549580472921434
75	I10	I3	Control group	Control group	0.7578184591914569
76	I10	I6	Control group	Control group	0.8342868039664378
77	I10	I8	Control group	Control group	0.6855453852021358
78	I11	B27	Control group	Control group	0.7911899313501144
79	I11	B66	Control group	Control group	0.8743325705568269
80	I11	B86	Control group	Control group	

0.8951182303585049
81 I11 B97 Control group Control group
0.757627765064836
82 I11 B98 Control group Control group
0.7915713196033562
83 I11 B100 Control group Control group
0.9170480549199085
84 I11 B112 Control group Control group
0.9500381388253242
85 I11 B115 Control group Control group
0.847254004576659
86 I11 I1 Control group Control group
0.8199847444698704
87 I11 I3 Control group Control group
0.8026315789473685
88 I11 I6 Control group Control group
0.7906178489702517
89 I11 I8 Control group Control group
0.8697559115179252
90 I11 I10 Control group Control group
0.8802440884820748
91 I13 B27 Control group Control group
0.814836003051106
92 I13 B66 Control group Control group
0.8115942028985508
93 I13 B86 Control group Control group
0.7702135774218154
94 I13 B97 Control group Control group
0.719488939740656
95 I13 B98 Control group Control group
0.7761250953470633
96 I13 B100 Control group Control group
0.9620518688024409
97 I13 B112 Control group Control group
0.8749046529366895
98 I13 B115 Control group Control group
0.7707856598016781
99 I13 I1 Control group Control group
0.7612509534706331
100 I13 I3 Control group Control group
0.8062547673531655
101 I13 I6 Control group Control group
0.7631578947368421
102 I13 I8 Control group Control group
0.7151029748283753
103 I13 I10 Control group Control group
0.7763157894736842
104 I13 I11 Control group Control group
0.8695652173913043
105 I15 B27 Control group Control group
0.8985507246376812
106 I15 B66 Control group Control group
0.7397025171624714
107 I15 B86 Control group Control group

0.8670861937452327
108 I15 B97 Control group Control group
0.772883295194508
109 I15 B98 Control group Control group
0.8636536994660564
110 I15 B100 Control group Control group
0.9614797864225781
111 I15 B112 Control group Control group
0.9176201372997712
112 I15 B115 Control group Control group
0.8287566742944318
113 I15 I1 Control group Control group
0.8754767353165522
114 I15 I3 Control group Control group
0.9046529366895499
115 I15 I6 Control group Control group
0.8157894736842105
116 I15 I8 Control group Control group
0.9212433257055682
117 I15 I10 Control group Control group
0.8449656750572082
118 I15 I11 Control group Control group
0.9387871853546911
119 I15 I13 Control group Control group
0.778794813119756
120 I17 B27 Control group Control group
0.7242562929061785
121 I17 B66 Control group Control group
0.8804347826086957
122 I17 B86 Control group Control group
0.620137299771167
123 I17 B97 Control group Control group
0.805301296720061
124 I17 B98 Control group Control group
0.8266590389016019
125 I17 B100 Control group Control group
0.9548054919908466
126 I17 B112 Control group Control group
0.7116704805491991
127 I17 B115 Control group Control group
0.8524027459954233
128 I17 I1 Control group Control group
0.7511441647597255
129 I17 I3 Control group Control group
0.8070175438596491
130 I17 I6 Control group Control group
0.8604118993135011
131 I17 I8 Control group Control group
0.7566742944317315
132 I17 I10 Control group Control group
0.7873760488176964
133 I17 I11 Control group Control group
0.9014111365369947
134 I17 I13 Control group Control group

0.7858504958047292
135	I17	I15	Control group	Control group
0.9359267734553776
136	I18	B27	Control group	Control group
0.7999618611746758
137	I18	B66	Control group	Control group
0.801487414187643
138	I18	B86	Control group	Control group
0.7686880244088482
139	I18	B97	Control group	Control group
0.656559877955759
140	I18	B98	Control group	Control group
0.7412280701754386
141	I18	B100	Control group	Control group
0.9567124332570557
142	I18	B112	Control group	Control group
0.8381006864988558
143	I18	B115	Control group	Control group
0.7126239511823036
144	I18	I1	Control group	Control group
0.7820366132723112
145	I18	I3	Control group	Control group
0.7009916094584286
146	I18	I6	Control group	Control group
0.7707856598016781
147	I18	I8	Control group	Control group
0.772883295194508
148	I18	I10	Control group	Control group
0.7288329519450801
149	I18	I11	Control group	Control group
0.8331426392067124
150	I18	I13	Control group	Control group
0.7088100686498856
151	I18	I15	Control group	Control group
0.7991990846681922
152	I18	I17	Control group	Control group
0.818649885583524
153	I19	B27	Control group	Control group
0.7940503432494279
154	I19	B66	Control group	Control group
0.8956903127383676
155	I19	B86	Control group	Control group
0.6464530892448512
156	I19	B97	Control group	Control group
0.8552631578947368
157	I19	B98	Control group	Control group
0.8783371472158658
158	I19	B100	Control group	Control group
0.9662471395881007
159	I19	B112	Control group	Control group
0.6359649122807017
160	I19	B115	Control group	Control group
0.873951182303585
161	I19	I1	Control group	Control group

0.8611746758199847
162 I19 I3 Control group Control group
0.9113272311212814
163 I19 I6 Control group Control group
0.8350495804729214
164 I19 I8 Control group Control group
0.8495423340961098
165 I19 I10 Control group Control group
0.8979786422578184
166 I19 I11 Control group Control group
0.9309687261632341
167 I19 I13 Control group Control group
0.835812356979405
168 I19 I15 Control group Control group
0.9378337147215866
169 I19 I17 Control group Control group
0.6500762776506483
170 I19 I18 Control group Control group
0.8503051106025934
171 I22 B27 Control group Control group
0.7946224256292906
172 I22 B66 Control group Control group
0.7812738367658276
173 I22 B86 Control group Control group
0.7355072463768116
174 I22 B97 Control group Control group
0.7345537757437071
175 I22 B98 Control group Control group
0.7629672006102212
176 I22 B100 Control group Control group
0.9366895499618612
177 I22 B112 Control group Control group
0.8665141113653699
178 I22 B115 Control group Control group
0.7894736842105263
179 I22 I1 Control group Control group
0.7143401983218917
180 I22 I3 Control group Control group
0.7971014492753623
181 I22 I6 Control group Control group
0.7700228832951945
182 I22 I8 Control group Control group
0.7690694126620901
183 I22 I10 Control group Control group
0.7536231884057971
184 I22 I11 Control group Control group
0.8089244851258581
185 I22 I13 Control group Control group
0.7911899313501144
186 I22 I15 Control group Control group
0.8045385202135774
187 I22 I17 Control group Control group
0.7292143401983219
188 I22 I18 Control group Control group

0.704042715484363
189	I22	I19	Control group	Control group
0.8363844393592678
190	I23	B27	Control group	Control group
0.7635392829900839
191	I23	B66	Control group	Control group
0.717391304347826
192	I23	B86	Control group	Control group
0.7130053394355453
193	I23	B97	Control group	Control group
0.6905034324942791
194	I23	B98	Control group	Control group
0.658276125095347
195	I23	B100	Control group	Control group
0.9227688787185355
196	I23	B112	Control group	Control group
0.8470633104500381
197	I23	B115	Control group	Control group
0.7496186117467581
198	I23	I1	Control group	Control group
0.6352021357742181
199	I23	I3	Control group	Control group
0.7038520213577422
200	I23	I6	Control group	Control group
0.6937452326468345
201	I23	I8	Control group	Control group
0.7877574370709383
202	I23	I10	Control group	Control group
0.7458047292143402
203	I23	I11	Control group	Control group
0.860602593440122
204	I23	I13	Control group	Control group
0.790045766590389
205	I23	I15	Control group	Control group
0.877765064836003
206	I23	I17	Control group	Control group
0.7738367658276125
207	I23	I18	Control group	Control group
0.6756292906178489
208	I23	I19	Control group	Control group
0.8628909229595728
209	I23	I22	Control group	Control group
0.7427536231884058
210	I24	B27	Control group	Control group
0.6615179252479023
211	I24	B66	Control group	Control group
0.8493516399694889
212	I24	B86	Control group	Control group
0.532608695652174
213	I24	B97	Control group	Control group
0.7972921434019832
214	I24	B98	Control group	Control group
0.8371472158657514
215	I24	B100	Control group	Control group

0.9813119755911518
216	I24	B112	Control group	Control group
0.6903127383676583
217	I24	B115	Control group	Control group
0.8676582761250954
218	I24	I1	Control group	Control group
0.7778413424866514
219	I24	I3	Control group	Control group
0.8533562166285278
220	I24	I6	Control group	Control group
0.8524027459954233
221	I24	I8	Control group	Control group
0.8037757437070938
222	I24	I10	Control group	Control group
0.8234172387490465
223	I24	I11	Control group	Control group
0.937070938215103
224	I24	I13	Control group	Control group
0.7986270022883295
225	I24	I15	Control group	Control group
0.8914950419527079
226	I24	I17	Control group	Control group
0.6348207475209764
227	I24	I18	Control group	Control group
0.7999618611746758
228	I24	I19	Control group	Control group
0.6876430205949656
229	I24	I22	Control group	Control group
0.8384820747520977
230	I24	I23	Control group	Control group
0.8083524027459954
231	I25	B27	Control group	Control group
0.7507627765064836
232	I25	B66	Control group	Control group
0.7191075514874142
233	I25	B86	Control group	Control group
0.7372234935163997
234	I25	B97	Control group	Control group
0.6615179252479023
235	I25	B98	Control group	Control group
0.7185354691075515
236	I25	B100	Control group	Control group
0.9551868802440885
237	I25	B112	Control group	Control group
0.8230358504958047
238	I25	B115	Control group	Control group
0.6910755148741419
239	I25	I1	Control group	Control group
0.6554157131960335
240	I25	I3	Control group	Control group
0.7122425629290617
241	I25	I6	Control group	Control group
0.7070938215102975
242	I25	I8	Control group	Control group

0.6689549961861174
243 I25 I10 Control group Control group
0.631769641495042
244 I25 I11 Control group Control group
0.8346681922196796
245 I25 I13 Control group Control group
0.742372234935164
246 I25 I15 Control group Control group
0.8726163234172387
247 I25 I17 Control group Control group
0.7774599542334096
248 I25 I18 Control group Control group
0.6544622425629291
249 I25 I19 Control group Control group
0.8197940503432495
250 I25 I22 Control group Control group
0.7456140350877193
251 I25 I23 Control group Control group
0.7240655987795576
252 I25 I24 Control group Control group
0.7534324942791762
253 I26 B27 Control group Control group
0.8398169336384439
254 I26 B66 Control group Control group
0.6893592677345538
255 I26 B86 Control group Control group
0.7963386727688787
256 I26 B97 Control group Control group
0.7008009153318078
257 I26 B98 Control group Control group
0.7520976353928299
258 I26 B100 Control group Control group
0.9221967963386728
259 I26 B112 Control group Control group
0.852974828375286
260 I26 B115 Control group Control group
0.8030129672006102
261 I26 I1 Control group Control group
0.7372234935163997
262 I26 I3 Control group Control group
0.7492372234935164
263 I26 I6 Control group Control group
0.7547673531655225
264 I26 I8 Control group Control group
0.8504958047292144
265 I26 I10 Control group Control group
0.7231121281464531
266 I26 I11 Control group Control group
0.9427917620137299
267 I26 I13 Control group Control group
0.6292906178489702
268 I26 I15 Control group Control group
0.7227307398932112
269 I26 I17 Control group Control group

0.8651792524790236
270 I26 I18 Control group Control group
0.7406559877955758
271 I26 I19 Control group Control group
0.9092295957284515
272 I26 I22 Control group Control group
0.8375286041189931
273 I26 I23 Control group Control group
0.704233409610984
274 I26 I24 Control group Control group
0.8266590389016019
275 I26 I25 Control group Control group
0.7261632341723875
276 I27 B27 Control group Control group
0.847254004576659
277 I27 B66 Control group Control group
0.7482837528604119
278 I27 B86 Control group Control group
0.7639206712433257
279 I27 B97 Control group Control group
0.7868039664378337
280 I27 B98 Control group Control group
0.7553394355453852
281 I27 B100 Control group Control group
0.9483218916857361
282 I27 B112 Control group Control group
0.8457284515636918
283 I27 B115 Control group Control group
0.7528604118993135
284 I27 I1 Control group Control group
0.7932875667429443
285 I27 I3 Control group Control group
0.6882151029748284
286 I27 I6 Control group Control group
0.7090007627765065
287 I27 I8 Control group Control group
0.8297101449275363
288 I27 I10 Control group Control group
0.7398932112890922
289 I27 I11 Control group Control group
0.8741418764302059
290 I27 I13 Control group Control group
0.763348588863463
291 I27 I15 Control group Control group
0.8062547673531655
292 I27 I17 Control group Control group
0.8710907704042715
293 I27 I18 Control group Control group
0.580091533180778
294 I27 I19 Control group Control group
0.891113653699466
295 I27 I22 Control group Control group
0.7871853546910755
296 I27 I23 Control group Control group

0.6914569031273837
297	I27	I24	Control group	Control group
0.8056826849733029
298	I27	I25	Control group	Control group
0.7744088482074752
299	I27	I26	Control group	Control group
0.7259725400457666
300	I28	B27	Control group	Control group
0.876048817696415
301	I28	B66	Control group	Control group
0.7860411899313501
302	I28	B86	Control group	Control group
0.8514492753623188
303	I28	B97	Control group	Control group
0.7841342486651411
304	I28	B98	Control group	Control group
0.7883295194508009
305	I28	B100	Control group	Control group
0.9155225019069413
306	I28	B112	Control group	Control group
0.9153318077803204
307	I28	B115	Control group	Control group
0.8623188405797102
308	I28	I1	Control group	Control group
0.8516399694889397
309	I28	I3	Control group	Control group
0.7517162471395881
310	I28	I6	Control group	Control group
0.9073226544622426
311	I28	I8	Control group	Control group
0.9021739130434783
312	I28	I10	Control group	Control group
0.855072463768116
313	I28	I11	Control group	Control group
0.8119755911517925
314	I28	I13	Control group	Control group
0.8545003813882532
315	I28	I15	Control group	Control group
0.8348588863463006
316	I28	I17	Control group	Control group
0.9145690312738368
317	I28	I18	Control group	Control group
0.7446605644546148
318	I28	I19	Control group	Control group
0.952326468344775
319	I28	I22	Control group	Control group
0.8447749809305873
320	I28	I23	Control group	Control group
0.877765064836003
321	I28	I24	Control group	Control group
0.9031273836765827
322	I28	I25	Control group	Control group
0.7950038138825324
323	I28	I26	Control group	Control group

0.8297101449275363
324	I28	I27	Control group	Control group
0.7948131197559115
325	I29	B27	Control group	Control group
0.8697559115179252
326	I29	B66	Control group	Control group
0.7704042715484363
327	I29	B86	Control group	Control group
0.7829900839054157
328	I29	B97	Control group	Control group
0.7852784134248665
329	I29	B98	Control group	Control group
0.8524027459954233
330	I29	B100	Control group	Control group
0.9645308924485125
331	I29	B112	Control group	Control group
0.8670861937452327
332	I29	B115	Control group	Control group
0.868421052631579
333	I29	I1	Control group	Control group
0.7244469870327994
334	I29	I3	Control group	Control group
0.7744088482074752
335	I29	I6	Control group	Control group
0.8470633104500381
336	I29	I8	Control group	Control group
0.7812738367658276
337	I29	I10	Control group	Control group
0.793859649122807
338	I29	I11	Control group	Control group
0.8731884057971014
339	I29	I13	Control group	Control group
0.8154080854309688
340	I29	I15	Control group	Control group
0.9128527841342486
341	I29	I17	Control group	Control group
0.7858504958047292
342	I29	I18	Control group	Control group
0.797673531655225
343	I29	I19	Control group	Control group
0.8985507246376812
344	I29	I22	Control group	Control group
0.7782227307398932
345	I29	I23	Control group	Control group
0.7885202135774219
346	I29	I24	Control group	Control group
0.7801296720061022
347	I29	I25	Control group	Control group
0.734744469870328
348	I29	I26	Control group	Control group
0.8035850495804729
349	I29	I27	Control group	Control group
0.8028222730739893
350	I29	I28	Control group	Control group

0.8432494279176201
351 I30 B27 Control group Control group
0.8647978642257819
352 I30 B66 Control group Control group
0.7263539282990084
353 I30 B86 Control group Control group
0.7980549199084668
354 I30 B97 Control group Control group
0.7536231884057971
355 I30 B98 Control group Control group
0.8260869565217391
356 I30 B100 Control group Control group
0.9429824561403509
357 I30 B112 Control group Control group
0.8867276887871853
358 I30 B115 Control group Control group
0.7639206712433257
359 I30 I1 Control group Control group
0.8115942028985508
360 I30 I3 Control group Control group
0.8178871090770404
361 I30 I6 Control group Control group
0.709954233409611
362 I30 I8 Control group Control group
0.8874904652936689
363 I30 I10 Control group Control group
0.8249427917620137
364 I30 I11 Control group Control group
0.8903508771929824
365 I30 I13 Control group Control group
0.8611746758199847
366 I30 I15 Control group Control group
0.6180396643783371
367 I30 I17 Control group Control group
0.9204805491990846
368 I30 I18 Control group Control group
0.7400839054157132
369 I30 I19 Control group Control group
0.9260106788710908
370 I30 I22 Control group Control group
0.8251334858886347
371 I30 I23 Control group Control group
0.7921434019832189
372 I30 I24 Control group Control group
0.8836765827612509
373 I30 I25 Control group Control group
0.8026315789473685
374 I30 I26 Control group Control group
0.7536231884057971
375 I30 I27 Control group Control group
0.6435926773455377
376 I30 I28 Control group Control group
0.7421815408085431
377 I30 I29 Control group Control group

0.8318077803203662
378	I31	B27	Control group	Control group
0.8604118993135011
379	I31	B66	Control group	Control group
0.8874904652936689
380	I31	B86	Control group	Control group
0.830282227307399
381	I31	B97	Control group	Control group
0.78813882532418
382	I31	B98	Control group	Control group
0.8142639206712433
383	I31	B100	Control group	Control group
0.9582379862700229
384	I31	B112	Control group	Control group
0.9090389016018307
385	I31	B115	Control group	Control group
0.8072082379862701
386	I31	I1	Control group	Control group
0.8127383676582761
387	I31	I3	Control group	Control group
0.7814645308924485
388	I31	I6	Control group	Control group
0.8032036613272311
389	I31	I8	Control group	Control group
0.843440122044241
390	I31	I10	Control group	Control group
0.7471395881006865
391	I31	I11	Control group	Control group
0.8552631578947368
392	I31	I13	Control group	Control group
0.8432494279176201
393	I31	I15	Control group	Control group
0.9376430205949656
394	I31	I17	Control group	Control group
0.8367658276125095
395	I31	I18	Control group	Control group
0.7677345537757437
396	I31	I19	Control group	Control group
0.9059877955758963
397	I31	I22	Control group	Control group
0.767162471395881
398	I31	I23	Control group	Control group
0.7254004576659039
399	I31	I24	Control group	Control group
0.8440122044241037
400	I31	I25	Control group	Control group
0.8125476735316552
401	I31	I26	Control group	Control group
0.8672768878718535
402	I31	I27	Control group	Control group
0.7566742944317315
403	I31	I28	Control group	Control group
0.8270404271548436
404	I31	I29	Control group	Control group

0.8463005339435545
405 I31 I30 Control group Control group
0.8363844393592678
406 I32 B27 Control group Control group
0.919908466819222
407 I32 B66 Control group Control group
0.8283752860411899
408 I32 B86 Control group Control group
0.843440122044241
409 I32 B97 Control group Control group
0.8197940503432495
410 I32 B98 Control group Control group
0.8485888634630053
411 I32 B100 Control group Control group
0.9876048817696415
412 I32 B112 Control group Control group
0.8888253241800153
413 I32 B115 Control group Control group
0.8176964149504196
414 I32 I1 Control group Control group
0.8457284515636918
415 I32 I3 Control group Control group
0.7753623188405797
416 I32 I6 Control group Control group
0.8781464530892449
417 I32 I8 Control group Control group
0.7126239511823036
418 I32 I10 Control group Control group
0.6439740655987796
419 I32 I11 Control group Control group
0.9265827612509535
420 I32 I13 Control group Control group
0.7974828375286042
421 I32 I15 Control group Control group
0.8787185354691075
422 I32 I17 Control group Control group
0.799771167048055
423 I32 I18 Control group Control group
0.727116704805492
424 I32 I19 Control group Control group
0.9364988558352403
425 I32 I22 Control group Control group
0.8405797101449275
426 I32 I23 Control group Control group
0.8655606407322655
427 I32 I24 Control group Control group
0.8447749809305873
428 I32 I25 Control group Control group
0.7065217391304348
429 I32 I26 Control group Control group
0.8163615560640732
430 I32 I27 Control group Control group
0.8188405797101449
431 I32 I28 Control group Control group

0.7991990846681922
432	I32	I29	Control group	Control group
0.7715484363081617
433	I32	I30	Control group	Control group
0.8565980167810832
434	I32	I31	Control group	Control group
0.7711670480549199
435	I33	B27	Control group	Control group
0.6718154080854309
436	I33	B66	Control group	Control group
0.841723874904653
437	I33	B86	Control group	Control group
0.4940884820747521
438	I33	B97	Control group	Control group
0.7732646834477498
439	I33	B98	Control group	Control group
0.7961479786422578
440	I33	B100	Control group	Control group
0.9668192219679634
441	I33	B112	Control group	Control group
0.6819221967963387
442	I33	B115	Control group	Control group
0.8007246376811594
443	I33	I1	Control group	Control group
0.7793668954996186
444	I33	I3	Control group	Control group
0.8081617086193745
445	I33	I6	Control group	Control group
0.7639206712433257
446	I33	I8	Control group	Control group
0.8079710144927537
447	I33	I10	Control group	Control group
0.7866132723112128
448	I33	I11	Control group	Control group
0.8867276887871853
449	I33	I13	Control group	Control group
0.7530511060259344
450	I33	I15	Control group	Control group
0.8852021357742181
451	I33	I17	Control group	Control group
0.6416857360793288
452	I33	I18	Control group	Control group
0.759534706331045
453	I33	I19	Control group	Control group
0.6638062547673532
454	I33	I22	Control group	Control group
0.8260869565217391
455	I33	I23	Control group	Control group
0.7885202135774219
456	I33	I24	Control group	Control group
0.4151411136536995
457	I33	I25	Control group	Control group
0.7204424103737604
458	I33	I26	Control group	Control group

0.8241800152555301
459	I33	I27	Control group	Control group
0.7513348588863463
460	I33	I28	Control group	Control group
0.8974065598779558
461	I33	I29	Control group	Control group
0.8571700991609459
462	I33	I30	Control group	Control group
0.8697559115179252
463	I33	I31	Control group	Control group
0.8699466056445462
464	I33	I32	Control group	Control group
0.8602212051868803
465	I34	B27	Control group	Control group
0.6727688787185355
466	I34	B66	Control group	Control group
0.7324561403508771
467	I34	B86	Control group	Control group
0.7627765064836003
468	I34	B97	Control group	Control group
0.8159801678108314
469	I34	B98	Control group	Control group
0.7967200610221206
470	I34	B100	Control group	Control group
0.9075133485888635
471	I34	B112	Control group	Control group
0.7999618611746758
472	I34	B115	Control group	Control group
0.799580472921434
473	I34	I1	Control group	Control group
0.7591533180778032
474	I34	I3	Control group	Control group
0.8676582761250954
475	I34	I6	Control group	Control group
0.7738367658276125
476	I34	I8	Control group	Control group
0.8771929824561403
477	I34	I10	Control group	Control group
0.8455377574370709
478	I34	I11	Control group	Control group
0.873951182303585
479	I34	I13	Control group	Control group
0.8190312738367659
480	I34	I15	Control group	Control group
0.8653699466056446
481	I34	I17	Control group	Control group
0.8041571319603357
482	I34	I18	Control group	Control group
0.7969107551487414
483	I34	I19	Control group	Control group
0.8184591914569032
484	I34	I22	Control group	Control group
0.8676582761250954
485	I34	I23	Control group	Control group

0.7513348588863463
486 I34 I24 Control group Control group
0.7286422578184591
487 I34 I25 Control group Control group
0.8297101449275363
488 I34 I26 Control group Control group
0.7726926010678871
489 I34 I27 Control group Control group
0.7623951182303585
490 I34 I28 Control group Control group
0.8979786422578184
491 I34 I29 Control group Control group
0.9017925247902364
492 I34 I30 Control group Control group
0.7991990846681922
493 I34 I31 Control group Control group
0.8930205949656751
494 I34 I32 Control group Control group
0.9122807017543859
495 I34 I33 Control group Control group
0.6872616323417239
496 I35 B27 Control group Control group
0.8451563691838292
497 I35 B66 Control group Control group
0.7536231884057971
498 I35 B86 Control group Control group
0.7713577421815409
499 I35 B97 Control group Control group 0.75
500 I35 B98 Control group Control group
0.6756292906178489
501 I35 B100 Control group Control group
0.9298245614035088
502 I35 B112 Control group Control group
0.8729977116704806
503 I35 B115 Control group Control group
0.8581235697940504
504 I35 I1 Control group Control group
0.71186117467582
505 I35 I3 Control group Control group
0.6655225019069413
506 I35 I6 Control group Control group
0.7778413424866514
507 I35 I8 Control group Control group
0.78813882532418
508 I35 I10 Control group Control group
0.7486651411136537
509 I35 I11 Control group Control group
0.8222730739893211
510 I35 I13 Control group Control group
0.7170099160945843
511 I35 I15 Control group Control group
0.935163996948894
512 I35 I17 Control group Control group
0.7677345537757437

513	I35	I18	Control group	Control group	0.7313119755911518
514	I35	I19	Control group	Control group	0.9031273836765827
515	I35	I22	Control group	Control group	0.7349351639969489
516	I35	I23	Control group	Control group	0.6401601830663616
517	I35	I24	Control group	Control group	0.8497330282227308
518	I35	I25	Control group	Control group	0.6920289855072463
519	I35	I26	Control group	Control group	0.8024408848207475
520	I35	I27	Control group	Control group	0.7904271548436308
521	I35	I28	Control group	Control group	0.704233409610984
522	I35	I29	Control group	Control group	0.7961479786422578
523	I35	I30	Control group	Control group	0.897025171624714
524	I35	I31	Control group	Control group	0.8325705568268498
525	I35	I32	Control group	Control group	0.7890922959572845
526	I35	I33	Control group	Control group	0.797673531655225
527	I35	I34	Control group	Control group	0.8476353928299009
528	I36	B27	Control group	Control group	0.9940884820747521
529	I36	B66	Control group	Control group	0.9879862700228833
530	I36	B86	Control group	Control group	0.9910373760488177
531	I36	B97	Control group	Control group	0.9940884820747521
532	I36	B98	Control group	Control group	0.9908466819221968
533	I36	B100	Control group	Control group	0.885392829900839
534	I36	B112	Control group	Control group	0.9988558352402745
535	I36	B115	Control group	Control group	0.9895118230358505
536	I36	I1	Control group	Control group	0.9979023646071701
537	I36	I3	Control group	Control group	0.9969488939740656
538	I36	I6	Control group	Control group	0.998093058733791
539	I36	I8	Control group	Control group	0.9929443173150267

540	I36	I10	Control group	Control group	0.9935163996948894
541	I36	I11	Control group	Control group	0.967581998474447
542	I36	I13	Control group	Control group	0.9876048817696415
543	I36	I15	Control group	Control group	0.868421052631579
544	I36	I17	Control group	Control group	0.998093058733791
545	I36	I18	Control group	Control group	0.929252479023646
546	I36	I19	Control group	Control group	0.9944698703279939
547	I36	I22	Control group	Control group	0.8741418764302059
548	I36	I23	Control group	Control group	0.9908466819221968
549	I36	I24	Control group	Control group	0.9990465293668955
550	I36	I25	Control group	Control group	0.9982837528604119
551	I36	I26	Control group	Control group	0.9919908466819222
552	I36	I27	Control group	Control group	0.9826468344774981
553	I36	I28	Control group	Control group	0.9323035850495804
554	I36	I29	Control group	Control group	0.9933257055682685
555	I36	I30	Control group	Control group	0.9765446224256293
556	I36	I31	Control group	Control group	0.9929443173150267
557	I36	I32	Control group	Control group	0.9952326468344775
558	I36	I33	Control group	Control group	0.9979023646071701
559	I36	I34	Control group	Control group	0.9933257055682685
560	I36	I35	Control group	Control group	0.9927536231884058
561	I37	B27	Control group	Control group	0.8405797101449275
562	I37	B66	Control group	Control group	0.8785278413424866
563	I37	B86	Control group	Control group	0.912090007627765
564	I37	B97	Control group	Control group	0.8951182303585049
565	I37	B98	Control group	Control group	0.8155987795575896
566	I37	B100	Control group	Control group	0.9265827612509535

567	I37	B112	Control group	Control group
0.9401220442410374
568	I37	B115	Control group	Control group
0.8773836765827613
569	I37	I1	Control group	Control group
0.8011060259344012
570	I37	I3	Control group	Control group
0.8943554538520213
571	I37	I6	Control group	Control group
0.8667048054919908
572	I37	I8	Control group	Control group
0.9057971014492754
573	I37	I10	Control group	Control group
0.8974065598779558
574	I37	I11	Control group	Control group
0.8419145690312738
575	I37	I13	Control group	Control group
0.8594584286803967
576	I37	I15	Control group	Control group
0.8470633104500381
577	I37	I17	Control group	Control group
0.9233409610983981
578	I37	I18	Control group	Control group
0.8592677345537757
579	I37	I19	Control group	Control group
0.9557589626239512
580	I37	I22	Control group	Control group
0.7940503432494279
581	I37	I23	Control group	Control group
0.8087337909992373
582	I37	I24	Control group	Control group
0.956140350877193
583	I37	I25	Control group	Control group
0.9138062547673532
584	I37	I26	Control group	Control group
0.9000762776506483
585	I37	I27	Control group	Control group
0.9231502669717773
586	I37	I28	Control group	Control group
0.8447749809305873
587	I37	I29	Control group	Control group
0.9347826086956522
588	I37	I30	Control group	Control group
0.9590007627765065
589	I37	I31	Control group	Control group
0.9256292906178489
590	I37	I32	Control group	Control group
0.9466056445461479
591	I37	I33	Control group	Control group
0.9359267734553776
592	I37	I34	Control group	Control group
0.9218154080854309
593	I37	I35	Control group	Control group
0.7648741418764302

594	I37	I36	Control group	Control group
0.7353165522501907
595	B27	B1	Control group	Idiopathic BAD
0.8424866514111365
596	B27	B5	Control group	Idiopathic BAD
0.8808161708619374
597	B27	B6	Control group	Idiopathic BAD
0.8735697940503433
598	B27	B10	Control group	Idiopathic BAD
0.7650648360030511
599	B27	B17	Control group	Idiopathic BAD
0.6607551487414187
600	B27	B20	Control group	Idiopathic BAD
0.5835240274599542
601	B27	B23	Control group	Idiopathic BAD
0.7608695652173914
602	B27	B31	Control group	Idiopathic BAD
0.7246376811594203
603	B27	B35	Control group	Idiopathic BAD
0.9692982456140351
604	B27	B39	Control group	Idiopathic BAD
0.8522120518688024
605	B27	B43	Control group	Idiopathic BAD
0.715675057208238
606	B27	B47	Control group	Idiopathic BAD
0.7854691075514875
607	B27	B48	Control group	Idiopathic BAD
0.8731884057971014
608	B27	B49	Control group	Idiopathic BAD
0.7890922959572845
609	B27	B53	Control group	Idiopathic BAD
0.8205568268497331
610	B27	B54	Control group	Idiopathic BAD
0.8106407322654462
611	B27	B55	Control group	Idiopathic BAD
0.8032036613272311
612	B27	B59	Control group	Idiopathic BAD
0.9754004576659039
613	B27	B70	Control group	Idiopathic BAD
0.9235316552250191
614	B27	B74	Control group	Idiopathic BAD
0.7951945080091534
615	B27	B77	Control group	Idiopathic BAD
0.7753623188405797
616	B27	B81	Control group	Idiopathic BAD
0.8104500381388253
617	B27	B84	Control group	Idiopathic BAD
0.8916857360793288
618	B27	B89	Control group	Idiopathic BAD
0.9088482074752098
619	B27	B92	Control group	Idiopathic BAD
0.6228070175438597
620	B27	B95	Control group	Idiopathic BAD
0.6407322654462243

621	B27	B99	Control group	Idiopathic BAD
0.8247520976353928
622	B27	B103	Control group	Idiopathic BAD
0.9214340198321892
623	B27	B106	Control group	Idiopathic BAD
0.8609839816933639
624	B27	B109	Control group	Idiopathic BAD
0.8806254767353165
625	B27	B118	Control group	Idiopathic BAD
0.7988176964149504
626	B27	B119	Control group	Idiopathic BAD
0.8497330282227308
627	B66	B1	Control group	Idiopathic BAD
0.8979786422578184
628	B66	B5	Control group	Idiopathic BAD
0.8220823798627003
629	B66	B6	Control group	Idiopathic BAD
0.8785278413424866
630	B66	B10	Control group	Idiopathic BAD
0.8672768878718535
631	B66	B17	Control group	Idiopathic BAD
0.7873760488176964
632	B66	B20	Control group	Idiopathic BAD
0.8295194508009154
633	B66	B23	Control group	Idiopathic BAD
0.6479786422578184
634	B66	B31	Control group	Idiopathic BAD
0.7890922959572845
635	B66	B35	Control group	Idiopathic BAD
0.9262013729977117
636	B66	B39	Control group	Idiopathic BAD
0.8678489702517163
637	B66	B43	Control group	Idiopathic BAD
0.9126620900076278
638	B66	B47	Control group	Idiopathic BAD
0.7936689549961862
639	B66	B48	Control group	Idiopathic BAD
0.8703279938977879
640	B66	B49	Control group	Idiopathic BAD
0.791952707856598
641	B66	B53	Control group	Idiopathic BAD
0.8136918382913806
642	B66	B54	Control group	Idiopathic BAD
0.8686117467581999
643	B66	B55	Control group	Idiopathic BAD
0.8503051106025934
644	B66	B59	Control group	Idiopathic BAD
0.9710144927536232
645	B66	B70	Control group	Idiopathic BAD
0.7711670480549199
646	B66	B74	Control group	Idiopathic BAD
0.7814645308924485
647	B66	B77	Control group	Idiopathic BAD
0.8089244851258581

648	B66	B81	Control group	Idiopathic BAD	0.7610602593440122
649	B66	B84	Control group	Idiopathic BAD	0.8077803203661327
650	B66	B89	Control group	Idiopathic BAD	0.812929061784897
651	B66	B92	Control group	Idiopathic BAD	0.7799389778794813
652	B66	B95	Control group	Idiopathic BAD	0.8890160183066361
653	B66	B99	Control group	Idiopathic BAD	0.6893592677345538
654	B66	B103	Control group	Idiopathic BAD	0.8569794050343249
655	B66	B106	Control group	Idiopathic BAD	0.9443173150266971
656	B66	B109	Control group	Idiopathic BAD	0.7479023646071701
657	B66	B118	Control group	Idiopathic BAD	0.8522120518688024
658	B66	B119	Control group	Idiopathic BAD	0.6960335621662853
659	B86	B1	Control group	Idiopathic BAD	0.9178108314263921
660	B86	B5	Control group	Idiopathic BAD	0.8203661327231121
661	B86	B6	Control group	Idiopathic BAD	0.8659420289855072
662	B86	B10	Control group	Idiopathic BAD	0.8956903127383676
663	B86	B17	Control group	Idiopathic BAD	0.6203279938977879
664	B86	B20	Control group	Idiopathic BAD	0.6651411136536994
665	B86	B23	Control group	Idiopathic BAD	0.8115942028985508
666	B86	B31	Control group	Idiopathic BAD	0.8689931350114416
667	B86	B35	Control group	Idiopathic BAD	0.9815026697177727
668	B86	B39	Control group	Idiopathic BAD	0.9014111365369947
669	B86	B43	Control group	Idiopathic BAD	0.7034706331045004
670	B86	B47	Control group	Idiopathic BAD	0.8056826849733029
671	B86	B48	Control group	Idiopathic BAD	0.9458428680396643
672	B86	B49	Control group	Idiopathic BAD	0.8270404271548436
673	B86	B53	Control group	Idiopathic BAD	0.8051106025934401
674	B86	B54	Control group	Idiopathic BAD	0.5983981693363845

675	B86	B55	Control group	Idiopathic BAD	0.6266209000762777
676	B86	B59	Control group	Idiopathic BAD	0.919717772692601
677	B86	B70	Control group	Idiopathic BAD	0.8844393592677345
678	B86	B74	Control group	Idiopathic BAD	0.8516399694889397
679	B86	B77	Control group	Idiopathic BAD	0.8051106025934401
680	B86	B81	Control group	Idiopathic BAD	0.8068268497330282
681	B86	B84	Control group	Idiopathic BAD	0.8752860411899314
682	B86	B89	Control group	Idiopathic BAD	0.809115179252479
683	B86	B92	Control group	Idiopathic BAD	0.580091533180778
684	B86	B95	Control group	Idiopathic BAD	0.7524790236460717
685	B86	B99	Control group	Idiopathic BAD	0.6990846681922197
686	B86	B103	Control group	Idiopathic BAD	0.9004576659038902
687	B86	B106	Control group	Idiopathic BAD	0.6784897025171625
688	B86	B109	Control group	Idiopathic BAD	0.8203661327231121
689	B86	B118	Control group	Idiopathic BAD	0.5678871090770404
690	B86	B119	Control group	Idiopathic BAD	0.7915713196033562
691	B97	B1	Control group	Idiopathic BAD	0.8520213577421816
692	B97	B5	Control group	Idiopathic BAD	0.8077803203661327
693	B97	B6	Control group	Idiopathic BAD	0.8598398169336384
694	B97	B10	Control group	Idiopathic BAD	0.8257055682684973
695	B97	B17	Control group	Idiopathic BAD	0.7522883295194508
696	B97	B20	Control group	Idiopathic BAD	0.8190312738367659
697	B97	B23	Control group	Idiopathic BAD	0.7028985507246377
698	B97	B31	Control group	Idiopathic BAD	0.7795575896262396
699	B97	B35	Control group	Idiopathic BAD	0.9309687261632341
700	B97	B39	Control group	Idiopathic BAD	0.8117848970251716
701	B97	B43	Control group	Idiopathic BAD	0.8384820747520977

702	B97	B47	Control group	Idiopathic BAD	0.7755530129672006
703	B97	B48	Control group	Idiopathic BAD	0.8872997711670481
704	B97	B49	Control group	Idiopathic BAD	0.6359649122807017
705	B97	B53	Control group	Idiopathic BAD	0.8194126620900076
706	B97	B54	Control group	Idiopathic BAD	0.8901601830663616
707	B97	B55	Control group	Idiopathic BAD	0.8508771929824561
708	B97	B59	Control group	Idiopathic BAD	0.9681540808543097
709	B97	B70	Control group	Idiopathic BAD	0.8157894736842105
710	B97	B74	Control group	Idiopathic BAD	0.6742944317315027
711	B97	B77	Control group	Idiopathic BAD	0.7677345537757437
712	B97	B81	Control group	Idiopathic BAD	0.7154843630816171
713	B97	B84	Control group	Idiopathic BAD	0.8419145690312738
714	B97	B89	Control group	Idiopathic BAD	0.8007246376811594
715	B97	B92	Control group	Idiopathic BAD	0.7376048817696415
716	B97	B95	Control group	Idiopathic BAD	0.8119755911517925
717	B97	B99	Control group	Idiopathic BAD	0.6235697940503433
718	B97	B103	Control group	Idiopathic BAD	0.9124713958810069
719	B97	B106	Control group	Idiopathic BAD	0.9298245614035088
720	B97	B109	Control group	Idiopathic BAD	0.698512585812357
721	B97	B118	Control group	Idiopathic BAD	0.8787185354691075
722	B97	B119	Control group	Idiopathic BAD	0.7446605644546148
723	B98	B1	Control group	Idiopathic BAD	0.7566742944317315
724	B98	B5	Control group	Idiopathic BAD	0.801487414187643
725	B98	B6	Control group	Idiopathic BAD	0.8699466056445462
726	B98	B10	Control group	Idiopathic BAD	0.7362700228832952
727	B98	B17	Control group	Idiopathic BAD	0.6250953470633105
728	B98	B20	Control group	Idiopathic BAD	0.7921434019832189

729	B98	B23	Control group	Idiopathic BAD
0.7215865751334859
730	B98	B31	Control group	Idiopathic BAD
0.6340579710144928
731	B98	B35	Control group	Idiopathic BAD
0.9319221967963387
732	B98	B39	Control group	Idiopathic BAD
0.7599160945842868
733	B98	B43	Control group	Idiopathic BAD
0.8668954996186118
734	B98	B47	Control group	Idiopathic BAD
0.7425629290617849
735	B98	B48	Control group	Idiopathic BAD
0.8165522501906941
736	B98	B49	Control group	Idiopathic BAD
0.7526697177726926
737	B98	B53	Control group	Idiopathic BAD
0.8251334858886347
738	B98	B54	Control group	Idiopathic BAD
0.8522120518688024
739	B98	B55	Control group	Idiopathic BAD
0.9105644546147978
740	B98	B59	Control group	Idiopathic BAD
0.9767353165522502
741	B98	B70	Control group	Idiopathic BAD
0.8972158657513348
742	B98	B74	Control group	Idiopathic BAD
0.759534706331045
743	B98	B77	Control group	Idiopathic BAD
0.8100686498855835
744	B98	B81	Control group	Idiopathic BAD
0.8381006864988558
745	B98	B84	Control group	Idiopathic BAD
0.8678489702517163
746	B98	B89	Control group	Idiopathic BAD
0.7503813882532419
747	B98	B92	Control group	Idiopathic BAD
0.7696414950419527
748	B98	B95	Control group	Idiopathic BAD
0.7459954233409611
749	B98	B99	Control group	Idiopathic BAD
0.7139588100686499
750	B98	B103	Control group	Idiopathic BAD
0.868421052631579
751	B98	B106	Control group	Idiopathic BAD
0.9315408085430968
752	B98	B109	Control group	Idiopathic BAD
0.8619374523264683
753	B98	B118	Control group	Idiopathic BAD
0.8443935926773455
754	B98	B119	Control group	Idiopathic BAD
0.8146453089244852
755	B100	B1	Control group	Idiopathic BAD
0.9435545385202135

756	B100	B5	Control group	Idiopathic BAD	0.9481311975591151
757	B100	B6	Control group	Idiopathic BAD	0.9639588100686499
758	B100	B10	Control group	Idiopathic BAD	0.8672768878718535
759	B100	B17	Control group	Idiopathic BAD	0.9231502669717773
760	B100	B20	Control group	Idiopathic BAD	0.9664378337147216
761	B100	B23	Control group	Idiopathic BAD	0.8630816170861938
762	B100	B31	Control group	Idiopathic BAD	0.9010297482837528
763	B100	B35	Control group	Idiopathic BAD	0.881578947368421
764	B100	B39	Control group	Idiopathic BAD	0.9439359267734554
765	B100	B43	Control group	Idiopathic BAD	0.9706331045003814
766	B100	B47	Control group	Idiopathic BAD	0.9515636918382914
767	B100	B48	Control group	Idiopathic BAD	0.8842486651411137
768	B100	B49	Control group	Idiopathic BAD	0.940884820747521
769	B100	B53	Control group	Idiopathic BAD	0.9126620900076278
770	B100	B54	Control group	Idiopathic BAD	0.9374523264683448
771	B100	B55	Control group	Idiopathic BAD	0.9845537757437071
772	B100	B59	Control group	Idiopathic BAD	0.9155225019069413
773	B100	B70	Control group	Idiopathic BAD	0.9090389016018307
774	B100	B74	Control group	Idiopathic BAD	0.9233409610983981
775	B100	B77	Control group	Idiopathic BAD	0.9052250190694127
776	B100	B81	Control group	Idiopathic BAD	0.9393592677345538
777	B100	B84	Control group	Idiopathic BAD	0.9294431731502669
778	B100	B89	Control group	Idiopathic BAD	0.9801678108314263
779	B100	B92	Control group	Idiopathic BAD	0.9195270785659801
780	B100	B95	Control group	Idiopathic BAD	0.9610983981693364
781	B100	B99	Control group	Idiopathic BAD	0.9624332570556827
782	B100	B103	Control group	Idiopathic BAD	0.9734935163996948

783	B100	B106	Control group	Idiopathic BAD	0.9855072463768116
784	B100	B109	Control group	Idiopathic BAD	0.9822654462242563
785	B100	B118	Control group	Idiopathic BAD	0.9530892448512586
786	B100	B119	Control group	Idiopathic BAD	0.9336384439359268
787	B112	B1	Control group	Idiopathic BAD	0.9311594202898551
788	B112	B5	Control group	Idiopathic BAD	0.8779557589626239
789	B112	B6	Control group	Idiopathic BAD	0.9153318077803204
790	B112	B10	Control group	Idiopathic BAD	0.9052250190694127
791	B112	B17	Control group	Idiopathic BAD	0.5106788710907704
792	B112	B20	Control group	Idiopathic BAD	0.631769641495042
793	B112	B23	Control group	Idiopathic BAD	0.8230358504958047
794	B112	B31	Control group	Idiopathic BAD	0.860602593440122
795	B112	B35	Control group	Idiopathic BAD	0.967581998474447
796	B112	B39	Control group	Idiopathic BAD	0.8918764302059496
797	B112	B43	Control group	Idiopathic BAD	0.7837528604118993
798	B112	B47	Control group	Idiopathic BAD	0.833905415713196
799	B112	B48	Control group	Idiopathic BAD	0.9336384439359268
800	B112	B49	Control group	Idiopathic BAD	0.8867276887871853
801	B112	B53	Control group	Idiopathic BAD	0.8699466056445462
802	B112	B54	Control group	Idiopathic BAD	0.6058352402745996
803	B112	B55	Control group	Idiopathic BAD	0.6624713958810069
804	B112	B59	Control group	Idiopathic BAD	0.9809305873379099
805	B112	B70	Control group	Idiopathic BAD	0.9138062547673532
806	B112	B74	Control group	Idiopathic BAD	0.8234172387490465
807	B112	B77	Control group	Idiopathic BAD	0.8524027459954233
808	B112	B81	Control group	Idiopathic BAD	0.8771929824561403
809	B112	B84	Control group	Idiopathic BAD	0.9042715484363082

810 B112 B89 Control group Idiopathic BAD
0.8859649122807017
811 B112 B92 Control group Idiopathic BAD
0.6859267734553776
812 B112 B95 Control group Idiopathic BAD
0.8070175438596491
813 B112 B99 Control group Idiopathic BAD
0.8266590389016019
814 B112 B103 Control group Idiopathic BAD
0.9361174675819984
815 B112 B106 Control group Idiopathic BAD
0.511441647597254
816 B112 B109 Control group Idiopathic BAD
0.8869183829138062
817 B112 B118 Control group Idiopathic BAD
0.6413043478260869
818 B112 B119 Control group Idiopathic BAD
0.8625095347063311
819 B115 B1 Control group Idiopathic BAD
0.8096872616323417
820 B115 B5 Control group Idiopathic BAD
0.8632723112128147
821 B115 B6 Control group Idiopathic BAD
0.8432494279176201
822 B115 B10 Control group Idiopathic BAD
0.732837528604119
823 B115 B17 Control group Idiopathic BAD
0.7192982456140351
824 B115 B20 Control group Idiopathic BAD
0.8356216628527842
825 B115 B23 Control group Idiopathic BAD
0.7644927536231884
826 B115 B31 Control group Idiopathic BAD
0.6916475972540046
827 B115 B35 Control group Idiopathic BAD
0.9803585049580473
828 B115 B39 Control group Idiopathic BAD
0.8615560640732265
829 B115 B43 Control group Idiopathic BAD
0.8663234172387491
830 B115 B47 Control group Idiopathic BAD
0.736651411136537
831 B115 B48 Control group Idiopathic BAD
0.7797482837528604
832 B115 B49 Control group Idiopathic BAD
0.778604118993135
833 B115 B53 Control group Idiopathic BAD
0.8518306636155606
834 B115 B54 Control group Idiopathic BAD
0.8991228070175439
835 B115 B55 Control group Idiopathic BAD
0.8218916857360793
836 B115 B59 Control group Idiopathic BAD
0.9839816933638444

837	B115	B70	Control group	Idiopathic BAD	0.881769641495042
838	B115	B74	Control group	Idiopathic BAD	0.7625858123569794
839	B115	B77	Control group	Idiopathic BAD	0.7219679633867276
840	B115	B81	Control group	Idiopathic BAD	0.8321891685736079
841	B115	B84	Control group	Idiopathic BAD	0.8802440884820748
842	B115	B89	Control group	Idiopathic BAD	0.868421052631579
843	B115	B92	Control group	Idiopathic BAD	0.8165522501906941
844	B115	B95	Control group	Idiopathic BAD	0.8874904652936689
845	B115	B99	Control group	Idiopathic BAD	0.7745995423340961
846	B115	B103	Control group	Idiopathic BAD	0.9349733028222731
847	B115	B106	Control group	Idiopathic BAD	0.933066361556064
848	B115	B109	Control group	Idiopathic BAD	0.784324942791762
849	B115	B118	Control group	Idiopathic BAD	0.9084668192219679
850	B115	B119	Control group	Idiopathic BAD	0.723302822273074
851	I1	B1	Control group	Idiopathic BAD	0.7942410373760488
852	I1	B5	Control group	Idiopathic BAD	0.8392448512585813
853	I1	B6	Control group	Idiopathic BAD	0.7965293668954996
854	I1	B10	Control group	Idiopathic BAD	0.6594202898550725
855	I1	B17	Control group	Idiopathic BAD	0.6712433257055682
856	I1	B20	Control group	Idiopathic BAD	0.7568649885583524
857	I1	B23	Control group	Idiopathic BAD	0.7185354691075515
858	I1	B31	Control group	Idiopathic BAD	0.7303585049580473
859	I1	B35	Control group	Idiopathic BAD	0.9801678108314263
860	I1	B39	Control group	Idiopathic BAD	0.8667048054919908
861	I1	B43	Control group	Idiopathic BAD	0.8020594965675057
862	I1	B47	Control group	Idiopathic BAD	0.6842105263157895
863	I1	B48	Control group	Idiopathic BAD	0.8543096872616324

864	I1	B49	Control group	Idiopathic BAD	0.8003432494279176
865	I1	B53	Control group	Idiopathic BAD	0.8220823798627003
866	I1	B54	Control group	Idiopathic BAD	0.8360030511060259
867	I1	B55	Control group	Idiopathic BAD	0.8844393592677345
868	I1	B59	Control group	Idiopathic BAD	0.9799771167048055
869	I1	B70	Control group	Idiopathic BAD	0.8695652173913043
870	I1	B74	Control group	Idiopathic BAD	0.7206331045003814
871	I1	B77	Control group	Idiopathic BAD	0.8028222730739893
872	I1	B81	Control group	Idiopathic BAD	0.776697177726926
873	I1	B84	Control group	Idiopathic BAD	0.8779557589626239
874	I1	B89	Control group	Idiopathic BAD	0.8197940503432495
875	I1	B92	Control group	Idiopathic BAD	0.8062547673531655
876	I1	B95	Control group	Idiopathic BAD	0.8165522501906941
877	I1	B99	Control group	Idiopathic BAD	0.6548436308161708
878	I1	B103	Control group	Idiopathic BAD	0.9286803966437833
879	I1	B106	Control group	Idiopathic BAD	0.9321128909229596
880	I1	B109	Control group	Idiopathic BAD	0.8237986270022883
881	I1	B118	Control group	Idiopathic BAD	0.8102593440122045
882	I1	B119	Control group	Idiopathic BAD	0.7658276125095347
883	I3	B1	Control group	Idiopathic BAD	0.8400076277650649
884	I3	B5	Control group	Idiopathic BAD	0.7282608695652174
885	I3	B6	Control group	Idiopathic BAD	0.813119755911518
886	I3	B10	Control group	Idiopathic BAD	0.8552631578947368
887	I3	B17	Control group	Idiopathic BAD	0.7921434019832189
888	I3	B20	Control group	Idiopathic BAD	0.8426773455377574
889	I3	B23	Control group	Idiopathic BAD	0.7990083905415714
890	I3	B31	Control group	Idiopathic BAD	0.830091533180778

891	I3	B35	Control group	Idiopathic BAD
0.8321891685736079
892	I3	B39	Control group	Idiopathic BAD
0.8314263920671243
893	I3	B43	Control group	Idiopathic BAD
0.8144546147978642
894	I3	B47	Control group	Idiopathic BAD
0.8546910755148741
895	I3	B48	Control group	Idiopathic BAD
0.7494279176201373
896	I3	B49	Control group	Idiopathic BAD
0.6578947368421053
897	I3	B53	Control group	Idiopathic BAD
0.8699466056445462
898	I3	B54	Control group	Idiopathic BAD
0.9233409610983981
899	I3	B55	Control group	Idiopathic BAD
0.948512585812357
900	I3	B59	Control group	Idiopathic BAD
0.9929443173150267
901	I3	B70	Control group	Idiopathic BAD
0.7961479786422578
902	I3	B74	Control group	Idiopathic BAD
0.889397406559878
903	I3	B77	Control group	Idiopathic BAD
0.8617467581998475
904	I3	B81	Control group	Idiopathic BAD
0.7812738367658276
905	I3	B84	Control group	Idiopathic BAD
0.8806254767353165
906	I3	B89	Control group	Idiopathic BAD
0.7488558352402745
907	I3	B92	Control group	Idiopathic BAD
0.8367658276125095
908	I3	B95	Control group	Idiopathic BAD
0.7965293668954996
909	I3	B99	Control group	Idiopathic BAD
0.6401601830663616
910	I3	B103	Control group	Idiopathic BAD
0.8996948893974066
911	I3	B106	Control group	Idiopathic BAD
0.9210526315789473
912	I3	B109	Control group	Idiopathic BAD
0.7627765064836003
913	I3	B118	Control group	Idiopathic BAD
0.8382913806254767
914	I3	B119	Control group	Idiopathic BAD
0.7318840579710145
915	I6	B1	Control group	Idiopathic BAD
0.8892067124332571
916	I6	B5	Control group	Idiopathic BAD
0.822463768115942
917	I6	B6	Control group	Idiopathic BAD
0.8501144164759725

918	I6	B10	Control group	Idiopathic BAD	0.8163615560640732
919	I6	B17	Control group	Idiopathic BAD	0.7585812356979404
920	I6	B20	Control group	Idiopathic BAD	0.8001525553012967
921	I6	B23	Control group	Idiopathic BAD	0.6590389016018307
922	I6	B31	Control group	Idiopathic BAD	0.778794813119756
923	I6	B35	Control group	Idiopathic BAD	0.9750190694126621
924	I6	B39	Control group	Idiopathic BAD	0.7526697177726926
925	I6	B43	Control group	Idiopathic BAD	0.8941647597254004
926	I6	B47	Control group	Idiopathic BAD	0.6884057971014492
927	I6	B48	Control group	Idiopathic BAD	0.8342868039664378
928	I6	B49	Control group	Idiopathic BAD	0.7812738367658276
929	I6	B53	Control group	Idiopathic BAD	0.7446605644546148
930	I6	B54	Control group	Idiopathic BAD	0.816742944317315
931	I6	B55	Control group	Idiopathic BAD	0.8371472158657514
932	I6	B59	Control group	Idiopathic BAD	0.9563310450038138
933	I6	B70	Control group	Idiopathic BAD	0.7580091533180778
934	I6	B74	Control group	Idiopathic BAD	0.7690694126620901
935	I6	B77	Control group	Idiopathic BAD	0.6748665141113653
936	I6	B81	Control group	Idiopathic BAD	0.8037757437070938
937	I6	B84	Control group	Idiopathic BAD	0.8348588863463006
938	I6	B89	Control group	Idiopathic BAD	0.8422959572845157
939	I6	B92	Control group	Idiopathic BAD	0.7873760488176964
940	I6	B95	Control group	Idiopathic BAD	0.8175057208237986
941	I6	B99	Control group	Idiopathic BAD	0.6771548436308161
942	I6	B103	Control group	Idiopathic BAD	0.9424103737604882
943	I6	B106	Control group	Idiopathic BAD	0.9246758199847445
944	I6	B109	Control group	Idiopathic BAD	0.7723112128146453

945 I6 B118 Control group Idiopathic BAD
0.8564073226544623
946 I6 B119 Control group Idiopathic BAD
0.6374904652936689
947 I8 B1 Control group Idiopathic BAD
0.9046529366895499
948 I8 B5 Control group Idiopathic BAD
0.8688024408848207
949 I8 B6 Control group Idiopathic BAD
0.8323798627002288
950 I8 B10 Control group Idiopathic BAD
0.8346681922196796
951 I8 B17 Control group Idiopathic BAD
0.837909992372235
952 I8 B20 Control group Idiopathic BAD
0.8257055682684973
953 I8 B23 Control group Idiopathic BAD
0.8064454614797865
954 I8 B31 Control group Idiopathic BAD
0.8508771929824561
955 I8 B35 Control group Idiopathic BAD
0.9912280701754386
956 I8 B39 Control group Idiopathic BAD
0.8960717009916095
957 I8 B43 Control group Idiopathic BAD
0.782608695652174
958 I8 B47 Control group Idiopathic BAD
0.8421052631578947
959 I8 B48 Control group Idiopathic BAD
0.967391304347826
960 I8 B49 Control group Idiopathic BAD
0.8146453089244852
961 I8 B53 Control group Idiopathic BAD
0.8607932875667429
962 I8 B54 Control group Idiopathic BAD
0.9178108314263921
963 I8 B55 Control group Idiopathic BAD
0.9214340198321892
964 I8 B59 Control group Idiopathic BAD
0.9666285278413425
965 I8 B70 Control group Idiopathic BAD
0.9345919145690312
966 I8 B74 Control group Idiopathic BAD
0.8119755911517925
967 I8 B77 Control group Idiopathic BAD
0.8243707093821511
968 I8 B81 Control group Idiopathic BAD
0.8636536994660564
969 I8 B84 Control group Idiopathic BAD
0.8945461479786423
970 I8 B89 Control group Idiopathic BAD
0.8573607932875668
971 I8 B92 Control group Idiopathic BAD
0.8199847444698704

972	I8	B95	Control group	Idiopathic BAD
0.8829138062547673
973	I8	B99	Control group	Idiopathic BAD
0.7437070938215103
974	I8	B103	Control group	Idiopathic BAD
0.9420289855072463
975	I8	B106	Control group	Idiopathic BAD
0.9315408085430968
976	I8	B109	Control group	Idiopathic BAD
0.8766209000762777
977	I8	B118	Control group	Idiopathic BAD
0.8787185354691075
978	I8	B119	Control group	Idiopathic BAD
0.7984363081617086
979	I10	B1	Control group	Idiopathic BAD
0.9071319603356217
980	I10	B5	Control group	Idiopathic BAD
0.7585812356979404
981	I10	B6	Control group	Idiopathic BAD
0.7858504958047292
982	I10	B10	Control group	Idiopathic BAD
0.916094584286804
983	I10	B17	Control group	Idiopathic BAD
0.8546910755148741
984	I10	B20	Control group	Idiopathic BAD
0.7591533180778032
985	I10	B23	Control group	Idiopathic BAD
0.8047292143401983
986	I10	B31	Control group	Idiopathic BAD
0.8573607932875668
987	I10	B35	Control group	Idiopathic BAD
0.9492753623188406
988	I10	B39	Control group	Idiopathic BAD
0.8750953470633105
989	I10	B43	Control group	Idiopathic BAD
0.7585812356979404
990	I10	B47	Control group	Idiopathic BAD
0.8585049580472921
991	I10	B48	Control group	Idiopathic BAD
0.9059877955758963
992	I10	B49	Control group	Idiopathic BAD
0.7639206712433257
993	I10	B53	Control group	Idiopathic BAD
0.8825324180015256
994	I10	B54	Control group	Idiopathic BAD
0.898932112890923
995	I10	B55	Control group	Idiopathic BAD
0.9555682684973302
996	I10	B59	Control group	Idiopathic BAD
0.9816933638443935
997	I10	B70	Control group	Idiopathic BAD
0.8520213577421816
998	I10	B74	Control group	Idiopathic BAD
0.9012204424103738

999 I10 B77 Control group Idiopathic BAD
0.8585049580472921
1000 I10 B81 Control group Idiopathic BAD
0.8981693363844394
1001 I10 B84 Control group Idiopathic BAD
0.8476353928299009
1002 I10 B89 Control group Idiopathic BAD
0.7663996948893974
1003 I10 B92 Control group Idiopathic BAD
0.7868039664378337
1004 I10 B95 Control group Idiopathic BAD
0.8400076277650649
1005 I10 B99 Control group Idiopathic BAD
0.7076659038901602
1006 I10 B103 Control group Idiopathic BAD
0.8884439359267735
1007 I10 B106 Control group Idiopathic BAD
0.9477498093058734
1008 I10 B109 Control group Idiopathic BAD
0.7951945080091534
1009 I10 B118 Control group Idiopathic BAD
0.8712814645308925
1010 I10 B119 Control group Idiopathic BAD
0.7694508009153318
1011 I11 B1 Control group Idiopathic BAD
0.8278032036613272
1012 I11 B5 Control group Idiopathic BAD
0.8758581235697941
1013 I11 B6 Control group Idiopathic BAD
0.8394355453852022
1014 I11 B10 Control group Idiopathic BAD
0.8398169336384439
1015 I11 B17 Control group Idiopathic BAD
0.8813882532418001
1016 I11 B20 Control group Idiopathic BAD
0.9088482074752098
1017 I11 B23 Control group Idiopathic BAD
0.8808161708619374
1018 I11 B31 Control group Idiopathic BAD
0.8276125095347063
1019 I11 B35 Control group Idiopathic BAD
0.9570938215102975
1020 I11 B39 Control group Idiopathic BAD
0.7986270022883295
1021 I11 B43 Control group Idiopathic BAD
0.9277269260106789
1022 I11 B47 Control group Idiopathic BAD
0.8926392067124332
1023 I11 B48 Control group Idiopathic BAD
0.8756674294431731
1024 I11 B49 Control group Idiopathic BAD
0.7263539282990084
1025 I11 B53 Control group Idiopathic BAD
0.9016018306636155

1026	I11	B54	Control group	Idiopathic BAD	0.9500381388253242
1027	I11	B55	Control group	Idiopathic BAD	0.9353546910755148
1028	I11	B59	Control group	Idiopathic BAD	0.9706331045003814
1029	I11	B70	Control group	Idiopathic BAD	0.9487032799389779
1030	I11	B74	Control group	Idiopathic BAD	0.8945461479786423
1031	I11	B77	Control group	Idiopathic BAD	0.9067505720823799
1032	I11	B81	Control group	Idiopathic BAD	0.8426773455377574
1033	I11	B84	Control group	Idiopathic BAD	0.9126620900076278
1034	I11	B89	Control group	Idiopathic BAD	0.8939740655987796
1035	I11	B92	Control group	Idiopathic BAD	0.8647978642257819
1036	I11	B95	Control group	Idiopathic BAD	0.860602593440122
1037	I11	B99	Control group	Idiopathic BAD	0.8438215102974829
1038	I11	B103	Control group	Idiopathic BAD	0.9416475972540046
1039	I11	B106	Control group	Idiopathic BAD	0.969488939740656
1040	I11	B109	Control group	Idiopathic BAD	0.9124713958810069
1041	I11	B118	Control group	Idiopathic BAD	0.9399313501144165
1042	I11	B119	Control group	Idiopathic BAD	0.9145690312738368
1043	I13	B1	Control group	Idiopathic BAD	0.9128527841342486
1044	I13	B5	Control group	Idiopathic BAD	0.8466819221967964
1045	I13	B6	Control group	Idiopathic BAD	0.746186117467582
1046	I13	B10	Control group	Idiopathic BAD	0.9056064073226545
1047	I13	B17	Control group	Idiopathic BAD	0.8627002288329519
1048	I13	B20	Control group	Idiopathic BAD	0.8451563691838292
1049	I13	B23	Control group	Idiopathic BAD	0.7808924485125858
1050	I13	B31	Control group	Idiopathic BAD	0.855072463768116
1051	I13	B35	Control group	Idiopathic BAD	0.9189549961861174
1052	I13	B39	Control group	Idiopathic BAD	0.8655606407322655

1053	I13	B43	Control group	Idiopathic BAD	0.6922196796338673
1054	I13	B47	Control group	Idiopathic BAD	0.7837528604118993
1055	I13	B48	Control group	Idiopathic BAD	0.9347826086956522
1056	I13	B49	Control group	Idiopathic BAD	0.7612509534706331
1057	I13	B53	Control group	Idiopathic BAD	0.8245614035087719
1058	I13	B54	Control group	Idiopathic BAD	0.8966437833714722
1059	I13	B55	Control group	Idiopathic BAD	0.8577421815408085
1060	I13	B59	Control group	Idiopathic BAD	0.9641495041952708
1061	I13	B70	Control group	Idiopathic BAD	0.9218154080854309
1062	I13	B74	Control group	Idiopathic BAD	0.8260869565217391
1063	I13	B77	Control group	Idiopathic BAD	0.7936689549961862
1064	I13	B81	Control group	Idiopathic BAD	0.8205568268497331
1065	I13	B84	Control group	Idiopathic BAD	0.6777269260106789
1066	I13	B89	Control group	Idiopathic BAD	0.8009153318077803
1067	I13	B92	Control group	Idiopathic BAD	0.7479023646071701
1068	I13	B95	Control group	Idiopathic BAD	0.851258581235698
1069	I13	B99	Control group	Idiopathic BAD	0.6689549961861174
1070	I13	B103	Control group	Idiopathic BAD	0.7835621662852784
1071	I13	B106	Control group	Idiopathic BAD	0.9363081617086194
1072	I13	B109	Control group	Idiopathic BAD	0.8455377574370709
1073	I13	B118	Control group	Idiopathic BAD	0.8724256292906178
1074	I13	B119	Control group	Idiopathic BAD	0.7601067887109078
1075	I15	B1	Control group	Idiopathic BAD	0.8239893211289092
1076	I15	B5	Control group	Idiopathic BAD	0.8054919908466819
1077	I15	B6	Control group	Idiopathic BAD	0.851258581235698
1078	I15	B10	Control group	Idiopathic BAD	0.8346681922196796
1079	I15	B17	Control group	Idiopathic BAD	0.9088482074752098

1080 I15 B20 Control group Idiopathic BAD
0.9057971014492754
1081 I15 B23 Control group Idiopathic BAD
0.7561022120518688
1082 I15 B31 Control group Idiopathic BAD
0.8070175438596491
1083 I15 B35 Control group Idiopathic BAD
0.9834096109839817
1084 I15 B39 Control group Idiopathic BAD
0.9017925247902364
1085 I15 B43 Control group Idiopathic BAD
0.8270404271548436
1086 I15 B47 Control group Idiopathic BAD
0.8516399694889397
1087 I15 B48 Control group Idiopathic BAD
0.8642257818459191
1088 I15 B49 Control group Idiopathic BAD
0.799771167048055
1089 I15 B53 Control group Idiopathic BAD
0.8422959572845157
1090 I15 B54 Control group Idiopathic BAD
0.9364988558352403
1091 I15 B55 Control group Idiopathic BAD
0.8813882532418001
1092 I15 B59 Control group Idiopathic BAD
0.8531655225019069
1093 I15 B70 Control group Idiopathic BAD
0.8142639206712433
1094 I15 B74 Control group Idiopathic BAD
0.9004576659038902
1095 I15 B77 Control group Idiopathic BAD
0.8483981693363845
1096 I15 B81 Control group Idiopathic BAD
0.9056064073226545
1097 I15 B84 Control group Idiopathic BAD
0.6847826086956522
1098 I15 B89 Control group Idiopathic BAD
0.8522120518688024
1099 I15 B92 Control group Idiopathic BAD
0.8007246376811594
1100 I15 B95 Control group Idiopathic BAD
0.992372234935164
1101 I15 B99 Control group Idiopathic BAD
0.7202517162471396
1102 I15 B103 Control group Idiopathic BAD
0.843440122044241
1103 I15 B106 Control group Idiopathic BAD
0.9813119755911518
1104 I15 B109 Control group Idiopathic BAD
0.7986270022883295
1105 I15 B118 Control group Idiopathic BAD
0.9107551487414187
1106 I15 B119 Control group Idiopathic BAD
0.6708619374523265

1107	I17	B1	Control group	Idiopathic BAD	0.9496567505720824
1108	I17	B5	Control group	Idiopathic BAD	0.9128527841342486
1109	I17	B6	Control group	Idiopathic BAD	0.8726163234172387
1110	I17	B10	Control group	Idiopathic BAD	0.8832951945080092
1111	I17	B17	Control group	Idiopathic BAD	0.6630434782608695
1112	I17	B20	Control group	Idiopathic BAD	0.6926010678871091
1113	I17	B23	Control group	Idiopathic BAD	0.839626239511823
1114	I17	B31	Control group	Idiopathic BAD	0.8575514874141876
1115	I17	B35	Control group	Idiopathic BAD	0.9921815408085431
1116	I17	B39	Control group	Idiopathic BAD	0.8956903127383676
1117	I17	B43	Control group	Idiopathic BAD	0.6626620900076278
1118	I17	B47	Control group	Idiopathic BAD	0.8686117467581999
1119	I17	B48	Control group	Idiopathic BAD	0.9620518688024409
1120	I17	B49	Control group	Idiopathic BAD	0.7950038138825324
1121	I17	B53	Control group	Idiopathic BAD	0.8638443935926774
1122	I17	B54	Control group	Idiopathic BAD	0.7284515636918383
1123	I17	B55	Control group	Idiopathic BAD	0.7400839054157132
1124	I17	B59	Control group	Idiopathic BAD	0.9704424103737604
1125	I17	B70	Control group	Idiopathic BAD	0.9168573607932876
1126	I17	B74	Control group	Idiopathic BAD	0.8672768878718535
1127	I17	B77	Control group	Idiopathic BAD	0.8716628527841342
1128	I17	B81	Control group	Idiopathic BAD	0.8327612509534706
1129	I17	B84	Control group	Idiopathic BAD	0.8882532418001525
1130	I17	B89	Control group	Idiopathic BAD	0.8996948893974066
1131	I17	B92	Control group	Idiopathic BAD	0.6666666666666666
1132	I17	B95	Control group	Idiopathic BAD	0.7520976353928299
1133	I17	B99	Control group	Idiopathic BAD	0.7528604118993135

1134	I17	B103	Control group	Idiopathic BAD	0.9061784897025171
1135	I17	B106	Control group	Idiopathic BAD	0.7364607170099161
1136	I17	B109	Control group	Idiopathic BAD	0.8859649122807017
1137	I17	B118	Control group	Idiopathic BAD	0.6617086193745233
1138	I17	B119	Control group	Idiopathic BAD	0.8676582761250954
1139	I18	B1	Control group	Idiopathic BAD	0.8257055682684973
1140	I18	B5	Control group	Idiopathic BAD	0.679252479023646
1141	I18	B6	Control group	Idiopathic BAD	0.8119755911517925
1142	I18	B10	Control group	Idiopathic BAD	0.8226544622425629
1143	I18	B17	Control group	Idiopathic BAD	0.8041571319603357
1144	I18	B20	Control group	Idiopathic BAD	0.7986270022883295
1145	I18	B23	Control group	Idiopathic BAD	0.7936689549961862
1146	I18	B31	Control group	Idiopathic BAD	0.7278794813119756
1147	I18	B35	Control group	Idiopathic BAD	0.9265827612509535
1148	I18	B39	Control group	Idiopathic BAD	0.7982456140350878
1149	I18	B43	Control group	Idiopathic BAD	0.7784134248665141
1150	I18	B47	Control group	Idiopathic BAD	0.7925247902364607
1151	I18	B48	Control group	Idiopathic BAD	0.8800533943554538
1152	I18	B49	Control group	Idiopathic BAD	0.7711670480549199
1153	I18	B53	Control group	Idiopathic BAD	0.8094965675057209
1154	I18	B54	Control group	Idiopathic BAD	0.921624713958810 1
1155	I18	B55	Control group	Idiopathic BAD	0.8710907704042715
1156	I18	B59	Control group	Idiopathic BAD	0.9300152555301296
1157	I18	B70	Control group	Idiopathic BAD	0.7435163996948894
1158	I18	B74	Control group	Idiopathic BAD	0.8230358504958047
1159	I18	B77	Control group	Idiopathic BAD	0.7555301296720061
1160	I18	B81	Control group	Idiopathic BAD	0.7829900839054157

1161	I18	B84	Control group	Idiopathic BAD	0.795766590389016
1162	I18	B89	Control group	Idiopathic BAD	0.7398932112890922
1163	I18	B92	Control group	Idiopathic BAD	0.7126239511823036
1164	I18	B95	Control group	Idiopathic BAD	0.7799389778794813
1165	I18	B99	Control group	Idiopathic BAD	0.6435926773455377
1166	I18	B103	Control group	Idiopathic BAD	0.8884439359267735
1167	I18	B106	Control group	Idiopathic BAD	0.9357360793287567
1168	I18	B109	Control group	Idiopathic BAD	0.7967200610221206
1169	I18	B118	Control group	Idiopathic BAD	0.7797482837528604
1170	I18	B119	Control group	Idiopathic BAD	0.7368421052631579
1171	I19	B1	Control group	Idiopathic BAD	0.9509916094584286
1172	I19	B5	Control group	Idiopathic BAD	0.8939740655987796
1173	I19	B6	Control group	Idiopathic BAD	0.919717772692601
1174	I19	B10	Control group	Idiopathic BAD	0.9309687261632341
1175	I19	B17	Control group	Idiopathic BAD	0.7280701754385965
1176	I19	B20	Control group	Idiopathic BAD	0.7236842105263158
1177	I19	B23	Control group	Idiopathic BAD	0.8995041952707856
1178	I19	B31	Control group	Idiopathic BAD	0.9508009153318078
1179	I19	B35	Control group	Idiopathic BAD	0.9881769641495042
1180	I19	B39	Control group	Idiopathic BAD	0.9477498093058734
1181	I19	B43	Control group	Idiopathic BAD	0.7486651411136537
1182	I19	B47	Control group	Idiopathic BAD	0.8689931350114416
1183	I19	B48	Control group	Idiopathic BAD	0.9809305873379099
1184	I19	B49	Control group	Idiopathic BAD	0.849160945842868
1185	I19	B53	Control group	Idiopathic BAD	0.8485888634630053
1186	I19	B54	Control group	Idiopathic BAD	0.7172006102212052
1187	I19	B55	Control group	Idiopathic BAD	0.7418001525553013

1188	I19	B59	Control group	Idiopathic BAD
0.9563310450038138
1189	I19	B70	Control group	Idiopathic BAD
0.9208619374523265
1190	I19	B74	Control group	Idiopathic BAD
0.9147597254004577
1191	I19	B77	Control group	Idiopathic BAD
0.833905415713196
1192	I19	B81	Control group	Idiopathic BAD
0.8930205949656751
1193	I19	B84	Control group	Idiopathic BAD
0.9014111365369947
1194	I19	B89	Control group	Idiopathic BAD
0.9206712433257056
1195	I19	B92	Control group	Idiopathic BAD
0.7187261632341724
1196	I19	B95	Control group	Idiopathic BAD
0.8289473684210527
1197	I19	B99	Control group	Idiopathic BAD
0.8497330282227308
1198	I19	B103	Control group	Idiopathic BAD
0.9307780320366132
1199	I19	B106	Control group	Idiopathic BAD
0.6826849733028223
1200	I19	B109	Control group	Idiopathic BAD
0.9031273836765827
1201	I19	B118	Control group	Idiopathic BAD
0.7133867276887872
1202	I19	B119	Control group	Idiopathic BAD
0.915903890160183
1203	I22	B1	Control group	Idiopathic BAD
0.8012967200610221
1204	I22	B5	Control group	Idiopathic BAD
0.7393211289092296
1205	I22	B6	Control group	Idiopathic BAD
0.8432494279176201
1206	I22	B10	Control group	Idiopathic BAD
0.7677345537757437
1207	I22	B17	Control group	Idiopathic BAD
0.8258962623951183
1208	I22	B20	Control group	Idiopathic BAD
0.8405797101449275
1209	I22	B23	Control group	Idiopathic BAD
0.8232265446224256
1210	I22	B31	Control group	Idiopathic BAD
0.7578184591914569
1211	I22	B35	Control group	Idiopathic BAD
0.9509916094584286
1212	I22	B39	Control group	Idiopathic BAD
0.7965293668954996
1213	I22	B43	Control group	Idiopathic BAD
0.8112128146453089
1214	I22	B47	Control group	Idiopathic BAD
0.843440122044241

1215 I22 B48 Control group Idiopathic BAD
0.9262013729977117
1216 I22 B49 Control group Idiopathic BAD
0.7906178489702517
1217 I22 B53 Control group Idiopathic BAD
0.8453470633104501
1218 I22 B54 Control group Idiopathic BAD
0.9136155606407322
1219 I22 B55 Control group Idiopathic BAD
0.9054157131960335
1220 I22 B59 Control group Idiopathic BAD
0.8686117467581999
1221 I22 B70 Control group Idiopathic BAD
0.9069412662090007
1222 I22 B74 Control group Idiopathic BAD
0.8257055682684973
1223 I22 B77 Control group Idiopathic BAD
0.8125476735316552
1224 I22 B81 Control group Idiopathic BAD
0.8516399694889397
1225 I22 B84 Control group Idiopathic BAD
0.8318077803203662
1226 I22 B89 Control group Idiopathic BAD
0.7721205186880244
1227 I22 B92 Control group Idiopathic BAD
0.8133104500381388
1228 I22 B95 Control group Idiopathic BAD
0.8762395118230358
1229 I22 B99 Control group Idiopathic BAD
0.7316933638443935
1230 I22 B103 Control group Idiopathic BAD
0.9385964912280702
1231 I22 B106 Control group Idiopathic BAD
0.9549961861174676
1232 I22 B109 Control group Idiopathic BAD
0.8556445461479787
1233 I22 B118 Control group Idiopathic BAD
0.8903508771929824
1234 I22 B119 Control group Idiopathic BAD
0.8297101449275363
1235 I23 B1 Control group Idiopathic BAD
0.8636536994660564
1236 I23 B5 Control group Idiopathic BAD
0.7547673531655225
1237 I23 B6 Control group Idiopathic BAD
0.8398169336384439
1238 I23 B10 Control group Idiopathic BAD
0.7848970251716247
1239 I23 B17 Control group Idiopathic BAD
0.704042715484363
1240 I23 B20 Control group Idiopathic BAD
0.7929061784897025
1241 I23 B23 Control group Idiopathic BAD
0.6823035850495804

1242	I23	B31	Control group	Idiopathic BAD	0.7644927536231884
1243	I23	B35	Control group	Idiopathic BAD	0.9603356216628528
1244	I23	B39	Control group	Idiopathic BAD	0.847254004576659
1245	I23	B43	Control group	Idiopathic BAD	0.835812356979405
1246	I23	B47	Control group	Idiopathic BAD	0.7135774218154081
1247	I23	B48	Control group	Idiopathic BAD	0.9021739130434783
1248	I23	B49	Control group	Idiopathic BAD	0.8068268497330282
1249	I23	B53	Control group	Idiopathic BAD	0.7120518688024409
1250	I23	B54	Control group	Idiopathic BAD	0.8123569794050344
1251	I23	B55	Control group	Idiopathic BAD	0.915903890160183
1252	I23	B59	Control group	Idiopathic BAD	0.9649122807017544
1253	I23	B70	Control group	Idiopathic BAD	0.7688787185354691
1254	I23	B74	Control group	Idiopathic BAD	0.8316170861937452
1255	I23	B77	Control group	Idiopathic BAD	0.8133104500381388
1256	I23	B81	Control group	Idiopathic BAD	0.7673531655225019
1257	I23	B84	Control group	Idiopathic BAD	0.8159801678108314
1258	I23	B89	Control group	Idiopathic BAD	0.7875667429443173
1259	I23	B92	Control group	Idiopathic BAD	0.7553394355453852
1260	I23	B95	Control group	Idiopathic BAD	0.851067887109077
1261	I23	B99	Control group	Idiopathic BAD	0.7051868802440885
1262	I23	B103	Control group	Idiopathic BAD	0.8850114416475973
1263	I23	B106	Control group	Idiopathic BAD	0.9250572082379863
1264	I23	B109	Control group	Idiopathic BAD	0.7883295194508009
1265	I23	B118	Control group	Idiopathic BAD	0.8245614035087719
1266	I23	B119	Control group	Idiopathic BAD	0.7583905415713196
1267	I24	B1	Control group	Idiopathic BAD	0.9578565980167811
1268	I24	B5	Control group	Idiopathic BAD	0.885392829900839

1269	I24	B6	Control group	Idiopathic BAD	0.8617467581998475
1270	I24	B10	Control group	Idiopathic BAD	0.944698703279939
1271	I24	B17	Control group	Idiopathic BAD	0.6458810068649885
1272	I24	B20	Control group	Idiopathic BAD	0.5072463768115942
1273	I24	B23	Control group	Idiopathic BAD	0.8808161708619374
1274	I24	B31	Control group	Idiopathic BAD	0.9250572082379863
1275	I24	B35	Control group	Idiopathic BAD	0.9815026697177727
1276	I24	B39	Control group	Idiopathic BAD	0.9252479023646072
1277	I24	B43	Control group	Idiopathic BAD	0.6027841342486652
1278	I24	B47	Control group	Idiopathic BAD	0.8642257818459191
1279	I24	B48	Control group	Idiopathic BAD	0.9778794813119756
1280	I24	B49	Control group	Idiopathic BAD	0.8627002288329519
1281	I24	B53	Control group	Idiopathic BAD	0.8823417238749046
1282	I24	B54	Control group	Idiopathic BAD	0.6254767353165522
1283	I24	B55	Control group	Idiopathic BAD	0.6573226544622426
1284	I24	B59	Control group	Idiopathic BAD	0.9612890922959573
1285	I24	B70	Control group	Idiopathic BAD	0.9075133485888635
1286	I24	B74	Control group	Idiopathic BAD	0.914187643020595
1287	I24	B77	Control group	Idiopathic BAD	0.868230358504958
1288	I24	B81	Control group	Idiopathic BAD	0.8638443935926774
1289	I24	B84	Control group	Idiopathic BAD	0.9004576659038902
1290	I24	B89	Control group	Idiopathic BAD	0.8344774980930587
1291	I24	B92	Control group	Idiopathic BAD	0.5194508009153318
1292	I24	B95	Control group	Idiopathic BAD	0.6454996186117468
1293	I24	B99	Control group	Idiopathic BAD	0.746186117467582
1294	I24	B103	Control group	Idiopathic BAD	0.9496567505720824
1295	I24	B106	Control group	Idiopathic BAD	0.6588482074752098

1296 I24 B109 Control group Idiopathic BAD
0.8470633104500381
1297 I24 B118 Control group Idiopathic BAD
0.5444317315026698
1298 I24 B119 Control group Idiopathic BAD
0.8518306636155606
1299 I25 B1 Control group Idiopathic BAD
0.8647978642257819
1300 I25 B5 Control group Idiopathic BAD
0.7797482837528604
1301 I25 B6 Control group Idiopathic BAD
0.7774599542334096
1302 I25 B10 Control group Idiopathic BAD
0.8766209000762777
1303 I25 B17 Control group Idiopathic BAD
0.813119755911518
1304 I25 B20 Control group Idiopathic BAD
0.706140350877193
1305 I25 B23 Control group Idiopathic BAD
0.7988176964149504
1306 I25 B31 Control group Idiopathic BAD
0.841723874904653
1307 I25 B35 Control group Idiopathic BAD
0.919908466819222
1308 I25 B39 Control group Idiopathic BAD
0.8735697940503433
1309 I25 B43 Control group Idiopathic BAD
0.7885202135774219
1310 I25 B47 Control group Idiopathic BAD
0.7812738367658276
1311 I25 B48 Control group Idiopathic BAD
0.8840579710144928
1312 I25 B49 Control group Idiopathic BAD
0.6538901601830663
1313 I25 B53 Control group Idiopathic BAD
0.8304729214340199
1314 I25 B54 Control group Idiopathic BAD
0.8621281464530892
1315 I25 B55 Control group Idiopathic BAD
0.88558352402746
1316 I25 B59 Control group Idiopathic BAD
0.9725400457665904
1317 I25 B70 Control group Idiopathic BAD
0.8935926773455377
1318 I25 B74 Control group Idiopathic BAD
0.8203661327231121
1319 I25 B77 Control group Idiopathic BAD
0.791952707856598
1320 I25 B81 Control group Idiopathic BAD
0.7467581998474447
1321 I25 B84 Control group Idiopathic BAD
0.8504958047292144
1322 I25 B89 Control group Idiopathic BAD
0.7254004576659039

1323	I25	B92	Control group	Idiopathic BAD	0.7820366132723112
1324	I25	B95	Control group	Idiopathic BAD	0.7906178489702517
1325	I25	B99	Control group	Idiopathic BAD	0.6454996186117468
1326	I25	B103	Control group	Idiopathic BAD	0.8924485125858124
1327	I25	B106	Control group	Idiopathic BAD	0.9262013729977117
1328	I25	B109	Control group	Idiopathic BAD	0.7892829900839055
1329	I25	B118	Control group	Idiopathic BAD	0.8293287566742944
1330	I25	B119	Control group	Idiopathic BAD	0.7435163996948894
1331	I26	B1	Control group	Idiopathic BAD	0.9187643020594966
1332	I26	B5	Control group	Idiopathic BAD	0.7698321891685737
1333	I26	B6	Control group	Idiopathic BAD	0.8051106025934401
1334	I26	B10	Control group	Idiopathic BAD	0.8867276887871853
1335	I26	B17	Control group	Idiopathic BAD	0.8203661327231121
1336	I26	B20	Control group	Idiopathic BAD	0.761632341723875
1337	I26	B23	Control group	Idiopathic BAD	0.7326468344774981
1338	I26	B31	Control group	Idiopathic BAD	0.8285659801678108
1339	I26	B35	Control group	Idiopathic BAD	0.9284897025171625
1340	I26	B39	Control group	Idiopathic BAD	0.8308543096872616
1341	I26	B43	Control group	Idiopathic BAD	0.7501906941266209
1342	I26	B47	Control group	Idiopathic BAD	0.669908466819222
1343	I26	B48	Control group	Idiopathic BAD	0.9016018306636155
1344	I26	B49	Control group	Idiopathic BAD	0.8485888634630053
1345	I26	B53	Control group	Idiopathic BAD	0.8466819221967964
1346	I26	B54	Control group	Idiopathic BAD	0.8342868039664378
1347	I26	B55	Control group	Idiopathic BAD	0.8865369946605645
1348	I26	B59	Control group	Idiopathic BAD	0.9927536231884058
1349	I26	B70	Control group	Idiopathic BAD	0.7690694126620901

1350 I26 B74 Control group Idiopathic BAD
0.8577421815408085
1351 I26 B77 Control group Idiopathic BAD
0.7564836003051106
1352 I26 B81 Control group Idiopathic BAD
0.8066361556064073
1353 I26 B84 Control group Idiopathic BAD
0.5776125095347063
1354 I26 B89 Control group Idiopathic BAD
0.7618230358504958
1355 I26 B92 Control group Idiopathic BAD
0.7765064836003052
1356 I26 B95 Control group Idiopathic BAD
0.8586956521739131
1357 I26 B99 Control group Idiopathic BAD
0.6941266209000763
1358 I26 B103 Control group Idiopathic BAD
0.7864225781845919
1359 I26 B106 Control group Idiopathic BAD
0.9487032799389779
1360 I26 B109 Control group Idiopathic BAD
0.7381769641495042
1361 I26 B118 Control group Idiopathic BAD
0.8756674294431731
1362 I26 B119 Control group Idiopathic BAD
0.6863081617086194
1363 I27 B1 Control group Idiopathic BAD
0.9086575133485889
1364 I27 B5 Control group Idiopathic BAD
0.6687643020594966
1365 I27 B6 Control group Idiopathic BAD
0.8375286041189931
1366 I27 B10 Control group Idiopathic BAD
0.9252479023646072
1367 I27 B17 Control group Idiopathic BAD
0.8041571319603357
1368 I27 B20 Control group Idiopathic BAD
0.8213196033562167
1369 I27 B23 Control group Idiopathic BAD
0.7151029748283753
1370 I27 B31 Control group Idiopathic BAD
0.7948131197559115
1371 I27 B35 Control group Idiopathic BAD
0.8571700991609459
1372 I27 B39 Control group Idiopathic BAD
0.7690694126620901
1373 I27 B43 Control group Idiopathic BAD
0.8232265446224256
1374 I27 B47 Control group Idiopathic BAD
0.7374141876430206
1375 I27 B48 Control group Idiopathic BAD
0.7734553775743707
1376 I27 B49 Control group Idiopathic BAD
0.7911899313501144

1377	I27	B53	Control group	Idiopathic BAD	0.7618230358504958
1378	I27	B54	Control group	Idiopathic BAD	0.8827231121281465
1379	I27	B55	Control group	Idiopathic BAD	0.8585049580472921
1380	I27	B59	Control group	Idiopathic BAD	0.986651411136537
1381	I27	B70	Control group	Idiopathic BAD	0.7763157894736842
1382	I27	B74	Control group	Idiopathic BAD	0.88558352402746
1383	I27	B77	Control group	Idiopathic BAD	0.7063310450038138
1384	I27	B81	Control group	Idiopathic BAD	0.7877574370709383
1385	I27	B84	Control group	Idiopathic BAD	0.8072082379862701
1386	I27	B89	Control group	Idiopathic BAD	0.7215865751334859
1387	I27	B92	Control group	Idiopathic BAD	0.7585812356979404
1388	I27	B95	Control group	Idiopathic BAD	0.8028222730739893
1389	I27	B99	Control group	Idiopathic BAD	0.6510297482837528
1390	I27	B103	Control group	Idiopathic BAD	0.9014111365369947
1391	I27	B106	Control group	Idiopathic BAD	0.9467963386727689
1392	I27	B109	Control group	Idiopathic BAD	0.7419908466819222
1393	I27	B118	Control group	Idiopathic BAD	0.7883295194508009
1394	I27	B119	Control group	Idiopathic BAD	0.6945080091533181
1395	I28	B1	Control group	Idiopathic BAD	0.8203661327231121
1396	I28	B5	Control group	Idiopathic BAD	0.7890922959572845
1397	I28	B6	Control group	Idiopathic BAD	0.88558352402746
1398	I28	B10	Control group	Idiopathic BAD	0.8789092295957285
1399	I28	B17	Control group	Idiopathic BAD	0.9075133485888635
1400	I28	B20	Control group	Idiopathic BAD	0.9279176201372997
1401	I28	B23	Control group	Idiopathic BAD	0.8956903127383676
1402	I28	B31	Control group	Idiopathic BAD	0.7192982456140351
1403	I28	B35	Control group	Idiopathic BAD	0.6332951945080092

1404	I28	B39	Control group	Idiopathic BAD	0.8531655225019069
1405	I28	B43	Control group	Idiopathic BAD	0.8951182303585049
1406	I28	B47	Control group	Idiopathic BAD	0.9246758199847445
1407	I28	B48	Control group	Idiopathic BAD	0.6767734553775744
1408	I28	B49	Control group	Idiopathic BAD	0.7305491990846682
1409	I28	B53	Control group	Idiopathic BAD	0.937070938215103
1410	I28	B54	Control group	Idiopathic BAD	0.9609077040427155
1411	I28	B55	Control group	Idiopathic BAD	0.9061784897025171
1412	I28	B59	Control group	Idiopathic BAD	0.9357360793287567
1413	I28	B70	Control group	Idiopathic BAD	0.9437452326468345
1414	I28	B74	Control group	Idiopathic BAD	0.8735697940503433
1415	I28	B77	Control group	Idiopathic BAD	0.9294431731502669
1416	I28	B81	Control group	Idiopathic BAD	0.6531273836765827
1417	I28	B84	Control group	Idiopathic BAD	0.8758581235697941
1418	I28	B89	Control group	Idiopathic BAD	0.793859649122807
1419	I28	B92	Control group	Idiopathic BAD	0.8539282990083905
1420	I28	B95	Control group	Idiopathic BAD	0.8565980167810832
1421	I28	B99	Control group	Idiopathic BAD	0.8247520976353928
1422	I28	B103	Control group	Idiopathic BAD	0.8993135011441648
1423	I28	B106	Control group	Idiopathic BAD	0.9626239511823036
1424	I28	B109	Control group	Idiopathic BAD	0.8790999237223494
1425	I28	B118	Control group	Idiopathic BAD	0.8916857360793288
1426	I28	B119	Control group	Idiopathic BAD	0.8211289092295957
1427	I29	B1	Control group	Idiopathic BAD	0.9252479023646072
1428	I29	B5	Control group	Idiopathic BAD	0.8796720061022121
1429	I29	B6	Control group	Idiopathic BAD	0.8394355453852022
1430	I29	B10	Control group	Idiopathic BAD	0.9185736079328757

1431	I29	B17	Control group	Idiopathic BAD	0.9061784897025171
1432	I29	B20	Control group	Idiopathic BAD	0.851067887109077
1433	I29	B23	Control group	Idiopathic BAD	0.8731884057971014
1434	I29	B31	Control group	Idiopathic BAD	0.9138062547673532
1435	I29	B35	Control group	Idiopathic BAD	0.969488939740656
1436	I29	B39	Control group	Idiopathic BAD	0.9319221967963387
1437	I29	B43	Control group	Idiopathic BAD	0.8121662852784134
1438	I29	B47	Control group	Idiopathic BAD	0.8371472158657514
1439	I29	B48	Control group	Idiopathic BAD	0.9685354691075515
1440	I29	B49	Control group	Idiopathic BAD	0.7458047292143402
1441	I29	B53	Control group	Idiopathic BAD	0.8495423340961098
1442	I29	B54	Control group	Idiopathic BAD	0.9210526315789473
1443	I29	B55	Control group	Idiopathic BAD	0.9300152555301296
1444	I29	B59	Control group	Idiopathic BAD	0.9837909992372235
1445	I29	B70	Control group	Idiopathic BAD	0.8707093821510298
1446	I29	B74	Control group	Idiopathic BAD	0.8716628527841342
1447	I29	B77	Control group	Idiopathic BAD	0.8438215102974829
1448	I29	B81	Control group	Idiopathic BAD	0.799771167048055
1449	I29	B84	Control group	Idiopathic BAD	0.8766209000762777
1450	I29	B89	Control group	Idiopathic BAD	0.8226544622425629
1451	I29	B92	Control group	Idiopathic BAD	0.856788710907704
1452	I29	B95	Control group	Idiopathic BAD	0.8138825324180016
1453	I29	B99	Control group	Idiopathic BAD	0.7095728451563692
1454	I29	B103	Control group	Idiopathic BAD	0.9006483600305111
1455	I29	B106	Control group	Idiopathic BAD	0.9506102212051869
1456	I29	B109	Control group	Idiopathic BAD	0.7873760488176964
1457	I29	B118	Control group	Idiopathic BAD	0.8678489702517163

1458 I29 B119 Control group Idiopathic BAD
0.7971014492753623
1459 I30 B1 Control group Idiopathic BAD
0.8876811594202898
1460 I30 B5 Control group Idiopathic BAD
0.6924103737604882
1461 I30 B6 Control group Idiopathic BAD
0.8546910755148741
1462 I30 B10 Control group Idiopathic BAD
0.9164759725400458
1463 I30 B17 Control group Idiopathic BAD
0.8619374523264683
1464 I30 B20 Control group Idiopathic BAD
0.8548817696414951
1465 I30 B23 Control group Idiopathic BAD
0.7152936689549961
1466 I30 B31 Control group Idiopathic BAD
0.812929061784897
1467 I30 B35 Control group Idiopathic BAD
0.9494660564454614
1468 I30 B39 Control group Idiopathic BAD
0.8464912280701754
1469 I30 B43 Control group Idiopathic BAD
0.8445842868039665
1470 I30 B47 Control group Idiopathic BAD
0.8077803203661327
1471 I30 B48 Control group Idiopathic BAD
0.7713577421815409
1472 I30 B49 Control group Idiopathic BAD
0.8924485125858124
1473 I30 B53 Control group Idiopathic BAD
0.7827993897787948
1474 I30 B54 Control group Idiopathic BAD
0.9128527841342486
1475 I30 B55 Control group Idiopathic BAD
0.855072463768116
1476 I30 B59 Control group Idiopathic BAD
0.975209763539283
1477 I30 B70 Control group Idiopathic BAD
0.7292143401983219
1478 I30 B74 Control group Idiopathic BAD
0.8710907704042715
1479 I30 B77 Control group Idiopathic BAD
0.8039664378337147
1480 I30 B81 Control group Idiopathic BAD
0.7993897787948131
1481 I30 B84 Control group Idiopathic BAD
0.8106407322654462
1482 I30 B89 Control group Idiopathic BAD
0.8081617086193745
1483 I30 B92 Control group Idiopathic BAD
0.8293287566742944
1484 I30 B95 Control group Idiopathic BAD
0.8947368421052632

1485	I30	B99	Control group	Idiopathic BAD
0.7551487414187643
1486	I30	B103	Control group	Idiopathic BAD
0.9193363844393593
1487	I30	B106	Control group	Idiopathic BAD
0.971205186880244
1488	I30	B109	Control group	Idiopathic BAD
0.742372234935164
1489	I30	B118	Control group	Idiopathic BAD
0.8752860411899314
1490	I30	B119	Control group	Idiopathic BAD
0.5696033562166285
1491	I31	B1	Control group	Idiopathic BAD
0.9412662090007627
1492	I31	B5	Control group	Idiopathic BAD
0.908276125095347
1493	I31	B6	Control group	Idiopathic BAD
0.8493516399694889
1494	I31	B10	Control group	Idiopathic BAD
0.8935926773455377
1495	I31	B17	Control group	Idiopathic BAD
0.8508771929824561
1496	I31	B20	Control group	Idiopathic BAD
0.8676582761250954
1497	I31	B23	Control group	Idiopathic BAD
0.8935926773455377
1498	I31	B31	Control group	Idiopathic BAD
0.8541189931350115
1499	I31	B35	Control group	Idiopathic BAD
0.9889397406559878
1500	I31	B39	Control group	Idiopathic BAD
0.8747139588100686
1501	I31	B43	Control group	Idiopathic BAD
0.8104500381388253
1502	I31	B47	Control group	Idiopathic BAD
0.8710907704042715
1503	I31	B48	Control group	Idiopathic BAD
0.9221967963386728
1504	I31	B49	Control group	Idiopathic BAD
0.7877574370709383
1505	I31	B53	Control group	Idiopathic BAD
0.8703279938977879
1506	I31	B54	Control group	Idiopathic BAD
0.8600305110602593
1507	I31	B55	Control group	Idiopathic BAD
0.9437452326468345
1508	I31	B59	Control group	Idiopathic BAD
0.9973302822273074
1509	I31	B70	Control group	Idiopathic BAD
0.9647215865751335
1510	I31	B74	Control group	Idiopathic BAD
0.8996948893974066
1511	I31	B77	Control group	Idiopathic BAD
0.910373760488177

1512	I31	B81	Control group	Idiopathic BAD	0.8508771929824561
1513	I31	B84	Control group	Idiopathic BAD	0.9265827612509535
1514	I31	B89	Control group	Idiopathic BAD	0.82627765064836
1515	I31	B92	Control group	Idiopathic BAD	0.8728070175438597
1516	I31	B95	Control group	Idiopathic BAD	0.8539282990083905
1517	I31	B99	Control group	Idiopathic BAD	0.7852784134248665
1518	I31	B103	Control group	Idiopathic BAD	0.9525171624713958
1519	I31	B106	Control group	Idiopathic BAD	0.9603356216628528
1520	I31	B109	Control group	Idiopathic BAD	0.8165522501906941
1521	I31	B118	Control group	Idiopathic BAD	0.8884439359267735
1522	I31	B119	Control group	Idiopathic BAD	0.8459191456903128
1523	I32	B1	Control group	Idiopathic BAD	0.9441266209000763
1524	I32	B5	Control group	Idiopathic BAD	0.8665141113653699
1525	I32	B6	Control group	Idiopathic BAD	0.8123569794050344
1526	I32	B10	Control group	Idiopathic BAD	0.9464149504195271
1527	I32	B17	Control group	Idiopathic BAD	0.9038901601830663
1528	I32	B20	Control group	Idiopathic BAD	0.8716628527841342
1529	I32	B23	Control group	Idiopathic BAD	0.8375286041189931
1530	I32	B31	Control group	Idiopathic BAD	0.8598398169336384
1531	I32	B35	Control group	Idiopathic BAD	0.9508009153318078
1532	I32	B39	Control group	Idiopathic BAD	0.9031273836765827
1533	I32	B43	Control group	Idiopathic BAD	0.7889016018306636
1534	I32	B47	Control group	Idiopathic BAD	0.9437452326468345
1535	I32	B48	Control group	Idiopathic BAD	0.9347826086956522
1536	I32	B49	Control group	Idiopathic BAD	0.7982456140350878
1537	I32	B53	Control group	Idiopathic BAD	0.9506102212051869
1538	I32	B54	Control group	Idiopathic BAD	0.9557589626239512

1539	I32	B55	Control group	Idiopathic BAD
0.9576659038901602
1540	I32	B59	Control group	Idiopathic BAD
0.9887490465293669
1541	I32	B70	Control group	Idiopathic BAD
0.9845537757437071
1542	I32	B74	Control group	Idiopathic BAD
0.9324942791762014
1543	I32	B77	Control group	Idiopathic BAD
0.9279176201372997
1544	I32	B81	Control group	Idiopathic BAD
0.8710907704042715
1545	I32	B84	Control group	Idiopathic BAD
0.9000762776506483
1546	I32	B89	Control group	Idiopathic BAD
0.7940503432494279
1547	I32	B92	Control group	Idiopathic BAD
0.8558352402745996
1548	I32	B95	Control group	Idiopathic BAD
0.8802440884820748
1549	I32	B99	Control group	Idiopathic BAD
0.7339816933638444
1550	I32	B103	Control group	Idiopathic BAD
0.8720442410373761
1551	I32	B106	Control group	Idiopathic BAD
0.9553775743707094
1552	I32	B109	Control group	Idiopathic BAD
0.8733790999237223
1553	I32	B118	Control group	Idiopathic BAD
0.8945461479786423
1554	I32	B119	Control group	Idiopathic BAD
0.7978642257818459
1555	I33	B1	Control group	Idiopathic BAD
0.910373760488177
1556	I33	B5	Control group	Idiopathic BAD
0.8121662852784134
1557	I33	B6	Control group	Idiopathic BAD
0.8621281464530892
1558	I33	B10	Control group	Idiopathic BAD
0.9323035850495804
1559	I33	B17	Control group	Idiopathic BAD
0.6273836765827613
1560	I33	B20	Control group	Idiopathic BAD
0.5192601067887109
1561	I33	B23	Control group	Idiopathic BAD
0.8373379099923722
1562	I33	B31	Control group	Idiopathic BAD
0.9036994660564455
1563	I33	B35	Control group	Idiopathic BAD
0.954042715484363
1564	I33	B39	Control group	Idiopathic BAD
0.8991228070175439
1565	I33	B43	Control group	Idiopathic BAD
0.6426392067124332

1566 I33 B47 Control group Idiopathic BAD
0.8119755911517925
1567 I33 B48 Control group Idiopathic BAD
0.9399313501144165
1568 I33 B49 Control group Idiopathic BAD
0.8030129672006102
1569 I33 B53 Control group Idiopathic BAD
0.8268497330282227
1570 I33 B54 Control group Idiopathic BAD
0.6235697940503433
1571 I33 B55 Control group Idiopathic BAD
0.6462623951182304
1572 I33 B59 Control group Idiopathic BAD
0.9755911517925248
1573 I33 B70 Control group Idiopathic BAD
0.8972158657513348
1574 I33 B74 Control group Idiopathic BAD
0.9054157131960335
1575 I33 B77 Control group Idiopathic BAD
0.7955758962623951
1576 I33 B81 Control group Idiopathic BAD
0.8745232646834478
1577 I33 B84 Control group Idiopathic BAD
0.8703279938977879
1578 I33 B89 Control group Idiopathic BAD
0.7795575896262396
1579 I33 B92 Control group Idiopathic BAD
0.4929443173150267
1580 I33 B95 Control group Idiopathic BAD
0.6155606407322655
1581 I33 B99 Control group Idiopathic BAD
0.7126239511823036
1582 I33 B103 Control group Idiopathic BAD
0.9342105263157895
1583 I33 B106 Control group Idiopathic BAD
0.6742944317315027
1584 I33 B109 Control group Idiopathic BAD
0.8649885583524027
1585 I33 B118 Control group Idiopathic BAD
0.5327993897787948
1586 I33 B119 Control group Idiopathic BAD
0.8333333333333334
1587 I34 B1 Control group Idiopathic BAD
0.9380244088482075
1588 I34 B5 Control group Idiopathic BAD
0.8213196033562167
1589 I34 B6 Control group Idiopathic BAD
0.8947368421052632
1590 I34 B10 Control group Idiopathic BAD
0.8802440884820748
1591 I34 B17 Control group Idiopathic BAD
0.7116704805491991
1592 I34 B20 Control group Idiopathic BAD
0.6805873379099924

1593	I34	B23	Control group	Idiopathic BAD	0.700419527078566
1594	I34	B31	Control group	Idiopathic BAD	0.8094965675057209
1595	I34	B35	Control group	Idiopathic BAD	0.9662471395881007
1596	I34	B39	Control group	Idiopathic BAD	0.846872616323417З
1597	I34	B43	Control group	Idiopathic BAD	0.7484744469870328
1598	I34	B47	Control group	Idiopathic BAD	0.7745995423340961
1599	I34	B48	Control group	Idiopathic BAD	0.9069412662090007
1600	I34	B49	Control group	Idiopathic BAD	0.8846300533943554
1601	I34	B53	Control group	Idiopathic BAD	0.8274218154080855
1602	I34	B54	Control group	Idiopathic BAD	0.7759344012204424
1603	I34	B55	Control group	Idiopathic BAD	0.6493135011441648
1604	I34	B59	Control group	Idiopathic BAD	0.9803585049580473
1605	I34	B70	Control group	Idiopathic BAD	0.8203661327231121
1606	I34	B74	Control group	Idiopathic BAD	0.876048817696415
1607	I34	B77	Control group	Idiopathic BAD	0.7784134248665141
1608	I34	B81	Control group	Idiopathic BAD	0.9061784897025171
1609	I34	B84	Control group	Idiopathic BAD	0.7972921434019832
1610	I34	B89	Control group	Idiopathic BAD	0.8810068649885584
1611	I34	B92	Control group	Idiopathic BAD	0.6760106788710908
1612	I34	B95	Control group	Idiopathic BAD	0.7160564454614798
1613	I34	B99	Control group	Idiopathic BAD	0.8123569794050344
1614	I34	B103	Control group	Idiopathic BAD	0.8647978642257819
1615	I34	B106	Control group	Idiopathic BAD	0.876048817696415
1616	I34	B109	Control group	Idiopathic BAD	0.8356216628527842
1617	I34	B118	Control group	Idiopathic BAD	0.7391304347826086
1618	I34	B119	Control group	Idiopathic BAD	0.7988176964149504
1619	I35	B1	Control group	Idiopathic BAD	0.8560259344012204

1620	I35	B5	Control group	Idiopathic BAD	0.8438215102974829
1621	I35	B6	Control group	Idiopathic BAD	0.811022120518688
1622	I35	B10	Control group	Idiopathic BAD	0.8564073226544623
1623	I35	B17	Control group	Idiopathic BAD	0.8253241800152555
1624	I35	B20	Control group	Idiopathic BAD	0.8291380625476735
1625	I35	B23	Control group	Idiopathic BAD	0.7473302822273074
1626	I35	B31	Control group	Idiopathic BAD	0.7929061784897025
1627	I35	B35	Control group	Idiopathic BAD	0.8634630053394355
1628	I35	B39	Control group	Idiopathic BAD	0.8371472158657514
1629	I35	B43	Control group	Idiopathic BAD	0.8241800152555301
1630	I35	B47	Control group	Idiopathic BAD	0.8377192982456141
1631	I35	B48	Control group	Idiopathic BAD	0.8729977116704806
1632	I35	B49	Control group	Idiopathic BAD	0.6725781845919145
1633	I35	B53	Control group	Idiopathic BAD	0.8157894736842105
1634	I35	B54	Control group	Idiopathic BAD	0.8838672768878718
1635	I35	B55	Control group	Idiopathic BAD	0.9496567505720824
1636	I35	B59	Control group	Idiopathic BAD	0.9889397406559878
1637	I35	B70	Control group	Idiopathic BAD	0.894927536231884
1638	I35	B74	Control group	Idiopathic BAD	0.8592677345537757
1639	I35	B77	Control group	Idiopathic BAD	0.8649885583524027
1640	I35	B81	Control group	Idiopathic BAD	0.7486651411136537
1641	I35	B84	Control group	Idiopathic BAD	0.8878718535469108
1642	I35	B89	Control group	Idiopathic BAD	0.6836384439359268
1643	I35	B92	Control group	Idiopathic BAD	0.7675438596491229
1644	I35	B95	Control group	Idiopathic BAD	0.7868039664378337
1645	I35	B99	Control group	Idiopathic BAD	0.6950800915331807
1646	I35	B103	Control group	Idiopathic BAD	0.8447749809305873

1647	I35	B106	Control group	Idiopathic BAD	0.9477498093058734
1648	I35	B109	Control group	Idiopathic BAD	0.9042715484363082
1649	I35	B118	Control group	Idiopathic BAD	0.8466819221967964
1650	I35	B119	Control group	Idiopathic BAD	0.7984363081617086
1651	I36	B1	Control group	Idiopathic BAD	0.8918764302059496
1652	I36	B5	Control group	Idiopathic BAD	0.9263920671243325
1653	I36	B6	Control group	Idiopathic BAD	0.9956140350877193
1654	I36	B10	Control group	Idiopathic BAD	0.7637299771167048
1655	I36	B17	Control group	Idiopathic BAD	0.9963768115942029
1656	I36	B20	Control group	Idiopathic BAD	0.9979023646071701
1657	I36	B23	Control group	Idiopathic BAD	0.9935163996948894
1658	I36	B31	Control group	Idiopathic BAD	0.8913043478260869
1659	I36	B35	Control group	Idiopathic BAD	0.9950419527078566
1660	I36	B39	Control group	Idiopathic BAD	0.9660564454614798
1661	I36	B43	Control group	Idiopathic BAD	0.9977116704805492
1662	I36	B47	Control group	Idiopathic BAD	0.9855072463768116
1663	I36	B48	Control group	Idiopathic BAD	0.9868421052631579
1664	I36	B49	Control group	Idiopathic BAD	0.9935163996948894
1665	I36	B53	Control group	Idiopathic BAD	0.9935163996948894
1666	I36	B54	Control group	Idiopathic BAD	0.9984744469870328
1667	I36	B55	Control group	Idiopathic BAD	0.994279176201373
1668	I36	B59	Control group	Idiopathic BAD	0.8011060259344012
1669	I36	B70	Control group	Idiopathic BAD	0.9855072463768116
1670	I36	B74	Control group	Idiopathic BAD	0.9971395881006865
1671	I36	B77	Control group	Idiopathic BAD	0.9891304347826086
1672	I36	B81	Control group	Idiopathic BAD	0.990465293668955
1673	I36	B84	Control group	Idiopathic BAD	0.9157131960335622

1674	I36	B89	Control group	Idiopathic BAD	0.988367658276125
1675	I36	B92	Control group	Idiopathic BAD	0.956140350877193
1676	I36	B95	Control group	Idiopathic BAD	1.0
1677	I36	B99	Control group	Idiopathic BAD	0.9956140350877193
1678	I36	B103	Control group	Idiopathic BAD	0.9887490465293669
1679	I36	B106	Control group	Idiopathic BAD	0.9996186117467581
1680	I36	B109	Control group	Idiopathic BAD	0.9998093058733791
1681	I36	B118	Control group	Idiopathic BAD	0.9994279176201373
1682	I36	B119	Control group	Idiopathic BAD	0.992372234935164
1683	I37	B1	Control group	Idiopathic BAD	0.7395118230358505
1684	I37	B5	Control group	Idiopathic BAD	0.8741418764302059
1685	I37	B6	Control group	Idiopathic BAD	0.8632723112128147
1686	I37	B10	Control group	Idiopathic BAD	0.5223112128146453
1687	I37	B17	Control group	Idiopathic BAD	0.855072463768116
1688	I37	B20	Control group	Idiopathic BAD	0.9439359267734554
1689	I37	B23	Control group	Idiopathic BAD	0.8443935926773455
1690	I37	B31	Control group	Idiopathic BAD	0.7475209763539283
1691	I37	B35	Control group	Idiopathic BAD	0.9189549961861174
1692	I37	B39	Control group	Idiopathic BAD	0.8699466056445462
1693	I37	B43	Control group	Idiopathic BAD	0.9424103737604882
1694	I37	B47	Control group	Idiopathic BAD	0.8983600305110603
1695	I37	B48	Control group	Idiopathic BAD	0.898741418764302
1696	I37	B49	Control group	Idiopathic BAD	0.8960717009916095
1697	I37	B53	Control group	Idiopathic BAD	0.8958810068649885
1698	I37	B54	Control group	Idiopathic BAD	0.9544241037376049
1699	I37	B55	Control group	Idiopathic BAD	0.9683447749809306
1700	I37	B59	Control group	Idiopathic BAD	0.8012967200610221
1701	I37	B70	Control group	Idiopathic BAD	

0.9527078565980168
1702 I37 B74 Control group Idiopathic BAD
0.8575514874141876
1703 I37 B77 Control group Idiopathic BAD
0.9105644546147978
1704 I37 B81 Control group Idiopathic BAD
0.8827231121281465
1705 I37 B84 Control group Idiopathic BAD
0.8670861937452327
1706 I37 B89 Control group Idiopathic BAD
0.8979786422578184
1707 I37 B92 Control group Idiopathic BAD
0.8531655225019069
1708 I37 B95 Control group Idiopathic BAD
0.9420289855072463
1709 I37 B99 Control group Idiopathic BAD
0.9130434782608695
1710 I37 B103 Control group Idiopathic BAD
0.9250572082379863
1711 I37 B106 Control group Idiopathic BAD
0.9691075514874142
1712 I37 B109 Control group Idiopathic BAD
0.9559496567505721
1713 I37 B118 Control group Idiopathic BAD
0.9445080091533181
1714 I37 B119 Control group Idiopathic BAD
0.8745232646834478
1715 B27 P1 Control group Post-cholecystectomy
0.9475591151792525
1716 B27 P2 Control group Post-cholecystectomy
0.8171243325705568
1717 B27 P4 Control group Post-cholecystectomy
0.7963386727688787
1718 B27 P5 Control group Post-cholecystectomy
0.704042715484363
1719 B27 P9 Control group Post-cholecystectomy
0.8163615560640732
1720 B27 P13 Control group Post-cholecystectomy
0.8720442410373761
1721 B27 P15 Control group Post-cholecystectomy
0.7196796338672768
1722 B27 P16 Control group Post-cholecystectomy
0.8422959572845157
1723 B27 P17 Control group Post-cholecystectomy
0.8668954996186118
1724 B27 P20 Control group Post-cholecystectomy
0.8560259344012204
1725 B27 P21 Control group Post-cholecystectomy
0.8686117467581999
1726 B27 P24 Control group Post-cholecystectomy
0.8369565217391305
1727 B27 P26 Control group Post-cholecystectomy
0.931350114416476
1728 B27 P30 Control group Post-cholecystectomy

0.8274218154080855
1729 B27 P33 Control group Post-cholecystectomy
0.835812356979405
1730 B27 P35 Control group Post-cholecystectomy
0.8655606407322655
1731 B27 P38 Control group Post-cholecystectomy
0.9393592677345538
1732 B27 P39 Control group Post-cholecystectomy
0.9101830663615561
1733 B27 P42 Control group Post-cholecystectomy
0.919908466819222
1734 B27 P43 Control group Post-cholecystectomy
0.8384820747520977
1735 B27 P46 Control group Post-cholecystectomy
0.7143401983218917
1736 B27 P47 Control group Post-cholecystectomy
0.5467200610221206
1737 B27 P50 Control group Post-cholecystectomy
0.7770785659801678
1738 B27 P55 Control group Post-cholecystectomy
0.7469488939740656
1739 B27 P58 Control group Post-cholecystectomy
0.8569794050343249
1740 B27 P60 Control group Post-cholecystectomy
0.8745232646834478
1741 B27 P63 Control group Post-cholecystectomy
0.8314263920671243
1742 B27 P65 Control group Post-cholecystectomy
0.8916857360793288
1743 B27 P68 Control group Post-cholecystectomy
0.8335240274599542
1744 B27 P70 Control group Post-cholecystectomy
0.9105644546147978
1745 B27 P71 Control group Post-cholecystectomy
0.7980549199084668
1746 B27 P74 Control group Post-cholecystectomy
0.860602593440122
1747 B27 P75 Control group Post-cholecystectomy
0.7740274599542334
1748 B66 P1 Control group Post-cholecystectomy
0.931350114416476
1749 B66 P2 Control group Post-cholecystectomy
0.7745995423340961
1750 B66 P4 Control group Post-cholecystectomy
0.761632341723875
1751 B66 P5 Control group Post-cholecystectomy
0.7818459191456903
1752 B66 P9 Control group Post-cholecystectomy
0.8001525553012967
1753 B66 P13 Control group Post-cholecystectomy
0.860602593440122
1754 B66 P15 Control group Post-cholecystectomy
0.7908085430968727
1755 B66 P16 Control group Post-cholecystectomy

0.719488939740656
1756 B66 P17 Control group Post-cholecystectomy
0.8133104500381388
1757 B66 P20 Control group Post-cholecystectomy
0.8253241800152555
1758 B66 P21 Control group Post-cholecystectomy
0.6802059496567505
1759 B66 P24 Control group Post-cholecystectomy
0.8552631578947368
1760 B66 P26 Control group Post-cholecystectomy
0.6769641495041953
1761 B66 P30 Control group Post-cholecystectomy
0.8901601830663616
1762 B66 P33 Control group Post-cholecystectomy
0.7778413424866514
1763 B66 P35 Control group Post-cholecystectomy
0.7181540808543097
1764 B66 P38 Control group Post-cholecystectomy
0.8157894736842105
1765 B66 P39 Control group Post-cholecystectomy
0.8459191456903128
1766 B66 P42 Control group Post-cholecystectomy
0.8011060259344012
1767 B66 P43 Control group Post-cholecystectomy
0.7721205186880244
1768 B66 P46 Control group Post-cholecystectomy
0.7456140350877193
1769 B66 P47 Control group Post-cholecystectomy
0.9460335621662853
1770 B66 P50 Control group Post-cholecystectomy
0.7608695652173914
1771 B66 P55 Control group Post-cholecystectomy
0.7623951182303585
1772 B66 P58 Control group Post-cholecystectomy
0.7185354691075515
1773 B66 P60 Control group Post-cholecystectomy
0.9300152555301296
1774 B66 P63 Control group Post-cholecystectomy
0.7602974828375286
1775 B66 P65 Control group Post-cholecystectomy
0.6794431731502669
1776 B66 P68 Control group Post-cholecystectomy
0.772883295194508
1777 B66 P70 Control group Post-cholecystectomy
0.8047292143401983
1778 B66 P71 Control group Post-cholecystectomy
0.8613653699466056
1779 B66 P74 Control group Post-cholecystectomy
0.7896643783371472
1780 B66 P75 Control group Post-cholecystectomy
0.8115942028985508
1781 B86 P1 Control group Post-cholecystectomy
0.9506102212051869
1782 B86 P2 Control group Post-cholecystectomy

0.7856598016781083
1783 B86 P4 Control group Post-cholecystectomy
0.8125476735316552
1784 B86 P5 Control group Post-cholecystectomy
0.7725019069412662
1785 B86 P9 Control group Post-cholecystectomy
0.8688024408848207
1786 B86 P13 Control group Post-cholecystectomy
0.8152173913043478
1787 B86 P15 Control group Post-cholecystectomy
0.7198703279938978
1788 B86 P16 Control group Post-cholecystectomy
0.8253241800152555
1789 B86 P17 Control group Post-cholecystectomy
0.7751716247139588
1790 B86 P20 Control group Post-cholecystectomy
0.8140732265446224
1791 B86 P21 Control group Post-cholecystectomy
0.8363844393592678
1792 B86 P24 Control group Post-cholecystectomy
0.8478260869565217
1793 B86 P26 Control group Post-cholecystectomy
0.8415331807780321
1794 B86 P30 Control group Post-cholecystectomy
0.9145690312738368
1795 B86 P33 Control group Post-cholecystectomy
0.7648741418764302
1796 B86 P35 Control group Post-cholecystectomy
0.8104500381388253
1797 B86 P38 Control group Post-cholecystectomy
0.9334477498093059
1798 B86 P39 Control group Post-cholecystectomy
0.9187643020594966
1799 B86 P42 Control group Post-cholecystectomy
0.8676582761250954
1800 B86 P43 Control group Post-cholecystectomy
0.8335240274599542
1801 B86 P46 Control group Post-cholecystectomy
0.690884820747521
1802 B86 P47 Control group Post-cholecystectomy
0.6903127383676583
1803 B86 P50 Control group Post-cholecystectomy
0.7570556826849733
1804 B86 P55 Control group Post-cholecystectomy
0.7368421052631579
1805 B86 P58 Control group Post-cholecystectomy
0.8217009916094584
1806 B86 P60 Control group Post-cholecystectomy
0.9387871853546911
1807 B86 P63 Control group Post-cholecystectomy
0.784324942791762
1808 B86 P65 Control group Post-cholecystectomy
0.7187261632341724
1809 B86 P68 Control group Post-cholecystectomy

0.8697559115179252
1810 B86 P70 Control group Post-cholecystectomy
0.8281845919145691
1811 B86 P71 Control group Post-cholecystectomy
0.7236842105263158
1812 B86 P74 Control group Post-cholecystectomy
0.8026315789473685
1813 B86 P75 Control group Post-cholecystectomy
0.7829900839054157
1814 B97 P1 Control group Post-cholecystectomy
0.9424103737604882
1815 B97 P2 Control group Post-cholecystectomy
0.729023646071701
1816 B97 P4 Control group Post-cholecystectomy
0.7315026697177727
1817 B97 P5 Control group Post-cholecystectomy
0.6977498093058734
1818 B97 P9 Control group Post-cholecystectomy
0.8487795575896262
1819 B97 P13 Control group Post-cholecystectomy
0.8159801678108314
1820 B97 P15 Control group Post-cholecystectomy
0.7528604118993135
1821 B97 P16 Control group Post-cholecystectomy
0.6634248665141114
1822 B97 P17 Control group Post-cholecystectomy
0.7313119755911518
1823 B97 P20 Control group Post-cholecystectomy
0.8176964149504196
1824 B97 P21 Control group Post-cholecystectomy
0.8287566742944318
1825 B97 P24 Control group Post-cholecystectomy
0.7028985507246377
1826 B97 P26 Control group Post-cholecystectomy
0.8022501906941266
1827 B97 P30 Control group Post-cholecystectomy
0.8373379099923722
1828 B97 P33 Control group Post-cholecystectomy
0.7183447749809306
1829 B97 P35 Control group Post-cholecystectomy
0.7343630816170862
1830 B97 P38 Control group Post-cholecystectomy
0.9113272311212814
1831 B97 P39 Control group Post-cholecystectomy
0.8613653699466056
1832 B97 P42 Control group Post-cholecystectomy
0.8232265446224256
1833 B97 P43 Control group Post-cholecystectomy
0.8079710144927537
1834 B97 P46 Control group Post-cholecystectomy
0.7204424103737604
1835 B97 P47 Control group Post-cholecystectomy
0.9113272311212814
1836 B97 P50 Control group Post-cholecystectomy

0.708047292143402
1837	B97	P55	Control group	Post-cholecystectomy
0.6544622425629291
1838	B97	P58	Control group	Post-cholecystectomy
0.7492372234935164
1839	B97	P60	Control group	Post-cholecystectomy
0.8533562166285278
1840	B97	P63	Control group	Post-cholecystectomy
0.6500762776506483
1841	B97	P65	Control group	Post-cholecystectomy
0.7580091533180778
1842	B97	P68	Control group	Post-cholecystectomy
0.7320747520976354
1843	B97	P70	Control group	Post-cholecystectomy
0.8607932875667429
1844	B97	P71	Control group	Post-cholecystectomy
0.8688024408848207
1845	B97	P74	Control group	Post-cholecystectomy
0.7166285278413425
1846	B97	P75	Control group	Post-cholecystectomy
0.732837528604119
1847	B98	P1	Control group	Post-cholecystectomy
0.9492753623188406
1848	B98	P2	Control group	Post-cholecystectomy
0.8049199084668193
1849	B98	P4	Control group	Post-cholecystectomy
0.6876430205949656
1850	B98	P5	Control group	Post-cholecystectomy
0.698512585812357
1851	B98	P9	Control group	Post-cholecystectomy
0.7707856598016781
1852	B98	P13	Control group	Post-cholecystectomy
0.7988176964149504
1853	B98	P15	Control group	Post-cholecystectomy
0.7048054919908466
1854	B98	P16	Control group	Post-cholecystectomy
0.8001525553012967
1855	B98	P17	Control group	Post-cholecystectomy
0.7759344012204424
1856	B98	P20	Control group	Post-cholecystectomy
0.7374141876430206
1857	B98	P21	Control group	Post-cholecystectomy
0.8257055682684973
1858	B98	P24	Control group	Post-cholecystectomy
0.7988176964149504
1859	B98	P26	Control group	Post-cholecystectomy
0.8670861937452327
1860	B98	P30	Control group	Post-cholecystectomy
0.8085430968726163
1861	B98	P33	Control group	Post-cholecystectomy
0.8003432494279176
1862	B98	P35	Control group	Post-cholecystectomy
0.8274218154080855
1863	B98	P38	Control group	Post-cholecystectomy

0.938977879481312
1864 B98 P39 Control group Post-cholecystectomy
0.8459191456903128
1865 B98 P42 Control group Post-cholecystectomy
0.8857742181540809
1866 B98 P43 Control group Post-cholecystectomy
0.7835621662852784
1867 B98 P46 Control group Post-cholecystectomy
0.7759344012204424
1868 B98 P47 Control group Post-cholecystectomy
0.9233409610983981
1869 B98 P50 Control group Post-cholecystectomy
0.7280701754385965
1870 B98 P55 Control group Post-cholecystectomy
0.7036613272311213
1871 B98 P58 Control group Post-cholecystectomy
0.7772692601067888
1872 B98 P60 Control group Post-cholecystectomy
0.8312356979405034
1873 B98 P63 Control group Post-cholecystectomy
0.7736460717009916
1874 B98 P65 Control group Post-cholecystectomy
0.8373379099923722
1875 B98 P68 Control group Post-cholecystectomy
0.8546910755148741
1876 B98 P70 Control group Post-cholecystectomy
0.8928299008390541
1877 B98 P71 Control group Post-cholecystectomy
0.8226544622425629
1878 B98 P74 Control group Post-cholecystectomy
0.8043478260869565
1879 B98 P75 Control group Post-cholecystectomy
0.7229214340198322
1880 B100 P1 Control group Post-cholecystectomy
0.9345919145690312
1881 B100 P2 Control group Post-cholecystectomy
0.9269641495041953
1882 B100 P4 Control group Post-cholecystectomy
0.9256292906178489
1883 B100 P5 Control group Post-cholecystectomy
0.9433638443935927
1884 B100 P9 Control group Post-cholecystectomy
0.8848207475209764
1885 B100 P13 Control group Post-cholecystectomy
0.9549961861174676
1886 B100 P15 Control group Post-cholecystectomy
0.9157131960335622
1887 B100 P16 Control group Post-cholecystectomy
0.8609839816933639
1888 B100 P17 Control group Post-cholecystectomy
0.9290617848970252
1889 B100 P20 Control group Post-cholecystectomy
0.9382151029748284
1890 B100 P21 Control group Post-cholecystectomy

0.9052250190694127
1891	B100	P24	Control group	Post-cholecystectomy
0.9508009153318078
1892	B100	P26	Control group	Post-cholecystectomy
0.8913043478260869
1893	B100	P30	Control group	Post-cholecystectomy
0.7734553775743707
1894	B100	P33	Control group	Post-cholecystectomy
0.9040808543096872
1895	B100	P35	Control group	Post-cholecystectomy
0.8985507246376812
1896	B100	P38	Control group	Post-cholecystectomy
0.21929824561403508
1897	B100	P39	Control group	Post-cholecystectomy
0.9210526315789473
1898	B100	P42	Control group	Post-cholecystectomy
0.9187643020594966
1899	B100	P43	Control group	Post-cholecystectomy
0.956140350877193
1900	B100	P46	Control group	Post-cholecystectomy
0.9496567505720824
1901	B100	P47	Control group	Post-cholecystectomy
0.9553775743707094
1902	B100	P50	Control group	Post-cholecystectomy
0.92372234935164
1903	B100	P55	Control group	Post-cholecystectomy
0.9635774218154081
1904	B100	P58	Control group	Post-cholecystectomy
0.9498474446987033
1905	B100	P60	Control group	Post-cholecystectomy
0.9273455377574371
1906	B100	P63	Control group	Post-cholecystectomy
0.9393592677345538
1907	B100	P65	Control group	Post-cholecystectomy
0.9565217391304348
1908	B100	P68	Control group	Post-cholecystectomy
0.9487032799389779
1909	B100	P70	Control group	Post-cholecystectomy
0.9706331045003814
1910	B100	P71	Control group	Post-cholecystectomy
0.9641495041952708
1911	B100	P74	Control group	Post-cholecystectomy
0.956140350877193
1912	B100	P75	Control group	Post-cholecystectomy
0.9382151029748284
1913	B112	P1	Control group	Post-cholecystectomy
0.9658657513348589
1914	B112	P2	Control group	Post-cholecystectomy
0.8209382151029748
1915	B112	P4	Control group	Post-cholecystectomy
0.8834858886346301
1916	B112	P5	Control group	Post-cholecystectomy
0.8083524027459954
1917	B112	P9	Control group	Post-cholecystectomy

0.9124713958810069
1918 B112 P13 Control group Post-cholecystectomy
0.9080854309687262
1919 B112 P15 Control group Post-cholecystectomy
0.6424485125858124
1920 B112 P16 Control group Post-cholecystectomy
0.8888253241800153
1921 B112 P17 Control group Post-cholecystectomy
0.8922578184591915
1922 B112 P20 Control group Post-cholecystectomy
0.9033180778032036
1923 B112 P21 Control group Post-cholecystectomy
0.900839054157132
1924 B112 P24 Control group Post-cholecystectomy
0.8604118993135011
1925 B112 P26 Control group Post-cholecystectomy
0.9059877955758963
1926 B112 P30 Control group Post-cholecystectomy
0.9487032799389779
1927 B112 P33 Control group Post-cholecystectomy
0.8779557589626239
1928 B112 P35 Control group Post-cholecystectomy
0.9004576659038902
1929 B112 P38 Control group Post-cholecystectomy
0.9662471395881007
1930 B112 P39 Control group Post-cholecystectomy
0.9155225019069413
1931 B112 P42 Control group Post-cholecystectomy
0.9393592677345538
1932 B112 P43 Control group Post-cholecystectomy
0.8779557589626239
1933 B112 P46 Control group Post-cholecystectomy
0.7541952707856598
1934 B112 P47 Control group Post-cholecystectomy
0.7847063310450039
1935 B112 P50 Control group Post-cholecystectomy
0.8215102974828375
1936 B112 P55 Control group Post-cholecystectomy
0.8197940503432495
1937 B112 P58 Control group Post-cholecystectomy
0.8934019832189168
1938 B112 P60 Control group Post-cholecystectomy
0.9855072463768116
1939 B112 P63 Control group Post-cholecystectomy
0.8794813119755912
1940 B112 P65 Control group Post-cholecystectomy
0.831998474446987
1941 B112 P68 Control group Post-cholecystectomy
0.8712814645308925
1942 B112 P70 Control group Post-cholecystectomy
0.8852021357742181
1943 B112 P71 Control group Post-cholecystectomy
0.6817315026697178
1944 B112 P74 Control group Post-cholecystectomy

0.849160945842868
1945 B112 P75 Control group Post-cholecystectomy
0.8426773455377574
1946 B115 P1 Control group Post-cholecystectomy
0.9244851258581236
1947 B115 P2 Control group Post-cholecystectomy
0.7841342486651411
1948 B115 P4 Control group Post-cholecystectomy
0.7587719298245614
1949 B115 P5 Control group Post-cholecystectomy
0.6931731502669718
1950 B115 P9 Control group Post-cholecystectomy
0.6657131960335622
1951 B115 P13 Control group Post-cholecystectomy
0.7349351639969489
1952 B115 P15 Control group Post-cholecystectomy
0.816742944317315
1953 B115 P16 Control group Post-cholecystectomy
0.7887109077040427
1954 B115 P17 Control group Post-cholecystectomy
0.7915713196033562
1955 B115 P20 Control group Post-cholecystectomy
0.8020594965675057
1956 B115 P21 Control group Post-cholecystectomy
0.7610602593440122
1957 B115 P24 Control group Post-cholecystectomy
0.7311212814645309
1958 B115 P26 Control group Post-cholecystectomy
0.8829138062547673
1959 B115 P30 Control group Post-cholecystectomy
0.8005339435545386
1960 B115 P33 Control group Post-cholecystectomy
0.8026315789473685
1961 B115 P35 Control group Post-cholecystectomy
0.7782227307398932
1962 B115 P38 Control group Post-cholecystectomy
0.9429824561403509
1963 B115 P39 Control group Post-cholecystectomy
0.8728070175438597
1964 B115 P42 Control group Post-cholecystectomy
0.8518306636155606
1965 B115 P43 Control group Post-cholecystectomy
0.8234172387490465
1966 B115 P46 Control group Post-cholecystectomy
0.8411517925247902
1967 B115 P47 Control group Post-cholecystectomy
0.9324942791762014
1968 B115 P50 Control group Post-cholecystectomy
0.7397025171624714
1969 B115 P55 Control group Post-cholecystectomy
0.6512204424103738
1970 B115 P58 Control group Post-cholecystectomy
0.8218916857360793
1971 B115 P60 Control group Post-cholecystectomy

0.8903508771929824
1972 B115 P63 Control group Post-cholecystectomy
0.7930968726163234
1973 B115 P65 Control group Post-cholecystectomy
0.7902364607170099
1974 B115 P68 Control group Post-cholecystectomy
0.809115179252479
1975 B115 P70 Control group Post-cholecystectomy
0.8674675819984744
1976 B115 P71 Control group Post-cholecystectomy
0.8424866514111365
1977 B115 P74 Control group Post-cholecystectomy
0.8331426392067124
1978 B115 P75 Control group Post-cholecystectomy
0.6935545385202135
1979 I1 P1 Control group Post-cholecystectomy
0.944698703279939
1980 I1 P2 Control group Post-cholecystectomy
0.7170099160945843
1981 I1 P4 Control group Post-cholecystectomy
0.7431350114416476
1982 I1 P5 Control group Post-cholecystectomy
0.6674294431731502
1983 I1 P9 Control group Post-cholecystectomy
0.7799389778794813
1984 I1 P13 Control group Post-cholecystectomy
0.8075896262395118
1985 I1 P15 Control group Post-cholecystectomy
0.7484744469870328
1986 I1 P16 Control group Post-cholecystectomy
0.8388634630053394
1987 I1 P17 Control group Post-cholecystectomy
0.8155987795575896
1988 I1 P20 Control group Post-cholecystectomy
0.7936689549961862
1989 I1 P21 Control group Post-cholecystectomy
0.8453470633104501
1990 I1 P24 Control group Post-cholecystectomy
0.7574370709382151
1991 I1 P26 Control group Post-cholecystectomy
0.8632723112128147
1992 I1 P30 Control group Post-cholecystectomy
0.7868039664378337
1993 I1 P33 Control group Post-cholecystectomy
0.814836003051106
1994 I1 P35 Control group Post-cholecystectomy
0.84744469870328
1995 I1 P38 Control group Post-cholecystectomy
0.9052250190694127
1996 I1 P39 Control group Post-cholecystectomy
0.8922578184591915
1997 I1 P42 Control group Post-cholecystectomy
0.9324942791762014
1998 I1 P43 Control group Post-cholecystectomy

0.7631578947368421
1999 I1 P46 Control group Post-cholecystectomy
0.8283752860411899
2000 I1 P47 Control group Post-cholecystectomy
0.937070938215103
2001 I1 P50 Control group Post-cholecystectomy
0.7318840579710145
2002 I1 P55 Control group Post-cholecystectomy
0.650839054157132
2003 I1 P58 Control group Post-cholecystectomy
0.7749809305873379
2004 I1 P60 Control group Post-cholecystectomy
0.8688024408848207
2005 I1 P63 Control group Post-cholecystectomy
0.7438977879481312
2006 I1 P65 Control group Post-cholecystectomy
0.7810831426392068
2007 I1 P68 Control group Post-cholecystectomy
0.8436308161708619
2008 I1 P70 Control group Post-cholecystectomy
0.879862700228833
2009 I1 P71 Control group Post-cholecystectomy
0.8754767353165522
2010 I1 P74 Control group Post-cholecystectomy
0.799580472921434
2011 I1 P75 Control group Post-cholecystectomy
0.6981311975591151
2012 I3 P1 Control group Post-cholecystectomy
0.9128527841342486
2013 I3 P2 Control group Post-cholecystectomy
0.7913806254767353
2014 I3 P4 Control group Post-cholecystectomy
0.7562929061784897
2015 I3 P5 Control group Post-cholecystectomy
0.725209763539283
2016 I3 P9 Control group Post-cholecystectomy
0.8318077803203662
2017 I3 P13 Control group Post-cholecystectomy
0.7488558352402745
2018 I3 P15 Control group Post-cholecystectomy
0.7337909992372235
2019 I3 P16 Control group Post-cholecystectomy
0.7090007627765065
2020 I3 P17 Control group Post-cholecystectomy
0.8136918382913806
2021 I3 P20 Control group Post-cholecystectomy
0.6485507246376812
2022 I3 P21 Control group Post-cholecystectomy
0.7130053394355453
2023 I3 P24 Control group Post-cholecystectomy
0.7906178489702517
2024 I3 P26 Control group Post-cholecystectomy
0.8138825324180016
2025 I3 P30 Control group Post-cholecystectomy

0.9376430205949656
2026	I3	P33	Control group	Post-cholecystectomy
0.6786803966437833
2027	I3	P35	Control group	Post-cholecystectomy
0.8531655225019069
2028	I3	P38	Control group	Post-cholecystectomy
0.936880244088482
2029	I3	P39	Control group	Post-cholecystectomy
0.7387490465293669
2030	I3	P42	Control group	Post-cholecystectomy
0.9597635392829901
2031	I3	P43	Control group	Post-cholecystectomy
0.6224256292906178
2032	I3	P46	Control group	Post-cholecystectomy
0.738367658276125
2033	I3	P47	Control group	Post-cholecystectomy
0.9138062547673532
2034	I3	P50	Control group	Post-cholecystectomy
0.6948893974065599
2035	I3	P55	Control group	Post-cholecystectomy
0.7540045766590389
2036	I3	P58	Control group	Post-cholecystectomy
0.791952707856598
2037	I3	P60	Control group	Post-cholecystectomy
0.7191075514874142
2038	I3	P63	Control group	Post-cholecystectomy
0.8165522501906941
2039	I3	P65	Control group	Post-cholecystectomy
0.7887109077040427
2040	I3	P68	Control group	Post-cholecystectomy
0.8424866514111365
2041	I3	P70	Control group	Post-cholecystectomy
0.877765064836003
2042	I3	P71	Control group	Post-cholecystectomy
0.8611746758199847
2043	I3	P74	Control group	Post-cholecystectomy
0.7336003051106026
2044	I3	P75	Control group	Post-cholecystectomy
0.6905034324942791
2045	I6	P1	Control group	Post-cholecystectomy
0.9548054919908466
2046	I6	P2	Control group	Post-cholecystectomy
0.738367658276125
2047	I6	P4	Control group	Post-cholecystectomy
0.8188405797101449
2048	I6	P5	Control group	Post-cholecystectomy
0.6584668192219679
2049	I6	P9	Control group	Post-cholecystectomy
0.6931731502669718
2050	I6	P13	Control group	Post-cholecystectomy
0.8363844393592678
2051	I6	P15	Control group	Post-cholecystectomy
0.8096872616323417
2052	I6	P16	Control group	Post-cholecystectomy

0.8056826849733029
2053	I6	P17	Control group	Post-cholecystectomy
0.7801296720061022
2054	I6	P20	Control group	Post-cholecystectomy
0.7738367658276125
2055	I6	P21	Control group	Post-cholecystectomy
0.709954233409611
2056	I6	P24	Control group	Post-cholecystectomy
0.7795575896262396
2057	I6	P26	Control group	Post-cholecystectomy
0.7808924485125858
2058	I6	P30	Control group	Post-cholecystectomy
0.841723874904653
2059	I6	P33	Control group	Post-cholecystectomy
0.7660183066361556
2060	I6	P35	Control group	Post-cholecystectomy
0.7559115179252479
2061	I6	P38	Control group	Post-cholecystectomy
0.9488939740655988
2062	I6	P39	Control group	Post-cholecystectomy
0.8035850495804729
2063	I6	P42	Control group	Post-cholecystectomy
0.8297101449275363
2064	I6	P43	Control group	Post-cholecystectomy
0.8047292143401983
2065	I6	P46	Control group	Post-cholecystectomy
0.8106407322654462
2066	I6	P47	Control group	Post-cholecystectomy
0.9248665141113653
2067	I6	P50	Control group	Post-cholecystectomy
0.7131960335621663
2068	I6	P55	Control group	Post-cholecystectomy
0.6340579710144928
2069	I6	P58	Control group	Post-cholecystectomy
0.6241418764302059
2070	I6	P60	Control group	Post-cholecystectomy
0.881769641495042
2071	I6	P63	Control group	Post-cholecystectomy
0.7858504958047292
2072	I6	P65	Control group	Post-cholecystectomy
0.7141495041952708
2073	I6	P68	Control group	Post-cholecystectomy
0.7015636918382914
2074	I6	P70	Control group	Post-cholecystectomy
0.7913806254767353
2075	I6	P71	Control group	Post-cholecystectomy
0.8943554538520213
2076	I6	P74	Control group	Post-cholecystectomy
0.7902364607170099
2077	I6	P75	Control group	Post-cholecystectomy
0.7200610221205187
2078	I8	P1	Control group	Post-cholecystectomy
0.9454614797864226
2079	I8	P2	Control group	Post-cholecystectomy

0.7318840579710145
2080 I8 P4 Control group Post-cholecystectomy
0.8836765827612509
2081 I8 P5 Control group Post-cholecystectomy
0.7665903890160183
2082 I8 P9 Control group Post-cholecystectomy
0.9052250190694127
2083 I8 P13 Control group Post-cholecystectomy
0.7055682684973302
2084 I8 P15 Control group Post-cholecystectomy
0.8381006864988558
2085 I8 P16 Control group Post-cholecystectomy
0.8838672768878718
2086 I8 P17 Control group Post-cholecystectomy
0.8323798627002288
2087 I8 P20 Control group Post-cholecystectomy
0.8617467581998475
2088 I8 P21 Control group Post-cholecystectomy
0.8920671243325705
2089 I8 P24 Control group Post-cholecystectomy
0.7679252479023646
2090 I8 P26 Control group Post-cholecystectomy
0.9271548436308161
2091 I8 P30 Control group Post-cholecystectomy
0.9098016781083142
2092 I8 P33 Control group Post-cholecystectomy
0.8653699466056446
2093 I8 P35 Control group Post-cholecystectomy
0.8632723112128147
2094 I8 P38 Control group Post-cholecystectomy
0.9435545385202135
2095 I8 P39 Control group Post-cholecystectomy
0.9189549961861174
2096 I8 P42 Control group Post-cholecystectomy
0.9515636918382914
2097 I8 P43 Control group Post-cholecystectomy
0.8381006864988558
2098 I8 P46 Control group Post-cholecystectomy
0.8773836765827613
2099 I8 P47 Control group Post-cholecystectomy
0.9405034324942791
2100 I8 P50 Control group Post-cholecystectomy
0.8136918382913806
2101 I8 P55 Control group Post-cholecystectomy
0.704042715484363
2102 I8 P58 Control group Post-cholecystectomy
0.897025171624714
2103 I8 P60 Control group Post-cholecystectomy
0.9776887871853547
2104 I8 P63 Control group Post-cholecystectomy
0.812929061784897
2105 I8 P65 Control group Post-cholecystectomy
0.8485888634630053
2106 I8 P68 Control group Post-cholecystectomy

0.8699466056445462
2107 I8 P70 Control group Post-cholecystectomy
0.8962623951182304
2108 I8 P71 Control group Post-cholecystectomy
0.9006483600305111
2109 I8 P74 Control group Post-cholecystectomy
0.8611746758199847
2110 I8 P75 Control group Post-cholecystectomy
0.7765064836003052
2111 I10 P1 Control group Post-cholecystectomy
0.9450800915331807
2112 I10 P2 Control group Post-cholecystectomy
0.7862318840579711
2113 I10 P4 Control group Post-cholecystectomy
0.8047292143401983
2114 I10 P5 Control group Post-cholecystectomy
0.7940503432494279
2115 I10 P9 Control group Post-cholecystectomy
0.8729977116704806
2116 I10 P13 Control group Post-cholecystectomy
0.6125095347063311
2117 I10 P15 Control group Post-cholecystectomy
0.8331426392067124
2118 I10 P16 Control group Post-cholecystectomy
0.8413424866514111
2119 I10 P17 Control group Post-cholecystectomy
0.8077803203661327
2120 I10 P20 Control group Post-cholecystectomy
0.7744088482074752
2121 I10 P21 Control group Post-cholecystectomy
0.7837528604118993
2122 I10 P24 Control group Post-cholecystectomy
0.8218916857360793
2123 I10 P26 Control group Post-cholecystectomy
0.8211289092295957
2124 I10 P30 Control group Post-cholecystectomy
0.9340198321891686
2125 I10 P33 Control group Post-cholecystectomy
0.736651411136537
2126 I10 P35 Control group Post-cholecystectomy
0.7929061784897025
2127 I10 P38 Control group Post-cholecystectomy
0.9538520213577422
2128 I10 P39 Control group Post-cholecystectomy
0.8975972540045767
2129 I10 P42 Control group Post-cholecystectomy
0.9359267734553776
2130 I10 P43 Control group Post-cholecystectomy
0.7915713196033562
2131 I10 P46 Control group Post-cholecystectomy
0.7742181540808543
2132 I10 P47 Control group Post-cholecystectomy
0.948512585812357
2133 I10 P50 Control group Post-cholecystectomy

0.7484744469870328
2134	I10	P55	Control group	Post-cholecystectomy
0.7110983981693364
2135	I10	P58	Control group	Post-cholecystectomy
0.8758581235697941
2136	I10	P60	Control group	Post-cholecystectomy
0.8920671243325705
2137	I10	P63	Control group	Post-cholecystectomy
0.782608695652174
2138	I10	P65	Control group	Post-cholecystectomy
0.780511060259344
2139	I10	P68	Control group	Post-cholecystectomy
0.8590770404271548
2140	I10	P70	Control group	Post-cholecystectomy
0.8012967200610221
2141	I10	P71	Control group	Post-cholecystectomy
0.8560259344012204
2142	I10	P74	Control group	Post-cholecystectomy
0.7419908466819222
2143	I10	P75	Control group	Post-cholecystectomy
0.7536231884057971
2144	I11	P1	Control group	Post-cholecystectomy
0.9366895499618612
2145	I11	P2	Control group	Post-cholecystectomy
0.8832951945080092
2146	I11	P4	Control group	Post-cholecystectomy
0.82627765064836
2147	I11	P5	Control group	Post-cholecystectomy
0.8623188405797102
2148	I11	P9	Control group	Post-cholecystectomy
0.8810068649885584
2149	I11	P13	Control group	Post-cholecystectomy
0.8878718535469108
2150	I11	P15	Control group	Post-cholecystectomy
0.8165522501906941
2151	I11	P16	Control group	Post-cholecystectomy
0.8619374523264683
2152	I11	P17	Control group	Post-cholecystectomy
0.8709000762776506
2153	I11	P20	Control group	Post-cholecystectomy
0.778604118993135
2154	I11	P21	Control group	Post-cholecystectomy
0.8922578184591915
2155	I11	P24	Control group	Post-cholecystectomy
0.8686117467581999
2156	I11	P26	Control group	Post-cholecystectomy
0.9795957284515637
2157	I11	P30	Control group	Post-cholecystectomy
0.8897787948131197
2158	I11	P33	Control group	Post-cholecystectomy
0.8762395118230358
2159	I11	P35	Control group	Post-cholecystectomy
0.9099923722349351
2160	I11	P38	Control group	Post-cholecystectomy

0.9191456903127384
2161	I11	P39	Control group	Post-cholecystectomy
0.8367658276125095
2162	I11	P42	Control group	Post-cholecystectomy
0.9235316552250191
2163	I11	P43	Control group	Post-cholecystectomy
0.8590770404271548
2164	I11	P46	Control group	Post-cholecystectomy
0.8607932875667429
2165	I11	P47	Control group	Post-cholecystectomy
0.9176201372997712
2166	I11	P50	Control group	Post-cholecystectomy
0.8861556064073226
2167	I11	P55	Control group	Post-cholecystectomy
0.8790999237223494
2168	I11	P58	Control group	Post-cholecystectomy
0.8897787948131197
2169	I11	P60	Control group	Post-cholecystectomy
0.8785278413424866
2170	I11	P63	Control group	Post-cholecystectomy
0.8878718535469108
2171	I11	P65	Control group	Post-cholecystectomy
0.8790999237223494
2172	I11	P68	Control group	Post-cholecystectomy
0.9042715484363082
2173	I11	P70	Control group	Post-cholecystectomy
0.9584286803966438
2174	I11	P71	Control group	Post-cholecystectomy
0.9689168573607932
2175	I11	P74	Control group	Post-cholecystectomy
0.9050343249427918
2176	I11	P75	Control group	Post-cholecystectomy
0.8382913806254767
2177	I13	P1	Control group	Post-cholecystectomy
0.9479405034324943
2178	I13	P2	Control group	Post-cholecystectomy
0.7463768115942029
2179	I13	P4	Control group	Post-cholecystectomy
0.8569794050343249
2180	I13	P5	Control group	Post-cholecystectomy
0.7177726926010679
2181	I13	P9	Control group	Post-cholecystectomy
0.8562166285278413
2182	I13	P13	Control group	Post-cholecystectomy
0.7032799389778794
2183	I13	P15	Control group	Post-cholecystectomy
0.818649885583524
2184	I13	P16	Control group	Post-cholecystectomy
0.830091533180778
2185	I13	P17	Control group	Post-cholecystectomy
0.7978642257818459
2186	I13	P20	Control group	Post-cholecystectomy
0.7765064836003052
2187	I13	P21	Control group	Post-cholecystectomy

0.8579328756674295
2188 I13 P24 Control group Post-cholecystectomy
0.7183447749809306
2189 I13 P26 Control group Post-cholecystectomy
0.7862318840579711
2190 I13 P30 Control group Post-cholecystectomy
0.9258199847444699
2191 I13 P33 Control group Post-cholecystectomy
0.8117848970251716
2192 I13 P35 Control group Post-cholecystectomy
0.7570556826849733
2193 I13 P38 Control group Post-cholecystectomy
0.959954233409611
2194 I13 P39 Control group Post-cholecystectomy
0.8594584286803967
2195 I13 P42 Control group Post-cholecystectomy
0.8792906178489702
2196 I13 P43 Control group Post-cholecystectomy
0.7049961861174676
2197 I13 P46 Control group Post-cholecystectomy
0.831998474446987
2198 I13 P47 Control group Post-cholecystectomy
0.8934019832189168
2199 I13 P50 Control group Post-cholecystectomy
0.8041571319603357
2200 I13 P55 Control group Post-cholecystectomy
0.7339816933638444
2201 I13 P58 Control group Post-cholecystectomy
0.8371472158657514
2202 I13 P60 Control group Post-cholecystectomy
0.9233409610983981
2203 I13 P63 Control group Post-cholecystectomy
0.8234172387490465
2204 I13 P65 Control group Post-cholecystectomy
0.8480167810831426
2205 I13 P68 Control group Post-cholecystectomy
0.8209382151029748
2206 I13 P70 Control group Post-cholecystectomy
0.8426773455377574
2207 I13 P71 Control group Post-cholecystectomy
0.8564073226544623
2208 I13 P74 Control group Post-cholecystectomy
0.6127002288329519
2209 I13 P75 Control group Post-cholecystectomy
0.7829900839054157
2210 I15 P1 Control group Post-cholecystectomy
0.8625095347063311
2211 I15 P2 Control group Post-cholecystectomy
0.8352402745995423
2212 I15 P4 Control group Post-cholecystectomy
0.8285659801678108
2213 I15 P5 Control group Post-cholecystectomy
0.8278032036613272
2214 I15 P9 Control group Post-cholecystectomy

0.8447749809305873
2215 I15 P13 Control group Post-cholecystectomy
0.8201754385964912
2216 I15 P15 Control group Post-cholecystectomy
0.8737604881769642
2217 I15 P16 Control group Post-cholecystectomy
0.698512585812357
2218 I15 P17 Control group Post-cholecystectomy
0.8829138062547673
2219 I15 P20 Control group Post-cholecystectomy
0.801487414187643
2220 I15 P21 Control group Post-cholecystectomy
0.7690694126620901
2221 I15 P24 Control group Post-cholecystectomy
0.9376430205949656
2222 I15 P26 Control group Post-cholecystectomy
0.6548436308161708
2223 I15 P30 Control group Post-cholecystectomy
0.9271548436308161
2224 I15 P33 Control group Post-cholecystectomy
0.6966056445461479
2225 I15 P35 Control group Post-cholecystectomy
0.7862318840579711
2226 I15 P38 Control group Post-cholecystectomy
0.9534706331045004
2227 I15 P39 Control group Post-cholecystectomy
0.8659420289855072
2228 I15 P42 Control group Post-cholecystectomy
0.7711670480549199
2229 I15 P43 Control group Post-cholecystectomy
0.7946224256292906
2230 I15 P46 Control group Post-cholecystectomy
0.8072082379862701
2231 I15 P47 Control group Post-cholecystectomy
0.9239130434782609
2232 I15 P50 Control group Post-cholecystectomy
0.7953852021357742
2233 I15 P55 Control group Post-cholecystectomy
0.7458047292143402
2234 I15 P58 Control group Post-cholecystectomy
0.7114797864225781
2235 I15 P60 Control group Post-cholecystectomy
0.9426010678871091
2236 I15 P63 Control group Post-cholecystectomy
0.772883295194508
2237 I15 P65 Control group Post-cholecystectomy
0.797673531655225
2238 I15 P68 Control group Post-cholecystectomy
0.7412280701754386
2239 I15 P70 Control group Post-cholecystectomy
0.7803203661327232
2240 I15 P71 Control group Post-cholecystectomy
0.8781464530892449
2241 I15 P74 Control group Post-cholecystectomy

0.721205186880244
2242 I15 P75 Control group Post-cholecystectomy
0.856788710907704
2243 I17 P1 Control group Post-cholecystectomy
0.9750190694126621
2244 I17 P2 Control group Post-cholecystectomy
0.7768878718535469
2245 I17 P4 Control group Post-cholecystectomy
0.8482074752097636
2246 I17 P5 Control group Post-cholecystectomy
0.7667810831426392
2247 I17 P9 Control group Post-cholecystectomy
0.9374523264683448
2248 I17 P13 Control group Post-cholecystectomy
0.7755530129672006
2249 I17 P15 Control group Post-cholecystectomy
0.7377955758962624
2250 I17 P16 Control group Post-cholecystectomy
0.9012204424103738
2251 I17 P17 Control group Post-cholecystectomy
0.8421052631578947
2252 I17 P20 Control group Post-cholecystectomy
0.8428680396643783
2253 I17 P21 Control group Post-cholecystectomy
0.9138062547673532
2254 I17 P24 Control group Post-cholecystectomy
0.7965293668954996
2255 I17 P26 Control group Post-cholecystectomy
0.937070938215103
2256 I17 P30 Control group Post-cholecystectomy
0.9195270785659801
2257 I17 P33 Control group Post-cholecystectomy
0.8463005339435545
2258 I17 P35 Control group Post-cholecystectomy
0.8895881006864989
2259 I17 P38 Control group Post-cholecystectomy
0.9601449275362319
2260 I17 P39 Control group Post-cholecystectomy
0.9111365369946606
2261 I17 P42 Control group Post-cholecystectomy
0.9881769641495042
2262 I17 P43 Control group Post-cholecystectomy
0.8487795575896262
2263 I17 P46 Control group Post-cholecystectomy
0.7641113653699466
2264 I17 P47 Control group Post-cholecystectomy
0.7088100686498856
2265 I17 P50 Control group Post-cholecystectomy
0.8293287566742944
2266 I17 P55 Control group Post-cholecystectomy
0.8018688024408849
2267 I17 P58 Control group Post-cholecystectomy
0.9162852784134249
2268 I17 P60 Control group Post-cholecystectomy

0.9498474446987033
2269 I17 P63 Control group Post-cholecystectomy
0.8449656750572082
2270 I17 P65 Control group Post-cholecystectomy
0.9117086193745233
2271 I17 P68 Control group Post-cholecystectomy
0.9130434782608695
2272 I17 P70 Control group Post-cholecystectomy
0.9269641495041953
2273 I17 P71 Control group Post-cholecystectomy
0.7438977879481312
2274 I17 P74 Control group Post-cholecystectomy
0.8657513348588863
2275 I17 P75 Control group Post-cholecystectomy
0.8175057208237986
2276 I18 P1 Control group Post-cholecystectomy
0.8184591914569032
2277 I18 P2 Control group Post-cholecystectomy
0.7330282227307399
2278 I18 P4 Control group Post-cholecystectomy
0.799771167048055
2279 I18 P5 Control group Post-cholecystectomy
0.7297864225781846
2280 I18 P9 Control group Post-cholecystectomy
0.8030129672006102
2281 I18 P13 Control group Post-cholecystectomy
0.7282608695652174
2282 I18 P15 Control group Post-cholecystectomy
0.7967200610221206
2283 I18 P16 Control group Post-cholecystectomy
0.8098779557589626
2284 I18 P17 Control group Post-cholecystectomy
0.7660183066361556
2285 I18 P20 Control group Post-cholecystectomy
0.6409229595728452
2286 I18 P21 Control group Post-cholecystectomy
0.7601067887109078
2287 I18 P24 Control group Post-cholecystectomy
0.7070938215102975
2288 I18 P26 Control group Post-cholecystectomy
0.8811975591151793
2289 I18 P30 Control group Post-cholecystectomy
0.9223874904652937
2290 I18 P33 Control group Post-cholecystectomy
0.7425629290617849
2291 I18 P35 Control group Post-cholecystectomy
0.8201754385964912
2292 I18 P38 Control group Post-cholecystectomy
0.948512585812357
2293 I18 P39 Control group Post-cholecystectomy
0.8314263920671243
2294 I18 P42 Control group Post-cholecystectomy
0.8689931350114416
2295 I18 P43 Control group Post-cholecystectomy

0.7683066361556065
2296 I18 P46 Control group Post−cholecystectomy
0.7650648360030511
2297 I18 P47 Control group Post−cholecystectomy
0.8861556064073226
2298 I18 P50 Control group Post−cholecystectomy
0.7196796338672768
2299 I18 P55 Control group Post−cholecystectomy
0.729023646071701
2300 I18 P58 Control group Post−cholecystectomy
0.8064454614797865
2301 I18 P60 Control group Post−cholecystectomy
0.8506864988558352
2302 I18 P63 Control group Post−cholecystectomy
0.7848970251716247
2303 I18 P65 Control group Post−cholecystectomy
0.8268497330282227
2304 I18 P68 Control group Post−cholecystectomy
0.797673531655225
2305 I18 P70 Control group Post−cholecystectomy
0.8321891685736079
2306 I18 P71 Control group Post−cholecystectomy
0.7482837528604119
2307 I18 P74 Control group Post−cholecystectomy
0.665903890160183
2308 I18 P75 Control group Post−cholecystectomy
0.5875286041189931
2309 I19 P1 Control group Post−cholecystectomy
0.9273455377574371
2310 I19 P2 Control group Post−cholecystectomy
0.791952707856598
2311 I19 P4 Control group Post−cholecystectomy
0.9496567505720824
2312 I19 P5 Control group Post−cholecystectomy
0.8154080854309688
2313 I19 P9 Control group Post−cholecystectomy
0.9347826086956522
2314 I19 P13 Control group Post−cholecystectomy
0.8577421815408085
2315 I19 P15 Control group Post−cholecystectomy
0.8150266971777269
2316 I19 P16 Control group Post−cholecystectomy
0.9221967963386728
2317 I19 P17 Control group Post−cholecystectomy
0.8972158657513348
2318 I19 P20 Control group Post−cholecystectomy
0.9342105263157895
2319 I19 P21 Control group Post−cholecystectomy
0.9149504195270786
2320 I19 P24 Control group Post−cholecystectomy
0.8003432494279176
2321 I19 P26 Control group Post−cholecystectomy
0.9416475972540046
2322 I19 P30 Control group Post−cholecystectomy

0.9565217391304348
2323 I19 P33 Control group Post-cholecystectomy
0.8960717009916095
2324 I19 P35 Control group Post-cholecystectomy
0.9101830663615561
2325 I19 P38 Control group Post-cholecystectomy
0.9549961861174676
2326 I19 P39 Control group Post-cholecystectomy
0.9704424103737604
2327 I19 P42 Control group Post-cholecystectomy
0.9456521739130435
2328 I19 P43 Control group Post-cholecystectomy
0.9191456903127384
2329 I19 P46 Control group Post-cholecystectomy
0.8012967200610221
2330 I19 P47 Control group Post-cholecystectomy
0.7831807780320366
2331 I19 P50 Control group Post-cholecystectomy
0.8480167810831426
2332 I19 P55 Control group Post-cholecystectomy
0.8832951945080092
2333 I19 P58 Control group Post-cholecystectomy
0.9040808543096872
2334 I19 P60 Control group Post-cholecystectomy
0.9837909992372235
2335 I19 P63 Control group Post-cholecystectomy
0.9143783371472158
2336 I19 P65 Control group Post-cholecystectomy
0.8878718535469108
2337 I19 P68 Control group Post-cholecystectomy
0.9063691838291381
2338 I19 P70 Control group Post-cholecystectomy
0.8995041952707856
2339 I19 P71 Control group Post-cholecystectomy
0.7044241037376049
2340 I19 P74 Control group Post-cholecystectomy
0.8947368421052632
2341 I19 P75 Control group Post-cholecystectomy
0.8461098398169337
2342 I22 P1 Control group Post-cholecystectomy
0.8367658276125095
2343 I22 P2 Control group Post-cholecystectomy
0.7337909992372235
2344 I22 P4 Control group Post-cholecystectomy
0.8579328756674295
2345 I22 P5 Control group Post-cholecystectomy
0.7807017543859649
2346 I22 P9 Control group Post-cholecystectomy
0.8623188405797102
2347 I22 P13 Control group Post-cholecystectomy
0.7848970251716247
2348 I22 P15 Control group Post-cholecystectomy
0.7662090007627765
2349 I22 P16 Control group Post-cholecystectomy

0.8649885583524027
2350 I22 P17 Control group Post-cholecystectomy
0.7829900839054157
2351 I22 P20 Control group Post-cholecystectomy
0.7498093058733791
2352 I22 P21 Control group Post-cholecystectomy
0.8209382151029748
2353 I22 P24 Control group Post-cholecystectomy
0.78813882532418
2354 I22 P26 Control group Post-cholecystectomy
0.8977879481311976
2355 I22 P30 Control group Post-cholecystectomy
0.8937833714721587
2356 I22 P33 Control group Post-cholecystectomy
0.8045385202135774
2357 I22 P35 Control group Post-cholecystectomy
0.8565980167810832
2358 I22 P38 Control group Post-cholecystectomy
0.9429824561403509
2359 I22 P39 Control group Post-cholecystectomy
0.8598398169336384
2360 I22 P42 Control group Post-cholecystectomy
0.9269641495041953
2361 I22 P43 Control group Post-cholecystectomy
0.7879481311975591
2362 I22 P46 Control group Post-cholecystectomy
0.8346681922196796
2363 I22 P47 Control group Post-cholecystectomy
0.9193363844393593
2364 I22 P50 Control group Post-cholecystectomy
0.8022501906941266
2365 I22 P55 Control group Post-cholecystectomy
0.782418001525553
2366 I22 P58 Control group Post-cholecystectomy
0.8518306636155606
2367 I22 P60 Control group Post-cholecystectomy
0.9128527841342486
2368 I22 P63 Control group Post-cholecystectomy
0.8020594965675057
2369 I22 P65 Control group Post-cholecystectomy
0.8323798627002288
2370 I22 P68 Control group Post-cholecystectomy
0.8394355453852022
2371 I22 P70 Control group Post-cholecystectomy
0.8789092295957285
2372 I22 P71 Control group Post-cholecystectomy
0.8882532418001525
2373 I22 P74 Control group Post-cholecystectomy
0.8089244851258581
2374 I22 P75 Control group Post-cholecystectomy
0.7753623188405797
2375 I23 P1 Control group Post-cholecystectomy
0.9193363844393593
2376 I23 P2 Control group Post-cholecystectomy

0.6912662090007627
2377	I23	P4	Control group	Post-cholecystectomy
0.7351258581235698
2378	I23	P5	Control group	Post-cholecystectomy
0.652745995423341
2379	I23	P9	Control group	Post-cholecystectomy
0.7986270022883295
2380	I23	P13	Control group	Post-cholecystectomy
0.7435163996948894
2381	I23	P15	Control group	Post-cholecystectomy
0.7225400457665904
2382	I23	P16	Control group	Post-cholecystectomy
0.8089244851258581
2383	I23	P17	Control group	Post-cholecystectomy
0.6529366895499619
2384	I23	P20	Control group	Post-cholecystectomy
0.7831807780320366
2385	I23	P21	Control group	Post-cholecystectomy
0.7452326468344775
2386	I23	P24	Control group	Post-cholecystectomy
0.7421815408085431
2387	I23	P26	Control group	Post-cholecystectomy
0.7974828375286042
2388	I23	P30	Control group	Post-cholecystectomy
0.8653699466056446
2389	I23	P33	Control group	Post-cholecystectomy
0.6922196796338673
2390	I23	P35	Control group	Post-cholecystectomy
0.7400839054157132
2391	I23	P38	Control group	Post-cholecystectomy
0.9250572082379863
2392	I23	P39	Control group	Post-cholecystectomy
0.8659420289855072
2393	I23	P42	Control group	Post-cholecystectomy
0.8920671243325705
2394	I23	P43	Control group	Post-cholecystectomy
0.7534324942791762
2395	I23	P46	Control group	Post-cholecystectomy
0.7684973302822273
2396	I23	P47	Control group	Post-cholecystectomy
0.915903890160183
2397	I23	P50	Control group	Post-cholecystectomy
0.6447368421052632
2398	I23	P55	Control group	Post-cholecystectomy
0.7093821510297483
2399	I23	P58	Control group	Post-cholecystectomy
0.7839435545385202
2400	I23	P60	Control group	Post-cholecystectomy
0.8499237223493517
2401	I23	P63	Control group	Post-cholecystectomy
0.7713577421815409
2402	I23	P65	Control group	Post-cholecystectomy
0.7568649885583524
2403	I23	P68	Control group	Post-cholecystectomy

0.8579328756674295
2404 I23 P70 Control group Post-cholecystectomy
0.8543096872616324
2405 I23 P71 Control group Post-cholecystectomy
0.7875667429443173
2406 I23 P74 Control group Post-cholecystectomy
0.6887871853546911
2407 I23 P75 Control group Post-cholecystectomy
0.7139588100686499
2408 I24 P1 Control group Post-cholecystectomy
0.9774980930587338
2409 I24 P2 Control group Post-cholecystectomy
0.8060640732265446
2410 I24 P4 Control group Post-cholecystectomy
0.8865369946605645
2411 I24 P5 Control group Post-cholecystectomy
0.8365751334858886
2412 I24 P9 Control group Post-cholecystectomy
0.9096109839816934
2413 I24 P13 Control group Post-cholecystectomy
0.8070175438596491
2414 I24 P15 Control group Post-cholecystectomy
0.7974828375286042
2415 I24 P16 Control group Post-cholecystectomy
0.9418382913806255
2416 I24 P17 Control group Post-cholecystectomy
0.8260869565217391
2417 I24 P20 Control group Post-cholecystectomy
0.8586956521739131
2418 I24 P21 Control group Post-cholecystectomy
0.8653699466056446
2419 I24 P24 Control group Post-cholecystectomy
0.8556445461479787
2420 I24 P26 Control group Post-cholecystectomy
0.8794813119755912
2421 I24 P30 Control group Post-cholecystectomy
0.9590007627765065
2422 I24 P33 Control group Post-cholecystectomy
0.8039664378337147
2423 I24 P35 Control group Post-cholecystectomy
0.8508771929824561
2424 I24 P38 Control group Post-cholecystectomy
0.9786422578184591
2425 I24 P39 Control group Post-cholecystectomy
0.9532799389778794
2426 I24 P42 Control group Post-cholecystectomy
0.9347826086956522
2427 I24 P43 Control group Post-cholecystectomy
0.8506864988558352
2428 I24 P46 Control group Post-cholecystectomy
0.6592295957284515
2429 I24 P47 Control group Post-cholecystectomy
0.5379481311975591
2430 I24 P50 Control group Post-cholecystectomy

0.8197940503432495
2431	I24	P55	Control group	Post-cholecystectomy
0.801487414187643
2432	I24	P58	Control group	Post-cholecystectomy
0.881769641495042
2433	I24	P60	Control group	Post-cholecystectomy
0.9757818459191457
2434	I24	P63	Control group	Post-cholecystectomy
0.8506864988558352
2435	I24	P65	Control group	Post-cholecystectomy
0.8455377574370709
2436	I24	P68	Control group	Post-cholecystectomy
0.8892067124332571
2437	I24	P70	Control group	Post-cholecystectomy
0.8255148741418764
2438	I24	P71	Control group	Post-cholecystectomy
0.7225400457665904
2439	I24	P74	Control group	Post-cholecystectomy
0.8674675819984744
2440	I24	P75	Control group	Post-cholecystectomy
0.813119755911518
2441	I25	P1	Control group	Post-cholecystectomy
0.9302059496567505
2442	I25	P2	Control group	Post-cholecystectomy
0.7677345537757437
2443	I25	P4	Control group	Post-cholecystectomy
0.8094965675057209
2444	I25	P5	Control group	Post-cholecystectomy
0.7210144927536232
2445	I25	P9	Control group	Post-cholecystectomy
0.8348588863463006
2446	I25	P13	Control group	Post-cholecystectomy
0.7141495041952708
2447	I25	P15	Control group	Post-cholecystectomy
0.8188405797101449
2448	I25	P16	Control group	Post-cholecystectomy
0.8243707093821511
2449	I25	P17	Control group	Post-cholecystectomy
0.7715484363081617
2450	I25	P20	Control group	Post-cholecystectomy
0.7313119755911518
2451	I25	P21	Control group	Post-cholecystectomy
0.7784134248665141
2452	I25	P24	Control group	Post-cholecystectomy
0.7570556826849733
2453	I25	P26	Control group	Post-cholecystectomy
0.8335240274599542
2454	I25	P30	Control group	Post-cholecystectomy
0.9006483600305111
2455	I25	P33	Control group	Post-cholecystectomy
0.7978642257818459
2456	I25	P35	Control group	Post-cholecystectomy
0.8451563691838292
2457	I25	P38	Control group	Post-cholecystectomy

0.9515636918382914
2458	I25	P39	Control group	Post-cholecystectomy
0.8672768878718535
2459	I25	P42	Control group	Post-cholecystectomy
0.9101830663615561
2460	I25	P43	Control group	Post-cholecystectomy
0.7236842105263158
2461	I25	P46	Control group	Post-cholecystectomy
0.7789855072463768
2462	I25	P47	Control group	Post-cholecystectomy
0.9366895499618612
2463	I25	P50	Control group	Post-cholecystectomy
0.7545766590389016
2464	I25	P55	Control group	Post-cholecystectomy
0.6510297482837528
2465	I25	P58	Control group	Post-cholecystectomy
0.8249427917620137
2466	I25	P60	Control group	Post-cholecystectomy
0.9126620900076278
2467	I25	P63	Control group	Post-cholecystectomy
0.7913806254767353
2468	I25	P65	Control group	Post-cholecystectomy
0.7908085430968727
2469	I25	P68	Control group	Post-cholecystectomy
0.8476353928299009
2470	I25	P70	Control group	Post-cholecystectomy
0.8720442410373761
2471	I25	P71	Control group	Post-cholecystectomy
0.8634630053394355
2472	I25	P74	Control group	Post-cholecystectomy
0.8220823798627003
2473	I25	P75	Control group	Post-cholecystectomy
0.7288329519450801
2474	I26	P1	Control group	Post-cholecystectomy
0.950419527078566
2475	I26	P2	Control group	Post-cholecystectomy
0.7471395881006865
2476	I26	P4	Control group	Post-cholecystectomy
0.839626239511823
2477	I26	P5	Control group	Post-cholecystectomy
0.7006102212051869
2478	I26	P9	Control group	Post-cholecystectomy
0.7839435545385202
2479	I26	P13	Control group	Post-cholecystectomy	0.75
2480	I26	P15	Control group	Post-cholecystectomy
0.8810068649885584
2481	I26	P16	Control group	Post-cholecystectomy
0.7953852021357742
2482	I26	P17	Control group	Post-cholecystectomy
0.8171243325705568
2483	I26	P20	Control group	Post-cholecystectomy
0.8236079328756675
2484	I26	P21	Control group	Post-cholecystectomy
0.7008009153318078

2485	I26	P24	Control group	Post-cholecystectomy
0.8562166285278413
2486	I26	P26	Control group	Post-cholecystectomy
0.6523646071700991
2487	I26	P30	Control group	Post-cholecystectomy
0.9302059496567505
2488	I26	P33	Control group	Post-cholecystectomy
0.6969870327993898
2489	I26	P35	Control group	Post-cholecystectomy
0.6960335621662853
2490	I26	P38	Control group	Post-cholecystectomy
0.9235316552250191
2491	I26	P39	Control group	Post-cholecystectomy
0.8831045003813882
2492	I26	P42	Control group	Post-cholecystectomy
0.8884439359267735
2493	I26	P43	Control group	Post-cholecystectomy
0.6750572082379863
2494	I26	P46	Control group	Post-cholecystectomy
0.7698321891685737
2495	I26	P47	Control group	Post-cholecystectomy
0.9244851258581236
2496	I26	P50	Control group	Post-cholecystectomy
0.6996567505720824
2497	I26	P55	Control group	Post-cholecystectomy
0.7221586575133486
2498	I26	P58	Control group	Post-cholecystectomy
0.7902364607170099
2499	I26	P60	Control group	Post-cholecystectomy
0.9014111365369947
2500	I26	P63	Control group	Post-cholecystectomy
0.8375286041189931
2501	I26	P65	Control group	Post-cholecystectomy
0.7700228832951945
2502	I26	P68	Control group	Post-cholecystectomy
0.7988176964149504
2503	I26	P70	Control group	Post-cholecystectomy
0.7694508009153318
2504	I26	P71	Control group	Post-cholecystectomy
0.7942410373760488
2505	I26	P74	Control group	Post-cholecystectomy
0.5409992372234935
2506	I26	P75	Control group	Post-cholecystectomy
0.7860411899313501
2507	I27	P1	Control group	Post-cholecystectomy
0.8857742181540809
2508	I27	P2	Control group	Post-cholecystectomy
0.7602974828375286
2509	I27	P4	Control group	Post-cholecystectomy
0.7793668954996186
2510	I27	P5	Control group	Post-cholecystectomy
0.719488939740656
2511	I27	P9	Control group	Post-cholecystectomy
0.7242562929061785

2512 I27 P13 Control group Post-cholecystectomy
0.7353165522501907
2513 I27 P15 Control group Post-cholecystectomy
0.8463005339435545
2514 I27 P16 Control group Post-cholecystectomy
0.8287566742944318
2515 I27 P17 Control group Post-cholecystectomy
0.7644927536231884
2516 I27 P20 Control group Post-cholecystectomy
0.6405415713196033
2517 I27 P21 Control group Post-cholecystectomy
0.6048817696414951
2518 I27 P24 Control group Post-cholecystectomy
0.8096872616323417
2519 I27 P26 Control group Post-cholecystectomy
0.7107170099160945
2520 I27 P30 Control group Post-cholecystectomy
0.9223874904652937
2521 I27 P33 Control group Post-cholecystectomy
0.7379862700228833
2522 I27 P35 Control group Post-cholecystectomy
0.6826849733028223
2523 I27 P38 Control group Post-cholecystectomy
0.9511823035850496
2524 I27 P39 Control group Post-cholecystectomy
0.7852784134248665
2525 I27 P42 Control group Post-cholecystectomy
0.8653699466056446
2526 I27 P43 Control group Post-cholecystectomy
0.7654462242562929
2527 I27 P46 Control group Post-cholecystectomy
0.8093058733790999
2528 I27 P47 Control group Post-cholecystectomy
0.9263920671243325
2529 I27 P50 Control group Post-cholecystectomy
0.690884820747521
2530 I27 P55 Control group Post-cholecystectomy
0.7646834477498093
2531 I27 P58 Control group Post-cholecystectomy
0.7234935163996948
2532 I27 P60 Control group Post-cholecystectomy
0.8800533943554538
2533 I27 P63 Control group Post-cholecystectomy
0.7909992372234935
2534 I27 P65 Control group Post-cholecystectomy
0.6727688787185355
2535 I27 P68 Control group Post-cholecystectomy
0.6731502669717773
2536 I27 P70 Control group Post-cholecystectomy
0.6786803966437833
2537 I27 P71 Control group Post-cholecystectomy
0.7343630816170862
2538 I27 P74 Control group Post-cholecystectomy
0.6903127383676583

2539	I27	P75	Control group	Post-cholecystectomy	0.5903890160183066
2540	I28	P1	Control group	Post-cholecystectomy	0.8476353928299009
2541	I28	P2	Control group	Post-cholecystectomy	0.916094584286804
2542	I28	P4	Control group	Post-cholecystectomy	0.7479023646071701
2543	I28	P5	Control group	Post-cholecystectomy	0.8823417238749046
2544	I28	P9	Control group	Post-cholecystectomy	0.8901601830663616
2545	I28	P13	Control group	Post-cholecystectomy	0.8611746758199847
2546	I28	P15	Control group	Post-cholecystectomy	0.8127383676582761
2547	I28	P16	Control group	Post-cholecystectomy	0.7433257055682685
2548	I28	P17	Control group	Post-cholecystectomy	0.8886346300533944
2549	I28	P20	Control group	Post-cholecystectomy	0.5930587337909993
2550	I28	P21	Control group	Post-cholecystectomy	0.8375286041189931
2551	I28	P24	Control group	Post-cholecystectomy	0.9244851258581236
2552	I28	P26	Control group	Post-cholecystectomy	0.8735697940503433
2553	I28	P30	Control group	Post-cholecystectomy	0.8825324180015256
2554	I28	P33	Control group	Post-cholecystectomy	0.8337147215865751
2555	I28	P35	Control group	Post-cholecystectomy	0.8926392067124332
2556	I28	P38	Control group	Post-cholecystectomy	0.9241037376048817
2557	I28	P39	Control group	Post-cholecystectomy	0.7789855072463768
2558	I28	P42	Control group	Post-cholecystectomy	0.9220061022120518
2559	I28	P43	Control group	Post-cholecystectomy	0.7379862700228833
2560	I28	P46	Control group	Post-cholecystectomy	0.8442028985507246
2561	I28	P47	Control group	Post-cholecystectomy	0.9176201372997712
2562	I28	P50	Control group	Post-cholecystectomy	0.8710907704042715
2563	I28	P55	Control group	Post-cholecystectomy	0.8585049580472921
2564	I28	P58	Control group	Post-cholecystectomy	0.9139969488939741
2565	I28	P60	Control group	Post-cholecystectomy	0.7210144927536232

2566	I28	P63	Control group	Post-cholecystectomy	0.8455377574370709
2567	I28	P65	Control group	Post-cholecystectomy	0.8832951945080092
2568	I28	P68	Control group	Post-cholecystectomy	0.9117086193745233
2569	I28	P70	Control group	Post-cholecystectomy	0.8157894736842105
2570	I28	P71	Control group	Post-cholecystectomy	0.9157131960335622
2571	I28	P74	Control group	Post-cholecystectomy	0.9057971014492754
2572	I28	P75	Control group	Post-cholecystectomy	0.8422959572845157
2573	I29	P1	Control group	Post-cholecystectomy	0.9326849733028223
2574	I29	P2	Control group	Post-cholecystectomy	0.8314263920671243
2575	I29	P4	Control group	Post-cholecystectomy	0.8712814645308925
2576	I29	P5	Control group	Post-cholecystectomy	0.8407704042715485
2577	I29	P9	Control group	Post-cholecystectomy	0.8035850495804729
2578	I29	P13	Control group	Post-cholecystectomy	0.8190312738367659
2579	I29	P15	Control group	Post-cholecystectomy	0.876048817696415
2580	I29	P16	Control group	Post-cholecystectomy	0.9187643020594966
2581	I29	P17	Control group	Post-cholecystectomy	0.7917620137299771
2582	I29	P20	Control group	Post-cholecystectomy	0.8335240274599542
2583	I29	P21	Control group	Post-cholecystectomy	0.8741418764302059
2584	I29	P24	Control group	Post-cholecystectomy	0.8163615560640732
2585	I29	P26	Control group	Post-cholecystectomy	0.834096109839817
2586	I29	P30	Control group	Post-cholecystectomy	0.9416475972540046
2587	I29	P33	Control group	Post-cholecystectomy	0.8438215102974829
2588	I29	P35	Control group	Post-cholecystectomy	0.8733790999237223
2589	I29	P38	Control group	Post-cholecystectomy	0.9620518688024409
2590	I29	P39	Control group	Post-cholecystectomy	0.9500381388253242
2591	I29	P42	Control group	Post-cholecystectomy	0.9441266209000763
2592	I29	P43	Control group	Post-cholecystectomy	0.7513348588863463

2593 I29 P46 Control group Post-cholecystectomy
0.8617467581998475
2594 I29 P47 Control group Post-cholecystectomy
0.9416475972540046
2595 I29 P50 Control group Post-cholecystectomy
0.8299008390541571
2596 I29 P55 Control group Post-cholecystectomy
0.8211289092295957
2597 I29 P58 Control group Post-cholecystectomy
0.7971014492753623
2598 I29 P60 Control group Post-cholecystectomy
0.9174294431731502
2599 I29 P63 Control group Post-cholecystectomy
0.7845156369183829
2600 I29 P65 Control group Post-cholecystectomy
0.7776506483600305
2601 I29 P68 Control group Post-cholecystectomy
0.8352402745995423
2602 I29 P70 Control group Post-cholecystectomy
0.8649885583524027
2603 I29 P71 Control group Post-cholecystectomy
0.8962623951182304
2604 I29 P74 Control group Post-cholecystectomy
0.7942410373760488
2605 I29 P75 Control group Post-cholecystectomy
0.809115179252479
2606 I30 P1 Control group Post-cholecystectomy
0.9685354691075515
2607 I30 P2 Control group Post-cholecystectomy
0.8522120518688024
2608 I30 P4 Control group Post-cholecystectomy
0.8043478260869565
2609 I30 P5 Control group Post-cholecystectomy
0.7585812356979404
2610 I30 P9 Control group Post-cholecystectomy
0.7295957284515637
2611 I30 P13 Control group Post-cholecystectomy
0.8163615560640732
2612 I30 P15 Control group Post-cholecystectomy
0.8737604881769642
2613 I30 P16 Control group Post-cholecystectomy
0.7869946605644547
2614 I30 P17 Control group Post-cholecystectomy
0.8325705568268498
2615 I30 P20 Control group Post-cholecystectomy
0.7601067887109078
2616 I30 P21 Control group Post-cholecystectomy
0.6334858886346301
2617 I30 P24 Control group Post-cholecystectomy
0.8787185354691075
2618 I30 P26 Control group Post-cholecystectomy
0.7238749046529367
2619 I30 P30 Control group Post-cholecystectomy
0.9256292906178489

2620	I30	P33	Control group	Post-cholecystectomy
0.639397406559878
2621	I30	P35	Control group	Post-cholecystectomy
0.772883295194508
2622	I30	P38	Control group	Post-cholecystectomy
0.9395499618611747
2623	I30	P39	Control group	Post-cholecystectomy
0.8007246376811594
2624	I30	P42	Control group	Post-cholecystectomy
0.7601067887109078
2625	I30	P43	Control group	Post-cholecystectomy
0.8012967200610221
2626	I30	P46	Control group	Post-cholecystectomy
0.7948131197559115
2627	I30	P47	Control group	Post-cholecystectomy
0.956140350877193
2628	I30	P50	Control group	Post-cholecystectomy
0.7597254004576659
2629	I30	P55	Control group	Post-cholecystectomy
0.7084286803966438
2630	I30	P58	Control group	Post-cholecystectomy
0.7032799389778794
2631	I30	P60	Control group	Post-cholecystectomy
0.9046529366895499
2632	I30	P63	Control group	Post-cholecystectomy
0.7254004576659039
2633	I30	P65	Control group	Post-cholecystectomy
0.7591533180778032
2634	I30	P68	Control group	Post-cholecystectomy
0.7170099160945843
2635	I30	P70	Control group	Post-cholecystectomy
0.725209763539283
2636	I30	P71	Control group	Post-cholecystectomy
0.8712814645308925
2637	I30	P74	Control group	Post-cholecystectomy
0.8041571319603357
2638	I30	P75	Control group	Post-cholecystectomy
0.755720823798627
2639	I31	P1	Control group	Post-cholecystectomy
0.9698703279938978
2640	I31	P2	Control group	Post-cholecystectomy
0.8525934401220442
2641	I31	P4	Control group	Post-cholecystectomy
0.8581235697940504
2642	I31	P5	Control group	Post-cholecystectomy
0.8228451563691839
2643	I31	P9	Control group	Post-cholecystectomy
0.8993135011441648
2644	I31	P13	Control group	Post-cholecystectomy
0.7484744469870328
2645	I31	P15	Control group	Post-cholecystectomy
0.8442028985507246
2646	I31	P16	Control group	Post-cholecystectomy
0.915903890160183

2647	I31	P17	Control group	Post-cholecystectomy
0.8049199084668193
2648	I31	P20	Control group	Post-cholecystectomy
0.7932875667429443
2649	I31	P21	Control group	Post-cholecystectomy
0.8840579710144928
2650	I31	P24	Control group	Post-cholecystectomy
0.7896643783371472
2651	I31	P26	Control group	Post-cholecystectomy
0.852974828375286
2652	I31	P30	Control group	Post-cholecystectomy
0.910373760488177
2653	I31	P33	Control group	Post-cholecystectomy
0.8699466056445462
2654	I31	P35	Control group	Post-cholecystectomy
0.8537376048817696
2655	I31	P38	Control group	Post-cholecystectomy
0.9731121281464531
2656	I31	P39	Control group	Post-cholecystectomy
0.9017925247902364
2657	I31	P42	Control group	Post-cholecystectomy
0.9748283752860412
2658	I31	P43	Control group	Post-cholecystectomy
0.7982456140350878
2659	I31	P46	Control group	Post-cholecystectomy
0.8953089244851259
2660	I31	P47	Control group	Post-cholecystectomy
0.9385964912280702
2661	I31	P50	Control group	Post-cholecystectomy
0.851067887109077
2662	I31	P55	Control group	Post-cholecystectomy
0.7961479786422578
2663	I31	P58	Control group	Post-cholecystectomy
0.8846300533943554
2664	I31	P60	Control group	Post-cholecystectomy
0.9200991609458429
2665	I31	P63	Control group	Post-cholecystectomy
0.8022501906941266
2666	I31	P65	Control group	Post-cholecystectomy
0.8325705568268498
2667	I31	P68	Control group	Post-cholecystectomy
0.9151411136536994
2668	I31	P70	Control group	Post-cholecystectomy
0.8983600305110603
2669	I31	P71	Control group	Post-cholecystectomy
0.92372234935164
2670	I31	P74	Control group	Post-cholecystectomy
0.8558352402745996
2671	I31	P75	Control group	Post-cholecystectomy
0.7894736842105263
2672	I32	P1	Control group	Post-cholecystectomy
0.9530892448512586
2673	I32	P2	Control group	Post-cholecystectomy
0.914187643020595

2674	I32	P4	Control group	Post-cholecystectomy	0.8531655225019069
2675	I32	P5	Control group	Post-cholecystectomy	0.8453470633104501
2676	I32	P9	Control group	Post-cholecystectomy	0.9302059496567505
2677	I32	P13	Control group	Post-cholecystectomy	0.6462623951182304
2678	I32	P15	Control group	Post-cholecystectomy	0.8792906178489702
2679	I32	P16	Control group	Post-cholecystectomy	0.8956903127383676
2680	I32	P17	Control group	Post-cholecystectomy	0.8920671243325705
2681	I32	P20	Control group	Post-cholecystectomy	0.7873760488176964
2682	I32	P21	Control group	Post-cholecystectomy	0.8871090770404272
2683	I32	P24	Control group	Post-cholecystectomy	0.8287566742944318
2684	I32	P26	Control group	Post-cholecystectomy	0.8705186880244088
2685	I32	P30	Control group	Post-cholecystectomy	0.9544241037376049
2686	I32	P33	Control group	Post-cholecystectomy	0.8459191456903128
2687	I32	P35	Control group	Post-cholecystectomy	0.8611746758199847
2688	I32	P38	Control group	Post-cholecystectomy	0.9858886346300534
2689	I32	P39	Control group	Post-cholecystectomy	0.8979786422578184
2690	I32	P42	Control group	Post-cholecystectomy	0.9557589626239512
2691	I32	P43	Control group	Post-cholecystectomy	0.7732646834477498
2692	I32	P46	Control group	Post-cholecystectomy	0.8642257818459191
2693	I32	P47	Control group	Post-cholecystectomy	0.9429824561403509
2694	I32	P50	Control group	Post-cholecystectomy	0.8632723112128147
2695	I32	P55	Control group	Post-cholecystectomy	0.7547673531655225
2696	I32	P58	Control group	Post-cholecystectomy	0.9115179252479023
2697	I32	P60	Control group	Post-cholecystectomy	0.9431731502669718
2698	I32	P63	Control group	Post-cholecystectomy	0.8350495804729214
2699	I32	P65	Control group	Post-cholecystectomy	0.841723874904653
2700	I32	P68	Control group	Post-cholecystectomy	0.8806254767353165

2701	I32	P70	Control group	Post-cholecystectomy
0.8859649122807017
2702	I32	P71	Control group	Post-cholecystectomy
0.8985507246376812
2703	I32	P74	Control group	Post-cholecystectomy
0.8548817696414951
2704	I32	P75	Control group	Post-cholecystectomy
0.8192219679633868
2705	I33	P1	Control group	Post-cholecystectomy
0.9649122807017544
2706	I33	P2	Control group	Post-cholecystectomy
0.7757437070938215
2707	I33	P4	Control group	Post-cholecystectomy
0.8571700991609459
2708	I33	P5	Control group	Post-cholecystectomy
0.8159801678108314
2709	I33	P9	Control group	Post-cholecystectomy
0.8775743707093822
2710	I33	P13	Control group	Post-cholecystectomy
0.8049199084668193
2711	I33	P15	Control group	Post-cholecystectomy
0.7696414950419527
2712	I33	P16	Control group	Post-cholecystectomy
0.86441647597254
2713	I33	P17	Control group	Post-cholecystectomy
0.7984363081617086
2714	I33	P20	Control group	Post-cholecystectomy
0.8152173913043478
2715	I33	P21	Control group	Post-cholecystectomy
0.8445842868039665
2716	I33	P24	Control group	Post-cholecystectomy
0.8625095347063311
2717	I33	P26	Control group	Post-cholecystectomy
0.8750953470633105
2718	I33	P30	Control group	Post-cholecystectomy
0.9376430205949656
2719	I33	P33	Control group	Post-cholecystectomy
0.8392448512585813
2720	I33	P35	Control group	Post-cholecystectomy
0.8283752860411899
2721	I33	P38	Control group	Post-cholecystectomy
0.9719679633867276
2722	I33	P39	Control group	Post-cholecystectomy
0.9004576659038902
2723	I33	P42	Control group	Post-cholecystectomy
0.9317315026697178
2724	I33	P43	Control group	Post-cholecystectomy
0.8560259344012204
2725	I33	P46	Control group	Post-cholecystectomy
0.6043096872616324
2726	I33	P47	Control group	Post-cholecystectomy
0.5694126620900076
2727	I33	P50	Control group	Post-cholecystectomy
0.7789855072463768

2728	I33	P55	Control group	Post-cholecystectomy
0.7936689549961862
2729	I33	P58	Control group	Post-cholecystectomy
0.8485888634630053
2730	I33	P60	Control group	Post-cholecystectomy
0.9412662090007627
2731	I33	P63	Control group	Post-cholecystectomy
0.8264683447749809
2732	I33	P65	Control group	Post-cholecystectomy
0.8028222730739893
2733	I33	P68	Control group	Post-cholecystectomy
0.8768115942028986
2734	I33	P70	Control group	Post-cholecystectomy
0.8121662852784134
2735	I33	P71	Control group	Post-cholecystectomy
0.7027078565980168
2736	I33	P74	Control group	Post-cholecystectomy
0.8304729214340199
2737	I33	P75	Control group	Post-cholecystectomy
0.7902364607170099
2738	I34	P1	Control group	Post-cholecystectomy
0.9624332570556827
2739	I34	P2	Control group	Post-cholecystectomy
0.7925247902364607
2740	I34	P4	Control group	Post-cholecystectomy
0.8962623951182304
2741	I34	P5	Control group	Post-cholecystectomy
0.7673531655225019
2742	I34	P9	Control group	Post-cholecystectomy
0.7898550724637681
2743	I34	P13	Control group	Post-cholecystectomy
0.8771929824561403
2744	I34	P15	Control group	Post-cholecystectomy
0.7749809305873379
2745	I34	P16	Control group	Post-cholecystectomy
0.8726163234172387
2746	I34	P17	Control group	Post-cholecystectomy
0.8135011441647597
2747	I34	P20	Control group	Post-cholecystectomy
0.8075896262395118
2748	I34	P21	Control group	Post-cholecystectomy
0.7656369183829138
2749	I34	P24	Control group	Post-cholecystectomy
0.9044622425629291
2750	I34	P26	Control group	Post-cholecystectomy
0.8077803203661327
2751	I34	P30	Control group	Post-cholecystectomy
0.9147597254004577
2752	I34	P33	Control group	Post-cholecystectomy
0.8209382151029748
2753	I34	P35	Control group	Post-cholecystectomy
0.753813882532418
2754	I34	P38	Control group	Post-cholecystectomy
0.9105644546147978

2755 I34 P39 Control group Post-cholecystectomy
0.9183829138062548
2756 I34 P42 Control group Post-cholecystectomy
0.7030892448512586
2757 I34 P43 Control group Post-cholecystectomy
0.9063691838291381
2758 I34 P46 Control group Post-cholecystectomy
0.7091914569031273
2759 I34 P47 Control group Post-cholecystectomy
0.7797482837528604
2760 I34 P50 Control group Post-cholecystectomy
0.7827993897787948
2761 I34 P55 Control group Post-cholecystectomy
0.8255148741418764
2762 I34 P58 Control group Post-cholecystectomy
0.78813882532418
2763 I34 P60 Control group Post-cholecystectomy
0.9492753623188406
2764 I34 P63 Control group Post-cholecystectomy
0.8846300533943554
2765 I34 P65 Control group Post-cholecystectomy
0.7967200610221206
2766 I34 P68 Control group Post-cholecystectomy
0.8176964149504196
2767 I34 P70 Control group Post-cholecystectomy
0.8045385202135774
2768 I34 P71 Control group Post-cholecystectomy
0.719488939740656
2769 I34 P74 Control group Post-cholecystectomy
0.8493516399694889
2770 I34 P75 Control group Post-cholecystectomy
0.7936689549961862
2771 I35 P1 Control group Post-cholecystectomy
0.8773836765827613
2772 I35 P2 Control group Post-cholecystectomy
0.7894736842105263
2773 I35 P4 Control group Post-cholecystectomy
0.7393211289092296
2774 I35 P5 Control group Post-cholecystectomy
0.8127383676582761
2775 I35 P9 Control group Post-cholecystectomy
0.8735697940503433
2776 I35 P13 Control group Post-cholecystectomy
0.7929061784897025
2777 I35 P15 Control group Post-cholecystectomy
0.679252479023646
2778 I35 P16 Control group Post-cholecystectomy
0.8369565217391305
2779 I35 P17 Control group Post-cholecystectomy
0.7827993897787948
2780 I35 P20 Control group Post-cholecystectomy
0.6746758199847445
2781 I35 P21 Control group Post-cholecystectomy
0.8535469107551488

2782	I35	P24	Control group	Post-cholecystectomy
0.8033943554538521
2783	I35	P26	Control group	Post-cholecystectomy
0.8621281464530892
2784	I35	P30	Control group	Post-cholecystectomy
0.9296338672768879
2785	I35	P33	Control group	Post-cholecystectomy
0.8363844393592678
2786	I35	P35	Control group	Post-cholecystectomy
0.8175057208237986
2787	I35	P38	Control group	Post-cholecystectomy
0.9340198321891686
2788	I35	P39	Control group	Post-cholecystectomy
0.8346681922196796
2789	I35	P42	Control group	Post-cholecystectomy
0.9576659038901602
2790	I35	P43	Control group	Post-cholecystectomy
0.7072845156369184
2791	I35	P46	Control group	Post-cholecystectomy
0.7929061784897025
2792	I35	P47	Control group	Post-cholecystectomy
0.9136155606407322
2793	I35	P50	Control group	Post-cholecystectomy
0.7745995423340961
2794	I35	P55	Control group	Post-cholecystectomy
0.7625858123569794
2795	I35	P58	Control group	Post-cholecystectomy
0.816742944317315
2796	I35	P60	Control group	Post-cholecystectomy
0.8489702517162472
2797	I35	P63	Control group	Post-cholecystectomy
0.7940503432494279
2798	I35	P65	Control group	Post-cholecystectomy
0.8613653699466056
2799	I35	P68	Control group	Post-cholecystectomy
0.9138062547673532
2800	I35	P70	Control group	Post-cholecystectomy
0.8710907704042715
2801	I35	P71	Control group	Post-cholecystectomy
0.8874904652936689
2802	I35	P74	Control group	Post-cholecystectomy
0.818649885583524
2803	I35	P75	Control group	Post-cholecystectomy
0.7559115179252479
2804	I36	P1	Control group	Post-cholecystectomy
0.8628909229595728
2805	I36	P2	Control group	Post-cholecystectomy
0.9639588100686499
2806	I36	P4	Control group	Post-cholecystectomy
0.9891304347826086
2807	I36	P5	Control group	Post-cholecystectomy
0.9959954233409611
2808	I36	P9	Control group	Post-cholecystectomy
0.9908466819221968

2809	I36	P13	Control group	Post-cholecystectomy
0.9973302822273074				
2810	I36	P15	Control group	Post-cholecystectomy
0.969488939740656				
2811	I36	P16	Control group	Post-cholecystectomy
0.979023646071701				
2812	I36	P17	Control group	Post-cholecystectomy
0.9969488939740656				
2813	I36	P20	Control group	Post-cholecystectomy
0.9118993135011442				
2814	I36	P21	Control group	Post-cholecystectomy
0.982837528604119				
2815	I36	P24	Control group	Post-cholecystectomy
0.9937070938215103				
2816	I36	P26	Control group	Post-cholecystectomy
0.9887490465293669				
2817	I36	P30	Control group	Post-cholecystectomy
0.9683447749809306				
2818	I36	P33	Control group	Post-cholecystectomy
0.9879862700228833				
2819	I36	P35	Control group	Post-cholecystectomy
0.9870327993897788				
2820	I36	P38	Control group	Post-cholecystectomy
0.893211289092296				
2821	I36	P39	Control group	Post-cholecystectomy
0.9601449275362319				
2822	I36	P42	Control group	Post-cholecystectomy
0.9811212814645309				
2823	I36	P43	Control group	Post-cholecystectomy
0.9931350114416476				
2824	I36	P46	Control group	Post-cholecystectomy
0.9647215865751335				
2825	I36	P47	Control group	Post-cholecystectomy
0.9593821510297483				
2826	I36	P50	Control group	Post-cholecystectomy
0.9929443173150267				
2827	I36	P55	Control group	Post-cholecystectomy
0.9998093058733791				
2828	I36	P58	Control group	Post-cholecystectomy
0.9841723874904653				
2829	I36	P60	Control group	Post-cholecystectomy
0.9715865751334859				
2830	I36	P63	Control group	Post-cholecystectomy
0.9952326468344775				
2831	I36	P65	Control group	Post-cholecystectomy
0.9933257055682685				
2832	I36	P68	Control group	Post-cholecystectomy
0.979023646071701				
2833	I36	P70	Control group	Post-cholecystectomy
0.9654843630816171				
2834	I36	P71	Control group	Post-cholecystectomy
0.9958047292143402				
2835	I36	P74	Control group	Post-cholecystectomy
0.9630053394355453				

2836	I36	P75	Control group	Post-cholecystectomy
0.9975209763539283
2837	I37	P1	Control group	Post-cholecystectomy
0.8062547673531655
2838	I37	P2	Control group	Post-cholecystectomy
0.8758581235697941
2839	I37	P4	Control group	Post-cholecystectomy
0.8371472158657514
2840	I37	P5	Control group	Post-cholecystectomy
0.8535469107551488
2841	I37	P9	Control group	Post-cholecystectomy
0.8686117467581999
2842	I37	P13	Control group	Post-cholecystectomy
0.9223874904652937
2843	I37	P15	Control group	Post-cholecystectomy
0.7971014492753623
2844	I37	P16	Control group	Post-cholecystectomy
0.8979786422578184
2845	I37	P17	Control group	Post-cholecystectomy
0.919908466819222
2846	I37	P20	Control group	Post-cholecystectomy
0.8068268497330282
2847	I37	P21	Control group	Post-cholecystectomy
0.9328756674294432
2848	I37	P24	Control group	Post-cholecystectomy
0.8838672768878718
2849	I37	P26	Control group	Post-cholecystectomy
0.9511823035850496
2850	I37	P30	Control group	Post-cholecystectomy
0.8691838291380626
2851	I37	P33	Control group	Post-cholecystectomy
0.9290617848970252
2852	I37	P35	Control group	Post-cholecystectomy
0.9202898550724637
2853	I37	P38	Control group	Post-cholecystectomy
0.9187643020594966
2854	I37	P39	Control group	Post-cholecystectomy
0.8806254767353165
2855	I37	P42	Control group	Post-cholecystectomy
0.9677726926010679
2856	I37	P43	Control group	Post-cholecystectomy
0.8813882532418001
2857	I37	P46	Control group	Post-cholecystectomy
0.9073226544622426
2858	I37	P47	Control group	Post-cholecystectomy
0.9420289855072463
2859	I37	P50	Control group	Post-cholecystectomy
0.8318077803203662
2860	I37	P55	Control group	Post-cholecystectomy
0.8257055682684973
2861	I37	P58	Control group	Post-cholecystectomy
0.9101830663615561
2862	I37	P60	Control group	Post-cholecystectomy
0.8745232646834478

2863 I37 P63 Control group Post-cholecystectomy
0.9057971014492754
2864 I37 P65 Control group Post-cholecystectomy
0.937070938215103
2865 I37 P68 Control group Post-cholecystectomy
0.9572845156369184
2866 I37 P70 Control group Post-cholecystectomy
0.897025171624714
2867 I37 P71 Control group Post-cholecystectomy
0.9666285278413425
2868 I37 P74 Control group Post-cholecystectomy
0.8943554538520213
2869 I37 P75 Control group Post-cholecystectomy
0.8783371472158658
2870 B27 C1 Control group Post-op CD
0.9645308924485125
2871 B27 C3 Control group Post-op CD
0.8968344774980931
2872 B27 C7 Control group Post-op CD
0.715675057208238
2873 B27 C8 Control group Post-op CD
0.9189549961861174
2874 B27 C11 Control group Post-op CD
0.9326849733028223
2875 B27 C15 Control group Post-op CD
0.9496567505720824
2876 B27 C19 Control group Post-op CD
0.8295194508009154
2877 B27 C22 Control group Post-op CD
0.9298245614035088
2878 B27 C26 Control group Post-op CD
0.9651029748283753
2879 B27 C28 Control group Post-op CD
0.9475591151792525
2880 B27 C31 Control group Post-op CD
0.9746376811594203
2881 B27 C35 Control group Post-op CD
0.7501906941266209
2882 B27 C38 Control group Post-op CD
0.9744469870327994
2883 B27 C40 Control group Post-op CD
0.9567124332570557
2884 B27 C44 Control group Post-op CD
0.9620518688024409
2885 B27 C47 Control group Post-op CD
0.9464149504195271
2886 B27 C48 Control group Post-op CD
0.7229214340198322
2887 B27 C49 Control group Post-op CD
0.8373379099923722
2888 B27 C53 Control group Post-op CD
0.8222730739893211
2889 B27 C56 Control group Post-op CD
0.7850877192982456

2890 B27 C60 Control group Post-op CD
0.7618230358504958
2891 B27 C62 Control group Post-op CD
0.8440122044241037
2892 B27 C64 Control group Post-op CD
0.811022120518688
2893 B27 C65 Control group Post-op CD
0.8094965675057209
2894 B27 C69 Control group Post-op CD
0.8239893211289092
2895 B27 C70 Control group Post-op CD
0.8213196033562167
2896 B27 C74 Control group Post-op CD
0.8413424866514111
2897 B27 C78 Control group Post-op CD
0.9193363844393593
2898 B66 C1 Control group Post-op CD
0.9412662090007627
2899 B66 C3 Control group Post-op CD
0.9412662090007627
2900 B66 C7 Control group Post-op CD
0.8861556064073226
2901 B66 C8 Control group Post-op CD
0.92372234935164
2902 B66 C11 Control group Post-op CD
0.7972921434019832
2903 B66 C15 Control group Post-op CD
0.8428680396643783
2904 B66 C19 Control group Post-op CD
0.8108314263920672
2905 B66 C22 Control group Post-op CD
0.9477498093058734
2906 B66 C26 Control group Post-op CD
0.9319221967963387
2907 B66 C28 Control group Post-op CD
0.8115942028985508
2908 B66 C31 Control group Post-op CD
0.8781464530892449
2909 B66 C35 Control group Post-op CD
0.8503051106025934
2910 B66 C38 Control group Post-op CD
0.9260106788710908
2911 B66 C40 Control group Post-op CD
0.9139969488939741
2912 B66 C44 Control group Post-op CD
0.8447749809305873
2913 B66 C47 Control group Post-op CD
0.8995041952707856
2914 B66 C48 Control group Post-op CD
0.9044622425629291
2915 B66 C49 Control group Post-op CD
0.950228832951945
2916 B66 C53 Control group Post-op CD
0.8575514874141876

2917	B66	C56	Control group	Post-op CD
0.8171243325705568
2918	B66	C60	Control group	Post-op CD
0.8363844393592678
2919	B66	C62	Control group	Post-op CD
0.8840579710144928
2920	B66	C64	Control group	Post-op CD
0.8251334858886347
2921	B66	C65	Control group	Post-op CD
0.9246758199847445
2922	B66	C69	Control group	Post-op CD
0.8476353928299009
2923	B66	C70	Control group	Post-op CD
0.9143783371472158
2924	B66	C74	Control group	Post-op CD
0.9509916094584286
2925	B66	C78	Control group	Post-op CD
0.7057589626239512
2926	B86	C1	Control group	Post-op CD
0.9563310450038138
2927	B86	C3	Control group	Post-op CD
0.9345919145690312
2928	B86	C7	Control group	Post-op CD
0.6561784897025171
2929	B86	C8	Control group	Post-op CD
0.9416475972540046
2930	B86	C11	Control group	Post-op CD
0.8813882532418001
2931	B86	C15	Control group	Post-op CD
0.8583142639206712
2932	B86	C19	Control group	Post-op CD
0.5932494279176201
2933	B86	C22	Control group	Post-op CD
0.9448893974065599
2934	B86	C26	Control group	Post-op CD
0.9637681159420289
2935	B86	C28	Control group	Post-op CD
0.8954996186117468
2936	B86	C31	Control group	Post-op CD
0.9719679633867276
2937	B86	C35	Control group	Post-op CD
0.8350495804729214
2938	B86	C38	Control group	Post-op CD
0.9296338672768879
2939	B86	C40	Control group	Post-op CD
0.9364988558352403
2940	B86	C44	Control group	Post-op CD
0.8762395118230358
2941	B86	C47	Control group	Post-op CD
0.8964530892448512
2942	B86	C48	Control group	Post-op CD
0.6287185354691075
2943	B86	C49	Control group	Post-op CD
0.8140732265446224

2944	B86	C53	Control group	Post-op CD
0.7206331045003814
2945	B86	C56	Control group	Post-op CD
0.679252479023646
2946	B86	C60	Control group	Post-op CD
0.7429443173150267
2947	B86	C62	Control group	Post-op CD
0.7694508009153318
2948	B86	C64	Control group	Post-op CD
0.7418001525553013
2949	B86	C65	Control group	Post-op CD
0.7027078565980168
2950	B86	C69	Control group	Post-op CD
0.685163996948894
2951	B86	C70	Control group	Post-op CD
0.7494279176201373
2952	B86	C74	Control group	Post-op CD
0.8081617086193745
2953	B86	C78	Control group	Post-op CD
0.8064454614797865
2954	B97	C1	Control group	Post-op CD
0.9738749046529367
2955	B97	C3	Control group	Post-op CD
0.8920671243325705
2956	B97	C7	Control group	Post-op CD
0.9145690312738368
2957	B97	C8	Control group	Post-op CD
0.900839054157132
2958	B97	C11	Control group	Post-op CD
0.8825324180015256
2959	B97	C15	Control group	Post-op CD
0.8756674294431731
2960	B97	C19	Control group	Post-op CD
0.8709000762776506
2961	B97	C22	Control group	Post-op CD
0.9105644546147978
2962	B97	C26	Control group	Post-op CD
0.90255530129672
2963	B97	C28	Control group	Post-op CD
0.8804347826086957
2964	B97	C31	Control group	Post-op CD
0.9099923722349351
2965	B97	C35	Control group	Post-op CD
0.88558352402746
2966	B97	C38	Control group	Post-op CD
0.9740655987795576
2967	B97	C40	Control group	Post-op CD
0.885392829900839
2968	B97	C44	Control group	Post-op CD
0.8880625476735317
2969	B97	C47	Control group	Post-op CD
0.9164759725400458
2970	B97	C48	Control group	Post-op CD
0.8485888634630053

2971 B97 C49 Control group Post-op CD
0.8495423340961098
2972 B97 C53 Control group Post-op CD
0.8537376048817696
2973 B97 C56 Control group Post-op CD
0.8054919908466819
2974 B97 C60 Control group Post-op CD
0.8440122044241037
2975 B97 C62 Control group Post-op CD
0.8790999237223494
2976 B97 C64 Control group Post-op CD
0.8625095347063311
2977 B97 C65 Control group Post-op CD
0.8857742181540809
2978 B97 C69 Control group Post-op CD
0.8255148741418764
2979 B97 C70 Control group Post-op CD
0.8872997711670481
2980 B97 C74 Control group Post-op CD
0.916094584286804
2981 B97 C78 Control group Post-op CD
0.7503813882532419
2982 B98 C1 Control group Post-op CD
0.9422196796338673
2983 B98 C3 Control group Post-op CD
0.8363844393592678
2984 B98 C7 Control group Post-op CD
0.9183829138062548
2985 B98 C8 Control group Post-op CD
0.8861556064073226
2986 B98 C11 Control group Post-op CD
0.8907322654462243
2987 B98 C15 Control group Post-op CD
0.8920671243325705
2988 B98 C19 Control group Post-op CD
0.8392448512585813
2989 B98 C22 Control group Post-op CD
0.9000762776506483
2990 B98 C26 Control group Post-op CD
0.9157131960335622
2991 B98 C28 Control group Post-op CD
0.9113272311212814
2992 B98 C31 Control group Post-op CD
0.9748283752860412
2993 B98 C35 Control group Post-op CD
0.88558352402746
2994 B98 C38 Control group Post-op CD
0.971205186880244
2995 B98 C40 Control group Post-op CD
0.9099923722349351
2996 B98 C44 Control group Post-op CD
0.8913043478260869
2997 B98 C47 Control group Post-op CD
0.9210526315789473

2998 B98 C48 Control group Post-op CD
0.8871090770404272
2999 B98 C49 Control group Post-op CD
0.9002669717772692
3000 B98 C53 Control group Post-op CD
0.8790999237223494
3001 B98 C56 Control group Post-op CD
0.8756674294431731
3002 B98 C60 Control group Post-op CD
0.8621281464530892
3003 B98 C62 Control group Post-op CD
0.9122807017543859
3004 B98 C64 Control group Post-op CD
0.893211289092296
3005 B98 C65 Control group Post-op CD
0.9130434782608695
3006 B98 C69 Control group Post-op CD
0.8539282990083905
3007 B98 C70 Control group Post-op CD
0.898932112890923
3008 B98 C74 Control group Post-op CD
0.927536231884058
3009 B98 C78 Control group Post-op CD
0.8506864988558352
3010 B100 C1 Control group Post-op CD
0.9355453852021358
3011 B100 C3 Control group Post-op CD
0.9176201372997712
3012 B100 C7 Control group Post-op CD
0.9856979405034325
3013 B100 C8 Control group Post-op CD
0.9530892448512586
3014 B100 C11 Control group Post-op CD
0.889397406559878
3015 B100 C15 Control group Post-op CD
0.9221967963386728
3016 B100 C19 Control group Post-op CD
0.9759725400457666
3017 B100 C22 Control group Post-op CD
0.9096109839816934
3018 B100 C26 Control group Post-op CD
0.8832951945080092
3019 B100 C28 Control group Post-op CD
0.9376430205949656
3020 B100 C31 Control group Post-op CD
0.8436308161708619
3021 B100 C35 Control group Post-op CD
0.9450800915331807
3022 B100 C38 Control group Post-op CD
0.8094965675057209
3023 B100 C40 Control group Post-op CD
0.8878718535469108
3024 B100 C44 Control group Post-op CD
0.976926010678871

3025	B100	C47	Control group	Post-op CD	0.9836003051106026
3026	B100	C48	Control group	Post-op CD	0.9567124332570557
3027	B100	C49	Control group	Post-op CD	0.940884820747521
3028	B100	C53	Control group	Post-op CD	0.9452707856598017
3029	B100	C56	Control group	Post-op CD	0.9460335621662853
3030	B100	C60	Control group	Post-op CD	0.9088482074752098
3031	B100	C62	Control group	Post-op CD	0.782608695652174
3032	B100	C64	Control group	Post-op CD	0.5947749809305873
3033	B100	C65	Control group	Post-op CD	0.8808161708619374
3034	B100	C69	Control group	Post-op CD	0.9086575133485889
3035	B100	C70	Control group	Post-op CD	0.9069412662090007
3036	B100	C74	Control group	Post-op CD	0.9727307398932112
3037	B100	C78	Control group	Post-op CD	0.9658657513348589
3038	B112	C1	Control group	Post-op CD	0.9593821510297483
3039	B112	C3	Control group	Post-op CD	0.9427917620137299
3040	B112	C7	Control group	Post-op CD	0.7299771167048055
3041	B112	C8	Control group	Post-op CD	0.938977879481312
3042	B112	C11	Control group	Post-op CD	0.9153318077803204
3043	B112	C15	Control group	Post-op CD	0.9342105263157895
3044	B112	C19	Control group	Post-op CD	0.7469488939740656
3045	B112	C22	Control group	Post-op CD	0.9376430205949656
3046	B112	C26	Control group	Post-op CD	0.9601449275362319
3047	B112	C28	Control group	Post-op CD	0.9443173150266971
3048	B112	C31	Control group	Post-op CD	0.9900839054157132
3049	B112	C35	Control group	Post-op CD	0.8632723112128147
3050	B112	C38	Control group	Post-op CD	0.9898932112890922
3051	B112	C40	Control group	Post-op CD	0.9426010678871091

3052 B112 C44 Control group Post-op CD
0.92372234935164
3053 B112 C47 Control group Post-op CD
0.9380244088482075
3054 B112 C48 Control group Post-op CD
0.677536231884058
3055 B112 C49 Control group Post-op CD
0.8274218154080855
3056 B112 C53 Control group Post-op CD
0.7694508009153318
3057 B112 C56 Control group Post-op CD
0.7145308924485125
3058 B112 C60 Control group Post-op CD
0.7759344012204424
3059 B112 C62 Control group Post-op CD
0.8056826849733029
3060 B112 C64 Control group Post-op CD
0.7726926010678871
3061 B112 C65 Control group Post-op CD
0.7433257055682685
3062 B112 C69 Control group Post-op CD
0.7410373760488177
3063 B112 C70 Control group Post-op CD
0.7662090007627765
3064 B112 C74 Control group Post-op CD
0.8178871090770404
3065 B112 C78 Control group Post-op CD
0.8934019832189168
3066 B115 C1 Control group Post-op CD
0.9706331045003814
3067 B115 C3 Control group Post-op CD
0.9361174675819984
3068 B115 C7 Control group Post-op CD
0.9288710907704043
3069 B115 C8 Control group Post-op CD
0.9654843630816171
3070 B115 C11 Control group Post-op CD
0.8741418764302059
3071 B115 C15 Control group Post-op CD
0.8966437833714722
3072 B115 C19 Control group Post-op CD
0.8647978642257819
3073 B115 C22 Control group Post-op CD
0.9086575133485889
3074 B115 C26 Control group Post-op CD
0.9586193745232647
3075 B115 C28 Control group Post-op CD
0.8592677345537757
3076 B115 C31 Control group Post-op CD
0.9679633867276888
3077 B115 C35 Control group Post-op CD
0.8897787948131197
3078 B115 C38 Control group Post-op CD
0.9527078565980168

3079 B115 C40 Control group Post-op CD
0.950419527078566
3080 B115 C44 Control group Post-op CD
0.9166666666666666
3081 B115 C47 Control group Post-op CD
0.906559877955759
3082 B115 C48 Control group Post-op CD
0.8640350877192983
3083 B115 C49 Control group Post-op CD
0.881769641495042
3084 B115 C53 Control group Post-op CD
0.8478260869565217
3085 B115 C56 Control group Post-op CD
0.8032036613272311
3086 B115 C60 Control group Post-op CD
0.8213196033562167
3087 B115 C62 Control group Post-op CD
0.9139969488939741
3088 B115 C64 Control group Post-op CD
0.8716628527841342
3089 B115 C65 Control group Post-op CD
0.8718535469107551
3090 B115 C69 Control group Post-op CD
0.8789092295957285
3091 B115 C70 Control group Post-op CD
0.8686117467581999
3092 B115 C74 Control group Post-op CD
0.9227688787185355
3093 B115 C78 Control group Post-op CD
0.8483981693363845
3094 I1 C1 Control group Post-op CD
0.9610983981693364
3095 I1 C3 Control group Post-op CD
0.8943554538520213
3096 I1 C7 Control group Post-op CD
0.9080854309687262
3097 I1 C8 Control group Post-op CD
0.9109458428680397
3098 I1 C11 Control group Post-op CD
0.9174294431731502
3099 I1 C15 Control group Post-op CD
0.8972158657513348
3100 I1 C19 Control group Post-op CD
0.8916857360793288
3101 I1 C22 Control group Post-op CD
0.933257055682685
3102 I1 C26 Control group Post-op CD
0.9477498093058734
3103 I1 C28 Control group Post-op CD
0.9088482074752098
3104 I1 C31 Control group Post-op CD
0.9792143401983219
3105 I1 C35 Control group Post-op CD
0.92372234935164

3106	I1	C38	Control group	Post-op CD	0.9734935163996948
3107	I1	C40	Control group	Post-op CD	0.9248665141113653
3108	I1	C44	Control group	Post-op CD	0.9265827612509535
3109	I1	C47	Control group	Post-op CD	0.9424103737604882
3110	I1	C48	Control group	Post-op CD	0.8659420289855072
3111	I1	C49	Control group	Post-op CD	0.9202898550724637
3112	I1	C53	Control group	Post-op CD	0.9021739130434783
3113	I1	C56	Control group	Post-op CD	0.8352402745995423
3114	I1	C60	Control group	Post-op CD	0.830091533180778
3115	I1	C62	Control group	Post-op CD	0.910373760488177
3116	I1	C64	Control group	Post-op CD	0.847254004576659
3117	I1	C65	Control group	Post-op CD	0.8945461479786423
3118	I1	C69	Control group	Post-op CD	0.8790999237223494
3119	I1	C70	Control group	Post-op CD	0.9033180778032036
3120	I1	C74	Control group	Post-op CD	0.9059877955758963
3121	I1	C78	Control group	Post-op CD	0.877765064836003
3122	I3	C1	Control group	Post-op CD	0.8827231121281465
3123	I3	C3	Control group	Post-op CD	0.940884820747521
3124	I3	C7	Control group	Post-op CD	0.9290617848970252
3125	I3	C8	Control group	Post-op CD	0.9830282227307399
3126	I3	C11	Control group	Post-op CD	0.9183829138062548
3127	I3	C15	Control group	Post-op CD	0.8787185354691075
3128	I3	C19	Control group	Post-op CD	0.9309687261632341
3129	I3	C22	Control group	Post-op CD	0.9557589626239512
3130	I3	C26	Control group	Post-op CD	0.8735697940503433
3131	I3	C28	Control group	Post-op CD	0.8342868039664378
3132	I3	C31	Control group	Post-op CD	0.8430587337909993

3133	I3	C35	Control group	Post-op CD	0.919908466819222
3134	I3	C38	Control group	Post-op CD	0.9660564454614798
3135	I3	C40	Control group	Post-op CD	0.7896643783371472
3136	I3	C44	Control group	Post-op CD	0.9610983981693364
3137	I3	C47	Control group	Post-op CD	0.9900839054157132
3138	I3	C48	Control group	Post-op CD	0.9139969488939741
3139	I3	C49	Control group	Post-op CD	0.8728070175438597
3140	I3	C53	Control group	Post-op CD	0.9338291380625476
3141	I3	C56	Control group	Post-op CD	0.8136918382913806
3142	I3	C60	Control group	Post-op CD	0.9050343249427918
3143	I3	C62	Control group	Post-op CD	0.8846300533943554
3144	I3	C64	Control group	Post-op CD	0.8941647597254004
3145	I3	C65	Control group	Post-op CD	0.9307780320366132
3146	I3	C69	Control group	Post-op CD	0.8165522501906941
3147	I3	C70	Control group	Post-op CD	0.9113272311212814
3148	I3	C74	Control group	Post-op CD	0.9380244088482075
3149	I3	C78	Control group	Post-op CD	0.7797482837528604
3150	I6	C1	Control group	Post-op CD	0.9696796338672768
3151	I6	C3	Control group	Post-op CD	0.9057971014492754
3152	I6	C7	Control group	Post-op CD	0.9403127383676583
3153	I6	C8	Control group	Post-op CD	0.9162852784134249
3154	I6	C11	Control group	Post-op CD	0.8482074752097636
3155	I6	C15	Control group	Post-op CD	0.7837528604118993
3156	I6	C19	Control group	Post-op CD	0.7990083905415714
3157	I6	C22	Control group	Post-op CD	0.9347826086956522
3158	I6	C26	Control group	Post-op CD	0.9244851258581236
3159	I6	C28	Control group	Post-op CD	0.8154080854309688

3160	I6	C31	Control group	Post-op CD	0.9323035850495804
3161	I6	C35	Control group	Post-op CD	0.8354309687261632
3162	I6	C38	Control group	Post-op CD	0.9548054919908466
3163	I6	C40	Control group	Post-op CD	0.8905415713196033
3164	I6	C44	Control group	Post-op CD	0.8432494279176201
3165	I6	C47	Control group	Post-op CD	0.860602593440122
3166	I6	C48	Control group	Post-op CD	0.8575514874141876
3167	I6	C49	Control group	Post-op CD	0.9073226544622426
3168	I6	C53	Control group	Post-op CD	0.7848970251716247
3169	I6	C56	Control group	Post-op CD	0.6983218916857361
3170	I6	C60	Control group	Post-op CD	0.7673531655225019
3171	I6	C62	Control group	Post-op CD	0.8432494279176201
3172	I6	C64	Control group	Post-op CD	0.8661327231121282
3173	I6	C65	Control group	Post-op CD	0.8790999237223494
3174	I6	C69	Control group	Post-op CD	0.86441647597254
3175	I6	C70	Control group	Post-op CD	0.879862700228833
3176	I6	C74	Control group	Post-op CD	0.9061784897025171
3177	I6	C78	Control group	Post-op CD	0.8356216628527842
3178	I8	C1	Control group	Post-op CD	0.9685354691075515
3179	I8	C3	Control group	Post-op CD	0.9071319603356217
3180	I8	C7	Control group	Post-op CD	0.9241037376048817
3181	I8	C8	Control group	Post-op CD	0.9206712433257056
3182	I8	C11	Control group	Post-op CD	0.9475591151792525
3183	I8	C15	Control group	Post-op CD	0.9448893974065599
3184	I8	C19	Control group	Post-op CD	0.9229595728451564
3185	I8	C22	Control group	Post-op CD	0.9475591151792525
3186	I8	C26	Control group	Post-op CD	0.9710144927536232

3187 I8 C28 Control group Post-op CD
0.9448893974065599
3188 I8 C31 Control group Post-op CD
0.9889397406559878
3189 I8 C35 Control group Post-op CD
0.9258199847444699
3190 I8 C38 Control group Post-op CD
0.9755911517925248
3191 I8 C40 Control group Post-op CD
0.952326468344775
3192 I8 C44 Control group Post-op CD
0.9570938215102975
3193 I8 C47 Control group Post-op CD
0.9563310450038138
3194 I8 C48 Control group Post-op CD
0.8545003813882532
3195 I8 C49 Control group Post-op CD
0.9090389016018307
3196 I8 C53 Control group Post-op CD
0.8857742181540809
3197 I8 C56 Control group Post-op CD
0.8430587337909993
3198 I8 C60 Control group Post-op CD
0.868230358504958
3199 I8 C62 Control group Post-op CD
0.9050343249427918
3200 I8 C64 Control group Post-op CD
0.8594584286803967
3201 I8 C65 Control group Post-op CD
0.8787185354691075
3202 I8 C69 Control group Post-op CD
0.8787185354691075
3203 I8 C70 Control group Post-op CD
0.8838672768878718
3204 I8 C74 Control group Post-op CD
0.9029366895499619
3205 I8 C78 Control group Post-op CD
0.8972158657513348
3206 I10 C1 Control group Post-op CD
0.9559496567505721
3207 I10 C3 Control group Post-op CD
0.9185736079328757
3208 I10 C7 Control group Post-op CD
0.910373760488177
3209 I10 C8 Control group Post-op CD
0.954042715484363
3210 I10 C11 Control group Post-op CD
0.8964530892448512
3211 I10 C15 Control group Post-op CD
0.8422959572845157
3212 I10 C19 Control group Post-op CD
0.8857742181540809
3213 I10 C22 Control group Post-op CD
0.9605263157894737

3214 I10 C26 Control group Post-op CD
0.9040808543096872
3215 I10 C28 Control group Post-op CD
0.9164759725400458
3216 I10 C31 Control group Post-op CD
0.9559496567505721
3217 I10 C35 Control group Post-op CD
0.8964530892448512
3218 I10 C38 Control group Post-op CD
0.9719679633867276
3219 I10 C40 Control group Post-op CD
0.8464912280701754
3220 I10 C44 Control group Post-op CD
0.918001525553013
3221 I10 C47 Control group Post-op CD
0.9597635392829901
3222 I10 C48 Control group Post-op CD
0.8937833714721587
3223 I10 C49 Control group Post-op CD
0.9286803966437833
3224 I10 C53 Control group Post-op CD
0.9210526315789473
3225 I10 C56 Control group Post-op CD
0.8676582761250954
3226 I10 C60 Control group Post-op CD
0.9088482074752098
3227 I10 C62 Control group Post-op CD
0.9088482074752098
3228 I10 C64 Control group Post-op CD
0.8943554538520213
3229 I10 C65 Control group Post-op CD
0.9147597254004577
3230 I10 C69 Control group Post-op CD
0.8665141113653699
3231 I10 C70 Control group Post-op CD
0.9067505720823799
3232 I10 C74 Control group Post-op CD
0.9242944317315027
3233 I10 C78 Control group Post-op CD
0.7675438596491229
3234 I11 C1 Control group Post-op CD
0.9450800915331807
3235 I11 C3 Control group Post-op CD
0.8920671243325705
3236 I11 C7 Control group Post-op CD
0.9864607170099161
3237 I11 C8 Control group Post-op CD
0.9155225019069413
3238 I11 C11 Control group Post-op CD
0.9715865751334859
3239 I11 C15 Control group Post-op CD
0.931350114416476
3240 I11 C19 Control group Post-op CD
0.950419527078566

3241 I11 C22 Control group Post-op CD
0.9452707856598017
3242 I11 C26 Control group Post-op CD
0.9414569031273837
3243 I11 C28 Control group Post-op CD
0.9427917620137299
3244 I11 C31 Control group Post-op CD
0.9677726926010679
3245 I11 C35 Control group Post-op CD
0.9858886346300534
3246 I11 C38 Control group Post-op CD
0.9565217391304348
3247 I11 C40 Control group Post-op CD
0.9002669717772692
3248 I11 C44 Control group Post-op CD
0.992372234935164
3249 I11 C47 Control group Post-op CD
0.9910373760488177
3250 I11 C48 Control group Post-op CD
0.9317315026697178
3251 I11 C49 Control group Post-op CD
0.8943554538520213
3252 I11 C53 Control group Post-op CD
0.9488939740655988
3253 I11 C56 Control group Post-op CD
0.9166666666666666
3254 I11 C60 Control group Post-op CD
0.8733790999237223
3255 I11 C62 Control group Post-op CD
0.9231502669717773
3256 I11 C64 Control group Post-op CD
0.9096109839816934
3257 I11 C65 Control group Post-op CD
0.9366895499618612
3258 I11 C69 Control group Post-op CD
0.8914950419527079
3259 I11 C70 Control group Post-op CD
0.9262013729977117
3260 I11 C74 Control group Post-op CD
0.9315408085430968
3261 I11 C78 Control group Post-op CD
0.9355453852021358
3262 I13 C1 Control group Post-op CD
0.9670099160945843
3263 I13 C3 Control group Post-op CD
0.9094202898550725
3264 I13 C7 Control group Post-op CD
0.8962623951182304
3265 I13 C8 Control group Post-op CD
0.9221967963386728
3266 I13 C11 Control group Post-op CD
0.8773836765827613
3267 I13 C15 Control group Post-op CD
0.8909229595728452

3268 I13 C19 Control group Post-op CD
0.8506864988558352
3269 I13 C22 Control group Post-op CD
0.956140350877193
3270 I13 C26 Control group Post-op CD
0.9170480549199085
3271 I13 C28 Control group Post-op CD
0.8661327231121282
3272 I13 C31 Control group Post-op CD
0.9710144927536232
3273 I13 C35 Control group Post-op CD
0.8522120518688024
3274 I13 C38 Control group Post-op CD
0.9677726926010679
3275 I13 C40 Control group Post-op CD
0.9181922196796338
3276 I13 C44 Control group Post-op CD
0.9118993135011442
3277 I13 C47 Control group Post-op CD
0.9254385964912281
3278 I13 C48 Control group Post-op CD
0.837909992372235
3279 I13 C49 Control group Post-op CD
0.8823417238749046
3280 I13 C53 Control group Post-op CD
0.8436308161708619
3281 I13 C56 Control group Post-op CD
0.8205568268497331
3282 I13 C60 Control group Post-op CD
0.8348588863463006
3283 I13 C62 Control group Post-op CD
0.8781464530892449
3284 I13 C64 Control group Post-op CD
0.8691838291380626
3285 I13 C65 Control group Post-op CD
0.8731884057971014
3286 I13 C69 Control group Post-op CD
0.8325705568268498
3287 I13 C70 Control group Post-op CD
0.8308543096872616
3288 I13 C74 Control group Post-op CD
0.8852021357742181
3289 I13 C78 Control group Post-op CD
0.8422959572845157
3290 I15 C1 Control group Post-op CD
0.8571700991609459
3291 I15 C3 Control group Post-op CD
0.8663234172387491
3292 I15 C7 Control group Post-op CD
0.9242944317315027
3293 I15 C8 Control group Post-op CD
0.933066361556064
3294 I15 C11 Control group Post-op CD
0.8325705568268498

3295 I15 C15 Control group Post-op CD
0.8190312738367659
3296 I15 C19 Control group Post-op CD
0.8278032036613272
3297 I15 C22 Control group Post-op CD
0.8499237223493517
3298 I15 C26 Control group Post-op CD
0.9448893974065599
3299 I15 C28 Control group Post-op CD
0.7982456140350878
3300 I15 C31 Control group Post-op CD
0.8287566742944318
3301 I15 C35 Control group Post-op CD
0.801487414187643
3302 I15 C38 Control group Post-op CD
0.8501144164759725
3303 I15 C40 Control group Post-op CD
0.9574752097635393
3304 I15 C44 Control group Post-op CD
0.7955758962623951
3305 I15 C47 Control group Post-op CD
0.8680396643783371
3306 I15 C48 Control group Post-op CD
0.9063691838291381
3307 I15 C49 Control group Post-op CD
0.9372616323417239
3308 I15 C53 Control group Post-op CD
0.858886346300534
3309 I15 C56 Control group Post-op CD
0.8310450038138826
3310 I15 C60 Control group Post-op CD
0.7116704805491991
3311 I15 C62 Control group Post-op CD
0.8888253241800153
3312 I15 C64 Control group Post-op CD
0.9126620900076278
3313 I15 C65 Control group Post-op CD
0.8155987795575896
3314 I15 C69 Control group Post-op CD
0.9155225019069413
3315 I15 C70 Control group Post-op CD
0.9500381388253242
3316 I15 C74 Control group Post-op CD
0.8485888634630053
3317 I15 C78 Control group Post-op CD
0.7473302822273074
3318 I17 C1 Control group Post-op CD
0.9622425629290617
3319 I17 C3 Control group Post-op CD
0.9469870327993898
3320 I17 C7 Control group Post-op CD
0.7377955758962624
3321 I17 C8 Control group Post-op CD
0.9715865751334859

3322	I17	C11	Control group	Post-op CD
0.9795957284515637
3323	I17	C15	Control group	Post-op CD
0.9660564454614798
3324	I17	C19	Control group	Post-op CD
0.7530511060259344
3325	I17	C22	Control group	Post-op CD
0.971205186880244
3326	I17	C26	Control group	Post-op CD
0.9593821510297483
3327	I17	C28	Control group	Post-op CD
0.9584286803966438
3328	I17	C31	Control group	Post-op CD
0.9944698703279939
3329	I17	C35	Control group	Post-op CD
0.8127383676582761
3330	I17	C38	Control group	Post-op CD
0.9815026697177727
3331	I17	C40	Control group	Post-op CD
0.9685354691075515
3332	I17	C44	Control group	Post-op CD
0.9822654462242563
3333	I17	C47	Control group	Post-op CD
0.971395881006865
3334	I17	C48	Control group	Post-op CD
0.6584668192219679
3335	I17	C49	Control group	Post-op CD
0.8470633104500381
3336	I17	C53	Control group	Post-op CD
0.7961479786422578
3337	I17	C56	Control group	Post-op CD
0.746186117467582
3338	I17	C60	Control group	Post-op CD
0.8043478260869565
3339	I17	C62	Control group	Post-op CD
0.8251334858886347
3340	I17	C64	Control group	Post-op CD
0.7974828375286042
3341	I17	C65	Control group	Post-op CD
0.7723112128146453
3342	I17	C69	Control group	Post-op CD
0.7677345537757437
3343	I17	C70	Control group	Post-op CD
0.7833714721586575
3344	I17	C74	Control group	Post-op CD
0.841723874904653
3345	I17	C78	Control group	Post-op CD
0.9149504195270786
3346	I18	C1	Control group	Post-op CD
0.9042715484363082
3347	I18	C3	Control group	Post-op CD
0.8905415713196033
3348	I18	C7	Control group	Post-op CD
0.8972158657513348

3349	I18	C8	Control group	Post-op CD
0.9406941266209001
3350	I18	C11	Control group	Post-op CD
0.8525934401220442
3351	I18	C15	Control group	Post-op CD
0.8299008390541571
3352	I18	C19	Control group	Post-op CD
0.8600305110602593
3353	I18	C22	Control group	Post-op CD
0.7580091533180778
3354	I18	C26	Control group	Post-op CD
0.8909229595728452
3355	I18	C28	Control group	Post-op CD
0.8852021357742181
3356	I18	C31	Control group	Post-op CD
0.9345919145690312
3357	I18	C35	Control group	Post-op CD
0.9200991609458429
3358	I18	C38	Control group	Post-op CD
0.9740655987795576
3359	I18	C40	Control group	Post-op CD
0.8627002288329519
3360	I18	C44	Control group	Post-op CD
0.9212433257055682
3361	I18	C47	Control group	Post-op CD
0.9311594202898551
3362	I18	C48	Control group	Post-op CD
0.8489702517162472
3363	I18	C49	Control group	Post-op CD
0.8537376048817696
3364	I18	C53	Control group	Post-op CD
0.8781464530892449
3365	I18	C56	Control group	Post-op CD
0.7822273073989321
3366	I18	C60	Control group	Post-op CD
0.818649885583524
3367	I18	C62	Control group	Post-op CD
0.8667048054919908
3368	I18	C64	Control group	Post-op CD
0.8741418764302059
3369	I18	C65	Control group	Post-op CD
0.813119755911518
3370	I18	C69	Control group	Post-op CD
0.8232265446224256
3371	I18	C70	Control group	Post-op CD
0.8220823798627003
3372	I18	C74	Control group	Post-op CD
0.8539282990083905
3373	I18	C78	Control group	Post-op CD
0.8136918382913806
3374	I19	C1	Control group	Post-op CD
0.9895118230358505
3375	I19	C3	Control group	Post-op CD
0.9406941266209001

3376 I19 C7 Control group Post-op CD
0.7692601067887109
3377 I19 C8 Control group Post-op CD
0.9719679633867276
3378 I19 C11 Control group Post-op CD
0.9490846681922197
3379 I19 C15 Control group Post-op CD
0.9353546910755148
3380 I19 C19 Control group Post-op CD
0.7688787185354691
3381 I19 C22 Control group Post-op CD
0.9641495041952708
3382 I19 C26 Control group Post-op CD
0.9818840579710145
3383 I19 C28 Control group Post-op CD
0.933257055682685
3384 I19 C31 Control group Post-op CD
0.9816933638443935
3385 I19 C35 Control group Post-op CD
0.8688024408848207
3386 I19 C38 Control group Post-op CD
0.9908466819221968
3387 I19 C40 Control group Post-op CD
0.9836003051106026
3388 I19 C44 Control group Post-op CD
0.9488939740655988
3389 I19 C47 Control group Post-op CD
0.9036994660564455
3390 I19 C48 Control group Post-op CD
0.3489702517162471
3391 I19 C49 Control group Post-op CD
0.6062166285278413
3392 I19 C53 Control group Post-op CD
0.4973302822273074
3393 I19 C56 Control group Post-op CD
0.4334477498093059
3394 I19 C60 Control group Post-op CD
0.587909992372235
3395 I19 C62 Control group Post-op CD
0.5734172387490465
3396 I19 C64 Control group Post-op CD
0.4977116704805492
3397 I19 C65 Control group Post-op CD
0.4342105263157895
3398 I19 C69 Control group Post-op CD
0.4652936689549962
3399 I19 C70 Control group Post-op CD
0.4687261632341724
3400 I19 C74 Control group Post-op CD
0.6254767353165522
3401 I19 C78 Control group Post-op CD
0.9094202898550725
3402 I22 C1 Control group Post-op CD
0.8442028985507246

3403 I22 C3 Control group Post-op CD
0.8220823798627003
3404 I22 C7 Control group Post-op CD
0.9334477498093059
3405 I22 C8 Control group Post-op CD
0.940884820747521
3406 I22 C11 Control group Post-op CD
0.9353546910755148
3407 I22 C15 Control group Post-op CD
0.9126620900076278
3408 I22 C19 Control group Post-op CD
0.9010297482837528
3409 I22 C22 Control group Post-op CD
0.8098779557589626
3410 I22 C26 Control group Post-op CD
0.9549961861174676
3411 I22 C28 Control group Post-op CD
0.9042715484363082
3412 I22 C31 Control group Post-op CD
0.9469870327993898
3413 I22 C35 Control group Post-op CD
0.940884820747521
3414 I22 C38 Control group Post-op CD
0.9628146453089245
3415 I22 C40 Control group Post-op CD
0.9212433257055682
3416 I22 C44 Control group Post-op CD
0.9530892448512586
3417 I22 C47 Control group Post-op CD
0.952326468344775
3418 I22 C48 Control group Post-op CD
0.8329519450800915
3419 I22 C49 Control group Post-op CD
0.8823417238749046
3420 I22 C53 Control group Post-op CD
0.8764302059496567
3421 I22 C56 Control group Post-op CD
0.8047292143401983
3422 I22 C60 Control group Post-op CD
0.7663996948893974
3423 I22 C62 Control group Post-op CD
0.8686117467581999
3424 I22 C64 Control group Post-op CD
0.8623188405797102
3425 I22 C65 Control group Post-op CD
0.7673531655225019
3426 I22 C69 Control group Post-op CD
0.855072463768116
3427 I22 C70 Control group Post-op CD
0.8686117467581999
3428 I22 C74 Control group Post-op CD
0.7965293668954996
3429 I22 C78 Control group Post-op CD
0.8901601830663616

3430 I23 C1 Control group Post-op CD
0.9588100686498856
3431 I23 C3 Control group Post-op CD
0.8924485125858124
3432 I23 C7 Control group Post-op CD
0.8995041952707856
3433 I23 C8 Control group Post-op CD
0.933257055682685
3434 I23 C11 Control group Post-op CD
0.8604118993135011
3435 I23 C15 Control group Post-op CD
0.8649885583524027
3436 I23 C19 Control group Post-op CD
0.8382913806254767
3437 I23 C22 Control group Post-op CD
0.9448893974065599
3438 I23 C26 Control group Post-op CD
0.8968344774980931
3439 I23 C28 Control group Post-op CD
0.855072463768116
3440 I23 C31 Control group Post-op CD
0.958047292143402
3441 I23 C35 Control group Post-op CD
0.8718535469107551
3442 I23 C38 Control group Post-op CD
0.975209763539283
3443 I23 C40 Control group Post-op CD
0.9344012204424104
3444 I23 C44 Control group Post-op CD
0.8935926773455377
3445 I23 C47 Control group Post-op CD
0.9115179252479023
3446 I23 C48 Control group Post-op CD
0.8464912280701754
3447 I23 C49 Control group Post-op CD
0.92372234935164
3448 I23 C53 Control group Post-op CD
0.8451563691838292
3449 I23 C56 Control group Post-op CD
0.8199847444698704
3450 I23 C60 Control group Post-op CD
0.8360030511060259
3451 I23 C62 Control group Post-op CD
0.912090007627765
3452 I23 C64 Control group Post-op CD
0.8514492753623188
3453 I23 C65 Control group Post-op CD
0.9056064073226545
3454 I23 C69 Control group Post-op CD
0.8546910755148741
3455 I23 C70 Control group Post-op CD
0.9071319603356217
3456 I23 C74 Control group Post-op CD
0.9172387490465294

3457 I23 C78 Control group Post-op CD
0.8258962623951183
3458 I24 C1 Control group Post-op CD
0.9931350114416476
3459 I24 C3 Control group Post-op CD
0.9691075514874142
3460 I24 C7 Control group Post-op CD
0.5108695652173914
3461 I24 C8 Control group Post-op CD
0.9689168573607932
3462 I24 C11 Control group Post-op CD
0.9229595728451564
3463 I24 C15 Control group Post-op CD
0.9220061022120518
3464 I24 C19 Control group Post-op CD
0.5921052631578947
3465 I24 C22 Control group Post-op CD
0.9588100686498856
3466 I24 C26 Control group Post-op CD
0.9679633867276888
3467 I24 C28 Control group Post-op CD
0.9296338672768879
3468 I24 C31 Control group Post-op CD
0.9876048817696415
3469 I24 C35 Control group Post-op CD
0.7278794813119756
3470 I24 C38 Control group Post-op CD
0.9959954233409611
3471 I24 C40 Control group Post-op CD
0.9345919145690312
3472 I24 C44 Control group Post-op CD
0.9242944317315027
3473 I24 C47 Control group Post-op CD
0.9307780320366132
3474 I24 C48 Control group Post-op CD
0.6128909229595728
3475 I24 C49 Control group Post-op CD
0.8487795575896262
3476 I24 C53 Control group Post-op CD
0.7715484363081617
3477 I24 C56 Control group Post-op CD
0.7545766590389016
3478 I24 C60 Control group Post-op CD
0.8056826849733029
3479 I24 C62 Control group Post-op CD
0.8142639206712433
3480 I24 C64 Control group Post-op CD
0.7902364607170099
3481 I24 C65 Control group Post-op CD
0.7704042715484363
3482 I24 C69 Control group Post-op CD
0.7561022120518688
3483 I24 C70 Control group Post-op CD
0.7852784134248665

3484	I24	C74	Control group	Post-op CD	0.8360030511060259
3485	I24	C78	Control group	Post-op CD	0.8705186880244088
3486	I25	C1	Control group	Post-op CD	0.952326468344775
3487	I25	C3	Control group	Post-op CD	0.9422196796338673
3488	I25	C7	Control group	Post-op CD	0.9044622425629291
3489	I25	C8	Control group	Post-op CD	0.9534706331045004
3490	I25	C11	Control group	Post-op CD	0.8794813119755912
3491	I25	C15	Control group	Post-op CD	0.8546910755148741
3492	I25	C19	Control group	Post-op CD	0.8638443935926774
3493	I25	C22	Control group	Post-op CD	0.9527078565980168
3494	I25	C26	Control group	Post-op CD	0.9172387490465294
3495	I25	C28	Control group	Post-op CD	0.8800533943554538
3496	I25	C31	Control group	Post-op CD	0.971395881006865
3497	I25	C35	Control group	Post-op CD	0.9193363844393593
3498	I25	C38	Control group	Post-op CD	0.9742562929061785
3499	I25	C40	Control group	Post-op CD	0.8651792524790236
3500	I25	C44	Control group	Post-op CD	0.9092295957284515
3501	I25	C47	Control group	Post-op CD	0.9336384439359268
3502	I25	C48	Control group	Post-op CD	0.8382913806254767
3503	I25	C49	Control group	Post-op CD	0.839626239511823
3504	I25	C53	Control group	Post-op CD	0.8543096872616324
3505	I25	C56	Control group	Post-op CD	0.7929061784897025
3506	I25	C60	Control group	Post-op CD	0.843440122044241
3507	I25	C62	Control group	Post-op CD	0.8909229595728452
3508	I25	C64	Control group	Post-op CD	0.8546910755148741
3509	I25	C65	Control group	Post-op CD	0.8598398169336384
3510	I25	C69	Control group	Post-op CD	0.8024408848207475

3511	I25	C70	Control group	Post-op CD	0.8522120518688024
3512	I25	C74	Control group	Post-op CD	0.8981693363844394
3513	I25	C78	Control group	Post-op CD	0.7887109077040427
3514	I26	C1	Control group	Post-op CD	0.9387871853546911
3515	I26	C3	Control group	Post-op CD	0.9471777269260107
3516	I26	C7	Control group	Post-op CD	0.8617467581998475
3517	I26	C8	Control group	Post-op CD	0.9294431731502669
3518	I26	C11	Control group	Post-op CD	0.8335240274599542
3519	I26	C15	Control group	Post-op CD	0.8209382151029748
3520	I26	C19	Control group	Post-op CD	0.8266590389016019
3521	I26	C22	Control group	Post-op CD	0.948512585812357
3522	I26	C26	Control group	Post-op CD	0.8972158657513348
3523	I26	C28	Control group	Post-op CD	0.8007246376811594
3524	I26	C31	Control group	Post-op CD	0.9405034324942791
3525	I26	C35	Control group	Post-op CD	0.8037757437070938
3526	I26	C38	Control group	Post-op CD	0.9797864225781846
3527	I26	C40	Control group	Post-op CD	0.872234935163997
3528	I26	C44	Control group	Post-op CD	0.8522120518688024
3529	I26	C47	Control group	Post-op CD	0.8676582761250954
3530	I26	C48	Control group	Post-op CD	0.8808161708619374
3531	I26	C49	Control group	Post-op CD	0.9528985507246377
3532	I26	C53	Control group	Post-op CD	0.8501144164759725
3533	I26	C56	Control group	Post-op CD	0.811022120518688
3534	I26	C60	Control group	Post-op CD	0.8546910755148741
3535	I26	C62	Control group	Post-op CD	0.929252479023646
3536	I26	C64	Control group	Post-op CD	0.8811975591151793
3537	I26	C65	Control group	Post-op CD	0.9248665141113653

3538 I26 C69 Control group Post-op CD
0.8733790999237223
3539 I26 C70 Control group Post-op CD
0.9176201372997712
3540 I26 C74 Control group Post-op CD
0.9357360793287567
3541 I26 C78 Control group Post-op CD
0.7604881769641495
3542 I27 C1 Control group Post-op CD
0.9099923722349351
3543 I27 C3 Control group Post-op CD
0.9300152555301296
3544 I27 C7 Control group Post-op CD
0.8972158657513348
3545 I27 C8 Control group Post-op CD
0.9412662090007627
3546 I27 C11 Control group Post-op CD
0.8197940503432495
3547 I27 C15 Control group Post-op CD
0.7389397406559878
3548 I27 C19 Control group Post-op CD
0.8119755911517925
3549 I27 C22 Control group Post-op CD
0.8659420289855072
3550 I27 C26 Control group Post-op CD
0.7990083905415714
3551 I27 C28 Control group Post-op CD
0.8232265446224256
3552 I27 C31 Control group Post-op CD
0.885202135774218l
3553 I27 C35 Control group Post-op CD
0.8449656750572082
3554 I27 C38 Control group Post-op CD
0.9738749046529367
3555 I27 C40 Control group Post-op CD
0.8335240274599542
3556 I27 C44 Control group Post-op CD
0.8531655225019069
3557 I27 C47 Control group Post-op CD
0.8974065598779558
3558 I27 C48 Control group Post-op CD
0.835812356979405
3559 I27 C49 Control group Post-op CD
0.9244851258581236
3560 I27 C53 Control group Post-op CD
0.8527841342486652
3561 I27 C56 Control group Post-op CD
0.721205186880244
3562 I27 C60 Control group Post-op CD
0.8413424866514111
3563 I27 C62 Control group Post-op CD
0.8579328756674295
3564 I27 C64 Control group Post-op CD
0.8869183829138062

3565 I27 C65 Control group Post-op CD
0.8972158657513348
3566 I27 C69 Control group Post-op CD
0.8733790999237223
3567 I27 C70 Control group Post-op CD
0.86441647597254
3568 I27 C74 Control group Post-op CD
0.9170480549199085
3569 I27 C78 Control group Post-op CD
0.6611365369946606
3570 I28 C1 Control group Post-op CD
0.8381006864988558
3571 I28 C3 Control group Post-op CD
0.8951182303585049
3572 I28 C7 Control group Post-op CD
0.9570938215102975
3573 I28 C8 Control group Post-op CD
0.8954996186117468
3574 I28 C11 Control group Post-op CD
0.8483981693363845
3575 I28 C15 Control group Post-op CD
0.9101830663615561
3576 I28 C19 Control group Post-op CD
0.9006483600305111
3577 I28 C22 Control group Post-op CD
0.9000762776506483
3578 I28 C26 Control group Post-op CD
0.8573607932875668
3579 I28 C28 Control group Post-op CD
0.9412662090007627
3580 I28 C31 Control group Post-op CD
0.9628146453089245
3581 I28 C35 Control group Post-op CD
0.9815026697177727
3582 I28 C38 Control group Post-op CD
0.9418382913806255
3583 I28 C40 Control group Post-op CD
0.6151792524790236
3584 I28 C44 Control group Post-op CD
0.9384057971014492
3585 I28 C47 Control group Post-op CD
0.982837528604119
3586 I28 C48 Control group Post-op CD
0.9429824561403509
3587 I28 C49 Control group Post-op CD
0.7065217391304348
3588 I28 C53 Control group Post-op CD
0.9528985507246377
3589 I28 C56 Control group Post-op CD
0.8993135011441648
3590 I28 C60 Control group Post-op CD
0.8630816170861938
3591 I28 C62 Control group Post-op CD
0.9290617848970252

3592 I28 C64 Control group Post-op CD
0.916094584286804
3593 I28 C65 Control group Post-op CD
0.9147597254004577
3594 I28 C69 Control group Post-op CD
0.8226544622425629
3595 I28 C70 Control group Post-op CD
0.8703279938977879
3596 I28 C74 Control group Post-op CD
0.9241037376048817
3597 I28 C78 Control group Post-op CD
0.8239893211289092
3598 I29 C1 Control group Post-op CD
0.9570938215102975
3599 I29 C3 Control group Post-op CD
0.952326468344775
3600 I29 C7 Control group Post-op CD
0.9406941266209001
3601 I29 C8 Control group Post-op CD
0.9494660564454614
3602 I29 C11 Control group Post-op CD
0.912090007627765
3603 I29 C15 Control group Post-op CD
0.9282990083905416
3604 I29 C19 Control group Post-op CD
0.8979786422578184
3605 I29 C22 Control group Post-op CD
0.9607170099160945
3606 I29 C26 Control group Post-op CD
0.9727307398932112
3607 I29 C28 Control group Post-op CD
0.8709000762776506
3608 I29 C31 Control group Post-op CD
0.9622425629290617
3609 I29 C35 Control group Post-op CD
0.9054157131960335
3610 I29 C38 Control group Post-op CD
0.988558352402746
3611 I29 C40 Control group Post-op CD
0.9221967963386728
3612 I29 C44 Control group Post-op CD
0.9231502669717773
3613 I29 C47 Control group Post-op CD
0.9511823035850496
3614 I29 C48 Control group Post-op CD
0.8954996186117468
3615 I29 C49 Control group Post-op CD
0.9361174675819984
3616 I29 C53 Control group Post-op CD
0.9231502669717773
3617 I29 C56 Control group Post-op CD
0.8728070175438597
3618 I29 C60 Control group Post-op CD
0.9246758199847445

3619	I29	C62	Control group	Post-op CD	0.9214340198321892
3620	I29	C64	Control group	Post-op CD	0.8981693363844394
3621	I29	C65	Control group	Post-op CD	0.9012204424103738
3622	I29	C69	Control group	Post-op CD	0.8804347826086957
3623	I29	C70	Control group	Post-op CD	0.9098016781083142
3624	I29	C74	Control group	Post-op CD	0.9302059496567505
3625	I29	C78	Control group	Post-op CD	0.8409610983981693
3626	I30	C1	Control group	Post-op CD	0.9218154080854309
3627	I30	C3	Control group	Post-op CD	0.9576659038901602
3628	I30	C7	Control group	Post-op CD	0.8920671243325705
3629	I30	C8	Control group	Post-op CD	0.9378337147215866
3630	I30	C11	Control group	Post-op CD	0.7818459191456903
3631	I30	C15	Control group	Post-op CD	0.7648741418764302
3632	I30	C19	Control group	Post-op CD	0.7688787185354691
3633	I30	C22	Control group	Post-op CD	0.9450800915331807
3634	I30	C26	Control group	Post-op CD	0.9191456903127384
3635	I30	C28	Control group	Post-op CD	0.7515255530129672
3636	I30	C31	Control group	Post-op CD	0.9220061022120518
3637	I30	C35	Control group	Post-op CD	0.8249427917620137
3638	I30	C38	Control group	Post-op CD	0.9170480549199085
3639	I30	C40	Control group	Post-op CD	0.8260869565217391
3640	I30	C44	Control group	Post-op CD	0.6891685736079328
3641	I30	C47	Control group	Post-op CD	0.8436308161708619
3642	I30	C48	Control group	Post-op CD	0.8979786422578184
3643	I30	C49	Control group	Post-op CD	0.9317315026697178
3644	I30	C53	Control group	Post-op CD	0.8171243325705568
3645	I30	C56	Control group	Post-op CD	0.6939359267734554

3646   I30   C60   Control group   Post-op CD
0.7864225781845919
3647   I30   C62   Control group   Post-op CD
0.8872997711670481
3648   I30   C64   Control group   Post-op CD
0.8939740655987796
3649   I30   C65   Control group   Post-op CD
0.9124713958810069
3650   I30   C69   Control group   Post-op CD
0.9014111365369947
3651   I30   C70   Control group   Post-op CD
0.9427917620137299
3652   I30   C74   Control group   Post-op CD
0.9534706331045004
3653   I30   C78   Control group   Post-op CD
0.7803203661327232
3654   I31   C1   Control group   Post-op CD
0.9633867276887872
3655   I31   C3   Control group   Post-op CD
0.9544241037376049
3656   I31   C7   Control group   Post-op CD
0.9740655987795576
3657   I31   C8   Control group   Post-op CD
0.9658657513348589
3658   I31   C11   Control group   Post-op CD
0.9591914569031273
3659   I31   C15   Control group   Post-op CD
0.9557589626239512
3660   I31   C19   Control group   Post-op CD
0.915903890160183
3661   I31   C22   Control group   Post-op CD
0.9736842105263158
3662   I31   C26   Control group   Post-op CD
0.9570938215102975
3663   I31   C28   Control group   Post-op CD
0.9239130434782609
3664   I31   C31   Control group   Post-op CD
0.9843630816170862
3665   I31   C35   Control group   Post-op CD
0.9755911517925248
3666   I31   C38   Control group   Post-op CD
0.9820747520976354
3667   I31   C40   Control group   Post-op CD
0.9044622425629291
3668   I31   C44   Control group   Post-op CD
0.9950419527078566
3669   I31   C47   Control group   Post-op CD
0.9902745995423341
3670   I31   C48   Control group   Post-op CD
0.9336384439359268
3671   I31   C49   Control group   Post-op CD
0.9433638443935927
3672   I31   C53   Control group   Post-op CD
0.943554538520213

3673	I31	C56	Control group	Post-op CD
0.8951182303585049
3674	I31	C60	Control group	Post-op CD
0.9107551487414187
3675	I31	C62	Control group	Post-op CD
0.9374523264683448
3676	I31	C64	Control group	Post-op CD
0.9397406559877955
3677	I31	C65	Control group	Post-op CD
0.9374523264683448
3678	I31	C69	Control group	Post-op CD
0.9258199847444699
3679	I31	C70	Control group	Post-op CD
0.9324942791762014
3680	I31	C74	Control group	Post-op CD
0.9418382913806255
3681	I31	C78	Control group	Post-op CD
0.9174294431731502
3682	I32	C1	Control group	Post-op CD
0.9691075514874142
3683	I32	C3	Control group	Post-op CD
0.9725400457665904
3684	I32	C7	Control group	Post-op CD
0.9431731502669718
3685	I32	C8	Control group	Post-op CD
0.9681540808543097
3686	I32	C11	Control group	Post-op CD
0.8958810068649885
3687	I32	C15	Control group	Post-op CD
0.9347826086956522
3688	I32	C19	Control group	Post-op CD
0.9210526315789473
3689	I32	C22	Control group	Post-op CD
0.9754004576659039
3690	I32	C26	Control group	Post-op CD
0.9559496567505721
3691	I32	C28	Control group	Post-op CD
0.9445080091533181
3692	I32	C31	Control group	Post-op CD
0.9685354691075515
3693	I32	C35	Control group	Post-op CD
0.9385964912280702
3694	I32	C38	Control group	Post-op CD
0.9971395881006865
3695	I32	C40	Control group	Post-op CD
0.9046529366895499
3696	I32	C44	Control group	Post-op CD
0.9532799389778794
3697	I32	C47	Control group	Post-op CD
0.9952326468344775
3698	I32	C48	Control group	Post-op CD
0.9454614797864226
3699	I32	C49	Control group	Post-op CD
0.9441266209000763

3700 I32 C53 Control group Post-op CD
0.9635774218154081
3701 I32 C56 Control group Post-op CD
0.910373760488177
3702 I32 C60 Control group Post-op CD
0.9553775743707094
3703 I32 C62 Control group Post-op CD
0.9315408085430968
3704 I32 C64 Control group Post-op CD
0.954042715484363
3705 I32 C65 Control group Post-op CD
0.9498474446987033
3706 I32 C69 Control group Post-op CD
0.9029366895499619
3707 I32 C70 Control group Post-op CD
0.9277269260106789
3708 I32 C74 Control group Post-op CD
0.9557589626239512
3709 I32 C78 Control group Post-op CD
0.8306636155606407
3710 I33 C1 Control group Post-op CD
0.9744469870327994
3711 I33 C3 Control group Post-op CD
0.9464149504195271
3712 I33 C7 Control group Post-op CD
0.5396643783371472
3713 I33 C8 Control group Post-op CD
0.9679633867276888
3714 I33 C11 Control group Post-op CD
0.9191456903127384
3715 I33 C15 Control group Post-op CD
0.8625095347063311
3716 I33 C19 Control group Post-op CD
0.623951182303585
3717 I33 C22 Control group Post-op CD
0.9702517162471396
3718 I33 C26 Control group Post-op CD
0.9326849733028223
3719 I33 C28 Control group Post-op CD
0.9323035850495804
3720 I33 C31 Control group Post-op CD
0.9855072463768116
3721 I33 C35 Control group Post-op CD
0.7187261632341724
3722 I33 C38 Control group Post-op CD
0.9809305873379099
3723 I33 C40 Control group Post-op CD
0.9473684210526315
3724 I33 C44 Control group Post-op CD
0.9118993135011442
3725 I33 C47 Control group Post-op CD
0.9252479023646072
3726 I33 C48 Control group Post-op CD
0.5692219679633868

3727	I33	C49	Control group	Post-op CD
0.7915713196033562
3728	I33	C53	Control group	Post-op CD
0.7374141876430206
3729	I33	C56	Control group	Post-op CD
0.6952707856598017
3730	I33	C60	Control group	Post-op CD
0.7561022120518688
3731	I33	C62	Control group	Post-op CD
0.7835621662852784
3732	I33	C64	Control group	Post-op CD
0.7520976353928299
3733	I33	C65	Control group	Post-op CD
0.7402745995423341
3734	I33	C69	Control group	Post-op CD
0.7049961861174676
3735	I33	C70	Control group	Post-op CD
0.7374141876430206
3736	I33	C74	Control group	Post-op CD
0.7999618611746758
3737	I33	C78	Control group	Post-op CD
0.811022120518688
3738	I34	C1	Control group	Post-op CD
0.9605263157894737
3739	I34	C3	Control group	Post-op CD
0.9294431731502669
3740	I34	C7	Control group	Post-op CD
0.7402745995423341
3741	I34	C8	Control group	Post-op CD
0.9441266209000763
3742	I34	C11	Control group	Post-op CD
0.795766590389016
3743	I34	C15	Control group	Post-op CD
0.8295194508009154
3744	I34	C19	Control group	Post-op CD
0.6781083142639207
3745	I34	C22	Control group	Post-op CD
0.9651029748283753
3746	I34	C26	Control group	Post-op CD
0.9212433257055682
3747	I34	C28	Control group	Post-op CD
0.8800533943554538
3748	I34	C31	Control group	Post-op CD
0.9803585049580473
3749	I34	C35	Control group	Post-op CD
0.6924103737604882
3750	I34	C38	Control group	Post-op CD
0.9755911517925248
3751	I34	C40	Control group	Post-op CD
0.9361174675819984
3752	I34	C44	Control group	Post-op CD
0.8327612509534706
3753	I34	C47	Control group	Post-op CD
0.8331426392067124

3754	I34	C48	Control group	Post-op CD
0.7395118230358505
3755	I34	C49	Control group	Post-op CD
0.8562166285278413
3756	I34	C53	Control group	Post-op CD
0.7276887871853547
3757	I34	C56	Control group	Post-op CD
0.7276887871853547
3758	I34	C60	Control group	Post-op CD
0.7273073989321129
3759	I34	C62	Control group	Post-op CD
0.8436308161708619
3760	I34	C64	Control group	Post-op CD
0.7816552250190694
3761	I34	C65	Control group	Post-op CD
0.8463005339435545
3762	I34	C69	Control group	Post-op CD
0.8310450038138826
3763	I34	C70	Control group	Post-op CD
0.8407704042715485
3764	I34	C74	Control group	Post-op CD
0.8499237223493517
3765	I34	C78	Control group	Post-op CD
0.8281845919145691
3766	I35	C1	Control group	Post-op CD
0.9565217391304348
3767	I35	C3	Control group	Post-op CD
0.8985507246376812
3768	I35	C7	Control group	Post-op CD
0.9437452326468345
3769	I35	C8	Control group	Post-op CD
0.8800533943554538
3770	I35	C11	Control group	Post-op CD
0.9071319603356217
3771	I35	C15	Control group	Post-op CD
0.9132341723874905
3772	I35	C19	Control group	Post-op CD
0.873951182303585
3773	I35	C22	Control group	Post-op CD
0.9374523264683448
3774	I35	C26	Control group	Post-op CD
0.8743325705568269
3775	I35	C28	Control group	Post-op CD
0.940884820747521
3776	I35	C31	Control group	Post-op CD
0.9614797864225781
3777	I35	C35	Control group	Post-op CD
0.9380244088482075
3778	I35	C38	Control group	Post-op CD
0.9689168573607932
3779	I35	C40	Control group	Post-op CD
0.8373379099923722
3780	I35	C44	Control group	Post-op CD
0.9527078565980168

3781 I35 C47 Control group Post-op CD
0.9826468344774981
3782 I35 C48 Control group Post-op CD
0.900839054157132
3783 I35 C49 Control group Post-op CD
0.8720442410373761
3784 I35 C53 Control group Post-op CD
0.9172387490465294
3785 I35 C56 Control group Post-op CD
0.8995041952707856
3786 I35 C60 Control group Post-op CD
0.9080854309687262
3787 I35 C62 Control group Post-op CD
0.9288710907704043
3788 I35 C64 Control group Post-op CD
0.9006483600305111
3789 I35 C65 Control group Post-op CD
0.9277269260106789
3790 I35 C69 Control group Post-op CD
0.8140732265446224
3791 I35 C70 Control group Post-op CD
0.8792906178489702
3792 I35 C74 Control group Post-op CD
0.9347826086956522
3793 I35 C78 Control group Post-op CD
0.8030129672006102
3794 I36 C1 Control group Post-op CD
0.8196033562166285
3795 I36 C3 Control group Post-op CD
0.8995041952707856
3796 I36 C7 Control group Post-op CD
0.9971395881006865
3797 I36 C8 Control group Post-op CD
0.9525171624713958
3798 I36 C11 Control group Post-op CD
0.9925629290617849
3799 I36 C15 Control group Post-op CD
0.982837528604119
3800 I36 C19 Control group Post-op CD
0.9858886346300534
3801 I36 C22 Control group Post-op CD
0.7654462242562929
3802 I36 C26 Control group Post-op CD
0.9860793287566743
3803 I36 C28 Control group Post-op CD
0.982837528604119
3804 I36 C31 Control group Post-op CD
0.9590007627765065
3805 I36 C35 Control group Post-op CD
0.9918001525553013
3806 I36 C38 Control group Post-op CD
0.975209763539283
3807 I36 C40 Control group Post-op CD
0.9652936689549961

3808	I36	C44	Control group	Post-op CD
0.9855072463768116
3809	I36	C47	Control group	Post-op CD
0.9954233409610984
3810	I36	C48	Control group	Post-op CD
0.9778794813119756
3811	I36	C49	Control group	Post-op CD
0.9670099160945843
3812	I36	C53	Control group	Post-op CD
0.9876048817696415
3813	I36	C56	Control group	Post-op CD
0.9788329519450801
3814	I36	C60	Control group	Post-op CD
0.8693745232646835
3815	I36	C62	Control group	Post-op CD
0.8464912280701754
3816	I36	C64	Control group	Post-op CD
0.9063691838291381
3817	I36	C65	Control group	Post-op CD
0.7986270022883295
3818	I36	C69	Control group	Post-op CD
0.9822654462242563
3819	I36	C70	Control group	Post-op CD
0.9326849733028223
3820	I36	C74	Control group	Post-op CD
0.8390541571319603
3821	I36	C78	Control group	Post-op CD
0.9963768115942029
3822	I37	C1	Control group	Post-op CD
0.8154080854309688
3823	I37	C3	Control group	Post-op CD
0.631769641495042
3824	I37	C7	Control group	Post-op CD
0.9639588100686499
3825	I37	C8	Control group	Post-op CD
0.8411517925247902
3826	I37	C11	Control group	Post-op CD
0.9530892448512586
3827	I37	C15	Control group	Post-op CD
0.965675057208238
3828	I37	C19	Control group	Post-op CD
0.93707093821510З
3829	I37	C22	Control group	Post-op CD
0.7059496567505721
3830	I37	C26	Control group	Post-op CD
0.9666285278413425
3831	I37	C28	Control group	Post-op CD
0.9603356216628528
3832	I37	C31	Control group	Post-op CD
0.9740655987795576
3833	I37	C35	Control group	Post-op CD
0.9740655987795576
3834	I37	C38	Control group	Post-op CD
0.952326468344775

3835	I37	C40	Control group	Post-op CD	0.9098016781083142
3836	I37	C44	Control group	Post-op CD	0.9729214340198322
3837	I37	C47	Control group	Post-op CD	0.9826468344774981
3838	I37	C48	Control group	Post-op CD	0.9382151029748284
3839	I37	C49	Control group	Post-op CD	0.9395499618611747
3840	I37	C53	Control group	Post-op CD	0.9555682684973302
3841	I37	C56	Control group	Post-op CD	0.9427917620137299
3842	I37	C60	Control group	Post-op CD	0.8011060259344012
3843	I37	C62	Control group	Post-op CD	0.9412662090007627
3844	I37	C64	Control group	Post-op CD	0.9122807017543859
3845	I37	C65	Control group	Post-op CD	0.784324942791762
3846	I37	C69	Control group	Post-op CD	0.9344012204424104
3847	I37	C70	Control group	Post-op CD	0.9136155606407322
3848	I37	C74	Control group	Post-op CD	0.7993897787948131
3849	I37	C78	Control group	Post-op CD	0.9469870327993898
0	B5	B1	Idiopathic BAD	Idiopathic BAD	0.7803203661327232
1	B6	B1	Idiopathic BAD	Idiopathic BAD	0.8768115942028986
2	B6	B5	Idiopathic BAD	Idiopathic BAD	0.8716628527841342
3	B10	B1	Idiopathic BAD	Idiopathic BAD	0.6937452326468345
4	B10	B5	Idiopathic BAD	Idiopathic BAD	0.8733790999237223
5	B10	B6	Idiopathic BAD	Idiopathic BAD	0.9035087719298246
6	B17	B1	Idiopathic BAD	Idiopathic BAD	0.8411517925247902
7	B17	B5	Idiopathic BAD	Idiopathic BAD	0.8579328756674295
8	B17	B6	Idiopathic BAD	Idiopathic BAD	0.8611746758199847
9	B17	B10	Idiopathic BAD	Idiopathic BAD	0.763348588863463
10	B20	B1	Idiopathic BAD	Idiopathic BAD	0.9431731502669718
11	B20	B5	Idiopathic BAD	Idiopathic BAD	0.866514111365369

12	B20	B6	Idiopathic BAD	Idiopathic BAD	0.8667048054919908
13	B20	B10	Idiopathic BAD	Idiopathic BAD	0.9218154080854309
14	B20	B17	Idiopathic BAD	Idiopathic BAD	0.5783752860411899
15	B23	B1	Idiopathic BAD	Idiopathic BAD	0.8785278413424866
16	B23	B5	Idiopathic BAD	Idiopathic BAD	0.811022120518688
17	B23	B6	Idiopathic BAD	Idiopathic BAD	0.8443935926773455
18	B23	B10	Idiopathic BAD	Idiopathic BAD	0.8182684973302822
19	B23	B17	Idiopathic BAD	Idiopathic BAD	0.7803203661327232
20	B23	B20	Idiopathic BAD	Idiopathic BAD	0.8354309687261632
21	B31	B1	Idiopathic BAD	Idiopathic BAD	0.6914569031273837
22	B31	B5	Idiopathic BAD	Idiopathic BAD	0.7917620137299771
23	B31	B6	Idiopathic BAD	Idiopathic BAD	0.9098016781083142
24	B31	B10	Idiopathic BAD	Idiopathic BAD	0.6168954996186118
25	B31	B17	Idiopathic BAD	Idiopathic BAD	0.7185354691075515
26	B31	B20	Idiopathic BAD	Idiopathic BAD	0.9012204424103738
27	B31	B23	Idiopathic BAD	Idiopathic BAD	0.7227307398932112
28	B35	B1	Idiopathic BAD	Idiopathic BAD	0.8964530892448512
29	B35	B5	Idiopathic BAD	Idiopathic BAD	0.872234935163997
30	B35	B6	Idiopathic BAD	Idiopathic BAD	0.9416475972540046
31	B35	B10	Idiopathic BAD	Idiopathic BAD	0.9889397406559878
32	B35	B17	Idiopathic BAD	Idiopathic BAD	0.9763539282990084
33	B35	B20	Idiopathic BAD	Idiopathic BAD	0.9906559877955758
34	B35	B23	Idiopathic BAD	Idiopathic BAD	0.9443173150266971
35	B35	B31	Idiopathic BAD	Idiopathic BAD	0.9035087719298246
36	B39	B1	Idiopathic BAD	Idiopathic BAD	0.8762395118230358
37	B39	B5	Idiopathic BAD	Idiopathic BAD	0.8306636155606407
38	B39	B6	Idiopathic BAD	Idiopathic BAD	0.8581235697940504

39	B39	B10	Idiopathic BAD	Idiopathic BAD	0.9052250190694127
40	B39	B17	Idiopathic BAD	Idiopathic BAD	0.8531655225019069
41	B39	B20	Idiopathic BAD	Idiopathic BAD	0.8699466056445462
42	B39	B23	Idiopathic BAD	Idiopathic BAD	0.8062547673531655
43	B39	B31	Idiopathic BAD	Idiopathic BAD	0.8180778032036613
44	B39	B35	Idiopathic BAD	Idiopathic BAD	0.9260106788710908
45	B43	B1	Idiopathic BAD	Idiopathic BAD	0.9631960335621663
46	B43	B5	Idiopathic BAD	Idiopathic BAD	0.8787185354691075
47	B43	B6	Idiopathic BAD	Idiopathic BAD	0.778604118993135
48	B43	B10	Idiopathic BAD	Idiopathic BAD	0.9282990083905416
49	B43	B17	Idiopathic BAD	Idiopathic BAD	0.7778413424866514
50	B43	B20	Idiopathic BAD	Idiopathic BAD	0.6327231121281465
51	B43	B23	Idiopathic BAD	Idiopathic BAD	0.8960717009916095
52	B43	B31	Idiopathic BAD	Idiopathic BAD	0.9138062547673532
53	B43	B35	Idiopathic BAD	Idiopathic BAD	0.9786422578184591
54	B43	B39	Idiopathic BAD	Idiopathic BAD	0.9122807017543859
55	B47	B1	Idiopathic BAD	Idiopathic BAD	0.8960717009916095
56	B47	B5	Idiopathic BAD	Idiopathic BAD	0.8306636155606407
57	B47	B6	Idiopathic BAD	Idiopathic BAD	0.8926392067124332
58	B47	B10	Idiopathic BAD	Idiopathic BAD	0.8451563691838292
59	B47	B17	Idiopathic BAD	Idiopathic BAD	0.7974828375286042
60	B47	B20	Idiopathic BAD	Idiopathic BAD	0.8083524027459954
61	B47	B23	Idiopathic BAD	Idiopathic BAD	0.7808924485125858
62	B47	B31	Idiopathic BAD	Idiopathic BAD	0.8222730739893211
63	B47	B35	Idiopathic BAD	Idiopathic BAD	0.9845537757437071
64	B47	B39	Idiopathic BAD	Idiopathic BAD	0.8079710144927537
65	B47	B43	Idiopathic BAD	Idiopathic BAD	0.8903508771929824

66	B48	B1	Idiopathic BAD	Idiopathic BAD	0.7337909992372235
67	B48	B5	Idiopathic BAD	Idiopathic BAD	0.7353165522501907
68	B48	B6	Idiopathic BAD	Idiopathic BAD	0.9254385964912281
69	B48	B10	Idiopathic BAD	Idiopathic BAD	0.8192219679633868
70	B48	B17	Idiopathic BAD	Idiopathic BAD	0.8604118993135011
71	B48	B20	Idiopathic BAD	Idiopathic BAD	0.9393592677345538
72	B48	B23	Idiopathic BAD	Idiopathic BAD	0.929252479023646
73	B48	B31	Idiopathic BAD	Idiopathic BAD	0.715675057208238
74	B48	B35	Idiopathic BAD	Idiopathic BAD	0.5711289092295957
75	B48	B39	Idiopathic BAD	Idiopathic BAD	0.8541189931350115
76	B48	B43	Idiopathic BAD	Idiopathic BAD	0.9559496567505721
77	B48	B47	Idiopathic BAD	Idiopathic BAD	0.9162852784134249
78	B49	B1	Idiopathic BAD	Idiopathic BAD	0.8640350877192983
79	B49	B5	Idiopathic BAD	Idiopathic BAD	0.831998474446987
80	B49	B6	Idiopathic BAD	Idiopathic BAD	0.7793668954996186
81	B49	B10	Idiopathic BAD	Idiopathic BAD	0.8752860411899314
82	B49	B17	Idiopathic BAD	Idiopathic BAD	0.8636536994660564
83	B49	B20	Idiopathic BAD	Idiopathic BAD	0.8729977116704806
84	B49	B23	Idiopathic BAD	Idiopathic BAD	0.8689931350114416
85	B49	B31	Idiopathic BAD	Idiopathic BAD	0.8466819221967964
86	B49	B35	Idiopathic BAD	Idiopathic BAD	0.8251334858886347
87	B49	B39	Idiopathic BAD	Idiopathic BAD	0.8525934401220442
88	B49	B43	Idiopathic BAD	Idiopathic BAD	0.8251334858886347
89	B49	B47	Idiopathic BAD	Idiopathic BAD	0.8785278413424866
90	B49	B48	Idiopathic BAD	Idiopathic BAD	0.8062547673531655
91	B53	B1	Idiopathic BAD	Idiopathic BAD	0.8594584286803967
92	B53	B5	Idiopathic BAD	Idiopathic BAD	0.861556064073226

93	B53	B6	Idiopathic BAD	Idiopathic BAD	0.9443173150266971
94	B53	B10	Idiopathic BAD	Idiopathic BAD	0.8979786422578184
95	B53	B17	Idiopathic BAD	Idiopathic BAD	0.8524027459954233
96	B53	B20	Idiopathic BAD	Idiopathic BAD	0.8981693363844394
97	B53	B23	Idiopathic BAD	Idiopathic BAD	0.7160564454614798
98	B53	B31	Idiopathic BAD	Idiopathic BAD	0.8590770404271548
99	B53	B35	Idiopathic BAD	Idiopathic BAD	0.9252479023646072
100	B53	B39	Idiopathic BAD	Idiopathic BAD	0.9094202898550725
101	B53	B43	Idiopathic BAD	Idiopathic BAD	0.8958810068649885
102	B53	B47	Idiopathic BAD	Idiopathic BAD	0.797673531655225
103	B53	B48	Idiopathic BAD	Idiopathic BAD	0.9172387490465294
104	B53	B49	Idiopathic BAD	Idiopathic BAD	0.8842486651411137
105	B54	B1	Idiopathic BAD	Idiopathic BAD	0.9414569031273837
106	B54	B5	Idiopathic BAD	Idiopathic BAD	0.9172387490465294
107	B54	B6	Idiopathic BAD	Idiopathic BAD	0.947177269260107
108	B54	B10	Idiopathic BAD	Idiopathic BAD	0.9347826086956522
109	B54	B17	Idiopathic BAD	Idiopathic BAD	0.5093440122044242
110	B54	B20	Idiopathic BAD	Idiopathic BAD	0.601258581235698
111	B54	B23	Idiopathic BAD	Idiopathic BAD	0.8569794050343249
112	B54	B31	Idiopathic BAD	Idiopathic BAD	0.9168573607932876
113	B54	B35	Idiopathic BAD	Idiopathic BAD	0.9876048817696415
114	B54	B39	Idiopathic BAD	Idiopathic BAD	0.9124713958810069
115	B54	B43	Idiopathic BAD	Idiopathic BAD	0.841723874904653
116	B54	B47	Idiopathic BAD	Idiopathic BAD	0.8047292143401983
117	B54	B48	Idiopathic BAD	Idiopathic BAD	0.954042715484363
118	B54	B49	Idiopathic BAD	Idiopathic BAD	0.9244851258581236
119	B54	B53	Idiopathic BAD	Idiopathic BAD	0.8590770404271548

120	B55	B1	Idiopathic BAD	Idiopathic BAD	0.9698703279938978
121	B55	B5	Idiopathic BAD	Idiopathic BAD	0.9170480549199085
122	B55	B6	Idiopathic BAD	Idiopathic BAD	0.9448893974065599
123	B55	B10	Idiopathic BAD	Idiopathic BAD	0.9630053394355453
124	B55	B17	Idiopathic BAD	Idiopathic BAD	0.5915331807780321
125	B55	B20	Idiopathic BAD	Idiopathic BAD	0.6983218916857361
126	B55	B23	Idiopathic BAD	Idiopathic BAD	0.8196033562166285
127	B55	B31	Idiopathic BAD	Idiopathic BAD	0.9366895499618612
128	B55	B35	Idiopathic BAD	Idiopathic BAD	0.9754004576659039
129	B55	B39	Idiopathic BAD	Idiopathic BAD	0.9035087719298246
130	B55	B43	Idiopathic BAD	Idiopathic BAD	0.8600305110602593
131	B55	B47	Idiopathic BAD	Idiopathic BAD	0.8770022883295194
132	B55	B48	Idiopathic BAD	Idiopathic BAD	0.967391304347826
133	B55	B49	Idiopathic BAD	Idiopathic BAD	0.9258199847444699
134	B55	B53	Idiopathic BAD	Idiopathic BAD	0.8602212051868803
135	B55	B54	Idiopathic BAD	Idiopathic BAD	0.5411899313501144
136	B59	B1	Idiopathic BAD	Idiopathic BAD	0.8665141113653699
137	B59	B5	Idiopathic BAD	Idiopathic BAD	0.9138062547673532
138	B59	B6	Idiopathic BAD	Idiopathic BAD	0.9731121281464531
139	B59	B10	Idiopathic BAD	Idiopathic BAD	0.7883295194508009
140	B59	B17	Idiopathic BAD	Idiopathic BAD	0.9809305873379099
141	B59	B20	Idiopathic BAD	Idiopathic BAD	0.9822654462242563
142	B59	B23	Idiopathic BAD	Idiopathic BAD	0.9597635392829901
143	B59	B31	Idiopathic BAD	Idiopathic BAD	0.8499237223493517
144	B59	B35	Idiopathic BAD	Idiopathic BAD	0.9994279176201373
145	B59	B39	Idiopathic BAD	Idiopathic BAD	0.9479405034324943
146	B59	B43	Idiopathic BAD	Idiopathic BAD	0.9837909992372235

147	B59	B47	Idiopathic BAD	Idiopathic BAD	0.9826468344774981
148	B59	B48	Idiopathic BAD	Idiopathic BAD	0.9769260106788711
149	B59	B49	Idiopathic BAD	Idiopathic BAD	0.9876048817696415
150	B59	B53	Idiopathic BAD	Idiopathic BAD	0.9761632341723875
151	B59	B54	Idiopathic BAD	Idiopathic BAD	0.9876048817696415
152	B59	B55	Idiopathic BAD	Idiopathic BAD	0.9809305873379099
153	B70	B1	Idiopathic BAD	Idiopathic BAD	0.9258199847444699
154	B70	B5	Idiopathic BAD	Idiopathic BAD	0.7898550724637681
155	B70	B6	Idiopathic BAD	Idiopathic BAD	0.9458428680396643
156	B70	B10	Idiopathic BAD	Idiopathic BAD	0.9519450800915332
157	B70	B17	Idiopathic BAD	Idiopathic BAD	0.8865369946605645
158	B70	B20	Idiopathic BAD	Idiopathic BAD	0.9130434782608695
159	B70	B23	Idiopathic BAD	Idiopathic BAD	0.702326468344775
160	B70	B31	Idiopathic BAD	Idiopathic BAD	0.9113272311212814
161	B70	B35	Idiopathic BAD	Idiopathic BAD	0.9683447749809306
162	B70	B39	Idiopathic BAD	Idiopathic BAD	0.8811975591151793
163	B70	B43	Idiopathic BAD	Idiopathic BAD	0.9534706331045004
164	B70	B47	Idiopathic BAD	Idiopathic BAD	0.8401983218916858
165	B70	B48	Idiopathic BAD	Idiopathic BAD	0.916094584286804
166	B70	B49	Idiopathic BAD	Idiopathic BAD	0.9614797864225781
167	B70	B53	Idiopathic BAD	Idiopathic BAD	0.7303585049580473
168	B70	B54	Idiopathic BAD	Idiopathic BAD	0.9086575133485889
169	B70	B55	Idiopathic BAD	Idiopathic BAD	0.9031273836765827
170	B70	B59	Idiopathic BAD	Idiopathic BAD	0.9830282227307399
171	B74	B1	Idiopathic BAD	Idiopathic BAD	0.9202898550724637
172	B74	B5	Idiopathic BAD	Idiopathic BAD	0.90255530129672
173	B74	B6	Idiopathic BAD	Idiopathic BAD	0.8831045003813882

174	B74	B10	Idiopathic BAD	Idiopathic BAD
0.8299008390541571
175	B74	B17	Idiopathic BAD	Idiopathic BAD
0.7589626239511823
176	B74	B20	Idiopathic BAD	Idiopathic BAD
0.8975972540045767
177	B74	B23	Idiopathic BAD	Idiopathic BAD
0.7541952707856598
178	B74	B31	Idiopathic BAD	Idiopathic BAD
0.8070175438596491
179	B74	B35	Idiopathic BAD	Idiopathic BAD
0.9473684210526315
180	B74	B39	Idiopathic BAD	Idiopathic BAD
0.8905415713196033
181	B74	B43	Idiopathic BAD	Idiopathic BAD
0.9344012204424104
182	B74	B47	Idiopathic BAD	Idiopathic BAD
0.7704042715484363
183	B74	B48	Idiopathic BAD	Idiopathic BAD
0.9660564454614798
184	B74	B49	Idiopathic BAD	Idiopathic BAD
0.8785278413424866
185	B74	B53	Idiopathic BAD	Idiopathic BAD
0.8882532418001525
186	B74	B54	Idiopathic BAD	Idiopathic BAD
0.9118993135011442
187	B74	B55	Idiopathic BAD	Idiopathic BAD
0.8651792524790236
188	B74	B59	Idiopathic BAD	Idiopathic BAD
0.9040808543096872
189	B74	B70	Idiopathic BAD	Idiopathic BAD
0.9046529366895499
190	B77	B1	Idiopathic BAD	Idiopathic BAD
0.9269641495041953
191	B77	B5	Idiopathic BAD	Idiopathic BAD
0.8443935926773455
192	B77	B6	Idiopathic BAD	Idiopathic BAD
0.9067505720823799
193	B77	B10	Idiopathic BAD	Idiopathic BAD
0.8781464530892449
194	B77	B17	Idiopathic BAD	Idiopathic BAD
0.8237986270022883
195	B77	B20	Idiopathic BAD	Idiopathic BAD
0.8337147215865751
196	B77	B23	Idiopathic BAD	Idiopathic BAD
0.7315026697177727
197	B77	B31	Idiopathic BAD	Idiopathic BAD
0.8495423340961098
198	B77	B35	Idiopathic BAD	Idiopathic BAD
0.9570938215102975
199	B77	B39	Idiopathic BAD	Idiopathic BAD
0.8592677345537757
200	B77	B43	Idiopathic BAD	Idiopathic BAD
0.8914950419527079

201 B77 B47 Idiopathic BAD Idiopathic BAD
0.6065980167810832
202 B77 B48 Idiopathic BAD Idiopathic BAD
0.9519450800915332
203 B77 B49 Idiopathic BAD Idiopathic BAD
0.8314263920671243
204 B77 B53 Idiopathic BAD Idiopathic BAD
0.7808924485125858
205 B77 B54 Idiopathic BAD Idiopathic BAD
0.8825324180015256
206 B77 B55 Idiopathic BAD Idiopathic BAD
0.8430587337909993
207 B77 B59 Idiopathic BAD Idiopathic BAD
0.9717772692601068
208 B77 B70 Idiopathic BAD Idiopathic BAD
0.8316170861937452
209 B77 B74 Idiopathic BAD Idiopathic BAD
0.8217009916094584
210 B81 B1 Idiopathic BAD Idiopathic BAD
0.8750953470633105
211 B81 B5 Idiopathic BAD Idiopathic BAD
0.8752860411899314
212 B81 B6 Idiopathic BAD Idiopathic BAD
0.8869183829138062
213 B81 B10 Idiopathic BAD Idiopathic BAD
0.8562166285278413
214 B81 B17 Idiopathic BAD Idiopathic BAD
0.8623188405797102
215 B81 B20 Idiopathic BAD Idiopathic BAD
0.8974065598779558
216 B81 B23 Idiopathic BAD Idiopathic BAD
0.8842486651411137
217 B81 B31 Idiopathic BAD Idiopathic BAD
0.7553394355453852
218 B81 B35 Idiopathic BAD Idiopathic BAD
0.7980549199084668
219 B81 B39 Idiopathic BAD Idiopathic BAD
0.9021739130434783
220 B81 B43 Idiopathic BAD Idiopathic BAD
0.8993135011441648
221 B81 B47 Idiopathic BAD Idiopathic BAD
0.8276125095347063
222 B81 B48 Idiopathic BAD Idiopathic BAD
0.8407704042715485
223 B81 B49 Idiopathic BAD Idiopathic BAD
0.7568649885583524
224 B81 B53 Idiopathic BAD Idiopathic BAD
0.8047292143401983
225 B81 B54 Idiopathic BAD Idiopathic BAD
0.9036994660564455
226 B81 B55 Idiopathic BAD Idiopathic BAD
0.9336384439359268
227 B81 B59 Idiopathic BAD Idiopathic BAD
0.9626239511823036

228	B81	B70	Idiopathic BAD	Idiopathic BAD
0.9399313501144165
229	B81	B74	Idiopathic BAD	Idiopathic BAD
0.7877574370709383
230	B81	B77	Idiopathic BAD	Idiopathic BAD
0.8781464530892449
231	B84	B1	Idiopathic BAD	Idiopathic BAD
0.8718535469107551
232	B84	B5	Idiopathic BAD	Idiopathic BAD
0.7753623188405797
233	B84	B6	Idiopathic BAD	Idiopathic BAD
0.8381006864988558
234	B84	B10	Idiopathic BAD	Idiopathic BAD
0.9004576659038902
235	B84	B17	Idiopathic BAD	Idiopathic BAD
0.8903508771929824
236	B84	B20	Idiopathic BAD	Idiopathic BAD
0.8615560640732265
237	B84	B23	Idiopathic BAD	Idiopathic BAD
0.8121662852784134
238	B84	B31	Idiopathic BAD	Idiopathic BAD
0.8699466056445462
239	B84	B35	Idiopathic BAD	Idiopathic BAD
0.9546147978642258
240	B84	B39	Idiopathic BAD	Idiopathic BAD
0.8966437833714722
241	B84	B43	Idiopathic BAD	Idiopathic BAD
0.8094965675057209
242	B84	B47	Idiopathic BAD	Idiopathic BAD
0.8264683447749809
243	B84	B48	Idiopathic BAD	Idiopathic BAD
0.9077040427154843
244	B84	B49	Idiopathic BAD	Idiopathic BAD
0.8922578184591915
245	B84	B53	Idiopathic BAD	Idiopathic BAD
0.8674675819984744
246	B84	B54	Idiopathic BAD	Idiopathic BAD
0.9189549961861174
247	B84	B55	Idiopathic BAD	Idiopathic BAD
0.8766209000762777
248	B84	B59	Idiopathic BAD	Idiopathic BAD
0.9233409610983981
249	B84	B70	Idiopathic BAD	Idiopathic BAD
0.8272311212814645
250	B84	B74	Idiopathic BAD	Idiopathic BAD
0.9029366895499619
251	B84	B77	Idiopathic BAD	Idiopathic BAD
0.8102593440122045
252	B84	B81	Idiopathic BAD	Idiopathic BAD
0.9321128909229596
253	B89	B1	Idiopathic BAD	Idiopathic BAD
0.8590770404271548
254	B89	B5	Idiopathic BAD	Idiopathic BAD
0.7660183066361556

255	B89	B6	Idiopathic BAD	Idiopathic BAD
0.8651792524790236
256	B89	B10	Idiopathic BAD	Idiopathic BAD
0.9666285278413425
257	B89	B17	Idiopathic BAD	Idiopathic BAD
0.9023646071700991
258	B89	B20	Idiopathic BAD	Idiopathic BAD
0.8079710144927537
259	B89	B23	Idiopathic BAD	Idiopathic BAD
0.8287566742944318
260	B89	B31	Idiopathic BAD	Idiopathic BAD
0.8897787948131197
261	B89	B35	Idiopathic BAD	Idiopathic BAD
0.8718535469107551
262	B89	B39	Idiopathic BAD	Idiopathic BAD
0.8838672768878718
263	B89	B43	Idiopathic BAD	Idiopathic BAD
0.8409610983981693
264	B89	B47	Idiopathic BAD	Idiopathic BAD
0.8607932875667429
265	B89	B48	Idiopathic BAD	Idiopathic BAD
0.8756674294431731
266	B89	B49	Idiopathic BAD	Idiopathic BAD
0.8222730739893211
267	B89	B53	Idiopathic BAD	Idiopathic BAD
0.868230358504958
268	B89	B54	Idiopathic BAD	Idiopathic BAD
0.9138062547673532
269	B89	B55	Idiopathic BAD	Idiopathic BAD
0.9399313501144165
270	B89	B59	Idiopathic BAD	Idiopathic BAD
0.9897025171624714
271	B89	B70	Idiopathic BAD	Idiopathic BAD
0.9098016781083142
272	B89	B74	Idiopathic BAD	Idiopathic BAD
0.9279176201372997
273	B89	B77	Idiopathic BAD	Idiopathic BAD
0.918001525553013
274	B89	B81	Idiopathic BAD	Idiopathic BAD
0.8390541571319603
275	B89	B84	Idiopathic BAD	Idiopathic BAD
0.8443935926773455
276	B92	B1	Idiopathic BAD	Idiopathic BAD
0.8611746758199847
277	B92	B5	Idiopathic BAD	Idiopathic BAD
0.7597254004576659
278	B92	B6	Idiopathic BAD	Idiopathic BAD
0.8766209000762777
279	B92	B10	Idiopathic BAD	Idiopathic BAD
0.8535469107551488
280	B92	B17	Idiopathic BAD	Idiopathic BAD
0.6203279938977879
281	B92	B20	Idiopathic BAD	Idiopathic BAD
0.5678871090770404

282	B92	B23	Idiopathic BAD	Idiopathic BAD	0.7049961861174676
283	B92	B31	Idiopathic BAD	Idiopathic BAD	0.7915713196033562
284	B92	B35	Idiopathic BAD	Idiopathic BAD	0.9353546910755148
285	B92	B39	Idiopathic BAD	Idiopathic BAD	0.8714721586575134
286	B92	B43	Idiopathic BAD	Idiopathic BAD	0.6756292906178489
287	B92	B47	Idiopathic BAD	Idiopathic BAD	0.8045385202135774
288	B92	B48	Idiopathic BAD	Idiopathic BAD	0.9443173150266971
289	B92	B49	Idiopathic BAD	Idiopathic BAD	0.8415331807780321
290	B92	B53	Idiopathic BAD	Idiopathic BAD	0.8026315789473685
291	B92	B54	Idiopathic BAD	Idiopathic BAD	0.6649504195270786
292	B92	B55	Idiopathic BAD	Idiopathic BAD	0.6866895499618612
293	B92	B59	Idiopathic BAD	Idiopathic BAD	0.9509916094584286
294	B92	B70	Idiopathic BAD	Idiopathic BAD	0.8260869565217391
295	B92	B74	Idiopathic BAD	Idiopathic BAD	0.835812356979405
296	B92	B77	Idiopathic BAD	Idiopathic BAD	0.8190312738367659
297	B92	B81	Idiopathic BAD	Idiopathic BAD	0.8745232646834478
298	B92	B84	Idiopathic BAD	Idiopathic BAD	0.7755530129672006
299	B92	B89	Idiopathic BAD	Idiopathic BAD	0.7860411899313501
300	B95	B1	Idiopathic BAD	Idiopathic BAD	0.8647978642257819
301	B95	B5	Idiopathic BAD	Idiopathic BAD	0.830091533180778
302	B95	B6	Idiopathic BAD	Idiopathic BAD	0.8964530892448512
303	B95	B10	Idiopathic BAD	Idiopathic BAD	0.9490846681922197
304	B95	B17	Idiopathic BAD	Idiopathic BAD	0.816933638443936
305	B95	B20	Idiopathic BAD	Idiopathic BAD	0.6033562166285278
306	B95	B23	Idiopathic BAD	Idiopathic BAD	0.9149504195270786
307	B95	B31	Idiopathic BAD	Idiopathic BAD	0.8827231121281465
308	B95	B35	Idiopathic BAD	Idiopathic BAD	0.8790999237223494

309 B95 B39 Idiopathic BAD Idiopathic BAD
0.8954996186117468
310 B95 B43 Idiopathic BAD Idiopathic BAD
0.7088100686498856
311 B95 B47 Idiopathic BAD Idiopathic BAD
0.8518306636155606
312 B95 B48 Idiopathic BAD Idiopathic BAD
0.8066361556064073
313 B95 B49 Idiopathic BAD Idiopathic BAD
0.7810831426392068
314 B95 B53 Idiopathic BAD Idiopathic BAD
0.8792906178489702
315 B95 B54 Idiopathic BAD Idiopathic BAD
0.7871853546910755
316 B95 B55 Idiopathic BAD Idiopathic BAD
0.8625095347063311
317 B95 B59 Idiopathic BAD Idiopathic BAD
0.9874141876430206
318 B95 B70 Idiopathic BAD Idiopathic BAD
0.8960717009916095
319 B95 B74 Idiopathic BAD Idiopathic BAD
0.9559496567505721
320 B95 B77 Idiopathic BAD Idiopathic BAD
0.9202898550724637
321 B95 B81 Idiopathic BAD Idiopathic BAD
0.8724256292906178
322 B95 B84 Idiopathic BAD Idiopathic BAD
0.9212433257055682
323 B95 B89 Idiopathic BAD Idiopathic BAD
0.8146453089244852
324 B95 B92 Idiopathic BAD Idiopathic BAD
0.6718154080854309
325 B99 B1 Idiopathic BAD Idiopathic BAD
0.8327612509534706
326 B99 B5 Idiopathic BAD Idiopathic BAD
0.7362700228832952
327 B99 B6 Idiopathic BAD Idiopathic BAD
0.7698321891685737
328 B99 B10 Idiopathic BAD Idiopathic BAD
0.9038901601830663
329 B99 B17 Idiopathic BAD Idiopathic BAD
0.8096872616323417
330 B99 B20 Idiopathic BAD Idiopathic BAD
0.7515255530129672
331 B99 B23 Idiopathic BAD Idiopathic BAD
0.7627765064836003
332 B99 B31 Idiopathic BAD Idiopathic BAD
0.8623188405797102
333 B99 B35 Idiopathic BAD Idiopathic BAD
0.9422196796338673
334 B99 B39 Idiopathic BAD Idiopathic BAD
0.7675438596491229
335 B99 B43 Idiopathic BAD Idiopathic BAD
0.7492372234935164

336	B99	B47	Idiopathic BAD	Idiopathic BAD	0.6891685736079328
337	B99	B48	Idiopathic BAD	Idiopathic BAD	0.8508771929824561
338	B99	B49	Idiopathic BAD	Idiopathic BAD	0.6430205949656751
339	B99	B53	Idiopathic BAD	Idiopathic BAD	0.7974828375286042
340	B99	B54	Idiopathic BAD	Idiopathic BAD	0.8592677345537757
341	B99	B55	Idiopathic BAD	Idiopathic BAD	0.885392829900839
342	B99	B59	Idiopathic BAD	Idiopathic BAD	0.9809305873379099
343	B99	B70	Idiopathic BAD	Idiopathic BAD	0.8196033562166285
344	B99	B74	Idiopathic BAD	Idiopathic BAD	0.8316170861937452
345	B99	B77	Idiopathic BAD	Idiopathic BAD	0.8087337909992373
346	B99	B81	Idiopathic BAD	Idiopathic BAD	0.7847063310450039
347	B99	B84	Idiopathic BAD	Idiopathic BAD	0.8152173913043478
348	B99	B89	Idiopathic BAD	Idiopathic BAD	0.6714340198321892
349	B99	B92	Idiopathic BAD	Idiopathic BAD	0.7309305873379099
350	B99	B95	Idiopathic BAD	Idiopathic BAD	0.7831807780320366
351	B103	B1	Idiopathic BAD	Idiopathic BAD	0.9546147978642258
352	B103	B5	Idiopathic BAD	Idiopathic BAD	0.8941647597254004
353	B103	B6	Idiopathic BAD	Idiopathic BAD	0.8377192982456141
354	B103	B10	Idiopathic BAD	Idiopathic BAD	0.9698703279938978
355	B103	B17	Idiopathic BAD	Idiopathic BAD	0.900839054157132
356	B103	B20	Idiopathic BAD	Idiopathic BAD	0.9363081617086194
357	B103	B23	Idiopathic BAD	Idiopathic BAD	0.8634630053394355
358	B103	B31	Idiopathic BAD	Idiopathic BAD	0.9183829138062548
359	B103	B35	Idiopathic BAD	Idiopathic BAD	0.9288710907704043
360	B103	B39	Idiopathic BAD	Idiopathic BAD	0.9250572082379863
361	B103	B43	Idiopathic BAD	Idiopathic BAD	0.86441647597254
362	B103	B47	Idiopathic BAD	Idiopathic BAD	0.8878718535469108

363	B103	B48	Idiopathic BAD	Idiopathic BAD
0.9586193745232647
364	B103	B49	Idiopathic BAD	Idiopathic BAD
0.8642257818459191
365	B103	B53	Idiopathic BAD	Idiopathic BAD
0.902745995423341
366	B103	B54	Idiopathic BAD	Idiopathic BAD
0.9807398932112891
367	B103	B55	Idiopathic BAD	Idiopathic BAD
0.958047292143402
368	B103	B59	Idiopathic BAD	Idiopathic BAD
0.9797864225781846
369	B103	B70	Idiopathic BAD	Idiopathic BAD
0.9372616323417239
370	B103	B74	Idiopathic BAD	Idiopathic BAD
0.9532799389778794
371	B103	B77	Idiopathic BAD	Idiopathic BAD
0.9115179252479023
372	B103	B81	Idiopathic BAD	Idiopathic BAD
0.9262013729977117
373	B103	B84	Idiopathic BAD	Idiopathic BAD
0.8371472158657514
374	B103	B89	Idiopathic BAD	Idiopathic BAD
0.9031273836765827
375	B103	B92	Idiopathic BAD	Idiopathic BAD
0.8895881006864989
376	B103	B95	Idiopathic BAD	Idiopathic BAD
0.9496567505720824
377	B103	B99	Idiopathic BAD	Idiopathic BAD
0.8503051106025934
378	B106	B1	Idiopathic BAD	Idiopathic BAD
0.973302822273074
379	B106	B5	Idiopathic BAD	Idiopathic BAD
0.9521357742181541
380	B106	B6	Idiopathic BAD	Idiopathic BAD
0.9548054919908466
381	B106	B10	Idiopathic BAD	Idiopathic BAD
0.9527078565980168
382	B106	B17	Idiopathic BAD	Idiopathic BAD
0.5751334858886347
383	B106	B20	Idiopathic BAD	Idiopathic BAD
0.6926010678871091
384	B106	B23	Idiopathic BAD	Idiopathic BAD
0.9366895499618612
385	B106	B31	Idiopathic BAD	Idiopathic BAD
0.9652936689549961
386	B106	B35	Idiopathic BAD	Idiopathic BAD
0.9893211289092296
387	B106	B39	Idiopathic BAD	Idiopathic BAD
0.9696796338672768
388	B106	B43	Idiopathic BAD	Idiopathic BAD
0.8217009916094584
389	B106	B47	Idiopathic BAD	Idiopathic BAD
0.9424103737604882

390	B106	B48	Idiopathic BAD	Idiopathic BAD
0.9742562929061785
391	B106	B49	Idiopathic BAD	Idiopathic BAD
0.9475591151792525
392	B106	B53	Idiopathic BAD	Idiopathic BAD
0.9433638443935927
393	B106	B54	Idiopathic BAD	Idiopathic BAD
0.5928680396643783
394	B106	B55	Idiopathic BAD	Idiopathic BAD
0.501906941266209
395	B106	B59	Idiopathic BAD	Idiopathic BAD
0.9681540808543097
396	B106	B70	Idiopathic BAD	Idiopathic BAD
0.9807398932112891
397	B106	B74	Idiopathic BAD	Idiopathic BAD
0.9548054919908466
398	B106	B77	Idiopathic BAD	Idiopathic BAD
0.9256292906178489
399	B106	B81	Idiopathic BAD	Idiopathic BAD
0.9481311975591151
400	B106	B84	Idiopathic BAD	Idiopathic BAD
0.9637681159420289
401	B106	B89	Idiopathic BAD	Idiopathic BAD
0.9616704805491991
402	B106	B92	Idiopathic BAD	Idiopathic BAD
0.7540045766590389
403	B106	B95	Idiopathic BAD	Idiopathic BAD
0.8476353928299009
404	B106	B99	Idiopathic BAD	Idiopathic BAD
0.9303966437833715
405	B106	B103	Idiopathic BAD	Idiopathic BAD
0.9645308924485125
406	B109	B1	Idiopathic BAD	Idiopathic BAD
0.8924485125858124
407	B109	B5	Idiopathic BAD	Idiopathic BAD
0.7847063310450039
408	B109	B6	Idiopathic BAD	Idiopathic BAD
0.9088482074752098
409	B109	B10	Idiopathic BAD	Idiopathic BAD
0.9445080091533181
410	B109	B17	Idiopathic BAD	Idiopathic BAD
0.8872997711670481
411	B109	B20	Idiopathic BAD	Idiopathic BAD
0.8840579710144928
412	B109	B23	Idiopathic BAD	Idiopathic BAD
0.7517162471395881
413	B109	B31	Idiopathic BAD	Idiopathic BAD
0.8926392067124332
414	B109	B35	Idiopathic BAD	Idiopathic BAD
0.9479405034324943
415	B109	B39	Idiopathic BAD	Idiopathic BAD
0.927536231884058
416	B109	B43	Idiopathic BAD	Idiopathic BAD
0.9317315026697178

417	B109	B47	Idiopathic BAD	Idiopathic BAD
0.8375286041189931
418	B109	B48	Idiopathic BAD	Idiopathic BAD
0.916094584286804
419	B109	B49	Idiopathic BAD	Idiopathic BAD
0.835812356979405
420	B109	B53	Idiopathic BAD	Idiopathic BAD
0.8329519450800915
421	B109	B54	Idiopathic BAD	Idiopathic BAD
0.916094584286804
422	B109	B55	Idiopathic BAD	Idiopathic BAD
0.8707093821510298
423	B109	B59	Idiopathic BAD	Idiopathic BAD
0.9938977879481312
424	B109	B70	Idiopathic BAD	Idiopathic BAD
0.6840198321891686
425	B109	B74	Idiopathic BAD	Idiopathic BAD
0.8945461479786423
426	B109	B77	Idiopathic BAD	Idiopathic BAD
0.8310450038138826
427	B109	B81	Idiopathic BAD	Idiopathic BAD
0.830091533180778
428	B109	B84	Idiopathic BAD	Idiopathic BAD
0.8586956521739131
429	B109	B89	Idiopathic BAD	Idiopathic BAD
0.864607170099161
430	B109	B92	Idiopathic BAD	Idiopathic BAD
0.8188405797101449
431	B109	B95	Idiopathic BAD	Idiopathic BAD
0.8327612509534706
432	B109	B99	Idiopathic BAD	Idiopathic BAD
0.7364607170099161
433	B109	B103	Idiopathic BAD	Idiopathic BAD
0.9012204424103738
434	B109	B106	Idiopathic BAD	Idiopathic BAD
0.9509916094584286
435	B118	B1	Idiopathic BAD	Idiopathic BAD
0.9416475972540046
436	B118	B5	Idiopathic BAD	Idiopathic BAD
0.9021739130434783
437	B118	B6	Idiopathic BAD	Idiopathic BAD
0.8979786422578184
438	B118	B10	Idiopathic BAD	Idiopathic BAD
0.9256292906178489
439	B118	B17	Idiopathic BAD	Idiopathic BAD
0.59744469870328
440	B118	B20	Idiopathic BAD	Idiopathic BAD
0.6413043478260869
441	B118	B23	Idiopathic BAD	Idiopathic BAD
0.872234935163997
442	B118	B31	Idiopathic BAD	Idiopathic BAD
0.8859649122807017
443	B118	B35	Idiopathic BAD	Idiopathic BAD
0.9813119755911518

444	B118	B39	Idiopathic BAD	Idiopathic BAD	0.9256292906178489
445	B118	B43	Idiopathic BAD	Idiopathic BAD	0.6937452326468345
446	B118	B47	Idiopathic BAD	Idiopathic BAD	0.8175057208237986
447	B118	B48	Idiopathic BAD	Idiopathic BAD	0.9609077040427155
448	B118	B49	Idiopathic BAD	Idiopathic BAD	0.9227688787185355
449	B118	B53	Idiopathic BAD	Idiopathic BAD	0.8440122044241037
450	B118	B54	Idiopathic BAD	Idiopathic BAD	0.5953470633104501
451	B118	B55	Idiopathic BAD	Idiopathic BAD	0.6878337147215866
452	B118	B59	Idiopathic BAD	Idiopathic BAD	0.9755911517925248
453	B118	B70	Idiopathic BAD	Idiopathic BAD	0.8773836765827613
454	B118	B74	Idiopathic BAD	Idiopathic BAD	0.8867276887871853
455	B118	B77	Idiopathic BAD	Idiopathic BAD	0.9086575133485889
456	B118	B81	Idiopathic BAD	Idiopathic BAD	0.8861556064073226
457	B118	B84	Idiopathic BAD	Idiopathic BAD	0.9397406559877955
458	B118	B89	Idiopathic BAD	Idiopathic BAD	0.8411517925247902
459	B118	B92	Idiopathic BAD	Idiopathic BAD	0.6098398169336384
460	B118	B95	Idiopathic BAD	Idiopathic BAD	0.7076659038901602
461	B118	B99	Idiopathic BAD	Idiopathic BAD	0.7902364607170099
462	B118	B103	Idiopathic BAD	Idiopathic BAD	0.9260106788710908
463	B118	B106	Idiopathic BAD	Idiopathic BAD	0.6767734553775744
464	B118	B109	Idiopathic BAD	Idiopathic BAD	0.9124713958810069
465	B119	B1	Idiopathic BAD	Idiopathic BAD	0.8497330282227308
466	B119	B5	Idiopathic BAD	Idiopathic BAD	0.6988939740655988
467	B119	B6	Idiopathic BAD	Idiopathic BAD	0.7509534706331045
468	B119	B10	Idiopathic BAD	Idiopathic BAD	0.8842486651411137
469	B119	B17	Idiopathic BAD	Idiopathic BAD	0.8194126620900076
470	B119	B20	Idiopathic BAD	Idiopathic BAD	0.861556064073226

471	B119	B23	Idiopathic BAD	Idiopathic BAD	0.6802059496567505
472	B119	B31	Idiopathic BAD	Idiopathic BAD	0.8579328756674295
473	B119	B35	Idiopathic BAD	Idiopathic BAD	0.8953089244851259
474	B119	B39	Idiopathic BAD	Idiopathic BAD	0.8428680396643783
475	B119	B43	Idiopathic BAD	Idiopathic BAD	0.8611746758199847
476	B119	B47	Idiopathic BAD	Idiopathic BAD	0.7866132723112128
477	B119	B48	Idiopathic BAD	Idiopathic BAD	0.7810831426392068
478	B119	B49	Idiopathic BAD	Idiopathic BAD	0.8205568268497331
479	B119	B53	Idiopathic BAD	Idiopathic BAD	0.7665903890160183
480	B119	B54	Idiopathic BAD	Idiopathic BAD	0.8922578184591915
481	B119	B55	Idiopathic BAD	Idiopathic BAD	0.8504958047292144
482	B119	B59	Idiopathic BAD	Idiopathic BAD	0.9549961861174676
483	B119	B70	Idiopathic BAD	Idiopathic BAD	0.7145308924485125
484	B119	B74	Idiopathic BAD	Idiopathic BAD	0.8138825324180016
485	B119	B77	Idiopathic BAD	Idiopathic BAD	0.7761250953470633
486	B119	B81	Idiopathic BAD	Idiopathic BAD	0.7469488939740656
487	B119	B84	Idiopathic BAD	Idiopathic BAD	0.8020594965675057
488	B119	B89	Idiopathic BAD	Idiopathic BAD	0.8363844393592678
489	B119	B92	Idiopathic BAD	Idiopathic BAD	0.7892829900839055
490	B119	B95	Idiopathic BAD	Idiopathic BAD	0.8394355453852022
491	B119	B99	Idiopathic BAD	Idiopathic BAD	0.6956521739130435
492	B119	B103	Idiopathic BAD	Idiopathic BAD	0.8714721586575134
493	B119	B106	Idiopathic BAD	Idiopathic BAD	0.9366895499618612
494	B119	B109	Idiopathic BAD	Idiopathic BAD	0.7299771167048055
495	B119	B118	Idiopathic BAD	Idiopathic BAD	0.9014111365369947
496	B1	B27	Idiopathic BAD	Control group	0.8424866514111365
497	B1	B66	Idiopathic BAD	Control group	0.8979786422578184

498 B1 B86 Idiopathic BAD Control group
0.9178108314263921
499 B1 B97 Idiopathic BAD Control group
0.8520213577421816
500 B1 B98 Idiopathic BAD Control group
0.7566742944317315
501 B1 B100 Idiopathic BAD Control group
0.9435545385202135
502 B1 B112 Idiopathic BAD Control group
0.9311594202898551
503 B1 B115 Idiopathic BAD Control group
0.8096872616323417
504 B1 I1 Idiopathic BAD Control group
0.7942410373760488
505 B1 I3 Idiopathic BAD Control group
0.8400076277650649
506 B1 I6 Idiopathic BAD Control group
0.8892067124332571
507 B1 I8 Idiopathic BAD Control group
0.9046529366895499
508 B1 I10 Idiopathic BAD Control group
0.9071319603356217
509 B1 I11 Idiopathic BAD Control group
0.8278032036613272
510 B1 I13 Idiopathic BAD Control group
0.9128527841342486
511 B1 I15 Idiopathic BAD Control group
0.8239893211289092
512 B1 I17 Idiopathic BAD Control group
0.9496567505720824
513 B1 I18 Idiopathic BAD Control group
0.8257055682684973
514 B1 I19 Idiopathic BAD Control group
0.9509916094584286
515 B1 I22 Idiopathic BAD Control group
0.8012967200610221
516 B1 I23 Idiopathic BAD Control group
0.8636536994660564
517 B1 I24 Idiopathic BAD Control group
0.9578565980167811
518 B1 I25 Idiopathic BAD Control group
0.8647978642257819
519 B1 I26 Idiopathic BAD Control group
0.9187643020594966
520 B1 I27 Idiopathic BAD Control group
0.9086575133485889
521 B1 I28 Idiopathic BAD Control group
0.8203661327231121
522 B1 I29 Idiopathic BAD Control group
0.9252479023646072
523 B1 I30 Idiopathic BAD Control group
0.8876811594202898
524 B1 I31 Idiopathic BAD Control group
0.9412662090007627

525 B1 I32 Idiopathic BAD Control group
0.9441266209000763
526 B1 I33 Idiopathic BAD Control group
0.910373760488177
527 B1 I34 Idiopathic BAD Control group
0.9380244088482075
528 B1 I35 Idiopathic BAD Control group
0.8560259344012204
529 B1 I36 Idiopathic BAD Control group
0.8918764302059496
530 B1 I37 Idiopathic BAD Control group
0.7395118230358505
531 B5 B27 Idiopathic BAD Control group
0.8808161708619374
532 B5 B66 Idiopathic BAD Control group
0.8220823798627003
533 B5 B86 Idiopathic BAD Control group
0.8203661327231121
534 B5 B97 Idiopathic BAD Control group
0.8077803203661327
535 B5 B98 Idiopathic BAD Control group
0.801487414187643
536 B5 B100 Idiopathic BAD Control group
0.9481311975591151
537 B5 B112 Idiopathic BAD Control group
0.8779557589626239
538 B5 B115 Idiopathic BAD Control group
0.8632723112128147
539 B5 I1 Idiopathic BAD Control group
0.8392448512585813
540 B5 I3 Idiopathic BAD Control group
0.7282608695652174
541 B5 I6 Idiopathic BAD Control group
0.822463768115942
542 B5 I8 Idiopathic BAD Control group
0.8688024408848207
543 B5 I10 Idiopathic BAD Control group
0.7585812356979404
544 B5 I11 Idiopathic BAD Control group
0.8758581235697941
545 B5 I13 Idiopathic BAD Control group
0.8466819221967964
546 B5 I15 Idiopathic BAD Control group
0.8054919908466819
547 B5 I17 Idiopathic BAD Control group
0.9128527841342486
548 B5 I18 Idiopathic BAD Control group
0.679252479023646
549 B5 I19 Idiopathic BAD Control group
0.8939740655987796
550 B5 I22 Idiopathic BAD Control group
0.7393211289092296
551 B5 I23 Idiopathic BAD Control group
0.7547673531655225

552	B5	I24	Idiopathic BAD	Control group
0.885392829900839
553	B5	I25	Idiopathic BAD	Control group
0.7797482837528604
554	B5	I26	Idiopathic BAD	Control group
0.7698321891685737
555	B5	I27	Idiopathic BAD	Control group
0.6687643020594966
556	B5	I28	Idiopathic BAD	Control group
0.7890922959572845
557	B5	I29	Idiopathic BAD	Control group
0.8796720061022121
558	B5	I30	Idiopathic BAD	Control group
0.6924103737604882
559	B5	I31	Idiopathic BAD	Control group
0.908276125095347
560	B5	I32	Idiopathic BAD	Control group
0.8665141113653699
561	B5	I33	Idiopathic BAD	Control group
0.8121662852784134
562	B5	I34	Idiopathic BAD	Control group
0.8213196033562167
563	B5	I35	Idiopathic BAD	Control group
0.8438215102974829
564	B5	I36	Idiopathic BAD	Control group
0.9263920671243325
565	B5	I37	Idiopathic BAD	Control group
0.8741418764302059
566	B6	B27	Idiopathic BAD	Control group
0.8735697940503433
567	B6	B66	Idiopathic BAD	Control group
0.8785278413424866
568	B6	B86	Idiopathic BAD	Control group
0.8659420289855072
569	B6	B97	Idiopathic BAD	Control group
0.8598398169336384
570	B6	B98	Idiopathic BAD	Control group
0.8699466056445462
571	B6	B100	Idiopathic BAD	Control group
0.9639588100686499
572	B6	B112	Idiopathic BAD	Control group
0.9153318077803204
573	B6	B115	Idiopathic BAD	Control group
0.8432494279176201
574	B6	I1	Idiopathic BAD	Control group
0.7965293668954996
575	B6	I3	Idiopathic BAD	Control group
0.813119755911518
576	B6	I6	Idiopathic BAD	Control group
0.8501144164759725
577	B6	I8	Idiopathic BAD	Control group
0.8323798627002288
578	B6	I10	Idiopathic BAD	Control group
0.7858504958047292

579	B6	I11	Idiopathic BAD	Control group	0.8394355453852022
580	B6	I13	Idiopathic BAD	Control group	0.746186117467582
581	B6	I15	Idiopathic BAD	Control group	0.851258581235698
582	B6	I17	Idiopathic BAD	Control group	0.8726163234172387
583	B6	I18	Idiopathic BAD	Control group	0.8119755911517925
584	B6	I19	Idiopathic BAD	Control group	0.919717772692601
585	B6	I22	Idiopathic BAD	Control group	0.8432494279176201
586	B6	I23	Idiopathic BAD	Control group	0.8398169336384439
587	B6	I24	Idiopathic BAD	Control group	0.8617467581998475
588	B6	I25	Idiopathic BAD	Control group	0.7774599542334096
589	B6	I26	Idiopathic BAD	Control group	0.8051106025934401
590	B6	I27	Idiopathic BAD	Control group	0.8375286041189931
591	B6	I28	Idiopathic BAD	Control group	0.88558352402746
592	B6	I29	Idiopathic BAD	Control group	0.8394355453852022
593	B6	I30	Idiopathic BAD	Control group	0.8546910755148741
594	B6	I31	Idiopathic BAD	Control group	0.8493516399694889
595	B6	I32	Idiopathic BAD	Control group	0.8123569794050344
596	B6	I33	Idiopathic BAD	Control group	0.8621281464530892
597	B6	I34	Idiopathic BAD	Control group	0.8947368421052632
598	B6	I35	Idiopathic BAD	Control group	0.811022120518688
599	B6	I36	Idiopathic BAD	Control group	0.9956140350877193
600	B6	I37	Idiopathic BAD	Control group	0.8632723112128147
601	B10	B27	Idiopathic BAD	Control group	0.7650648360030511
602	B10	B66	Idiopathic BAD	Control group	0.8672768878718535
603	B10	B86	Idiopathic BAD	Control group	0.8956903127383676
604	B10	B97	Idiopathic BAD	Control group	0.8257055682684973
605	B10	B98	Idiopathic BAD	Control group	0.7362700228832952

606	B10	B100	Idiopathic BAD	Control group
0.8672768878718535
607	B10	B112	Idiopathic BAD	Control group
0.9052250190694127
608	B10	B115	Idiopathic BAD	Control group
0.732837528604119
609	B10	I1	Idiopathic BAD	Control group
0.6594202898550725
610	B10	I3	Idiopathic BAD	Control group
0.8552631578947368
611	B10	I6	Idiopathic BAD	Control group
0.8163615560640732
612	B10	I8	Idiopathic BAD	Control group
0.8346681922196796
613	B10	I10	Idiopathic BAD	Control group
0.916094584286804
614	B10	I11	Idiopathic BAD	Control group
0.8398169336384439
615	B10	I13	Idiopathic BAD	Control group
0.9056064073226545
616	B10	I15	Idiopathic BAD	Control group
0.8346681922196796
617	B10	I17	Idiopathic BAD	Control group
0.8832951945080092
618	B10	I18	Idiopathic BAD	Control group
0.8226544622425629
619	B10	I19	Idiopathic BAD	Control group
0.9309687261632341
620	B10	I22	Idiopathic BAD	Control group
0.7677345537757437
621	B10	I23	Idiopathic BAD	Control group
0.7848970251716247
622	B10	I24	Idiopathic BAD	Control group
0.944698703279939
623	B10	I25	Idiopathic BAD	Control group
0.8766209000762777
624	B10	I26	Idiopathic BAD	Control group
0.8867276887871853
625	B10	I27	Idiopathic BAD	Control group
0.9252479023646072
626	B10	I28	Idiopathic BAD	Control group
0.8789092295957285
627	B10	I29	Idiopathic BAD	Control group
0.9185736079328757
628	B10	I30	Idiopathic BAD	Control group
0.9164759725400458
629	B10	I31	Idiopathic BAD	Control group
0.8935926773455377
630	B10	I32	Idiopathic BAD	Control group
0.9464149504195271
631	B10	I33	Idiopathic BAD	Control group
0.9323035850495804
632	B10	I34	Idiopathic BAD	Control group
0.8802440884820748

633 B10 I35 Idiopathic BAD Control group
0.8564073226544623
634 B10 I36 Idiopathic BAD Control group
0.7637299771167048
635 B10 I37 Idiopathic BAD Control group
0.5223112128146453
636 B17 B27 Idiopathic BAD Control group
0.6607551487414187
637 B17 B66 Idiopathic BAD Control group
0.7873760488176964
638 B17 B86 Idiopathic BAD Control group
0.6203279938977879
639 B17 B97 Idiopathic BAD Control group
0.7522883295194508
640 B17 B98 Idiopathic BAD Control group
0.6250953470633105
641 B17 B100 Idiopathic BAD Control group
0.9231502669717773
642 B17 B112 Idiopathic BAD Control group
0.5106788710907704
643 B17 B115 Idiopathic BAD Control group
0.7192982456140351
644 B17 I1 Idiopathic BAD Control group
0.6712433257055682
645 B17 I3 Idiopathic BAD Control group
0.7921434019832189
646 B17 I6 Idiopathic BAD Control group
0.7585812356979404
647 B17 I8 Idiopathic BAD Control group
0.837909992372235
648 B17 I10 Idiopathic BAD Control group
0.8546910755148741
649 B17 I11 Idiopathic BAD Control group
0.8813882532418001
650 B17 I13 Idiopathic BAD Control group
0.8627002288329519
651 B17 I15 Idiopathic BAD Control group
0.9088482074752098
652 B17 I17 Idiopathic BAD Control group
0.6630434782608695
653 B17 I18 Idiopathic BAD Control group
0.8041571319603357
654 B17 I19 Idiopathic BAD Control group
0.7280701754385965
655 B17 I22 Idiopathic BAD Control group
0.8258962623951183
656 B17 I23 Idiopathic BAD Control group
0.704042715484363
657 B17 I24 Idiopathic BAD Control group
0.6458810068649885
658 B17 I25 Idiopathic BAD Control group
0.813119755911518
659 B17 I26 Idiopathic BAD Control group
0.8203661327231121

660 B17 I27 Idiopathic BAD Control group
0.8041571319603357
661 B17 I28 Idiopathic BAD Control group
0.9075133485888635
662 B17 I29 Idiopathic BAD Control group
0.9061784897025171
663 B17 I30 Idiopathic BAD Control group
0.8619374523264683
664 B17 I31 Idiopathic BAD Control group
0.8508771929824561
665 B17 I32 Idiopathic BAD Control group
0.9038901601830663
666 B17 I33 Idiopathic BAD Control group
0.6273836765827613
667 B17 I34 Idiopathic BAD Control group
0.7116704805491991
668 B17 I35 Idiopathic BAD Control group
0.8253241800152555
669 B17 I36 Idiopathic BAD Control group
0.9963768115942029
670 B17 I37 Idiopathic BAD Control group
0.855072463768116
671 B20 B27 Idiopathic BAD Control group
0.5835240274599542
672 B20 B66 Idiopathic BAD Control group
0.8295194508009154
673 B20 B86 Idiopathic BAD Control group
0.6651411136536994
674 B20 B97 Idiopathic BAD Control group
0.8190312738367659
675 B20 B98 Idiopathic BAD Control group
0.7921434019832189
676 B20 B100 Idiopathic BAD Control group
0.9664378337147216
677 B20 B112 Idiopathic BAD Control group
0.631769641495042
678 B20 B115 Idiopathic BAD Control group
0.8356216628527842
679 B20 I1 Idiopathic BAD Control group
0.7568649885583524
680 B20 I3 Idiopathic BAD Control group
0.8426773455377574
681 B20 I6 Idiopathic BAD Control group
0.8001525553012967
682 B20 I8 Idiopathic BAD Control group
0.8257055682684973
683 B20 I10 Idiopathic BAD Control group
0.7591533180778032
684 B20 I11 Idiopathic BAD Control group
0.9088482074752098
685 B20 I13 Idiopathic BAD Control group
0.8451563691838292
686 B20 I15 Idiopathic BAD Control group
0.9057971014492754

687	B20	I17	Idiopathic BAD	Control group	0.6926010678871091
688	B20	I18	Idiopathic BAD	Control group	0.7986270022883295
689	B20	I19	Idiopathic BAD	Control group	0.7236842105263158
690	B20	I22	Idiopathic BAD	Control group	0.8405797101449275
691	B20	I23	Idiopathic BAD	Control group	0.7929061784897025
692	B20	I24	Idiopathic BAD	Control group	0.5072463768115942
693	B20	I25	Idiopathic BAD	Control group	0.706140350877193
694	B20	I26	Idiopathic BAD	Control group	0.761632341723875
695	B20	I27	Idiopathic BAD	Control group	0.8213196033562167
696	B20	I28	Idiopathic BAD	Control group	0.9279176201372997
697	B20	I29	Idiopathic BAD	Control group	0.851067887109077
698	B20	I30	Idiopathic BAD	Control group	0.8548817696414951
699	B20	I31	Idiopathic BAD	Control group	0.8676582761250954
700	B20	I32	Idiopathic BAD	Control group	0.8716628527841342
701	B20	I33	Idiopathic BAD	Control group	0.5192601067887109
702	B20	I34	Idiopathic BAD	Control group	0.6805873379099924
703	B20	I35	Idiopathic BAD	Control group	0.8291380625476735
704	B20	I36	Idiopathic BAD	Control group	0.9979023646071701
705	B20	I37	Idiopathic BAD	Control group	0.9439359267734554
706	B23	B27	Idiopathic BAD	Control group	0.7608695652173914
707	B23	B66	Idiopathic BAD	Control group	0.6479786422578184
708	B23	B86	Idiopathic BAD	Control group	0.8115942028985508
709	B23	B97	Idiopathic BAD	Control group	0.7028985507246377
710	B23	B98	Idiopathic BAD	Control group	0.7215865751334859
711	B23	B100	Idiopathic BAD	Control group	0.8630816170861938
712	B23	B112	Idiopathic BAD	Control group	0.8230358504958047
713	B23	B115	Idiopathic BAD	Control group	0.7644927536231884

714	B23	I1	Idiopathic BAD	Control group
0.7185354691075515
715	B23	I3	Idiopathic BAD	Control group
0.7990083905415714
716	B23	I6	Idiopathic BAD	Control group
0.6590389016018307
717	B23	I8	Idiopathic BAD	Control group
0.8064454614797865
718	B23	I10	Idiopathic BAD	Control group
0.8047292143401983
719	B23	I11	Idiopathic BAD	Control group
0.8808161708619374
720	B23	I13	Idiopathic BAD	Control group
0.7808924485125858
721	B23	I15	Idiopathic BAD	Control group
0.7561022120518688
722	B23	I17	Idiopathic BAD	Control group
0.839626239511823
723	B23	I18	Idiopathic BAD	Control group
0.7936689549961862
724	B23	I19	Idiopathic BAD	Control group
0.8995041952707856
725	B23	I22	Idiopathic BAD	Control group
0.8232265446224256
726	B23	I23	Idiopathic BAD	Control group
0.6823035850495804
727	B23	I24	Idiopathic BAD	Control group
0.8808161708619374
728	B23	I25	Idiopathic BAD	Control group
0.7988176964149504
729	B23	I26	Idiopathic BAD	Control group
0.7326468344774981
730	B23	I27	Idiopathic BAD	Control group
0.7151029748283753
731	B23	I28	Idiopathic BAD	Control group
0.8956903127383676
732	B23	I29	Idiopathic BAD	Control group
0.8731884057971014
733	B23	I30	Idiopathic BAD	Control group
0.7152936689549961
734	B23	I31	Idiopathic BAD	Control group
0.8935926773455377
735	B23	I32	Idiopathic BAD	Control group
0.8375286041189931
736	B23	I33	Idiopathic BAD	Control group
0.8373379099923722
737	B23	I34	Idiopathic BAD	Control group
0.700419527078566
738	B23	I35	Idiopathic BAD	Control group
0.7473302822273074
739	B23	I36	Idiopathic BAD	Control group
0.9935163996948894
740	B23	I37	Idiopathic BAD	Control group
0.844393592677345

741	B31	B27	Idiopathic BAD	Control group
0.7246376811594203
742	B31	B66	Idiopathic BAD	Control group
0.7890922959572845
743	B31	B86	Idiopathic BAD	Control group
0.8689931350114416
744	B31	B97	Idiopathic BAD	Control group
0.7795575896262396
745	B31	B98	Idiopathic BAD	Control group
0.6340579710144928
746	B31	B100	Idiopathic BAD	Control group
0.9010297482837528
747	B31	B112	Idiopathic BAD	Control group
0.860602593440122
748	B31	B115	Idiopathic BAD	Control group
0.6916475972540046
749	B31	I1	Idiopathic BAD	Control group
0.7303585049580473
750	B31	I3	Idiopathic BAD	Control group
0.830091533180778
751	B31	I6	Idiopathic BAD	Control group
0.778794813119756
752	B31	I8	Idiopathic BAD	Control group
0.8508771929824561
753	B31	I10	Idiopathic BAD	Control group
0.8573607932875668
754	B31	I11	Idiopathic BAD	Control group
0.8276125095347063
755	B31	I13	Idiopathic BAD	Control group
0.855072463768116
756	B31	I15	Idiopathic BAD	Control group
0.8070175438596491
757	B31	I17	Idiopathic BAD	Control group
0.8575514874141876
758	B31	I18	Idiopathic BAD	Control group
0.7278794813119756
759	B31	I19	Idiopathic BAD	Control group
0.9508009153318078
760	B31	I22	Idiopathic BAD	Control group
0.7578184591914569
761	B31	I23	Idiopathic BAD	Control group
0.7644927536231884
762	B31	I24	Idiopathic BAD	Control group
0.9250572082379863
763	B31	I25	Idiopathic BAD	Control group
0.841723874904653
764	B31	I26	Idiopathic BAD	Control group
0.8285659801678108
765	B31	I27	Idiopathic BAD	Control group
0.7948131197559115
766	B31	I28	Idiopathic BAD	Control group
0.7192982456140351
767	B31	I29	Idiopathic BAD	Control group
0.9138062547673532

768	B31	I30	Idiopathic BAD	Control group	0.812929061784897
769	B31	I31	Idiopathic BAD	Control group	0.8541189931350115
770	B31	I32	Idiopathic BAD	Control group	0.8598398169336384
771	B31	I33	Idiopathic BAD	Control group	0.9036994660564455
772	B31	I34	Idiopathic BAD	Control group	0.8094965675057209
773	B31	I35	Idiopathic BAD	Control group	0.7929061784897025
774	B31	I36	Idiopathic BAD	Control group	0.8913043478260869
775	B31	I37	Idiopathic BAD	Control group	0.7475209763539283
776	B35	B27	Idiopathic BAD	Control group	0.9692982456140351
777	B35	B66	Idiopathic BAD	Control group	0.9262013729977117
778	B35	B86	Idiopathic BAD	Control group	0.9815026697177727
779	B35	B97	Idiopathic BAD	Control group	0.9309687261632341
780	B35	B98	Idiopathic BAD	Control group	0.9319221967963387
781	B35	B100	Idiopathic BAD	Control group	0.881578947368421
782	B35	B112	Idiopathic BAD	Control group	0.967581998474447
783	B35	B115	Idiopathic BAD	Control group	0.9803585049580473
784	B35	I1	Idiopathic BAD	Control group	0.9801678108314263
785	B35	I3	Idiopathic BAD	Control group	0.8321891685736079
786	B35	I6	Idiopathic BAD	Control group	0.9750190694126621
787	B35	I8	Idiopathic BAD	Control group	0.9912280701754386
788	B35	I10	Idiopathic BAD	Control group	0.9492753623188406
789	B35	I11	Idiopathic BAD	Control group	0.9570938215102975
790	B35	I13	Idiopathic BAD	Control group	0.9189549961861174
791	B35	I15	Idiopathic BAD	Control group	0.9834096109839817
792	B35	I17	Idiopathic BAD	Control group	0.9921815408085431
793	B35	I18	Idiopathic BAD	Control group	0.9265827612509535
794	B35	I19	Idiopathic BAD	Control group	0.9881769641495042

795	B35	I22	Idiopathic BAD	Control group
0.9509916094584286
796	B35	I23	Idiopathic BAD	Control group
0.9603356216628528
797	B35	I24	Idiopathic BAD	Control group
0.9815026697177727
798	B35	I25	Idiopathic BAD	Control group
0.919908466819222
799	B35	I26	Idiopathic BAD	Control group
0.9284897025171625
800	B35	I27	Idiopathic BAD	Control group
0.8571700991609459
801	B35	I28	Idiopathic BAD	Control group
0.6332951945080092
802	B35	I29	Idiopathic BAD	Control group
0.969488939740656
803	B35	I30	Idiopathic BAD	Control group
0.9494660564454614
804	B35	I31	Idiopathic BAD	Control group
0.9889397406559878
805	B35	I32	Idiopathic BAD	Control group
0.9508009153318078
806	B35	I33	Idiopathic BAD	Control group
0.954042715484363
807	B35	I34	Idiopathic BAD	Control group
0.9662471395881007
808	B35	I35	Idiopathic BAD	Control group
0.8634630053394355
809	B35	I36	Idiopathic BAD	Control group
0.9950419527078566
810	B35	I37	Idiopathic BAD	Control group
0.9189549961861174
811	B39	B27	Idiopathic BAD	Control group
0.8522120518688024
812	B39	B66	Idiopathic BAD	Control group
0.8678489702517163
813	B39	B86	Idiopathic BAD	Control group
0.9014111365369947
814	B39	B97	Idiopathic BAD	Control group
0.8117848970251716
815	B39	B98	Idiopathic BAD	Control group
0.7599160945842868
816	B39	B100	Idiopathic BAD	Control group
0.9439359267734554
817	B39	B112	Idiopathic BAD	Control group
0.8918764302059496
818	B39	B115	Idiopathic BAD	Control group
0.8615560640732265
819	B39	I1	Idiopathic BAD	Control group
0.8667048054919908
820	B39	I3	Idiopathic BAD	Control group
0.8314263920671243
821	B39	I6	Idiopathic BAD	Control group
0.7526697177726926

822	B39	I8	Idiopathic BAD	Control group	0.8960717009916095
823	B39	I10	Idiopathic BAD	Control group	0.8750953470633105
824	B39	I11	Idiopathic BAD	Control group	0.7986270022883295
825	B39	I13	Idiopathic BAD	Control group	0.8655606407322655
826	B39	I15	Idiopathic BAD	Control group	0.9017925247902364
827	B39	I17	Idiopathic BAD	Control group	0.8956903127383676
828	B39	I18	Idiopathic BAD	Control group	0.7982456140350878
829	B39	I19	Idiopathic BAD	Control group	0.9477498093058734
830	B39	I22	Idiopathic BAD	Control group	0.7965293668954996
831	B39	I23	Idiopathic BAD	Control group	0.847254004576659
832	B39	I24	Idiopathic BAD	Control group	0.9252479023646072
833	B39	I25	Idiopathic BAD	Control group	0.8735697940503433
834	B39	I26	Idiopathic BAD	Control group	0.8308543096872616
835	B39	I27	Idiopathic BAD	Control group	0.7690694126620901
836	B39	I28	Idiopathic BAD	Control group	0.8531655225019069
837	B39	I29	Idiopathic BAD	Control group	0.9319221967963387
838	B39	I30	Idiopathic BAD	Control group	0.8464912280701754
839	B39	I31	Idiopathic BAD	Control group	0.8747139588100686
840	B39	I32	Idiopathic BAD	Control group	0.9031273836765827
841	B39	I33	Idiopathic BAD	Control group	0.8991228070175439
842	B39	I34	Idiopathic BAD	Control group	0.8468726163234173
843	B39	I35	Idiopathic BAD	Control group	0.8371472158657514
844	B39	I36	Idiopathic BAD	Control group	0.9660564454614798
845	B39	I37	Idiopathic BAD	Control group	0.8699466056445462
846	B43	B27	Idiopathic BAD	Control group	0.715675057208238
847	B43	B66	Idiopathic BAD	Control group	0.9126620900076278
848	B43	B86	Idiopathic BAD	Control group	0.7034706331045004

849	B43	B97	Idiopathic BAD	Control group
0.8384820747520977				
850	B43	B98	Idiopathic BAD	Control group
0.8668954996186118				
851	B43	B100	Idiopathic BAD	Control group
0.9706331045003814				
852	B43	B112	Idiopathic BAD	Control group
0.7837528604118993				
853	B43	B115	Idiopathic BAD	Control group
0.8663234172387491				
854	B43	I1	Idiopathic BAD	Control group
0.8020594965675057				
855	B43	I3	Idiopathic BAD	Control group
0.8144546147978642				
856	B43	I6	Idiopathic BAD	Control group
0.8941647597254004				
857	B43	I8	Idiopathic BAD	Control group
0.782608695652174				
858	B43	I10	Idiopathic BAD	Control group
0.7585812356979404				
859	B43	I11	Idiopathic BAD	Control group
0.9277269260106789				
860	B43	I13	Idiopathic BAD	Control group
0.6922196796338673				
861	B43	I15	Idiopathic BAD	Control group
0.8270404271548436				
862	B43	I17	Idiopathic BAD	Control group
0.6626620900076278				
863	B43	I18	Idiopathic BAD	Control group
0.7784134248665141				
864	B43	I19	Idiopathic BAD	Control group
0.7486651411136537				
865	B43	I22	Idiopathic BAD	Control group
0.8112128146453089				
866	B43	I23	Idiopathic BAD	Control group
0.835812356979405				
867	B43	I24	Idiopathic BAD	Control group
0.6027841342486652				
868	B43	I25	Idiopathic BAD	Control group
0.7885202135774219				
869	B43	I26	Idiopathic BAD	Control group
0.7501906941266209				
870	B43	I27	Idiopathic BAD	Control group
0.8232265446224256				
871	B43	I28	Idiopathic BAD	Control group
0.8951182303585049				
872	B43	I29	Idiopathic BAD	Control group
0.8121662852784134				
873	B43	I30	Idiopathic BAD	Control group
0.8445842868039665				
874	B43	I31	Idiopathic BAD	Control group
0.8104500381388253				
875	B43	I32	Idiopathic BAD	Control group
0.7889016018306636				

876	B43	I33	Idiopathic BAD	Control group
0.6426392067124332
877	B43	I34	Idiopathic BAD	Control group
0.7484744469870328
878	B43	I35	Idiopathic BAD	Control group
0.8241800152555301
879	B43	I36	Idiopathic BAD	Control group
0.9977116704805492
880	B43	I37	Idiopathic BAD	Control group
0.9424103737604882
881	B47	B27	Idiopathic BAD	Control group
0.7854691075514875
882	B47	B66	Idiopathic BAD	Control group
0.7936689549961862
883	B47	B86	Idiopathic BAD	Control group
0.8056826849733029
884	B47	B97	Idiopathic BAD	Control group
0.7755530129672006
885	B47	B98	Idiopathic BAD	Control group
0.7425629290617849
886	B47	B100	Idiopathic BAD	Control group
0.9515636918382914
887	B47	B112	Idiopathic BAD	Control group
0.833905415713196
888	B47	B115	Idiopathic BAD	Control group
0.736651411136537
889	B47	I1	Idiopathic BAD	Control group
0.6842105263157895
890	B47	I3	Idiopathic BAD	Control group
0.8546910755148741
891	B47	I6	Idiopathic BAD	Control group
0.6884057971014492
892	B47	I8	Idiopathic BAD	Control group
0.8421052631578947
893	B47	I10	Idiopathic BAD	Control group
0.8585049580472921
894	B47	I11	Idiopathic BAD	Control group
0.8926392067124332
895	B47	I13	Idiopathic BAD	Control group
0.7837528604118993
896	B47	I15	Idiopathic BAD	Control group
0.8516399694889397
897	B47	I17	Idiopathic BAD	Control group
0.8686117467581999
898	B47	I18	Idiopathic BAD	Control group
0.7925247902364607
899	B47	I19	Idiopathic BAD	Control group
0.8689931350114416
900	B47	I22	Idiopathic BAD	Control group
0.843440122044241
901	B47	I23	Idiopathic BAD	Control group
0.7135774218154081
902	B47	I24	Idiopathic BAD	Control group
0.8642257818459191

903	B47	I25	Idiopathic BAD	Control group
0.7812738367658276
904	B47	I26	Idiopathic BAD	Control group
0.669908466819222
905	B47	I27	Idiopathic BAD	Control group
0.7374141876430206
906	B47	I28	Idiopathic BAD	Control group
0.9246758199847445
907	B47	I29	Idiopathic BAD	Control group
0.8371472158657514
908	B47	I30	Idiopathic BAD	Control group
0.8077803203661327
909	B47	I31	Idiopathic BAD	Control group
0.8710907704042715
910	B47	I32	Idiopathic BAD	Control group
0.9437452326468345
911	B47	I33	Idiopathic BAD	Control group
0.8119755911517925
912	B47	I34	Idiopathic BAD	Control group
0.7745995423340961
913	B47	I35	Idiopathic BAD	Control group
0.8377192982456141
914	B47	I36	Idiopathic BAD	Control group
0.9855072463768116
915	B47	I37	Idiopathic BAD	Control group
0.8983600305110603
916	B48	B27	Idiopathic BAD	Control group
0.8731884057971014
917	B48	B66	Idiopathic BAD	Control group
0.8703279938977879
918	B48	B86	Idiopathic BAD	Control group
0.9458428680396643
919	B48	B97	Idiopathic BAD	Control group
0.8872997711670481
920	B48	B98	Idiopathic BAD	Control group
0.8165522501906941
921	B48	B100	Idiopathic BAD	Control group
0.8842486651411137
922	B48	B112	Idiopathic BAD	Control group
0.9336384439359268
923	B48	B115	Idiopathic BAD	Control group
0.7797482837528604
924	B48	I1	Idiopathic BAD	Control group
0.8543096872616324
925	B48	I3	Idiopathic BAD	Control group
0.7494279176201373
926	B48	I6	Idiopathic BAD	Control group
0.8342868039664378
927	B48	I8	Idiopathic BAD	Control group
0.967391304347826
928	B48	I10	Idiopathic BAD	Control group
0.9059877955758963
929	B48	I11	Idiopathic BAD	Control group
0.8756674294431731

930 B48 I13 Idiopathic BAD Control group
0.9347826086956522
931 B48 I15 Idiopathic BAD Control group
0.8642257818459191
932 B48 I17 Idiopathic BAD Control group
0.9620518688024409
933 B48 I18 Idiopathic BAD Control group
0.8800533943554538
934 B48 I19 Idiopathic BAD Control group
0.9809305873379099
935 B48 I22 Idiopathic BAD Control group
0.9262013729977117
936 B48 I23 Idiopathic BAD Control group
0.9021739130434783
937 B48 I24 Idiopathic BAD Control group
0.9778794813119756
938 B48 I25 Idiopathic BAD Control group
0.8840579710144928
939 B48 I26 Idiopathic BAD Control group
0.9016018306636155
940 B48 I27 Idiopathic BAD Control group
0.7734553775743707
941 B48 I28 Idiopathic BAD Control group
0.6767734553775744
942 B48 I29 Idiopathic BAD Control group
0.9685354691075515
943 B48 I30 Idiopathic BAD Control group
0.7713577421815409
944 B48 I31 Idiopathic BAD Control group
0.9221967963386728
945 B48 I32 Idiopathic BAD Control group
0.9347826086956522
946 B48 I33 Idiopathic BAD Control group
0.9399313501144165
947 B48 I34 Idiopathic BAD Control group
0.9069412662090007
948 B48 I35 Idiopathic BAD Control group
0.8729977116704806
949 B48 I36 Idiopathic BAD Control group
0.9868421052631579
950 B48 I37 Idiopathic BAD Control group
0.898741418764302
951 B49 B27 Idiopathic BAD Control group
0.7890922959572845
952 B49 B66 Idiopathic BAD Control group
0.791952707856598
953 B49 B86 Idiopathic BAD Control group
0.8270404271548436
954 B49 B97 Idiopathic BAD Control group
0.6359649122807017
955 B49 B98 Idiopathic BAD Control group
0.7526697177726926
956 B49 B100 Idiopathic BAD Control group
0.940884820747521

957	B49	B112	Idiopathic BAD	Control group	0.8867276887871853
958	B49	B115	Idiopathic BAD	Control group	0.778604118993135
959	B49	I1	Idiopathic BAD	Control group	0.8003432494279176
960	B49	I3	Idiopathic BAD	Control group	0.6578947368421053
961	B49	I6	Idiopathic BAD	Control group	0.7812738367658276
962	B49	I8	Idiopathic BAD	Control group	0.8146453089244852
963	B49	I10	Idiopathic BAD	Control group	0.7639206712433257
964	B49	I11	Idiopathic BAD	Control group	0.7263539282990084
965	B49	I13	Idiopathic BAD	Control group	0.7612509534706331
966	B49	I15	Idiopathic BAD	Control group	0.799771167048055
967	B49	I17	Idiopathic BAD	Control group	0.7950038138825324
968	B49	I18	Idiopathic BAD	Control group	0.7711670480549199
969	B49	I19	Idiopathic BAD	Control group	0.849160945842868
970	B49	I22	Idiopathic BAD	Control group	0.7906178489702517
971	B49	I23	Idiopathic BAD	Control group	0.8068268497330282
972	B49	I24	Idiopathic BAD	Control group	0.8627002288329519
973	B49	I25	Idiopathic BAD	Control group	0.6538901601830663
974	B49	I26	Idiopathic BAD	Control group	0.8485888634630053
975	B49	I27	Idiopathic BAD	Control group	0.7911899313501144
976	B49	I28	Idiopathic BAD	Control group	0.7305491990846682
977	B49	I29	Idiopathic BAD	Control group	0.7458047292143402
978	B49	I30	Idiopathic BAD	Control group	0.8924485125858124
979	B49	I31	Idiopathic BAD	Control group	0.7877574370709383
980	B49	I32	Idiopathic BAD	Control group	0.7982456140350878
981	B49	I33	Idiopathic BAD	Control group	0.8030129672006102
982	B49	I34	Idiopathic BAD	Control group	0.8846300533943554
983	B49	I35	Idiopathic BAD	Control group	0.6725781845919145

984	B49	I36	Idiopathic BAD	Control group	0.9935163996948894
985	B49	I37	Idiopathic BAD	Control group	0.8960717009916095
986	B53	B27	Idiopathic BAD	Control group	0.8205568268497331
987	B53	B66	Idiopathic BAD	Control group	0.8136918382913806
988	B53	B86	Idiopathic BAD	Control group	0.8051106025934401
989	B53	B97	Idiopathic BAD	Control group	0.8194126620900076
990	B53	B98	Idiopathic BAD	Control group	0.8251334858886347
991	B53	B100	Idiopathic BAD	Control group	0.9126620900076278
992	B53	B112	Idiopathic BAD	Control group	0.8699466056445462
993	B53	B115	Idiopathic BAD	Control group	0.8518306636155606
994	B53	I1	Idiopathic BAD	Control group	0.8220823798627003
995	B53	I3	Idiopathic BAD	Control group	0.8699466056445462
996	B53	I6	Idiopathic BAD	Control group	0.7446605644546148
997	B53	I8	Idiopathic BAD	Control group	0.8607932875667429
998	B53	I10	Idiopathic BAD	Control group	0.8825324180015256
999	B53	I11	Idiopathic BAD	Control group	0.9016018306636155
1000	B53	I13	Idiopathic BAD	Control group	0.8245614035087719
1001	B53	I15	Idiopathic BAD	Control group	0.8422959572845157
1002	B53	I17	Idiopathic BAD	Control group	0.8638443935926774
1003	B53	I18	Idiopathic BAD	Control group	0.8094965675057209
1004	B53	I19	Idiopathic BAD	Control group	0.8485888634630053
1005	B53	I22	Idiopathic BAD	Control group	0.8453470633104501
1006	B53	I23	Idiopathic BAD	Control group	0.7120518688024409
1007	B53	I24	Idiopathic BAD	Control group	0.8823417238749046
1008	B53	I25	Idiopathic BAD	Control group	0.8304729214340199
1009	B53	I26	Idiopathic BAD	Control group	0.8466819221967964
1010	B53	I27	Idiopathic BAD	Control group	0.7618230358504958

1011	B53	I28	Idiopathic BAD	Control group	0.937070938215103
1012	B53	I29	Idiopathic BAD	Control group	0.8495423340961098
1013	B53	I30	Idiopathic BAD	Control group	0.7827993897787948
1014	B53	I31	Idiopathic BAD	Control group	0.8703279938977879
1015	B53	I32	Idiopathic BAD	Control group	0.9506102212051869
1016	B53	I33	Idiopathic BAD	Control group	0.8268497330282227
1017	B53	I34	Idiopathic BAD	Control group	0.8274218154080855
1018	B53	I35	Idiopathic BAD	Control group	0.8157894736842105
1019	B53	I36	Idiopathic BAD	Control group	0.9935163996948894
1020	B53	I37	Idiopathic BAD	Control group	0.8958810068649885
1021	B54	B27	Idiopathic BAD	Control group	0.8106407322654462
1022	B54	B66	Idiopathic BAD	Control group	0.8686117467581999
1023	B54	B86	Idiopathic BAD	Control group	0.5983981693363845
1024	B54	B97	Idiopathic BAD	Control group	0.8901601830663616
1025	B54	B98	Idiopathic BAD	Control group	0.8522120518688024
1026	B54	B100	Idiopathic BAD	Control group	0.9374523264683448
1027	B54	B112	Idiopathic BAD	Control group	0.6058352402745996
1028	B54	B115	Idiopathic BAD	Control group	0.8991228070175439
1029	B54	I1	Idiopathic BAD	Control group	0.8360030511060259
1030	B54	I3	Idiopathic BAD	Control group	0.9233409610983981
1031	B54	I6	Idiopathic BAD	Control group	0.816742944317315
1032	B54	I8	Idiopathic BAD	Control group	0.9178108314263921
1033	B54	I10	Idiopathic BAD	Control group	0.898932112890923
1034	B54	I11	Idiopathic BAD	Control group	0.9500381388253242
1035	B54	I13	Idiopathic BAD	Control group	0.8966437833714722
1036	B54	I15	Idiopathic BAD	Control group	0.9364988558352403
1037	B54	I17	Idiopathic BAD	Control group	0.7284515636918383

1038 B54 I18 Idiopathic BAD Control group
0.9216247139588101
1039 B54 I19 Idiopathic BAD Control group
0.7172006102212052
1040 B54 I22 Idiopathic BAD Control group
0.9136155606407322
1041 B54 I23 Idiopathic BAD Control group
0.8123569794050344
1042 B54 I24 Idiopathic BAD Control group
0.6254767353165522
1043 B54 I25 Idiopathic BAD Control group
0.8621281464530892
1044 B54 I26 Idiopathic BAD Control group
0.8342868039664378
1045 B54 I27 Idiopathic BAD Control group
0.8827231121281465
1046 B54 I28 Idiopathic BAD Control group
0.9609077040427155
1047 B54 I29 Idiopathic BAD Control group
0.9210526315789473
1048 B54 I30 Idiopathic BAD Control group
0.9128527841342486
1049 B54 I31 Idiopathic BAD Control group
0.8600305110602593
1050 B54 I32 Idiopathic BAD Control group
0.9557589626239512
1051 B54 I33 Idiopathic BAD Control group
0.6235697940503433
1052 B54 I34 Idiopathic BAD Control group
0.7759344012204424
1053 B54 I35 Idiopathic BAD Control group
0.8838672768878718
1054 B54 I36 Idiopathic BAD Control group
0.9984744469870328
1055 B54 I37 Idiopathic BAD Control group
0.9544241037376049
1056 B55 B27 Idiopathic BAD Control group
0.8032036613272311
1057 B55 B66 Idiopathic BAD Control group
0.8503051106025934
1058 B55 B86 Idiopathic BAD Control group
0.6266209000762777
1059 B55 B97 Idiopathic BAD Control group
0.8508771929824561
1060 B55 B98 Idiopathic BAD Control group
0.9105644546147978
1061 B55 B100 Idiopathic BAD Control group
0.9845537757437071
1062 B55 B112 Idiopathic BAD Control group
0.6624713958810069
1063 B55 B115 Idiopathic BAD Control group
0.8218916857360793
1064 B55 I1 Idiopathic BAD Control group
0.8844393592677345

1065	B55	I3	Idiopathic BAD	Control group	0.948512585812357
1066	B55	I6	Idiopathic BAD	Control group	0.8371472158657514
1067	B55	I8	Idiopathic BAD	Control group	0.9214340198321892
1068	B55	I10	Idiopathic BAD	Control group	0.9555682684973302
1069	B55	I11	Idiopathic BAD	Control group	0.9353546910755148
1070	B55	I13	Idiopathic BAD	Control group	0.8577421815408085
1071	B55	I15	Idiopathic BAD	Control group	0.8813882532418001
1072	B55	I17	Idiopathic BAD	Control group	0.7400839054157132
1073	B55	I18	Idiopathic BAD	Control group	0.8710907704042715
1074	B55	I19	Idiopathic BAD	Control group	0.7418001525553013
1075	B55	I22	Idiopathic BAD	Control group	0.9054157131960335
1076	B55	I23	Idiopathic BAD	Control group	0.915903890160183
1077	B55	I24	Idiopathic BAD	Control group	0.6573226544622426
1078	B55	I25	Idiopathic BAD	Control group	0.88558352402746
1079	B55	I26	Idiopathic BAD	Control group	0.8865369946605645
1080	B55	I27	Idiopathic BAD	Control group	0.8585049580472921
1081	B55	I28	Idiopathic BAD	Control group	0.9061784897025171
1082	B55	I29	Idiopathic BAD	Control group	0.9300152555301296
1083	B55	I30	Idiopathic BAD	Control group	0.855072463768116
1084	B55	I31	Idiopathic BAD	Control group	0.9437452326468345
1085	B55	I32	Idiopathic BAD	Control group	0.9576659038901602
1086	B55	I33	Idiopathic BAD	Control group	0.6462623951182304
1087	B55	I34	Idiopathic BAD	Control group	0.6493135011441648
1088	B55	I35	Idiopathic BAD	Control group	0.9496567505720824
1089	B55	I36	Idiopathic BAD	Control group	0.994279176201373
1090	B55	I37	Idiopathic BAD	Control group	0.9683447749809306
1091	B59	B27	Idiopathic BAD	Control group	0.9754004576659039

1092	B59	B66	Idiopathic BAD	Control group	0.9710144927536232
1093	B59	B86	Idiopathic BAD	Control group	0.919717772692601
1094	B59	B97	Idiopathic BAD	Control group	0.9681540808543097
1095	B59	B98	Idiopathic BAD	Control group	0.9767353165522502
1096	B59	B100	Idiopathic BAD	Control group	0.9155225019069413
1097	B59	B112	Idiopathic BAD	Control group	0.9809305873379099
1098	B59	B115	Idiopathic BAD	Control group	0.9839816933638444
1099	B59	I1	Idiopathic BAD	Control group	0.9799771167048055
1100	B59	I3	Idiopathic BAD	Control group	0.9929443173150267
1101	B59	I6	Idiopathic BAD	Control group	0.9563310450038138
1102	B59	I8	Idiopathic BAD	Control group	0.9666285278413425
1103	B59	I10	Idiopathic BAD	Control group	0.9816933638443935
1104	B59	I11	Idiopathic BAD	Control group	0.9706331045003814
1105	B59	I13	Idiopathic BAD	Control group	0.9641495041952708
1106	B59	I15	Idiopathic BAD	Control group	0.8531655225019069
1107	B59	I17	Idiopathic BAD	Control group	0.9704424103737604
1108	B59	I18	Idiopathic BAD	Control group	0.9300152555301296
1109	B59	I19	Idiopathic BAD	Control group	0.9563310450038138
1110	B59	I22	Idiopathic BAD	Control group	0.8686117467581999
1111	B59	I23	Idiopathic BAD	Control group	0.9649122807017544
1112	B59	I24	Idiopathic BAD	Control group	0.9612890922959573
1113	B59	I25	Idiopathic BAD	Control group	0.9725400457665904
1114	B59	I26	Idiopathic BAD	Control group	0.9927536231884058
1115	B59	I27	Idiopathic BAD	Control group	0.986651411136537
1116	B59	I28	Idiopathic BAD	Control group	0.9357360793287567
1117	B59	I29	Idiopathic BAD	Control group	0.9837909992372235
1118	B59	I30	Idiopathic BAD	Control group	0.975209763539283

1119	B59	I31	Idiopathic BAD	Control group	0.9973302822273074
1120	B59	I32	Idiopathic BAD	Control group	0.9887490465293669
1121	B59	I33	Idiopathic BAD	Control group	0.9755911517925248
1122	B59	I34	Idiopathic BAD	Control group	0.9803585049580473
1123	B59	I35	Idiopathic BAD	Control group	0.9889397406559878
1124	B59	I36	Idiopathic BAD	Control group	0.8011060259344012
1125	B59	I37	Idiopathic BAD	Control group	0.8012967200610221
1126	B70	B27	Idiopathic BAD	Control group	0.9235316552250191
1127	B70	B66	Idiopathic BAD	Control group	0.7711670480549199
1128	B70	B86	Idiopathic BAD	Control group	0.8844393592677345
1129	B70	B97	Idiopathic BAD	Control group	0.8157894736842105
1130	B70	B98	Idiopathic BAD	Control group	0.8972158657513348
1131	B70	B100	Idiopathic BAD	Control group	0.9090389016018307
1132	B70	B112	Idiopathic BAD	Control group	0.9138062547673532
1133	B70	B115	Idiopathic BAD	Control group	0.881769641495042
1134	B70	I1	Idiopathic BAD	Control group	0.8695652173913043
1135	B70	I3	Idiopathic BAD	Control group	0.7961479786422578
1136	B70	I6	Idiopathic BAD	Control group	0.7580091533180778
1137	B70	I8	Idiopathic BAD	Control group	0.9345919145690312
1138	B70	I10	Idiopathic BAD	Control group	0.8520213577421816
1139	B70	I11	Idiopathic BAD	Control group	0.9487032799389779
1140	B70	I13	Idiopathic BAD	Control group	0.9218154080854309
1141	B70	I15	Idiopathic BAD	Control group	0.8142639206712433
1142	B70	I17	Idiopathic BAD	Control group	0.9168573607932876
1143	B70	I18	Idiopathic BAD	Control group	0.7435163996948894
1144	B70	I19	Idiopathic BAD	Control group	0.9208619374523265
1145	B70	I22	Idiopathic BAD	Control group	0.9069412662090007

1146 B70 I23 Idiopathic BAD Control group
0.7688787185354691
1147 B70 I24 Idiopathic BAD Control group
0.9075133485888635
1148 B70 I25 Idiopathic BAD Control group
0.8935926773455377
1149 B70 I26 Idiopathic BAD Control group
0.7690694126620901
1150 B70 I27 Idiopathic BAD Control group
0.7763157894736842
1151 B70 I28 Idiopathic BAD Control group
0.9437452326468345
1152 B70 I29 Idiopathic BAD Control group
0.8707093821510298
1153 B70 I30 Idiopathic BAD Control group
0.7292143401983219
1154 B70 I31 Idiopathic BAD Control group
0.9647215865751335
1155 B70 I32 Idiopathic BAD Control group
0.9845537757437071
1156 B70 I33 Idiopathic BAD Control group
0.8972158657513348
1157 B70 I34 Idiopathic BAD Control group
0.8203661327231121
1158 B70 I35 Idiopathic BAD Control group
0.894927536231884
1159 B70 I36 Idiopathic BAD Control group
0.9855072463768116
1160 B70 I37 Idiopathic BAD Control group
0.9527078565980168
1161 B74 B27 Idiopathic BAD Control group
0.7951945080091534
1162 B74 B66 Idiopathic BAD Control group
0.7814645308924485
1163 B74 B86 Idiopathic BAD Control group
0.8516399694889397
1164 B74 B97 Idiopathic BAD Control group
0.6742944317315027
1165 B74 B98 Idiopathic BAD Control group
0.759534706331045
1166 B74 B100 Idiopathic BAD Control group
0.9233409610983981
1167 B74 B112 Idiopathic BAD Control group
0.8234172387490465
1168 B74 B115 Idiopathic BAD Control group
0.7625858123569794
1169 B74 I1 Idiopathic BAD Control group
0.7206331045003814
1170 B74 I3 Idiopathic BAD Control group
0.889397406559878
1171 B74 I6 Idiopathic BAD Control group
0.7690694126620901
1172 B74 I8 Idiopathic BAD Control group
0.8119755911517925

1173 B74 I10 Idiopathic BAD Control group
0.9012204424103738
1174 B74 I11 Idiopathic BAD Control group
0.8945461479786423
1175 B74 I13 Idiopathic BAD Control group
0.8260869565217391
1176 B74 I15 Idiopathic BAD Control group
0.9004576659038902
1177 B74 I17 Idiopathic BAD Control group
0.8672768878718535
1178 B74 I18 Idiopathic BAD Control group
0.8230358504958047
1179 B74 I19 Idiopathic BAD Control group
0.9147597254004577
1180 B74 I22 Idiopathic BAD Control group
0.8257055682684973
1181 B74 I23 Idiopathic BAD Control group
0.8316170861937452
1182 B74 I24 Idiopathic BAD Control group
0.914187643020595
1183 B74 I25 Idiopathic BAD Control group
0.8203661327231121
1184 B74 I26 Idiopathic BAD Control group
0.8577421815408085
1185 B74 I27 Idiopathic BAD Control group
0.88558352402746
1186 B74 I28 Idiopathic BAD Control group
0.8735697940503433
1187 B74 I29 Idiopathic BAD Control group
0.8716628527841342
1188 B74 I30 Idiopathic BAD Control group
0.8710907704042715
1189 B74 I31 Idiopathic BAD Control group
0.8996948893974066
1190 B74 I32 Idiopathic BAD Control group
0.9324942791762014
1191 B74 I33 Idiopathic BAD Control group
0.9054157131960335
1192 B74 I34 Idiopathic BAD Control group
0.876048817696415
1193 B74 I35 Idiopathic BAD Control group
0.8592677345537757
1194 B74 I36 Idiopathic BAD Control group
0.9971395881006865
1195 B74 I37 Idiopathic BAD Control group
0.8575514874141876
1196 B77 B27 Idiopathic BAD Control group
0.7753623188405797
1197 B77 B66 Idiopathic BAD Control group
0.8089244851258581
1198 B77 B86 Idiopathic BAD Control group
0.8051106025934401
1199 B77 B97 Idiopathic BAD Control group
0.7677345537757437

1200 B77 B98 Idiopathic BAD Control group
0.8100686498855835
1201 B77 B100 Idiopathic BAD Control group
0.9052250190694127
1202 B77 B112 Idiopathic BAD Control group
0.8524027459954233
1203 B77 B115 Idiopathic BAD Control group
0.7219679633867276
1204 B77 I1 Idiopathic BAD Control group
0.8028222730739893
1205 B77 I3 Idiopathic BAD Control group
0.8617467581998475
1206 B77 I6 Idiopathic BAD Control group
0.6748665141113653
1207 B77 I8 Idiopathic BAD Control group
0.8243707093821511
1208 B77 I10 Idiopathic BAD Control group
0.8585049580472921
1209 B77 I11 Idiopathic BAD Control group
0.9067505720823799
1210 B77 I13 Idiopathic BAD Control group
0.7936689549961862
1211 B77 I15 Idiopathic BAD Control group
0.8483981693363845
1212 B77 I17 Idiopathic BAD Control group
0.8716628527841342
1213 B77 I18 Idiopathic BAD Control group
0.7555301296720061
1214 B77 I19 Idiopathic BAD Control group
0.833905415713196
1215 B77 I22 Idiopathic BAD Control group
0.8125476735316552
1216 B77 I23 Idiopathic BAD Control group
0.8133104500381388
1217 B77 I24 Idiopathic BAD Control group
0.868230358504958
1218 B77 I25 Idiopathic BAD Control group
0.791952707856598
1219 B77 I26 Idiopathic BAD Control group
0.7564836003051106
1220 B77 I27 Idiopathic BAD Control group
0.7063310450038138
1221 B77 I28 Idiopathic BAD Control group
0.9294431731502669
1222 B77 I29 Idiopathic BAD Control group
0.8438215102974829
1223 B77 I30 Idiopathic BAD Control group
0.8039664378337147
1224 B77 I31 Idiopathic BAD Control group
0.910373760488177
1225 B77 I32 Idiopathic BAD Control group
0.9279176201372997
1226 B77 I33 Idiopathic BAD Control group
0.7955758962623951

1227 B77 I34 Idiopathic BAD Control group
0.7784134248665141
1228 B77 I35 Idiopathic BAD Control group
0.8649885583524027
1229 B77 I36 Idiopathic BAD Control group
0.9891304347826086
1230 B77 I37 Idiopathic BAD Control group
0.9105644546147978
1231 B81 B27 Idiopathic BAD Control group
0.8104500381388253
1232 B81 B66 Idiopathic BAD Control group
0.7610602593440122
1233 B81 B86 Idiopathic BAD Control group
0.8068268497330282
1234 B81 B97 Idiopathic BAD Control group
0.7154843630816171
1235 B81 B98 Idiopathic BAD Control group
0.8381006864988558
1236 B81 B100 Idiopathic BAD Control group
0.9393592677345538
1237 B81 B112 Idiopathic BAD Control group
0.8771929824561403
1238 B81 B115 Idiopathic BAD Control group
0.8321891685736079
1239 B81 I1 Idiopathic BAD Control group
0.776697177726926
1240 B81 I3 Idiopathic BAD Control group
0.7812738367658276
1241 B81 I6 Idiopathic BAD Control group
0.8037757437070938
1242 B81 I8 Idiopathic BAD Control group
0.8636536994660564
1243 B81 I10 Idiopathic BAD Control group
0.8981693363844394
1244 B81 I11 Idiopathic BAD Control group
0.8426773455377574
1245 B81 I13 Idiopathic BAD Control group
0.8205568268497331
1246 B81 I15 Idiopathic BAD Control group
0.9056064073226545
1247 B81 I17 Idiopathic BAD Control group
0.8327612509534706
1248 B81 I18 Idiopathic BAD Control group
0.7829900839054157
1249 B81 I19 Idiopathic BAD Control group
0.8930205949656751
1250 B81 I22 Idiopathic BAD Control group
0.8516399694889397
1251 B81 I23 Idiopathic BAD Control group
0.7673531655225019
1252 B81 I24 Idiopathic BAD Control group
0.8638443935926774
1253 B81 I25 Idiopathic BAD Control group
0.7467581998474447

1254	B81	I26	Idiopathic BAD	Control group	0.8066361556064073
1255	B81	I27	Idiopathic BAD	Control group	0.7877574370709383
1256	B81	I28	Idiopathic BAD	Control group	0.6531273836765827
1257	B81	I29	Idiopathic BAD	Control group	0.799771167048055
1258	B81	I30	Idiopathic BAD	Control group	0.7993897787948131
1259	B81	I31	Idiopathic BAD	Control group	0.8508771929824561
1260	B81	I32	Idiopathic BAD	Control group	0.8710907704042715
1261	B81	I33	Idiopathic BAD	Control group	0.8745232646834478
1262	B81	I34	Idiopathic BAD	Control group	0.9061784897025171
1263	B81	I35	Idiopathic BAD	Control group	0.7486651411136537
1264	B81	I36	Idiopathic BAD	Control group	0.990465293668955
1265	B81	I37	Idiopathic BAD	Control group	0.8827231121281465
1266	B84	B27	Idiopathic BAD	Control group	0.8916857360793288
1267	B84	B66	Idiopathic BAD	Control group	0.8077803203661327
1268	B84	B86	Idiopathic BAD	Control group	0.8752860411899314
1269	B84	B97	Idiopathic BAD	Control group	0.8419145690312738
1270	B84	B98	Idiopathic BAD	Control group	0.8678489702517163
1271	B84	B100	Idiopathic BAD	Control group	0.9294431731502669
1272	B84	B112	Idiopathic BAD	Control group	0.9042715484363082
1273	B84	B115	Idiopathic BAD	Control group	0.8802440884820748
1274	B84	I1	Idiopathic BAD	Control group	0.8779557589626239
1275	B84	I3	Idiopathic BAD	Control group	0.8806254767353165
1276	B84	I6	Idiopathic BAD	Control group	0.8348588863463006
1277	B84	I8	Idiopathic BAD	Control group	0.8945461479786423
1278	B84	I10	Idiopathic BAD	Control group	0.8476353928299009
1279	B84	I11	Idiopathic BAD	Control group	0.9126620900076278
1280	B84	I13	Idiopathic BAD	Control group	0.6777269260106789

1281 B84 I15 Idiopathic BAD Control group
0.6847826086956522
1282 B84 I17 Idiopathic BAD Control group
0.8882532418001525
1283 B84 I18 Idiopathic BAD Control group
0.795766590389016
1284 B84 I19 Idiopathic BAD Control group
0.9014111365369947
1285 B84 I22 Idiopathic BAD Control group
0.8318077803203662
1286 B84 I23 Idiopathic BAD Control group
0.8159801678108314
1287 B84 I24 Idiopathic BAD Control group
0.9004576659038902
1288 B84 I25 Idiopathic BAD Control group
0.8504958047292144
1289 B84 I26 Idiopathic BAD Control group
0.5776125095347063
1290 B84 I27 Idiopathic BAD Control group
0.8072082379862701
1291 B84 I28 Idiopathic BAD Control group
0.8758581235697941
1292 B84 I29 Idiopathic BAD Control group
0.8766209000762777
1293 B84 I30 Idiopathic BAD Control group
0.8106407322654462
1294 B84 I31 Idiopathic BAD Control group
0.9265827612509535
1295 B84 I32 Idiopathic BAD Control group
0.9000762776506483
1296 B84 I33 Idiopathic BAD Control group
0.8703279938977879
1297 B84 I34 Idiopathic BAD Control group
0.7972921434019832
1298 B84 I35 Idiopathic BAD Control group
0.8878718535469108
1299 B84 I36 Idiopathic BAD Control group
0.9157131960335622
1300 B84 I37 Idiopathic BAD Control group
0.8670861937452327
1301 B89 B27 Idiopathic BAD Control group
0.9088482074752098
1302 B89 B66 Idiopathic BAD Control group
0.812929061784897
1303 B89 B86 Idiopathic BAD Control group
0.809115179252479
1304 B89 B97 Idiopathic BAD Control group
0.8007246376811594
1305 B89 B98 Idiopathic BAD Control group
0.7503813882532419
1306 B89 B100 Idiopathic BAD Control group
0.9801678108314263
1307 B89 B112 Idiopathic BAD Control group
0.8859649122807017

1308	B89	B115	Idiopathic BAD	Control group	0.868421052631579
1309	B89	I1	Idiopathic BAD	Control group	0.8197940503432495
1310	B89	I3	Idiopathic BAD	Control group	0.7488558352402745
1311	B89	I6	Idiopathic BAD	Control group	0.8422959572845157
1312	B89	I8	Idiopathic BAD	Control group	0.8573607932875668
1313	B89	I10	Idiopathic BAD	Control group	0.7663996948893974
1314	B89	I11	Idiopathic BAD	Control group	0.8939740655987796
1315	B89	I13	Idiopathic BAD	Control group	0.8009153318077803
1316	B89	I15	Idiopathic BAD	Control group	0.8522120518688024
1317	B89	I17	Idiopathic BAD	Control group	0.8996948893974066
1318	B89	I18	Idiopathic BAD	Control group	0.7398932112890922
1319	B89	I19	Idiopathic BAD	Control group	0.9206712433257056
1320	B89	I22	Idiopathic BAD	Control group	0.7721205186880244
1321	B89	I23	Idiopathic BAD	Control group	0.7875667429443173
1322	B89	I24	Idiopathic BAD	Control group	0.8344774980930587
1323	B89	I25	Idiopathic BAD	Control group	0.7254004576659039
1324	B89	I26	Idiopathic BAD	Control group	0.7618230358504958
1325	B89	I27	Idiopathic BAD	Control group	0.7215865751334859
1326	B89	I28	Idiopathic BAD	Control group	0.793859649122807
1327	B89	I29	Idiopathic BAD	Control group	0.8226544622425629
1328	B89	I30	Idiopathic BAD	Control group	0.8081617086193745
1329	B89	I31	Idiopathic BAD	Control group	0.82627765064836
1330	B89	I32	Idiopathic BAD	Control group	0.7940503432494279
1331	B89	I33	Idiopathic BAD	Control group	0.7795575896262396
1332	B89	I34	Idiopathic BAD	Control group	0.8810068649885584
1333	B89	I35	Idiopathic BAD	Control group	0.6836384439359268
1334	B89	I36	Idiopathic BAD	Control group	0.988367658276125

1335 B89 I37 Idiopathic BAD Control group
0.8979786422578184
1336 B92 B27 Idiopathic BAD Control group
0.6228070175438597
1337 B92 B66 Idiopathic BAD Control group
0.7799389778794813
1338 B92 B86 Idiopathic BAD Control group
0.580091533180778
1339 B92 B97 Idiopathic BAD Control group
0.7376048817696415
1340 B92 B98 Idiopathic BAD Control group
0.7696414950419527
1341 B92 B100 Idiopathic BAD Control group
0.9195270785659801
1342 B92 B112 Idiopathic BAD Control group
0.6859267734553776
1343 B92 B115 Idiopathic BAD Control group
0.8165522501906941
1344 B92 I1 Idiopathic BAD Control group
0.8062547673531655
1345 B92 I3 Idiopathic BAD Control group
0.8367658276125095
1346 B92 I6 Idiopathic BAD Control group
0.7873760488176964
1347 B92 I8 Idiopathic BAD Control group
0.8199847444698704
1348 B92 I10 Idiopathic BAD Control group
0.7868039664378337
1349 B92 I11 Idiopathic BAD Control group
0.8647978642257819
1350 B92 I13 Idiopathic BAD Control group
0.7479023646071701
1351 B92 I15 Idiopathic BAD Control group
0.8007246376811594
1352 B92 I17 Idiopathic BAD Control group
0.6666666666666666
1353 B92 I18 Idiopathic BAD Control group
0.7126239511823036
1354 B92 I19 Idiopathic BAD Control group
0.7187261632341724
1355 B92 I22 Idiopathic BAD Control group
0.8133104500381388
1356 B92 I23 Idiopathic BAD Control group
0.7553394355453852
1357 B92 I24 Idiopathic BAD Control group
0.5194508009153318
1358 B92 I25 Idiopathic BAD Control group
0.7820366132723112
1359 B92 I26 Idiopathic BAD Control group
0.7765064836003052
1360 B92 I27 Idiopathic BAD Control group
0.7585812356979404
1361 B92 I28 Idiopathic BAD Control group
0.8539282990083905

1362 B92 I29 Idiopathic BAD Control group
0.856788710907704
1363 B92 I30 Idiopathic BAD Control group
0.8293287566742944
1364 B92 I31 Idiopathic BAD Control group
0.8728070175438597
1365 B92 I32 Idiopathic BAD Control group
0.8558352402745996
1366 B92 I33 Idiopathic BAD Control group
0.4929443173150267
1367 B92 I34 Idiopathic BAD Control group
0.6760106788710908
1368 B92 I35 Idiopathic BAD Control group
0.7675438596491229
1369 B92 I36 Idiopathic BAD Control group
0.956140350877193
1370 B92 I37 Idiopathic BAD Control group
0.8531655225019069
1371 B95 B27 Idiopathic BAD Control group
0.6407322654462243
1372 B95 B66 Idiopathic BAD Control group
0.8890160183066361
1373 B95 B86 Idiopathic BAD Control group
0.7524790236460717
1374 B95 B97 Idiopathic BAD Control group
0.8119755911517925
1375 B95 B98 Idiopathic BAD Control group
0.7459954233409611
1376 B95 B100 Idiopathic BAD Control group
0.9610983981693364
1377 B95 B112 Idiopathic BAD Control group
0.8070175438596491
1378 B95 B115 Idiopathic BAD Control group
0.8874904652936689
1379 B95 I1 Idiopathic BAD Control group
0.8165522501906941
1380 B95 I3 Idiopathic BAD Control group
0.7965293668954996
1381 B95 I6 Idiopathic BAD Control group
0.8175057208237986
1382 B95 I8 Idiopathic BAD Control group
0.8829138062547673
1383 B95 I10 Idiopathic BAD Control group
0.8400076277650649
1384 B95 I11 Idiopathic BAD Control group
0.860602593440122
1385 B95 I13 Idiopathic BAD Control group
0.851258581235698
1386 B95 I15 Idiopathic BAD Control group
0.992372234935164
1387 B95 I17 Idiopathic BAD Control group
0.7520976353928299
1388 B95 I18 Idiopathic BAD Control group
0.7799389778794813

1389	B95	I19	Idiopathic BAD	Control group	0.8289473684210527
1390	B95	I22	Idiopathic BAD	Control group	0.8762395118230358
1391	B95	I23	Idiopathic BAD	Control group	0.851067887109077
1392	B95	I24	Idiopathic BAD	Control group	0.6454996186117468
1393	B95	I25	Idiopathic BAD	Control group	0.7906178489702517
1394	B95	I26	Idiopathic BAD	Control group	0.8586956521739131
1395	B95	I27	Idiopathic BAD	Control group	0.8028222730739893
1396	B95	I28	Idiopathic BAD	Control group	0.8565980167810832
1397	B95	I29	Idiopathic BAD	Control group	0.8138825324180016
1398	B95	I30	Idiopathic BAD	Control group	0.8947368421052632
1399	B95	I31	Idiopathic BAD	Control group	0.8539282990083905
1400	B95	I32	Idiopathic BAD	Control group	0.8802440884820748
1401	B95	I33	Idiopathic BAD	Control group	0.6155606407322655
1402	B95	I34	Idiopathic BAD	Control group	0.7160564454614798
1403	B95	I35	Idiopathic BAD	Control group	0.7868039664378337
1404	B95	I36	Idiopathic BAD	Control group	1.0
1405	B95	I37	Idiopathic BAD	Control group	0.9420289855072463
1406	B99	B27	Idiopathic BAD	Control group	0.8247520976353928
1407	B99	B66	Idiopathic BAD	Control group	0.6893592677345538
1408	B99	B86	Idiopathic BAD	Control group	0.6990846681922197
1409	B99	B97	Idiopathic BAD	Control group	0.6235697940503433
1410	B99	B98	Idiopathic BAD	Control group	0.7139588100686499
1411	B99	B100	Idiopathic BAD	Control group	0.9624332570556827
1412	B99	B112	Idiopathic BAD	Control group	0.8266590389016019
1413	B99	B115	Idiopathic BAD	Control group	0.7745995423340961
1414	B99	I1	Idiopathic BAD	Control group	0.6548436308161708
1415	B99	I3	Idiopathic BAD	Control group	0.6401601830663616
1416	B99	I6	Idiopathic BAD	Control group	

0.6771548436308161
1417 B99 I8 Idiopathic BAD Control group
0.7437070938215103
1418 B99 I10 Idiopathic BAD Control group
0.7076659038901602
1419 B99 I11 Idiopathic BAD Control group
0.8438215102974829
1420 B99 I13 Idiopathic BAD Control group
0.6689549961861174
1421 B99 I15 Idiopathic BAD Control group
0.7202517162471396
1422 B99 I17 Idiopathic BAD Control group
0.7528604118993135
1423 B99 I18 Idiopathic BAD Control group
0.6435926773455377
1424 B99 I19 Idiopathic BAD Control group
0.8497330282227308
1425 B99 I22 Idiopathic BAD Control group
0.7316933638443935
1426 B99 I23 Idiopathic BAD Control group
0.7051868802440885
1427 B99 I24 Idiopathic BAD Control group
0.746186117467582
1428 B99 I25 Idiopathic BAD Control group
0.6454996186117468
1429 B99 I26 Idiopathic BAD Control group
0.6941266209000763
1430 B99 I27 Idiopathic BAD Control group
0.6510297482837528
1431 B99 I28 Idiopathic BAD Control group
0.8247520976353928
1432 B99 I29 Idiopathic BAD Control group
0.7095728451563692
1433 B99 I30 Idiopathic BAD Control group
0.7551487414187643
1434 B99 I31 Idiopathic BAD Control group
0.7852784134248665
1435 B99 I32 Idiopathic BAD Control group
0.7339816933638444
1436 B99 I33 Idiopathic BAD Control group
0.7126239511823036
1437 B99 I34 Idiopathic BAD Control group
0.8123569794050344
1438 B99 I35 Idiopathic BAD Control group
0.6950800915331807
1439 B99 I36 Idiopathic BAD Control group
0.9956140350877193
1440 B99 I37 Idiopathic BAD Control group
0.9130434782608695
1441 B103 B27 Idiopathic BAD Control group
0.9214340198321892
1442 B103 B66 Idiopathic BAD Control group
0.8569794050343249
1443 B103 B86 Idiopathic BAD Control group

0.9004576659038902
1444 B103 B97 Idiopathic BAD Control group
0.9124713958810069
1445 B103 B98 Idiopathic BAD Control group
0.868421052631579
1446 B103 B100 Idiopathic BAD Control group
0.9734935163996948
1447 B103 B112 Idiopathic BAD Control group
0.9361174675819984
1448 B103 B115 Idiopathic BAD Control group
0.9349733028222731
1449 B103 I1 Idiopathic BAD Control group
0.9286803966437833
1450 B103 I3 Idiopathic BAD Control group
0.8996948893974066
1451 B103 I6 Idiopathic BAD Control group
0.9424103737604882
1452 B103 I8 Idiopathic BAD Control group
0.9420289855072463
1453 B103 I10 Idiopathic BAD Control group
0.8884439359267735
1454 B103 I11 Idiopathic BAD Control group
0.9416475972540046
1455 B103 I13 Idiopathic BAD Control group
0.7835621662852784
1456 B103 I15 Idiopathic BAD Control group
0.843440122044241
1457 B103 I17 Idiopathic BAD Control group
0.9061784897025171
1458 B103 I18 Idiopathic BAD Control group
0.8884439359267735
1459 B103 I19 Idiopathic BAD Control group
0.9307780320366132
1460 B103 I22 Idiopathic BAD Control group
0.9385964912280702
1461 B103 I23 Idiopathic BAD Control group
0.8850114416475973
1462 B103 I24 Idiopathic BAD Control group
0.9496567505720824
1463 B103 I25 Idiopathic BAD Control group
0.8924485125858124
1464 B103 I26 Idiopathic BAD Control group
0.7864225781845919
1465 B103 I27 Idiopathic BAD Control group
0.9014111365369947
1466 B103 I28 Idiopathic BAD Control group
0.8993135011441648
1467 B103 I29 Idiopathic BAD Control group
0.9006483600305111
1468 B103 I30 Idiopathic BAD Control group
0.9193363844393593
1469 B103 I31 Idiopathic BAD Control group
0.9525171624713958
1470 B103 I32 Idiopathic BAD Control group

0.8720442410373761
1471 B103 I33 Idiopathic BAD Control group
0.9342105263157895
1472 B103 I34 Idiopathic BAD Control group
0.8647978642257819
1473 B103 I35 Idiopathic BAD Control group
0.8447749809305873
1474 B103 I36 Idiopathic BAD Control group
0.9887490465293669
1475 B103 I37 Idiopathic BAD Control group
0.9250572082379863
1476 B106 B27 Idiopathic BAD Control group
0.8609839816933639
1477 B106 B66 Idiopathic BAD Control group
0.9443173150266971
1478 B106 B86 Idiopathic BAD Control group
0.6784897025171625
1479 B106 B97 Idiopathic BAD Control group
0.9298245614035088
1480 B106 B98 Idiopathic BAD Control group
0.9315408085430968
1481 B106 B100 Idiopathic BAD Control group
0.9855072463768116
1482 B106 B112 Idiopathic BAD Control group
0.511441647597254
1483 B106 B115 Idiopathic BAD Control group
0.933066361556064
1484 B106 I1 Idiopathic BAD Control group
0.9321128909229596
1485 B106 I3 Idiopathic BAD Control group
0.9210526315789473
1486 B106 I6 Idiopathic BAD Control group
0.9246758199847445
1487 B106 I8 Idiopathic BAD Control group
0.9315408085430968
1488 B106 I10 Idiopathic BAD Control group
0.9477498093058734
1489 B106 I11 Idiopathic BAD Control group
0.969488939740656
1490 B106 I13 Idiopathic BAD Control group
0.9363081617086194
1491 B106 I15 Idiopathic BAD Control group
0.9813119755911518
1492 B106 I17 Idiopathic BAD Control group
0.7364607170099161
1493 B106 I18 Idiopathic BAD Control group
0.9357360793287567
1494 B106 I19 Idiopathic BAD Control group
0.6826849733028223
1495 B106 I22 Idiopathic BAD Control group
0.9549961861174676
1496 B106 I23 Idiopathic BAD Control group
0.9250572082379863
1497 B106 I24 Idiopathic BAD Control group

0.6588482074752098
1498 B106 I25 Idiopathic BAD Control group
0.9262013729977117
1499 B106 I26 Idiopathic BAD Control group
0.9487032799389779
1500 B106 I27 Idiopathic BAD Control group
0.9467963386727689
1501 B106 I28 Idiopathic BAD Control group
0.9626239511823036
1502 B106 I29 Idiopathic BAD Control group
0.9506102212051869
1503 B106 I30 Idiopathic BAD Control group
0.971205186880244
1504 B106 I31 Idiopathic BAD Control group
0.9603356216628528
1505 B106 I32 Idiopathic BAD Control group
0.9553775743707094
1506 B106 I33 Idiopathic BAD Control group
0.6742944317315027
1507 B106 I34 Idiopathic BAD Control group
0.876048817696415
1508 B106 I35 Idiopathic BAD Control group
0.9477498093058734
1509 B106 I36 Idiopathic BAD Control group
0.9996186117467581
1510 B106 I37 Idiopathic BAD Control group
0.9691075514874142
1511 B109 B27 Idiopathic BAD Control group
0.8806254767353165
1512 B109 B66 Idiopathic BAD Control group
0.7479023646071701
1513 B109 B86 Idiopathic BAD Control group
0.8203661327231121
1514 B109 B97 Idiopathic BAD Control group
0.698512585812357
1515 B109 B98 Idiopathic BAD Control group
0.8619374523264683
1516 B109 B100 Idiopathic BAD Control group
0.9822654462242563
1517 B109 B112 Idiopathic BAD Control group
0.8869183829138062
1518 B109 B115 Idiopathic BAD Control group
0.784324942791762
1519 B109 I1 Idiopathic BAD Control group
0.8237986270022883
1520 B109 I3 Idiopathic BAD Control group
0.7627765064836003
1521 B109 I6 Idiopathic BAD Control group
0.7723112128146453
1522 B109 I8 Idiopathic BAD Control group
0.8766209000762777
1523 B109 I10 Idiopathic BAD Control group
0.7951945080091534
1524 B109 I11 Idiopathic BAD Control group

0.9124713958810069
1525	B109	I13	Idiopathic BAD	Control group
0.8455377574370709
1526	B109	I15	Idiopathic BAD	Control group
0.7986270022883295
1527	B109	I17	Idiopathic BAD	Control group
0.8859649122807017
1528	B109	I18	Idiopathic BAD	Control group
0.7967200610221206
1529	B109	I19	Idiopathic BAD	Control group
0.9031273836765827
1530	B109	I22	Idiopathic BAD	Control group
0.8556445461479787
1531	B109	I23	Idiopathic BAD	Control group
0.7883295194508009
1532	B109	I24	Idiopathic BAD	Control group
0.8470633104500381
1533	B109	I25	Idiopathic BAD	Control group
0.7892829900839055
1534	B109	I26	Idiopathic BAD	Control group
0.7381769641495042
1535	B109	I27	Idiopathic BAD	Control group
0.7419908466819222
1536	B109	I28	Idiopathic BAD	Control group
0.8790999237223494
1537	B109	I29	Idiopathic BAD	Control group
0.7873760488176964
1538	B109	I30	Idiopathic BAD	Control group
0.742372234935164
1539	B109	I31	Idiopathic BAD	Control group
0.8165522501906941
1540	B109	I32	Idiopathic BAD	Control group
0.8733790999237223
1541	B109	I33	Idiopathic BAD	Control group
0.8649885583524027
1542	B109	I34	Idiopathic BAD	Control group
0.8356216628527842
1543	B109	I35	Idiopathic BAD	Control group
0.9042715484363082
1544	B109	I36	Idiopathic BAD	Control group
0.9998093058733791
1545	B109	I37	Idiopathic BAD	Control group
0.9559496567505721
1546	B118	B27	Idiopathic BAD	Control group
0.7988176964149504
1547	B118	B66	Idiopathic BAD	Control group
0.8522120518688024
1548	B118	B86	Idiopathic BAD	Control group
0.5678871090770404
1549	B118	B97	Idiopathic BAD	Control group
0.8787185354691075
1550	B118	B98	Idiopathic BAD	Control group
0.8443935926773455
1551	B118	B100	Idiopathic BAD	Control group

0.9530892448512586
1552	B118	B112	Idiopathic BAD	Control group
0.6413043478260869
1553	B118	B115	Idiopathic BAD	Control group
0.9084668192219679
1554	B118	I1	Idiopathic BAD	Control group
0.8102593440122045
1555	B118	I3	Idiopathic BAD	Control group
0.8382913806254767
1556	B118	I6	Idiopathic BAD	Control group
0.8564073226544623
1557	B118	I8	Idiopathic BAD	Control group
0.8787185354691075
1558	B118	I10	Idiopathic BAD	Control group
0.8712814645308925
1559	B118	I11	Idiopathic BAD	Control group
0.9399313501144165
1560	B118	I13	Idiopathic BAD	Control group
0.8724256292906178
1561	B118	I15	Idiopathic BAD	Control group
0.9107551487414187
1562	B118	I17	Idiopathic BAD	Control group
0.6617086193745233
1563	B118	I18	Idiopathic BAD	Control group
0.7797482837528604
1564	B118	I19	Idiopathic BAD	Control group
0.7133867276887872
1565	B118	I22	Idiopathic BAD	Control group
0.8903508771929824
1566	B118	I23	Idiopathic BAD	Control group
0.8245614035087719
1567	B118	I24	Idiopathic BAD	Control group
0.5444317315026698
1568	B118	I25	Idiopathic BAD	Control group
0.8293287566742944
1569	B118	I26	Idiopathic BAD	Control group
0.8756674294431731
1570	B118	I27	Idiopathic BAD	Control group
0.7883295194508009
1571	B118	I28	Idiopathic BAD	Control group
0.8916857360793288
1572	B118	I29	Idiopathic BAD	Control group
0.8678489702517163
1573	B118	I30	Idiopathic BAD	Control group
0.8752860411899314
1574	B118	I31	Idiopathic BAD	Control group
0.8884439359267735
1575	B118	I32	Idiopathic BAD	Control group
0.8945461479786423
1576	B118	I33	Idiopathic BAD	Control group
0.5327993897787948
1577	B118	I34	Idiopathic BAD	Control group
0.7391304347826086
1578	B118	I35	Idiopathic BAD	Control group

0.8466819221967964
1579 B118 I36 Idiopathic BAD Control group
0.9994279176201373
1580 B118 I37 Idiopathic BAD Control group
0.9445080091533181
1581 B119 B27 Idiopathic BAD Control group
0.8497330282227308
1582 B119 B66 Idiopathic BAD Control group
0.6960335621662853
1583 B119 B86 Idiopathic BAD Control group
0.7915713196033562
1584 B119 B97 Idiopathic BAD Control group
0.7446605644546148
1585 B119 B98 Idiopathic BAD Control group
0.8146453089244852
1586 B119 B100 Idiopathic BAD Control group
0.9336384439359268
1587 B119 B112 Idiopathic BAD Control group
0.8625095347063311
1588 B119 B115 Idiopathic BAD Control group
0.723302822273074
1589 B119 I1 Idiopathic BAD Control group
0.7658276125095347
1590 B119 I3 Idiopathic BAD Control group
0.7318840579710145
1591 B119 I6 Idiopathic BAD Control group
0.6374904652936689
1592 B119 I8 Idiopathic BAD Control group
0.7984363081617086
1593 B119 I10 Idiopathic BAD Control group
0.7694508009153318
1594 B119 I11 Idiopathic BAD Control group
0.9145690312738368
1595 B119 I13 Idiopathic BAD Control group
0.7601067887109078
1596 B119 I15 Idiopathic BAD Control group
0.6708619374523265
1597 B119 I17 Idiopathic BAD Control group
0.8676582761250954
1598 B119 I18 Idiopathic BAD Control group
0.7368421052631579
1599 B119 I19 Idiopathic BAD Control group
0.915903890160183
1600 B119 I22 Idiopathic BAD Control group
0.8297101449275363
1601 B119 I23 Idiopathic BAD Control group
0.7583905415713196
1602 B119 I24 Idiopathic BAD Control group
0.8518306636155606
1603 B119 I25 Idiopathic BAD Control group
0.7435163996948894
1604 B119 I26 Idiopathic BAD Control group
0.6863081617086194
1605 B119 I27 Idiopathic BAD Control group

0.6945080091533181
1606 B119 I28 Idiopathic BAD Control group
0.8211289092295957
1607 B119 I29 Idiopathic BAD Control group
0.7971014492753623
1608 B119 I30 Idiopathic BAD Control group
0.5696033562166285
1609 B119 I31 Idiopathic BAD Control group
0.8459191456903128
1610 B119 I32 Idiopathic BAD Control group
0.7978642257818459
1611 B119 I33 Idiopathic BAD Control group
0.8333333333333334
1612 B119 I34 Idiopathic BAD Control group
0.7988176964149504
1613 B119 I35 Idiopathic BAD Control group
0.7984363081617086
1614 B119 I36 Idiopathic BAD Control group
0.992372234935164
1615 B119 I37 Idiopathic BAD Control group
0.8745232646834478
1616 B1 P1 Idiopathic BAD Post-cholecystectomy
0.8483981693363845
1617 B1 P2 Idiopathic BAD Post-cholecystectomy
0.8945461479786423
1618 B1 P4 Idiopathic BAD Post-cholecystectomy
0.7416094584286804
1619 B1 P5 Idiopathic BAD Post-cholecystectomy
0.8354309687261632
1620 B1 P9 Idiopathic BAD Post-cholecystectomy
0.765255530129672
1621 B1 P13 Idiopathic BAD Post-cholecystectomy
0.9042715484363082
1622 B1 P15 Idiopathic BAD Post-cholecystectomy
0.8030129672006102
1623 B1 P16 Idiopathic BAD Post-cholecystectomy
0.8030129672006102
1624 B1 P17 Idiopathic BAD Post-cholecystectomy
0.9092295957284515
1625 B1 P20 Idiopathic BAD Post-cholecystectomy
0.7467581998474447
1626 B1 P21 Idiopathic BAD Post-cholecystectomy
0.8909229595728452
1627 B1 P24 Idiopathic BAD Post-cholecystectomy
0.927536231884058
1628 B1 P26 Idiopathic BAD Post-cholecystectomy
0.872234935163997
1629 B1 P30 Idiopathic BAD Post-cholecystectomy
0.8621281464530892
1630 B1 P33 Idiopathic BAD Post-cholecystectomy
0.8861556064073226
1631 B1 P35 Idiopathic BAD Post-cholecystectomy
0.9500381388253242
1632 B1 P38 Idiopathic BAD Post-cholecystectomy

0.9391685736079328
1633	B1	P39	Idiopathic BAD	Post-cholecystectomy
0.8165522501906941
1634	B1	P42	Idiopathic BAD	Post-cholecystectomy
0.9229595728451564
1635	B1	P43	Idiopathic BAD	Post-cholecystectomy
0.8007246376811594
1636	B1	P46	Idiopathic BAD	Post-cholecystectomy
0.7969107551487414
1637	B1	P47	Idiopathic BAD	Post-cholecystectomy
0.9511823035850496
1638	B1	P50	Idiopathic BAD	Post-cholecystectomy
0.8560259344012204
1639	B1	P55	Idiopathic BAD	Post-cholecystectomy
0.8125476735316552
1640	B1	P58	Idiopathic BAD	Post-cholecystectomy
0.8745232646834478
1641	B1	P60	Idiopathic BAD	Post-cholecystectomy
0.7841342486651411
1642	B1	P63	Idiopathic BAD	Post-cholecystectomy
0.8993135011441648
1643	B1	P65	Idiopathic BAD	Post-cholecystectomy
0.9235316552250191
1644	B1	P68	Idiopathic BAD	Post-cholecystectomy
0.9469870327993898
1645	B1	P70	Idiopathic BAD	Post-cholecystectomy
0.9136155606407322
1646	B1	P71	Idiopathic BAD	Post-cholecystectomy
0.9435545385202135
1647	B1	P74	Idiopathic BAD	Post-cholecystectomy
0.8905415713196033
1648	B1	P75	Idiopathic BAD	Post-cholecystectomy
0.8554538520213577
1649	B5	P1	Idiopathic BAD	Post-cholecystectomy
0.9107551487414187
1650	B5	P2	Idiopathic BAD	Post-cholecystectomy
0.8159801678108314
1651	B5	P4	Idiopathic BAD	Post-cholecystectomy
0.7644927536231884
1652	B5	P5	Idiopathic BAD	Post-cholecystectomy
0.7587719298245614
1653	B5	P9	Idiopathic BAD	Post-cholecystectomy
0.8316170861937452
1654	B5	P13	Idiopathic BAD	Post-cholecystectomy
0.8546910755148741
1655	B5	P15	Idiopathic BAD	Post-cholecystectomy
0.7799389778794813
1656	B5	P16	Idiopathic BAD	Post-cholecystectomy
0.8161708619374524
1657	B5	P17	Idiopathic BAD	Post-cholecystectomy
0.8585049580472921
1658	B5	P20	Idiopathic BAD	Post-cholecystectomy
0.6864988558352403
1659	B5	P21	Idiopathic BAD	Post-cholecystectomy

0.6727688787185355
1660 B5 P24 Idiopathic BAD Post-cholecystectomy
0.8995041952707856
1661 B5 P26 Idiopathic BAD Post-cholecystectomy
0.7663996948893974
1662 B5 P30 Idiopathic BAD Post-cholecystectomy
0.8943554538520213
1663 B5 P33 Idiopathic BAD Post-cholecystectomy
0.7730739893211289
1664 B5 P35 Idiopathic BAD Post-cholecystectomy
0.8623188405797102
1665 B5 P38 Idiopathic BAD Post-cholecystectomy
0.9397406559877955
1666 B5 P39 Idiopathic BAD Post-cholecystectomy
0.7446605644546148
1667 B5 P42 Idiopathic BAD Post-cholecystectomy
0.8956903127383676
1668 B5 P43 Idiopathic BAD Post-cholecystectomy
0.8150266971777269
1669 B5 P46 Idiopathic BAD Post-cholecystectomy
0.7377955758962624
1670 B5 P47 Idiopathic BAD Post-cholecystectomy
0.938977879481312
1671 B5 P50 Idiopathic BAD Post-cholecystectomy
0.7879481311975591
1672 B5 P55 Idiopathic BAD Post-cholecystectomy
0.8350495804729214
1673 B5 P58 Idiopathic BAD Post-cholecystectomy
0.7742181540808543
1674 B5 P60 Idiopathic BAD Post-cholecystectomy
0.8114035087719298
1675 B5 P63 Idiopathic BAD Post-cholecystectomy
0.8459191456903128
1676 B5 P65 Idiopathic BAD Post-cholecystectomy
0.8278032036613272
1677 B5 P68 Idiopathic BAD Post-cholecystectomy
0.8087337909992373
1678 B5 P70 Idiopathic BAD Post-cholecystectomy
0.7868039664378337
1679 B5 P71 Idiopathic BAD Post-cholecystectomy
0.8196033562166285
1680 B5 P74 Idiopathic BAD Post-cholecystectomy
0.7564836003051106
1681 B5 P75 Idiopathic BAD Post-cholecystectomy
0.738558352402746
1682 B6 P1 Idiopathic BAD Post-cholecystectomy
0.9401220442410374
1683 B6 P2 Idiopathic BAD Post-cholecystectomy
0.8825324180015256
1684 B6 P4 Idiopathic BAD Post-cholecystectomy
0.855072463768116
1685 B6 P5 Idiopathic BAD Post-cholecystectomy
0.8325705568268498
1686 B6 P9 Idiopathic BAD Post-cholecystectomy

0.9084668192219679
1687 B6 P13 Idiopathic BAD Post−cholecystectomy
0.7305491990846682
1688 B6 P15 Idiopathic BAD Post−cholecystectomy
0.8686117467581999
1689 B6 P16 Idiopathic BAD Post−cholecystectomy
0.933066361556064
1690 B6 P17 Idiopathic BAD Post−cholecystectomy
0.9012204424103738
1691 B6 P20 Idiopathic BAD Post−cholecystectomy
0.7745995423340961
1692 B6 P21 Idiopathic BAD Post−cholecystectomy
0.8968344774980931
1693 B6 P24 Idiopathic BAD Post−cholecystectomy
0.7692601067887109
1694 B6 P26 Idiopathic BAD Post−cholecystectomy
0.8337147215865751
1695 B6 P30 Idiopathic BAD Post−cholecystectomy
0.9403127383676583
1696 B6 P33 Idiopathic BAD Post−cholecystectomy
0.8178871090770404
1697 B6 P35 Idiopathic BAD Post−cholecystectomy
0.8861556064073226
1698 B6 P38 Idiopathic BAD Post−cholecystectomy
0.9570938215102975
1699 B6 P39 Idiopathic BAD Post−cholecystectomy
0.8903508771929824
1700 B6 P42 Idiopathic BAD Post−cholecystectomy
0.9614797864225781
1701 B6 P43 Idiopathic BAD Post−cholecystectomy
0.7618230358504958
1702 B6 P46 Idiopathic BAD Post−cholecystectomy
0.8663234172387491
1703 B6 P47 Idiopathic BAD Post−cholecystectomy
0.9317315026697178
1704 B6 P50 Idiopathic BAD Post−cholecystectomy
0.8647978642257819
1705 B6 P55 Idiopathic BAD Post−cholecystectomy
0.7890922959572845
1706 B6 P58 Idiopathic BAD Post−cholecystectomy
0.9099923722349351
1707 B6 P60 Idiopathic BAD Post−cholecystectomy
0.9372616323417239
1708 B6 P63 Idiopathic BAD Post−cholecystectomy
0.8489702517162472
1709 B6 P65 Idiopathic BAD Post−cholecystectomy
0.8556445461479787
1710 B6 P68 Idiopathic BAD Post−cholecystectomy
0.8636536994660564
1711 B6 P70 Idiopathic BAD Post−cholecystectomy
0.8796720061022121
1712 B6 P71 Idiopathic BAD Post−cholecystectomy
0.9080854309687262
1713 B6 P74 Idiopathic BAD Post−cholecystectomy

0.8117848970251716
1714 B6 P75 Idiopathic BAD Post-cholecystectomy
0.8251334858886347
1715 B10 P1 Idiopathic BAD Post-cholecystectomy
0.8432494279176201
1716 B10 P2 Idiopathic BAD Post-cholecystectomy
0.8377192982456141
1717 B10 P4 Idiopathic BAD Post-cholecystectomy
0.8119755911517925
1718 B10 P5 Idiopathic BAD Post-cholecystectomy
0.7942410373760488
1719 B10 P9 Idiopathic BAD Post-cholecystectomy
0.8274218154080855
1720 B10 P13 Idiopathic BAD Post-cholecystectomy
0.9256292906178489
1721 B10 P15 Idiopathic BAD Post-cholecystectomy
0.7875667429443173
1722 B10 P16 Idiopathic BAD Post-cholecystectomy
0.8827231121281465
1723 B10 P17 Idiopathic BAD Post-cholecystectomy
0.9317315026697178
1724 B10 P20 Idiopathic BAD Post-cholecystectomy
0.8333333333333334
1725 B10 P21 Idiopathic BAD Post-cholecystectomy
0.9487032799389779
1726 B10 P24 Idiopathic BAD Post-cholecystectomy
0.8390541571319603
1727 B10 P26 Idiopathic BAD Post-cholecystectomy
0.9815026697177727
1728 B10 P30 Idiopathic BAD Post-cholecystectomy
0.7656369183829138
1729 B10 P33 Idiopathic BAD Post-cholecystectomy
0.8914950419527079
1730 B10 P35 Idiopathic BAD Post-cholecystectomy
0.9420289855072463
1731 B10 P38 Idiopathic BAD Post-cholecystectomy
0.8680396643783371
1732 B10 P39 Idiopathic BAD Post-cholecystectomy
0.9057971014492754
1733 B10 P42 Idiopathic BAD Post-cholecystectomy
0.9708237986270023
1734 B10 P43 Idiopathic BAD Post-cholecystectomy
0.8733790999237223
1735 B10 P46 Idiopathic BAD Post-cholecystectomy
0.9229595728451564
1736 B10 P47 Idiopathic BAD Post-cholecystectomy
0.9229595728451564
1737 B10 P50 Idiopathic BAD Post-cholecystectomy
0.8459191456903128
1738 B10 P55 Idiopathic BAD Post-cholecystectomy
0.8012967200610221
1739 B10 P58 Idiopathic BAD Post-cholecystectomy
0.9138062547673532
1740 B10 P60 Idiopathic BAD Post-cholecystectomy

0.8918764302059496
1741 B10 P63 Idiopathic BAD Post–cholecystectomy
0.8501144164759725
1742 B10 P65 Idiopathic BAD Post–cholecystectomy
0.9109458428680397
1743 B10 P68 Idiopathic BAD Post–cholecystectomy
0.8983600305110603
1744 B10 P70 Idiopathic BAD Post–cholecystectomy
0.9610983981693364
1745 B10 P71 Idiopathic BAD Post–cholecystectomy
0.9612890922959573
1746 B10 P74 Idiopathic BAD Post–cholecystectomy
0.8890160183066361
1747 B10 P75 Idiopathic BAD Post–cholecystectomy
0.831998474446987
1748 B17 P1 Idiopathic BAD Post–cholecystectomy
0.9631960335621663
1749 B17 P2 Idiopathic BAD Post–cholecystectomy
0.7953852021357742
1750 B17 P4 Idiopathic BAD Post–cholecystectomy
0.7377955758962624
1751 B17 P5 Idiopathic BAD Post–cholecystectomy
0.7459954233409611
1752 B17 P9 Idiopathic BAD Post–cholecystectomy
0.7980549199084668
1753 B17 P13 Idiopathic BAD Post–cholecystectomy
0.8726163234172387
1754 B17 P15 Idiopathic BAD Post–cholecystectomy
0.5575896262395118
1755 B17 P16 Idiopathic BAD Post–cholecystectomy
0.8361937452326468
1756 B17 P17 Idiopathic BAD Post–cholecystectomy
0.8318077803203662
1757 B17 P20 Idiopathic BAD Post–cholecystectomy
0.8516399694889397
1758 B17 P21 Idiopathic BAD Post–cholecystectomy
0.8457284515636918
1759 B17 P24 Idiopathic BAD Post–cholecystectomy
0.8135011441647597
1760 B17 P26 Idiopathic BAD Post–cholecystectomy
0.8794813119755912
1761 B17 P30 Idiopathic BAD Post–cholecystectomy
0.8575514874141876
1762 B17 P33 Idiopathic BAD Post–cholecystectomy
0.8276125095347063
1763 B17 P35 Idiopathic BAD Post–cholecystectomy
0.8569794050343249
1764 B17 P38 Idiopathic BAD Post–cholecystectomy
0.9281083142639207
1765 B17 P39 Idiopathic BAD Post–cholecystectomy
0.8880625476735317
1766 B17 P42 Idiopathic BAD Post–cholecystectomy
0.9183829138062548
1767 B17 P43 Idiopathic BAD Post–cholecystectomy

0.8447749809305873
1768	B17	P46	Idiopathic BAD	Post-cholecystectomy
0.7343630816170862
1769	B17	P47	Idiopathic BAD	Post-cholecystectomy
0.6903127383676583
1770	B17	P50	Idiopathic BAD	Post-cholecystectomy
0.7467581998474447
1771	B17	P55	Idiopathic BAD	Post-cholecystectomy
0.7364607170099161
1772	B17	P58	Idiopathic BAD	Post-cholecystectomy
0.8558352402745996
1773	B17	P60	Idiopathic BAD	Post-cholecystectomy
0.8865369946605645
1774	B17	P63	Idiopathic BAD	Post-cholecystectomy
0.8274218154080855
1775	B17	P65	Idiopathic BAD	Post-cholecystectomy
0.8247520976353928
1776	B17	P68	Idiopathic BAD	Post-cholecystectomy
0.8453470633104501
1777	B17	P70	Idiopathic BAD	Post-cholecystectomy
0.8743325705568269
1778	B17	P71	Idiopathic BAD	Post-cholecystectomy
0.6512204424103738
1779	B17	P74	Idiopathic BAD	Post-cholecystectomy
0.8176964149504196
1780	B17	P75	Idiopathic BAD	Post-cholecystectomy
0.7467581998474447
1781	B20	P1	Idiopathic BAD	Post-cholecystectomy
0.9631960335621663
1782	B20	P2	Idiopathic BAD	Post-cholecystectomy
0.7934782608695652
1783	B20	P4	Idiopathic BAD	Post-cholecystectomy
0.8497330282227308
1784	B20	P5	Idiopathic BAD	Post-cholecystectomy
0.7923340961098398
1785	B20	P9	Idiopathic BAD	Post-cholecystectomy
0.9023646071700991
1786	B20	P13	Idiopathic BAD	Post-cholecystectomy
0.8163615560640732
1787	B20	P15	Idiopathic BAD	Post-cholecystectomy
0.6735316552250191
1788	B20	P16	Idiopathic BAD	Post-cholecystectomy
0.8905415713196033
1789	B20	P17	Idiopathic BAD	Post-cholecystectomy
0.8094965675057209
1790	B20	P20	Idiopathic BAD	Post-cholecystectomy
0.8413424866514111
1791	B20	P21	Idiopathic BAD	Post-cholecystectomy
0.8194126620900076
1792	B20	P24	Idiopathic BAD	Post-cholecystectomy
0.8712814645308925
1793	B20	P26	Idiopathic BAD	Post-cholecystectomy
0.8821510297482837
1794	B20	P30	Idiopathic BAD	Post-cholecystectomy

0.9471777269260107
1795	B20	P33	Idiopathic BAD	Post-cholecystectomy
0.8278032036613272
1796	B20	P35	Idiopathic BAD	Post-cholecystectomy
0.8255148741418764
1797	B20	P38	Idiopathic BAD	Post-cholecystectomy
0.9715865751334859
1798	B20	P39	Idiopathic BAD	Post-cholecystectomy
0.9290617848970252
1799	B20	P42	Idiopathic BAD	Post-cholecystectomy
0.9439359267734554
1800	B20	P43	Idiopathic BAD	Post-cholecystectomy
0.8495423340961098
1801	B20	P46	Idiopathic BAD	Post-cholecystectomy
0.6426392067124332
1802	B20	P47	Idiopathic BAD	Post-cholecystectomy
0.6111746758199847
1803	B20	P50	Idiopathic BAD	Post-cholecystectomy
0.7953852021357742
1804	B20	P55	Idiopathic BAD	Post-cholecystectomy
0.8098779557589626
1805	B20	P58	Idiopathic BAD	Post-cholecystectomy
0.8453470633104501
1806	B20	P60	Idiopathic BAD	Post-cholecystectomy
0.9530892448512586
1807	B20	P63	Idiopathic BAD	Post-cholecystectomy
0.8361937452326468
1808	B20	P65	Idiopathic BAD	Post-cholecystectomy
0.8560259344012204
1809	B20	P68	Idiopathic BAD	Post-cholecystectomy
0.8596491228070176
1810	B20	P70	Idiopathic BAD	Post-cholecystectomy
0.8281845919145691
1811	B20	P71	Idiopathic BAD	Post-cholecystectomy
0.6577040427154843
1812	B20	P74	Idiopathic BAD	Post-cholecystectomy
0.8503051106025934
1813	B20	P75	Idiopathic BAD	Post-cholecystectomy
0.7906178489702517
1814	B23	P1	Idiopathic BAD	Post-cholecystectomy
0.9490846681922197
1815	B23	P2	Idiopathic BAD	Post-cholecystectomy
0.7490465293668955
1816	B23	P4	Idiopathic BAD	Post-cholecystectomy
0.7917620137299771
1817	B23	P5	Idiopathic BAD	Post-cholecystectomy
0.698512585812357
1818	B23	P9	Idiopathic BAD	Post-cholecystectomy
0.7044241037376049
1819	B23	P13	Idiopathic BAD	Post-cholecystectomy
0.8716628527841342
1820	B23	P15	Idiopathic BAD	Post-cholecystectomy
0.7509534706331045
1821	B23	P16	Idiopathic BAD	Post-cholecystectomy

0.7734553775743707
1822 B23 P17 Idiopathic BAD Post-cholecystectomy
0.8382913806254767
1823 B23 P20 Idiopathic BAD Post-cholecystectomy
0.8136918382913806
1824 B23 P21 Idiopathic BAD Post-cholecystectomy
0.7332189168573608
1825 B23 P24 Idiopathic BAD Post-cholecystectomy
0.8636536994660564
1826 B23 P26 Idiopathic BAD Post-cholecystectomy
0.681350114416476
1827 B23 P30 Idiopathic BAD Post-cholecystectomy
0.9166666666666666
1828 B23 P33 Idiopathic BAD Post-cholecystectomy
0.7774599542334096
1829 B23 P35 Idiopathic BAD Post-cholecystectomy
0.6424485125858124
1830 B23 P38 Idiopathic BAD Post-cholecystectomy
0.8525934401220442
1831 B23 P39 Idiopathic BAD Post-cholecystectomy
0.8480167810831426
1832 B23 P42 Idiopathic BAD Post-cholecystectomy
0.7299771167048055
1833 B23 P43 Idiopathic BAD Post-cholecystectomy
0.8709000762776506
1834 B23 P46 Idiopathic BAD Post-cholecystectomy
0.7934782608695652
1835 B23 P47 Idiopathic BAD Post-cholecystectomy
0.912090007627765
1836 B23 P50 Idiopathic BAD Post-cholecystectomy
0.6844012204424104
1837 B23 P55 Idiopathic BAD Post-cholecystectomy
0.5938215102974829
1838 B23 P58 Idiopathic BAD Post-cholecystectomy
0.6897406559877955
1839 B23 P60 Idiopathic BAD Post-cholecystectomy
0.916094584286804
1840 B23 P63 Idiopathic BAD Post-cholecystectomy
0.8081617086193745
1841 B23 P65 Idiopathic BAD Post-cholecystectomy
0.7524790236460717
1842 B23 P68 Idiopathic BAD Post-cholecystectomy
0.6966056445461479
1843 B23 P70 Idiopathic BAD Post-cholecystectomy
0.7151029748283753
1844 B23 P71 Idiopathic BAD Post-cholecystectomy
0.8272311212814645
1845 B23 P74 Idiopathic BAD Post-cholecystectomy
0.7658276125095347
1846 B23 P75 Idiopathic BAD Post-cholecystectomy
0.7479023646071701
1847 B31 P1 Idiopathic BAD Post-cholecystectomy
0.8577421815408085
1848 B31 P2 Idiopathic BAD Post-cholecystectomy

0.8415331807780321
1849	B31	P4	Idiopathic BAD	Post-cholecystectomy
0.7555301296720061
1850	B31	P5	Idiopathic BAD	Post-cholecystectomy
0.7852784134248665
1851	B31	P9	Idiopathic BAD	Post-cholecystectomy
0.6651411136536994
1852	B31	P13	Idiopathic BAD	Post-cholecystectomy
0.8771929824561403
1853	B31	P15	Idiopathic BAD	Post-cholecystectomy
0.7437070938215103
1854	B31	P16	Idiopathic BAD	Post-cholecystectomy
0.834096109839817
1855	B31	P17	Idiopathic BAD	Post-cholecystectomy
0.9139969488939741
1856	B31	P20	Idiopathic BAD	Post-cholecystectomy
0.7265446224256293
1857	B31	P21	Idiopathic BAD	Post-cholecystectomy
0.8281845919145691
1858	B31	P24	Idiopathic BAD	Post-cholecystectomy
0.8487795575896262
1859	B31	P26	Idiopathic BAD	Post-cholecystectomy
0.8667048054919908
1860	B31	P30	Idiopathic BAD	Post-cholecystectomy
0.7942410373760488
1861	B31	P33	Idiopathic BAD	Post-cholecystectomy
0.8579328756674295
1862	B31	P35	Idiopathic BAD	Post-cholecystectomy
0.8440122044241037
1863	B31	P38	Idiopathic BAD	Post-cholecystectomy
0.9033180778032036
1864	B31	P39	Idiopathic BAD	Post-cholecystectomy
0.818649885583524
1865	B31	P42	Idiopathic BAD	Post-cholecystectomy
0.9508009153318078
1866	B31	P43	Idiopathic BAD	Post-cholecystectomy
0.8009153318077803
1867	B31	P46	Idiopathic BAD	Post-cholecystectomy
0.8808161708619374
1868	B31	P47	Idiopathic BAD	Post-cholecystectomy
0.881769641495042
1869	B31	P50	Idiopathic BAD	Post-cholecystectomy
0.7795575896262396
1870	B31	P55	Idiopathic BAD	Post-cholecystectomy
0.757627765064836
1871	B31	P58	Idiopathic BAD	Post-cholecystectomy
0.8775743707093822
1872	B31	P60	Idiopathic BAD	Post-cholecystectomy
0.8729977116704806
1873	B31	P63	Idiopathic BAD	Post-cholecystectomy
0.8257055682684973
1874	B31	P65	Idiopathic BAD	Post-cholecystectomy
0.933066361556064
1875	B31	P68	Idiopathic BAD	Post-cholecystectomy

0.8781464530892449
1876 B31 P70 Idiopathic BAD Post-cholecystectomy
0.8710907704042715
1877 B31 P71 Idiopathic BAD Post-cholecystectomy
0.8745232646834478
1878 B31 P74 Idiopathic BAD Post-cholecystectomy
0.8350495804729214
1879 B31 P75 Idiopathic BAD Post-cholecystectomy
0.7873760488176964
1880 B35 P1 Idiopathic BAD Post-cholecystectomy
0.9824561403508771
1881 B35 P2 Idiopathic BAD Post-cholecystectomy
0.9845537757437071
1882 B35 P4 Idiopathic BAD Post-cholecystectomy
0.8485888634630053
1883 B35 P5 Idiopathic BAD Post-cholecystectomy
0.9456521739130435
1884 B35 P9 Idiopathic BAD Post-cholecystectomy
0.9664378337147216
1885 B35 P13 Idiopathic BAD Post-cholecystectomy
0.9668192219679634
1886 B35 P15 Idiopathic BAD Post-cholecystectomy
0.9324942791762014
1887 B35 P16 Idiopathic BAD Post-cholecystectomy
0.8205568268497331
1888 B35 P17 Idiopathic BAD Post-cholecystectomy
0.994279176201373
1889 B35 P20 Idiopathic BAD Post-cholecystectomy
0.6279557589626239
1890 B35 P21 Idiopathic BAD Post-cholecystectomy
0.8848207475209764
1891 B35 P24 Idiopathic BAD Post-cholecystectomy
0.9729214340198322
1892 B35 P26 Idiopathic BAD Post-cholecystectomy
0.8226544622425629
1893 B35 P30 Idiopathic BAD Post-cholecystectomy
0.9431731502669718
1894 B35 P33 Idiopathic BAD Post-cholecystectomy
0.9794050343249427
1895 B35 P35 Idiopathic BAD Post-cholecystectomy
0.9832189168573608
1896 B35 P38 Idiopathic BAD Post-cholecystectomy
0.9029366895499619
1897 B35 P39 Idiopathic BAD Post-cholecystectomy
0.8724256292906178
1898 B35 P42 Idiopathic BAD Post-cholecystectomy
0.9311594202898551
1899 B35 P43 Idiopathic BAD Post-cholecystectomy
0.8237986270022883
1900 B35 P46 Idiopathic BAD Post-cholecystectomy
0.9151411136536994
1901 B35 P47 Idiopathic BAD Post-cholecystectomy
0.9853165522501907
1902 B35 P50 Idiopathic BAD Post-cholecystectomy

0.9702517162471396
1903 B35 P55 Idiopathic BAD Post-cholecystectomy
0.9706331045003814
1904 B35 P58 Idiopathic BAD Post-cholecystectomy
0.9935163996948894
1905 B35 P60 Idiopathic BAD Post-cholecystectomy
0.7837528604118993
1906 B35 P63 Idiopathic BAD Post-cholecystectomy
0.9977116704805492
1907 B35 P65 Idiopathic BAD Post-cholecystectomy
0.9584286803966438
1908 B35 P68 Idiopathic BAD Post-cholecystectomy
0.8217009916094584
1909 B35 P70 Idiopathic BAD Post-cholecystectomy
0.8577421815408085
1910 B35 P71 Idiopathic BAD Post-cholecystectomy
0.9670099160945843
1911 B35 P74 Idiopathic BAD Post-cholecystectomy
0.971205186880244
1912 B35 P75 Idiopathic BAD Post-cholecystectomy
0.9521357742181541
1913 B39 P1 Idiopathic BAD Post-cholecystectomy
0.9178108314263921
1914 B39 P2 Idiopathic BAD Post-cholecystectomy
0.8537376048817696
1915 B39 P4 Idiopathic BAD Post-cholecystectomy
0.8792906178489702
1916 B39 P5 Idiopathic BAD Post-cholecystectomy
0.8255148741418764
1917 B39 P9 Idiopathic BAD Post-cholecystectomy
0.8260869565217391
1918 B39 P13 Idiopathic BAD Post-cholecystectomy
0.8703279938977879
1919 B39 P15 Idiopathic BAD Post-cholecystectomy
0.84744469870328
1920 B39 P16 Idiopathic BAD Post-cholecystectomy
0.8834858886346301
1921 B39 P17 Idiopathic BAD Post-cholecystectomy
0.8504958047292144
1922 B39 P20 Idiopathic BAD Post-cholecystectomy
0.7827993897787948
1923 B39 P21 Idiopathic BAD Post-cholecystectomy
0.812929061784897
1924 B39 P24 Idiopathic BAD Post-cholecystectomy
0.8966437833714722
1925 B39 P26 Idiopathic BAD Post-cholecystectomy
0.8882532418001525
1926 B39 P30 Idiopathic BAD Post-cholecystectomy
0.898741418764302
1927 B39 P33 Idiopathic BAD Post-cholecystectomy
0.8518306636155606
1928 B39 P35 Idiopathic BAD Post-cholecystectomy
0.8651792524790236
1929 B39 P38 Idiopathic BAD Post-cholecystectomy

0.9361174675819984
1930 B39 P39 Idiopathic BAD Post-cholecystectomy
0.8136918382913806
1931 B39 P42 Idiopathic BAD Post-cholecystectomy
0.8432494279176201
1932 B39 P43 Idiopathic BAD Post-cholecystectomy
0.88558352402746
1933 B39 P46 Idiopathic BAD Post-cholecystectomy
0.8478260869565217
1934 B39 P47 Idiopathic BAD Post-cholecystectomy
0.9132341723874905
1935 B39 P50 Idiopathic BAD Post-cholecystectomy
0.8426773455377574
1936 B39 P55 Idiopathic BAD Post-cholecystectomy
0.8653699466056446
1937 B39 P58 Idiopathic BAD Post-cholecystectomy
0.8558352402745996
1938 B39 P60 Idiopathic BAD Post-cholecystectomy
0.8865369946605645
1939 B39 P63 Idiopathic BAD Post-cholecystectomy
0.8726163234172387
1940 B39 P65 Idiopathic BAD Post-cholecystectomy
0.9101830663615561
1941 B39 P68 Idiopathic BAD Post-cholecystectomy
0.8342868039664378
1942 B39 P70 Idiopathic BAD Post-cholecystectomy
0.8878718535469108
1943 B39 P71 Idiopathic BAD Post-cholecystectomy
0.9048436308161708
1944 B39 P74 Idiopathic BAD Post-cholecystectomy
0.8785278413424866
1945 B39 P75 Idiopathic BAD Post-cholecystectomy
0.8222730739893211
1946 B43 P1 Idiopathic BAD Post-cholecystectomy
0.9628146453089245
1947 B43 P2 Idiopathic BAD Post-cholecystectomy
0.7934782608695652
1948 B43 P4 Idiopathic BAD Post-cholecystectomy
0.8766209000762777
1949 B43 P5 Idiopathic BAD Post-cholecystectomy
0.835812356979405
1950 B43 P9 Idiopathic BAD Post-cholecystectomy
0.9391685736079328
1951 B43 P13 Idiopathic BAD Post-cholecystectomy
0.6096491228070176
1952 B43 P15 Idiopathic BAD Post-cholecystectomy
0.7763157894736842
1953 B43 P16 Idiopathic BAD Post-cholecystectomy
0.9574752097635393
1954 B43 P17 Idiopathic BAD Post-cholecystectomy
0.8453470633104501
1955 B43 P20 Idiopathic BAD Post-cholecystectomy
0.822463768115942
1956 B43 P21 Idiopathic BAD Post-cholecystectomy

0.8705186880244088
1957	B43	P24	Idiopathic BAD	Post-cholecystectomy
0.7589626239511823
1958	B43	P26	Idiopathic BAD	Post-cholecystectomy
0.8493516399694889
1959	B43	P30	Idiopathic BAD	Post-cholecystectomy
0.9563310450038138
1960	B43	P33	Idiopathic BAD	Post-cholecystectomy
0.8476353928299009
1961	B43	P35	Idiopathic BAD	Post-cholecystectomy
0.8909229595728452
1962	B43	P38	Idiopathic BAD	Post-cholecystectomy
0.9658657513348589
1963	B43	P39	Idiopathic BAD	Post-cholecystectomy
0.9475591151792525
1964	B43	P42	Idiopathic BAD	Post-cholecystectomy
0.9683447749809306
1965	B43	P43	Idiopathic BAD	Post-cholecystectomy
0.7395118230358505
1966	B43	P46	Idiopathic BAD	Post-cholecystectomy
0.7149122807017544
1967	B43	P47	Idiopathic BAD	Post-cholecystectomy
0.7376048817696415
1968	B43	P50	Idiopathic BAD	Post-cholecystectomy
0.8297101449275363
1969	B43	P55	Idiopathic BAD	Post-cholecystectomy
0.8152173913043478
1970	B43	P58	Idiopathic BAD	Post-cholecystectomy
0.9315408085430968
1971	B43	P60	Idiopathic BAD	Post-cholecystectomy
0.9773073989321129
1972	B43	P63	Idiopathic BAD	Post-cholecystectomy
0.839626239511823
1973	B43	P65	Idiopathic BAD	Post-cholecystectomy
0.8836765827612509
1974	B43	P68	Idiopathic BAD	Post-cholecystectomy
0.891113653699466
1975	B43	P70	Idiopathic BAD	Post-cholecystectomy
0.8880625476735317
1976	B43	P71	Idiopathic BAD	Post-cholecystectomy
0.7534324942791762
1977	B43	P74	Idiopathic BAD	Post-cholecystectomy
0.8150266971777269
1978	B43	P75	Idiopathic BAD	Post-cholecystectomy
0.7364607170099161
1979	B47	P1	Idiopathic BAD	Post-cholecystectomy
0.9559496567505721
1980	B47	P2	Idiopathic BAD	Post-cholecystectomy
0.7854691075514875
1981	B47	P4	Idiopathic BAD	Post-cholecystectomy
0.809115179252479
1982	B47	P5	Idiopathic BAD	Post-cholecystectomy
0.6826849733028223
1983	B47	P9	Idiopathic BAD	Post-cholecystectomy

0.7250190694126621
1984 B47 P13 Idiopathic BAD Post-cholecystectomy
0.8573607932875668
1985 B47 P15 Idiopathic BAD Post-cholecystectomy
0.8197940503432495
1986 B47 P16 Idiopathic BAD Post-cholecystectomy
0.8180778032036613
1987 B47 P17 Idiopathic BAD Post-cholecystectomy
0.8083524027459954
1988 B47 P20 Idiopathic BAD Post-cholecystectomy
0.8190312738367659
1989 B47 P21 Idiopathic BAD Post-cholecystectomy
0.8070175438596491
1990 B47 P24 Idiopathic BAD Post-cholecystectomy
0.8548817696414951
1991 B47 P26 Idiopathic BAD Post-cholecystectomy
0.8466819221967964
1992 B47 P30 Idiopathic BAD Post-cholecystectomy
0.8796720061022121
1993 B47 P33 Idiopathic BAD Post-cholecystectomy
0.7820366132723112
1994 B47 P35 Idiopathic BAD Post-cholecystectomy
0.7568649885583524
1995 B47 P38 Idiopathic BAD Post-cholecystectomy
0.9534706331045004
1996 B47 P39 Idiopathic BAD Post-cholecystectomy
0.8861556064073226
1997 B47 P42 Idiopathic BAD Post-cholecystectomy
0.894927536231884
1998 B47 P43 Idiopathic BAD Post-cholecystectomy
0.8493516399694889
1999 B47 P46 Idiopathic BAD Post-cholecystectomy
0.8163615560640732
2000 B47 P47 Idiopathic BAD Post-cholecystectomy
0.9212433257055682
2001 B47 P50 Idiopathic BAD Post-cholecystectomy
0.6845919145690312
2002 B47 P55 Idiopathic BAD Post-cholecystectomy
0.7732646834477498
2003 B47 P58 Idiopathic BAD Post-cholecystectomy
0.7421815408085431
2004 B47 P60 Idiopathic BAD Post-cholecystectomy
0.9057971014492754
2005 B47 P63 Idiopathic BAD Post-cholecystectomy
0.7923340961098398
2006 B47 P65 Idiopathic BAD Post-cholecystectomy
0.7841342486651411
2007 B47 P68 Idiopathic BAD Post-cholecystectomy
0.7827993897787948
2008 B47 P70 Idiopathic BAD Post-cholecystectomy
0.820747520976354
2009 B47 P71 Idiopathic BAD Post-cholecystectomy
0.8373379099923722
2010 B47 P74 Idiopathic BAD Post-cholecystectomy

0.7572463768115942
2011 B47 P75 Idiopathic BAD Post-cholecystectomy
0.7051868802440885
2012 B48 P1 Idiopathic BAD Post-cholecystectomy
0.9761632341723875
2013 B48 P2 Idiopathic BAD Post-cholecystectomy
0.9605263157894737
2014 B48 P4 Idiopathic BAD Post-cholecystectomy
0.704233409610984
2015 B48 P5 Idiopathic BAD Post-cholecystectomy
0.8182684973302822
2016 B48 P9 Idiopathic BAD Post-cholecystectomy
0.7492372234935164
2017 B48 P13 Idiopathic BAD Post-cholecystectomy
0.9145690312738368
2018 B48 P15 Idiopathic BAD Post-cholecystectomy
0.8632723112128147
2019 B48 P16 Idiopathic BAD Post-cholecystectomy
0.6493135011441648
2020 B48 P17 Idiopathic BAD Post-cholecystectomy
0.9563310450038138
2021 B48 P20 Idiopathic BAD Post-cholecystectomy
0.5783752860411899
2022 B48 P21 Idiopathic BAD Post-cholecystectomy
0.6691456903127384
2023 B48 P24 Idiopathic BAD Post-cholecystectomy
0.9441266209000763
2024 B48 P26 Idiopathic BAD Post-cholecystectomy
0.8392448512585813
2025 B48 P30 Idiopathic BAD Post-cholecystectomy
0.8249427917620137
2026 B48 P33 Idiopathic BAD Post-cholecystectomy
0.8688024408848207
2027 B48 P35 Idiopathic BAD Post-cholecystectomy
0.9477498093058734
2028 B48 P38 Idiopathic BAD Post-cholecystectomy
0.912090007627765
2029 B48 P39 Idiopathic BAD Post-cholecystectomy
0.6882151029748284
2030 B48 P42 Idiopathic BAD Post-cholecystectomy
0.8958810068649885
2031 B48 P43 Idiopathic BAD Post-cholecystectomy
0.738367658276125
2032 B48 P46 Idiopathic BAD Post-cholecystectomy
0.8190312738367659
2033 B48 P47 Idiopathic BAD Post-cholecystectomy
0.9734935163996948
2034 B48 P50 Idiopathic BAD Post-cholecystectomy
0.8939740655987796
2035 B48 P55 Idiopathic BAD Post-cholecystectomy
0.84744469870328
2036 B48 P58 Idiopathic BAD Post-cholecystectomy
0.8668954996186118
2037 B48 P60 Idiopathic BAD Post-cholecystectomy

0.644927536231884
2038 B48 P63 Idiopathic BAD Post−cholecystectomy
0.9534706331045004
2039 B48 P65 Idiopathic BAD Post−cholecystectomy
0.9006483600305111
2040 B48 P68 Idiopathic BAD Post−cholecystectomy
0.793859649122807
2041 B48 P70 Idiopathic BAD Post−cholecystectomy
0.8859649122807017
2042 B48 P71 Idiopathic BAD Post−cholecystectomy
0.9527078565980168
2043 B48 P74 Idiopathic BAD Post−cholecystectomy
0.9477498093058734
2044 B48 P75 Idiopathic BAD Post−cholecystectomy
0.843440122044241
2045 B49 P1 Idiopathic BAD Post−cholecystectomy
0.9124713958810069
2046 B49 P2 Idiopathic BAD Post−cholecystectomy
0.8442028985507246
2047 B49 P4 Idiopathic BAD Post−cholecystectomy
0.7341723874904653
2048 B49 P5 Idiopathic BAD Post−cholecystectomy
0.8119755911517925
2049 B49 P9 Idiopathic BAD Post−cholecystectomy
0.9046529366895499
2050 B49 P13 Idiopathic BAD Post−cholecystectomy
0.7768878718535469
2051 B49 P15 Idiopathic BAD Post−cholecystectomy
0.8226544622425629
2052 B49 P16 Idiopathic BAD Post−cholecystectomy
0.7288329519450801
2053 B49 P17 Idiopathic BAD Post−cholecystectomy
0.7889016018306636
2054 B49 P20 Idiopathic BAD Post−cholecystectomy
0.6512204424103738
2055 B49 P21 Idiopathic BAD Post−cholecystectomy
0.9052250190694127
2056 B49 P24 Idiopathic BAD Post−cholecystectomy
0.7465675057208238
2057 B49 P26 Idiopathic BAD Post−cholecystectomy
0.8070175438596491
2058 B49 P30 Idiopathic BAD Post−cholecystectomy
0.9101830663615561
2059 B49 P33 Idiopathic BAD Post−cholecystectomy
0.8581235697940504
2060 B49 P35 Idiopathic BAD Post−cholecystectomy
0.8468726163234173
2061 B49 P38 Idiopathic BAD Post−cholecystectomy
0.9467963386727689
2062 B49 P39 Idiopathic BAD Post−cholecystectomy
0.8811975591151793
2063 B49 P42 Idiopathic BAD Post−cholecystectomy
0.8804347826086957
2064 B49 P43 Idiopathic BAD Post−cholecystectomy

0.7059496567505721
2065	B49	P46	Idiopathic BAD	Post-cholecystectomy
0.8030129672006102
2066	B49	P47	Idiopathic BAD	Post-cholecystectomy
0.90255530129672
2067	B49	P50	Idiopathic BAD	Post-cholecystectomy
0.8333333333333334
2068	B49	P55	Idiopathic BAD	Post-cholecystectomy
0.7991990846681922
2069	B49	P58	Idiopathic BAD	Post-cholecystectomy
0.7221586575133486
2070	B49	P60	Idiopathic BAD	Post-cholecystectomy
0.8247520976353928
2071	B49	P63	Idiopathic BAD	Post-cholecystectomy
0.7650648360030511
2072	B49	P65	Idiopathic BAD	Post-cholecystectomy
0.7622044241037376
2073	B49	P68	Idiopathic BAD	Post-cholecystectomy
0.7589626239511823
2074	B49	P70	Idiopathic BAD	Post-cholecystectomy
0.9239130434782609
2075	B49	P71	Idiopathic BAD	Post-cholecystectomy
0.9094202898550725
2076	B49	P74	Idiopathic BAD	Post-cholecystectomy
0.8384820747520977
2077	B49	P75	Idiopathic BAD	Post-cholecystectomy
0.8077803203661327
2078	B53	P1	Idiopathic BAD	Post-cholecystectomy
0.9622425629290617
2079	B53	P2	Idiopathic BAD	Post-cholecystectomy
0.8062547673531655
2080	B53	P4	Idiopathic BAD	Post-cholecystectomy
0.856788710907704
2081	B53	P5	Idiopathic BAD	Post-cholecystectomy
0.7776506483600305
2082	B53	P9	Idiopathic BAD	Post-cholecystectomy
0.7238749046529367
2083	B53	P13	Idiopathic BAD	Post-cholecystectomy
0.8499237223493517
2084	B53	P15	Idiopathic BAD	Post-cholecystectomy
0.8661327231121282
2085	B53	P16	Idiopathic BAD	Post-cholecystectomy
0.8527841342486652
2086	B53	P17	Idiopathic BAD	Post-cholecystectomy
0.8081617086193745
2087	B53	P20	Idiopathic BAD	Post-cholecystectomy
0.8747139588100686
2088	B53	P21	Idiopathic BAD	Post-cholecystectomy
0.8308543096872616
2089	B53	P24	Idiopathic BAD	Post-cholecystectomy
0.8954996186117468
2090	B53	P26	Idiopathic BAD	Post-cholecystectomy
0.6664759725400458
2091	B53	P30	Idiopathic BAD	Post-cholecystectomy

0.9061784897025171
2092	B53	P33	Idiopathic BAD	Post-cholecystectomy
0.8457284515636918
2093	B53	P35	Idiopathic BAD	Post-cholecystectomy
0.7946224256292906
2094	B53	P38	Idiopathic BAD	Post-cholecystectomy
0.9164759725400458
2095	B53	P39	Idiopathic BAD	Post-cholecystectomy
0.9221967963386728
2096	B53	P42	Idiopathic BAD	Post-cholecystectomy
0.7713577421815409
2097	B53	P43	Idiopathic BAD	Post-cholecystectomy
0.8991228070175439
2098	B53	P46	Idiopathic BAD	Post-cholecystectomy
0.8463005339435545
2099	B53	P47	Idiopathic BAD	Post-cholecystectomy
0.9218154080854309
2100	B53	P50	Idiopathic BAD	Post-cholecystectomy
0.7681159420289855
2101	B53	P55	Idiopathic BAD	Post-cholecystectomy
0.7459954233409611
2102	B53	P58	Idiopathic BAD	Post-cholecystectomy
0.7116704805491991
2103	B53	P60	Idiopathic BAD	Post-cholecystectomy
0.873951182303585
2104	B53	P63	Idiopathic BAD	Post-cholecystectomy
0.881578947368421
2105	B53	P65	Idiopathic BAD	Post-cholecystectomy
0.8123569794050344
2106	B53	P68	Idiopathic BAD	Post-cholecystectomy
0.7896643783371472
2107	B53	P70	Idiopathic BAD	Post-cholecystectomy
0.8018688024408849
2108	B53	P71	Idiopathic BAD	Post-cholecystectomy
0.864607170099161
2109	B53	P74	Idiopathic BAD	Post-cholecystectomy
0.7923340961098398
2110	B53	P75	Idiopathic BAD	Post-cholecystectomy
0.8545003813882532
2111	B54	P1	Idiopathic BAD	Post-cholecystectomy
0.9744469870327994
2112	B54	P2	Idiopathic BAD	Post-cholecystectomy
0.8804347826086957
2113	B54	P4	Idiopathic BAD	Post-cholecystectomy
0.8575514874141876
2114	B54	P5	Idiopathic BAD	Post-cholecystectomy
0.8581235697940504
2115	B54	P9	Idiopathic BAD	Post-cholecystectomy
0.8443935926773455
2116	B54	P13	Idiopathic BAD	Post-cholecystectomy
0.9218154080854309
2117	B54	P15	Idiopathic BAD	Post-cholecystectomy
0.709954233409611
2118	B54	P16	Idiopathic BAD	Post-cholecystectomy

0.8991228070175439
2119 B54 P17 Idiopathic BAD Post-cholecystectomy
0.9155225019069413
2120 B54 P20 Idiopathic BAD Post-cholecystectomy
0.8874904652936689
2121 B54 P21 Idiopathic BAD Post-cholecystectomy
0.8712814645308925
2122 B54 P24 Idiopathic BAD Post-cholecystectomy
0.9528985507246377
2123 B54 P26 Idiopathic BAD Post-cholecystectomy
0.8468726163234173
2124 B54 P30 Idiopathic BAD Post-cholecystectomy
0.9553775743707094
2125 B54 P33 Idiopathic BAD Post-cholecystectomy
0.8577421815408085
2126 B54 P35 Idiopathic BAD Post-cholecystectomy
0.7921434019832189
2127 B54 P38 Idiopathic BAD Post-cholecystectomy
0.8880625476735317
2128 B54 P39 Idiopathic BAD Post-cholecystectomy
0.9466056445461479
2129 B54 P42 Idiopathic BAD Post-cholecystectomy
0.9462242562929062
2130 B54 P43 Idiopathic BAD Post-cholecystectomy
0.9204805491990846
2131 B54 P46 Idiopathic BAD Post-cholecystectomy
0.7534324942791762
2132 B54 P47 Idiopathic BAD Post-cholecystectomy
0.6575133485888635
2133 B54 P50 Idiopathic BAD Post-cholecystectomy
0.8400076277650649
2134 B54 P55 Idiopathic BAD Post-cholecystectomy
0.8800533943554538
2135 B54 P58 Idiopathic BAD Post-cholecystectomy
0.8409610983981693
2136 B54 P60 Idiopathic BAD Post-cholecystectomy
0.9593821510297483
2137 B54 P63 Idiopathic BAD Post-cholecystectomy
0.9267734553775744
2138 B54 P65 Idiopathic BAD Post-cholecystectomy
0.839626239511823
2139 B54 P68 Idiopathic BAD Post-cholecystectomy
0.9176201372997712
2140 B54 P70 Idiopathic BAD Post-cholecystectomy
0.8996948893974066
2141 B54 P71 Idiopathic BAD Post-cholecystectomy
0.7170099160945843
2142 B54 P74 Idiopathic BAD Post-cholecystectomy
0.8813882532418001
2143 B54 P75 Idiopathic BAD Post-cholecystectomy
0.8876811594202898
2144 B55 P1 Idiopathic BAD Post-cholecystectomy
0.984744469870328
2145 B55 P2 Idiopathic BAD Post-cholecystectomy

0.8699466056445462
2146 B55 P4 Idiopathic BAD Post-cholecystectomy
0.9509916094584286
2147 B55 P5 Idiopathic BAD Post-cholecystectomy
0.8598398169336384
2148 B55 P9 Idiopathic BAD Post-cholecystectomy
0.8201754385964912
2149 B55 P13 Idiopathic BAD Post-cholecystectomy
0.950228832951945
2150 B55 P15 Idiopathic BAD Post-cholecystectomy
0.7608695652173914
2151 B55 P16 Idiopathic BAD Post-cholecystectomy
0.8861556064073226
2152 B55 P17 Idiopathic BAD Post-cholecystectomy
0.9073226544622426
2153 B55 P20 Idiopathic BAD Post-cholecystectomy
0.9258199847444699
2154 B55 P21 Idiopathic BAD Post-cholecystectomy
0.8724256292906178
2155 B55 P24 Idiopathic BAD Post-cholecystectomy
0.9637681159420289
2156 B55 P26 Idiopathic BAD Post-cholecystectomy
0.8874904652936689
2157 B55 P30 Idiopathic BAD Post-cholecystectomy
0.969488939740656
2158 B55 P33 Idiopathic BAD Post-cholecystectomy
0.9057971014492754
2159 B55 P35 Idiopathic BAD Post-cholecystectomy
0.8539282990083905
2160 B55 P38 Idiopathic BAD Post-cholecystectomy
0.9824561403508771
2161 B55 P39 Idiopathic BAD Post-cholecystectomy
0.9668192219679634
2162 B55 P42 Idiopathic BAD Post-cholecystectomy
0.7585812356979404
2163 B55 P43 Idiopathic BAD Post-cholecystectomy
0.9595728451563692
2164 B55 P46 Idiopathic BAD Post-cholecystectomy
0.8058733790999237
2165 B55 P47 Idiopathic BAD Post-cholecystectomy
0.5661708619374524
2166 B55 P50 Idiopathic BAD Post-cholecystectomy
0.8503051106025934
2167 B55 P55 Idiopathic BAD Post-cholecystectomy
0.8663234172387491
2168 B55 P58 Idiopathic BAD Post-cholecystectomy
0.8764302059496567
2169 B55 P60 Idiopathic BAD Post-cholecystectomy
0.9849351639969489
2170 B55 P63 Idiopathic BAD Post-cholecystectomy
0.9288710907704043
2171 B55 P65 Idiopathic BAD Post-cholecystectomy
0.8478260869565217
2172 B55 P68 Idiopathic BAD Post-cholecystectomy

0.8539282990083905
2173 B55 P70 Idiopathic BAD Post-cholecystectomy
0.8518306636155606
2174 B55 P71 Idiopathic BAD Post-cholecystectomy
0.7768878718535469
2175 B55 P74 Idiopathic BAD Post-cholecystectomy
0.921434019832189 2
2176 B55 P75 Idiopathic BAD Post-cholecystectomy
0.9448893974065599
2177 B59 P1 Idiopathic BAD Post-cholecystectomy
0.8535469107551488
2178 B59 P2 Idiopathic BAD Post-cholecystectomy
0.9464149504195271
2179 B59 P4 Idiopathic BAD Post-cholecystectomy
0.9822654462242563
2180 B59 P5 Idiopathic BAD Post-cholecystectomy
0.9691075514874142
2181 B59 P9 Idiopathic BAD Post-cholecystectomy
0.9933257055682685
2182 B59 P13 Idiopathic BAD Post-cholecystectomy
0.9872234935163997
2183 B59 P15 Idiopathic BAD Post-cholecystectomy
0.9454614797864226
2184 B59 P16 Idiopathic BAD Post-cholecystectomy
0.9609077040427155
2185 B59 P17 Idiopathic BAD Post-cholecystectomy
0.9872234935163997
2186 B59 P20 Idiopathic BAD Post-cholecystectomy
0.9096109839816934
2187 B59 P21 Idiopathic BAD Post-cholecystectomy
0.9849351639969489
2188 B59 P24 Idiopathic BAD Post-cholecystectomy
0.9782608695652174
2189 B59 P26 Idiopathic BAD Post-cholecystectomy
0.9832189168573608
2190 B59 P30 Idiopathic BAD Post-cholecystectomy
0.9546147978642258
2191 B59 P33 Idiopathic BAD Post-cholecystectomy
0.9610983981693364
2192 B59 P35 Idiopathic BAD Post-cholecystectomy
0.9881769641495042
2193 B59 P38 Idiopathic BAD Post-cholecystectomy
0.9195270785659801
2194 B59 P39 Idiopathic BAD Post-cholecystectomy
0.9164759725400458
2195 B59 P42 Idiopathic BAD Post-cholecystectomy
0.9509916094584286
2196 B59 P43 Idiopathic BAD Post-cholecystectomy
0.9877955758962624
2197 B59 P46 Idiopathic BAD Post-cholecystectomy
0.9757818459191457
2198 B59 P47 Idiopathic BAD Post-cholecystectomy
0.9490846681922197
2199 B59 P50 Idiopathic BAD Post-cholecystectomy

0.9721586575133486
2200 B59 P55 Idiopathic BAD Post-cholecystectomy
0.9746376811594203
2201 B59 P58 Idiopathic BAD Post-cholecystectomy
0.9921815408085431
2202 B59 P60 Idiopathic BAD Post-cholecystectomy
0.9839816933638444
2203 B59 P63 Idiopathic BAD Post-cholecystectomy
0.9706331045003814
2204 B59 P65 Idiopathic BAD Post-cholecystectomy
0.9841723874904653
2205 B59 P68 Idiopathic BAD Post-cholecystectomy
0.9759725400457666
2206 B59 P70 Idiopathic BAD Post-cholecystectomy
0.9631960335621663
2207 B59 P71 Idiopathic BAD Post-cholecystectomy
0.9891304347826086
2208 B59 P74 Idiopathic BAD Post-cholecystectomy
0.9628146453089245
2209 B59 P75 Idiopathic BAD Post-cholecystectomy
0.9876048817696415
2210 B70 P1 Idiopathic BAD Post-cholecystectomy
0.979023646071701
2211 B70 P2 Idiopathic BAD Post-cholecystectomy
0.7906178489702517
2212 B70 P4 Idiopathic BAD Post-cholecystectomy
0.8749046529366895
2213 B70 P5 Idiopathic BAD Post-cholecystectomy
0.8056826849733029
2214 B70 P9 Idiopathic BAD Post-cholecystectomy
0.7107170099160945
2215 B70 P13 Idiopathic BAD Post-cholecystectomy
0.9380244088482075
2216 B70 P15 Idiopathic BAD Post-cholecystectomy
0.9326849733028223
2217 B70 P16 Idiopathic BAD Post-cholecystectomy
0.8009153318077803
2218 B70 P17 Idiopathic BAD Post-cholecystectomy
0.8661327231121282
2219 B70 P20 Idiopathic BAD Post-cholecystectomy
0.8537376048817696
2220 B70 P21 Idiopathic BAD Post-cholecystectomy
0.7065217391304348
2221 B70 P24 Idiopathic BAD Post-cholecystectomy
0.9405034324942791
2222 B70 P26 Idiopathic BAD Post-cholecystectomy
0.7124332570556827
2223 B70 P30 Idiopathic BAD Post-cholecystectomy
0.9639588100686499
2224 B70 P33 Idiopathic BAD Post-cholecystectomy
0.6720061022120518
2225 B70 P35 Idiopathic BAD Post-cholecystectomy
0.7858504958047292
2226 B70 P38 Idiopathic BAD Post-cholecystectomy

0.9080854309687262
2227 B70 P39 Idiopathic BAD Post-cholecystectomy
0.9151411136536994
2228 B70 P42 Idiopathic BAD Post-cholecystectomy
0.6710526315789473
2229 B70 P43 Idiopathic BAD Post-cholecystectomy
0.9477498093058734
2230 B70 P46 Idiopathic BAD Post-cholecystectomy
0.7965293668954996
2231 B70 P47 Idiopathic BAD Post-cholecystectomy
0.9672006102212052
2232 B70 P50 Idiopathic BAD Post-cholecystectomy
0.6826849733028223
2233 B70 P55 Idiopathic BAD Post-cholecystectomy
0.7883295194508009
2234 B70 P58 Idiopathic BAD Post-cholecystectomy
0.6735316552250191
2235 B70 P60 Idiopathic BAD Post-cholecystectomy
0.761441647597254
2236 B70 P63 Idiopathic BAD Post-cholecystectomy
0.881769641495042
2237 B70 P65 Idiopathic BAD Post-cholecystectomy
0.8171243325705568
2238 B70 P68 Idiopathic BAD Post-cholecystectomy
0.6632341723874905
2239 B70 P70 Idiopathic BAD Post-cholecystectomy
0.7215865751334859
2240 B70 P71 Idiopathic BAD Post-cholecystectomy
0.8960717009916095
2241 B70 P74 Idiopathic BAD Post-cholecystectomy
0.7934782608695652
2242 B70 P75 Idiopathic BAD Post-cholecystectomy
0.8407704042715485
2243 B74 P1 Idiopathic BAD Post-cholecystectomy
0.9652936689549961
2244 B74 P2 Idiopathic BAD Post-cholecystectomy
0.8251334858886347
2245 B74 P4 Idiopathic BAD Post-cholecystectomy
0.8676582761250954
2246 B74 P5 Idiopathic BAD Post-cholecystectomy
0.8173150266971777
2247 B74 P9 Idiopathic BAD Post-cholecystectomy
0.9096109839816934
2248 B74 P13 Idiopathic BAD Post-cholecystectomy
0.9338291380625476
2249 B74 P15 Idiopathic BAD Post-cholecystectomy
0.78813882532418
2250 B74 P16 Idiopathic BAD Post-cholecystectomy
0.8754767353165522
2251 B74 P17 Idiopathic BAD Post-cholecystectomy
0.8926392067124332
2252 B74 P20 Idiopathic BAD Post-cholecystectomy
0.9405034324942791
2253 B74 P21 Idiopathic BAD Post-cholecystectomy

0.9176201372997712
2254 B74 P24 Idiopathic BAD Post−cholecystectomy
0.7759344012204424
2255 B74 P26 Idiopathic BAD Post−cholecystectomy
0.9319221967963387
2256 B74 P30 Idiopathic BAD Post−cholecystectomy
0.9101830663615561
2257 B74 P33 Idiopathic BAD Post−cholecystectomy
0.897025171624714
2258 B74 P35 Idiopathic BAD Post−cholecystectomy
0.9117086193745233
2259 B74 P38 Idiopathic BAD Post−cholecystectomy
0.9210526315789473
2260 B74 P39 Idiopathic BAD Post−cholecystectomy
0.9214340198321892
2261 B74 P42 Idiopathic BAD Post−cholecystectomy
0.9387871853546911
2262 B74 P43 Idiopathic BAD Post−cholecystectomy
0.9124713958810069
2263 B74 P46 Idiopathic BAD Post−cholecystectomy
0.9139969488939741
2264 B74 P47 Idiopathic BAD Post−cholecystectomy
0.9534706331045004
2265 B74 P50 Idiopathic BAD Post−cholecystectomy
0.8375286041189931
2266 B74 P55 Idiopathic BAD Post−cholecystectomy
0.8049199084668193
2267 B74 P58 Idiopathic BAD Post−cholecystectomy
0.9004576659038902
2268 B74 P60 Idiopathic BAD Post−cholecystectomy
0.988367658276125
2269 B74 P63 Idiopathic BAD Post−cholecystectomy
0.8321891685736079
2270 B74 P65 Idiopathic BAD Post−cholecystectomy
0.877765064836003
2271 B74 P68 Idiopathic BAD Post−cholecystectomy
0.8659420289855072
2272 B74 P70 Idiopathic BAD Post−cholecystectomy
0.9012204424103738
2273 B74 P71 Idiopathic BAD Post−cholecystectomy
0.9422196796338673
2274 B74 P74 Idiopathic BAD Post−cholecystectomy
0.834096109839817
2275 B74 P75 Idiopathic BAD Post−cholecystectomy
0.8121662852784134
2276 B77 P1 Idiopathic BAD Post−cholecystectomy
0.8844393592677345
2277 B77 P2 Idiopathic BAD Post−cholecystectomy
0.7833714721586575
2278 B77 P4 Idiopathic BAD Post−cholecystectomy
0.885202135774218
2279 B77 P5 Idiopathic BAD Post−cholecystectomy
0.7345537757437071
2280 B77 P9 Idiopathic BAD Post−cholecystectomy

0.7650648360030511
2281 B77 P13 Idiopathic BAD Post-cholecystectomy
0.8802440884820748
2282 B77 P15 Idiopathic BAD Post-cholecystectomy
0.852974828375286
2283 B77 P16 Idiopathic BAD Post-cholecystectomy
0.8394355453852022
2284 B77 P17 Idiopathic BAD Post-cholecystectomy
0.8426773455377574
2285 B77 P20 Idiopathic BAD Post-cholecystectomy
0.8794813119755912
2286 B77 P21 Idiopathic BAD Post-cholecystectomy
0.8117848970251716
2287 B77 P24 Idiopathic BAD Post-cholecystectomy
0.8657513348588863
2288 B77 P26 Idiopathic BAD Post-cholecystectomy
0.8184591914569032
2289 B77 P30 Idiopathic BAD Post-cholecystectomy
0.8852021357742181
2290 B77 P33 Idiopathic BAD Post-cholecystectomy
0.822463768115942
2291 B77 P35 Idiopathic BAD Post-cholecystectomy
0.7316933638443935
2292 B77 P38 Idiopathic BAD Post-cholecystectomy
0.9054157131960335
2293 B77 P39 Idiopathic BAD Post-cholecystectomy
0.9200991609458429
2294 B77 P42 Idiopathic BAD Post-cholecystectomy
0.847254004576659
2295 B77 P43 Idiopathic BAD Post-cholecystectomy
0.8934019832189168
2296 B77 P46 Idiopathic BAD Post-cholecystectomy
0.8665141113653699
2297 B77 P47 Idiopathic BAD Post-cholecystectomy
0.9328756674294432
2298 B77 P50 Idiopathic BAD Post-cholecystectomy
0.7076659038901602
2299 B77 P55 Idiopathic BAD Post-cholecystectomy
0.7553394355453852
2300 B77 P58 Idiopathic BAD Post-cholecystectomy
0.7917620137299771
2301 B77 P60 Idiopathic BAD Post-cholecystectomy
0.9609077040427155
2302 B77 P63 Idiopathic BAD Post-cholecystectomy
0.8276125095347063
2303 B77 P65 Idiopathic BAD Post-cholecystectomy
0.7715484363081617
2304 B77 P68 Idiopathic BAD Post-cholecystectomy
0.7200610221205187
2305 B77 P70 Idiopathic BAD Post-cholecystectomy
0.7669717772692601
2306 B77 P71 Idiopathic BAD Post-cholecystectomy
0.7948131197559115
2307 B77 P74 Idiopathic BAD Post-cholecystectomy

0.8285659801678108
2308 B77 P75 Idiopathic BAD Post-cholecystectomy
0.729023646071701
2309 B81 P1 Idiopathic BAD Post-cholecystectomy
0.876048817696415
2310 B81 P2 Idiopathic BAD Post-cholecystectomy
0.8861556064073226
2311 B81 P4 Idiopathic BAD Post-cholecystectomy
0.7972921434019832
2312 B81 P5 Idiopathic BAD Post-cholecystectomy
0.8270404271548436
2313 B81 P9 Idiopathic BAD Post-cholecystectomy
0.873951182303585
2314 B81 P13 Idiopathic BAD Post-cholecystectomy
0.8400076277650649
2315 B81 P15 Idiopathic BAD Post-cholecystectomy
0.8537376048817696
2316 B81 P16 Idiopathic BAD Post-cholecystectomy
0.8590770404271548
2317 B81 P17 Idiopathic BAD Post-cholecystectomy
0.7936689549961862
2318 B81 P20 Idiopathic BAD Post-cholecystectomy
0.843440122044241
2319 B81 P21 Idiopathic BAD Post-cholecystectomy
0.864607170099161
2320 B81 P24 Idiopathic BAD Post-cholecystectomy
0.7990083905415714
2321 B81 P26 Idiopathic BAD Post-cholecystectomy
0.8548817696414951
2322 B81 P30 Idiopathic BAD Post-cholecystectomy
0.885392829900839
2323 B81 P33 Idiopathic BAD Post-cholecystectomy
0.8565980167810832
2324 B81 P35 Idiopathic BAD Post-cholecystectomy
0.8974065598779558
2325 B81 P38 Idiopathic BAD Post-cholecystectomy
0.9393592677345538
2326 B81 P39 Idiopathic BAD Post-cholecystectomy
0.8405797101449275
2327 B81 P42 Idiopathic BAD Post-cholecystectomy
0.9324942791762014
2328 B81 P43 Idiopathic BAD Post-cholecystectomy
0.7858504958047292
2329 B81 P46 Idiopathic BAD Post-cholecystectomy
0.8504958047292144
2330 B81 P47 Idiopathic BAD Post-cholecystectomy
0.918001525553013
2331 B81 P50 Idiopathic BAD Post-cholecystectomy
0.8392448512585813
2332 B81 P55 Idiopathic BAD Post-cholecystectomy
0.8531655225019069
2333 B81 P58 Idiopathic BAD Post-cholecystectomy
0.8827231121281465
2334 B81 P60 Idiopathic BAD Post-cholecystectomy

0.9227688787185355
2335	B81	P63	Idiopathic BAD	Post-cholecystectomy
0.7837528604118993
2336	B81	P65	Idiopathic BAD	Post-cholecystectomy
0.8390541571319603
2337	B81	P68	Idiopathic BAD	Post-cholecystectomy
0.8863463005339436
2338	B81	P70	Idiopathic BAD	Post-cholecystectomy
0.8726163234172387
2339	B81	P71	Idiopathic BAD	Post-cholecystectomy
0.8749046529366895
2340	B81	P74	Idiopathic BAD	Post-cholecystectomy
0.8281845919145691
2341	B81	P75	Idiopathic BAD	Post-cholecystectomy
0.8096872616323417
2342	B84	P1	Idiopathic BAD	Post-cholecystectomy
0.9010297482837528
2343	B84	P2	Idiopathic BAD	Post-cholecystectomy
0.7677345537757437
2344	B84	P4	Idiopathic BAD	Post-cholecystectomy
0.8659420289855072
2345	B84	P5	Idiopathic BAD	Post-cholecystectomy
0.7873760488176964
2346	B84	P9	Idiopathic BAD	Post-cholecystectomy
0.8594584286803967
2347	B84	P13	Idiopathic BAD	Post-cholecystectomy
0.8150266971777269
2348	B84	P15	Idiopathic BAD	Post-cholecystectomy
0.8951182303585049
2349	B84	P16	Idiopathic BAD	Post-cholecystectomy
0.8525934401220442
2350	B84	P17	Idiopathic BAD	Post-cholecystectomy
0.8239893211289092
2351	B84	P20	Idiopathic BAD	Post-cholecystectomy
0.8154080854309688
2352	B84	P21	Idiopathic BAD	Post-cholecystectomy
0.7200610221205187
2353	B84	P24	Idiopathic BAD	Post-cholecystectomy
0.893211289092296
2354	B84	P26	Idiopathic BAD	Post-cholecystectomy
0.7591533180778032
2355	B84	P30	Idiopathic BAD	Post-cholecystectomy
0.9344012204424104
2356	B84	P33	Idiopathic BAD	Post-cholecystectomy
0.7864225781845919
2357	B84	P35	Idiopathic BAD	Post-cholecystectomy
0.7717391304347826
2358	B84	P38	Idiopathic BAD	Post-cholecystectomy
0.9206712433257056
2359	B84	P39	Idiopathic BAD	Post-cholecystectomy
0.9185736079328757
2360	B84	P42	Idiopathic BAD	Post-cholecystectomy
0.8806254767353165
2361	B84	P43	Idiopathic BAD	Post-cholecystectomy

0.7858504958047292
2362 B84 P46 Idiopathic BAD Post-cholecystectomy
0.8731884057971014
2363 B84 P47 Idiopathic BAD Post-cholecystectomy
0.908276125095347
2364 B84 P50 Idiopathic BAD Post-cholecystectomy
0.8056826849733029
2365 B84 P55 Idiopathic BAD Post-cholecystectomy
0.8209382151029748
2366 B84 P58 Idiopathic BAD Post-cholecystectomy
0.8371472158657514
2367 B84 P60 Idiopathic BAD Post-cholecystectomy
0.9458428680396643
2368 B84 P63 Idiopathic BAD Post-cholecystectomy
0.8907322654462243
2369 B84 P65 Idiopathic BAD Post-cholecystectomy
0.8392448512585813
2370 B84 P68 Idiopathic BAD Post-cholecystectomy
0.831998474446987
2371 B84 P70 Idiopathic BAD Post-cholecystectomy
0.8308543096872616
2372 B84 P71 Idiopathic BAD Post-cholecystectomy
0.8323798627002288
2373 B84 P74 Idiopathic BAD Post-cholecystectomy
0.5636918382913806
2374 B84 P75 Idiopathic BAD Post-cholecystectomy
0.8628909229595728
2375 B89 P1 Idiopathic BAD Post-cholecystectomy
0.9584286803966438
2376 B89 P2 Idiopathic BAD Post-cholecystectomy
0.7967200610221206
2377 B89 P4 Idiopathic BAD Post-cholecystectomy
0.8022501906941266
2378 B89 P5 Idiopathic BAD Post-cholecystectomy
0.8432494279176201
2379 B89 P9 Idiopathic BAD Post-cholecystectomy
0.8901601830663616
2380 B89 P13 Idiopathic BAD Post-cholecystectomy
0.795957284515637
2381 B89 P15 Idiopathic BAD Post-cholecystectomy
0.8264683447749809
2382 B89 P16 Idiopathic BAD Post-cholecystectomy
0.8411517925247902
2383 B89 P17 Idiopathic BAD Post-cholecystectomy
0.8133104500381388
2384 B89 P20 Idiopathic BAD Post-cholecystectomy
0.7307398932112891
2385 B89 P21 Idiopathic BAD Post-cholecystectomy
0.8239893211289092
2386 B89 P24 Idiopathic BAD Post-cholecystectomy
0.860602593440122
2387 B89 P26 Idiopathic BAD Post-cholecystectomy
0.8615560640732265
2388 B89 P30 Idiopathic BAD Post-cholecystectomy

0.9729214340198322
2389 B89 P33 Idiopathic BAD Post−cholecystectomy
0.8419145690312738
2390 B89 P35 Idiopathic BAD Post−cholecystectomy
0.8422959572845157
2391 B89 P38 Idiopathic BAD Post−cholecystectomy
0.9757818459191457
2392 B89 P39 Idiopathic BAD Post−cholecystectomy
0.839626239511823
2393 B89 P42 Idiopathic BAD Post−cholecystectomy
0.9414569031273837
2394 B89 P43 Idiopathic BAD Post−cholecystectomy
0.7738367658276125
2395 B89 P46 Idiopathic BAD Post−cholecystectomy
0.7921434019832189
2396 B89 P47 Idiopathic BAD Post−cholecystectomy
0.9631960335621663
2397 B89 P50 Idiopathic BAD Post−cholecystectomy
0.8272311212814645
2398 B89 P55 Idiopathic BAD Post−cholecystectomy
0.8194126620900076
2399 B89 P58 Idiopathic BAD Post−cholecystectomy
0.8766209000762777
2400 B89 P60 Idiopathic BAD Post−cholecystectomy
0.9113272311212814
2401 B89 P63 Idiopathic BAD Post−cholecystectomy
0.8314263920671243
2402 B89 P65 Idiopathic BAD Post−cholecystectomy
0.8459191456903128
2403 B89 P68 Idiopathic BAD Post−cholecystectomy
0.931350114416476
2404 B89 P70 Idiopathic BAD Post−cholecystectomy
0.8348588863463006
2405 B89 P71 Idiopathic BAD Post−cholecystectomy
0.8695652173913043
2406 B89 P74 Idiopathic BAD Post−cholecystectomy
0.8304729214340199
2407 B89 P75 Idiopathic BAD Post−cholecystectomy
0.8146453089244852
2408 B92 P1 Idiopathic BAD Post−cholecystectomy
0.9216247139588101
2409 B92 P2 Idiopathic BAD Post−cholecystectomy
0.7479023646071701
2410 B92 P4 Idiopathic BAD Post−cholecystectomy
0.7648741418764302
2411 B92 P5 Idiopathic BAD Post−cholecystectomy
0.7717391304347826
2412 B92 P9 Idiopathic BAD Post−cholecystectomy
0.7986270022883295
2413 B92 P13 Idiopathic BAD Post−cholecystectomy
0.8409610983981693
2414 B92 P15 Idiopathic BAD Post−cholecystectomy
0.7019450800915332
2415 B92 P16 Idiopathic BAD Post−cholecystectomy

0.8253241800152555
2416 B92 P17 Idiopathic BAD Post-cholecystectomy
0.8260869565217391
2417 B92 P20 Idiopathic BAD Post-cholecystectomy
0.780511060259344
2418 B92 P21 Idiopathic BAD Post-cholecystectomy
0.7276887871853547
2419 B92 P24 Idiopathic BAD Post-cholecystectomy
0.8668954996186118
2420 B92 P26 Idiopathic BAD Post-cholecystectomy
0.8173150266971777
2421 B92 P30 Idiopathic BAD Post-cholecystectomy
0.9290617848970252
2422 B92 P33 Idiopathic BAD Post-cholecystectomy
0.8106407322654462
2423 B92 P35 Idiopathic BAD Post-cholecystectomy
0.7673531655225019
2424 B92 P38 Idiopathic BAD Post-cholecystectomy
0.9029366895499619
2425 B92 P39 Idiopathic BAD Post-cholecystectomy
0.8710907704042715
2426 B92 P42 Idiopathic BAD Post-cholecystectomy
0.876048817696415
2427 B92 P43 Idiopathic BAD Post-cholecystectomy
0.8714721586575134
2428 B92 P46 Idiopathic BAD Post-cholecystectomy
0.6193745232646835
2429 B92 P47 Idiopathic BAD Post-cholecystectomy
0.5770404271548436
2430 B92 P50 Idiopathic BAD Post-cholecystectomy
0.7273073989321129
2431 B92 P55 Idiopathic BAD Post-cholecystectomy
0.7444698703279939
2432 B92 P58 Idiopathic BAD Post-cholecystectomy
0.7734553775743707
2433 B92 P60 Idiopathic BAD Post-cholecystectomy
0.9170480549199085
2434 B92 P63 Idiopathic BAD Post-cholecystectomy
0.7879481311975591
2435 B92 P65 Idiopathic BAD Post-cholecystectomy
0.8184591914569032
2436 B92 P68 Idiopathic BAD Post-cholecystectomy
0.8508771929824561
2437 B92 P70 Idiopathic BAD Post-cholecystectomy
0.7894736842105263
2438 B92 P71 Idiopathic BAD Post-cholecystectomy
0.7299771167048055
2439 B92 P74 Idiopathic BAD Post-cholecystectomy
0.7192982456140351
2440 B92 P75 Idiopathic BAD Post-cholecystectomy
0.7734553775743707
2441 B95 P1 Idiopathic BAD Post-cholecystectomy
0.9605263157894737
2442 B95 P2 Idiopathic BAD Post-cholecystectomy

0.8779557589626239
2443 B95 P4 Idiopathic BAD Post-cholecystectomy
0.84744469870328
2444 B95 P5 Idiopathic BAD Post-cholecystectomy
0.8409610983981693
2445 B95 P9 Idiopathic BAD Post-cholecystectomy
0.894927536231884
2446 B95 P13 Idiopathic BAD Post-cholecystectomy
0.8257055682684973
2447 B95 P15 Idiopathic BAD Post-cholecystectomy
0.7551487414187643
2448 B95 P16 Idiopathic BAD Post-cholecystectomy
0.8342868039664378
2449 B95 P17 Idiopathic BAD Post-cholecystectomy
0.8579328756674295
2450 B95 P20 Idiopathic BAD Post-cholecystectomy
0.7395118230358505
2451 B95 P21 Idiopathic BAD Post-cholecystectomy
0.851067887109077
2452 B95 P24 Idiopathic BAD Post-cholecystectomy
0.8779557589626239
2453 B95 P26 Idiopathic BAD Post-cholecystectomy
0.8556445461479787
2454 B95 P30 Idiopathic BAD Post-cholecystectomy
0.9250572082379863
2455 B95 P33 Idiopathic BAD Post-cholecystectomy
0.8941647597254004
2456 B95 P35 Idiopathic BAD Post-cholecystectomy
0.8140732265446224
2457 B95 P38 Idiopathic BAD Post-cholecystectomy
0.9689168573607932
2458 B95 P39 Idiopathic BAD Post-cholecystectomy
0.8455377574370709
2459 B95 P42 Idiopathic BAD Post-cholecystectomy
0.9731121281464531
2460 B95 P43 Idiopathic BAD Post-cholecystectomy
0.8012967200610221
2461 B95 P46 Idiopathic BAD Post-cholecystectomy
0.6500762776506483
2462 B95 P47 Idiopathic BAD Post-cholecystectomy
0.6567505720823799
2463 B95 P50 Idiopathic BAD Post-cholecystectomy
0.8842486651411137
2464 B95 P55 Idiopathic BAD Post-cholecystectomy
0.8792906178489702
2465 B95 P58 Idiopathic BAD Post-cholecystectomy
0.8308543096872616
2466 B95 P60 Idiopathic BAD Post-cholecystectomy
0.8972158657513348
2467 B95 P63 Idiopathic BAD Post-cholecystectomy
0.9153318077803204
2468 B95 P65 Idiopathic BAD Post-cholecystectomy
0.8790999237223494
2469 B95 P68 Idiopathic BAD Post-cholecystectomy

0.919717772692601
2470 B95 P70 Idiopathic BAD Post-cholecystectomy
0.8564073226544623
2471 B95 P71 Idiopathic BAD Post-cholecystectomy
0.8026315789473685
2472 B95 P74 Idiopathic BAD Post-cholecystectomy
0.8913043478260869
2473 B95 P75 Idiopathic BAD Post-cholecystectomy
0.8350495804729214
2474 B99 P1 Idiopathic BAD Post-cholecystectomy
0.950228832951945
2475 B99 P2 Idiopathic BAD Post-cholecystectomy
0.7145308924485125
2476 B99 P4 Idiopathic BAD Post-cholecystectomy
0.6950800915331807
2477 B99 P5 Idiopathic BAD Post-cholecystectomy
0.7109077040427155
2478 B99 P9 Idiopathic BAD Post-cholecystectomy
0.791952707856598
2479 B99 P13 Idiopathic BAD Post-cholecystectomy
0.6668573607932876
2480 B99 P15 Idiopathic BAD Post-cholecystectomy
0.7782227307398932
2481 B99 P16 Idiopathic BAD Post-cholecystectomy
0.7356979405034325
2482 B99 P17 Idiopathic BAD Post-cholecystectomy
0.7429443173150267
2483 B99 P20 Idiopathic BAD Post-cholecystectomy
0.6498855835240275
2484 B99 P21 Idiopathic BAD Post-cholecystectomy
0.7587719298245614
2485 B99 P24 Idiopathic BAD Post-cholecystectomy
0.7475209763539283
2486 B99 P26 Idiopathic BAD Post-cholecystectomy
0.7114797864225781
2487 B99 P30 Idiopathic BAD Post-cholecystectomy
0.9220061022120518
2488 B99 P33 Idiopathic BAD Post-cholecystectomy
0.7227307398932112
2489 B99 P35 Idiopathic BAD Post-cholecystectomy
0.7683066361556065
2490 B99 P38 Idiopathic BAD Post-cholecystectomy
0.9681540808543097
2491 B99 P39 Idiopathic BAD Post-cholecystectomy
0.8058733790999237
2492 B99 P42 Idiopathic BAD Post-cholecystectomy
0.8327612509534706
2493 B99 P43 Idiopathic BAD Post-cholecystectomy
0.7208237986270023
2494 B99 P46 Idiopathic BAD Post-cholecystectomy
0.6662852784134249
2495 B99 P47 Idiopathic BAD Post-cholecystectomy
0.9088482074752098
2496 B99 P50 Idiopathic BAD Post-cholecystectomy

0.7278794813119756
2497 B99 P55 Idiopathic BAD Post-cholecystectomy
0.7090007627765065
2498 B99 P58 Idiopathic BAD Post-cholecystectomy
0.6542715484363082
2499 B99 P60 Idiopathic BAD Post-cholecystectomy
0.8295194508009154
2500 B99 P63 Idiopathic BAD Post-cholecystectomy
0.658276125095347
2501 B99 P65 Idiopathic BAD Post-cholecystectomy
0.7648741418764302
2502 B99 P68 Idiopathic BAD Post-cholecystectomy
0.7273073989321129
2503 B99 P70 Idiopathic BAD Post-cholecystectomy
0.8192219679633868
2504 B99 P71 Idiopathic BAD Post-cholecystectomy
0.8419145690312738
2505 B99 P74 Idiopathic BAD Post-cholecystectomy
0.6948893974065599
2506 B99 P75 Idiopathic BAD Post-cholecystectomy
0.6643783371472158
2507 B103 P1 Idiopathic BAD Post-cholecystectomy
0.9889397406559878
2508 B103 P2 Idiopathic BAD Post-cholecystectomy
0.9479405034324943
2509 B103 P4 Idiopathic BAD Post-cholecystectomy
0.8993135011441648
2510 B103 P5 Idiopathic BAD Post-cholecystectomy
0.8430587337909993
2511 B103 P9 Idiopathic BAD Post-cholecystectomy
0.9174294431731502
2512 B103 P13 Idiopathic BAD Post-cholecystectomy
0.8272311212814645
2513 B103 P15 Idiopathic BAD Post-cholecystectomy
0.9170480549199085
2514 B103 P16 Idiopathic BAD Post-cholecystectomy
0.914187643020595
2515 B103 P17 Idiopathic BAD Post-cholecystectomy
0.9040808543096872
2516 B103 P20 Idiopathic BAD Post-cholecystectomy
0.8176964149504196
2517 B103 P21 Idiopathic BAD Post-cholecystectomy
0.8901601830663616
2518 B103 P24 Idiopathic BAD Post-cholecystectomy
0.9361174675819984
2519 B103 P26 Idiopathic BAD Post-cholecystectomy
0.7879481311975591
2520 B103 P30 Idiopathic BAD Post-cholecystectomy
0.9729214340198322
2521 B103 P33 Idiopathic BAD Post-cholecystectomy
0.9002669717772692
2522 B103 P35 Idiopathic BAD Post-cholecystectomy
0.8230358504958047
2523 B103 P38 Idiopathic BAD Post-cholecystectomy

0.9708237986270023
2524 B103 P39 Idiopathic BAD Post-cholecystectomy
0.8972158657513348
2525 B103 P42 Idiopathic BAD Post-cholecystectomy
0.7932875667429443
2526 B103 P43 Idiopathic BAD Post-cholecystectomy
0.8323798627002288
2527 B103 P46 Idiopathic BAD Post-cholecystectomy
0.8752860411899314
2528 B103 P47 Idiopathic BAD Post-cholecystectomy
0.9378337147215866
2529 B103 P50 Idiopathic BAD Post-cholecystectomy
0.9029366895499619
2530 B103 P55 Idiopathic BAD Post-cholecystectomy
0.9256292906178489
2531 B103 P58 Idiopathic BAD Post-cholecystectomy
0.8935926773455377
2532 B103 P60 Idiopathic BAD Post-cholecystectomy
0.9328756674294432
2533 B103 P63 Idiopathic BAD Post-cholecystectomy
0.8991228070175439
2534 B103 P65 Idiopathic BAD Post-cholecystectomy
0.8905415713196033
2535 B103 P68 Idiopathic BAD Post-cholecystectomy
0.8880625476735317
2536 B103 P70 Idiopathic BAD Post-cholecystectomy
0.9021739130434783
2537 B103 P71 Idiopathic BAD Post-cholecystectomy
0.7463768115942029
2538 B103 P74 Idiopathic BAD Post-cholecystectomy
0.8154080854309688
2539 B103 P75 Idiopathic BAD Post-cholecystectomy
0.910373760488177
2540 B106 P1 Idiopathic BAD Post-cholecystectomy
0.9740655987795576
2541 B106 P2 Idiopathic BAD Post-cholecystectomy
0.9225781845919145
2542 B106 P4 Idiopathic BAD Post-cholecystectomy
0.9439359267734554
2543 B106 P5 Idiopathic BAD Post-cholecystectomy
0.9153318077803204
2544 B106 P9 Idiopathic BAD Post-cholecystectomy
0.9624332570556827
2545 B106 P13 Idiopathic BAD Post-cholecystectomy
0.9420289855072463
2546 B106 P15 Idiopathic BAD Post-cholecystectomy
0.6763920671243325
2547 B106 P16 Idiopathic BAD Post-cholecystectomy
0.9469870327993898
2548 B106 P17 Idiopathic BAD Post-cholecystectomy
0.96186117467582
2549 B106 P20 Idiopathic BAD Post-cholecystectomy
0.9469870327993898
2550 B106 P21 Idiopathic BAD Post-cholecystectomy

0.9614797864225781
2551	B106	P24	Idiopathic BAD	Post-cholecystectomy
0.9391685736079328
2552	B106	P26	Idiopathic BAD	Post-cholecystectomy
0.9670099160945843
2553	B106	P30	Idiopathic BAD	Post-cholecystectomy
0.9546147978642258
2554	B106	P33	Idiopathic BAD	Post-cholecystectomy
0.9481311975591151
2555	B106	P35	Idiopathic BAD	Post-cholecystectomy
0.956140350877193
2556	B106	P38	Idiopathic BAD	Post-cholecystectomy
0.9824561403508771
2557	B106	P39	Idiopathic BAD	Post-cholecystectomy
0.933066361556064
2558	B106	P42	Idiopathic BAD	Post-cholecystectomy
0.9822654462242563
2559	B106	P43	Idiopathic BAD	Post-cholecystectomy
0.9445080091533181
2560	B106	P46	Idiopathic BAD	Post-cholecystectomy
0.8279938977879482
2561	B106	P47	Idiopathic BAD	Post-cholecystectomy
0.5276506483600305
2562	B106	P50	Idiopathic BAD	Post-cholecystectomy
0.9282990083905416
2563	B106	P55	Idiopathic BAD	Post-cholecystectomy
0.9254385964912281
2564	B106	P58	Idiopathic BAD	Post-cholecystectomy
0.9593821510297483
2565	B106	P60	Idiopathic BAD	Post-cholecystectomy
0.9811212814645309
2566	B106	P63	Idiopathic BAD	Post-cholecystectomy
0.9374523264683448
2567	B106	P65	Idiopathic BAD	Post-cholecystectomy
0.9437452326468345
2568	B106	P68	Idiopathic BAD	Post-cholecystectomy
0.9506102212051869
2569	B106	P70	Idiopathic BAD	Post-cholecystectomy
0.937070938215103
2570	B106	P71	Idiopathic BAD	Post-cholecystectomy
0.7520976353928299
2571	B106	P74	Idiopathic BAD	Post-cholecystectomy
0.9549961861174676
2572	B106	P75	Idiopathic BAD	Post-cholecystectomy
0.9281083142639207
2573	B109	P1	Idiopathic BAD	Post-cholecystectomy
0.9744469870327994
2574	B109	P2	Idiopathic BAD	Post-cholecystectomy
0.8114035087719298
2575	B109	P4	Idiopathic BAD	Post-cholecystectomy
0.8653699466056446
2576	B109	P5	Idiopathic BAD	Post-cholecystectomy
0.7444698703279939
2577	B109	P9	Idiopathic BAD	Post-cholecystectomy

0.790045766590389
2578   B109   P13   Idiopathic BAD Post-cholecystectomy
0.8878718535469108
2579   B109   P15   Idiopathic BAD Post-cholecystectomy
0.9057971014492754
2580   B109   P16   Idiopathic BAD Post-cholecystectomy
0.7139588100686499
2581   B109   P17   Idiopathic BAD Post-cholecystectomy
0.8251334858886347
2582   B109   P20   Idiopathic BAD Post-cholecystectomy
0.8787185354691075
2583   B109   P21   Idiopathic BAD Post-cholecystectomy
0.7570556826849733
2584   B109   P24   Idiopathic BAD Post-cholecystectomy
0.8771929824561403
2585   B109   P26   Idiopathic BAD Post-cholecystectomy
0.7036613272311213
2586   B109   P30   Idiopathic BAD Post-cholecystectomy
0.9427917620137299
2587   B109   P33   Idiopathic BAD Post-cholecystectomy
0.6521739130434783
2588   B109   P35   Idiopathic BAD Post-cholecystectomy
0.7046147978642258
2589   B109   P38   Idiopathic BAD Post-cholecystectomy
0.9784515636918383
2590   B109   P39   Idiopathic BAD Post-cholecystectomy
0.9202898550724637
2591   B109   P42   Idiopathic BAD Post-cholecystectomy
0.7972921434019832
2592   B109   P43   Idiopathic BAD Post-cholecystectomy
0.8325705568268498
2593   B109   P46   Idiopathic BAD Post-cholecystectomy
0.6624713958810069
2594   B109   P47   Idiopathic BAD Post-cholecystectomy
0.9738749046529367
2595   B109   P50   Idiopathic BAD Post-cholecystectomy
0.6598016781083142
2596   B109   P55   Idiopathic BAD Post-cholecystectomy
0.7522883295194508
2597   B109   P58   Idiopathic BAD Post-cholecystectomy
0.6912662090007627
2598   B109   P60   Idiopathic BAD Post-cholecystectomy
0.7814645308924485
2599   B109   P63   Idiopathic BAD Post-cholecystectomy
0.8047292143401983
2600   B109   P65   Idiopathic BAD Post-cholecystectomy
0.7244469870327994
2601   B109   P68   Idiopathic BAD Post-cholecystectomy
0.7801296720061022
2602   B109   P70   Idiopathic BAD Post-cholecystectomy
0.7438977879481312
2603   B109   P71   Idiopathic BAD Post-cholecystectomy
0.8676582761250954
2604   B109   P74   Idiopathic BAD Post-cholecystectomy

0.7534324942791762
2605 B109 P75 Idiopathic BAD Post-cholecystectomy
0.8293287566742944
2606 B118 P1 Idiopathic BAD Post-cholecystectomy
0.971395881006865
2607 B118 P2 Idiopathic BAD Post-cholecystectomy
0.8325705568268498
2608 B118 P4 Idiopathic BAD Post-cholecystectomy
0.8821510297482837
2609 B118 P5 Idiopathic BAD Post-cholecystectomy
0.8712814645308925
2610 B118 P9 Idiopathic BAD Post-cholecystectomy
0.9145690312738368
2611 B118 P13 Idiopathic BAD Post-cholecystectomy
0.8297101449275363
2612 B118 P15 Idiopathic BAD Post-cholecystectomy
0.7662090007627765
2613 B118 P16 Idiopathic BAD Post-cholecystectomy
0.940884820747521
2614 B118 P17 Idiopathic BAD Post-cholecystectomy
0.8716628527841342
2615 B118 P20 Idiopathic BAD Post-cholecystectomy
0.814836003051106
2616 B118 P21 Idiopathic BAD Post-cholecystectomy
0.8872997711670481
2617 B118 P24 Idiopathic BAD Post-cholecystectomy
0.9063691838291381
2618 B118 P26 Idiopathic BAD Post-cholecystectomy
0.8947368421052632
2619 B118 P30 Idiopathic BAD Post-cholecystectomy
0.9664378337147216
2620 B118 P33 Idiopathic BAD Post-cholecystectomy
0.8642257818459191
2621 B118 P35 Idiopathic BAD Post-cholecystectomy
0.9012204424103738
2622 B118 P38 Idiopathic BAD Post-cholecystectomy
0.9492753623188406
2623 B118 P39 Idiopathic BAD Post-cholecystectomy
0.9416475972540046
2624 B118 P42 Idiopathic BAD Post-cholecystectomy
0.9647215865751335
2625 B118 P43 Idiopathic BAD Post-cholecystectomy
0.8874904652936689
2626 B118 P46 Idiopathic BAD Post-cholecystectomy
0.7032799389778794
2627 B118 P47 Idiopathic BAD Post-cholecystectomy
0.6571319603356217
2628 B118 P50 Idiopathic BAD Post-cholecystectomy
0.791952707856598
2629 B118 P55 Idiopathic BAD Post-cholecystectomy
0.8634630053394355
2630 B118 P58 Idiopathic BAD Post-cholecystectomy
0.868421052631579
2631 B118 P60 Idiopathic BAD Post-cholecystectomy

0.9672006102212052
2632 B118 P63 Idiopathic BAD Post-cholecystectomy
0.8613653699466056
2633 B118 P65 Idiopathic BAD Post-cholecystectomy
0.8928299008390541
2634 B118 P68 Idiopathic BAD Post-cholecystectomy
0.9378337147215866
2635 B118 P70 Idiopathic BAD Post-cholecystectomy
0.8552631578947368
2636 B118 P71 Idiopathic BAD Post-cholecystectomy
0.7265446224256293
2637 B118 P74 Idiopathic BAD Post-cholecystectomy
0.8728070175438597
2638 B118 P75 Idiopathic BAD Post-cholecystectomy
0.8144546147978642
2639 B119 P1 Idiopathic BAD Post-cholecystectomy
0.9075133485888635
2640 B119 P2 Idiopathic BAD Post-cholecystectomy
0.8243707093821511
2641 B119 P4 Idiopathic BAD Post-cholecystectomy
0.7732646834477498
2642 B119 P5 Idiopathic BAD Post-cholecystectomy
0.6885964912280702
2643 B119 P9 Idiopathic BAD Post-cholecystectomy
0.6584668192219679
2644 B119 P13 Idiopathic BAD Post-cholecystectomy
0.7803203661327232
2645 B119 P15 Idiopathic BAD Post-cholecystectomy
0.8234172387490465
2646 B119 P16 Idiopathic BAD Post-cholecystectomy
0.7725019069412662
2647 B119 P17 Idiopathic BAD Post-cholecystectomy
0.7677345537757437
2648 B119 P20 Idiopathic BAD Post-cholecystectomy
0.7374141876430206
2649 B119 P21 Idiopathic BAD Post-cholecystectomy
0.662090007627765
2650 B119 P24 Idiopathic BAD Post-cholecystectomy
0.8382913806254767
2651 B119 P26 Idiopathic BAD Post-cholecystectomy
0.6571319603356217
2652 B119 P30 Idiopathic BAD Post-cholecystectomy
0.8800533943554538
2653 B119 P33 Idiopathic BAD Post-cholecystectomy
0.6678108314263921
2654 B119 P35 Idiopathic BAD Post-cholecystectomy
0.717391304347826
2655 B119 P38 Idiopathic BAD Post-cholecystectomy
0.9269641495041953
2656 B119 P39 Idiopathic BAD Post-cholecystectomy
0.7400839054157132
2657 B119 P42 Idiopathic BAD Post-cholecystectomy
0.7698321891685737
2658 B119 P43 Idiopathic BAD Post-cholecystectomy

0.7261632341723875
2659 B119 P46 Idiopathic BAD Post-cholecystectomy
0.7810831426392068
2660 B119 P47 Idiopathic BAD Post-cholecystectomy
0.9317315026697178
2661 B119 P50 Idiopathic BAD Post-cholecystectomy
0.7667810831426392
2662 B119 P55 Idiopathic BAD Post-cholecystectomy
0.6662852784134249
2663 B119 P58 Idiopathic BAD Post-cholecystectomy
0.7158657513348589
2664 B119 P60 Idiopathic BAD Post-cholecystectomy
0.8829138062547673
2665 B119 P63 Idiopathic BAD Post-cholecystectomy
0.7561022120518688
2666 B119 P65 Idiopathic BAD Post-cholecystectomy
0.7231121281464531
2667 B119 P68 Idiopathic BAD Post-cholecystectomy
0.6668573607932876
2668 B119 P70 Idiopathic BAD Post-cholecystectomy
0.6842105263157895
2669 B119 P71 Idiopathic BAD Post-cholecystectomy
0.8445842868039665
2670 B119 P74 Idiopathic BAD Post-cholecystectomy
0.7534324942791762
2671 B119 P75 Idiopathic BAD Post-cholecystectomy
0.7755530129672006
2672 B1 C1 Idiopathic BAD Post-op CD
0.830282227307399
2673 B1 C3 Idiopathic BAD Post-op CD
0.8155987795575896
2674 B1 C7 Idiopathic BAD Post-op CD
0.9414569031273837
2675 B1 C8 Idiopathic BAD Post-op CD
0.9084668192219679
2676 B1 C11 Idiopathic BAD Post-op CD
0.9231502669717773
2677 B1 C15 Idiopathic BAD Post-op CD
0.9050343249427918
2678 B1 C19 Idiopathic BAD Post-op CD
0.9269641495041953
2679 B1 C22 Idiopathic BAD Post-op CD
0.8607932875667429
2680 B1 C26 Idiopathic BAD Post-op CD
0.900839054157132
2681 B1 C28 Idiopathic BAD Post-op CD
0.9134248665141114
2682 B1 C31 Idiopathic BAD Post-op CD
0.9797864225781846
2683 B1 C35 Idiopathic BAD Post-op CD
0.9622425629290617
2684 B1 C38 Idiopathic BAD Post-op CD
0.9567124332570557
2685 B1 C40 Idiopathic BAD Post-op CD

0.8670861937452327
2686 B1 C44 Idiopathic BAD Post-op CD
0.8895881006864989
2687 B1 C47 Idiopathic BAD Post-op CD
0.931350114416476
2688 B1 C48 Idiopathic BAD Post-op CD
0.9324942791762014
2689 B1 C49 Idiopathic BAD Post-op CD
0.8724256292906178
2690 B1 C53 Idiopathic BAD Post-op CD
0.9521357742181541
2691 B1 C56 Idiopathic BAD Post-op CD
0.9078947368421053
2692 B1 C60 Idiopathic BAD Post-op CD
0.805301296720061
2693 B1 C62 Idiopathic BAD Post-op CD
0.9401220442410374
2694 B1 C64 Idiopathic BAD Post-op CD
0.9309687261632341
2695 B1 C65 Idiopathic BAD Post-op CD
0.858886346300534
2696 B1 C69 Idiopathic BAD Post-op CD
0.8115942028985508
2697 B1 C70 Idiopathic BAD Post-op CD
0.9317315026697178
2698 B1 C74 Idiopathic BAD Post-op CD
0.8506864988558352
2699 B1 C78 Idiopathic BAD Post-op CD
0.8640350877192983
2700 B5 C1 Idiopathic BAD Post-op CD
0.8716628527841342
2701 B5 C3 Idiopathic BAD Post-op CD
0.8996948893974066
2702 B5 C7 Idiopathic BAD Post-op CD
0.8785278413424866
2703 B5 C8 Idiopathic BAD Post-op CD
0.9429824561403509
2704 B5 C11 Idiopathic BAD Post-op CD
0.8638443935926774
2705 B5 C15 Idiopathic BAD Post-op CD
0.7692601067887109
2706 B5 C19 Idiopathic BAD Post-op CD
0.8680396643783371
2707 B5 C22 Idiopathic BAD Post-op CD
0.8832951945080092
2708 B5 C26 Idiopathic BAD Post-op CD
0.7179633867276888
2709 B5 C28 Idiopathic BAD Post-op CD
0.8655606407322655
2710 B5 C31 Idiopathic BAD Post-op CD
0.8571700991609459
2711 B5 C35 Idiopathic BAD Post-op CD
0.9086575133485889
2712 B5 C38 Idiopathic BAD Post-op CD

0.9551868802440885
2713 B5 C40 Idiopathic BAD Post-op CD
0.7816552250190694
2714 B5 C44 Idiopathic BAD Post-op CD
0.8728070175438597
2715 B5 C47 Idiopathic BAD Post-op CD
0.9052250190694127
2716 B5 C48 Idiopathic BAD Post-op CD
0.8632723112128147
2717 B5 C49 Idiopathic BAD Post-op CD
0.8640350877192983
2718 B5 C53 Idiopathic BAD Post-op CD
0.8956903127383676
2719 B5 C56 Idiopathic BAD Post-op CD
0.770976353928299
2720 B5 C60 Idiopathic BAD Post-op CD
0.8220823798627003
2721 B5 C62 Idiopathic BAD Post-op CD
0.8346681922196796
2722 B5 C64 Idiopathic BAD Post-op CD
0.8707093821510298
2723 B5 C65 Idiopathic BAD Post-op CD
0.8518306636155606
2724 B5 C69 Idiopathic BAD Post-op CD
0.7953852021357742
2725 B5 C70 Idiopathic BAD Post-op CD
0.8890160183066361
2726 B5 C74 Idiopathic BAD Post-op CD
0.8714721586575134
2727 B5 C78 Idiopathic BAD Post-op CD
0.7822273073989321
2728 B6 C1 Idiopathic BAD Post-op CD
0.9860793287566743
2729 B6 C3 Idiopathic BAD Post-op CD
0.9212433257055682
2730 B6 C7 Idiopathic BAD Post-op CD
0.9603356216628528
2731 B6 C8 Idiopathic BAD Post-op CD
0.8878718535469108
2732 B6 C11 Idiopathic BAD Post-op CD
0.954233409610984
2733 B6 C15 Idiopathic BAD Post-op CD
0.9241037376048817
2734 B6 C19 Idiopathic BAD Post-op CD
0.9145690312738368
2735 B6 C22 Idiopathic BAD Post-op CD
0.9576659038901602
2736 B6 C26 Idiopathic BAD Post-op CD
0.9155225019069413
2737 B6 C28 Idiopathic BAD Post-op CD
0.8975972540045767
2738 B6 C31 Idiopathic BAD Post-op CD
0.9622425629290617
2739 B6 C35 Idiopathic BAD Post-op CD

0.9071319603356217
2740	B6	C38	Idiopathic BAD	Post-op CD
0.9862700228832952
2741	B6	C40	Idiopathic BAD	Post-op CD
0.9029366895499619
2742	B6	C44	Idiopathic BAD	Post-op CD
0.9773073989321129
2743	B6	C47	Idiopathic BAD	Post-op CD
0.979023646071701
2744	B6	C48	Idiopathic BAD	Post-op CD
0.9269641495041953
2745	B6	C49	Idiopathic BAD	Post-op CD
0.9183829138062548
2746	B6	C53	Idiopathic BAD	Post-op CD
0.9321128909229596
2747	B6	C56	Idiopathic BAD	Post-op CD
0.8819603356216629
2748	B6	C60	Idiopathic BAD	Post-op CD
0.908276125095347
2749	B6	C62	Idiopathic BAD	Post-op CD
0.9162852784134249
2750	B6	C64	Idiopathic BAD	Post-op CD
0.9218154080854309
2751	B6	C65	Idiopathic BAD	Post-op CD
0.9345919145690312
2752	B6	C69	Idiopathic BAD	Post-op CD
0.9069412662090007
2753	B6	C70	Idiopathic BAD	Post-op CD
0.8996948893974066
2754	B6	C74	Idiopathic BAD	Post-op CD
0.9347826086956522
2755	B6	C78	Idiopathic BAD	Post-op CD
0.9052250190694127
2756	B10	C1	Idiopathic BAD	Post-op CD
0.8096872616323417
2757	B10	C3	Idiopathic BAD	Post-op CD
0.8173150266971777
2758	B10	C7	Idiopathic BAD	Post-op CD
0.9906559877955758
2759	B10	C8	Idiopathic BAD	Post-op CD
0.8996948893974066
2760	B10	C11	Idiopathic BAD	Post-op CD
0.9950419527078566
2761	B10	C15	Idiopathic BAD	Post-op CD
0.977116704805492
2762	B10	C19	Idiopathic BAD	Post-op CD
0.9788329519450801
2763	B10	C22	Idiopathic BAD	Post-op CD
0.6836384439359268
2764	B10	C26	Idiopathic BAD	Post-op CD
0.9893211289092296
2765	B10	C28	Idiopathic BAD	Post-op CD
0.9799771167048055
2766	B10	C31	Idiopathic BAD	Post-op CD

0.9836003051106026
2767 B10 C35 Idiopathic BAD Post-op CD
0.9918001525553013
2768 B10 C38 Idiopathic BAD Post-op CD
0.9475591151792525
2769 B10 C40 Idiopathic BAD Post-op CD
0.9530892448512586
2770 B10 C44 Idiopathic BAD Post-op CD
0.9834096109839817
2771 B10 C47 Idiopathic BAD Post-op CD
0.9889397406559878
2772 B10 C48 Idiopathic BAD Post-op CD
0.92372234935164
2773 B10 C49 Idiopathic BAD Post-op CD
0.9303966437833715
2774 B10 C53 Idiopathic BAD Post-op CD
0.9546147978642258
2775 B10 C56 Idiopathic BAD Post-op CD
0.9204805491990846
2776 B10 C60 Idiopathic BAD Post-op CD
0.7927154843630816
2777 B10 C62 Idiopathic BAD Post-op CD
0.9429824561403509
2778 B10 C64 Idiopathic BAD Post-op CD
0.8779557589626239
2779 B10 C65 Idiopathic BAD Post-op CD
0.753813882532418
2780 B10 C69 Idiopathic BAD Post-op CD
0.9475591151792525
2781 B10 C70 Idiopathic BAD Post-op CD
0.952326468344775
2782 B10 C74 Idiopathic BAD Post-op CD
0.8012967200610221
2783 B10 C78 Idiopathic BAD Post-op CD
0.9672006102212052
2784 B17 C1 Idiopathic BAD Post-op CD
0.9525171624713958
2785 B17 C3 Idiopathic BAD Post-op CD
0.9233409610983981
2786 B17 C7 Idiopathic BAD Post-op CD
0.6624713958810069
2787 B17 C8 Idiopathic BAD Post-op CD
0.9252479023646072
2788 B17 C11 Idiopathic BAD Post-op CD
0.8890160183066361
2789 B17 C15 Idiopathic BAD Post-op CD
0.9239130434782609
2790 B17 C19 Idiopathic BAD Post-op CD
0.6289092295957285
2791 B17 C22 Idiopathic BAD Post-op CD
0.9130434782608695
2792 B17 C26 Idiopathic BAD Post-op CD
0.938977879481312
2793 B17 C28 Idiopathic BAD Post-op CD

0.9094202898550725
2794	B17	C31	Idiopathic BAD	Post-op CD
0.9681540808543097
2795	B17	C35	Idiopathic BAD	Post-op CD
0.8030129672006102
2796	B17	C38	Idiopathic BAD	Post-op CD
0.9786422578184591
2797	B17	C40	Idiopathic BAD	Post-op CD
0.9473684210526315
2798	B17	C44	Idiopathic BAD	Post-op CD
0.9021739130434783
2799	B17	C47	Idiopathic BAD	Post-op CD
0.910373760488177
2800	B17	C48	Idiopathic BAD	Post-op CD
0.700419527078566
2801	B17	C49	Idiopathic BAD	Post-op CD
0.8548817696414951
2802	B17	C53	Idiopathic BAD	Post-op CD
0.7448512585812357
2803	B17	C56	Idiopathic BAD	Post-op CD
0.7496186117467581
2804	B17	C60	Idiopathic BAD	Post-op CD
0.7433257055682685
2805	B17	C62	Idiopathic BAD	Post-op CD
0.831998474446987
2806	B17	C64	Idiopathic BAD	Post-op CD
0.7683066361556065
2807	B17	C65	Idiopathic BAD	Post-op CD
0.7984363081617086
2808	B17	C69	Idiopathic BAD	Post-op CD
0.7658276125095347
2809	B17	C70	Idiopathic BAD	Post-op CD
0.8135011441647597
2810	B17	C74	Idiopathic BAD	Post-op CD
0.8468726163234173
2811	B17	C78	Idiopathic BAD	Post-op CD
0.8668954996186118
2812	B20	C1	Idiopathic BAD	Post-op CD
0.9759725400457666
2813	B20	C3	Idiopathic BAD	Post-op CD
0.9456521739130435
2814	B20	C7	Idiopathic BAD	Post-op CD
0.5922959572845157
2815	B20	C8	Idiopathic BAD	Post-op CD
0.9740655987795576
2816	B20	C11	Idiopathic BAD	Post-op CD
0.9229595728451564
2817	B20	C15	Idiopathic BAD	Post-op CD
0.9023646071700991
2818	B20	C19	Idiopathic BAD	Post-op CD
0.7091914569031273
2819	B20	C22	Idiopathic BAD	Post-op CD
0.9433638443935927
2820	B20	C26	Idiopathic BAD	Post-op CD

0.9662471395881007
2821 B20 C28 Idiopathic BAD Post-op CD
0.9406941266209001
2822 B20 C31 Idiopathic BAD Post-op CD
0.9853165522501907
2823 B20 C35 Idiopathic BAD Post-op CD
0.721205186880244
2824 B20 C38 Idiopathic BAD Post-op CD
0.9774980930587338
2825 B20 C40 Idiopathic BAD Post-op CD
0.9364988558352403
2826 B20 C44 Idiopathic BAD Post-op CD
0.9218154080854309
2827 B20 C47 Idiopathic BAD Post-op CD
0.9342105263157895
2828 B20 C48 Idiopathic BAD Post-op CD
0.6338672768878718
2829 B20 C49 Idiopathic BAD Post-op CD
0.8415331807780321
2830 B20 C53 Idiopathic BAD Post-op CD
0.7776506483600305
2831 B20 C56 Idiopathic BAD Post-op CD
0.7358886346300534
2832 B20 C60 Idiopathic BAD Post-op CD
0.7848970251716247
2833 B20 C62 Idiopathic BAD Post-op CD
0.8068268497330282
2834 B20 C64 Idiopathic BAD Post-op CD
0.7908085430968727
2835 B20 C65 Idiopathic BAD Post-op CD
0.7765064836003052
2836 B20 C69 Idiopathic BAD Post-op CD
0.7721205186880244
2837 B20 C70 Idiopathic BAD Post-op CD
0.7925247902364607
2838 B20 C74 Idiopathic BAD Post-op CD
0.8211289092295957
2839 B20 C78 Idiopathic BAD Post-op CD
0.8565980167810832
2840 B23 C1 Idiopathic BAD Post-op CD
0.9454614797864226
2841 B23 C3 Idiopathic BAD Post-op CD
0.8503051106025934
2842 B23 C7 Idiopathic BAD Post-op CD
0.8758581235697941
2843 B23 C8 Idiopathic BAD Post-op CD
0.8827231121281465
2844 B23 C11 Idiopathic BAD Post-op CD
0.7210144927536232
2845 B23 C15 Idiopathic BAD Post-op CD
0.7456140350877193
2846 B23 C19 Idiopathic BAD Post-op CD
0.719488939740656
2847 B23 C22 Idiopathic BAD Post-op CD

0.9454614797864226
2848	B23	C26	Idiopathic BAD	Post-op CD
0.8714721586575134
2849	B23	C28	Idiopathic BAD	Post-op CD
0.7540045766590389
2850	B23	C31	Idiopathic BAD	Post-op CD
0.9422196796338673
2851	B23	C35	Idiopathic BAD	Post-op CD
0.7334096109839817
2852	B23	C38	Idiopathic BAD	Post-op CD
0.9782608695652174
2853	B23	C40	Idiopathic BAD	Post-op CD
0.9549961861174676
2854	B23	C44	Idiopathic BAD	Post-op CD
0.7679252479023646
2855	B23	C47	Idiopathic BAD	Post-op CD
0.7879481311975591
2856	B23	C48	Idiopathic BAD	Post-op CD
0.8447749809305873
2857	B23	C49	Idiopathic BAD	Post-op CD
0.935163996948894
2858	B23	C53	Idiopathic BAD	Post-op CD
0.7782227307398932
2859	B23	C56	Idiopathic BAD	Post-op CD
0.7307398932112891
2860	B23	C60	Idiopathic BAD	Post-op CD
0.7326468344774981
2861	B23	C62	Idiopathic BAD	Post-op CD
0.9061784897025171
2862	B23	C64	Idiopathic BAD	Post-op CD
0.833905415713196
2863	B23	C65	Idiopathic BAD	Post-op CD
0.9138062547673532
2864	B23	C69	Idiopathic BAD	Post-op CD
0.8731884057971014
2865	B23	C70	Idiopathic BAD	Post-op CD
0.9250572082379863
2866	B23	C74	Idiopathic BAD	Post-op CD
0.933257055682685
2867	B23	C78	Idiopathic BAD	Post-op CD
0.7568649885583524
2868	B31	C1	Idiopathic BAD	Post-op CD
0.7240655987795576
2869	B31	C3	Idiopathic BAD	Post-op CD
0.8144546147978642
2870	B31	C7	Idiopathic BAD	Post-op CD
0.950419527078566
2871	B31	C8	Idiopathic BAD	Post-op CD
0.9052250190694127
2872	B31	C11	Idiopathic BAD	Post-op CD
0.8838672768878718
2873	B31	C15	Idiopathic BAD	Post-op CD
0.952326468344775
2874	B31	C19	Idiopathic BAD	Post-op CD

0.912090007627765
2875 B31 C22 Idiopathic BAD Post-op CD
0.8285659801678108
2876 B31 C26 Idiopathic BAD Post-op CD
0.9258199847444699
2877 B31 C28 Idiopathic BAD Post-op CD
0.9525171624713958
2878 B31 C31 Idiopathic BAD Post-op CD
0.9652936689549961
2879 B31 C35 Idiopathic BAD Post-op CD
0.9528985507246377
2880 B31 C38 Idiopathic BAD Post-op CD
0.9570938215102975
2881 B31 C40 Idiopathic BAD Post-op CD
0.9242944317315027
2882 B31 C44 Idiopathic BAD Post-op CD
0.9418382913806255
2883 B31 C47 Idiopathic BAD Post-op CD
0.9929443173150267
2884 B31 C48 Idiopathic BAD Post-op CD
0.9105644546147978
2885 B31 C49 Idiopathic BAD Post-op CD
0.9403127383676583
2886 B31 C53 Idiopathic BAD Post-op CD
0.9517543859649122
2887 B31 C56 Idiopathic BAD Post-op CD
0.9042715484363082
2888 B31 C60 Idiopathic BAD Post-op CD
0.8005339435545386
2889 B31 C62 Idiopathic BAD Post-op CD
0.9563310450038138
2890 B31 C64 Idiopathic BAD Post-op CD
0.90255530129672
2891 B31 C65 Idiopathic BAD Post-op CD
0.8571700991609459
2892 B31 C69 Idiopathic BAD Post-op CD
0.9143783371472158
2893 B31 C70 Idiopathic BAD Post-op CD
0.9609077040427155
2894 B31 C74 Idiopathic BAD Post-op CD
0.8737604881769642
2895 B31 C78 Idiopathic BAD Post-op CD
0.9057971014492754
2896 B35 C1 Idiopathic BAD Post-op CD
0.7738367658276125
2897 B35 C3 Idiopathic BAD Post-op CD
0.9761632341723875
2898 B35 C7 Idiopathic BAD Post-op CD
0.9630053394355453
2899 B35 C8 Idiopathic BAD Post-op CD
0.9557589626239512
2900 B35 C11 Idiopathic BAD Post-op CD
0.8525934401220442
2901 B35 C15 Idiopathic BAD Post-op CD

0.8573607932875668
2902	B35	C19	Idiopathic BAD	Post-op CD
0.9765446224256293
2903	B35	C22	Idiopathic BAD	Post-op CD
0.9738749046529367
2904	B35	C26	Idiopathic BAD	Post-op CD
0.6992753623188406
2905	B35	C28	Idiopathic BAD	Post-op CD
0.8848207475209764
2906	B35	C31	Idiopathic BAD	Post-op CD
0.8461098398169337
2907	B35	C35	Idiopathic BAD	Post-op CD
0.9769260106788711
2908	B35	C38	Idiopathic BAD	Post-op CD
0.9380244088482075
2909	B35	C40	Idiopathic BAD	Post-op CD
0.6332951945080092
2910	B35	C44	Idiopathic BAD	Post-op CD
0.950228832951945
2911	B35	C47	Idiopathic BAD	Post-op CD
0.9406941266209001
2912	B35	C48	Idiopathic BAD	Post-op CD
0.9822654462242563
2913	B35	C49	Idiopathic BAD	Post-op CD
0.7440884820747521
2914	B35	C53	Idiopathic BAD	Post-op CD
0.9363081617086194
2915	B35	C56	Idiopathic BAD	Post-op CD
0.9525171624713958
2916	B35	C60	Idiopathic BAD	Post-op CD
0.9170480549199085
2917	B35	C62	Idiopathic BAD	Post-op CD
0.9265827612509535
2918	B35	C64	Idiopathic BAD	Post-op CD
0.9208619374523265
2919	B35	C65	Idiopathic BAD	Post-op CD
0.9643401983218917
2920	B35	C69	Idiopathic BAD	Post-op CD
0.8554538520213577
2921	B35	C70	Idiopathic BAD	Post-op CD
0.7902364607170099
2922	B35	C74	Idiopathic BAD	Post-op CD
0.9935163996948894
2923	B35	C78	Idiopathic BAD	Post-op CD
0.839626239511823
2924	B39	C1	Idiopathic BAD	Post-op CD
0.8838672768878718
2925	B39	C3	Idiopathic BAD	Post-op CD
0.833905415713196
2926	B39	C7	Idiopathic BAD	Post-op CD
0.9443173150266971
2927	B39	C8	Idiopathic BAD	Post-op CD
0.8836765827612509
2928	B39	C11	Idiopathic BAD	Post-op CD

0.9050343249427918
2929 B39 C15 Idiopathic BAD Post-op CD
0.9016018306636155
2930 B39 C19 Idiopathic BAD Post-op CD
0.8937833714721587
2931 B39 C22 Idiopathic BAD Post-op CD
0.9012204424103738
2932 B39 C26 Idiopathic BAD Post-op CD
0.9023646071700991
2933 B39 C28 Idiopathic BAD Post-op CD
0.8888253241800153
2934 B39 C31 Idiopathic BAD Post-op CD
0.9191456903127384
2935 B39 C35 Idiopathic BAD Post-op CD
0.9311594202898551
2936 B39 C38 Idiopathic BAD Post-op CD
0.9528985507246377
2937 B39 C40 Idiopathic BAD Post-op CD
0.9023646071700991
2938 B39 C44 Idiopathic BAD Post-op CD
0.9439359267734554
2939 B39 C47 Idiopathic BAD Post-op CD
0.9271548436308161
2940 B39 C48 Idiopathic BAD Post-op CD
0.8968344774980931
2941 B39 C49 Idiopathic BAD Post-op CD
0.9088482074752098
2942 B39 C53 Idiopathic BAD Post-op CD
0.9328756674294432
2943 B39 C56 Idiopathic BAD Post-op CD
0.864607170099161
2944 B39 C60 Idiopathic BAD Post-op CD
0.8686117467581999
2945 B39 C62 Idiopathic BAD Post-op CD
0.8770022883295194
2946 B39 C64 Idiopathic BAD Post-op CD
0.9077040427154843
2947 B39 C65 Idiopathic BAD Post-op CD
0.9231502669717773
2948 B39 C69 Idiopathic BAD Post-op CD
0.9269641495041953
2949 B39 C70 Idiopathic BAD Post-op CD
0.9246758199847445
2950 B39 C74 Idiopathic BAD Post-op CD
0.9214340198321892
2951 B39 C78 Idiopathic BAD Post-op CD
0.8802440884820748
2952 B43 C1 Idiopathic BAD Post-op CD
0.984744469870328
2953 B43 C3 Idiopathic BAD Post-op CD
0.9467963386727689
2954 B43 C7 Idiopathic BAD Post-op CD
0.748093058733791
2955 B43 C8 Idiopathic BAD Post-op CD

0.9759725400457666
2956 B43 C11 Idiopathic BAD Post-op CD
0.9668192219679634
2957 B43 C15 Idiopathic BAD Post-op CD
0.9626239511823036
2958 B43 C19 Idiopathic BAD Post-op CD
0.8276125095347063
2959 B43 C22 Idiopathic BAD Post-op CD
0.9578565980167811
2960 B43 C26 Idiopathic BAD Post-op CD
0.975209763539283
2961 B43 C28 Idiopathic BAD Post-op CD
0.9221967963386728
2962 B43 C31 Idiopathic BAD Post-op CD
0.9834096109839817
2963 B43 C35 Idiopathic BAD Post-op CD
0.7656369183829138
2964 B43 C38 Idiopathic BAD Post-op CD
0.9876048817696415
2965 B43 C40 Idiopathic BAD Post-op CD
0.9231502669717773
2966 B43 C44 Idiopathic BAD Post-op CD
0.9809305873379099
2967 B43 C47 Idiopathic BAD Post-op CD
0.9546147978642258
2968 B43 C48 Idiopathic BAD Post-op CD
0.6956521739130435
2969 B43 C49 Idiopathic BAD Post-op CD
0.820747520976354
2970 B43 C53 Idiopathic BAD Post-op CD
0.7965293668954996
2971 B43 C56 Idiopathic BAD Post-op CD
0.7549580472921434
2972 B43 C60 Idiopathic BAD Post-op CD
0.8049199084668193
2973 B43 C62 Idiopathic BAD Post-op CD
0.8102593440122045
2974 B43 C64 Idiopathic BAD Post-op CD
0.7847063310450039
2975 B43 C65 Idiopathic BAD Post-op CD
0.7833714721586575
2976 B43 C69 Idiopathic BAD Post-op CD
0.7822273073989321
2977 B43 C70 Idiopathic BAD Post-op CD
0.7848970251716247
2978 B43 C74 Idiopathic BAD Post-op CD
0.8199847444698704
2979 B43 C78 Idiopathic BAD Post-op CD
0.9134248665141114
2980 B47 C1 Idiopathic BAD Post-op CD
0.9481311975591151
2981 B47 C3 Idiopathic BAD Post-op CD
0.9340198321891686
2982 B47 C7 Idiopathic BAD Post-op CD

0.8918764302059496
2983 B47 C8 Idiopathic BAD Post-op CD
0.9414569031273837
2984 B47 C11 Idiopathic BAD Post-op CD
0.8918764302059496
2985 B47 C15 Idiopathic BAD Post-op CD
0.8056826849733029
2986 B47 C19 Idiopathic BAD Post-op CD
0.8556445461479787
2987 B47 C22 Idiopathic BAD Post-op CD
0.9467963386727689
2988 B47 C26 Idiopathic BAD Post-op CD
0.9050343249427918
2989 B47 C28 Idiopathic BAD Post-op CD
0.8630816170861938
2990 B47 C31 Idiopathic BAD Post-op CD
0.9536613272311213
2991 B47 C35 Idiopathic BAD Post-op CD
0.8716628527841342
2992 B47 C38 Idiopathic BAD Post-op CD
0.9692982456140351
2993 B47 C40 Idiopathic BAD Post-op CD
0.9315408085430968
2994 B47 C44 Idiopathic BAD Post-op CD
0.8869183829138062
2995 B47 C47 Idiopathic BAD Post-op CD
0.8663234172387491
2996 B47 C48 Idiopathic BAD Post-op CD
0.8360030511060259
2997 B47 C49 Idiopathic BAD Post-op CD
0.9174294431731502
2998 B47 C53 Idiopathic BAD Post-op CD
0.84744469870328
2999 B47 C56 Idiopathic BAD Post-op CD
0.8011060259344012
3000 B47 C60 Idiopathic BAD Post-op CD
0.7950038138825324
3001 B47 C62 Idiopathic BAD Post-op CD
0.8972158657513348
3002 B47 C64 Idiopathic BAD Post-op CD
0.879862700228833
3003 B47 C65 Idiopathic BAD Post-op CD
0.8874904652936689
3004 B47 C69 Idiopathic BAD Post-op CD
0.8647978642257819
3005 B47 C70 Idiopathic BAD Post-op CD
0.8958810068649885
3006 B47 C74 Idiopathic BAD Post-op CD
0.9084668192219679
3007 B47 C78 Idiopathic BAD Post-op CD
0.8072082379862701
3008 B48 C1 Idiopathic BAD Post-op CD
0.8283752860411899
3009 B48 C3 Idiopathic BAD Post-op CD

0.971395881006865
3010 B48 C7 Idiopathic BAD Post-op CD
0.9464149504195271
3011 B48 C8 Idiopathic BAD Post-op CD
0.9464149504195271
3012 B48 C11 Idiopathic BAD Post-op CD
0.9016018306636155
3013 B48 C15 Idiopathic BAD Post-op CD
0.8138825324180016
3014 B48 C19 Idiopathic BAD Post-op CD
0.9862700228832952
3015 B48 C22 Idiopathic BAD Post-op CD
0.9628146453089245
3016 B48 C26 Idiopathic BAD Post-op CD
0.7301678108314263
3017 B48 C28 Idiopathic BAD Post-op CD
0.9122807017543859
3018 B48 C31 Idiopathic BAD Post-op CD
0.8146453089244852
3019 B48 C35 Idiopathic BAD Post-op CD
0.9683447749809306
3020 B48 C38 Idiopathic BAD Post-op CD
0.8199847444698704
3021 B48 C40 Idiopathic BAD Post-op CD
0.669717772692601
3022 B48 C44 Idiopathic BAD Post-op CD
0.937070938215103
3023 B48 C47 Idiopathic BAD Post-op CD
0.9832189168573608
3024 B48 C48 Idiopathic BAD Post-op CD
0.9750190694126621
3025 B48 C49 Idiopathic BAD Post-op CD
0.7913806254767353
3026 B48 C53 Idiopathic BAD Post-op CD
0.9757818459191457
3027 B48 C56 Idiopathic BAD Post-op CD
0.8775743707093822
3028 B48 C60 Idiopathic BAD Post-op CD
0.8981693363844394
3029 B48 C62 Idiopathic BAD Post-op CD
0.8872997711670481
3030 B48 C64 Idiopathic BAD Post-op CD
0.9263920671243325
3031 B48 C65 Idiopathic BAD Post-op CD
0.9479405034324943
3032 B48 C69 Idiopathic BAD Post-op CD
0.8241800152555301
3033 B48 C70 Idiopathic BAD Post-op CD
0.9326849733028223
3034 B48 C74 Idiopathic BAD Post-op CD
0.9723493516399695
3035 B48 C78 Idiopathic BAD Post-op CD
0.7980549199084668
3036 B49 C1 Idiopathic BAD Post-op CD

0.9584286803966438
3037	B49	C3	Idiopathic BAD	Post-op CD
0.935163996948894
3038	B49	C7	Idiopathic BAD	Post-op CD
0.9815026697177727
3039	B49	C8	Idiopathic BAD	Post-op CD
0.965675057208238
3040	B49	C11	Idiopathic BAD	Post-op CD
0.915903890160183
3041	B49	C15	Idiopathic BAD	Post-op CD
0.8922578184591915
3042	B49	C19	Idiopathic BAD	Post-op CD
0.9376430205949656
3043	B49	C22	Idiopathic BAD	Post-op CD
0.96186117467582
3044	B49	C26	Idiopathic BAD	Post-op CD
0.8352402745995423
3045	B49	C28	Idiopathic BAD	Post-op CD
0.9307780320366132
3046	B49	C31	Idiopathic BAD	Post-op CD
0.8266590389016019
3047	B49	C35	Idiopathic BAD	Post-op CD
0.9185736079328757
3048	B49	C38	Idiopathic BAD	Post-op CD
0.9803585049580473
3049	B49	C40	Idiopathic BAD	Post-op CD
0.8480167810831426
3050	B49	C44	Idiopathic BAD	Post-op CD
0.9626239511823036
3051	B49	C47	Idiopathic BAD	Post-op CD
0.9740655987795576
3052	B49	C48	Idiopathic BAD	Post-op CD
0.8831045003813882
3053	B49	C49	Idiopathic BAD	Post-op CD
0.7942410373760488
3054	B49	C53	Idiopathic BAD	Post-op CD
0.8909229595728452
3055	B49	C56	Idiopathic BAD	Post-op CD
0.8714721586575134
3056	B49	C60	Idiopathic BAD	Post-op CD
0.9092295957284515
3057	B49	C62	Idiopathic BAD	Post-op CD
0.8749046529366895
3058	B49	C64	Idiopathic BAD	Post-op CD
0.8859649122807017
3059	B49	C65	Idiopathic BAD	Post-op CD
0.8947368421052632
3060	B49	C69	Idiopathic BAD	Post-op CD
0.7793668954996186
3061	B49	C70	Idiopathic BAD	Post-op CD
0.885392829900839
3062	B49	C74	Idiopathic BAD	Post-op CD
0.9223874904652937
3063	B49	C78	Idiopathic BAD	Post-op CD

0.765255530129672
3064 B53 C1 Idiopathic BAD Post-op CD
0.9439359267734554
3065 B53 C3 Idiopathic BAD Post-op CD
0.9155225019069413
3066 B53 C7 Idiopathic BAD Post-op CD
0.8947368421052632
3067 B53 C8 Idiopathic BAD Post-op CD
0.9719679633867276
3068 B53 C11 Idiopathic BAD Post-op CD
0.795766590389016
3069 B53 C15 Idiopathic BAD Post-op CD
0.8028222730739893
3070 B53 C19 Idiopathic BAD Post-op CD
0.7627765064836003
3071 B53 C22 Idiopathic BAD Post-op CD
0.96186117467582
3072 B53 C26 Idiopathic BAD Post-op CD
0.9551868802440885
3073 B53 C28 Idiopathic BAD Post-op CD
0.7185354691075515
3074 B53 C31 Idiopathic BAD Post-op CD
0.969488939740656
3075 B53 C35 Idiopathic BAD Post-op CD
0.7984363081617086
3076 B53 C38 Idiopathic BAD Post-op CD
0.9620518688024409
3077 B53 C40 Idiopathic BAD Post-op CD
0.9288710907704043
3078 B53 C44 Idiopathic BAD Post-op CD
0.7782227307398932
3079 B53 C47 Idiopathic BAD Post-op CD
0.7669717772692601
3080 B53 C48 Idiopathic BAD Post-op CD
0.8617467581998475
3081 B53 C49 Idiopathic BAD Post-op CD
0.9113272311212814
3082 B53 C53 Idiopathic BAD Post-op CD
0.7721205186880244
3083 B53 C56 Idiopathic BAD Post-op CD
0.7398932112890922
3084 B53 C60 Idiopathic BAD Post-op CD
0.6966056445461479
3085 B53 C62 Idiopathic BAD Post-op CD
0.9174294431731502
3086 B53 C64 Idiopathic BAD Post-op CD
0.8520213577421816
3087 B53 C65 Idiopathic BAD Post-op CD
0.8991228070175439
3088 B53 C69 Idiopathic BAD Post-op CD
0.8371472158657514
3089 B53 C70 Idiopathic BAD Post-op CD
0.9038901601830663
3090 B53 C74 Idiopathic BAD Post-op CD

0.9262013729977117
3091	B53	C78	Idiopathic BAD	Post-op CD
0.8560259344012204
3092	B54	C1	Idiopathic BAD	Post-op CD
0.967391304347826
3093	B54	C3	Idiopathic BAD	Post-op CD
0.9565217391304348
3094	B54	C7	Idiopathic BAD	Post-op CD
0.6437833714721587
3095	B54	C8	Idiopathic BAD	Post-op CD
0.9778794813119756
3096	B54	C11	Idiopathic BAD	Post-op CD
0.9355453852021358
3097	B54	C15	Idiopathic BAD	Post-op CD
0.9122807017543859
3098	B54	C19	Idiopathic BAD	Post-op CD
0.5606407322654462
3099	B54	C22	Idiopathic BAD	Post-op CD
0.9729214340198322
3100	B54	C26	Idiopathic BAD	Post-op CD
0.9300152555301296
3101	B54	C28	Idiopathic BAD	Post-op CD
0.9212433257055682
3102	B54	C31	Idiopathic BAD	Post-op CD
0.9185736079328757
3103	B54	C35	Idiopathic BAD	Post-op CD
0.8394355453852022
3104	B54	C38	Idiopathic BAD	Post-op CD
0.982837528604119
3105	B54	C40	Idiopathic BAD	Post-op CD
0.9685354691075515
3106	B54	C44	Idiopathic BAD	Post-op CD
0.9063691838291381
3107	B54	C47	Idiopathic BAD	Post-op CD
0.9374523264683448
3108	B54	C48	Idiopathic BAD	Post-op CD
0.6969870327993898
3109	B54	C49	Idiopathic BAD	Post-op CD
0.864607170099161
3110	B54	C53	Idiopathic BAD	Post-op CD
0.7757437070938215
3111	B54	C56	Idiopathic BAD	Post-op CD
0.7753623188405797
3112	B54	C60	Idiopathic BAD	Post-op CD
0.7831807780320366
3113	B54	C62	Idiopathic BAD	Post-op CD
0.8451563691838292
3114	B54	C64	Idiopathic BAD	Post-op CD
0.7889016018306636
3115	B54	C65	Idiopathic BAD	Post-op CD
0.8133104500381388
3116	B54	C69	Idiopathic BAD	Post-op CD
0.7807017543859649
3117	B54	C70	Idiopathic BAD	Post-op CD

0.8201754385964912
3118 B54 C74 Idiopathic BAD Post-op CD
0.8493516399694889
3119 B54 C78 Idiopathic BAD Post-op CD
0.8916857360793288
3120 B55 C1 Idiopathic BAD Post-op CD
0.9938977879481312
3121 B55 C3 Idiopathic BAD Post-op CD
0.9532799389778794
3122 B55 C7 Idiopathic BAD Post-op CD
0.5671243325705568
3123 B55 C8 Idiopathic BAD Post-op CD
0.9645308924485125
3124 B55 C11 Idiopathic BAD Post-op CD
0.8581235697940504
3125 B55 C15 Idiopathic BAD Post-op CD
0.8899694889397407
3126 B55 C19 Idiopathic BAD Post-op CD
0.4744469870327994
3127 B55 C22 Idiopathic BAD Post-op CD
0.973302822273074
3128 B55 C26 Idiopathic BAD Post-op CD
0.9616704805491991
3129 B55 C28 Idiopathic BAD Post-op CD
0.8972158657513348
3130 B55 C31 Idiopathic BAD Post-op CD
0.9959954233409611
3131 B55 C35 Idiopathic BAD Post-op CD
0.8222730739893211
3132 B55 C38 Idiopathic BAD Post-op CD
0.9938977879481312
3133 B55 C40 Idiopathic BAD Post-op CD
0.9879862700228833
3134 B55 C44 Idiopathic BAD Post-op CD
0.9021739130434783
3135 B55 C47 Idiopathic BAD Post-op CD
0.8859649122807017
3136 B55 C48 Idiopathic BAD Post-op CD
0.7248283752860412
3137 B55 C49 Idiopathic BAD Post-op CD
0.8657513348588863
3138 B55 C53 Idiopathic BAD Post-op CD
0.7494279176201373
3139 B55 C56 Idiopathic BAD Post-op CD
0.7336003051106026
3140 B55 C60 Idiopathic BAD Post-op CD
0.7583905415713196
3141 B55 C62 Idiopathic BAD Post-op CD
0.8482074752097636
3142 B55 C64 Idiopathic BAD Post-op CD
0.8312356979405034
3143 B55 C65 Idiopathic BAD Post-op CD
0.8205568268497331
3144 B55 C69 Idiopathic BAD Post-op CD

0.8213196033562167
3145 B55 C70 Idiopathic BAD Post-op CD
0.8161708619374524
3146 B55 C74 Idiopathic BAD Post-op CD
0.8651792524790236
3147 B55 C78 Idiopathic BAD Post-op CD
0.8794813119755912
3148 B59 C1 Idiopathic BAD Post-op CD
0.8316170861937452
3149 B59 C3 Idiopathic BAD Post-op CD
0.8089244851258581
3150 B59 C7 Idiopathic BAD Post-op CD
0.9900839054157132
3151 B59 C8 Idiopathic BAD Post-op CD
0.9225781845919145
3152 B59 C11 Idiopathic BAD Post-op CD
0.9910373760488177
3153 B59 C15 Idiopathic BAD Post-op CD
0.9456521739130435
3154 B59 C19 Idiopathic BAD Post-op CD
0.9832189168573608
3155 B59 C22 Idiopathic BAD Post-op CD
0.7492372234935164
3156 B59 C26 Idiopathic BAD Post-op CD
0.9897025171624714
3157 B59 C28 Idiopathic BAD Post-op CD
0.9849351639969489
3158 B59 C31 Idiopathic BAD Post-op CD
0.9839816933638444
3159 B59 C35 Idiopathic BAD Post-op CD
0.9849351639969489
3160 B59 C38 Idiopathic BAD Post-op CD
0.9338291380625476
3161 B59 C40 Idiopathic BAD Post-op CD
0.9765446224256293
3162 B59 C44 Idiopathic BAD Post-op CD
0.9816933638443935
3163 B59 C47 Idiopathic BAD Post-op CD
0.9839816933638444
3164 B59 C48 Idiopathic BAD Post-op CD
0.9521357742181541
3165 B59 C49 Idiopathic BAD Post-op CD
0.9366895499618612
3166 B59 C53 Idiopathic BAD Post-op CD
0.9643401983218917
3167 B59 C56 Idiopathic BAD Post-op CD
0.9530892448512586
3168 B59 C60 Idiopathic BAD Post-op CD
0.8525934401220442
3169 B59 C62 Idiopathic BAD Post-op CD
0.9565217391304348
3170 B59 C64 Idiopathic BAD Post-op CD
0.9437452326468345
3171 B59 C65 Idiopathic BAD Post-op CD

0.7532418001525553
3172	B59	C69	Idiopathic BAD	Post-op CD
0.9626239511823036
3173	B59	C70	Idiopathic BAD	Post-op CD
0.9639588100686499
3174	B59	C74	Idiopathic BAD	Post-op CD
0.8150266971777269
3175	B59	C78	Idiopathic BAD	Post-op CD
0.9830282227307399
3176	B70	C1	Idiopathic BAD	Post-op CD
0.9746376811594203
3177	B70	C3	Idiopathic BAD	Post-op CD
0.9631960335621663
3178	B70	C7	Idiopathic BAD	Post-op CD
0.915903890160183
3179	B70	C8	Idiopathic BAD	Post-op CD
0.9666285278413425
3180	B70	C11	Idiopathic BAD	Post-op CD
0.7082379862700229
3181	B70	C15	Idiopathic BAD	Post-op CD
0.6361556064073226
3182	B70	C19	Idiopathic BAD	Post-op CD
0.7200610221205187
3183	B70	C22	Idiopathic BAD	Post-op CD
0.9689168573607932
3184	B70	C26	Idiopathic BAD	Post-op CD
0.9010297482837528
3185	B70	C28	Idiopathic BAD	Post-op CD
0.7170099160945843
3186	B70	C31	Idiopathic BAD	Post-op CD
0.9063691838291381
3187	B70	C35	Idiopathic BAD	Post-op CD	0.75
3188	B70	C38	Idiopathic BAD	Post-op CD
0.977116704805492
3189	B70	C40	Idiopathic BAD	Post-op CD
0.9098016781083142
3190	B70	C44	Idiopathic BAD	Post-op CD
0.6632341723874905
3191	B70	C47	Idiopathic BAD	Post-op CD
0.7011823035850496
3192	B70	C48	Idiopathic BAD	Post-op CD
0.9157131960335622
3193	B70	C49	Idiopathic BAD	Post-op CD
0.9414569031273837
3194	B70	C53	Idiopathic BAD	Post-op CD
0.8011060259344012
3195	B70	C56	Idiopathic BAD	Post-op CD
0.7524790236460717
3196	B70	C60	Idiopathic BAD	Post-op CD
0.7265446224256293
3197	B70	C62	Idiopathic BAD	Post-op CD
0.9016018306636155
3198	B70	C64	Idiopathic BAD	Post-op CD
0.8670861937452327

3199	B70	C65	Idiopathic BAD	Post-op CD
0.9361174675819984
3200	B70	C69	Idiopathic BAD	Post-op CD
0.881578947368421
3201	B70	C70	Idiopathic BAD	Post-op CD
0.9448893974065599
3202	B70	C74	Idiopathic BAD	Post-op CD
0.9578565980167811
3203	B70	C78	Idiopathic BAD	Post-op CD
0.8558352402745996
3204	B74	C1	Idiopathic BAD	Post-op CD
0.9773073989321129
3205	B74	C3	Idiopathic BAD	Post-op CD
0.7889016018306636
3206	B74	C7	Idiopathic BAD	Post-op CD
0.9487032799389779
3207	B74	C8	Idiopathic BAD	Post-op CD
0.7236842105263158
3208	B74	C11	Idiopathic BAD	Post-op CD
0.9460335621662853
3209	B74	C15	Idiopathic BAD	Post-op CD
0.9452707856598017
3210	B74	C19	Idiopathic BAD	Post-op CD
0.9191456903127384
3211	B74	C22	Idiopathic BAD	Post-op CD
0.8937833714721587
3212	B74	C26	Idiopathic BAD	Post-op CD
0.9487032799389779
3213	B74	C28	Idiopathic BAD	Post-op CD
0.944698703279939
3214	B74	C31	Idiopathic BAD	Post-op CD
0.9944698703279939
3215	B74	C35	Idiopathic BAD	Post-op CD
0.9427917620137299
3216	B74	C38	Idiopathic BAD	Post-op CD
0.9860793287566743
3217	B74	C40	Idiopathic BAD	Post-op CD
0.9429824561403509
3218	B74	C44	Idiopathic BAD	Post-op CD
0.9509916094584286
3219	B74	C47	Idiopathic BAD	Post-op CD
0.948512585812357
3220	B74	C48	Idiopathic BAD	Post-op CD
0.9010297482837528
3221	B74	C49	Idiopathic BAD	Post-op CD
0.9445080091533181
3222	B74	C53	Idiopathic BAD	Post-op CD
0.9189549961861174
3223	B74	C56	Idiopathic BAD	Post-op CD
0.8928299008390541
3224	B74	C60	Idiopathic BAD	Post-op CD
0.8579328756674295
3225	B74	C62	Idiopathic BAD	Post-op CD
0.9517543859649122

3226	B74	C64	Idiopathic BAD	Post-op CD
0.8983600305110603
3227	B74	C65	Idiopathic BAD	Post-op CD
0.9242944317315027
3228	B74	C69	Idiopathic BAD	Post-op CD
0.9405034324942791
3229	B74	C70	Idiopathic BAD	Post-op CD
0.8979786422578184
3230	B74	C74	Idiopathic BAD	Post-op CD
0.9435545385202135
3231	B74	C78	Idiopathic BAD	Post-op CD
0.9174294431731502
3232	B77	C1	Idiopathic BAD	Post-op CD
0.952326468344775
3233	B77	C3	Idiopathic BAD	Post-op CD
0.9014111365369947
3234	B77	C7	Idiopathic BAD	Post-op CD
0.9061784897025171
3235	B77	C8	Idiopathic BAD	Post-op CD
0.938977879481312
3236	B77	C11	Idiopathic BAD	Post-op CD
0.8516399694889397
3237	B77	C15	Idiopathic BAD	Post-op CD
0.8173150266971777
3238	B77	C19	Idiopathic BAD	Post-op CD
0.8237986270022883
3239	B77	C22	Idiopathic BAD	Post-op CD
0.8625095347063311
3240	B77	C26	Idiopathic BAD	Post-op CD
0.898741418764302
3241	B77	C28	Idiopathic BAD	Post-op CD
0.8331426392067124
3242	B77	C31	Idiopathic BAD	Post-op CD
0.9044622425629291
3243	B77	C35	Idiopathic BAD	Post-op CD
0.8072082379862701
3244	B77	C38	Idiopathic BAD	Post-op CD
0.9405034324942791
3245	B77	C40	Idiopathic BAD	Post-op CD
0.9326849733028223
3246	B77	C44	Idiopathic BAD	Post-op CD
0.8352402745995423
3247	B77	C47	Idiopathic BAD	Post-op CD
0.8609839816933639
3248	B77	C48	Idiopathic BAD	Post-op CD
0.8180778032036613
3249	B77	C49	Idiopathic BAD	Post-op CD
0.8852021357742181
3250	B77	C53	Idiopathic BAD	Post-op CD
0.7742181540808543
3251	B77	C56	Idiopathic BAD	Post-op CD
0.7416094584286804
3252	B77	C60	Idiopathic BAD	Post-op CD
0.7692601067887109

3253	B77	C62	Idiopathic BAD	Post-op CD
0.8432494279176201
3254	B77	C64	Idiopathic BAD	Post-op CD
0.8346681922196796
3255	B77	C65	Idiopathic BAD	Post-op CD
0.8255148741418764
3256	B77	C69	Idiopathic BAD	Post-op CD
0.86441647597254
3257	B77	C70	Idiopathic BAD	Post-op CD
0.8407704042715485
3258	B77	C74	Idiopathic BAD	Post-op CD
0.9002669717772692
3259	B77	C78	Idiopathic BAD	Post-op CD
0.8073989321128909
3260	B81	C1	Idiopathic BAD	Post-op CD
0.8596491228070176
3261	B81	C3	Idiopathic BAD	Post-op CD
0.9565217391304348
3262	B81	C7	Idiopathic BAD	Post-op CD
0.9385964912280702
3263	B81	C8	Idiopathic BAD	Post-op CD
0.9195270785659801
3264	B81	C11	Idiopathic BAD	Post-op CD
0.8653699466056446
3265	B81	C15	Idiopathic BAD	Post-op CD
0.9479405034324943
3266	B81	C19	Idiopathic BAD	Post-op CD
0.9078947368421053
3267	B81	C22	Idiopathic BAD	Post-op CD
0.9666285278413425
3268	B81	C26	Idiopathic BAD	Post-op CD
0.9344012204424104
3269	B81	C28	Idiopathic BAD	Post-op CD
0.9281083142639207
3270	B81	C31	Idiopathic BAD	Post-op CD
0.9837909992372235
3271	B81	C35	Idiopathic BAD	Post-op CD
0.9628146453089245
3272	B81	C38	Idiopathic BAD	Post-op CD
0.9448893974065599
3273	B81	C40	Idiopathic BAD	Post-op CD
0.8287566742944318
3274	B81	C44	Idiopathic BAD	Post-op CD
0.8752860411899314
3275	B81	C47	Idiopathic BAD	Post-op CD
0.9490846681922197
3276	B81	C48	Idiopathic BAD	Post-op CD
0.8802440884820748
3277	B81	C49	Idiopathic BAD	Post-op CD
0.9218154080854309
3278	B81	C53	Idiopathic BAD	Post-op CD
0.9214340198321892
3279	B81	C56	Idiopathic BAD	Post-op CD
0.864607170099161

3280	B81	C60	Idiopathic BAD	Post-op CD
0.9134248665141114
3281	B81	C62	Idiopathic BAD	Post-op CD
0.9397406559877955
3282	B81	C64	Idiopathic BAD	Post-op CD
0.9044622425629291
3283	B81	C65	Idiopathic BAD	Post-op CD
0.9170480549199085
3284	B81	C69	Idiopathic BAD	Post-op CD
0.8533562166285278
3285	B81	C70	Idiopathic BAD	Post-op CD
0.7738367658276125
3286	B81	C74	Idiopathic BAD	Post-op CD
0.9374523264683448
3287	B81	C78	Idiopathic BAD	Post-op CD
0.8670861937452327
3288	B84	C1	Idiopathic BAD	Post-op CD
0.9105644546147978
3289	B84	C3	Idiopathic BAD	Post-op CD
0.8859649122807017
3290	B84	C7	Idiopathic BAD	Post-op CD
0.8956903127383676
3291	B84	C8	Idiopathic BAD	Post-op CD
0.9214340198321892
3292	B84	C11	Idiopathic BAD	Post-op CD
0.88558352402746
3293	B84	C15	Idiopathic BAD	Post-op CD
0.8188405797101449
3294	B84	C19	Idiopathic BAD	Post-op CD
0.8676582761250954
3295	B84	C22	Idiopathic BAD	Post-op CD
0.9056064073226545
3296	B84	C26	Idiopathic BAD	Post-op CD
0.9061784897025171
3297	B84	C28	Idiopathic BAD	Post-op CD
0.8236079328756675
3298	B84	C31	Idiopathic BAD	Post-op CD
0.9616704805491991
3299	B84	C35	Idiopathic BAD	Post-op CD
0.8173150266971777
3300	B84	C38	Idiopathic BAD	Post-op CD
0.9549961861174676
3301	B84	C40	Idiopathic BAD	Post-op CD
0.9305873379099924
3302	B84	C44	Idiopathic BAD	Post-op CD
0.8832951945080092
3303	B84	C47	Idiopathic BAD	Post-op CD
0.8914950419527079
3304	B84	C48	Idiopathic BAD	Post-op CD
0.8810068649885584
3305	B84	C49	Idiopathic BAD	Post-op CD
0.916094584286804
3306	B84	C53	Idiopathic BAD	Post-op CD
0.8466819221967964

3307	B84	C56	Idiopathic BAD	Post-op CD
0.8218916857360793
3308	B84	C60	Idiopathic BAD	Post-op CD
0.7660183066361556
3309	B84	C62	Idiopathic BAD	Post-op CD
0.9040808543096872
3310	B84	C64	Idiopathic BAD	Post-op CD
0.8775743707093822
3311	B84	C65	Idiopathic BAD	Post-op CD
0.8562166285278413
3312	B84	C69	Idiopathic BAD	Post-op CD
0.8956903127383676
3313	B84	C70	Idiopathic BAD	Post-op CD
0.9052250190694127
3314	B84	C74	Idiopathic BAD	Post-op CD
0.8857742181540809
3315	B84	C78	Idiopathic BAD	Post-op CD
0.8401983218916858
3316	B89	C1	Idiopathic BAD	Post-op CD
0.9717772692601068
3317	B89	C3	Idiopathic BAD	Post-op CD
0.9477498093058734
3318	B89	C7	Idiopathic BAD	Post-op CD
0.910373760488177
3319	B89	C8	Idiopathic BAD	Post-op CD
0.9519450800915332
3320	B89	C11	Idiopathic BAD	Post-op CD
0.8954996186117468
3321	B89	C15	Idiopathic BAD	Post-op CD
0.8375286041189931
3322	B89	C19	Idiopathic BAD	Post-op CD
0.8741418764302059
3323	B89	C22	Idiopathic BAD	Post-op CD
0.9746376811594203
3324	B89	C26	Idiopathic BAD	Post-op CD
0.858886346300534
3325	B89	C28	Idiopathic BAD	Post-op CD
0.9218154080854309
3326	B89	C31	Idiopathic BAD	Post-op CD
0.9851258581235698
3327	B89	C35	Idiopathic BAD	Post-op CD
0.9397406559877955
3328	B89	C38	Idiopathic BAD	Post-op CD
0.9807398932112891
3329	B89	C40	Idiopathic BAD	Post-op CD
0.8878718535469108
3330	B89	C44	Idiopathic BAD	Post-op CD
0.9164759725400458
3331	B89	C47	Idiopathic BAD	Post-op CD
0.9643401983218917
3332	B89	C48	Idiopathic BAD	Post-op CD
0.9157131960335622
3333	B89	C49	Idiopathic BAD	Post-op CD
0.8924485125858124

3334 B89 C53 Idiopathic BAD Post-op CD
0.9256292906178489
3335 B89 C56 Idiopathic BAD Post-op CD
0.8729977116704806
3336 B89 C60 Idiopathic BAD Post-op CD
0.9410755148741419
3337 B89 C62 Idiopathic BAD Post-op CD
0.9242944317315027
3338 B89 C64 Idiopathic BAD Post-op CD
0.9265827612509535
3339 B89 C65 Idiopathic BAD Post-op CD
0.9328756674294432
3340 B89 C69 Idiopathic BAD Post-op CD
0.8285659801678108
3341 B89 C70 Idiopathic BAD Post-op CD
0.8884439359267735
3342 B89 C74 Idiopathic BAD Post-op CD
0.9458428680396643
3343 B89 C78 Idiopathic BAD Post-op CD
0.7698321891685737
3344 B92 C1 Idiopathic BAD Post-op CD
0.9355453852021358
3345 B92 C3 Idiopathic BAD Post-op CD
0.877765064836003
3346 B92 C7 Idiopathic BAD Post-op CD
0.5797101449275363
3347 B92 C8 Idiopathic BAD Post-op CD
0.908276125095347
3348 B92 C11 Idiopathic BAD Post-op CD
0.8691838291380626
3349 B92 C15 Idiopathic BAD Post-op CD
0.8508771929824561
3350 B92 C19 Idiopathic BAD Post-op CD
0.6401601830663616
3351 B92 C22 Idiopathic BAD Post-op CD
0.9269641495041953
3352 B92 C26 Idiopathic BAD Post-op CD
0.8598398169336384
3353 B92 C28 Idiopathic BAD Post-op CD
0.8581235697940504
3354 B92 C31 Idiopathic BAD Post-op CD
0.9385964912280702
3355 B92 C35 Idiopathic BAD Post-op CD
0.6838291380625476
3356 B92 C38 Idiopathic BAD Post-op CD
0.9786422578184591
3357 B92 C40 Idiopathic BAD Post-op CD
0.9300152555301296
3358 B92 C44 Idiopathic BAD Post-op CD
0.8897787948131197
3359 B92 C47 Idiopathic BAD Post-op CD
0.9016018306636155
3360 B92 C48 Idiopathic BAD Post-op CD
0.5968726163234173

3361	B92	C49	Idiopathic BAD	Post-op CD
0.818649885583524
3362	B92	C53	Idiopathic BAD	Post-op CD
0.740465293668955
3363	B92	C56	Idiopathic BAD	Post-op CD
0.719488939740656
3364	B92	C60	Idiopathic BAD	Post-op CD
0.7475209763539283
3365	B92	C62	Idiopathic BAD	Post-op CD
0.8041571319603357
3366	B92	C64	Idiopathic BAD	Post-op CD
0.7610602593440122
3367	B92	C65	Idiopathic BAD	Post-op CD
0.7713577421815409
3368	B92	C69	Idiopathic BAD	Post-op CD
0.7086193745232647
3369	B92	C70	Idiopathic BAD	Post-op CD
0.7807017543859649
3370	B92	C74	Idiopathic BAD	Post-op CD
0.8157894736842105
3371	B92	C78	Idiopathic BAD	Post-op CD
0.8064454614797865
3372	B95	C1	Idiopathic BAD	Post-op CD
0.9374523264683448
3373	B95	C3	Idiopathic BAD	Post-op CD
0.9586193745232647
3374	B95	C7	Idiopathic BAD	Post-op CD
0.7210144927536232
3375	B95	C8	Idiopathic BAD	Post-op CD
0.9836003051106026
3376	B95	C11	Idiopathic BAD	Post-op CD
0.9479405034324943
3377	B95	C15	Idiopathic BAD	Post-op CD
0.864607170099161
3378	B95	C19	Idiopathic BAD	Post-op CD
0.8442028985507246
3379	B95	C22	Idiopathic BAD	Post-op CD
0.9778794813119756
3380	B95	C26	Idiopathic BAD	Post-op CD
0.8709000762776506
3381	B95	C28	Idiopathic BAD	Post-op CD
0.9530892448512586
3382	B95	C31	Idiopathic BAD	Post-op CD
0.9805491990846682
3383	B95	C35	Idiopathic BAD	Post-op CD
0.7387490465293669
3384	B95	C38	Idiopathic BAD	Post-op CD
0.9839816933638444
3385	B95	C40	Idiopathic BAD	Post-op CD
0.847254004576659
3386	B95	C44	Idiopathic BAD	Post-op CD
0.937070938215103
3387	B95	C47	Idiopathic BAD	Post-op CD
0.971395881006865

3388	B95	C48	Idiopathic BAD	Post-op CD	0.7358886346300534
3389	B95	C49	Idiopathic BAD	Post-op CD	0.801487414187643
3390	B95	C53	Idiopathic BAD	Post-op CD	0.837909992372235
3391	B95	C56	Idiopathic BAD	Post-op CD	0.7763157894736842
3392	B95	C60	Idiopathic BAD	Post-op CD	0.8333333333333334
3393	B95	C62	Idiopathic BAD	Post-op CD	0.8476353928299009
3394	B95	C64	Idiopathic BAD	Post-op CD	0.8440122044241037
3395	B95	C65	Idiopathic BAD	Post-op CD	0.8411517925247902
3396	B95	C69	Idiopathic BAD	Post-op CD	0.761632341723875
3397	B95	C70	Idiopathic BAD	Post-op CD	0.8228451563691839
3398	B95	C74	Idiopathic BAD	Post-op CD	0.8745232646834478
3399	B95	C78	Idiopathic BAD	Post-op CD	0.843440122044241
3400	B99	C1	Idiopathic BAD	Post-op CD	0.9698703279938978
3401	B99	C3	Idiopathic BAD	Post-op CD	0.9439359267734554
3402	B99	C7	Idiopathic BAD	Post-op CD	0.9016018306636155
3403	B99	C8	Idiopathic BAD	Post-op CD	0.9557589626239512
3404	B99	C11	Idiopathic BAD	Post-op CD	0.86441647597254
3405	B99	C15	Idiopathic BAD	Post-op CD	0.8026315789473685
3406	B99	C19	Idiopathic BAD	Post-op CD	0.8230358504958047
3407	B99	C22	Idiopathic BAD	Post-op CD	0.9391685736079328
3408	B99	C26	Idiopathic BAD	Post-op CD	0.8741418764302059
3409	B99	C28	Idiopathic BAD	Post-op CD	0.8495423340961098
3410	B99	C31	Idiopathic BAD	Post-op CD	0.86441647597254
3411	B99	C35	Idiopathic BAD	Post-op CD	0.8661327231121282
3412	B99	C38	Idiopathic BAD	Post-op CD	0.9631960335621663
3413	B99	C40	Idiopathic BAD	Post-op CD	0.881769641495042
3414	B99	C44	Idiopathic BAD	Post-op CD	0.8794813119755912

3415 B99 C47 Idiopathic BAD Post-op CD
0.877765064836003
3416 B99 C48 Idiopathic BAD Post-op CD
0.8583142639206712
3417 B99 C49 Idiopathic BAD Post-op CD
0.8688024408848207
3418 B99 C53 Idiopathic BAD Post-op CD
0.8861556064073226
3419 B99 C56 Idiopathic BAD Post-op CD
0.7898550724637681
3420 B99 C60 Idiopathic BAD Post-op CD
0.8602212051868803
3421 B99 C62 Idiopathic BAD Post-op CD
0.8272311212814645
3422 B99 C64 Idiopathic BAD Post-op CD
0.8865369946605645
3423 B99 C65 Idiopathic BAD Post-op CD
0.8928299008390541
3424 B99 C69 Idiopathic BAD Post-op CD
0.7871853546910755
3425 B99 C70 Idiopathic BAD Post-op CD
0.8695652173913043
3426 B99 C74 Idiopathic BAD Post-op CD
0.9050343249427918
3427 B99 C78 Idiopathic BAD Post-op CD
0.7044241037376049
3428 B103 C1 Idiopathic BAD Post-op CD
0.9643401983218917
3429 B103 C3 Idiopathic BAD Post-op CD
0.9816933638443935
3430 B103 C7 Idiopathic BAD Post-op CD
0.912090007627765
3431 B103 C8 Idiopathic BAD Post-op CD
0.9437452326468345
3432 B103 C11 Idiopathic BAD Post-op CD
0.7734553775743707
3433 B103 C15 Idiopathic BAD Post-op CD
0.9576659038901602
3434 B103 C19 Idiopathic BAD Post-op CD
0.8098779557589626
3435 B103 C22 Idiopathic BAD Post-op CD
0.9696796338672768
3436 B103 C26 Idiopathic BAD Post-op CD
0.9553775743707094
3437 B103 C28 Idiopathic BAD Post-op CD
0.8306636155606407
3438 B103 C31 Idiopathic BAD Post-op CD
0.9273455377574371
3439 B103 C35 Idiopathic BAD Post-op CD
0.6842105263157895
3440 B103 C38 Idiopathic BAD Post-op CD
0.9824561403508771
3441 B103 C40 Idiopathic BAD Post-op CD
0.919908466819222

3442   B103   C44   Idiopathic BAD Post-op CD
0.8676582761250954
3443   B103   C47   Idiopathic BAD Post-op CD
0.811022120518688
3444   B103   C48   Idiopathic BAD Post-op CD
0.9385964912280702
3445   B103   C49   Idiopathic BAD Post-op CD
0.9677726926010679
3446   B103   C53   Idiopathic BAD Post-op CD
0.7606788710907704
3447   B103   C56   Idiopathic BAD Post-op CD
0.9267734553775744
3448   B103   C60   Idiopathic BAD Post-op CD
0.9450800915331807
3449   B103   C62   Idiopathic BAD Post-op CD
0.8958810068649885
3450   B103   C64   Idiopathic BAD Post-op CD
0.9363081617086194
3451   B103   C65   Idiopathic BAD Post-op CD
0.9551868802440885
3452   B103   C69   Idiopathic BAD Post-op CD
0.897025171624714
3453   B103   C70   Idiopathic BAD Post-op CD
0.9132341723874905
3454   B103   C74   Idiopathic BAD Post-op CD
0.9727307398932112
3455   B103   C78   Idiopathic BAD Post-op CD
0.8741418764302059
3456   B106   C1   Idiopathic BAD Post-op CD
0.9830282227307399
3457   B106   C3   Idiopathic BAD Post-op CD
0.9723493516399695
3458   B106   C7   Idiopathic BAD Post-op CD
0.5240274599542334
3459   B106   C8   Idiopathic BAD Post-op CD
0.9780701754385965
3460   B106   C11   Idiopathic BAD Post-op CD
0.9849351639969489
3461   B106   C15   Idiopathic BAD Post-op CD
0.9754004576659039
3462   B106   C19   Idiopathic BAD Post-op CD
0.5909610983981693
3463   B106   C22   Idiopathic BAD Post-op CD
0.9803585049580473
3464   B106   C26   Idiopathic BAD Post-op CD
0.9851258581235698
3465   B106   C28   Idiopathic BAD Post-op CD
0.9782608695652174
3466   B106   C31   Idiopathic BAD Post-op CD
0.9937070938215103
3467   B106   C35   Idiopathic BAD Post-op CD
0.9040808543096872
3468   B106   C38   Idiopathic BAD Post-op CD
0.9916094584286804

3469 B106 C40 Idiopathic BAD Post-op CD
0.9773073989321129
3470 B106 C44 Idiopathic BAD Post-op CD
0.9795957284515637
3471 B106 C47 Idiopathic BAD Post-op CD
0.9672006102212052
3472 B106 C48 Idiopathic BAD Post-op CD
0.7093821510297483
3473 B106 C49 Idiopathic BAD Post-op CD
0.8367658276125095
3474 B106 C53 Idiopathic BAD Post-op CD
0.8041571319603357
3475 B106 C56 Idiopathic BAD Post-op CD
0.7721205186880244
3476 B106 C60 Idiopathic BAD Post-op CD
0.822463768115942
3477 B106 C62 Idiopathic BAD Post-op CD
0.8390541571319603
3478 B106 C64 Idiopathic BAD Post-op CD
0.8070175438596491
3479 B106 C65 Idiopathic BAD Post-op CD
0.7929061784897025
3480 B106 C69 Idiopathic BAD Post-op CD
0.78813882532418
3481 B106 C70 Idiopathic BAD Post-op CD
0.7999618611746758
3482 B106 C74 Idiopathic BAD Post-op CD
0.8369565217391305
3483 B106 C78 Idiopathic BAD Post-op CD
0.9513729977116705
3484 B109 C1 Idiopathic BAD Post-op CD
0.9433638443935927
3485 B109 C3 Idiopathic BAD Post-op CD
0.9677726926010679
3486 B109 C7 Idiopathic BAD Post-op CD
0.9088482074752098
3487 B109 C8 Idiopathic BAD Post-op CD
0.9647215865751335
3488 B109 C11 Idiopathic BAD Post-op CD
0.7869946605644547
3489 B109 C15 Idiopathic BAD Post-op CD
0.8415331807780321
3490 B109 C19 Idiopathic BAD Post-op CD
0.8272311212814645
3491 B109 C22 Idiopathic BAD Post-op CD
0.9692982456140351
3492 B109 C26 Idiopathic BAD Post-op CD
0.9511823035850496
3493 B109 C28 Idiopathic BAD Post-op CD
0.7419908466819222
3494 B109 C31 Idiopathic BAD Post-op CD
0.9239130434782609
3495 B109 C35 Idiopathic BAD Post-op CD
0.8535469107551488

3496 B109 C38 Idiopathic BAD Post-op CD
0.9940884820747521
3497 B109 C40 Idiopathic BAD Post-op CD
0.9019832189168574
3498 B109 C44 Idiopathic BAD Post-op CD
0.7915713196033562
3499 B109 C47 Idiopathic BAD Post-op CD
0.8565980167810832
3500 B109 C48 Idiopathic BAD Post-op CD
0.8953089244851259
3501 B109 C49 Idiopathic BAD Post-op CD
0.9420289855072463
3502 B109 C53 Idiopathic BAD Post-op CD
0.8569794050343249
3503 B109 C56 Idiopathic BAD Post-op CD
0.7753623188405797
3504 B109 C60 Idiopathic BAD Post-op CD
0.8459191456903128
3505 B109 C62 Idiopathic BAD Post-op CD
0.8779557589626239
3506 B109 C64 Idiopathic BAD Post-op CD
0.9134248665141114
3507 B109 C65 Idiopathic BAD Post-op CD
0.9195270785659801
3508 B109 C69 Idiopathic BAD Post-op CD
0.8560259344012204
3509 B109 C70 Idiopathic BAD Post-op CD
0.9260106788710908
3510 B109 C74 Idiopathic BAD Post-op CD
0.9420289855072463
3511 B109 C78 Idiopathic BAD Post-op CD
0.7379862700228833
3512 B118 C1 Idiopathic BAD Post-op CD
0.9794050343249427
3513 B118 C3 Idiopathic BAD Post-op CD
0.9452707856598017
3514 B118 C7 Idiopathic BAD Post-op CD
0.6462623951182304
3515 B118 C8 Idiopathic BAD Post-op CD
0.9811212814645309
3516 B118 C11 Idiopathic BAD Post-op CD
0.9376430205949656
3517 B118 C15 Idiopathic BAD Post-op CD
0.9303966437833715
3518 B118 C19 Idiopathic BAD Post-op CD
0.6485507246376812
3519 B118 C22 Idiopathic BAD Post-op CD
0.9698703279938978
3520 B118 C26 Idiopathic BAD Post-op CD
0.9654843630816171
3521 B118 C28 Idiopathic BAD Post-op CD
0.9395499618611747
3522 B118 C31 Idiopathic BAD Post-op CD
0.9877955758962624

3523 B118 C35 Idiopathic BAD Post-op CD
0.8468726163234173
3524 B118 C38 Idiopathic BAD Post-op CD
0.9900839054157132
3525 B118 C40 Idiopathic BAD Post-op CD
0.9624332570556827
3526 B118 C44 Idiopathic BAD Post-op CD
0.9437452326468345
3527 B118 C47 Idiopathic BAD Post-op CD
0.9481311975591151
3528 B118 C48 Idiopathic BAD Post-op CD
0.6887871853546911
3529 B118 C49 Idiopathic BAD Post-op CD
0.8470633104500381
3530 B118 C53 Idiopathic BAD Post-op CD
0.8114035087719298
3531 B118 C56 Idiopathic BAD Post-op CD
0.7753623188405797
3532 B118 C60 Idiopathic BAD Post-op CD
0.8089244851258581
3533 B118 C62 Idiopathic BAD Post-op CD
0.830091533180778
3534 B118 C64 Idiopathic BAD Post-op CD
0.7948131197559115
3535 B118 C65 Idiopathic BAD Post-op CD
0.8049199084668193
3536 B118 C69 Idiopathic BAD Post-op CD
0.7839435545385202
3537 B118 C70 Idiopathic BAD Post-op CD
0.8121662852784134
3538 B118 C74 Idiopathic BAD Post-op CD
0.8365751334858886
3539 B118 C78 Idiopathic BAD Post-op CD
0.9098016781083142
3540 B119 C1 Idiopathic BAD Post-op CD
0.9464149504195271
3541 B119 C3 Idiopathic BAD Post-op CD
0.9286803966437833
3542 B119 C7 Idiopathic BAD Post-op CD
0.9042715484363082
3543 B119 C8 Idiopathic BAD Post-op CD
0.8688024408848207
3544 B119 C11 Idiopathic BAD Post-op CD
0.7572463768115942
3545 B119 C15 Idiopathic BAD Post-op CD
0.7183447749809306
3546 B119 C19 Idiopathic BAD Post-op CD
0.7587719298245614
3547 B119 C22 Idiopathic BAD Post-op CD
0.9620518688024409
3548 B119 C26 Idiopathic BAD Post-op CD
0.851067887109077
3549 B119 C28 Idiopathic BAD Post-op CD
0.7229214340198322

3550	B119	C31	Idiopathic BAD	Post-op CD	0.9105644546147978
3551	B119	C35	Idiopathic BAD	Post-op CD	0.7587719298245614
3552	B119	C38	Idiopathic BAD	Post-op CD	0.9225781845919145
3553	B119	C40	Idiopathic BAD	Post-op CD	0.8257055682684973
3554	B119	C44	Idiopathic BAD	Post-op CD	0.7292143401983219
3555	B119	C47	Idiopathic BAD	Post-op CD	0.8175057208237986
3556	B119	C48	Idiopathic BAD	Post-op CD	0.8768115942028986
3557	B119	C49	Idiopathic BAD	Post-op CD	0.9164759725400458
3558	B119	C53	Idiopathic BAD	Post-op CD	0.7990083905415714
3559	B119	C56	Idiopathic BAD	Post-op CD	0.6857360793287567
3560	B119	C60	Idiopathic BAD	Post-op CD	0.7845156369183829
3561	B119	C62	Idiopathic BAD	Post-op CD	0.8737604881769642
3562	B119	C64	Idiopathic BAD	Post-op CD	0.9099923722349351
3563	B119	C65	Idiopathic BAD	Post-op CD	0.929252479023646
3564	B119	C69	Idiopathic BAD	Post-op CD	0.8527841342486652
3565	B119	C70	Idiopathic BAD	Post-op CD	0.9059877955758963
3566	B119	C74	Idiopathic BAD	Post-op CD	0.9321128909229596
3567	B119	C78	Idiopathic BAD	Post-op CD	0.7149122807017544
0	P2	P1	Post-cholecystectomy	Post-cholecystectomy	0.9046529366895499
1	P4	P1	Post-cholecystectomy	Post-cholecystectomy	0.9385964912280702
2	P4	P2	Post-cholecystectomy	Post-cholecystectomy	0.8501144164759725
3	P5	P1	Post-cholecystectomy	Post-cholecystectomy	0.9382151029748284
4	P5	P2	Post-cholecystectomy	Post-cholecystectomy	0.7446605644546148
5	P5	P4	Post-cholecystectomy	Post-cholecystectomy	0.7812738367658276
6	P9	P1	Post-cholecystectomy	Post-cholecystectomy	0.9357360793287567
7	P9	P2	Post-cholecystectomy	Post-cholecystectomy	0.8609839816933639
8	P9	P4	Post-cholecystectomy	Post-cholecystectomy	0.7902364607170099

9	P9	P5	Post-cholecystectomy	Post-cholecystectomy	0.7450419527078566
10	P13	P1	Post-cholecystectomy	Post-cholecystectomy	0.9254385964912281
11	P13	P2	Post-cholecystectomy	Post-cholecystectomy	0.8173150266971777
12	P13	P4	Post-cholecystectomy	Post-cholecystectomy	0.8314263920671243
13	P13	P5	Post-cholecystectomy	Post-cholecystectomy	0.7690694126620901
14	P13	P9	Post-cholecystectomy	Post-cholecystectomy	0.8709000762776506
15	P15	P1	Post-cholecystectomy	Post-cholecystectomy	0.9359267734553776
16	P15	P2	Post-cholecystectomy	Post-cholecystectomy	0.8001525553012967
17	P15	P4	Post-cholecystectomy	Post-cholecystectomy	0.7295957284515637
18	P15	P5	Post-cholecystectomy	Post-cholecystectomy	0.8253241800152555
19	P15	P9	Post-cholecystectomy	Post-cholecystectomy	0.9054157131960335
20	P15	P13	Post-cholecystectomy	Post-cholecystectomy	0.8834858886346301
21	P16	P1	Post-cholecystectomy	Post-cholecystectomy	0.8768115942028986
22	P16	P2	Post-cholecystectomy	Post-cholecystectomy	0.8152173913043478
23	P16	P4	Post-cholecystectomy	Post-cholecystectomy	0.6708619374523265
24	P16	P5	Post-cholecystectomy	Post-cholecystectomy	0.7856598016781083
25	P16	P9	Post-cholecystectomy	Post-cholecystectomy	0.7822273073989321
26	P16	P13	Post-cholecystectomy	Post-cholecystectomy	0.889397406559878
27	P16	P15	Post-cholecystectomy	Post-cholecystectomy	0.7818459191456903
28	P17	P1	Post-cholecystectomy	Post-cholecystectomy	0.9183829138062548
29	P17	P2	Post-cholecystectomy	Post-cholecystectomy	0.7667810831426392
30	P17	P4	Post-cholecystectomy	Post-cholecystectomy	0.8627002288329519
31	P17	P5	Post-cholecystectomy	Post-cholecystectomy	0.7822273073989321
32	P17	P9	Post-cholecystectomy	Post-cholecystectomy	0.7948131197559115
33	P17	P13	Post-cholecystectomy	Post-cholecystectomy	0.7955758962623951
34	P17	P15	Post-cholecystectomy	Post-cholecystectomy	0.8651792524790236
35	P17	P16	Post-cholecystectomy	Post-cholecystectomy	0.8401983218916858

36	P20	P1	Post−cholecystectomy	Post−cholecystectomy	0.8756674294431731
37	P20	P2	Post−cholecystectomy	Post−cholecystectomy	0.8478260869565217
38	P20	P4	Post−cholecystectomy	Post−cholecystectomy	0.6586575133485889
39	P20	P5	Post−cholecystectomy	Post−cholecystectomy	0.7581998474446987
40	P20	P9	Post−cholecystectomy	Post−cholecystectomy	0.7738367658276125
41	P20	P13	Post−cholecystectomy	Post−cholecystectomy	0.7183447749809306
42	P20	P15	Post−cholecystectomy	Post−cholecystectomy	0.7667810831426392
43	P20	P16	Post−cholecystectomy	Post−cholecystectomy	0.7419908466819222
44	P20	P17	Post−cholecystectomy	Post−cholecystectomy	0.8499237223493517
45	P21	P1	Post−cholecystectomy	Post−cholecystectomy	0.9298245614035088
46	P21	P2	Post−cholecystectomy	Post−cholecystectomy	0.7875667429443173
47	P21	P4	Post−cholecystectomy	Post−cholecystectomy	0.7814645308924485
48	P21	P5	Post−cholecystectomy	Post−cholecystectomy	0.7604881769641495
49	P21	P9	Post−cholecystectomy	Post−cholecystectomy	0.694698703279939
50	P21	P13	Post−cholecystectomy	Post−cholecystectomy	0.8327612509534706
51	P21	P15	Post−cholecystectomy	Post−cholecystectomy	0.8850114416475973
52	P21	P16	Post−cholecystectomy	Post−cholecystectomy	0.7892829900839055
53	P21	P17	Post−cholecystectomy	Post−cholecystectomy	0.8039664378337147
54	P21	P20	Post−cholecystectomy	Post−cholecystectomy	0.7414187643020596
55	P24	P1	Post−cholecystectomy	Post−cholecystectomy	0.9193363844393593
56	P24	P2	Post−cholecystectomy	Post−cholecystectomy	0.7520976353928299
57	P24	P4	Post−cholecystectomy	Post−cholecystectomy	0.8752860411899314
58	P24	P5	Post−cholecystectomy	Post−cholecystectomy	0.8001525553012967
59	P24	P9	Post−cholecystectomy	Post−cholecystectomy	0.9128527841342486
60	P24	P13	Post−cholecystectomy	Post−cholecystectomy	0.7191075514874142
61	P24	P15	Post−cholecystectomy	Post−cholecystectomy	0.8377192982456141
62	P24	P16	Post−cholecystectomy	Post−cholecystectomy	0.9187643020594966

63	P24	P17	Post−cholecystectomy	Post−cholecystectomy	0.8329519450800915
64	P24	P20	Post−cholecystectomy	Post−cholecystectomy	0.7902364607170099
65	P24	P21	Post−cholecystectomy	Post−cholecystectomy	0.8941647597254004
66	P26	P1	Post−cholecystectomy	Post−cholecystectomy	0.9631960335621663
67	P26	P2	Post−cholecystectomy	Post−cholecystectomy	0.8506864988558352
68	P26	P4	Post−cholecystectomy	Post−cholecystectomy	0.8255148741418764
69	P26	P5	Post−cholecystectomy	Post−cholecystectomy	0.8060640732265446
70	P26	P9	Post−cholecystectomy	Post−cholecystectomy	0.6897406559877955
71	P26	P13	Post−cholecystectomy	Post−cholecystectomy	0.8106407322654462
72	P26	P15	Post−cholecystectomy	Post−cholecystectomy	0.9153318077803204
73	P26	P16	Post−cholecystectomy	Post−cholecystectomy	0.7524790236460717
74	P26	P17	Post−cholecystectomy	Post−cholecystectomy	0.8539282990083905
75	P26	P20	Post−cholecystectomy	Post−cholecystectomy	0.7845156369183829
76	P26	P21	Post−cholecystectomy	Post−cholecystectomy	0.6847826086956522
77	P26	P24	Post−cholecystectomy	Post−cholecystectomy	0.9298245614035088
78	P30	P1	Post−cholecystectomy	Post−cholecystectomy	0.9010297482837528
79	P30	P2	Post−cholecystectomy	Post−cholecystectomy	0.919908466819222
80	P30	P4	Post−cholecystectomy	Post−cholecystectomy	0.814836003051106
81	P30	P5	Post−cholecystectomy	Post−cholecystectomy	0.8401983218916858
82	P30	P9	Post−cholecystectomy	Post−cholecystectomy	0.8157894736842105
83	P30	P13	Post−cholecystectomy	Post−cholecystectomy	0.8829138062547673
84	P30	P15	Post−cholecystectomy	Post−cholecystectomy	0.8663234172387491
85	P30	P16	Post−cholecystectomy	Post−cholecystectomy	0.7583905415713196
86	P30	P17	Post−cholecystectomy	Post−cholecystectomy	0.9017925247902364
87	P30	P20	Post−cholecystectomy	Post−cholecystectomy	0.9115179252479023
88	P30	P21	Post−cholecystectomy	Post−cholecystectomy	0.9239130434782609
89	P30	P24	Post−cholecystectomy	Post−cholecystectomy	0.8996948893974066

90	P30	P26	Post-cholecystectomy	Post-cholecystectomy	0.9246758199847445
91	P33	P1	Post-cholecystectomy	Post-cholecystectomy	0.9454614797864226
92	P33	P2	Post-cholecystectomy	Post-cholecystectomy	0.7780320366132724
93	P33	P4	Post-cholecystectomy	Post-cholecystectomy	0.7524790236460717
94	P33	P5	Post-cholecystectomy	Post-cholecystectomy	0.7051868802440885
95	P33	P9	Post-cholecystectomy	Post-cholecystectomy	0.7917620137299771
96	P33	P13	Post-cholecystectomy	Post-cholecystectomy	0.7982456140350878
97	P33	P15	Post-cholecystectomy	Post-cholecystectomy	0.8361937452326468
98	P33	P16	Post-cholecystectomy	Post-cholecystectomy	0.7009916094584286
99	P33	P17	Post-cholecystectomy	Post-cholecystectomy	0.7898550724637681
100	P33	P20	Post-cholecystectomy	Post-cholecystectomy	0.7807017543859649
101	P33	P21	Post-cholecystectomy	Post-cholecystectomy	0.7006102212051869
102	P33	P24	Post-cholecystectomy	Post-cholecystectomy	0.8770022883295194
103	P33	P26	Post-cholecystectomy	Post-cholecystectomy	0.7721205186880244
104	P33	P30	Post-cholecystectomy	Post-cholecystectomy	0.8914950419527079
105	P35	P1	Post-cholecystectomy	Post-cholecystectomy	0.9294431731502669
106	P35	P2	Post-cholecystectomy	Post-cholecystectomy	0.7950038138825324
107	P35	P4	Post-cholecystectomy	Post-cholecystectomy	0.8060640732265446
108	P35	P5	Post-cholecystectomy	Post-cholecystectomy	0.7574370709382151
109	P35	P9	Post-cholecystectomy	Post-cholecystectomy	0.7356979405034325
110	P35	P13	Post-cholecystectomy	Post-cholecystectomy	0.8253241800152555
111	P35	P15	Post-cholecystectomy	Post-cholecystectomy	0.8577421815408085
112	P35	P16	Post-cholecystectomy	Post-cholecystectomy	0.7759344012204424
113	P35	P17	Post-cholecystectomy	Post-cholecystectomy	0.7948131197559115
114	P35	P20	Post-cholecystectomy	Post-cholecystectomy	0.8068268497330282
115	P35	P21	Post-cholecystectomy	Post-cholecystectomy	0.6434019832189168
116	P35	P24	Post-cholecystectomy	Post-cholecystectomy	0.879862700228833

117	P35	P26	Post-cholecystectomy	Post-cholecystectomy	0.6632341723874905
118	P35	P30	Post-cholecystectomy	Post-cholecystectomy	0.9075133485888635
119	P35	P33	Post-cholecystectomy	Post-cholecystectomy	0.7381769641495042
120	P38	P1	Post-cholecystectomy	Post-cholecystectomy	0.9080854309687262
121	P38	P2	Post-cholecystectomy	Post-cholecystectomy	0.9170480549199085
122	P38	P4	Post-cholecystectomy	Post-cholecystectomy	0.912090007627765
123	P38	P5	Post-cholecystectomy	Post-cholecystectomy	0.9433638443935927
124	P38	P9	Post-cholecystectomy	Post-cholecystectomy	0.9031273836765827
125	P38	P13	Post-cholecystectomy	Post-cholecystectomy	0.9443173150266971
126	P38	P15	Post-cholecystectomy	Post-cholecystectomy	0.9168573607932876
127	P38	P16	Post-cholecystectomy	Post-cholecystectomy	0.9157131960335622
128	P38	P17	Post-cholecystectomy	Post-cholecystectomy	0.959954233409611
129	P38	P20	Post-cholecystectomy	Post-cholecystectomy	0.9323035850495804
130	P38	P21	Post-cholecystectomy	Post-cholecystectomy	0.910373760488177
131	P38	P24	Post-cholecystectomy	Post-cholecystectomy	0.9412662090007627
132	P38	P26	Post-cholecystectomy	Post-cholecystectomy	0.8964530892448512
133	P38	P30	Post-cholecystectomy	Post-cholecystectomy	0.8306636155606407
134	P38	P33	Post-cholecystectomy	Post-cholecystectomy	0.9105644546147978
135	P38	P35	Post-cholecystectomy	Post-cholecystectomy	0.8363844393592678
136	P39	P1	Post-cholecystectomy	Post-cholecystectomy	0.927536231884058
137	P39	P2	Post-cholecystectomy	Post-cholecystectomy	0.9092295957284515
138	P39	P4	Post-cholecystectomy	Post-cholecystectomy	0.7833714721586575
139	P39	P5	Post-cholecystectomy	Post-cholecystectomy	0.8138825324180016
140	P39	P9	Post-cholecystectomy	Post-cholecystectomy	0.8182684973302822
141	P39	P13	Post-cholecystectomy	Post-cholecystectomy	0.8934019832189168
142	P39	P15	Post-cholecystectomy	Post-cholecystectomy	0.7858504958047292
143	P39	P16	Post-cholecystectomy	Post-cholecystectomy	0.7465675057208238

144	P39	P17	Post-cholecystectomy	Post-cholecystectomy	0.9284897025171625
145	P39	P20	Post-cholecystectomy	Post-cholecystectomy	0.7345537757437071
146	P39	P21	Post-cholecystectomy	Post-cholecystectomy	0.7412280701754386
147	P39	P24	Post-cholecystectomy	Post-cholecystectomy	0.9263920671243325
148	P39	P26	Post-cholecystectomy	Post-cholecystectomy	0.8829138062547673
149	P39	P30	Post-cholecystectomy	Post-cholecystectomy	0.8819603356216629
150	P39	P33	Post-cholecystectomy	Post-cholecystectomy	0.841723874904653
151	P39	P35	Post-cholecystectomy	Post-cholecystectomy	0.8863463005339436
152	P39	P38	Post-cholecystectomy	Post-cholecystectomy	0.9256292906178489
153	P42	P1	Post-cholecystectomy	Post-cholecystectomy	0.9586193745232647
154	P42	P2	Post-cholecystectomy	Post-cholecystectomy	0.893211289092296
155	P42	P4	Post-cholecystectomy	Post-cholecystectomy	0.8886346300533944
156	P42	P5	Post-cholecystectomy	Post-cholecystectomy	0.8747139588100686
157	P42	P9	Post-cholecystectomy	Post-cholecystectomy	0.7172006102212052
158	P42	P13	Post-cholecystectomy	Post-cholecystectomy	0.9416475972540046
159	P42	P15	Post-cholecystectomy	Post-cholecystectomy	0.9300152555301296
160	P42	P16	Post-cholecystectomy	Post-cholecystectomy	0.8045385202135774
161	P42	P17	Post-cholecystectomy	Post-cholecystectomy	0.8531655225019069
162	P42	P20	Post-cholecystectomy	Post-cholecystectomy	0.9096109839816934
163	P42	P21	Post-cholecystectomy	Post-cholecystectomy	0.812929061784897
164	P42	P24	Post-cholecystectomy	Post-cholecystectomy	0.9595728451563692
165	P42	P26	Post-cholecystectomy	Post-cholecystectomy	0.7183447749809306
166	P42	P30	Post-cholecystectomy	Post-cholecystectomy	0.919717772692601
167	P42	P33	Post-cholecystectomy	Post-cholecystectomy	0.8253241800152555
168	P42	P35	Post-cholecystectomy	Post-cholecystectomy	0.7683066361556065
169	P42	P38	Post-cholecystectomy	Post-cholecystectomy	0.9067505720823799
170	P42	P39	Post-cholecystectomy	Post-cholecystectomy	0.9069412662090007

171	P43	P1	Post-cholecystectomy	Post-cholecystectomy	0.9279176201372997
172	P43	P2	Post-cholecystectomy	Post-cholecystectomy	0.8651792524790236
173	P43	P4	Post-cholecystectomy	Post-cholecystectomy	0.7063310450038138
174	P43	P5	Post-cholecystectomy	Post-cholecystectomy	0.7585812356979404
175	P43	P9	Post-cholecystectomy	Post-cholecystectomy	0.8457284515636918
176	P43	P13	Post-cholecystectomy	Post-cholecystectomy	0.7297864225781846
177	P43	P15	Post-cholecystectomy	Post-cholecystectomy	0.7707856598016781
178	P43	P16	Post-cholecystectomy	Post-cholecystectomy	0.7635392829900839
179	P43	P17	Post-cholecystectomy	Post-cholecystectomy	0.8371472158657514
180	P43	P20	Post-cholecystectomy	Post-cholecystectomy	0.6811594202898551
181	P43	P21	Post-cholecystectomy	Post-cholecystectomy	0.780511060259344
182	P43	P24	Post-cholecystectomy	Post-cholecystectomy	0.8276125095347063
183	P43	P26	Post-cholecystectomy	Post-cholecystectomy	0.7578184591914569
184	P43	P30	Post-cholecystectomy	Post-cholecystectomy	0.9056064073226545
185	P43	P33	Post-cholecystectomy	Post-cholecystectomy	0.7631578947368421
186	P43	P35	Post-cholecystectomy	Post-cholecystectomy	0.8609839816933639
187	P43	P38	Post-cholecystectomy	Post-cholecystectomy	0.9616704805491991
188	P43	P39	Post-cholecystectomy	Post-cholecystectomy	0.7599160945842868
189	P43	P42	Post-cholecystectomy	Post-cholecystectomy	0.9572845156369184
190	P46	P1	Post-cholecystectomy	Post-cholecystectomy	0.9567124332570557
191	P46	P2	Post-cholecystectomy	Post-cholecystectomy	0.8306636155606407
192	P46	P4	Post-cholecystectomy	Post-cholecystectomy	0.6893592677345538
193	P46	P5	Post-cholecystectomy	Post-cholecystectomy	0.7416094584286804
194	P46	P9	Post-cholecystectomy	Post-cholecystectomy	0.8213196033562167
195	P46	P13	Post-cholecystectomy	Post-cholecystectomy	0.8657513348588863
196	P46	P15	Post-cholecystectomy	Post-cholecystectomy	0.709954233409611
197	P46	P16	Post-cholecystectomy	Post-cholecystectomy	0.6941266209000763

198	P46	P17	Post-cholecystectomy	Post-cholecystectomy	0.8375286041189931
199	P46	P20	Post-cholecystectomy	Post-cholecystectomy	0.7332189168573608
200	P46	P21	Post-cholecystectomy	Post-cholecystectomy	0.7763157894736842
201	P46	P24	Post-cholecystectomy	Post-cholecystectomy	0.8947368421052632
202	P46	P26	Post-cholecystectomy	Post-cholecystectomy	0.7446605644546148
203	P46	P30	Post-cholecystectomy	Post-cholecystectomy	0.9361174675819984
204	P46	P33	Post-cholecystectomy	Post-cholecystectomy	0.7200610221205187
205	P46	P35	Post-cholecystectomy	Post-cholecystectomy	0.7894736842105263
206	P46	P38	Post-cholecystectomy	Post-cholecystectomy	0.958047292143402
207	P46	P39	Post-cholecystectomy	Post-cholecystectomy	0.799771167048055
208	P46	P42	Post-cholecystectomy	Post-cholecystectomy	0.8457284515636918
209	P46	P43	Post-cholecystectomy	Post-cholecystectomy	0.7581998474446987
210	P47	P1	Post-cholecystectomy	Post-cholecystectomy	0.9466056445461479
211	P47	P2	Post-cholecystectomy	Post-cholecystectomy	0.9040808543096872
212	P47	P4	Post-cholecystectomy	Post-cholecystectomy	0.9239130434782609
213	P47	P5	Post-cholecystectomy	Post-cholecystectomy	0.9246758199847445
214	P47	P9	Post-cholecystectomy	Post-cholecystectomy	0.9296338672768879
215	P47	P13	Post-cholecystectomy	Post-cholecystectomy	0.9225781845919145
216	P47	P15	Post-cholecystectomy	Post-cholecystectomy	0.8096872616323417
217	P47	P16	Post-cholecystectomy	Post-cholecystectomy	0.9498474446987033
218	P47	P17	Post-cholecystectomy	Post-cholecystectomy	0.9458428680396643
219	P47	P20	Post-cholecystectomy	Post-cholecystectomy	0.877765064836003
220	P47	P21	Post-cholecystectomy	Post-cholecystectomy	0.9326849733028223
221	P47	P24	Post-cholecystectomy	Post-cholecystectomy	0.9181922196796338
222	P47	P26	Post-cholecystectomy	Post-cholecystectomy	0.9454614797864226
223	P47	P30	Post-cholecystectomy	Post-cholecystectomy	0.9509916094584286
224	P47	P33	Post-cholecystectomy	Post-cholecystectomy	0.9168573607932876

225	P47	P35	Post-cholecystectomy	Post-cholecystectomy
0.9359267734553776
226	P47	P38	Post-cholecystectomy	Post-cholecystectomy
0.9488939740655988
227	P47	P39	Post-cholecystectomy	Post-cholecystectomy
0.933066361556064
228	P47	P42	Post-cholecystectomy	Post-cholecystectomy
0.9586193745232647
229	P47	P43	Post-cholecystectomy	Post-cholecystectomy
0.9324942791762014
230	P47	P46	Post-cholecystectomy	Post-cholecystectomy
0.7555301296720061
231	P50	P1	Post-cholecystectomy	Post-cholecystectomy
0.9431731502669718
232	P50	P2	Post-cholecystectomy	Post-cholecystectomy
0.7112890922959573
233	P50	P4	Post-cholecystectomy	Post-cholecystectomy
0.7234935163996948
234	P50	P5	Post-cholecystectomy	Post-cholecystectomy
0.6403508771929824
235	P50	P9	Post-cholecystectomy	Post-cholecystectomy
0.7116704805491991
236	P50	P13	Post-cholecystectomy	Post-cholecystectomy
0.7967200610221206
237	P50	P15	Post-cholecystectomy	Post-cholecystectomy
0.8041571319603357
238	P50	P16	Post-cholecystectomy	Post-cholecystectomy
0.6910755148741419
239	P50	P17	Post-cholecystectomy	Post-cholecystectomy
0.7835621662852784
240	P50	P20	Post-cholecystectomy	Post-cholecystectomy
0.7929061784897025
241	P50	P21	Post-cholecystectomy	Post-cholecystectomy
0.7520976353928299
242	P50	P24	Post-cholecystectomy	Post-cholecystectomy
0.8590770404271548
243	P50	P26	Post-cholecystectomy	Post-cholecystectomy
0.8064454614797865
244	P50	P30	Post-cholecystectomy	Post-cholecystectomy
0.8861556064073226
245	P50	P33	Post-cholecystectomy	Post-cholecystectomy
0.57627765064836
246	P50	P35	Post-cholecystectomy	Post-cholecystectomy
0.7559115179252479
247	P50	P38	Post-cholecystectomy	Post-cholecystectomy
0.9227688787185355
248	P50	P39	Post-cholecystectomy	Post-cholecystectomy
0.8665141113653699
249	P50	P42	Post-cholecystectomy	Post-cholecystectomy
0.8581235697940504
250	P50	P43	Post-cholecystectomy	Post-cholecystectomy
0.7965293668954996
251	P50	P46	Post-cholecystectomy	Post-cholecystectomy
0.7513348588863463

252	P50	P47	Post-cholecystectomy	Post-cholecystectomy	0.9258199847444699
253	P55	P1	Post-cholecystectomy	Post-cholecystectomy	0.9391685736079328
254	P55	P2	Post-cholecystectomy	Post-cholecystectomy	0.7414187643020596
255	P55	P4	Post-cholecystectomy	Post-cholecystectomy	0.729023646071701
256	P55	P5	Post-cholecystectomy	Post-cholecystectomy	0.6577040427154843
257	P55	P9	Post-cholecystectomy	Post-cholecystectomy	0.6844012204424104
258	P55	P13	Post-cholecystectomy	Post-cholecystectomy	0.7774599542334096
259	P55	P15	Post-cholecystectomy	Post-cholecystectomy	0.805301296720061
260	P55	P16	Post-cholecystectomy	Post-cholecystectomy	0.7654462242562929
261	P55	P17	Post-cholecystectomy	Post-cholecystectomy	0.7896643783371472
262	P55	P20	Post-cholecystectomy	Post-cholecystectomy	0.7835621662852784
263	P55	P21	Post-cholecystectomy	Post-cholecystectomy	0.7915713196033562
264	P55	P24	Post-cholecystectomy	Post-cholecystectomy	0.7715484363081617
265	P55	P26	Post-cholecystectomy	Post-cholecystectomy	0.7553394355453852
266	P55	P30	Post-cholecystectomy	Post-cholecystectomy	0.8436308161708619
267	P55	P33	Post-cholecystectomy	Post-cholecystectomy	0.7425629290617849
268	P55	P35	Post-cholecystectomy	Post-cholecystectomy	0.7520976353928299
269	P55	P38	Post-cholecystectomy	Post-cholecystectomy	0.9610983981693364
270	P55	P39	Post-cholecystectomy	Post-cholecystectomy	0.8661327231121282
271	P55	P42	Post-cholecystectomy	Post-cholecystectomy	0.799580472921434
272	P55	P43	Post-cholecystectomy	Post-cholecystectomy	0.7427536231884058
273	P55	P46	Post-cholecystectomy	Post-cholecystectomy	0.7944317315026698
274	P55	P47	Post-cholecystectomy	Post-cholecystectomy	0.9532799389778794
275	P55	P50	Post-cholecystectomy	Post-cholecystectomy	0.6617086193745233
276	P58	P1	Post-cholecystectomy	Post-cholecystectomy	0.9624332570556827
277	P58	P2	Post-cholecystectomy	Post-cholecystectomy	0.8102593440122045
278	P58	P4	Post-cholecystectomy	Post-cholecystectomy	0.7008009153318078

279	P58	P5	Post-cholecystectomy	Post-cholecystectomy	0.7730739893211289
280	P58	P9	Post-cholecystectomy	Post-cholecystectomy	0.6022120518688024
281	P58	P13	Post-cholecystectomy	Post-cholecystectomy	0.8962623951182304
282	P58	P15	Post-cholecystectomy	Post-cholecystectomy	0.8607932875667429
283	P58	P16	Post-cholecystectomy	Post-cholecystectomy	0.763348588863463
284	P58	P17	Post-cholecystectomy	Post-cholecystectomy	0.8398169336384439
285	P58	P20	Post-cholecystectomy	Post-cholecystectomy	0.776697177726926
286	P58	P21	Post-cholecystectomy	Post-cholecystectomy	0.6859267734553776
287	P58	P24	Post-cholecystectomy	Post-cholecystectomy	0.8886346300533944
288	P58	P26	Post-cholecystectomy	Post-cholecystectomy	0.6127002288329519
289	P58	P30	Post-cholecystectomy	Post-cholecystectomy	0.9300152555301296
290	P58	P33	Post-cholecystectomy	Post-cholecystectomy	0.78813882532418
291	P58	P35	Post-cholecystectomy	Post-cholecystectomy	0.7248283752860412
292	P58	P38	Post-cholecystectomy	Post-cholecystectomy	0.9546147978642258
293	P58	P39	Post-cholecystectomy	Post-cholecystectomy	0.8323798627002288
294	P58	P42	Post-cholecystectomy	Post-cholecystectomy	0.7034706331045004
295	P58	P43	Post-cholecystectomy	Post-cholecystectomy	0.8848207475209764
296	P58	P46	Post-cholecystectomy	Post-cholecystectomy	0.7276887871853547
297	P58	P47	Post-cholecystectomy	Post-cholecystectomy	0.9195270785659801
298	P58	P50	Post-cholecystectomy	Post-cholecystectomy	0.7702135774218154
299	P58	P55	Post-cholecystectomy	Post-cholecystectomy	0.7185354691075515
300	P60	P1	Post-cholecystectomy	Post-cholecystectomy	0.9740655987795576
301	P60	P2	Post-cholecystectomy	Post-cholecystectomy	0.9385964912280702
302	P60	P4	Post-cholecystectomy	Post-cholecystectomy	0.6207093821510298
303	P60	P5	Post-cholecystectomy	Post-cholecystectomy	0.8476353928299009
304	P60	P9	Post-cholecystectomy	Post-cholecystectomy	0.8016781083142639
305	P60	P13	Post-cholecystectomy	Post-cholecystectomy	0.9393592677345538

306	P60	P15	Post-cholecystectomy	Post-cholecystectomy	0.8632723112128147
307	P60	P16	Post-cholecystectomy	Post-cholecystectomy	0.665903890160183
308	P60	P17	Post-cholecystectomy	Post-cholecystectomy	0.9347826086956522
309	P60	P20	Post-cholecystectomy	Post-cholecystectomy	0.6580854309687262
310	P60	P21	Post-cholecystectomy	Post-cholecystectomy	0.8592677345537757
311	P60	P24	Post-cholecystectomy	Post-cholecystectomy	0.9397406559877955
312	P60	P26	Post-cholecystectomy	Post-cholecystectomy	0.8689931350114416
313	P60	P30	Post-cholecystectomy	Post-cholecystectomy	0.858886346300534
314	P60	P33	Post-cholecystectomy	Post-cholecystectomy	0.7469488939740656
315	P60	P35	Post-cholecystectomy	Post-cholecystectomy	0.9303966437833715
316	P60	P38	Post-cholecystectomy	Post-cholecystectomy	0.9338291380625476
317	P60	P39	Post-cholecystectomy	Post-cholecystectomy	0.8192219679633868
318	P60	P42	Post-cholecystectomy	Post-cholecystectomy	0.918001525553013
319	P60	P43	Post-cholecystectomy	Post-cholecystectomy	0.820747520976354
320	P60	P46	Post-cholecystectomy	Post-cholecystectomy	0.75
321	P60	P47	Post-cholecystectomy	Post-cholecystectomy	0.9546147978642258
322	P60	P50	Post-cholecystectomy	Post-cholecystectomy	0.7459954233409611
323	P60	P55	Post-cholecystectomy	Post-cholecystectomy	0.8432494279176201
324	P60	P58	Post-cholecystectomy	Post-cholecystectomy	0.7564836003051106
325	P63	P1	Post-cholecystectomy	Post-cholecystectomy	0.9588100686498856
326	P63	P2	Post-cholecystectomy	Post-cholecystectomy	0.8154080854309688
327	P63	P4	Post-cholecystectomy	Post-cholecystectomy	0.7172006102212052
328	P63	P5	Post-cholecystectomy	Post-cholecystectomy	0.8285659801678108
329	P63	P9	Post-cholecystectomy	Post-cholecystectomy	0.8367658276125095
330	P63	P13	Post-cholecystectomy	Post-cholecystectomy	0.8226544622425629
331	P63	P15	Post-cholecystectomy	Post-cholecystectomy	0.8068268497330282
332	P63	P16	Post-cholecystectomy	Post-cholecystectomy	0.8445842868039665

333	P63	P17	Post−cholecystectomy	Post−cholecystectomy	0.8068268497330282
334	P63	P20	Post−cholecystectomy	Post−cholecystectomy	0.8253241800152555
335	P63	P21	Post−cholecystectomy	Post−cholecystectomy	0.852974828375286
336	P63	P24	Post−cholecystectomy	Post−cholecystectomy	0.7856598016781083
337	P63	P26	Post−cholecystectomy	Post−cholecystectomy	0.8360030511060259
338	P63	P30	Post−cholecystectomy	Post−cholecystectomy	0.9006483600305111
339	P63	P33	Post−cholecystectomy	Post−cholecystectomy	0.7749809305873379
340	P63	P35	Post−cholecystectomy	Post−cholecystectomy	0.8165522501906941
341	P63	P38	Post−cholecystectomy	Post−cholecystectomy	0.9460335621662853
342	P63	P39	Post−cholecystectomy	Post−cholecystectomy	0.9109458428680397
343	P63	P42	Post−cholecystectomy	Post−cholecystectomy	0.8892067124332571
344	P63	P43	Post−cholecystectomy	Post−cholecystectomy	0.8552631578947368
345	P63	P46	Post−cholecystectomy	Post−cholecystectomy	0.776697177726926
346	P63	P47	Post−cholecystectomy	Post−cholecystectomy	0.9256292906178489
347	P63	P50	Post−cholecystectomy	Post−cholecystectomy	0.7307398932112891
348	P63	P55	Post−cholecystectomy	Post−cholecystectomy	0.7940503432494279
349	P63	P58	Post−cholecystectomy	Post−cholecystectomy	0.7969107551487414
350	P63	P60	Post−cholecystectomy	Post−cholecystectomy	0.9002669717772692
351	P65	P1	Post−cholecystectomy	Post−cholecystectomy	0.971205186880244
352	P65	P2	Post−cholecystectomy	Post−cholecystectomy	0.8135011441647597
353	P65	P4	Post−cholecystectomy	Post−cholecystectomy	0.7601067887109078
354	P65	P5	Post−cholecystectomy	Post−cholecystectomy	0.7608695652173914
355	P65	P9	Post−cholecystectomy	Post−cholecystectomy	0.7723112128146453
356	P65	P13	Post−cholecystectomy	Post−cholecystectomy	0.8804347826086957
357	P65	P15	Post−cholecystectomy	Post−cholecystectomy	0.8693745232646835
358	P65	P16	Post−cholecystectomy	Post−cholecystectomy	0.8136918382913806
359	P65	P17	Post−cholecystectomy	Post−cholecystectomy	0.8228451563691839

360	P65	P20	Post-cholecystectomy	Post-cholecystectomy	0.8411517925247902
361	P65	P21	Post-cholecystectomy	Post-cholecystectomy	0.7721205186880244
362	P65	P24	Post-cholecystectomy	Post-cholecystectomy	0.8668954996186118
363	P65	P26	Post-cholecystectomy	Post-cholecystectomy	0.736651411136537
364	P65	P30	Post-cholecystectomy	Post-cholecystectomy	0.9429824561403509
365	P65	P33	Post-cholecystectomy	Post-cholecystectomy	0.7581998474446987
366	P65	P35	Post-cholecystectomy	Post-cholecystectomy	0.7027078565980168
367	P65	P38	Post-cholecystectomy	Post-cholecystectomy	0.9574752097635393
368	P65	P39	Post-cholecystectomy	Post-cholecystectomy	0.8863463005339436
369	P65	P42	Post-cholecystectomy	Post-cholecystectomy	0.7988176964149504
370	P65	P43	Post-cholecystectomy	Post-cholecystectomy	0.8211289092295957
371	P65	P46	Post-cholecystectomy	Post-cholecystectomy	0.7698321891685737
372	P65	P47	Post-cholecystectomy	Post-cholecystectomy	0.9427917620137299
373	P65	P50	Post-cholecystectomy	Post-cholecystectomy	0.7397025171624714
374	P65	P55	Post-cholecystectomy	Post-cholecystectomy	0.7265446224256293
375	P65	P58	Post-cholecystectomy	Post-cholecystectomy	0.7438977879481312
376	P65	P60	Post-cholecystectomy	Post-cholecystectomy	0.9385964912280702
377	P65	P63	Post-cholecystectomy	Post-cholecystectomy	0.8098779557589626
378	P68	P1	Post-cholecystectomy	Post-cholecystectomy	0.9691075514874142
379	P68	P2	Post-cholecystectomy	Post-cholecystectomy	0.8268497330282227
380	P68	P4	Post-cholecystectomy	Post-cholecystectomy	0.877765064836003
381	P68	P5	Post-cholecystectomy	Post-cholecystectomy	0.7414187643020596
382	P68	P9	Post-cholecystectomy	Post-cholecystectomy	0.6712433257055682
383	P68	P13	Post-cholecystectomy	Post-cholecystectomy	0.8960717009916095
384	P68	P15	Post-cholecystectomy	Post-cholecystectomy	0.879862700228833
385	P68	P16	Post-cholecystectomy	Post-cholecystectomy	0.8308543096872616
386	P68	P17	Post-cholecystectomy	Post-cholecystectomy	0.8729977116704806

387	P68	P20	Post-cholecystectomy	Post-cholecystectomy	0.7269260106788711
388	P68	P21	Post-cholecystectomy	Post-cholecystectomy	0.7852784134248665
389	P68	P24	Post-cholecystectomy	Post-cholecystectomy	0.8386727688787186
390	P68	P26	Post-cholecystectomy	Post-cholecystectomy	0.6800152555301296
391	P68	P30	Post-cholecystectomy	Post-cholecystectomy	0.9164759725400458
392	P68	P33	Post-cholecystectomy	Post-cholecystectomy	0.7700228832951945
393	P68	P35	Post-cholecystectomy	Post-cholecystectomy	0.772883295194508
394	P68	P38	Post-cholecystectomy	Post-cholecystectomy	0.9431731502669718
395	P68	P39	Post-cholecystectomy	Post-cholecystectomy	0.8884439359267735
396	P68	P42	Post-cholecystectomy	Post-cholecystectomy	0.6024027459954233
397	P68	P43	Post-cholecystectomy	Post-cholecystectomy	0.8775743707093822
398	P68	P46	Post-cholecystectomy	Post-cholecystectomy	0.8361937452326468
399	P68	P47	Post-cholecystectomy	Post-cholecystectomy	0.9035087719298246
400	P68	P50	Post-cholecystectomy	Post-cholecystectomy	0.7242562929061785
401	P68	P55	Post-cholecystectomy	Post-cholecystectomy	0.7246376811594203
402	P68	P58	Post-cholecystectomy	Post-cholecystectomy	0.6342486651411137
403	P68	P60	Post-cholecystectomy	Post-cholecystectomy	0.8756674294431731
404	P68	P63	Post-cholecystectomy	Post-cholecystectomy	0.7755530129672006
405	P68	P65	Post-cholecystectomy	Post-cholecystectomy	0.7254004576659039
406	P70	P1	Post-cholecystectomy	Post-cholecystectomy	0.9569031273836766
407	P70	P2	Post-cholecystectomy	Post-cholecystectomy	0.8163615560640732
408	P70	P4	Post-cholecystectomy	Post-cholecystectomy	0.9080854309687262
409	P70	P5	Post-cholecystectomy	Post-cholecystectomy	0.8176964149504196
410	P70	P9	Post-cholecystectomy	Post-cholecystectomy	0.7166285278413425
411	P70	P13	Post-cholecystectomy	Post-cholecystectomy	0.8720442410373761
412	P70	P15	Post-cholecystectomy	Post-cholecystectomy	0.872234935163997
413	P70	P16	Post-cholecystectomy	Post-cholecystectomy	0.8651792524790236

414	P70	P17	Post-cholecystectomy	Post-cholecystectomy	0.8699466056445462
415	P70	P20	Post-cholecystectomy	Post-cholecystectomy	0.8482074752097636
416	P70	P21	Post-cholecystectomy	Post-cholecystectomy	0.7774599542334096
417	P70	P24	Post-cholecystectomy	Post-cholecystectomy	0.9458428680396643
418	P70	P26	Post-cholecystectomy	Post-cholecystectomy	0.6380625476735317
419	P70	P30	Post-cholecystectomy	Post-cholecystectomy	0.9626239511823036
420	P70	P33	Post-cholecystectomy	Post-cholecystectomy	0.8184591914569032
421	P70	P35	Post-cholecystectomy	Post-cholecystectomy	0.7456140350877193
422	P70	P38	Post-cholecystectomy	Post-cholecystectomy	0.9637681159420289
423	P70	P39	Post-cholecystectomy	Post-cholecystectomy	0.8796720061022121
424	P70	P42	Post-cholecystectomy	Post-cholecystectomy	0.7454233409610984
425	P70	P43	Post-cholecystectomy	Post-cholecystectomy	0.8520213577421816
426	P70	P46	Post-cholecystectomy	Post-cholecystectomy	0.8281845919145691
427	P70	P47	Post-cholecystectomy	Post-cholecystectomy	0.9374523264683448
428	P70	P50	Post-cholecystectomy	Post-cholecystectomy	0.7301678108314263
429	P70	P55	Post-cholecystectomy	Post-cholecystectomy	0.732837528604119
430	P70	P58	Post-cholecystectomy	Post-cholecystectomy	0.7456140350877193
431	P70	P60	Post-cholecystectomy	Post-cholecystectomy	0.9496567505720824
432	P70	P63	Post-cholecystectomy	Post-cholecystectomy	0.5997330282227308
433	P70	P65	Post-cholecystectomy	Post-cholecystectomy	0.7492372234935164
434	P70	P68	Post-cholecystectomy	Post-cholecystectomy	0.618421052631579
435	P71	P1	Post-cholecystectomy	Post-cholecystectomy	0.9006483600305111
436	P71	P2	Post-cholecystectomy	Post-cholecystectomy	0.8266590389016019
437	P71	P4	Post-cholecystectomy	Post-cholecystectomy	0.8895881006864989
438	P71	P5	Post-cholecystectomy	Post-cholecystectomy	0.8043478260869565
439	P71	P9	Post-cholecystectomy	Post-cholecystectomy	0.8951182303585049
440	P71	P13	Post-cholecystectomy	Post-cholecystectomy	0.8609839816933639

441	P71	P15	Post-cholecystectomy	Post-cholecystectomy	0.7477116704805492
442	P71	P16	Post-cholecystectomy	Post-cholecystectomy	0.8770022883295194
443	P71	P17	Post-cholecystectomy	Post-cholecystectomy	0.7988176964149504
444	P71	P20	Post-cholecystectomy	Post-cholecystectomy	0.8665141113653699
445	P71	P21	Post-cholecystectomy	Post-cholecystectomy	0.8005339435545386
446	P71	P24	Post-cholecystectomy	Post-cholecystectomy	0.894927536231884
447	P71	P26	Post-cholecystectomy	Post-cholecystectomy	0.8554538520213577
448	P71	P30	Post-cholecystectomy	Post-cholecystectomy	0.9414569031273837
449	P71	P33	Post-cholecystectomy	Post-cholecystectomy	0.7780320366132724
450	P71	P35	Post-cholecystectomy	Post-cholecystectomy	0.7908085430968727
451	P71	P38	Post-cholecystectomy	Post-cholecystectomy	0.9616704805491991
452	P71	P39	Post-cholecystectomy	Post-cholecystectomy	0.9090389016018307
453	P71	P42	Post-cholecystectomy	Post-cholecystectomy	0.8274218154080855
454	P71	P43	Post-cholecystectomy	Post-cholecystectomy	0.8625095347063311
455	P71	P46	Post-cholecystectomy	Post-cholecystectomy	0.7187261632341724
456	P71	P47	Post-cholecystectomy	Post-cholecystectomy	0.7951945080091534
457	P71	P50	Post-cholecystectomy	Post-cholecystectomy	0.799771167048055
458	P71	P55	Post-cholecystectomy	Post-cholecystectomy	0.8613653699466056
459	P71	P58	Post-cholecystectomy	Post-cholecystectomy	0.88558352402746
460	P71	P60	Post-cholecystectomy	Post-cholecystectomy	0.9557589626239512
461	P71	P63	Post-cholecystectomy	Post-cholecystectomy	0.9033180778032036
462	P71	P65	Post-cholecystectomy	Post-cholecystectomy	0.8627002288329519
463	P71	P68	Post-cholecystectomy	Post-cholecystectomy	0.8731884057971014
464	P71	P70	Post-cholecystectomy	Post-cholecystectomy	0.8314263920671243
465	P74	P1	Post-cholecystectomy	Post-cholecystectomy	0.9282990083905416
466	P74	P2	Post-cholecystectomy	Post-cholecystectomy	0.7486651411136537
467	P74	P4	Post-cholecystectomy	Post-cholecystectomy	0.791952707856598

468	P74	P5	Post-cholecystectomy	Post-cholecystectomy	0.7265446224256293
469	P74	P9	Post-cholecystectomy	Post-cholecystectomy	0.8348588863463006
470	P74	P13	Post-cholecystectomy	Post-cholecystectomy	0.7669717772692601
471	P74	P15	Post-cholecystectomy	Post-cholecystectomy	0.8295194508009154
472	P74	P16	Post-cholecystectomy	Post-cholecystectomy	0.8270404271548436
473	P74	P17	Post-cholecystectomy	Post-cholecystectomy	0.8011060259344012
474	P74	P20	Post-cholecystectomy	Post-cholecystectomy	0.7971014492753623
475	P74	P21	Post-cholecystectomy	Post-cholecystectomy	0.7219679633867276
476	P74	P24	Post-cholecystectomy	Post-cholecystectomy	0.7845156369183829
477	P74	P26	Post-cholecystectomy	Post-cholecystectomy	0.717391304347826
478	P74	P30	Post-cholecystectomy	Post-cholecystectomy	0.950228832951945
479	P74	P33	Post-cholecystectomy	Post-cholecystectomy	0.7250190694126621
480	P74	P35	Post-cholecystectomy	Post-cholecystectomy	0.7875667429443173
481	P74	P38	Post-cholecystectomy	Post-cholecystectomy	0.9483218916857361
482	P74	P39	Post-cholecystectomy	Post-cholecystectomy	0.8880625476735317
483	P74	P42	Post-cholecystectomy	Post-cholecystectomy	0.8880625476735317
484	P74	P43	Post-cholecystectomy	Post-cholecystectomy	0.709954233409611
485	P74	P46	Post-cholecystectomy	Post-cholecystectomy	0.8026315789473685
486	P74	P47	Post-cholecystectomy	Post-cholecystectomy	0.8859649122807017
487	P74	P50	Post-cholecystectomy	Post-cholecystectomy	0.7702135774218154
488	P74	P55	Post-cholecystectomy	Post-cholecystectomy	0.7845156369183829
489	P74	P58	Post-cholecystectomy	Post-cholecystectomy	0.7961479786422578
490	P74	P60	Post-cholecystectomy	Post-cholecystectomy	0.8979786422578184
491	P74	P63	Post-cholecystectomy	Post-cholecystectomy	0.8022501906941266
492	P74	P65	Post-cholecystectomy	Post-cholecystectomy	0.7532418001525553
493	P74	P68	Post-cholecystectomy	Post-cholecystectomy	0.8056826849733029
494	P74	P70	Post-cholecystectomy	Post-cholecystectomy	0.8018688024408849

495	P74	P71	Post−cholecystectomy	Post−cholecystectomy	0.82627765064836
496	P75	P1	Post−cholecystectomy	Post−cholecystectomy	0.8865369946605645
497	P75	P2	Post−cholecystectomy	Post−cholecystectomy	0.7587719298245614
498	P75	P4	Post−cholecystectomy	Post−cholecystectomy	0.7620137299771167
499	P75	P5	Post−cholecystectomy	Post−cholecystectomy	0.6990846681922197
500	P75	P9	Post−cholecystectomy	Post−cholecystectomy	0.7725019069412662
501	P75	P13	Post−cholecystectomy	Post−cholecystectomy	0.7623951182303585
502	P75	P15	Post−cholecystectomy	Post−cholecystectomy	0.7559115179252479
503	P75	P16	Post−cholecystectomy	Post−cholecystectomy	0.8308543096872616
504	P75	P17	Post−cholecystectomy	Post−cholecystectomy	0.7984363081617086
505	P75	P20	Post−cholecystectomy	Post−cholecystectomy	0.7088100686498856
506	P75	P21	Post−cholecystectomy	Post−cholecystectomy	0.7562929061784897
507	P75	P24	Post−cholecystectomy	Post−cholecystectomy	0.7124332570556827
508	P75	P26	Post−cholecystectomy	Post−cholecystectomy	0.8789092295957285
509	P75	P30	Post−cholecystectomy	Post−cholecystectomy	0.881578947368421
510	P75	P33	Post−cholecystectomy	Post−cholecystectomy	0.7715484363081617
511	P75	P35	Post−cholecystectomy	Post−cholecystectomy	0.8072082379862701
512	P75	P38	Post−cholecystectomy	Post−cholecystectomy	0.9401220442410374
513	P75	P39	Post−cholecystectomy	Post−cholecystectomy	0.7816552250190694
514	P75	P42	Post−cholecystectomy	Post−cholecystectomy	0.9288710907704043
515	P75	P43	Post−cholecystectomy	Post−cholecystectomy	0.7726926010678871
516	P75	P46	Post−cholecystectomy	Post−cholecystectomy	0.7839435545385202
517	P75	P47	Post−cholecystectomy	Post−cholecystectomy	0.9366895499618612
518	P75	P50	Post−cholecystectomy	Post−cholecystectomy	0.6746758199847445
519	P75	P55	Post−cholecystectomy	Post−cholecystectomy	0.7170099160945843
520	P75	P58	Post−cholecystectomy	Post−cholecystectomy	0.7801296720061022
521	P75	P60	Post−cholecystectomy	Post−cholecystectomy	0.852212051868802 4

522	P75	P63	Post-cholecystectomy	Post-cholecystectomy	0.7257818459191457
523	P75	P65	Post-cholecystectomy	Post-cholecystectomy	0.8106407322654462
524	P75	P68	Post-cholecystectomy	Post-cholecystectomy	0.7963386727688787
525	P75	P70	Post-cholecystectomy	Post-cholecystectomy	0.8428680396643783
526	P75	P71	Post-cholecystectomy	Post-cholecystectomy	0.6649504195270786
527	P75	P74	Post-cholecystectomy	Post-cholecystectomy	0.7942410373760488
528	P1	B27	Post-cholecystectomy	Control group	0.9475591151792525
529	P1	B66	Post-cholecystectomy	Control group	0.931350114416476
530	P1	B86	Post-cholecystectomy	Control group	0.9506102212051869
531	P1	B97	Post-cholecystectomy	Control group	0.9424103737604882
532	P1	B98	Post-cholecystectomy	Control group	0.9492753623188406
533	P1	B100	Post-cholecystectomy	Control group	0.9345919145690312
534	P1	B112	Post-cholecystectomy	Control group	0.9658657513348589
535	P1	B115	Post-cholecystectomy	Control group	0.9244851258581236
536	P1	I1	Post-cholecystectomy	Control group	0.944698703279939
537	P1	I3	Post-cholecystectomy	Control group	0.9128527841342486
538	P1	I6	Post-cholecystectomy	Control group	0.9548054919908466
539	P1	I8	Post-cholecystectomy	Control group	0.9454614797864226
540	P1	I10	Post-cholecystectomy	Control group	0.9450800915331807
541	P1	I11	Post-cholecystectomy	Control group	0.9366895499618612
542	P1	I13	Post-cholecystectomy	Control group	0.9479405034324943
543	P1	I15	Post-cholecystectomy	Control group	0.8625095347063311
544	P1	I17	Post-cholecystectomy	Control group	0.9750190694126621
545	P1	I18	Post-cholecystectomy	Control group	0.8184591914569032
546	P1	I19	Post-cholecystectomy	Control group	0.9273455377574371
547	P1	I22	Post-cholecystectomy	Control group	0.8367658276125095
548	P1	I23	Post-cholecystectomy	Control group	0.9193363844393593

549	P1	I24	Post−cholecystectomy	Control group
0.9774980930587338
550	P1	I25	Post−cholecystectomy	Control group
0.9302059496567505
551	P1	I26	Post−cholecystectomy	Control group
0.950419527078566
552	P1	I27	Post−cholecystectomy	Control group
0.8857742181540809
553	P1	I28	Post−cholecystectomy	Control group
0.8476353928299009
554	P1	I29	Post−cholecystectomy	Control group
0.9326849733028223
555	P1	I30	Post−cholecystectomy	Control group
0.9685354691075515
556	P1	I31	Post−cholecystectomy	Control group
0.9698703279938978
557	P1	I32	Post−cholecystectomy	Control group
0.9530892448512586
558	P1	I33	Post−cholecystectomy	Control group
0.9649122807017544
559	P1	I34	Post−cholecystectomy	Control group
0.9624332570556827
560	P1	I35	Post−cholecystectomy	Control group
0.8773836765827613
561	P1	I36	Post−cholecystectomy	Control group
0.8628909229595728
562	P1	I37	Post−cholecystectomy	Control group
0.8062547673531655
563	P2	B27	Post−cholecystectomy	Control group
0.8171243325705568
564	P2	B66	Post−cholecystectomy	Control group
0.7745995423340961
565	P2	B86	Post−cholecystectomy	Control group
0.7856598016781083
566	P2	B97	Post−cholecystectomy	Control group
0.729023646071701
567	P2	B98	Post−cholecystectomy	Control group
0.8049199084668193
568	P2	B100	Post−cholecystectomy	Control group
0.9269641495041953
569	P2	B112	Post−cholecystectomy	Control group
0.8209382151029748
570	P2	B115	Post−cholecystectomy	Control group
0.7841342486651411
571	P2	I1	Post−cholecystectomy	Control group
0.7170099160945843
572	P2	I3	Post−cholecystectomy	Control group
0.7913806254767353
573	P2	I6	Post−cholecystectomy	Control group
0.738367658276125
574	P2	I8	Post−cholecystectomy	Control group
0.7318840579710145
575	P2	I10	Post−cholecystectomy	Control group
0.7862318840579711

576	P2	I11	Post-cholecystectomy	Control group	0.8832951945080092
577	P2	I13	Post-cholecystectomy	Control group	0.7463768115942029
578	P2	I15	Post-cholecystectomy	Control group	0.8352402745995423
579	P2	I17	Post-cholecystectomy	Control group	0.7768878718535469
580	P2	I18	Post-cholecystectomy	Control group	0.7330282227307399
581	P2	I19	Post-cholecystectomy	Control group	0.791952707856598
582	P2	I22	Post-cholecystectomy	Control group	0.7337909992372235
583	P2	I23	Post-cholecystectomy	Control group	0.6912662090007627
584	P2	I24	Post-cholecystectomy	Control group	0.8060640732265446
585	P2	I25	Post-cholecystectomy	Control group	0.7677345537757437
586	P2	I26	Post-cholecystectomy	Control group	0.7471395881006865
587	P2	I27	Post-cholecystectomy	Control group	0.7602974828375286
588	P2	I28	Post-cholecystectomy	Control group	0.916094584286804
589	P2	I29	Post-cholecystectomy	Control group	0.8314263920671243
590	P2	I30	Post-cholecystectomy	Control group	0.8522120518688024
591	P2	I31	Post-cholecystectomy	Control group	0.8525934401220442
592	P2	I32	Post-cholecystectomy	Control group	0.914187643020595
593	P2	I33	Post-cholecystectomy	Control group	0.7757437070938215
594	P2	I34	Post-cholecystectomy	Control group	0.7925247902364607
595	P2	I35	Post-cholecystectomy	Control group	0.7894736842105263
596	P2	I36	Post-cholecystectomy	Control group	0.9639588100686499
597	P2	I37	Post-cholecystectomy	Control group	0.8758581235697941
598	P4	B27	Post-cholecystectomy	Control group	0.7963386727688787
599	P4	B66	Post-cholecystectomy	Control group	0.761632341723875
600	P4	B86	Post-cholecystectomy	Control group	0.8125476735316552
601	P4	B97	Post-cholecystectomy	Control group	0.7315026697177727
602	P4	B98	Post-cholecystectomy	Control group	0.6876430205949656

603	P4	B100	Post-cholecystectomy	Control group	0.9256292906178489
604	P4	B112	Post-cholecystectomy	Control group	0.8834858886346301
605	P4	B115	Post-cholecystectomy	Control group	0.7587719298245614
606	P4	I1	Post-cholecystectomy	Control group	0.7431350114416476
607	P4	I3	Post-cholecystectomy	Control group	0.7562929061784897
608	P4	I6	Post-cholecystectomy	Control group	0.8188405797101449
609	P4	I8	Post-cholecystectomy	Control group	0.8836765827612509
610	P4	I10	Post-cholecystectomy	Control group	0.8047292143401983
611	P4	I11	Post-cholecystectomy	Control group	0.82627765064836
612	P4	I13	Post-cholecystectomy	Control group	0.8569794050343249
613	P4	I15	Post-cholecystectomy	Control group	0.8285659801678108
614	P4	I17	Post-cholecystectomy	Control group	0.8482074752097636
615	P4	I18	Post-cholecystectomy	Control group	0.799771167048055
616	P4	I19	Post-cholecystectomy	Control group	0.9496567505720824
617	P4	I22	Post-cholecystectomy	Control group	0.8579328756674295
618	P4	I23	Post-cholecystectomy	Control group	0.7351258581235698
619	P4	I24	Post-cholecystectomy	Control group	0.8865369946605645
620	P4	I25	Post-cholecystectomy	Control group	0.8094965675057209
621	P4	I26	Post-cholecystectomy	Control group	0.839626239511823
622	P4	I27	Post-cholecystectomy	Control group	0.7793668954996186
623	P4	I28	Post-cholecystectomy	Control group	0.7479023646071701
624	P4	I29	Post-cholecystectomy	Control group	0.8712814645308925
625	P4	I30	Post-cholecystectomy	Control group	0.8043478260869565
626	P4	I31	Post-cholecystectomy	Control group	0.8581235697940504
627	P4	I32	Post-cholecystectomy	Control group	0.8531655225019069
628	P4	I33	Post-cholecystectomy	Control group	0.8571700991609459
629	P4	I34	Post-cholecystectomy	Control group	0.8962623951182304

630	P4	I35	Post-cholecystectomy	Control group	0.7393211289092296
631	P4	I36	Post-cholecystectomy	Control group	0.9891304347826086
632	P4	I37	Post-cholecystectomy	Control group	0.8371472158657514
633	P5	B27	Post-cholecystectomy	Control group	0.704042715484363
634	P5	B66	Post-cholecystectomy	Control group	0.7818459191456903
635	P5	B86	Post-cholecystectomy	Control group	0.7725019069412662
636	P5	B97	Post-cholecystectomy	Control group	0.6977498093058734
637	P5	B98	Post-cholecystectomy	Control group	0.698512585812357
638	P5	B100	Post-cholecystectomy	Control group	0.9433638443935927
639	P5	B112	Post-cholecystectomy	Control group	0.8083524027459954
640	P5	B115	Post-cholecystectomy	Control group	0.6931731502669718
641	P5	I1	Post-cholecystectomy	Control group	0.6674294431731502
642	P5	I3	Post-cholecystectomy	Control group	0.725209763539283
643	P5	I6	Post-cholecystectomy	Control group	0.6584668192219679
644	P5	I8	Post-cholecystectomy	Control group	0.7665903890160183
645	P5	I10	Post-cholecystectomy	Control group	0.7940503432494279
646	P5	I11	Post-cholecystectomy	Control group	0.8623188405797102
647	P5	I13	Post-cholecystectomy	Control group	0.7177726926010679
648	P5	I15	Post-cholecystectomy	Control group	0.8278032036613272
649	P5	I17	Post-cholecystectomy	Control group	0.7667810831426392
650	P5	I18	Post-cholecystectomy	Control group	0.7297864225781846
651	P5	I19	Post-cholecystectomy	Control group	0.8154080854309688
652	P5	I22	Post-cholecystectomy	Control group	0.7807017543859649
653	P5	I23	Post-cholecystectomy	Control group	0.652745995423341
654	P5	I24	Post-cholecystectomy	Control group	0.8365751334858886
655	P5	I25	Post-cholecystectomy	Control group	0.7210144927536232
656	P5	I26	Post-cholecystectomy	Control group	0.7006102212051869

657	P5	I27	Post-cholecystectomy	Control group
0.719488939740656
658	P5	I28	Post-cholecystectomy	Control group
0.8823417238749046
659	P5	I29	Post-cholecystectomy	Control group
0.8407704042715485
660	P5	I30	Post-cholecystectomy	Control group
0.7585812356979404
661	P5	I31	Post-cholecystectomy	Control group
0.8228451563691839
662	P5	I32	Post-cholecystectomy	Control group
0.8453470633104501
663	P5	I33	Post-cholecystectomy	Control group
0.8159801678108314
664	P5	I34	Post-cholecystectomy	Control group
0.7673531655225019
665	P5	I35	Post-cholecystectomy	Control group
0.8127383676582761
666	P5	I36	Post-cholecystectomy	Control group
0.9959954233409611
667	P5	I37	Post-cholecystectomy	Control group
0.8535469107551488
668	P9	B27	Post-cholecystectomy	Control group
0.8163615560640732
669	P9	B66	Post-cholecystectomy	Control group
0.8001525553012967
670	P9	B86	Post-cholecystectomy	Control group
0.8688024408848207
671	P9	B97	Post-cholecystectomy	Control group
0.8487795575896262
672	P9	B98	Post-cholecystectomy	Control group
0.7707856598016781
673	P9	B100	Post-cholecystectomy	Control group
0.8848207475209764
674	P9	B112	Post-cholecystectomy	Control group
0.9124713958810069
675	P9	B115	Post-cholecystectomy	Control group
0.6657131960335622
676	P9	I1	Post-cholecystectomy	Control group
0.7799389778794813
677	P9	I3	Post-cholecystectomy	Control group
0.8318077803203662
678	P9	I6	Post-cholecystectomy	Control group
0.6931731502669718
679	P9	I8	Post-cholecystectomy	Control group
0.9052250190694127
680	P9	I10	Post-cholecystectomy	Control group
0.8729977116704806
681	P9	I11	Post-cholecystectomy	Control group
0.8810068649885584
682	P9	I13	Post-cholecystectomy	Control group
0.8562166285278413
683	P9	I15	Post-cholecystectomy	Control group
0.8447749809305873

684	P9	I17	Post-cholecystectomy	Control group	0.9374523264683448
685	P9	I18	Post-cholecystectomy	Control group	0.8030129672006102
686	P9	I19	Post-cholecystectomy	Control group	0.9347826086956522
687	P9	I22	Post-cholecystectomy	Control group	0.8623188405797102
688	P9	I23	Post-cholecystectomy	Control group	0.7986270022883295
689	P9	I24	Post-cholecystectomy	Control group	0.9096109839816934
690	P9	I25	Post-cholecystectomy	Control group	0.8348588863463006
691	P9	I26	Post-cholecystectomy	Control group	0.7839435545385202
692	P9	I27	Post-cholecystectomy	Control group	0.7242562929061785
693	P9	I28	Post-cholecystectomy	Control group	0.8901601830663616
694	P9	I29	Post-cholecystectomy	Control group	0.8035850495804729
695	P9	I30	Post-cholecystectomy	Control group	0.7295957284515637
696	P9	I31	Post-cholecystectomy	Control group	0.8993135011441648
697	P9	I32	Post-cholecystectomy	Control group	0.9302059496567505
698	P9	I33	Post-cholecystectomy	Control group	0.8775743707093822
699	P9	I34	Post-cholecystectomy	Control group	0.7898550724637681
700	P9	I35	Post-cholecystectomy	Control group	0.8735697940503433
701	P9	I36	Post-cholecystectomy	Control group	0.9908466819221968
702	P9	I37	Post-cholecystectomy	Control group	0.8686117467581999
703	P13	B27	Post-cholecystectomy	Control group	0.8720442410373761
704	P13	B66	Post-cholecystectomy	Control group	0.860602593440122
705	P13	B86	Post-cholecystectomy	Control group	0.8152173913043478
706	P13	B97	Post-cholecystectomy	Control group	0.8159801678108314
707	P13	B98	Post-cholecystectomy	Control group	0.7988176964149504
708	P13	B100	Post-cholecystectomy	Control group	0.9549961861174676
709	P13	B112	Post-cholecystectomy	Control group	0.9080854309687262
710	P13	B115	Post-cholecystectomy	Control group	0.7349351639969489

711	P13	I1	Post-cholecystectomy	Control group	0.8075896262395118
712	P13	I3	Post-cholecystectomy	Control group	0.7488558352402745
713	P13	I6	Post-cholecystectomy	Control group	0.8363844393592678
714	P13	I8	Post-cholecystectomy	Control group	0.7055682684973302
715	P13	I10	Post-cholecystectomy	Control group	0.6125095347063311
716	P13	I11	Post-cholecystectomy	Control group	0.8878718535469108
717	P13	I13	Post-cholecystectomy	Control group	0.7032799389778794
718	P13	I15	Post-cholecystectomy	Control group	0.8201754385964912
719	P13	I17	Post-cholecystectomy	Control group	0.7755530129672006
720	P13	I18	Post-cholecystectomy	Control group	0.7282608695652174
721	P13	I19	Post-cholecystectomy	Control group	0.8577421815408085
722	P13	I22	Post-cholecystectomy	Control group	0.7848970251716247
723	P13	I23	Post-cholecystectomy	Control group	0.7435163996948894
724	P13	I24	Post-cholecystectomy	Control group	0.8070175438596491
725	P13	I25	Post-cholecystectomy	Control group	0.7141495041952708
726	P13	I26	Post-cholecystectomy	Control group	0.75
727	P13	I27	Post-cholecystectomy	Control group	0.7353165522501907
728	P13	I28	Post-cholecystectomy	Control group	0.8611746758199847
729	P13	I29	Post-cholecystectomy	Control group	0.8190312738367659
730	P13	I30	Post-cholecystectomy	Control group	0.8163615560640732
731	P13	I31	Post-cholecystectomy	Control group	0.7484744469870328
732	P13	I32	Post-cholecystectomy	Control group	0.6462623951182304
733	P13	I33	Post-cholecystectomy	Control group	0.8049199084668193
734	P13	I34	Post-cholecystectomy	Control group	0.8771929824561403
735	P13	I35	Post-cholecystectomy	Control group	0.7929061784897025
736	P13	I36	Post-cholecystectomy	Control group	0.9973302822273074
737	P13	I37	Post-cholecystectomy	Control group	0.9223874904652937
738	P15	B27	Post-cholecystectomy	Control group	

0.7196796338672768
739 P15 B66 Post-cholecystectomy Control group
0.7908085430968727
740 P15 B86 Post-cholecystectomy Control group
0.7198703279938978
741 P15 B97 Post-cholecystectomy Control group
0.7528604118993135
742 P15 B98 Post-cholecystectomy Control group
0.7048054919908466
743 P15 B100 Post-cholecystectomy Control group
0.9157131960335622
744 P15 B112 Post-cholecystectomy Control group
0.6424485125858124
745 P15 B115 Post-cholecystectomy Control group
0.816742944317315
746 P15 I1 Post-cholecystectomy Control group
0.7484744469870328
747 P15 I3 Post-cholecystectomy Control group
0.7337909992372235
748 P15 I6 Post-cholecystectomy Control group
0.8096872616323417
749 P15 I8 Post-cholecystectomy Control group
0.8381006864988558
750 P15 I10 Post-cholecystectomy Control group
0.8331426392067124
751 P15 I11 Post-cholecystectomy Control group
0.8165522501906941
752 P15 I13 Post-cholecystectomy Control group
0.818649885583524
753 P15 I15 Post-cholecystectomy Control group
0.8737604881769642
754 P15 I17 Post-cholecystectomy Control group
0.7377955758962624
755 P15 I18 Post-cholecystectomy Control group
0.7967200610221206
756 P15 I19 Post-cholecystectomy Control group
0.8150266971777269
757 P15 I22 Post-cholecystectomy Control group
0.7662090007627765
758 P15 I23 Post-cholecystectomy Control group
0.7225400457665904
759 P15 I24 Post-cholecystectomy Control group
0.7974828375286042
760 P15 I25 Post-cholecystectomy Control group
0.8188405797101449
761 P15 I26 Post-cholecystectomy Control group
0.8810068649885584
762 P15 I27 Post-cholecystectomy Control group
0.8463005339435545
763 P15 I28 Post-cholecystectomy Control group
0.8127383676582761
764 P15 I29 Post-cholecystectomy Control group
0.876048817696415
765 P15 I30 Post-cholecystectomy Control group

0.8737604881769642
766	P15	I31	Post-cholecystectomy	Control group
0.8442028985507246
767	P15	I32	Post-cholecystectomy	Control group
0.8792906178489702
768	P15	I33	Post-cholecystectomy	Control group
0.7696414950419527
769	P15	I34	Post-cholecystectomy	Control group
0.7749809305873379
770	P15	I35	Post-cholecystectomy	Control group
0.679252479023646
771	P15	I36	Post-cholecystectomy	Control group
0.969488939740656
772	P15	I37	Post-cholecystectomy	Control group
0.7971014492753623
773	P16	B27	Post-cholecystectomy	Control group
0.8422959572845157
774	P16	B66	Post-cholecystectomy	Control group
0.719488939740656
775	P16	B86	Post-cholecystectomy	Control group
0.8253241800152555
776	P16	B97	Post-cholecystectomy	Control group
0.6634248665141114
777	P16	B98	Post-cholecystectomy	Control group
0.8001525553012967
778	P16	B100	Post-cholecystectomy	Control group
0.8609839816933639
779	P16	B112	Post-cholecystectomy	Control group
0.8888253241800153
780	P16	B115	Post-cholecystectomy	Control group
0.7887109077040427
781	P16	I1	Post-cholecystectomy	Control group
0.8388634630053394
782	P16	I3	Post-cholecystectomy	Control group
0.7090007627765065
783	P16	I6	Post-cholecystectomy	Control group
0.8056826849733029
784	P16	I8	Post-cholecystectomy	Control group
0.8838672768878718
785	P16	I10	Post-cholecystectomy	Control group
0.8413424866514111
786	P16	I11	Post-cholecystectomy	Control group
0.8619374523264683
787	P16	I13	Post-cholecystectomy	Control group
0.830091533180778
788	P16	I15	Post-cholecystectomy	Control group
0.698512585812357
789	P16	I17	Post-cholecystectomy	Control group
0.9012204424103738
790	P16	I18	Post-cholecystectomy	Control group
0.8098779557589626
791	P16	I19	Post-cholecystectomy	Control group
0.9221967963386728
792	P16	I22	Post-cholecystectomy	Control group

0.8649885583524027
793	P16	I23	Post-cholecystectomy	Control group
0.8089244851258581
794	P16	I24	Post-cholecystectomy	Control group
0.9418382913806255
795	P16	I25	Post-cholecystectomy	Control group
0.8243707093821511
796	P16	I26	Post-cholecystectomy	Control group
0.7953852021357742
797	P16	I27	Post-cholecystectomy	Control group
0.8287566742944318
798	P16	I28	Post-cholecystectomy	Control group
0.7433257055682685
799	P16	I29	Post-cholecystectomy	Control group
0.9187643020594966
800	P16	I30	Post-cholecystectomy	Control group
0.7869946605644547
801	P16	I31	Post-cholecystectomy	Control group
0.915903890160183
802	P16	I32	Post-cholecystectomy	Control group
0.8956903127383676
803	P16	I33	Post-cholecystectomy	Control group
0.86441647597254
804	P16	I34	Post-cholecystectomy	Control group
0.8726163234172387
805	P16	I35	Post-cholecystectomy	Control group
0.8369565217391305
806	P16	I36	Post-cholecystectomy	Control group
0.979023646071701
807	P16	I37	Post-cholecystectomy	Control group
0.8979786422578184
808	P17	B27	Post-cholecystectomy	Control group
0.8668954996186118
809	P17	B66	Post-cholecystectomy	Control group
0.8133104500381388
810	P17	B86	Post-cholecystectomy	Control group
0.7751716247139588
811	P17	B97	Post-cholecystectomy	Control group
0.7313119755911518
812	P17	B98	Post-cholecystectomy	Control group
0.7759344012204424
813	P17	B100	Post-cholecystectomy	Control group
0.9290617848970252
814	P17	B112	Post-cholecystectomy	Control group
0.8922578184591915
815	P17	B115	Post-cholecystectomy	Control group
0.7915713196033562
816	P17	I1	Post-cholecystectomy	Control group
0.8155987795575896
817	P17	I3	Post-cholecystectomy	Control group
0.8136918382913806
818	P17	I6	Post-cholecystectomy	Control group
0.7801296720061022
819	P17	I8	Post-cholecystectomy	Control group

0.8323798627002288
820 P17 I10 Post-cholecystectomy Control group
0.8077803203661327
821 P17 I11 Post-cholecystectomy Control group
0.8709000762776506
822 P17 I13 Post-cholecystectomy Control group
0.7978642257818459
823 P17 I15 Post-cholecystectomy Control group
0.8829138062547673
824 P17 I17 Post-cholecystectomy Control group
0.8421052631578947
825 P17 I18 Post-cholecystectomy Control group
0.7660183066361556
826 P17 I19 Post-cholecystectomy Control group
0.8972158657513348
827 P17 I22 Post-cholecystectomy Control group
0.7829900839054157
828 P17 I23 Post-cholecystectomy Control group
0.6529366895499619
829 P17 I24 Post-cholecystectomy Control group
0.8260869565217391
830 P17 I25 Post-cholecystectomy Control group
0.7715484363081617
831 P17 I26 Post-cholecystectomy Control group
0.8171243325705568
832 P17 I27 Post-cholecystectomy Control group
0.7644927536231884
833 P17 I28 Post-cholecystectomy Control group
0.8886346300533944
834 P17 I29 Post-cholecystectomy Control group
0.7917620137299771
835 P17 I30 Post-cholecystectomy Control group
0.8325705568268498
836 P17 I31 Post-cholecystectomy Control group
0.8049199084668193
837 P17 I32 Post-cholecystectomy Control group
0.8920671243325705
838 P17 I33 Post-cholecystectomy Control group
0.7984363081617086
839 P17 I34 Post-cholecystectomy Control group
0.8135011441647597
840 P17 I35 Post-cholecystectomy Control group
0.7827993897787948
841 P17 I36 Post-cholecystectomy Control group
0.9969488939740656
842 P17 I37 Post-cholecystectomy Control group
0.919908466819222
843 P20 B27 Post-cholecystectomy Control group
0.8560259344012204
844 P20 B66 Post-cholecystectomy Control group
0.8253241800152555
845 P20 B86 Post-cholecystectomy Control group
0.8140732265446224
846 P20 B97 Post-cholecystectomy Control group

0.8176964149504196
847	P20	B98	Post-cholecystectomy	Control group
0.7374141876430206
848	P20	B100	Post-cholecystectomy	Control group
0.9382151029748284
849	P20	B112	Post-cholecystectomy	Control group
0.9033180778032036
850	P20	B115	Post-cholecystectomy	Control group
0.8020594965675057
851	P20	I1	Post-cholecystectomy	Control group
0.7936689549961862
852	P20	I3	Post-cholecystectomy	Control group
0.6485507246376812
853	P20	I6	Post-cholecystectomy	Control group
0.7738367658276125
854	P20	I8	Post-cholecystectomy	Control group
0.8617467581998475
855	P20	I10	Post-cholecystectomy	Control group
0.7744088482074752
856	P20	I11	Post-cholecystectomy	Control group
0.778604118993135
857	P20	I13	Post-cholecystectomy	Control group
0.7765064836003052
858	P20	I15	Post-cholecystectomy	Control group
0.801487414187643
859	P20	I17	Post-cholecystectomy	Control group
0.8428680396643783
860	P20	I18	Post-cholecystectomy	Control group
0.6409229595728452
861	P20	I19	Post-cholecystectomy	Control group
0.9342105263157895
862	P20	I22	Post-cholecystectomy	Control group
0.7498093058733791
863	P20	I23	Post-cholecystectomy	Control group
0.7831807780320366
864	P20	I24	Post-cholecystectomy	Control group
0.8586956521739131
865	P20	I25	Post-cholecystectomy	Control group
0.7313119755911518
866	P20	I26	Post-cholecystectomy	Control group
0.8236079328756675
867	P20	I27	Post-cholecystectomy	Control group
0.6405415713196033
868	P20	I28	Post-cholecystectomy	Control group
0.5930587337909993
869	P20	I29	Post-cholecystectomy	Control group
0.8335240274599542
870	P20	I30	Post-cholecystectomy	Control group
0.7601067887109078
871	P20	I31	Post-cholecystectomy	Control group
0.7932875667429443
872	P20	I32	Post-cholecystectomy	Control group
0.7873760488176964
873	P20	I33	Post-cholecystectomy	Control group

0.8152173913043478
874	P20	I34	Post-cholecystectomy	Control group
0.8075896262395118
875	P20	I35	Post-cholecystectomy	Control group
0.6746758199847445
876	P20	I36	Post-cholecystectomy	Control group
0.9118993135011442
877	P20	I37	Post-cholecystectomy	Control group
0.8068268497330282
878	P21	B27	Post-cholecystectomy	Control group
0.8686117467581999
879	P21	B66	Post-cholecystectomy	Control group
0.6802059496567505
880	P21	B86	Post-cholecystectomy	Control group
0.8363844393592678
881	P21	B97	Post-cholecystectomy	Control group
0.8287566742944318
882	P21	B98	Post-cholecystectomy	Control group
0.8257055682684973
883	P21	B100	Post-cholecystectomy	Control group
0.9052250190694127
884	P21	B112	Post-cholecystectomy	Control group
0.900839054157132
885	P21	B115	Post-cholecystectomy	Control group
0.7610602593440122
886	P21	I1	Post-cholecystectomy	Control group
0.8453470633104501
887	P21	I3	Post-cholecystectomy	Control group
0.7130053394355453
888	P21	I6	Post-cholecystectomy	Control group
0.709954233409611
889	P21	I8	Post-cholecystectomy	Control group
0.8920671243325705
890	P21	I10	Post-cholecystectomy	Control group
0.7837528604118993
891	P21	I11	Post-cholecystectomy	Control group
0.8922578184591915
892	P21	I13	Post-cholecystectomy	Control group
0.8579328756674295
893	P21	I15	Post-cholecystectomy	Control group
0.7690694126620901
894	P21	I17	Post-cholecystectomy	Control group
0.9138062547673532
895	P21	I18	Post-cholecystectomy	Control group
0.7601067887109078
896	P21	I19	Post-cholecystectomy	Control group
0.9149504195270786
897	P21	I22	Post-cholecystectomy	Control group
0.8209382151029748
898	P21	I23	Post-cholecystectomy	Control group
0.7452326468344775
899	P21	I24	Post-cholecystectomy	Control group
0.8653699466056446
900	P21	I25	Post-cholecystectomy	Control group

0.7784134248665141
901 P21 I26 Post-cholecystectomy Control group
0.7008009153318078
902 P21 I27 Post-cholecystectomy Control group
0.6048817696414951
903 P21 I28 Post-cholecystectomy Control group
0.8375286041189931
904 P21 I29 Post-cholecystectomy Control group
0.8741418764302059
905 P21 I30 Post-cholecystectomy Control group
0.6334858886346301
906 P21 I31 Post-cholecystectomy Control group
0.8840579710144928
907 P21 I32 Post-cholecystectomy Control group
0.8871090770404272
908 P21 I33 Post-cholecystectomy Control group
0.8445842868039665
909 P21 I34 Post-cholecystectomy Control group
0.7656369183829138
910 P21 I35 Post-cholecystectomy Control group
0.8535469107551488
911 P21 I36 Post-cholecystectomy Control group
0.982837528604119
912 P21 I37 Post-cholecystectomy Control group
0.9328756674294432
913 P24 B27 Post-cholecystectomy Control group
0.8369565217391305
914 P24 B66 Post-cholecystectomy Control group
0.8552631578947368
915 P24 B86 Post-cholecystectomy Control group
0.8478260869565217
916 P24 B97 Post-cholecystectomy Control group
0.7028985507246377
917 P24 B98 Post-cholecystectomy Control group
0.7988176964149504
918 P24 B100 Post-cholecystectomy Control group
0.9508009153318078
919 P24 B112 Post-cholecystectomy Control group
0.8604118993135011
920 P24 B115 Post-cholecystectomy Control group
0.7311212814645309
921 P24 I1 Post-cholecystectomy Control group
0.7574370709382151
922 P24 I3 Post-cholecystectomy Control group
0.7906178489702517
923 P24 I6 Post-cholecystectomy Control group
0.7795575896262396
924 P24 I8 Post-cholecystectomy Control group
0.7679252479023646
925 P24 I10 Post-cholecystectomy Control group
0.8218916857360793
926 P24 I11 Post-cholecystectomy Control group
0.8686117467581999
927 P24 I13 Post-cholecystectomy Control group

0.7183447749809306
928	P24	I15	Post-cholecystectomy	Control group
0.9376430205949656
929	P24	I17	Post-cholecystectomy	Control group
0.7965293668954996
930	P24	I18	Post-cholecystectomy	Control group
0.7070938215102975
931	P24	I19	Post-cholecystectomy	Control group
0.8003432494279176
932	P24	I22	Post-cholecystectomy	Control group
0.78813882532418
933	P24	I23	Post-cholecystectomy	Control group
0.7421815408085431
934	P24	I24	Post-cholecystectomy	Control group
0.8556445461479787
935	P24	I25	Post-cholecystectomy	Control group
0.7570556826849733
936	P24	I26	Post-cholecystectomy	Control group
0.8562166285278413
937	P24	I27	Post-cholecystectomy	Control group
0.8096872616323417
938	P24	I28	Post-cholecystectomy	Control group
0.9244851258581236
939	P24	I29	Post-cholecystectomy	Control group
0.8163615560640732
940	P24	I30	Post-cholecystectomy	Control group
0.8787185354691075
941	P24	I31	Post-cholecystectomy	Control group
0.7896643783371472
942	P24	I32	Post-cholecystectomy	Control group
0.8287566742944318
943	P24	I33	Post-cholecystectomy	Control group
0.8625095347063311
944	P24	I34	Post-cholecystectomy	Control group
0.9044622425629291
945	P24	I35	Post-cholecystectomy	Control group
0.8033943554538521
946	P24	I36	Post-cholecystectomy	Control group
0.9937070938215103
947	P24	I37	Post-cholecystectomy	Control group
0.8838672768878718
948	P26	B27	Post-cholecystectomy	Control group
0.931350114416476
949	P26	B66	Post-cholecystectomy	Control group
0.6769641495041953
950	P26	B86	Post-cholecystectomy	Control group
0.8415331807780321
951	P26	B97	Post-cholecystectomy	Control group
0.8022501906941266
952	P26	B98	Post-cholecystectomy	Control group
0.8670861937452327
953	P26	B100	Post-cholecystectomy	Control group
0.8913043478260869
954	P26	B112	Post-cholecystectomy	Control group

0.9059877955758963
955	P26	B115	Post-cholecystectomy	Control group
0.8829138062547673
956	P26	I1	Post-cholecystectomy	Control group
0.8632723112128147
957	P26	I3	Post-cholecystectomy	Control group
0.8138825324180016
958	P26	I6	Post-cholecystectomy	Control group
0.7808924485125858
959	P26	I8	Post-cholecystectomy	Control group
0.9271548436308161
960	P26	I10	Post-cholecystectomy	Control group
0.8211289092295957
961	P26	I11	Post-cholecystectomy	Control group
0.9795957284515637
962	P26	I13	Post-cholecystectomy	Control group
0.7862318840579711
963	P26	I15	Post-cholecystectomy	Control group
0.6548436308161708
964	P26	I17	Post-cholecystectomy	Control group
0.937070938215103
965	P26	I18	Post-cholecystectomy	Control group
0.8811975591151793
966	P26	I19	Post-cholecystectomy	Control group
0.9416475972540046
967	P26	I22	Post-cholecystectomy	Control group
0.8977879481311976
968	P26	I23	Post-cholecystectomy	Control group
0.7974828375286042
969	P26	I24	Post-cholecystectomy	Control group
0.8794813119755912
970	P26	I25	Post-cholecystectomy	Control group
0.8335240274599542
971	P26	I26	Post-cholecystectomy	Control group
0.6523646071700991
972	P26	I27	Post-cholecystectomy	Control group
0.7107170099160945
973	P26	I28	Post-cholecystectomy	Control group
0.8735697940503433
974	P26	I29	Post-cholecystectomy	Control group
0.834096109839817
975	P26	I30	Post-cholecystectomy	Control group
0.7238749046529367
976	P26	I31	Post-cholecystectomy	Control group
0.852974828375286
977	P26	I32	Post-cholecystectomy	Control group
0.8705186880244088
978	P26	I33	Post-cholecystectomy	Control group
0.8750953470633105
979	P26	I34	Post-cholecystectomy	Control group
0.8077803203661327
980	P26	I35	Post-cholecystectomy	Control group
0.8621281464530892
981	P26	I36	Post-cholecystectomy	Control group

0.9887490465293669
982 P26 I37 Post-cholecystectomy Control group
0.9511823035850496
983 P30 B27 Post-cholecystectomy Control group
0.8274218154080855
984 P30 B66 Post-cholecystectomy Control group
0.8901601830663616
985 P30 B86 Post-cholecystectomy Control group
0.9145690312738368
986 P30 B97 Post-cholecystectomy Control group
0.8373379099923722
987 P30 B98 Post-cholecystectomy Control group
0.8085430968726163
988 P30 B100 Post-cholecystectomy Control group
0.7734553775743707
989 P30 B112 Post-cholecystectomy Control group
0.9487032799389779
990 P30 B115 Post-cholecystectomy Control group
0.8005339435545386
991 P30 I1 Post-cholecystectomy Control group
0.7868039664378337
992 P30 I3 Post-cholecystectomy Control group
0.9376430205949656
993 P30 I6 Post-cholecystectomy Control group
0.841723874904653
994 P30 I8 Post-cholecystectomy Control group
0.9098016781083142
995 P30 I10 Post-cholecystectomy Control group
0.9340198321891686
996 P30 I11 Post-cholecystectomy Control group
0.8897787948131197
997 P30 I13 Post-cholecystectomy Control group
0.9258199847444699
998 P30 I15 Post-cholecystectomy Control group
0.9271548436308161
999 P30 I17 Post-cholecystectomy Control group
0.9195270785659801
1000 P30 I18 Post-cholecystectomy Control group
0.9223874904652937
1001 P30 I19 Post-cholecystectomy Control group
0.9565217391304348
1002 P30 I22 Post-cholecystectomy Control group
0.8937833714721587
1003 P30 I23 Post-cholecystectomy Control group
0.8653699466056446
1004 P30 I24 Post-cholecystectomy Control group
0.9590007627765065
1005 P30 I25 Post-cholecystectomy Control group
0.9006483600305111
1006 P30 I26 Post-cholecystectomy Control group
0.9302059496567505
1007 P30 I27 Post-cholecystectomy Control group
0.9223874904652937
1008 P30 I28 Post-cholecystectomy Control group

0.8825324180015256
1009	P30	I29	Post-cholecystectomy	Control group
0.9416475972540046
1010	P30	I30	Post-cholecystectomy	Control group
0.9256292906178489
1011	P30	I31	Post-cholecystectomy	Control group
0.910373760488177
1012	P30	I32	Post-cholecystectomy	Control group
0.9544241037376049
1013	P30	I33	Post-cholecystectomy	Control group
0.9376430205949656
1014	P30	I34	Post-cholecystectomy	Control group
0.9147597254004577
1015	P30	I35	Post-cholecystectomy	Control group
0.9296338672768879
1016	P30	I36	Post-cholecystectomy	Control group
0.9683447749809306
1017	P30	I37	Post-cholecystectomy	Control group
0.8691838291380626
1018	P33	B27	Post-cholecystectomy	Control group
0.835812356979405
1019	P33	B66	Post-cholecystectomy	Control group
0.7778413424866514
1020	P33	B86	Post-cholecystectomy	Control group
0.7648741418764302
1021	P33	B97	Post-cholecystectomy	Control group
0.7183447749809306
1022	P33	B98	Post-cholecystectomy	Control group
0.8003432494279176
1023	P33	B100	Post-cholecystectomy	Control group
0.9040808543096872
1024	P33	B112	Post-cholecystectomy	Control group
0.8779557589626239
1025	P33	B115	Post-cholecystectomy	Control group
0.8026315789473685
1026	P33	I1	Post-cholecystectomy	Control group
0.814836003051106
1027	P33	I3	Post-cholecystectomy	Control group
0.6786803966437833
1028	P33	I6	Post-cholecystectomy	Control group
0.7660183066361556
1029	P33	I8	Post-cholecystectomy	Control group
0.8653699466056446
1030	P33	I10	Post-cholecystectomy	Control group
0.736651411136537
1031	P33	I11	Post-cholecystectomy	Control group
0.8762395118230358
1032	P33	I13	Post-cholecystectomy	Control group
0.8117848970251716
1033	P33	I15	Post-cholecystectomy	Control group
0.6966056445461479
1034	P33	I17	Post-cholecystectomy	Control group
0.8463005339435545
1035	P33	I18	Post-cholecystectomy	Control group

0.7425629290617849
1036	P33	I19	Post-cholecystectomy	Control group
0.8960717009916095
1037	P33	I22	Post-cholecystectomy	Control group
0.8045385202135774
1038	P33	I23	Post-cholecystectomy	Control group
0.6922196796338673
1039	P33	I24	Post-cholecystectomy	Control group
0.8039664378337147
1040	P33	I25	Post-cholecystectomy	Control group
0.7978642257818459
1041	P33	I26	Post-cholecystectomy	Control group
0.6969870327993898
1042	P33	I27	Post-cholecystectomy	Control group
0.7379862700228833
1043	P33	I28	Post-cholecystectomy	Control group
0.8337147215865751
1044	P33	I29	Post-cholecystectomy	Control group
0.8438215102974829
1045	P33	I30	Post-cholecystectomy	Control group
0.639397406559878
1046	P33	I31	Post-cholecystectomy	Control group
0.8699466056445462
1047	P33	I32	Post-cholecystectomy	Control group
0.8459191456903128
1048	P33	I33	Post-cholecystectomy	Control group
0.8392448512585813
1049	P33	I34	Post-cholecystectomy	Control group
0.8209382151029748
1050	P33	I35	Post-cholecystectomy	Control group
0.8363844393592678
1051	P33	I36	Post-cholecystectomy	Control group
0.9879862700228833
1052	P33	I37	Post-cholecystectomy	Control group
0.9290617848970252
1053	P35	B27	Post-cholecystectomy	Control group
0.8655606407322655
1054	P35	B66	Post-cholecystectomy	Control group
0.7181540808543097
1055	P35	B86	Post-cholecystectomy	Control group
0.8104500381388253
1056	P35	B97	Post-cholecystectomy	Control group
0.7343630816170862
1057	P35	B98	Post-cholecystectomy	Control group
0.8274218154080855
1058	P35	B100	Post-cholecystectomy	Control group
0.8985507246376812
1059	P35	B112	Post-cholecystectomy	Control group
0.9004576659038902
1060	P35	B115	Post-cholecystectomy	Control group
0.7782227307398932
1061	P35	I1	Post-cholecystectomy	Control group
0.84744469870328
1062	P35	I3	Post-cholecystectomy	Control group

0.8531655225019069
1063   P35   I6   Post-cholecystectomy   Control group
0.7559115179252479
1064   P35   I8   Post-cholecystectomy   Control group
0.8632723112128147
1065   P35   I10   Post-cholecystectomy   Control group
0.7929061784897025
1066   P35   I11   Post-cholecystectomy   Control group
0.9099923722349351
1067   P35   I13   Post-cholecystectomy   Control group
0.7570556826849733
1068   P35   I15   Post-cholecystectomy   Control group
0.7862318840579711
1069   P35   I17   Post-cholecystectomy   Control group
0.8895881006864989
1070   P35   I18   Post-cholecystectomy   Control group
0.8201754385964912
1071   P35   I19   Post-cholecystectomy   Control group
0.9101830663615561
1072   P35   I22   Post-cholecystectomy   Control group
0.8565980167810832
1073   P35   I23   Post-cholecystectomy   Control group
0.7400839054157132
1074   P35   I24   Post-cholecystectomy   Control group
0.8508771929824561
1075   P35   I25   Post-cholecystectomy   Control group
0.8451563691838292
1076   P35   I26   Post-cholecystectomy   Control group
0.6960335621662853
1077   P35   I27   Post-cholecystectomy   Control group
0.6826849733028223
1078   P35   I28   Post-cholecystectomy   Control group
0.8926392067124332
1079   P35   I29   Post-cholecystectomy   Control group
0.8733790999237223
1080   P35   I30   Post-cholecystectomy   Control group
0.772883295194508
1081   P35   I31   Post-cholecystectomy   Control group
0.8537376048817696
1082   P35   I32   Post-cholecystectomy   Control group
0.8611746758199847
1083   P35   I33   Post-cholecystectomy   Control group
0.8283752860411899
1084   P35   I34   Post-cholecystectomy   Control group
0.753813882532418
1085   P35   I35   Post-cholecystectomy   Control group
0.8175057208237986
1086   P35   I36   Post-cholecystectomy   Control group
0.9870327993897788
1087   P35   I37   Post-cholecystectomy   Control group
0.9202898550724637
1088   P38   B27   Post-cholecystectomy   Control group
0.9393592677345538
1089   P38   B66   Post-cholecystectomy   Control group

0.8157894736842105
1090 P38 B86 Post-cholecystectomy Control group
0.9334477498093059
1091 P38 B97 Post-cholecystectomy Control group
0.9113272311212814
1092 P38 B98 Post-cholecystectomy Control group
0.938977879481312
1093 P38 B100 Post-cholecystectomy Control group
0.21929824561403508
1094 P38 B112 Post-cholecystectomy Control group
0.9662471395881007
1095 P38 B115 Post-cholecystectomy Control group
0.9429824561403509
1096 P38 I1 Post-cholecystectomy Control group
0.9052250190694127
1097 P38 I3 Post-cholecystectomy Control group
0.936880244088482
1098 P38 I6 Post-cholecystectomy Control group
0.9488939740655988
1099 P38 I8 Post-cholecystectomy Control group
0.9435545385202135
1100 P38 I10 Post-cholecystectomy Control group
0.9538520213577422
1101 P38 I11 Post-cholecystectomy Control group
0.9191456903127384
1102 P38 I13 Post-cholecystectomy Control group
0.959954233409611
1103 P38 I15 Post-cholecystectomy Control group
0.9534706331045004
1104 P38 I17 Post-cholecystectomy Control group
0.9601449275362319
1105 P38 I18 Post-cholecystectomy Control group
0.948512585812357
1106 P38 I19 Post-cholecystectomy Control group
0.9549961861174676
1107 P38 I22 Post-cholecystectomy Control group
0.9429824561403509
1108 P38 I23 Post-cholecystectomy Control group
0.9250572082379863
1109 P38 I24 Post-cholecystectomy Control group
0.9786422578184591
1110 P38 I25 Post-cholecystectomy Control group
0.9515636918382914
1111 P38 I26 Post-cholecystectomy Control group
0.9235316552250191
1112 P38 I27 Post-cholecystectomy Control group
0.9511823035850496
1113 P38 I28 Post-cholecystectomy Control group
0.9241037376048817
1114 P38 I29 Post-cholecystectomy Control group
0.9620518688024409
1115 P38 I30 Post-cholecystectomy Control group
0.9395499618611747
1116 P38 I31 Post-cholecystectomy Control group

0.9731121281464531
1117	P38	I32	Post-cholecystectomy	Control group
0.9858886346300534
1118	P38	I33	Post-cholecystectomy	Control group
0.9719679633867276
1119	P38	I34	Post-cholecystectomy	Control group
0.9105644546147978
1120	P38	I35	Post-cholecystectomy	Control group
0.9340198321891686
1121	P38	I36	Post-cholecystectomy	Control group
0.893211289092296
1122	P38	I37	Post-cholecystectomy	Control group
0.9187643020594966
1123	P39	B27	Post-cholecystectomy	Control group
0.9101830663615561
1124	P39	B66	Post-cholecystectomy	Control group
0.8459191456903128
1125	P39	B86	Post-cholecystectomy	Control group
0.9187643020594966
1126	P39	B97	Post-cholecystectomy	Control group
0.8613653699466056
1127	P39	B98	Post-cholecystectomy	Control group
0.8459191456903128
1128	P39	B100	Post-cholecystectomy	Control group
0.9210526315789473
1129	P39	B112	Post-cholecystectomy	Control group
0.9155225019069413
1130	P39	B115	Post-cholecystectomy	Control group
0.8728070175438597
1131	P39	I1	Post-cholecystectomy	Control group
0.8922578184591915
1132	P39	I3	Post-cholecystectomy	Control group
0.7387490465293669
1133	P39	I6	Post-cholecystectomy	Control group
0.8035850495804729
1134	P39	I8	Post-cholecystectomy	Control group
0.9189549961861174
1135	P39	I10	Post-cholecystectomy	Control group
0.8975972540045767
1136	P39	I11	Post-cholecystectomy	Control group
0.8367658276125095
1137	P39	I13	Post-cholecystectomy	Control group
0.8594584286803967
1138	P39	I15	Post-cholecystectomy	Control group
0.8659420289855072
1139	P39	I17	Post-cholecystectomy	Control group
0.9111365369946606
1140	P39	I18	Post-cholecystectomy	Control group
0.8314263920671243
1141	P39	I19	Post-cholecystectomy	Control group
0.9704424103737604
1142	P39	I22	Post-cholecystectomy	Control group
0.8598398169336384
1143	P39	I23	Post-cholecystectomy	Control group

0.8659420289855072
1144	P39	I24	Post-cholecystectomy	Control group
0.9532799389778794
1145	P39	I25	Post-cholecystectomy	Control group
0.8672768878718535
1146	P39	I26	Post-cholecystectomy	Control group
0.8831045003813882
1147	P39	I27	Post-cholecystectomy	Control group
0.7852784134248665
1148	P39	I28	Post-cholecystectomy	Control group
0.7789855072463768
1149	P39	I29	Post-cholecystectomy	Control group
0.9500381388253242
1150	P39	I30	Post-cholecystectomy	Control group
0.8007246376811594
1151	P39	I31	Post-cholecystectomy	Control group
0.9017925247902364
1152	P39	I32	Post-cholecystectomy	Control group
0.8979786422578184
1153	P39	I33	Post-cholecystectomy	Control group
0.9004576659038902
1154	P39	I34	Post-cholecystectomy	Control group
0.9183829138062548
1155	P39	I35	Post-cholecystectomy	Control group
0.8346681922196796
1156	P39	I36	Post-cholecystectomy	Control group
0.9601449275362319
1157	P39	I37	Post-cholecystectomy	Control group
0.8806254767353165
1158	P42	B27	Post-cholecystectomy	Control group
0.919908466819222
1159	P42	B66	Post-cholecystectomy	Control group
0.8011060259344012
1160	P42	B86	Post-cholecystectomy	Control group
0.8676582761250954
1161	P42	B97	Post-cholecystectomy	Control group
0.8232265446224256
1162	P42	B98	Post-cholecystectomy	Control group
0.8857742181540809
1163	P42	B100	Post-cholecystectomy	Control group
0.9187643020594966
1164	P42	B112	Post-cholecystectomy	Control group
0.9393592677345538
1165	P42	B115	Post-cholecystectomy	Control group
0.8518306636155606
1166	P42	I1	Post-cholecystectomy	Control group
0.9324942791762014
1167	P42	I3	Post-cholecystectomy	Control group
0.9597635392829901
1168	P42	I6	Post-cholecystectomy	Control group
0.8297101449275363
1169	P42	I8	Post-cholecystectomy	Control group
0.9515636918382914
1170	P42	I10	Post-cholecystectomy	Control group

0.9359267734553776
1171 P42 I11 Post-cholecystectomy Control group
0.9235316552250191
1172 P42 I13 Post-cholecystectomy Control group
0.8792906178489702
1173 P42 I15 Post-cholecystectomy Control group
0.7711670480549199
1174 P42 I17 Post-cholecystectomy Control group
0.9881769641495042
1175 P42 I18 Post-cholecystectomy Control group
0.8689931350114416
1176 P42 I19 Post-cholecystectomy Control group
0.9456521739130435
1177 P42 I22 Post-cholecystectomy Control group
0.9269641495041953
1178 P42 I23 Post-cholecystectomy Control group
0.8920671243325705
1179 P42 I24 Post-cholecystectomy Control group
0.9347826086956522
1180 P42 I25 Post-cholecystectomy Control group
0.9101830663615561
1181 P42 I26 Post-cholecystectomy Control group
0.8884439359267735
1182 P42 I27 Post-cholecystectomy Control group
0.8653699466056446
1183 P42 I28 Post-cholecystectomy Control group
0.9220061022120518
1184 P42 I29 Post-cholecystectomy Control group
0.9441266209000763
1185 P42 I30 Post-cholecystectomy Control group
0.7601067887109078
1186 P42 I31 Post-cholecystectomy Control group
0.9748283752860412
1187 P42 I32 Post-cholecystectomy Control group
0.9557589626239512
1188 P42 I33 Post-cholecystectomy Control group
0.9317315026697178
1189 P42 I34 Post-cholecystectomy Control group
0.7030892448512586
1190 P42 I35 Post-cholecystectomy Control group
0.9576659038901602
1191 P42 I36 Post-cholecystectomy Control group
0.9811212814645309
1192 P42 I37 Post-cholecystectomy Control group
0.9677726926010679
1193 P43 B27 Post-cholecystectomy Control group
0.8384820747520977
1194 P43 B66 Post-cholecystectomy Control group
0.7721205186880244
1195 P43 B86 Post-cholecystectomy Control group
0.8335240274599542
1196 P43 B97 Post-cholecystectomy Control group
0.8079710144927537
1197 P43 B98 Post-cholecystectomy Control group

0.7835621662852784
1198	P43	B100	Post-cholecystectomy	Control group
0.956140350877193
1199	P43	B112	Post-cholecystectomy	Control group
0.8779557589626239
1200	P43	B115	Post-cholecystectomy	Control group
0.8234172387490465
1201	P43	I1	Post-cholecystectomy	Control group
0.7631578947368421
1202	P43	I3	Post-cholecystectomy	Control group
0.6224256292906178
1203	P43	I6	Post-cholecystectomy	Control group
0.8047292143401983
1204	P43	I8	Post-cholecystectomy	Control group
0.8381006864988558
1205	P43	I10	Post-cholecystectomy	Control group
0.7915713196033562
1206	P43	I11	Post-cholecystectomy	Control group
0.8590770404271548
1207	P43	I13	Post-cholecystectomy	Control group
0.7049961861174676
1208	P43	I15	Post-cholecystectomy	Control group
0.7946224256292906
1209	P43	I17	Post-cholecystectomy	Control group
0.8487795575896262
1210	P43	I18	Post-cholecystectomy	Control group
0.7683066361556065
1211	P43	I19	Post-cholecystectomy	Control group
0.9191456903127384
1212	P43	I22	Post-cholecystectomy	Control group
0.7879481311975591
1213	P43	I23	Post-cholecystectomy	Control group
0.7534324942791762
1214	P43	I24	Post-cholecystectomy	Control group
0.8506864988558352
1215	P43	I25	Post-cholecystectomy	Control group
0.7236842105263158
1216	P43	I26	Post-cholecystectomy	Control group
0.6750572082379863
1217	P43	I27	Post-cholecystectomy	Control group
0.7654462242562929
1218	P43	I28	Post-cholecystectomy	Control group
0.7379862700228833
1219	P43	I29	Post-cholecystectomy	Control group
0.7513348588863463
1220	P43	I30	Post-cholecystectomy	Control group
0.8012967200610221
1221	P43	I31	Post-cholecystectomy	Control group
0.7982456140350878
1222	P43	I32	Post-cholecystectomy	Control group
0.7732646834477498
1223	P43	I33	Post-cholecystectomy	Control group
0.8560259344012204
1224	P43	I34	Post-cholecystectomy	Control group

					0.9063691838291381
1225	P43	I35	Post-cholecystectomy	Control group	0.7072845156369184
1226	P43	I36	Post-cholecystectomy	Control group	0.9931350114416476
1227	P43	I37	Post-cholecystectomy	Control group	0.8813882532418001
1228	P46	B27	Post-cholecystectomy	Control group	0.7143401983218917
1229	P46	B66	Post-cholecystectomy	Control group	0.7456140350877193
1230	P46	B86	Post-cholecystectomy	Control group	0.690884820747521
1231	P46	B97	Post-cholecystectomy	Control group	0.7204424103737604
1232	P46	B98	Post-cholecystectomy	Control group	0.7759344012204424
1233	P46	B100	Post-cholecystectomy	Control group	0.9496567505720824
1234	P46	B112	Post-cholecystectomy	Control group	0.7541952707856598
1235	P46	B115	Post-cholecystectomy	Control group	0.8411517925247902
1236	P46	I1	Post-cholecystectomy	Control group	0.8283752860411899
1237	P46	I3	Post-cholecystectomy	Control group	0.738367658276125
1238	P46	I6	Post-cholecystectomy	Control group	0.8106407322654462
1239	P46	I8	Post-cholecystectomy	Control group	0.8773836765827613
1240	P46	I10	Post-cholecystectomy	Control group	0.7742181540808543
1241	P46	I11	Post-cholecystectomy	Control group	0.8607932875667429
1242	P46	I13	Post-cholecystectomy	Control group	0.831998474446987
1243	P46	I15	Post-cholecystectomy	Control group	0.8072082379862701
1244	P46	I17	Post-cholecystectomy	Control group	0.7641113653699466
1245	P46	I18	Post-cholecystectomy	Control group	0.7650648360030511
1246	P46	I19	Post-cholecystectomy	Control group	0.8012967200610221
1247	P46	I22	Post-cholecystectomy	Control group	0.8346681922196796
1248	P46	I23	Post-cholecystectomy	Control group	0.7684973302822273
1249	P46	I24	Post-cholecystectomy	Control group	0.6592295957284515
1250	P46	I25	Post-cholecystectomy	Control group	0.7789855072463768
1251	P46	I26	Post-cholecystectomy	Control group	

0.7698321891685737
1252	P46	I27	Post-cholecystectomy	Control group
0.8093058733790999
1253	P46	I28	Post-cholecystectomy	Control group
0.8442028985507246
1254	P46	I29	Post-cholecystectomy	Control group
0.8617467581998475
1255	P46	I30	Post-cholecystectomy	Control group
0.7948131197559115
1256	P46	I31	Post-cholecystectomy	Control group
0.8953089244851259
1257	P46	I32	Post-cholecystectomy	Control group
0.8642257818459191
1258	P46	I33	Post-cholecystectomy	Control group
0.6043096872616324
1259	P46	I34	Post-cholecystectomy	Control group
0.7091914569031273
1260	P46	I35	Post-cholecystectomy	Control group
0.7929061784897025
1261	P46	I36	Post-cholecystectomy	Control group
0.9647215865751335
1262	P46	I37	Post-cholecystectomy	Control group
0.9073226544622426
1263	P47	B27	Post-cholecystectomy	Control group
0.5467200610221206
1264	P47	B66	Post-cholecystectomy	Control group
0.9460335621662853
1265	P47	B86	Post-cholecystectomy	Control group
0.6903127383676583
1266	P47	B97	Post-cholecystectomy	Control group
0.9113272311212814
1267	P47	B98	Post-cholecystectomy	Control group
0.9233409610983981
1268	P47	B100	Post-cholecystectomy	Control group
0.9553775743707094
1269	P47	B112	Post-cholecystectomy	Control group
0.7847063310450039
1270	P47	B115	Post-cholecystectomy	Control group
0.9324942791762014
1271	P47	I1	Post-cholecystectomy	Control group
0.937070938215103
1272	P47	I3	Post-cholecystectomy	Control group
0.9138062547673532
1273	P47	I6	Post-cholecystectomy	Control group
0.9248665141113653
1274	P47	I8	Post-cholecystectomy	Control group
0.9405034324942791
1275	P47	I10	Post-cholecystectomy	Control group
0.948512585812357
1276	P47	I11	Post-cholecystectomy	Control group
0.9176201372997712
1277	P47	I13	Post-cholecystectomy	Control group
0.8934019832189168
1278	P47	I15	Post-cholecystectomy	Control group

0.9239130434782609
1279 P47 I17 Post-cholecystectomy Control group
0.7088100686498856
1280 P47 I18 Post-cholecystectomy Control group
0.8861556064073226
1281 P47 I19 Post-cholecystectomy Control group
0.7831807780320366
1282 P47 I22 Post-cholecystectomy Control group
0.9193363844393593
1283 P47 I23 Post-cholecystectomy Control group
0.915903890160183
1284 P47 I24 Post-cholecystectomy Control group
0.5379481311975591
1285 P47 I25 Post-cholecystectomy Control group
0.9366895499618612
1286 P47 I26 Post-cholecystectomy Control group
0.9244851258581236
1287 P47 I27 Post-cholecystectomy Control group
0.9263920671243325
1288 P47 I28 Post-cholecystectomy Control group
0.9176201372997712
1289 P47 I29 Post-cholecystectomy Control group
0.9416475972540046
1290 P47 I30 Post-cholecystectomy Control group
0.956140350877193
1291 P47 I31 Post-cholecystectomy Control group
0.9385964912280702
1292 P47 I32 Post-cholecystectomy Control group
0.9429824561403509
1293 P47 I33 Post-cholecystectomy Control group
0.5694126620900076
1294 P47 I34 Post-cholecystectomy Control group
0.7797482837528604
1295 P47 I35 Post-cholecystectomy Control group
0.9136155606407322
1296 P47 I36 Post-cholecystectomy Control group
0.9593821510297483
1297 P47 I37 Post-cholecystectomy Control group
0.9420289855072463
1298 P50 B27 Post-cholecystectomy Control group
0.7770785659801678
1299 P50 B66 Post-cholecystectomy Control group
0.7608695652173914
1300 P50 B86 Post-cholecystectomy Control group
0.7570556826849733
1301 P50 B97 Post-cholecystectomy Control group
0.708047292143402
1302 P50 B98 Post-cholecystectomy Control group
0.7280701754385965
1303 P50 B100 Post-cholecystectomy Control group
0.92372234935164
1304 P50 B112 Post-cholecystectomy Control group
0.8215102974828375
1305 P50 B115 Post-cholecystectomy Control group

0.7397025171624714
1306 P50 I1 Post-cholecystectomy Control group
0.7318840579710145
1307 P50 I3 Post-cholecystectomy Control group
0.6948893974065599
1308 P50 I6 Post-cholecystectomy Control group
0.7131960335621663
1309 P50 I8 Post-cholecystectomy Control group
0.8136918382913806
1310 P50 I10 Post-cholecystectomy Control group
0.7484744469870328
1311 P50 I11 Post-cholecystectomy Control group
0.8861556064073226
1312 P50 I13 Post-cholecystectomy Control group
0.8041571319603357
1313 P50 I15 Post-cholecystectomy Control group
0.7953852021357742
1314 P50 I17 Post-cholecystectomy Control group
0.8293287566742944
1315 P50 I18 Post-cholecystectomy Control group
0.7196796338672768
1316 P50 I19 Post-cholecystectomy Control group
0.8480167810831426
1317 P50 I22 Post-cholecystectomy Control group
0.8022501906941266
1318 P50 I23 Post-cholecystectomy Control group
0.6447368421052632
1319 P50 I24 Post-cholecystectomy Control group
0.8197940503432495
1320 P50 I25 Post-cholecystectomy Control group
0.7545766590389016
1321 P50 I26 Post-cholecystectomy Control group
0.6996567505720824
1322 P50 I27 Post-cholecystectomy Control group
0.690884820747521
1323 P50 I28 Post-cholecystectomy Control group
0.8710907704042715
1324 P50 I29 Post-cholecystectomy Control group
0.8299008390541571
1325 P50 I30 Post-cholecystectomy Control group
0.7597254004576659
1326 P50 I31 Post-cholecystectomy Control group
0.851067887109077
1327 P50 I32 Post-cholecystectomy Control group
0.8632723112128147
1328 P50 I33 Post-cholecystectomy Control group
0.7789855072463768
1329 P50 I34 Post-cholecystectomy Control group
0.7827993897787948
1330 P50 I35 Post-cholecystectomy Control group
0.7745995423340961
1331 P50 I36 Post-cholecystectomy Control group
0.9929443173150267
1332 P50 I37 Post-cholecystectomy Control group

0.8318077803203662
1333 P55 B27 Post-cholecystectomy Control group
0.7469488939740656
1334 P55 B66 Post-cholecystectomy Control group
0.7623951182303585
1335 P55 B86 Post-cholecystectomy Control group
0.7368421052631579
1336 P55 B97 Post-cholecystectomy Control group
0.6544622425629291
1337 P55 B98 Post-cholecystectomy Control group
0.7036613272311213
1338 P55 B100 Post-cholecystectomy Control group
0.9635774218154081
1339 P55 B112 Post-cholecystectomy Control group
0.8197940503432495
1340 P55 B115 Post-cholecystectomy Control group
0.6512204424103738
1341 P55 I1 Post-cholecystectomy Control group
0.650839054157132
1342 P55 I3 Post-cholecystectomy Control group
0.7540045766590389
1343 P55 I6 Post-cholecystectomy Control group
0.6340579710144928
1344 P55 I8 Post-cholecystectomy Control group
0.704042715484363
1345 P55 I10 Post-cholecystectomy Control group
0.7110983981693364
1346 P55 I11 Post-cholecystectomy Control group
0.8790999237223494
1347 P55 I13 Post-cholecystectomy Control group
0.7339816933638444
1348 P55 I15 Post-cholecystectomy Control group
0.7458047292143402
1349 P55 I17 Post-cholecystectomy Control group
0.8018688024408849
1350 P55 I18 Post-cholecystectomy Control group
0.729023646071701
1351 P55 I19 Post-cholecystectomy Control group
0.8832951945080092
1352 P55 I22 Post-cholecystectomy Control group
0.782418001525553
1353 P55 I23 Post-cholecystectomy Control group
0.7093821510297483
1354 P55 I24 Post-cholecystectomy Control group
0.801487414187643
1355 P55 I25 Post-cholecystectomy Control group
0.6510297482837528
1356 P55 I26 Post-cholecystectomy Control group
0.7221586575133486
1357 P55 I27 Post-cholecystectomy Control group
0.7646834477498093
1358 P55 I28 Post-cholecystectomy Control group
0.8585049580472921
1359 P55 I29 Post-cholecystectomy Control group

0.8211289092295957
1360 P55 I30 Post-cholecystectomy Control group
0.7084286803966438
1361 P55 I31 Post-cholecystectomy Control group
0.7961479786422578
1362 P55 I32 Post-cholecystectomy Control group
0.7547673531655225
1363 P55 I33 Post-cholecystectomy Control group
0.7936689549961862
1364 P55 I34 Post-cholecystectomy Control group
0.8255148741418764
1365 P55 I35 Post-cholecystectomy Control group
0.7625858123569794
1366 P55 I36 Post-cholecystectomy Control group
0.9998093058733791
1367 P55 I37 Post-cholecystectomy Control group
0.8257055682684973
1368 P58 B27 Post-cholecystectomy Control group
0.8569794050343249
1369 P58 B66 Post-cholecystectomy Control group
0.7185354691075515
1370 P58 B86 Post-cholecystectomy Control group
0.8217009916094584
1371 P58 B97 Post-cholecystectomy Control group
0.7492372234935164
1372 P58 B98 Post-cholecystectomy Control group
0.7772692601067888
1373 P58 B100 Post-cholecystectomy Control group
0.9498474446987033
1374 P58 B112 Post-cholecystectomy Control group
0.8934019832189168
1375 P58 B115 Post-cholecystectomy Control group
0.8218916857360793
1376 P58 I1 Post-cholecystectomy Control group
0.7749809305873379
1377 P58 I3 Post-cholecystectomy Control group
0.791952707856598
1378 P58 I6 Post-cholecystectomy Control group
0.6241418764302059
1379 P58 I8 Post-cholecystectomy Control group
0.897025171624714
1380 P58 I10 Post-cholecystectomy Control group
0.8758581235697941
1381 P58 I11 Post-cholecystectomy Control group
0.8897787948131197
1382 P58 I13 Post-cholecystectomy Control group
0.8371472158657514
1383 P58 I15 Post-cholecystectomy Control group
0.7114797864225781
1384 P58 I17 Post-cholecystectomy Control group
0.9162852784134249
1385 P58 I18 Post-cholecystectomy Control group
0.8064454614797865
1386 P58 I19 Post-cholecystectomy Control group

0.9040808543096872
1387 P58 I22 Post-cholecystectomy Control group
0.8518306636155606
1388 P58 I23 Post-cholecystectomy Control group
0.7839435545385202
1389 P58 I24 Post-cholecystectomy Control group
0.881769641495042
1390 P58 I25 Post-cholecystectomy Control group
0.8249427917620137
1391 P58 I26 Post-cholecystectomy Control group
0.7902364607170099
1392 P58 I27 Post-cholecystectomy Control group
0.7234935163996948
1393 P58 I28 Post-cholecystectomy Control group
0.9139969488939741
1394 P58 I29 Post-cholecystectomy Control group
0.7971014492753623
1395 P58 I30 Post-cholecystectomy Control group
0.7032799389778794
1396 P58 I31 Post-cholecystectomy Control group
0.8846300533943554
1397 P58 I32 Post-cholecystectomy Control group
0.9115179252479023
1398 P58 I33 Post-cholecystectomy Control group
0.8485888634630053
1399 P58 I34 Post-cholecystectomy Control group
0.78813882532418
1400 P58 I35 Post-cholecystectomy Control group
0.816742944317315
1401 P58 I36 Post-cholecystectomy Control group
0.9841723874904653
1402 P58 I37 Post-cholecystectomy Control group
0.9101830663615561
1403 P60 B27 Post-cholecystectomy Control group
0.8745232646834478
1404 P60 B66 Post-cholecystectomy Control group
0.9300152555301296
1405 P60 B86 Post-cholecystectomy Control group
0.9387871853546911
1406 P60 B97 Post-cholecystectomy Control group
0.8533562166285278
1407 P60 B98 Post-cholecystectomy Control group
0.8312356979405034
1408 P60 B100 Post-cholecystectomy Control group
0.9273455377574371
1409 P60 B112 Post-cholecystectomy Control group
0.9855072463768116
1410 P60 B115 Post-cholecystectomy Control group
0.8903508771929824
1411 P60 I1 Post-cholecystectomy Control group
0.8688024408848207
1412 P60 I3 Post-cholecystectomy Control group
0.7191075514874142
1413 P60 I6 Post-cholecystectomy Control group

0.881769641495042
1414	P60	I8	Post-cholecystectomy	Control group
0.9776887871853547
1415	P60	I10	Post-cholecystectomy	Control group
0.8920671243325705
1416	P60	I11	Post-cholecystectomy	Control group
0.8785278413424866
1417	P60	I13	Post-cholecystectomy	Control group
0.9233409610983981
1418	P60	I15	Post-cholecystectomy	Control group
0.9426010678871091
1419	P60	I17	Post-cholecystectomy	Control group
0.9498474446987033
1420	P60	I18	Post-cholecystectomy	Control group
0.8506864988558352
1421	P60	I19	Post-cholecystectomy	Control group
0.9837909992372235
1422	P60	I22	Post-cholecystectomy	Control group
0.9128527841342486
1423	P60	I23	Post-cholecystectomy	Control group
0.8499237223493517
1424	P60	I24	Post-cholecystectomy	Control group
0.9757818459191457
1425	P60	I25	Post-cholecystectomy	Control group
0.9126620900076278
1426	P60	I26	Post-cholecystectomy	Control group
0.9014111365369947
1427	P60	I27	Post-cholecystectomy	Control group
0.8800533943554538
1428	P60	I28	Post-cholecystectomy	Control group
0.7210144927536232
1429	P60	I29	Post-cholecystectomy	Control group
0.9174294431731502
1430	P60	I30	Post-cholecystectomy	Control group
0.9046529366895499
1431	P60	I31	Post-cholecystectomy	Control group
0.9200991609458429
1432	P60	I32	Post-cholecystectomy	Control group
0.9431731502669718
1433	P60	I33	Post-cholecystectomy	Control group
0.9412662090007627
1434	P60	I34	Post-cholecystectomy	Control group
0.9492753623188406
1435	P60	I35	Post-cholecystectomy	Control group
0.8489702517162472
1436	P60	I36	Post-cholecystectomy	Control group
0.9715865751334859
1437	P60	I37	Post-cholecystectomy	Control group
0.8745232646834478
1438	P63	B27	Post-cholecystectomy	Control group
0.8314263920671243
1439	P63	B66	Post-cholecystectomy	Control group
0.7602974828375286
1440	P63	B86	Post-cholecystectomy	Control group

0.784324942791762
1441	P63	B97	Post-cholecystectomy	Control group
0.6500762776506483
1442	P63	B98	Post-cholecystectomy	Control group
0.7736460717009916
1443	P63	B100	Post-cholecystectomy	Control group
0.9393592677345538
1444	P63	B112	Post-cholecystectomy	Control group
0.8794813119755912
1445	P63	B115	Post-cholecystectomy	Control group
0.7930968726163234
1446	P63	I1	Post-cholecystectomy	Control group
0.7438977879481312
1447	P63	I3	Post-cholecystectomy	Control group
0.8165522501906941
1448	P63	I6	Post-cholecystectomy	Control group
0.7858504958047292
1449	P63	I8	Post-cholecystectomy	Control group
0.812929061784897
1450	P63	I10	Post-cholecystectomy	Control group
0.782608695652174
1451	P63	I11	Post-cholecystectomy	Control group
0.8878718535469108
1452	P63	I13	Post-cholecystectomy	Control group
0.8234172387490465
1453	P63	I15	Post-cholecystectomy	Control group
0.772883295194508
1454	P63	I17	Post-cholecystectomy	Control group
0.8449656750572082
1455	P63	I18	Post-cholecystectomy	Control group
0.7848970251716247
1456	P63	I19	Post-cholecystectomy	Control group
0.9143783371472158
1457	P63	I22	Post-cholecystectomy	Control group
0.8020594965675057
1458	P63	I23	Post-cholecystectomy	Control group
0.7713577421815409
1459	P63	I24	Post-cholecystectomy	Control group
0.8506864988558352
1460	P63	I25	Post-cholecystectomy	Control group
0.7913806254767353
1461	P63	I26	Post-cholecystectomy	Control group
0.8375286041189931
1462	P63	I27	Post-cholecystectomy	Control group
0.7909992372234935
1463	P63	I28	Post-cholecystectomy	Control group
0.8455377574370709
1464	P63	I29	Post-cholecystectomy	Control group
0.7845156369183829
1465	P63	I30	Post-cholecystectomy	Control group
0.7254004576659039
1466	P63	I31	Post-cholecystectomy	Control group
0.8022501906941266
1467	P63	I32	Post-cholecystectomy	Control group

0.8350495804729214
1468 P63 I33 Post-cholecystectomy Control group
0.8264683447749809
1469 P63 I34 Post-cholecystectomy Control group
0.8846300533943554
1470 P63 I35 Post-cholecystectomy Control group
0.7940503432494279
1471 P63 I36 Post-cholecystectomy Control group
0.9952326468344775
1472 P63 I37 Post-cholecystectomy Control group
0.9057971014492754
1473 P65 B27 Post-cholecystectomy Control group
0.8916857360793288
1474 P65 B66 Post-cholecystectomy Control group
0.6794431731502669
1475 P65 B86 Post-cholecystectomy Control group
0.7187261632341724
1476 P65 B97 Post-cholecystectomy Control group
0.7580091533180778
1477 P65 B98 Post-cholecystectomy Control group
0.8373379099923722
1478 P65 B100 Post-cholecystectomy Control group
0.9565217391304348
1479 P65 B112 Post-cholecystectomy Control group
0.831998474446987
1480 P65 B115 Post-cholecystectomy Control group
0.7902364607170099
1481 P65 I1 Post-cholecystectomy Control group
0.7810831426392068
1482 P65 I3 Post-cholecystectomy Control group
0.7887109077040427
1483 P65 I6 Post-cholecystectomy Control group
0.7141495041952708
1484 P65 I8 Post-cholecystectomy Control group
0.8485888634630053
1485 P65 I10 Post-cholecystectomy Control group
0.780511060259344
1486 P65 I11 Post-cholecystectomy Control group
0.8790999237223494
1487 P65 I13 Post-cholecystectomy Control group
0.8480167810831426
1488 P65 I15 Post-cholecystectomy Control group
0.797673531655225
1489 P65 I17 Post-cholecystectomy Control group
0.9117086193745233
1490 P65 I18 Post-cholecystectomy Control group
0.8268497330282227
1491 P65 I19 Post-cholecystectomy Control group
0.8878718535469108
1492 P65 I22 Post-cholecystectomy Control group
0.8323798627002288
1493 P65 I23 Post-cholecystectomy Control group
0.7568649885583524
1494 P65 I24 Post-cholecystectomy Control group

0.8455377574370709
1495	P65	I25	Post-cholecystectomy	Control group
0.7908085430968727
1496	P65	I26	Post-cholecystectomy	Control group
0.7700228832951945
1497	P65	I27	Post-cholecystectomy	Control group
0.6727688787185355
1498	P65	I28	Post-cholecystectomy	Control group
0.8832951945080092
1499	P65	I29	Post-cholecystectomy	Control group
0.7776506483600305
1500	P65	I30	Post-cholecystectomy	Control group
0.7591533180778032
1501	P65	I31	Post-cholecystectomy	Control group
0.8325705568268498
1502	P65	I32	Post-cholecystectomy	Control group
0.841723874904653
1503	P65	I33	Post-cholecystectomy	Control group
0.8028222730739893
1504	P65	I34	Post-cholecystectomy	Control group
0.7967200610221206
1505	P65	I35	Post-cholecystectomy	Control group
0.8613653699466056
1506	P65	I36	Post-cholecystectomy	Control group
0.9933257055682685
1507	P65	I37	Post-cholecystectomy	Control group
0.937070938215103
1508	P68	B27	Post-cholecystectomy	Control group
0.8335240274599542
1509	P68	B66	Post-cholecystectomy	Control group
0.772883295194508
1510	P68	B86	Post-cholecystectomy	Control group
0.8697559115179252
1511	P68	B97	Post-cholecystectomy	Control group
0.7320747520976354
1512	P68	B98	Post-cholecystectomy	Control group
0.8546910755148741
1513	P68	B100	Post-cholecystectomy	Control group
0.9487032799389779
1514	P68	B112	Post-cholecystectomy	Control group
0.8712814645308925
1515	P68	B115	Post-cholecystectomy	Control group
0.809115179252479
1516	P68	I1	Post-cholecystectomy	Control group
0.8436308161708619
1517	P68	I3	Post-cholecystectomy	Control group
0.8424866514111365
1518	P68	I6	Post-cholecystectomy	Control group
0.7015636918382914
1519	P68	I8	Post-cholecystectomy	Control group
0.8699466056445462
1520	P68	I10	Post-cholecystectomy	Control group
0.8590770404271548
1521	P68	I11	Post-cholecystectomy	Control group

0.9042715484363082
1522 P68 I13 Post-cholecystectomy Control group
0.8209382151029748
1523 P68 I15 Post-cholecystectomy Control group
0.7412280701754386
1524 P68 I17 Post-cholecystectomy Control group
0.9130434782608695
1525 P68 I18 Post-cholecystectomy Control group
0.797673531655225
1526 P68 I19 Post-cholecystectomy Control group
0.9063691838291381
1527 P68 I22 Post-cholecystectomy Control group
0.8394355453852022
1528 P68 I23 Post-cholecystectomy Control group
0.8579328756674295
1529 P68 I24 Post-cholecystectomy Control group
0.8892067124332571
1530 P68 I25 Post-cholecystectomy Control group
0.8476353928299009
1531 P68 I26 Post-cholecystectomy Control group
0.7988176964149504
1532 P68 I27 Post-cholecystectomy Control group
0.6731502669717773
1533 P68 I28 Post-cholecystectomy Control group
0.9117086193745233
1534 P68 I29 Post-cholecystectomy Control group
0.8352402745995423
1535 P68 I30 Post-cholecystectomy Control group
0.7170099160945843
1536 P68 I31 Post-cholecystectomy Control group
0.9151411136536994
1537 P68 I32 Post-cholecystectomy Control group
0.8806254767353165
1538 P68 I33 Post-cholecystectomy Control group
0.8768115942028986
1539 P68 I34 Post-cholecystectomy Control group
0.8176964149504196
1540 P68 I35 Post-cholecystectomy Control group
0.9138062547673532
1541 P68 I36 Post-cholecystectomy Control group
0.979023646071701
1542 P68 I37 Post-cholecystectomy Control group
0.9572845156369184
1543 P70 B27 Post-cholecystectomy Control group
0.9105644546147978
1544 P70 B66 Post-cholecystectomy Control group
0.8047292143401983
1545 P70 B86 Post-cholecystectomy Control group
0.8281845919145691
1546 P70 B97 Post-cholecystectomy Control group
0.8607932875667429
1547 P70 B98 Post-cholecystectomy Control group
0.8928299008390541
1548 P70 B100 Post-cholecystectomy Control group

0.9706331045003814
1549	P70	B112	Post-cholecystectomy	Control group
0.8852021357742181
1550	P70	B115	Post-cholecystectomy	Control group
0.8674675819984744
1551	P70	I1	Post-cholecystectomy	Control group
0.879862700228833
1552	P70	I3	Post-cholecystectomy	Control group
0.877765064836003
1553	P70	I6	Post-cholecystectomy	Control group
0.7913806254767353
1554	P70	I8	Post-cholecystectomy	Control group
0.8962623951182304
1555	P70	I10	Post-cholecystectomy	Control group
0.8012967200610221
1556	P70	I11	Post-cholecystectomy	Control group
0.9584286803966438
1557	P70	I13	Post-cholecystectomy	Control group
0.8426773455377574
1558	P70	I15	Post-cholecystectomy	Control group
0.7803203661327232
1559	P70	I17	Post-cholecystectomy	Control group
0.9269641495041953
1560	P70	I18	Post-cholecystectomy	Control group
0.8321891685736079
1561	P70	I19	Post-cholecystectomy	Control group
0.8995041952707856
1562	P70	I22	Post-cholecystectomy	Control group
0.8789092295957285
1563	P70	I23	Post-cholecystectomy	Control group
0.8543096872616324
1564	P70	I24	Post-cholecystectomy	Control group
0.8255148741418764
1565	P70	I25	Post-cholecystectomy	Control group
0.8720442410373761
1566	P70	I26	Post-cholecystectomy	Control group
0.7694508009153318
1567	P70	I27	Post-cholecystectomy	Control group
0.6786803966437833
1568	P70	I28	Post-cholecystectomy	Control group
0.8157894736842105
1569	P70	I29	Post-cholecystectomy	Control group
0.8649885583524027
1570	P70	I30	Post-cholecystectomy	Control group
0.725209763539283
1571	P70	I31	Post-cholecystectomy	Control group
0.8983600305110603
1572	P70	I32	Post-cholecystectomy	Control group
0.8859649122807017
1573	P70	I33	Post-cholecystectomy	Control group
0.8121662852784134
1574	P70	I34	Post-cholecystectomy	Control group
0.8045385202135774
1575	P70	I35	Post-cholecystectomy	Control group

0.8710907704042715
1576	P70	I36	Post-cholecystectomy	Control group
0.9654843630816171
1577	P70	I37	Post-cholecystectomy	Control group
0.897025171624714
1578	P71	B27	Post-cholecystectomy	Control group
0.7980549199084668
1579	P71	B66	Post-cholecystectomy	Control group
0.8613653699466056
1580	P71	B86	Post-cholecystectomy	Control group
0.7236842105263158
1581	P71	B97	Post-cholecystectomy	Control group
0.8688024408848207
1582	P71	B98	Post-cholecystectomy	Control group
0.8226544622425629
1583	P71	B100	Post-cholecystectomy	Control group
0.9641495041952708
1584	P71	B112	Post-cholecystectomy	Control group
0.6817315026697178
1585	P71	B115	Post-cholecystectomy	Control group
0.8424866514111365
1586	P71	I1	Post-cholecystectomy	Control group
0.8754767353165522
1587	P71	I3	Post-cholecystectomy	Control group
0.8611746758199847
1588	P71	I6	Post-cholecystectomy	Control group
0.8943554538520213
1589	P71	I8	Post-cholecystectomy	Control group
0.9006483600305111
1590	P71	I10	Post-cholecystectomy	Control group
0.8560259344012204
1591	P71	I11	Post-cholecystectomy	Control group
0.9689168573607932
1592	P71	I13	Post-cholecystectomy	Control group
0.8564073226544623
1593	P71	I15	Post-cholecystectomy	Control group
0.8781464530892449
1594	P71	I17	Post-cholecystectomy	Control group
0.7438977879481312
1595	P71	I18	Post-cholecystectomy	Control group
0.7482837528604119
1596	P71	I19	Post-cholecystectomy	Control group
0.7044241037376049
1597	P71	I22	Post-cholecystectomy	Control group
0.8882532418001525
1598	P71	I23	Post-cholecystectomy	Control group
0.7875667429443173
1599	P71	I24	Post-cholecystectomy	Control group
0.7225400457665904
1600	P71	I25	Post-cholecystectomy	Control group
0.8634630053394355
1601	P71	I26	Post-cholecystectomy	Control group
0.7942410373760488
1602	P71	I27	Post-cholecystectomy	Control group

0.7343630816170862
1603	P71	I28	Post-cholecystectomy	Control group
0.9157131960335622
1604	P71	I29	Post-cholecystectomy	Control group
0.8962623951182304
1605	P71	I30	Post-cholecystectomy	Control group
0.8712814645308925
1606	P71	I31	Post-cholecystectomy	Control group
0.92372234935164
1607	P71	I32	Post-cholecystectomy	Control group
0.8985507246376812
1608	P71	I33	Post-cholecystectomy	Control group
0.7027078565980168
1609	P71	I34	Post-cholecystectomy	Control group
0.719488939740656
1610	P71	I35	Post-cholecystectomy	Control group
0.8874904652936689
1611	P71	I36	Post-cholecystectomy	Control group
0.9958047292143402
1612	P71	I37	Post-cholecystectomy	Control group
0.9666285278413425
1613	P74	B27	Post-cholecystectomy	Control group
0.860602593440122
1614	P74	B66	Post-cholecystectomy	Control group
0.7896643783371472
1615	P74	B86	Post-cholecystectomy	Control group
0.8026315789473685
1616	P74	B97	Post-cholecystectomy	Control group
0.7166285278413425
1617	P74	B98	Post-cholecystectomy	Control group
0.8043478260869565
1618	P74	B100	Post-cholecystectomy	Control group
0.956140350877193
1619	P74	B112	Post-cholecystectomy	Control group
0.849160945842868
1620	P74	B115	Post-cholecystectomy	Control group
0.8331426392067124
1621	P74	I1	Post-cholecystectomy	Control group
0.799580472921434
1622	P74	I3	Post-cholecystectomy	Control group
0.7336003051106026
1623	P74	I6	Post-cholecystectomy	Control group
0.7902364607170099
1624	P74	I8	Post-cholecystectomy	Control group
0.8611746758199847
1625	P74	I10	Post-cholecystectomy	Control group
0.7419908466819222
1626	P74	I11	Post-cholecystectomy	Control group
0.9050343249427918
1627	P74	I13	Post-cholecystectomy	Control group
0.6127002288329519
1628	P74	I15	Post-cholecystectomy	Control group
0.721205186880244
1629	P74	I17	Post-cholecystectomy	Control group

0.8657513348588863
1630 P74 I18 Post-cholecystectomy Control group
0.665903890160183
1631 P74 I19 Post-cholecystectomy Control group
0.8947368421052632
1632 P74 I22 Post-cholecystectomy Control group
0.8089244851258581
1633 P74 I23 Post-cholecystectomy Control group
0.6887871853546911
1634 P74 I24 Post-cholecystectomy Control group
0.8674675819984744
1635 P74 I25 Post-cholecystectomy Control group
0.8220823798627003
1636 P74 I26 Post-cholecystectomy Control group
0.5409992372234935
1637 P74 I27 Post-cholecystectomy Control group
0.6903127383676583
1638 P74 I28 Post-cholecystectomy Control group
0.9057971014492754
1639 P74 I29 Post-cholecystectomy Control group
0.7942410373760488
1640 P74 I30 Post-cholecystectomy Control group
0.8041571319603357
1641 P74 I31 Post-cholecystectomy Control group
0.8558352402745996
1642 P74 I32 Post-cholecystectomy Control group
0.8548817696414951
1643 P74 I33 Post-cholecystectomy Control group
0.8304729214340199
1644 P74 I34 Post-cholecystectomy Control group
0.8493516399694889
1645 P74 I35 Post-cholecystectomy Control group
0.818649885583524
1646 P74 I36 Post-cholecystectomy Control group
0.9630053394355453
1647 P74 I37 Post-cholecystectomy Control group
0.8943554538520213
1648 P75 B27 Post-cholecystectomy Control group
0.7740274599542334
1649 P75 B66 Post-cholecystectomy Control group
0.8115942028985508
1650 P75 B86 Post-cholecystectomy Control group
0.7829900839054157
1651 P75 B97 Post-cholecystectomy Control group
0.732837528604119
1652 P75 B98 Post-cholecystectomy Control group
0.7229214340198322
1653 P75 B100 Post-cholecystectomy Control group
0.9382151029748284
1654 P75 B112 Post-cholecystectomy Control group
0.8426773455377574
1655 P75 B115 Post-cholecystectomy Control group
0.6935545385202135
1656 P75 I1 Post-cholecystectomy Control group

0.6981311975591151
1657 P75 I3 Post-cholecystectomy Control group
0.6905034324942791
1658 P75 I6 Post-cholecystectomy Control group
0.7200610221205187
1659 P75 I8 Post-cholecystectomy Control group
0.7765064836003052
1660 P75 I10 Post-cholecystectomy Control group
0.7536231884057971
1661 P75 I11 Post-cholecystectomy Control group
0.8382913806254767
1662 P75 I13 Post-cholecystectomy Control group
0.7829900839054157
1663 P75 I15 Post-cholecystectomy Control group
0.856788710907704
1664 P75 I17 Post-cholecystectomy Control group
0.8175057208237986
1665 P75 I18 Post-cholecystectomy Control group
0.5875286041189931
1666 P75 I19 Post-cholecystectomy Control group
0.8461098398169337
1667 P75 I22 Post-cholecystectomy Control group
0.7753623188405797
1668 P75 I23 Post-cholecystectomy Control group
0.7139588100686499
1669 P75 I24 Post-cholecystectomy Control group
0.813119755911518
1670 P75 I25 Post-cholecystectomy Control group
0.7288329519450801
1671 P75 I26 Post-cholecystectomy Control group
0.7860411899313501
1672 P75 I27 Post-cholecystectomy Control group
0.5903890160183066
1673 P75 I28 Post-cholecystectomy Control group
0.8422959572845157
1674 P75 I29 Post-cholecystectomy Control group
0.809115179252479
1675 P75 I30 Post-cholecystectomy Control group
0.755720823798627
1676 P75 I31 Post-cholecystectomy Control group
0.7894736842105263
1677 P75 I32 Post-cholecystectomy Control group
0.8192219679633868
1678 P75 I33 Post-cholecystectomy Control group
0.7902364607170099
1679 P75 I34 Post-cholecystectomy Control group
0.7936689549961862
1680 P75 I35 Post-cholecystectomy Control group
0.7559115179252479
1681 P75 I36 Post-cholecystectomy Control group
0.9975209763539283
1682 P75 I37 Post-cholecystectomy Control group
0.8783371472158658
1683 P1 B1 Post-cholecystectomy Idiopathic BAD

0.8483981693363845
1684 P1 B5 Post-cholecystectomy Idiopathic BAD
0.9107551487414187
1685 P1 B6 Post-cholecystectomy Idiopathic BAD
0.9401220442410374
1686 P1 B10 Post-cholecystectomy Idiopathic BAD
0.8432494279176201
1687 P1 B17 Post-cholecystectomy Idiopathic BAD
0.9631960335621663
1688 P1 B20 Post-cholecystectomy Idiopathic BAD
0.9631960335621663
1689 P1 B23 Post-cholecystectomy Idiopathic BAD
0.9490846681922197
1690 P1 B31 Post-cholecystectomy Idiopathic BAD
0.8577421815408085
1691 P1 B35 Post-cholecystectomy Idiopathic BAD
0.9824561403508771
1692 P1 B39 Post-cholecystectomy Idiopathic BAD
0.9178108314263921
1693 P1 B43 Post-cholecystectomy Idiopathic BAD
0.9628146453089245
1694 P1 B47 Post-cholecystectomy Idiopathic BAD
0.9559496567505721
1695 P1 B48 Post-cholecystectomy Idiopathic BAD
0.9761632341723875
1696 P1 B49 Post-cholecystectomy Idiopathic BAD
0.9124713958810069
1697 P1 B53 Post-cholecystectomy Idiopathic BAD
0.9622425629290617
1698 P1 B54 Post-cholecystectomy Idiopathic BAD
0.9744469870327994
1699 P1 B55 Post-cholecystectomy Idiopathic BAD
0.984744469870328
1700 P1 B59 Post-cholecystectomy Idiopathic BAD
0.8535469107551488
1701 P1 B70 Post-cholecystectomy Idiopathic BAD
0.979023646071701
1702 P1 B74 Post-cholecystectomy Idiopathic BAD
0.9652936689549961
1703 P1 B77 Post-cholecystectomy Idiopathic BAD
0.8844393592677345
1704 P1 B81 Post-cholecystectomy Idiopathic BAD
0.876048817696415
1705 P1 B84 Post-cholecystectomy Idiopathic BAD
0.9010297482837528
1706 P1 B89 Post-cholecystectomy Idiopathic BAD
0.9584286803966438
1707 P1 B92 Post-cholecystectomy Idiopathic BAD
0.9216247139588101
1708 P1 B95 Post-cholecystectomy Idiopathic BAD
0.9605263157894737
1709 P1 B99 Post-cholecystectomy Idiopathic BAD
0.950228832951945
1710 P1 B103 Post-cholecystectomy Idiopathic BAD

0.9889397406559878
1711	P1	B106	Post-cholecystectomy	Idiopathic BAD
0.9740655987795576
1712	P1	B109	Post-cholecystectomy	Idiopathic BAD
0.9744469870327994
1713	P1	B118	Post-cholecystectomy	Idiopathic BAD
0.971395881006865
1714	P1	B119	Post-cholecystectomy	Idiopathic BAD
0.9075133485888635
1715	P2	B1	Post-cholecystectomy	Idiopathic BAD
0.8945461479786423
1716	P2	B5	Post-cholecystectomy	Idiopathic BAD
0.8159801678108314
1717	P2	B6	Post-cholecystectomy	Idiopathic BAD
0.8825324180015256
1718	P2	B10	Post-cholecystectomy	Idiopathic BAD
0.8377192982456141
1719	P2	B17	Post-cholecystectomy	Idiopathic BAD
0.7953852021357742
1720	P2	B20	Post-cholecystectomy	Idiopathic BAD
0.7934782608695652
1721	P2	B23	Post-cholecystectomy	Idiopathic BAD
0.7490465293668955
1722	P2	B31	Post-cholecystectomy	Idiopathic BAD
0.8415331807780321
1723	P2	B35	Post-cholecystectomy	Idiopathic BAD
0.9845537757437071
1724	P2	B39	Post-cholecystectomy	Idiopathic BAD
0.8537376048817696
1725	P2	B43	Post-cholecystectomy	Idiopathic BAD
0.7934782608695652
1726	P2	B47	Post-cholecystectomy	Idiopathic BAD
0.7854691075514875
1727	P2	B48	Post-cholecystectomy	Idiopathic BAD
0.9605263157894737
1728	P2	B49	Post-cholecystectomy	Idiopathic BAD
0.8442028985507246
1729	P2	B53	Post-cholecystectomy	Idiopathic BAD
0.8062547673531655
1730	P2	B54	Post-cholecystectomy	Idiopathic BAD
0.8804347826086957
1731	P2	B55	Post-cholecystectomy	Idiopathic BAD
0.8699466056445462
1732	P2	B59	Post-cholecystectomy	Idiopathic BAD
0.9464149504195271
1733	P2	B70	Post-cholecystectomy	Idiopathic BAD
0.7906178489702517
1734	P2	B74	Post-cholecystectomy	Idiopathic BAD
0.8251334858886347
1735	P2	B77	Post-cholecystectomy	Idiopathic BAD
0.7833714721586575
1736	P2	B81	Post-cholecystectomy	Idiopathic BAD
0.8861556064073226
1737	P2	B84	Post-cholecystectomy	Idiopathic BAD

0.7677345537757437
1738 P2 B89 Post-cholecystectomy Idiopathic BAD
0.7967200610221206
1739 P2 B92 Post-cholecystectomy Idiopathic BAD
0.7479023646071701
1740 P2 B95 Post-cholecystectomy Idiopathic BAD
0.8779557589626239
1741 P2 B99 Post-cholecystectomy Idiopathic BAD
0.7145308924485125
1742 P2 B103 Post-cholecystectomy Idiopathic BAD
0.9479405034324943
1743 P2 B106 Post-cholecystectomy Idiopathic BAD
0.9225781845919145
1744 P2 B109 Post-cholecystectomy Idiopathic BAD
0.8114035087719298
1745 P2 B118 Post-cholecystectomy Idiopathic BAD
0.8325705568268498
1746 P2 B119 Post-cholecystectomy Idiopathic BAD
0.8243707093821511
1747 P4 B1 Post-cholecystectomy Idiopathic BAD
0.7416094584286804
1748 P4 B5 Post-cholecystectomy Idiopathic BAD
0.7644927536231884
1749 P4 B6 Post-cholecystectomy Idiopathic BAD
0.855072463768116
1750 P4 B10 Post-cholecystectomy Idiopathic BAD
0.8119755911517925
1751 P4 B17 Post-cholecystectomy Idiopathic BAD
0.7377955758962624
1752 P4 B20 Post-cholecystectomy Idiopathic BAD
0.8497330282227308
1753 P4 B23 Post-cholecystectomy Idiopathic BAD
0.7917620137299771
1754 P4 B31 Post-cholecystectomy Idiopathic BAD
0.7555301296720061
1755 P4 B35 Post-cholecystectomy Idiopathic BAD
0.8485888634630053
1756 P4 B39 Post-cholecystectomy Idiopathic BAD
0.8792906178489702
1757 P4 B43 Post-cholecystectomy Idiopathic BAD
0.8766209000762777
1758 P4 B47 Post-cholecystectomy Idiopathic BAD
0.809115179252479
1759 P4 B48 Post-cholecystectomy Idiopathic BAD
0.704233409610984
1760 P4 B49 Post-cholecystectomy Idiopathic BAD
0.7341723874904653
1761 P4 B53 Post-cholecystectomy Idiopathic BAD
0.856788710907704
1762 P4 B54 Post-cholecystectomy Idiopathic BAD
0.8575514874141876
1763 P4 B55 Post-cholecystectomy Idiopathic BAD
0.9509916094584286
1764 P4 B59 Post-cholecystectomy Idiopathic BAD

0.9822654462242563
1765	P4	B70	Post-cholecystectomy	Idiopathic BAD
0.8749046529366895
1766	P4	B74	Post-cholecystectomy	Idiopathic BAD
0.8676582761250954
1767	P4	B77	Post-cholecystectomy	Idiopathic BAD
0.8852021357742181
1768	P4	B81	Post-cholecystectomy	Idiopathic BAD
0.7972921434019832
1769	P4	B84	Post-cholecystectomy	Idiopathic BAD
0.8659420289855072
1770	P4	B89	Post-cholecystectomy	Idiopathic BAD
0.8022501906941266
1771	P4	B92	Post-cholecystectomy	Idiopathic BAD
0.7648741418764302
1772	P4	B95	Post-cholecystectomy	Idiopathic BAD
0.84744469870328
1773	P4	B99	Post-cholecystectomy	Idiopathic BAD
0.6950800915331807
1774	P4	B103	Post-cholecystectomy	Idiopathic BAD
0.8993135011441648
1775	P4	B106	Post-cholecystectomy	Idiopathic BAD
0.9439359267734554
1776	P4	B109	Post-cholecystectomy	Idiopathic BAD
0.8653699466056446
1777	P4	B118	Post-cholecystectomy	Idiopathic BAD
0.8821510297482837
1778	P4	B119	Post-cholecystectomy	Idiopathic BAD
0.7732646834477498
1779	P5	B1	Post-cholecystectomy	Idiopathic BAD
0.8354309687261632
1780	P5	B5	Post-cholecystectomy	Idiopathic BAD
0.7587719298245614
1781	P5	B6	Post-cholecystectomy	Idiopathic BAD
0.8325705568268498
1782	P5	B10	Post-cholecystectomy	Idiopathic BAD
0.7942410373760488
1783	P5	B17	Post-cholecystectomy	Idiopathic BAD
0.7459954233409611
1784	P5	B20	Post-cholecystectomy	Idiopathic BAD
0.7923340961098398
1785	P5	B23	Post-cholecystectomy	Idiopathic BAD
0.698512585812357
1786	P5	B31	Post-cholecystectomy	Idiopathic BAD
0.7852784134248665
1787	P5	B35	Post-cholecystectomy	Idiopathic BAD
0.9456521739130435
1788	P5	B39	Post-cholecystectomy	Idiopathic BAD
0.8255148741418764
1789	P5	B43	Post-cholecystectomy	Idiopathic BAD
0.835812356979405
1790	P5	B47	Post-cholecystectomy	Idiopathic BAD
0.6826849733028223
1791	P5	B48	Post-cholecystectomy	Idiopathic BAD

0.8182684973302822
1792 P5 B49 Post−cholecystectomy Idiopathic BAD
0.8119755911517925
1793 P5 B53 Post−cholecystectomy Idiopathic BAD
0.7776506483600305
1794 P5 B54 Post−cholecystectomy Idiopathic BAD
0.8581235697940504
1795 P5 B55 Post−cholecystectomy Idiopathic BAD
0.8598398169336384
1796 P5 B59 Post−cholecystectomy Idiopathic BAD
0.9691075514874142
1797 P5 B70 Post−cholecystectomy Idiopathic BAD
0.8056826849733029
1798 P5 B74 Post−cholecystectomy Idiopathic BAD
0.8173150266971777
1799 P5 B77 Post−cholecystectomy Idiopathic BAD
0.7345537757437071
1800 P5 B81 Post−cholecystectomy Idiopathic BAD
0.8270404271548436
1801 P5 B84 Post−cholecystectomy Idiopathic BAD
0.7873760488176964
1802 P5 B89 Post−cholecystectomy Idiopathic BAD
0.8432494279176201
1803 P5 B92 Post−cholecystectomy Idiopathic BAD
0.7717391304347826
1804 P5 B95 Post−cholecystectomy Idiopathic BAD
0.8409610983981693
1805 P5 B99 Post−cholecystectomy Idiopathic BAD
0.7109077040427155
1806 P5 B103 Post−cholecystectomy Idiopathic BAD
0.8430587337909993
1807 P5 B106 Post−cholecystectomy Idiopathic BAD
0.9153318077803204
1808 P5 B109 Post−cholecystectomy Idiopathic BAD
0.7444698703279939
1809 P5 B118 Post−cholecystectomy Idiopathic BAD
0.8712814645308925
1810 P5 B119 Post−cholecystectomy Idiopathic BAD
0.6885964912280702
1811 P9 B1 Post−cholecystectomy Idiopathic BAD
0.765255530129672
1812 P9 B5 Post−cholecystectomy Idiopathic BAD
0.8316170861937452
1813 P9 B6 Post−cholecystectomy Idiopathic BAD
0.9084668192219679
1814 P9 B10 Post−cholecystectomy Idiopathic BAD
0.8274218154080855
1815 P9 B17 Post−cholecystectomy Idiopathic BAD
0.7980549199084668
1816 P9 B20 Post−cholecystectomy Idiopathic BAD
0.9023646071700991
1817 P9 B23 Post−cholecystectomy Idiopathic BAD
0.7044241037376049
1818 P9 B31 Post−cholecystectomy Idiopathic BAD

0.6651411136536994
1819 P9 B35 Post-cholecystectomy Idiopathic BAD
0.9664378337147216
1820 P9 B39 Post-cholecystectomy Idiopathic BAD
0.8260869565217391
1821 P9 B43 Post-cholecystectomy Idiopathic BAD
0.9391685736079328
1822 P9 B47 Post-cholecystectomy Idiopathic BAD
0.7250190694126621
1823 P9 B48 Post-cholecystectomy Idiopathic BAD
0.7492372234935164
1824 P9 B49 Post-cholecystectomy Idiopathic BAD
0.9046529366895499
1825 P9 B53 Post-cholecystectomy Idiopathic BAD
0.7238749046529367
1826 P9 B54 Post-cholecystectomy Idiopathic BAD
0.8443935926773455
1827 P9 B55 Post-cholecystectomy Idiopathic BAD
0.8201754385964912
1828 P9 B59 Post-cholecystectomy Idiopathic BAD
0.9933257055682685
1829 P9 B70 Post-cholecystectomy Idiopathic BAD
0.7107170099160945
1830 P9 B74 Post-cholecystectomy Idiopathic BAD
0.9096109839816934
1831 P9 B77 Post-cholecystectomy Idiopathic BAD
0.7650648360030511
1832 P9 B81 Post-cholecystectomy Idiopathic BAD
0.873951182303585
1833 P9 B84 Post-cholecystectomy Idiopathic BAD
0.8594584286803967
1834 P9 B89 Post-cholecystectomy Idiopathic BAD
0.8901601830663616
1835 P9 B92 Post-cholecystectomy Idiopathic BAD
0.7986270022883295
1836 P9 B95 Post-cholecystectomy Idiopathic BAD
0.894927536231884
1837 P9 B99 Post-cholecystectomy Idiopathic BAD
0.791952707856598
1838 P9 B103 Post-cholecystectomy Idiopathic BAD
0.9174294431731502
1839 P9 B106 Post-cholecystectomy Idiopathic BAD
0.9624332570556827
1840 P9 B109 Post-cholecystectomy Idiopathic BAD
0.790045766590389
1841 P9 B118 Post-cholecystectomy Idiopathic BAD
0.9145690312738368
1842 P9 B119 Post-cholecystectomy Idiopathic BAD
0.6584668192219679
1843 P13 B1 Post-cholecystectomy Idiopathic BAD
0.9042715484363082
1844 P13 B5 Post-cholecystectomy Idiopathic BAD
0.8546910755148741
1845 P13 B6 Post-cholecystectomy Idiopathic BAD

0.7305491990846682
1846 P13 B10 Post-cholecystectomy Idiopathic BAD
0.9256292906178489
1847 P13 B17 Post-cholecystectomy Idiopathic BAD
0.8726163234172387
1848 P13 B20 Post-cholecystectomy Idiopathic BAD
0.8163615560640732
1849 P13 B23 Post-cholecystectomy Idiopathic BAD
0.8716628527841342
1850 P13 B31 Post-cholecystectomy Idiopathic BAD
0.8771929824561403
1851 P13 B35 Post-cholecystectomy Idiopathic BAD
0.9668192219679634
1852 P13 B39 Post-cholecystectomy Idiopathic BAD
0.8703279938977879
1853 P13 B43 Post-cholecystectomy Idiopathic BAD
0.6096491228070176
1854 P13 B47 Post-cholecystectomy Idiopathic BAD
0.8573607932875668
1855 P13 B48 Post-cholecystectomy Idiopathic BAD
0.9145690312738368
1856 P13 B49 Post-cholecystectomy Idiopathic BAD
0.7768878718535469
1857 P13 B53 Post-cholecystectomy Idiopathic BAD
0.8499237223493517
1858 P13 B54 Post-cholecystectomy Idiopathic BAD
0.9218154080854309
1859 P13 B55 Post-cholecystectomy Idiopathic BAD
0.950228832951945
1860 P13 B59 Post-cholecystectomy Idiopathic BAD
0.9872234935163997
1861 P13 B70 Post-cholecystectomy Idiopathic BAD
0.9380244088482075
1862 P13 B74 Post-cholecystectomy Idiopathic BAD
0.9338291380625476
1863 P13 B77 Post-cholecystectomy Idiopathic BAD
0.8802440884820748
1864 P13 B81 Post-cholecystectomy Idiopathic BAD
0.8400076277650649
1865 P13 B84 Post-cholecystectomy Idiopathic BAD
0.8150266971777269
1866 P13 B89 Post-cholecystectomy Idiopathic BAD
0.795957284515637
1867 P13 B92 Post-cholecystectomy Idiopathic BAD
0.8409610983981693
1868 P13 B95 Post-cholecystectomy Idiopathic BAD
0.8257055682684973
1869 P13 B99 Post-cholecystectomy Idiopathic BAD
0.6668573607932876
1870 P13 B103 Post-cholecystectomy Idiopathic BAD
0.8272311212814645
1871 P13 B106 Post-cholecystectomy Idiopathic BAD
0.9420289855072463
1872 P13 B109 Post-cholecystectomy Idiopathic BAD

0.8878718535469108
1873	P13	B118	Post-cholecystectomy	Idiopathic BAD
0.8297101449275363
1874	P13	B119	Post-cholecystectomy	Idiopathic BAD
0.7803203661327232
1875	P15	B1	Post-cholecystectomy	Idiopathic BAD
0.8030129672006102
1876	P15	B5	Post-cholecystectomy	Idiopathic BAD
0.7799389778794813
1877	P15	B6	Post-cholecystectomy	Idiopathic BAD
0.8686117467581999
1878	P15	B10	Post-cholecystectomy	Idiopathic BAD
0.7875667429443173
1879	P15	B17	Post-cholecystectomy	Idiopathic BAD
0.5575896262395118
1880	P15	B20	Post-cholecystectomy	Idiopathic BAD
0.6735316552250191
1881	P15	B23	Post-cholecystectomy	Idiopathic BAD
0.7509534706331045
1882	P15	B31	Post-cholecystectomy	Idiopathic BAD
0.7437070938215103
1883	P15	B35	Post-cholecystectomy	Idiopathic BAD
0.9324942791762014
1884	P15	B39	Post-cholecystectomy	Idiopathic BAD
0.84744469870328
1885	P15	B43	Post-cholecystectomy	Idiopathic BAD
0.7763157894736842
1886	P15	B47	Post-cholecystectomy	Idiopathic BAD
0.8197940503432495
1887	P15	B48	Post-cholecystectomy	Idiopathic BAD
0.8632723112128147
1888	P15	B49	Post-cholecystectomy	Idiopathic BAD
0.8226544622425629
1889	P15	B53	Post-cholecystectomy	Idiopathic BAD
0.8661327231121282
1890	P15	B54	Post-cholecystectomy	Idiopathic BAD
0.709954233409611
1891	P15	B55	Post-cholecystectomy	Idiopathic BAD
0.7608695652173914
1892	P15	B59	Post-cholecystectomy	Idiopathic BAD
0.9454614797864226
1893	P15	B70	Post-cholecystectomy	Idiopathic BAD
0.9326849733028223
1894	P15	B74	Post-cholecystectomy	Idiopathic BAD
0.78813882532418
1895	P15	B77	Post-cholecystectomy	Idiopathic BAD
0.852974828375286
1896	P15	B81	Post-cholecystectomy	Idiopathic BAD
0.8537376048817696
1897	P15	B84	Post-cholecystectomy	Idiopathic BAD
0.8951182303585049
1898	P15	B89	Post-cholecystectomy	Idiopathic BAD
0.8264683447749809
1899	P15	B92	Post-cholecystectomy	Idiopathic BAD

0.7019450800915332
1900	P15	B95	Post-cholecystectomy	Idiopathic BAD
0.7551487414187643
1901	P15	B99	Post-cholecystectomy	Idiopathic BAD
0.7782227307398932
1902	P15	B103	Post-cholecystectomy	Idiopathic BAD
0.9170480549199085
1903	P15	B106	Post-cholecystectomy	Idiopathic BAD
0.6763920671243325
1904	P15	B109	Post-cholecystectomy	Idiopathic BAD
0.9057971014492754
1905	P15	B118	Post-cholecystectomy	Idiopathic BAD
0.7662090007627765
1906	P15	B119	Post-cholecystectomy	Idiopathic BAD
0.8234172387490465
1907	P16	B1	Post-cholecystectomy	Idiopathic BAD
0.8030129672006102
1908	P16	B5	Post-cholecystectomy	Idiopathic BAD
0.8161708619374524
1909	P16	B6	Post-cholecystectomy	Idiopathic BAD
0.933066361556064
1910	P16	B10	Post-cholecystectomy	Idiopathic BAD
0.8827231121281465
1911	P16	B17	Post-cholecystectomy	Idiopathic BAD
0.8361937452326468
1912	P16	B20	Post-cholecystectomy	Idiopathic BAD
0.8905415713196033
1913	P16	B23	Post-cholecystectomy	Idiopathic BAD
0.7734553775743707
1914	P16	B31	Post-cholecystectomy	Idiopathic BAD
0.834096109839817
1915	P16	B35	Post-cholecystectomy	Idiopathic BAD
0.8205568268497331
1916	P16	B39	Post-cholecystectomy	Idiopathic BAD
0.8834858886346301
1917	P16	B43	Post-cholecystectomy	Idiopathic BAD
0.9574752097635393
1918	P16	B47	Post-cholecystectomy	Idiopathic BAD
0.8180778032036613
1919	P16	B48	Post-cholecystectomy	Idiopathic BAD
0.6493135011441648
1920	P16	B49	Post-cholecystectomy	Idiopathic BAD
0.7288329519450801
1921	P16	B53	Post-cholecystectomy	Idiopathic BAD
0.8527841342486652
1922	P16	B54	Post-cholecystectomy	Idiopathic BAD
0.8991228070175439
1923	P16	B55	Post-cholecystectomy	Idiopathic BAD
0.8861556064073226
1924	P16	B59	Post-cholecystectomy	Idiopathic BAD
0.9609077040427155
1925	P16	B70	Post-cholecystectomy	Idiopathic BAD
0.8009153318077803
1926	P16	B74	Post-cholecystectomy	Idiopathic BAD

0.8754767353165522
1927	P16	B77	Post-cholecystectomy	Idiopathic BAD
0.8394355453852022
1928	P16	B81	Post-cholecystectomy	Idiopathic BAD
0.8590770404271548
1929	P16	B84	Post-cholecystectomy	Idiopathic BAD
0.8525934401220442
1930	P16	B89	Post-cholecystectomy	Idiopathic BAD
0.8411517925247902
1931	P16	B92	Post-cholecystectomy	Idiopathic BAD
0.8253241800152555
1932	P16	B95	Post-cholecystectomy	Idiopathic BAD
0.8342868039664378
1933	P16	B99	Post-cholecystectomy	Idiopathic BAD
0.7356979405034325
1934	P16	B103	Post-cholecystectomy	Idiopathic BAD
0.914187643020595
1935	P16	B106	Post-cholecystectomy	Idiopathic BAD
0.9469870327993898
1936	P16	B109	Post-cholecystectomy	Idiopathic BAD
0.7139588100686499
1937	P16	B118	Post-cholecystectomy	Idiopathic BAD
0.940884820747521
1938	P16	B119	Post-cholecystectomy	Idiopathic BAD
0.7725019069412662
1939	P17	B1	Post-cholecystectomy	Idiopathic BAD
0.9092295957284515
1940	P17	B5	Post-cholecystectomy	Idiopathic BAD
0.8585049580472921
1941	P17	B6	Post-cholecystectomy	Idiopathic BAD
0.9012204424103738
1942	P17	B10	Post-cholecystectomy	Idiopathic BAD
0.9317315026697178
1943	P17	B17	Post-cholecystectomy	Idiopathic BAD
0.8318077803203662
1944	P17	B20	Post-cholecystectomy	Idiopathic BAD
0.8094965675057209
1945	P17	B23	Post-cholecystectomy	Idiopathic BAD
0.8382913806254767
1946	P17	B31	Post-cholecystectomy	Idiopathic BAD
0.9139969488939741
1947	P17	B35	Post-cholecystectomy	Idiopathic BAD
0.994279176201373
1948	P17	B39	Post-cholecystectomy	Idiopathic BAD
0.8504958047292144
1949	P17	B43	Post-cholecystectomy	Idiopathic BAD
0.8453470633104501
1950	P17	B47	Post-cholecystectomy	Idiopathic BAD
0.8083524027459954
1951	P17	B48	Post-cholecystectomy	Idiopathic BAD
0.9563310450038138
1952	P17	B49	Post-cholecystectomy	Idiopathic BAD
0.7889016018306636
1953	P17	B53	Post-cholecystectomy	Idiopathic BAD

0.8081617086193745
1954 P17 B54 Post−cholecystectomy Idiopathic BAD
0.9155225019069413
1955 P17 B55 Post−cholecystectomy Idiopathic BAD
0.9073226544622426
1956 P17 B59 Post−cholecystectomy Idiopathic BAD
0.9872234935163997
1957 P17 B70 Post−cholecystectomy Idiopathic BAD
0.8661327231121282
1958 P17 B74 Post−cholecystectomy Idiopathic BAD
0.8926392067124332
1959 P17 B77 Post−cholecystectomy Idiopathic BAD
0.8426773455377574
1960 P17 B81 Post−cholecystectomy Idiopathic BAD
0.7936689549961862
1961 P17 B84 Post−cholecystectomy Idiopathic BAD
0.8239893211289092
1962 P17 B89 Post−cholecystectomy Idiopathic BAD
0.8133104500381388
1963 P17 B92 Post−cholecystectomy Idiopathic BAD
0.8260869565217391
1964 P17 B95 Post−cholecystectomy Idiopathic BAD
0.8579328756674295
1965 P17 B99 Post−cholecystectomy Idiopathic BAD
0.7429443173150267
1966 P17 B103 Post−cholecystectomy Idiopathic BAD
0.9040808543096872
1967 P17 B106 Post−cholecystectomy Idiopathic BAD
0.96186117467582
1968 P17 B109 Post−cholecystectomy Idiopathic BAD
0.8251334858886347
1969 P17 B118 Post−cholecystectomy Idiopathic BAD
0.8716628527841342
1970 P17 B119 Post−cholecystectomy Idiopathic BAD
0.7677345537757437
1971 P20 B1 Post−cholecystectomy Idiopathic BAD
0.7467581998474447
1972 P20 B5 Post−cholecystectomy Idiopathic BAD
0.6864988558352403
1973 P20 B6 Post−cholecystectomy Idiopathic BAD
0.7745995423340961
1974 P20 B10 Post−cholecystectomy Idiopathic BAD
0.8333333333333334
1975 P20 B17 Post−cholecystectomy Idiopathic BAD
0.8516399694889397
1976 P20 B20 Post−cholecystectomy Idiopathic BAD
0.8413424866514111
1977 P20 B23 Post−cholecystectomy Idiopathic BAD
0.8136918382913806
1978 P20 B31 Post−cholecystectomy Idiopathic BAD
0.7265446224256293
1979 P20 B35 Post−cholecystectomy Idiopathic BAD
0.6279557589626239
1980 P20 B39 Post−cholecystectomy Idiopathic BAD

0.7827993897787948
1981	P20	B43	Post-cholecystectomy	Idiopathic BAD
0.822463768115942
1982	P20	B47	Post-cholecystectomy	Idiopathic BAD
0.8190312738367659
1983	P20	B48	Post-cholecystectomy	Idiopathic BAD
0.5783752860411899
1984	P20	B49	Post-cholecystectomy	Idiopathic BAD
0.6512204424103738
1985	P20	B53	Post-cholecystectomy	Idiopathic BAD
0.8747139588100686
1986	P20	B54	Post-cholecystectomy	Idiopathic BAD
0.8874904652936689
1987	P20	B55	Post-cholecystectomy	Idiopathic BAD
0.9258199847444699
1988	P20	B59	Post-cholecystectomy	Idiopathic BAD
0.9096109839816934
1989	P20	B70	Post-cholecystectomy	Idiopathic BAD
0.8537376048817696
1990	P20	B74	Post-cholecystectomy	Idiopathic BAD
0.9405034324942791
1991	P20	B77	Post-cholecystectomy	Idiopathic BAD
0.8794813119755912
1992	P20	B81	Post-cholecystectomy	Idiopathic BAD
0.843440122044241
1993	P20	B84	Post-cholecystectomy	Idiopathic BAD
0.8154080854309688
1994	P20	B89	Post-cholecystectomy	Idiopathic BAD
0.7307398932112891
1995	P20	B92	Post-cholecystectomy	Idiopathic BAD
0.780511060259344
1996	P20	B95	Post-cholecystectomy	Idiopathic BAD
0.7395118230358505
1997	P20	B99	Post-cholecystectomy	Idiopathic BAD
0.6498855835240275
1998	P20	B103	Post-cholecystectomy	Idiopathic BAD
0.8176964149504196
1999	P20	B106	Post-cholecystectomy	Idiopathic BAD
0.9469870327993898
2000	P20	B109	Post-cholecystectomy	Idiopathic BAD
0.8787185354691075
2001	P20	B118	Post-cholecystectomy	Idiopathic BAD
0.814836003051106
2002	P20	B119	Post-cholecystectomy	Idiopathic BAD
0.7374141876430206
2003	P21	B1	Post-cholecystectomy	Idiopathic BAD
0.8909229595728452
2004	P21	B5	Post-cholecystectomy	Idiopathic BAD
0.6727688787185355
2005	P21	B6	Post-cholecystectomy	Idiopathic BAD
0.8968344774980931
2006	P21	B10	Post-cholecystectomy	Idiopathic BAD
0.9487032799389779
2007	P21	B17	Post-cholecystectomy	Idiopathic BAD

0.8457284515636918
2008 P21 B20 Post-cholecystectomy Idiopathic BAD
0.8194126620900076
2009 P21 B23 Post-cholecystectomy Idiopathic BAD
0.7332189168573608
2010 P21 B31 Post-cholecystectomy Idiopathic BAD
0.8281845919145691
2011 P21 B35 Post-cholecystectomy Idiopathic BAD
0.8848207475209764
2012 P21 B39 Post-cholecystectomy Idiopathic BAD
0.812929061784897
2013 P21 B43 Post-cholecystectomy Idiopathic BAD
0.8705186880244088
2014 P21 B47 Post-cholecystectomy Idiopathic BAD
0.8070175438596491
2015 P21 B48 Post-cholecystectomy Idiopathic BAD
0.6691456903127384
2016 P21 B49 Post-cholecystectomy Idiopathic BAD
0.9052250190694127
2017 P21 B53 Post-cholecystectomy Idiopathic BAD
0.8308543096872616
2018 P21 B54 Post-cholecystectomy Idiopathic BAD
0.8712814645308925
2019 P21 B55 Post-cholecystectomy Idiopathic BAD
0.8724256292906178
2020 P21 B59 Post-cholecystectomy Idiopathic BAD
0.9849351639969489
2021 P21 B70 Post-cholecystectomy Idiopathic BAD
0.7065217391304348
2022 P21 B74 Post-cholecystectomy Idiopathic BAD
0.9176201372997712
2023 P21 B77 Post-cholecystectomy Idiopathic BAD
0.8117848970251716
2024 P21 B81 Post-cholecystectomy Idiopathic BAD
0.864607170099161
2025 P21 B84 Post-cholecystectomy Idiopathic BAD
0.7200610221205187
2026 P21 B89 Post-cholecystectomy Idiopathic BAD
0.8239893211289092
2027 P21 B92 Post-cholecystectomy Idiopathic BAD
0.7276887871853547
2028 P21 B95 Post-cholecystectomy Idiopathic BAD
0.851067887109077
2029 P21 B99 Post-cholecystectomy Idiopathic BAD
0.7587719298245614
2030 P21 B103 Post-cholecystectomy Idiopathic BAD
0.8901601830663616
2031 P21 B106 Post-cholecystectomy Idiopathic BAD
0.9614797864225781
2032 P21 B109 Post-cholecystectomy Idiopathic BAD
0.7570556826849733
2033 P21 B118 Post-cholecystectomy Idiopathic BAD
0.8872997711670481
2034 P21 B119 Post-cholecystectomy Idiopathic BAD

0.662090007627765
2035	P24	B1	Post−cholecystectomy	Idiopathic BAD
0.927536231884058
2036	P24	B5	Post−cholecystectomy	Idiopathic BAD
0.8995041952707856
2037	P24	B6	Post−cholecystectomy	Idiopathic BAD
0.7692601067887109
2038	P24	B10	Post−cholecystectomy	Idiopathic BAD
0.8390541571319603
2039	P24	B17	Post−cholecystectomy	Idiopathic BAD
0.8135011441647597
2040	P24	B20	Post−cholecystectomy	Idiopathic BAD
0.8712814645308925
2041	P24	B23	Post−cholecystectomy	Idiopathic BAD
0.8636536994660564
2042	P24	B31	Post−cholecystectomy	Idiopathic BAD
0.8487795575896262
2043	P24	B35	Post−cholecystectomy	Idiopathic BAD
0.9729214340198322
2044	P24	B39	Post−cholecystectomy	Idiopathic BAD
0.8966437833714722
2045	P24	B43	Post−cholecystectomy	Idiopathic BAD
0.7589626239511823
2046	P24	B47	Post−cholecystectomy	Idiopathic BAD
0.8548817696414951
2047	P24	B48	Post−cholecystectomy	Idiopathic BAD
0.9441266209000763
2048	P24	B49	Post−cholecystectomy	Idiopathic BAD
0.7465675057208238
2049	P24	B53	Post−cholecystectomy	Idiopathic BAD
0.8954996186117468
2050	P24	B54	Post−cholecystectomy	Idiopathic BAD
0.9528985507246377
2051	P24	B55	Post−cholecystectomy	Idiopathic BAD
0.9637681159420289
2052	P24	B59	Post−cholecystectomy	Idiopathic BAD
0.9782608695652174
2053	P24	B70	Post−cholecystectomy	Idiopathic BAD
0.9405034324942791
2054	P24	B74	Post−cholecystectomy	Idiopathic BAD
0.7759344012204424
2055	P24	B77	Post−cholecystectomy	Idiopathic BAD
0.8657513348588863
2056	P24	B81	Post−cholecystectomy	Idiopathic BAD
0.7990083905415714
2057	P24	B84	Post−cholecystectomy	Idiopathic BAD
0.893211289092296
2058	P24	B89	Post−cholecystectomy	Idiopathic BAD
0.860602593440122
2059	P24	B92	Post−cholecystectomy	Idiopathic BAD
0.8668954996186118
2060	P24	B95	Post−cholecystectomy	Idiopathic BAD
0.8779557589626239
2061	P24	B99	Post−cholecystectomy	Idiopathic BAD

0.7475209763539283
2062 P24 B103 Post-cholecystectomy Idiopathic BAD
0.9361174675819984
2063 P24 B106 Post-cholecystectomy Idiopathic BAD
0.9391685736079328
2064 P24 B109 Post-cholecystectomy Idiopathic BAD
0.8771929824561403
2065 P24 B118 Post-cholecystectomy Idiopathic BAD
0.9063691838291381
2066 P24 B119 Post-cholecystectomy Idiopathic BAD
0.8382913806254767
2067 P26 B1 Post-cholecystectomy Idiopathic BAD
0.872234935163997
2068 P26 B5 Post-cholecystectomy Idiopathic BAD
0.7663996948893974
2069 P26 B6 Post-cholecystectomy Idiopathic BAD
0.8337147215865751
2070 P26 B10 Post-cholecystectomy Idiopathic BAD
0.9815026697177727
2071 P26 B17 Post-cholecystectomy Idiopathic BAD
0.8794813119755912
2072 P26 B20 Post-cholecystectomy Idiopathic BAD
0.8821510297482837
2073 P26 B23 Post-cholecystectomy Idiopathic BAD
0.681350114416476
2074 P26 B31 Post-cholecystectomy Idiopathic BAD
0.8667048054919908
2075 P26 B35 Post-cholecystectomy Idiopathic BAD
0.8226544622425629
2076 P26 B39 Post-cholecystectomy Idiopathic BAD
0.8882532418001525
2077 P26 B43 Post-cholecystectomy Idiopathic BAD
0.8493516399694889
2078 P26 B47 Post-cholecystectomy Idiopathic BAD
0.8466819221967964
2079 P26 B48 Post-cholecystectomy Idiopathic BAD
0.8392448512585813
2080 P26 B49 Post-cholecystectomy Idiopathic BAD
0.8070175438596491
2081 P26 B53 Post-cholecystectomy Idiopathic BAD
0.6664759725400458
2082 P26 B54 Post-cholecystectomy Idiopathic BAD
0.8468726163234173
2083 P26 B55 Post-cholecystectomy Idiopathic BAD
0.8874904652936689
2084 P26 B59 Post-cholecystectomy Idiopathic BAD
0.9832189168573608
2085 P26 B70 Post-cholecystectomy Idiopathic BAD
0.7124332570556827
2086 P26 B74 Post-cholecystectomy Idiopathic BAD
0.9319221967963387
2087 P26 B77 Post-cholecystectomy Idiopathic BAD
0.8184591914569032
2088 P26 B81 Post-cholecystectomy Idiopathic BAD

0.8548817696414951
2089	P26	B84	Post-cholecystectomy	Idiopathic BAD
0.7591533180778032
2090	P26	B89	Post-cholecystectomy	Idiopathic BAD
0.8615560640732265
2091	P26	B92	Post-cholecystectomy	Idiopathic BAD
0.8173150266971777
2092	P26	B95	Post-cholecystectomy	Idiopathic BAD
0.8556445461479787
2093	P26	B99	Post-cholecystectomy	Idiopathic BAD
0.7114797864225781
2094	P26	B103	Post-cholecystectomy	Idiopathic BAD
0.7879481311975591
2095	P26	B106	Post-cholecystectomy	Idiopathic BAD
0.9670099160945843
2096	P26	B109	Post-cholecystectomy	Idiopathic BAD
0.7036613272311213
2097	P26	B118	Post-cholecystectomy	Idiopathic BAD
0.8947368421052632
2098	P26	B119	Post-cholecystectomy	Idiopathic BAD
0.6571319603356217
2099	P30	B1	Post-cholecystectomy	Idiopathic BAD
0.8621281464530892
2100	P30	B5	Post-cholecystectomy	Idiopathic BAD
0.8943554538520213
2101	P30	B6	Post-cholecystectomy	Idiopathic BAD
0.9403127383676583
2102	P30	B10	Post-cholecystectomy	Idiopathic BAD
0.7656369183829138
2103	P30	B17	Post-cholecystectomy	Idiopathic BAD
0.8575514874141876
2104	P30	B20	Post-cholecystectomy	Idiopathic BAD
0.9471777269260107
2105	P30	B23	Post-cholecystectomy	Idiopathic BAD
0.9166666666666666
2106	P30	B31	Post-cholecystectomy	Idiopathic BAD
0.7942410373760488
2107	P30	B35	Post-cholecystectomy	Idiopathic BAD
0.9431731502669718
2108	P30	B39	Post-cholecystectomy	Idiopathic BAD
0.898741418764302
2109	P30	B43	Post-cholecystectomy	Idiopathic BAD
0.9563310450038138
2110	P30	B47	Post-cholecystectomy	Idiopathic BAD
0.8796720061022121
2111	P30	B48	Post-cholecystectomy	Idiopathic BAD
0.8249427917620137
2112	P30	B49	Post-cholecystectomy	Idiopathic BAD
0.9101830663615561
2113	P30	B53	Post-cholecystectomy	Idiopathic BAD
0.9061784897025171
2114	P30	B54	Post-cholecystectomy	Idiopathic BAD
0.9553775743707094
2115	P30	B55	Post-cholecystectomy	Idiopathic BAD

0.969488939740656
2116 P30 B59 Post-cholecystectomy Idiopathic BAD
0.9546147978642258
2117 P30 B70 Post-cholecystectomy Idiopathic BAD
0.9639588100686499
2118 P30 B74 Post-cholecystectomy Idiopathic BAD
0.9101830663615561
2119 P30 B77 Post-cholecystectomy Idiopathic BAD
0.8852021357742181
2120 P30 B81 Post-cholecystectomy Idiopathic BAD
0.885392829900839
2121 P30 B84 Post-cholecystectomy Idiopathic BAD
0.9344012204424104
2122 P30 B89 Post-cholecystectomy Idiopathic BAD
0.9729214340198322
2123 P30 B92 Post-cholecystectomy Idiopathic BAD
0.9290617848970252
2124 P30 B95 Post-cholecystectomy Idiopathic BAD
0.9250572082379863
2125 P30 B99 Post-cholecystectomy Idiopathic BAD
0.9220061022120518
2126 P30 B103 Post-cholecystectomy Idiopathic BAD
0.9729214340198322
2127 P30 B106 Post-cholecystectomy Idiopathic BAD
0.9546147978642258
2128 P30 B109 Post-cholecystectomy Idiopathic BAD
0.9427917620137299
2129 P30 B118 Post-cholecystectomy Idiopathic BAD
0.9664378337147216
2130 P30 B119 Post-cholecystectomy Idiopathic BAD
0.8800533943554538
2131 P33 B1 Post-cholecystectomy Idiopathic BAD
0.8861556064073226
2132 P33 B5 Post-cholecystectomy Idiopathic BAD
0.7730739893211289
2133 P33 B6 Post-cholecystectomy Idiopathic BAD
0.8178871090770404
2134 P33 B10 Post-cholecystectomy Idiopathic BAD
0.8914950419527079
2135 P33 B17 Post-cholecystectomy Idiopathic BAD
0.8276125095347063
2136 P33 B20 Post-cholecystectomy Idiopathic BAD
0.8278032036613272
2137 P33 B23 Post-cholecystectomy Idiopathic BAD
0.7774599542334096
2138 P33 B31 Post-cholecystectomy Idiopathic BAD
0.8579328756674295
2139 P33 B35 Post-cholecystectomy Idiopathic BAD
0.9794050343249427
2140 P33 B39 Post-cholecystectomy Idiopathic BAD
0.8518306636155606
2141 P33 B43 Post-cholecystectomy Idiopathic BAD
0.8476353928299009
2142 P33 B47 Post-cholecystectomy Idiopathic BAD

0.7820366132723112
2143	P33	B48	Post−cholecystectomy	Idiopathic BAD
0.8688024408848207
2144	P33	B49	Post−cholecystectomy	Idiopathic BAD
0.8581235697940504
2145	P33	B53	Post−cholecystectomy	Idiopathic BAD
0.8457284515636918
2146	P33	B54	Post−cholecystectomy	Idiopathic BAD
0.8577421815408085
2147	P33	B55	Post−cholecystectomy	Idiopathic BAD
0.9057971014492754
2148	P33	B59	Post−cholecystectomy	Idiopathic BAD
0.9610983981693364
2149	P33	B70	Post−cholecystectomy	Idiopathic BAD
0.6720061022120518
2150	P33	B74	Post−cholecystectomy	Idiopathic BAD
0.897025171624714
2151	P33	B77	Post−cholecystectomy	Idiopathic BAD
0.822463768115942
2152	P33	B81	Post−cholecystectomy	Idiopathic BAD
0.8565980167810832
2153	P33	B84	Post−cholecystectomy	Idiopathic BAD
0.7864225781845919
2154	P33	B89	Post−cholecystectomy	Idiopathic BAD
0.8419145690312738
2155	P33	B92	Post−cholecystectomy	Idiopathic BAD
0.8106407322654462
2156	P33	B95	Post−cholecystectomy	Idiopathic BAD
0.8941647597254004
2157	P33	B99	Post−cholecystectomy	Idiopathic BAD
0.7227307398932112
2158	P33	B103	Post−cholecystectomy	Idiopathic BAD
0.9002669717772692
2159	P33	B106	Post−cholecystectomy	Idiopathic BAD
0.9481311975591151
2160	P33	B109	Post−cholecystectomy	Idiopathic BAD
0.6521739130434783
2161	P33	B118	Post−cholecystectomy	Idiopathic BAD
0.8642257818459191
2162	P33	B119	Post−cholecystectomy	Idiopathic BAD
0.6678108314263921
2163	P35	B1	Post−cholecystectomy	Idiopathic BAD
0.9500381388253242
2164	P35	B5	Post−cholecystectomy	Idiopathic BAD
0.8623188405797102
2165	P35	B6	Post−cholecystectomy	Idiopathic BAD
0.8861556064073226
2166	P35	B10	Post−cholecystectomy	Idiopathic BAD
0.9420289855072463
2167	P35	B17	Post−cholecystectomy	Idiopathic BAD
0.8569794050343249
2168	P35	B20	Post−cholecystectomy	Idiopathic BAD
0.8255148741418764
2169	P35	B23	Post−cholecystectomy	Idiopathic BAD

0.6424485125858124
2170	P35	B31	Post-cholecystectomy	Idiopathic BAD
0.8440122044241037
2171	P35	B35	Post-cholecystectomy	Idiopathic BAD
0.9832189168573608
2172	P35	B39	Post-cholecystectomy	Idiopathic BAD
0.8651792524790236
2173	P35	B43	Post-cholecystectomy	Idiopathic BAD
0.8909229595728452
2174	P35	B47	Post-cholecystectomy	Idiopathic BAD
0.7568649885583524
2175	P35	B48	Post-cholecystectomy	Idiopathic BAD
0.9477498093058734
2176	P35	B49	Post-cholecystectomy	Idiopathic BAD
0.8468726163234173
2177	P35	B53	Post-cholecystectomy	Idiopathic BAD
0.7946224256292906
2178	P35	B54	Post-cholecystectomy	Idiopathic BAD
0.7921434019832189
2179	P35	B55	Post-cholecystectomy	Idiopathic BAD
0.8539282990083905
2180	P35	B59	Post-cholecystectomy	Idiopathic BAD
0.9881769641495042
2181	P35	B70	Post-cholecystectomy	Idiopathic BAD
0.7858504958047292
2182	P35	B74	Post-cholecystectomy	Idiopathic BAD
0.9117086193745233
2183	P35	B77	Post-cholecystectomy	Idiopathic BAD
0.7316933638443935
2184	P35	B81	Post-cholecystectomy	Idiopathic BAD
0.8974065598779558
2185	P35	B84	Post-cholecystectomy	Idiopathic BAD
0.7717391304347826
2186	P35	B89	Post-cholecystectomy	Idiopathic BAD
0.8422959572845157
2187	P35	B92	Post-cholecystectomy	Idiopathic BAD
0.7673531655225019
2188	P35	B95	Post-cholecystectomy	Idiopathic BAD
0.8140732265446224
2189	P35	B99	Post-cholecystectomy	Idiopathic BAD
0.7683066361556065
2190	P35	B103	Post-cholecystectomy	Idiopathic BAD
0.8230358504958047
2191	P35	B106	Post-cholecystectomy	Idiopathic BAD
0.956140350877193
2192	P35	B109	Post-cholecystectomy	Idiopathic BAD
0.7046147978642258
2193	P35	B118	Post-cholecystectomy	Idiopathic BAD
0.9012204424103738
2194	P35	B119	Post-cholecystectomy	Idiopathic BAD
0.717391304347826
2195	P38	B1	Post-cholecystectomy	Idiopathic BAD
0.9391685736079328
2196	P38	B5	Post-cholecystectomy	Idiopathic BAD

0.9397406559877955
2197 P38 B6 Post−cholecystectomy Idiopathic BAD
0.9570938215102975
2198 P38 B10 Post−cholecystectomy Idiopathic BAD
0.8680396643783371
2199 P38 B17 Post−cholecystectomy Idiopathic BAD
0.9281083142639207
2200 P38 B20 Post−cholecystectomy Idiopathic BAD
0.9715865751334859
2201 P38 B23 Post−cholecystectomy Idiopathic BAD
0.8525934401220442
2202 P38 B31 Post−cholecystectomy Idiopathic BAD
0.9033180778032036
2203 P38 B35 Post−cholecystectomy Idiopathic BAD
0.9029366895499619
2204 P38 B39 Post−cholecystectomy Idiopathic BAD
0.9361174675819984
2205 P38 B43 Post−cholecystectomy Idiopathic BAD
0.9658657513348589
2206 P38 B47 Post−cholecystectomy Idiopathic BAD
0.9534706331045004
2207 P38 B48 Post−cholecystectomy Idiopathic BAD
0.912090007627765
2208 P38 B49 Post−cholecystectomy Idiopathic BAD
0.9467963386727689
2209 P38 B53 Post−cholecystectomy Idiopathic BAD
0.9164759725400458
2210 P38 B54 Post−cholecystectomy Idiopathic BAD
0.8880625476735317
2211 P38 B55 Post−cholecystectomy Idiopathic BAD
0.9824561403508771
2212 P38 B59 Post−cholecystectomy Idiopathic BAD
0.9195270785659801
2213 P38 B70 Post−cholecystectomy Idiopathic BAD
0.9080854309687262
2214 P38 B74 Post−cholecystectomy Idiopathic BAD
0.9210526315789473
2215 P38 B77 Post−cholecystectomy Idiopathic BAD
0.9054157131960335
2216 P38 B81 Post−cholecystectomy Idiopathic BAD
0.9393592677345538
2217 P38 B84 Post−cholecystectomy Idiopathic BAD
0.9206712433257056
2218 P38 B89 Post−cholecystectomy Idiopathic BAD
0.9757818459191457
2219 P38 B92 Post−cholecystectomy Idiopathic BAD
0.9029366895499619
2220 P38 B95 Post−cholecystectomy Idiopathic BAD
0.9689168573607932
2221 P38 B99 Post−cholecystectomy Idiopathic BAD
0.9681540808543097
2222 P38 B103 Post−cholecystectomy Idiopathic BAD
0.9708237986270023
2223 P38 B106 Post−cholecystectomy Idiopathic BAD

0.9824561403508771
2224 P38 B109 Post-cholecystectomy Idiopathic BAD
0.9784515636918383
2225 P38 B118 Post-cholecystectomy Idiopathic BAD
0.9492753623188406
2226 P38 B119 Post-cholecystectomy Idiopathic BAD
0.9269641495041953
2227 P39 B1 Post-cholecystectomy Idiopathic BAD
0.8165522501906941
2228 P39 B5 Post-cholecystectomy Idiopathic BAD
0.7446605644546148
2229 P39 B6 Post-cholecystectomy Idiopathic BAD
0.8903508771929824
2230 P39 B10 Post-cholecystectomy Idiopathic BAD
0.9057971014492754
2231 P39 B17 Post-cholecystectomy Idiopathic BAD
0.8880625476735317
2232 P39 B20 Post-cholecystectomy Idiopathic BAD
0.9290617848970252
2233 P39 B23 Post-cholecystectomy Idiopathic BAD
0.8480167810831426
2234 P39 B31 Post-cholecystectomy Idiopathic BAD
0.818649885583524
2235 P39 B35 Post-cholecystectomy Idiopathic BAD
0.8724256292906178
2236 P39 B39 Post-cholecystectomy Idiopathic BAD
0.8136918382913806
2237 P39 B43 Post-cholecystectomy Idiopathic BAD
0.9475591151792525
2238 P39 B47 Post-cholecystectomy Idiopathic BAD
0.8861556064073226
2239 P39 B48 Post-cholecystectomy Idiopathic BAD
0.6882151029748284
2240 P39 B49 Post-cholecystectomy Idiopathic BAD
0.8811975591151793
2241 P39 B53 Post-cholecystectomy Idiopathic BAD
0.9221967963386728
2242 P39 B54 Post-cholecystectomy Idiopathic BAD
0.9466056445461479
2243 P39 B55 Post-cholecystectomy Idiopathic BAD
0.9668192219679634
2244 P39 B59 Post-cholecystectomy Idiopathic BAD
0.9164759725400458
2245 P39 B70 Post-cholecystectomy Idiopathic BAD
0.9151411136536994
2246 P39 B74 Post-cholecystectomy Idiopathic BAD
0.9214340198321892
2247 P39 B77 Post-cholecystectomy Idiopathic BAD
0.9200991609458429
2248 P39 B81 Post-cholecystectomy Idiopathic BAD
0.8405797101449275
2249 P39 B84 Post-cholecystectomy Idiopathic BAD
0.9185736079328757
2250 P39 B89 Post-cholecystectomy Idiopathic BAD

0.839626239511823
2251 P39 B92 Post−cholecystectomy Idiopathic BAD
0.8710907704042715
2252 P39 B95 Post−cholecystectomy Idiopathic BAD
0.8455377574370709
2253 P39 B99 Post−cholecystectomy Idiopathic BAD
0.8058733790999237
2254 P39 B103 Post−cholecystectomy Idiopathic BAD
0.8972158657513348
2255 P39 B106 Post−cholecystectomy Idiopathic BAD
0.933066361556064
2256 P39 B109 Post−cholecystectomy Idiopathic BAD
0.9202898550724637
2257 P39 B118 Post−cholecystectomy Idiopathic BAD
0.9416475972540046
2258 P39 B119 Post−cholecystectomy Idiopathic BAD
0.7400839054157132
2259 P42 B1 Post−cholecystectomy Idiopathic BAD
0.9229595728451564
2260 P42 B5 Post−cholecystectomy Idiopathic BAD
0.8956903127383676
2261 P42 B6 Post−cholecystectomy Idiopathic BAD
0.9614797864225781
2262 P42 B10 Post−cholecystectomy Idiopathic BAD
0.9708237986270023
2263 P42 B17 Post−cholecystectomy Idiopathic BAD
0.9183829138062548
2264 P42 B20 Post−cholecystectomy Idiopathic BAD
0.9439359267734554
2265 P42 B23 Post−cholecystectomy Idiopathic BAD
0.7299771167048055
2266 P42 B31 Post−cholecystectomy Idiopathic BAD
0.9508009153318078
2267 P42 B35 Post−cholecystectomy Idiopathic BAD
0.9311594202898551
2268 P42 B39 Post−cholecystectomy Idiopathic BAD
0.8432494279176201
2269 P42 B43 Post−cholecystectomy Idiopathic BAD
0.9683447749809306
2270 P42 B47 Post−cholecystectomy Idiopathic BAD
0.894927536231884
2271 P42 B48 Post−cholecystectomy Idiopathic BAD
0.8958810068649885
2272 P42 B49 Post−cholecystectomy Idiopathic BAD
0.8804347826086957
2273 P42 B53 Post−cholecystectomy Idiopathic BAD
0.7713577421815409
2274 P42 B54 Post−cholecystectomy Idiopathic BAD
0.9462242562929062
2275 P42 B55 Post−cholecystectomy Idiopathic BAD
0.7585812356979404
2276 P42 B59 Post−cholecystectomy Idiopathic BAD
0.9509916094584286
2277 P42 B70 Post−cholecystectomy Idiopathic BAD

0.6710526315789473
2278	P42	B74	Post-cholecystectomy	Idiopathic BAD
0.9387871853546911
2279	P42	B77	Post-cholecystectomy	Idiopathic BAD
0.847254004576659
2280	P42	B81	Post-cholecystectomy	Idiopathic BAD
0.9324942791762014
2281	P42	B84	Post-cholecystectomy	Idiopathic BAD
0.8806254767353165
2282	P42	B89	Post-cholecystectomy	Idiopathic BAD
0.9414569031273837
2283	P42	B92	Post-cholecystectomy	Idiopathic BAD
0.876048817696415
2284	P42	B95	Post-cholecystectomy	Idiopathic BAD
0.9731121281464531
2285	P42	B99	Post-cholecystectomy	Idiopathic BAD
0.8327612509534706
2286	P42	B103	Post-cholecystectomy	Idiopathic BAD
0.7932875667429443
2287	P42	B106	Post-cholecystectomy	Idiopathic BAD
0.9822654462242563
2288	P42	B109	Post-cholecystectomy	Idiopathic BAD
0.7972921434019832
2289	P42	B118	Post-cholecystectomy	Idiopathic BAD
0.9647215865751335
2290	P42	B119	Post-cholecystectomy	Idiopathic BAD
0.7698321891685737
2291	P43	B1	Post-cholecystectomy	Idiopathic BAD
0.8007246376811594
2292	P43	B5	Post-cholecystectomy	Idiopathic BAD
0.8150266971777269
2293	P43	B6	Post-cholecystectomy	Idiopathic BAD
0.7618230358504958
2294	P43	B10	Post-cholecystectomy	Idiopathic BAD
0.8733790999237223
2295	P43	B17	Post-cholecystectomy	Idiopathic BAD
0.8447749809305873
2296	P43	B20	Post-cholecystectomy	Idiopathic BAD
0.8495423340961098
2297	P43	B23	Post-cholecystectomy	Idiopathic BAD
0.8709000762776506
2298	P43	B31	Post-cholecystectomy	Idiopathic BAD
0.8009153318077803
2299	P43	B35	Post-cholecystectomy	Idiopathic BAD
0.8237986270022883
2300	P43	B39	Post-cholecystectomy	Idiopathic BAD
0.88558352402746
2301	P43	B43	Post-cholecystectomy	Idiopathic BAD
0.7395118230358505
2302	P43	B47	Post-cholecystectomy	Idiopathic BAD
0.8493516399694889
2303	P43	B48	Post-cholecystectomy	Idiopathic BAD
0.738367658276125
2304	P43	B49	Post-cholecystectomy	Idiopathic BAD

0.7059496567505721
2305 P43 B53 Post−cholecystectomy Idiopathic BAD
0.8991228070175439
2306 P43 B54 Post−cholecystectomy Idiopathic BAD
0.9204805491990846
2307 P43 B55 Post−cholecystectomy Idiopathic BAD
0.9595728451563692
2308 P43 B59 Post−cholecystectomy Idiopathic BAD
0.9877955758962624
2309 P43 B70 Post−cholecystectomy Idiopathic BAD
0.9477498093058734
2310 P43 B74 Post−cholecystectomy Idiopathic BAD
0.9124713958810069
2311 P43 B77 Post−cholecystectomy Idiopathic BAD
0.8934019832189168
2312 P43 B81 Post−cholecystectomy Idiopathic BAD
0.7858504958047292
2313 P43 B84 Post−cholecystectomy Idiopathic BAD
0.7858504958047292
2314 P43 B89 Post−cholecystectomy Idiopathic BAD
0.7738367658276125
2315 P43 B92 Post−cholecystectomy Idiopathic BAD
0.8714721586575134
2316 P43 B95 Post−cholecystectomy Idiopathic BAD
0.8012967200610221
2317 P43 B99 Post−cholecystectomy Idiopathic BAD
0.7208237986270023
2318 P43 B103 Post−cholecystectomy Idiopathic BAD
0.8323798627002288
2319 P43 B106 Post−cholecystectomy Idiopathic BAD
0.9445080091533181
2320 P43 B109 Post−cholecystectomy Idiopathic BAD
0.8325705568268498
2321 P43 B118 Post−cholecystectomy Idiopathic BAD
0.8874904652936689
2322 P43 B119 Post−cholecystectomy Idiopathic BAD
0.7261632341723875
2323 P46 B1 Post−cholecystectomy Idiopathic BAD
0.7969107551487414
2324 P46 B5 Post−cholecystectomy Idiopathic BAD
0.7377955758962624
2325 P46 B6 Post−cholecystectomy Idiopathic BAD
0.8663234172387491
2326 P46 B10 Post−cholecystectomy Idiopathic BAD
0.9229595728451564
2327 P46 B17 Post−cholecystectomy Idiopathic BAD
0.7343630816170862
2328 P46 B20 Post−cholecystectomy Idiopathic BAD
0.6426392067124332
2329 P46 B23 Post−cholecystectomy Idiopathic BAD
0.7934782608695652
2330 P46 B31 Post−cholecystectomy Idiopathic BAD
0.8808161708619374
2331 P46 B35 Post−cholecystectomy Idiopathic BAD

0.9151411136536994
2332	P46	B39	Post−cholecystectomy	Idiopathic BAD
0.8478260869565217
2333	P46	B43	Post−cholecystectomy	Idiopathic BAD
0.7149122807017544
2334	P46	B47	Post−cholecystectomy	Idiopathic BAD
0.8163615560640732
2335	P46	B48	Post−cholecystectomy	Idiopathic BAD
0.8190312738367659
2336	P46	B49	Post−cholecystectomy	Idiopathic BAD
0.8030129672006102
2337	P46	B53	Post−cholecystectomy	Idiopathic BAD
0.8463005339435545
2338	P46	B54	Post−cholecystectomy	Idiopathic BAD
0.7534324942791762
2339	P46	B55	Post−cholecystectomy	Idiopathic BAD
0.8058733790999237
2340	P46	B59	Post−cholecystectomy	Idiopathic BAD
0.9757818459191457
2341	P46	B70	Post−cholecystectomy	Idiopathic BAD
0.7965293668954996
2342	P46	B74	Post−cholecystectomy	Idiopathic BAD
0.9139969488939741
2343	P46	B77	Post−cholecystectomy	Idiopathic BAD
0.8665141113653699
2344	P46	B81	Post−cholecystectomy	Idiopathic BAD
0.8504958047292144
2345	P46	B84	Post−cholecystectomy	Idiopathic BAD
0.8731884057971014
2346	P46	B89	Post−cholecystectomy	Idiopathic BAD
0.7921434019832189
2347	P46	B92	Post−cholecystectomy	Idiopathic BAD
0.6193745232646835
2348	P46	B95	Post−cholecystectomy	Idiopathic BAD
0.6500762776506483
2349	P46	B99	Post−cholecystectomy	Idiopathic BAD
0.6662852784134249
2350	P46	B103	Post−cholecystectomy	Idiopathic BAD
0.8752860411899314
2351	P46	B106	Post−cholecystectomy	Idiopathic BAD
0.8279938977879482
2352	P46	B109	Post−cholecystectomy	Idiopathic BAD
0.6624713958810069
2353	P46	B118	Post−cholecystectomy	Idiopathic BAD
0.7032799389778794
2354	P46	B119	Post−cholecystectomy	Idiopathic BAD
0.7810831426392068
2355	P47	B1	Post−cholecystectomy	Idiopathic BAD
0.9511823035850496
2356	P47	B5	Post−cholecystectomy	Idiopathic BAD
0.938977879481312
2357	P47	B6	Post−cholecystectomy	Idiopathic BAD
0.9317315026697178
2358	P47	B10	Post−cholecystectomy	Idiopathic BAD

0.9229595728451564
2359	P47	B17	Post-cholecystectomy	Idiopathic BAD
0.6903127383676583
2360	P47	B20	Post-cholecystectomy	Idiopathic BAD
0.6111746758199847
2361	P47	B23	Post-cholecystectomy	Idiopathic BAD
0.912090007627765
2362	P47	B31	Post-cholecystectomy	Idiopathic BAD
0.881769641495042
2363	P47	B35	Post-cholecystectomy	Idiopathic BAD
0.9853165522501907
2364	P47	B39	Post-cholecystectomy	Idiopathic BAD
0.9132341723874905
2365	P47	B43	Post-cholecystectomy	Idiopathic BAD
0.7376048817696415
2366	P47	B47	Post-cholecystectomy	Idiopathic BAD
0.9212433257055682
2367	P47	B48	Post-cholecystectomy	Idiopathic BAD
0.9734935163996948
2368	P47	B49	Post-cholecystectomy	Idiopathic BAD
0.90255530129672
2369	P47	B53	Post-cholecystectomy	Idiopathic BAD
0.9218154080854309
2370	P47	B54	Post-cholecystectomy	Idiopathic BAD
0.6575133485888635
2371	P47	B55	Post-cholecystectomy	Idiopathic BAD
0.5661708619374524
2372	P47	B59	Post-cholecystectomy	Idiopathic BAD
0.9490846681922197
2373	P47	B70	Post-cholecystectomy	Idiopathic BAD
0.9672006102212052
2374	P47	B74	Post-cholecystectomy	Idiopathic BAD
0.9534706331045004
2375	P47	B77	Post-cholecystectomy	Idiopathic BAD
0.9328756674294432
2376	P47	B81	Post-cholecystectomy	Idiopathic BAD
0.918001525553013
2377	P47	B84	Post-cholecystectomy	Idiopathic BAD
0.908276125095347
2378	P47	B89	Post-cholecystectomy	Idiopathic BAD
0.9631960335621663
2379	P47	B92	Post-cholecystectomy	Idiopathic BAD
0.5770404271548436
2380	P47	B95	Post-cholecystectomy	Idiopathic BAD
0.6567505720823799
2381	P47	B99	Post-cholecystectomy	Idiopathic BAD
0.9088482074752098
2382	P47	B103	Post-cholecystectomy	Idiopathic BAD
0.9378337147215866
2383	P47	B106	Post-cholecystectomy	Idiopathic BAD
0.5276506483600305
2384	P47	B109	Post-cholecystectomy	Idiopathic BAD
0.9738749046529367
2385	P47	B118	Post-cholecystectomy	Idiopathic BAD

0.6571319603356217
2386	P47	B119	Post-cholecystectomy	Idiopathic BAD
0.9317315026697178
2387	P50	B1	Post-cholecystectomy	Idiopathic BAD
0.8560259344012204
2388	P50	B5	Post-cholecystectomy	Idiopathic BAD
0.7879481311975591
2389	P50	B6	Post-cholecystectomy	Idiopathic BAD
0.8647978642257819
2390	P50	B10	Post-cholecystectomy	Idiopathic BAD
0.8459191456903128
2391	P50	B17	Post-cholecystectomy	Idiopathic BAD
0.7467581998474447
2392	P50	B20	Post-cholecystectomy	Idiopathic BAD
0.7953852021357742
2393	P50	B23	Post-cholecystectomy	Idiopathic BAD
0.6844012204424104
2394	P50	B31	Post-cholecystectomy	Idiopathic BAD
0.7795575896262396
2395	P50	B35	Post-cholecystectomy	Idiopathic BAD
0.9702517162471396
2396	P50	B39	Post-cholecystectomy	Idiopathic BAD
0.8426773455377574
2397	P50	B43	Post-cholecystectomy	Idiopathic BAD
0.8297101449275363
2398	P50	B47	Post-cholecystectomy	Idiopathic BAD
0.6845919145690312
2399	P50	B48	Post-cholecystectomy	Idiopathic BAD
0.8939740655987796
2400	P50	B49	Post-cholecystectomy	Idiopathic BAD
0.8333333333333334
2401	P50	B53	Post-cholecystectomy	Idiopathic BAD
0.7681159420289855
2402	P50	B54	Post-cholecystectomy	Idiopathic BAD
0.8400076277650649
2403	P50	B55	Post-cholecystectomy	Idiopathic BAD
0.8503051106025934
2404	P50	B59	Post-cholecystectomy	Idiopathic BAD
0.9721586575133486
2405	P50	B70	Post-cholecystectomy	Idiopathic BAD
0.6826849733028223
2406	P50	B74	Post-cholecystectomy	Idiopathic BAD
0.8375286041189931
2407	P50	B77	Post-cholecystectomy	Idiopathic BAD
0.7076659038901602
2408	P50	B81	Post-cholecystectomy	Idiopathic BAD
0.8392448512585813
2409	P50	B84	Post-cholecystectomy	Idiopathic BAD
0.8056826849733029
2410	P50	B89	Post-cholecystectomy	Idiopathic BAD
0.8272311212814645
2411	P50	B92	Post-cholecystectomy	Idiopathic BAD
0.7273073989321129
2412	P50	B95	Post-cholecystectomy	Idiopathic BAD

0.8842486651411137
2413 P50 B99 Post-cholecystectomy Idiopathic BAD
0.7278794813119756
2414 P50 B103 Post-cholecystectomy Idiopathic BAD
0.9029366895499619
2415 P50 B106 Post-cholecystectomy Idiopathic BAD
0.9282990083905416
2416 P50 B109 Post-cholecystectomy Idiopathic BAD
0.6598016781083142
2417 P50 B118 Post-cholecystectomy Idiopathic BAD
0.791952707856598
2418 P50 B119 Post-cholecystectomy Idiopathic BAD
0.7667810831426392
2419 P55 B1 Post-cholecystectomy Idiopathic BAD
0.8125476735316552
2420 P55 B5 Post-cholecystectomy Idiopathic BAD
0.8350495804729214
2421 P55 B6 Post-cholecystectomy Idiopathic BAD
0.7890922959572845
2422 P55 B10 Post-cholecystectomy Idiopathic BAD
0.8012967200610221
2423 P55 B17 Post-cholecystectomy Idiopathic BAD
0.7364607170099161
2424 P55 B20 Post-cholecystectomy Idiopathic BAD
0.8098779557589626
2425 P55 B23 Post-cholecystectomy Idiopathic BAD
0.5938215102974829
2426 P55 B31 Post-cholecystectomy Idiopathic BAD
0.757627765064836
2427 P55 B35 Post-cholecystectomy Idiopathic BAD
0.9706331045003814
2428 P55 B39 Post-cholecystectomy Idiopathic BAD
0.8653699466056446
2429 P55 B43 Post-cholecystectomy Idiopathic BAD
0.8152173913043478
2430 P55 B47 Post-cholecystectomy Idiopathic BAD
0.7732646834477498
2431 P55 B48 Post-cholecystectomy Idiopathic BAD
0.84744469870328
2432 P55 B49 Post-cholecystectomy Idiopathic BAD
0.7991990846681922
2433 P55 B53 Post-cholecystectomy Idiopathic BAD
0.7459954233409611
2434 P55 B54 Post-cholecystectomy Idiopathic BAD
0.8800533943554538
2435 P55 B55 Post-cholecystectomy Idiopathic BAD
0.8663234172387491
2436 P55 B59 Post-cholecystectomy Idiopathic BAD
0.9746376811594203
2437 P55 B70 Post-cholecystectomy Idiopathic BAD
0.7883295194508009
2438 P55 B74 Post-cholecystectomy Idiopathic BAD
0.8049199084668193
2439 P55 B77 Post-cholecystectomy Idiopathic BAD

0.7553394355453852
2440 P55 B81 Post−cholecystectomy Idiopathic BAD
0.8531655225019069
2441 P55 B84 Post−cholecystectomy Idiopathic BAD
0.8209382151029748
2442 P55 B89 Post−cholecystectomy Idiopathic BAD
0.8194126620900076
2443 P55 B92 Post−cholecystectomy Idiopathic BAD
0.7444698703279939
2444 P55 B95 Post−cholecystectomy Idiopathic BAD
0.8792906178489702
2445 P55 B99 Post−cholecystectomy Idiopathic BAD
0.7090007627765065
2446 P55 B103 Post−cholecystectomy Idiopathic BAD
0.9256292906178489
2447 P55 B106 Post−cholecystectomy Idiopathic BAD
0.9254385964912281
2448 P55 B109 Post−cholecystectomy Idiopathic BAD
0.7522883295194508
2449 P55 B118 Post−cholecystectomy Idiopathic BAD
0.8634630053394355
2450 P55 B119 Post−cholecystectomy Idiopathic BAD
0.6662852784134249
2451 P58 B1 Post−cholecystectomy Idiopathic BAD
0.8745232646834478
2452 P58 B5 Post−cholecystectomy Idiopathic BAD
0.7742181540808543
2453 P58 B6 Post−cholecystectomy Idiopathic BAD
0.9099923722349351
2454 P58 B10 Post−cholecystectomy Idiopathic BAD
0.9138062547673532
2455 P58 B17 Post−cholecystectomy Idiopathic BAD
0.8558352402745996
2456 P58 B20 Post−cholecystectomy Idiopathic BAD
0.8453470633104501
2457 P58 B23 Post−cholecystectomy Idiopathic BAD
0.6897406559877955
2458 P58 B31 Post−cholecystectomy Idiopathic BAD
0.8775743707093822
2459 P58 B35 Post−cholecystectomy Idiopathic BAD
0.9935163996948894
2460 P58 B39 Post−cholecystectomy Idiopathic BAD
0.8558352402745996
2461 P58 B43 Post−cholecystectomy Idiopathic BAD
0.9315408085430968
2462 P58 B47 Post−cholecystectomy Idiopathic BAD
0.7421815408085431
2463 P58 B48 Post−cholecystectomy Idiopathic BAD
0.8668954996186118
2464 P58 B49 Post−cholecystectomy Idiopathic BAD
0.7221586575133486
2465 P58 B53 Post−cholecystectomy Idiopathic BAD
0.7116704805491991
2466 P58 B54 Post−cholecystectomy Idiopathic BAD

0.8409610983981693
2467 P58 B55 Post-cholecystectomy Idiopathic BAD
0.8764302059496567
2468 P58 B59 Post-cholecystectomy Idiopathic BAD
0.9921815408085431
2469 P58 B70 Post-cholecystectomy Idiopathic BAD
0.6735316552250191
2470 P58 B74 Post-cholecystectomy Idiopathic BAD
0.9004576659038902
2471 P58 B77 Post-cholecystectomy Idiopathic BAD
0.7917620137299771
2472 P58 B81 Post-cholecystectomy Idiopathic BAD
0.8827231121281465
2473 P58 B84 Post-cholecystectomy Idiopathic BAD
0.8371472158657514
2474 P58 B89 Post-cholecystectomy Idiopathic BAD
0.8766209000762777
2475 P58 B92 Post-cholecystectomy Idiopathic BAD
0.7734553775743707
2476 P58 B95 Post-cholecystectomy Idiopathic BAD
0.8308543096872616
2477 P58 B99 Post-cholecystectomy Idiopathic BAD
0.6542715484363082
2478 P58 B103 Post-cholecystectomy Idiopathic BAD
0.8935926773455377
2479 P58 B106 Post-cholecystectomy Idiopathic BAD
0.9593821510297483
2480 P58 B109 Post-cholecystectomy Idiopathic BAD
0.6912662090007627
2481 P58 B118 Post-cholecystectomy Idiopathic BAD
0.868421052631579
2482 P58 B119 Post-cholecystectomy Idiopathic BAD
0.7158657513348589
2483 P60 B1 Post-cholecystectomy Idiopathic BAD
0.7841342486651411
2484 P60 B5 Post-cholecystectomy Idiopathic BAD
0.8114035087719298
2485 P60 B6 Post-cholecystectomy Idiopathic BAD
0.9372616323417239
2486 P60 B10 Post-cholecystectomy Idiopathic BAD
0.8918764302059496
2487 P60 B17 Post-cholecystectomy Idiopathic BAD
0.8865369946605645
2488 P60 B20 Post-cholecystectomy Idiopathic BAD
0.9530892448512586
2489 P60 B23 Post-cholecystectomy Idiopathic BAD
0.916094584286804
2490 P60 B31 Post-cholecystectomy Idiopathic BAD
0.8729977116704806
2491 P60 B35 Post-cholecystectomy Idiopathic BAD
0.7837528604118993
2492 P60 B39 Post-cholecystectomy Idiopathic BAD
0.8865369946605645
2493 P60 B43 Post-cholecystectomy Idiopathic BAD

0.9773073989321129
2494	P60	B47	Post-cholecystectomy	Idiopathic BAD
0.9057971014492754
2495	P60	B48	Post-cholecystectomy	Idiopathic BAD
0.644927536231884
2496	P60	B49	Post-cholecystectomy	Idiopathic BAD
0.8247520976353928
2497	P60	B53	Post-cholecystectomy	Idiopathic BAD
0.873951182303585
2498	P60	B54	Post-cholecystectomy	Idiopathic BAD
0.9593821510297483
2499	P60	B55	Post-cholecystectomy	Idiopathic BAD
0.9849351639969489
2500	P60	B59	Post-cholecystectomy	Idiopathic BAD
0.9839816933638444
2501	P60	B70	Post-cholecystectomy	Idiopathic BAD
0.761441647597254
2502	P60	B74	Post-cholecystectomy	Idiopathic BAD
0.988367658276125
2503	P60	B77	Post-cholecystectomy	Idiopathic BAD
0.9609077040427155
2504	P60	B81	Post-cholecystectomy	Idiopathic BAD
0.9227688787185355
2505	P60	B84	Post-cholecystectomy	Idiopathic BAD
0.9458428680396643
2506	P60	B89	Post-cholecystectomy	Idiopathic BAD
0.9113272311212814
2507	P60	B92	Post-cholecystectomy	Idiopathic BAD
0.9170480549199085
2508	P60	B95	Post-cholecystectomy	Idiopathic BAD
0.8972158657513348
2509	P60	B99	Post-cholecystectomy	Idiopathic BAD
0.8295194508009154
2510	P60	B103	Post-cholecystectomy	Idiopathic BAD
0.9328756674294432
2511	P60	B106	Post-cholecystectomy	Idiopathic BAD
0.9811212814645309
2512	P60	B109	Post-cholecystectomy	Idiopathic BAD
0.7814645308924485
2513	P60	B118	Post-cholecystectomy	Idiopathic BAD
0.9672006102212052
2514	P60	B119	Post-cholecystectomy	Idiopathic BAD
0.8829138062547673
2515	P63	B1	Post-cholecystectomy	Idiopathic BAD
0.8993135011441648
2516	P63	B5	Post-cholecystectomy	Idiopathic BAD
0.8459191456903128
2517	P63	B6	Post-cholecystectomy	Idiopathic BAD
0.8489702517162472
2518	P63	B10	Post-cholecystectomy	Idiopathic BAD
0.8501144164759725
2519	P63	B17	Post-cholecystectomy	Idiopathic BAD
0.8274218154080855
2520	P63	B20	Post-cholecystectomy	Idiopathic BAD

0.8361937452326468
2521	P63	B23	Post-cholecystectomy	Idiopathic BAD
0.8081617086193745
2522	P63	B31	Post-cholecystectomy	Idiopathic BAD
0.8257055682684973
2523	P63	B35	Post-cholecystectomy	Idiopathic BAD
0.9977116704805492
2524	P63	B39	Post-cholecystectomy	Idiopathic BAD
0.8726163234172387
2525	P63	B43	Post-cholecystectomy	Idiopathic BAD
0.839626239511823
2526	P63	B47	Post-cholecystectomy	Idiopathic BAD
0.7923340961098398
2527	P63	B48	Post-cholecystectomy	Idiopathic BAD
0.9534706331045004
2528	P63	B49	Post-cholecystectomy	Idiopathic BAD
0.7650648360030511
2529	P63	B53	Post-cholecystectomy	Idiopathic BAD
0.881578947368421
2530	P63	B54	Post-cholecystectomy	Idiopathic BAD
0.9267734553775744
2531	P63	B55	Post-cholecystectomy	Idiopathic BAD
0.9288710907704043
2532	P63	B59	Post-cholecystectomy	Idiopathic BAD
0.9706331045003814
2533	P63	B70	Post-cholecystectomy	Idiopathic BAD
0.881769641495042
2534	P63	B74	Post-cholecystectomy	Idiopathic BAD
0.8321891685736079
2535	P63	B77	Post-cholecystectomy	Idiopathic BAD
0.8276125095347063
2536	P63	B81	Post-cholecystectomy	Idiopathic BAD
0.7837528604118993
2537	P63	B84	Post-cholecystectomy	Idiopathic BAD
0.8907322654462243
2538	P63	B89	Post-cholecystectomy	Idiopathic BAD
0.8314263920671243
2539	P63	B92	Post-cholecystectomy	Idiopathic BAD
0.7879481311975591
2540	P63	B95	Post-cholecystectomy	Idiopathic BAD
0.9153318077803204
2541	P63	B99	Post-cholecystectomy	Idiopathic BAD
0.658276125095347
2542	P63	B103	Post-cholecystectomy	Idiopathic BAD
0.8991228070175439
2543	P63	B106	Post-cholecystectomy	Idiopathic BAD
0.9374523264683448
2544	P63	B109	Post-cholecystectomy	Idiopathic BAD
0.8047292143401983
2545	P63	B118	Post-cholecystectomy	Idiopathic BAD
0.8613653699466056
2546	P63	B119	Post-cholecystectomy	Idiopathic BAD
0.7561022120518688
2547	P65	B1	Post-cholecystectomy	Idiopathic BAD

0.9235316552250191
2548	P65	B5	Post-cholecystectomy	Idiopathic BAD
0.8278032036613272
2549	P65	B6	Post-cholecystectomy	Idiopathic BAD
0.8556445461479787
2550	P65	B10	Post-cholecystectomy	Idiopathic BAD
0.9109458428680397
2551	P65	B17	Post-cholecystectomy	Idiopathic BAD
0.8247520976353928
2552	P65	B20	Post-cholecystectomy	Idiopathic BAD
0.8560259344012204
2553	P65	B23	Post-cholecystectomy	Idiopathic BAD
0.7524790236460717
2554	P65	B31	Post-cholecystectomy	Idiopathic BAD
0.933066361556064
2555	P65	B35	Post-cholecystectomy	Idiopathic BAD
0.9584286803966438
2556	P65	B39	Post-cholecystectomy	Idiopathic BAD
0.9101830663615561
2557	P65	B43	Post-cholecystectomy	Idiopathic BAD
0.8836765827612509
2558	P65	B47	Post-cholecystectomy	Idiopathic BAD
0.7841342486651411
2559	P65	B48	Post-cholecystectomy	Idiopathic BAD
0.9006483600305111
2560	P65	B49	Post-cholecystectomy	Idiopathic BAD
0.7622044241037376
2561	P65	B53	Post-cholecystectomy	Idiopathic BAD
0.8123569794050344
2562	P65	B54	Post-cholecystectomy	Idiopathic BAD
0.839626239511823
2563	P65	B55	Post-cholecystectomy	Idiopathic BAD
0.8478260869565217
2564	P65	B59	Post-cholecystectomy	Idiopathic BAD
0.9841723874904653
2565	P65	B70	Post-cholecystectomy	Idiopathic BAD
0.8171243325705568
2566	P65	B74	Post-cholecystectomy	Idiopathic BAD
0.877765064836003
2567	P65	B77	Post-cholecystectomy	Idiopathic BAD
0.7715484363081617
2568	P65	B81	Post-cholecystectomy	Idiopathic BAD
0.8390541571319603
2569	P65	B84	Post-cholecystectomy	Idiopathic BAD
0.8392448512585813
2570	P65	B89	Post-cholecystectomy	Idiopathic BAD
0.8459191456903128
2571	P65	B92	Post-cholecystectomy	Idiopathic BAD
0.8184591914569032
2572	P65	B95	Post-cholecystectomy	Idiopathic BAD
0.8790999237223494
2573	P65	B99	Post-cholecystectomy	Idiopathic BAD
0.7648741418764302
2574	P65	B103	Post-cholecystectomy	Idiopathic BAD

0.8905415713196033
2575 P65 B106 Post-cholecystectomy Idiopathic BAD
0.9437452326468345
2576 P65 B109 Post-cholecystectomy Idiopathic BAD
0.7244469870327994
2577 P65 B118 Post-cholecystectomy Idiopathic BAD
0.8928299008390541
2578 P65 B119 Post-cholecystectomy Idiopathic BAD
0.7231121281464531
2579 P68 B1 Post-cholecystectomy Idiopathic BAD
0.9469870327993898
2580 P68 B5 Post-cholecystectomy Idiopathic BAD
0.8087337909992373
2581 P68 B6 Post-cholecystectomy Idiopathic BAD
0.8636536994660564
2582 P68 B10 Post-cholecystectomy Idiopathic BAD
0.8983600305110603
2583 P68 B17 Post-cholecystectomy Idiopathic BAD
0.8453470633104501
2584 P68 B20 Post-cholecystectomy Idiopathic BAD
0.8596491228070176
2585 P68 B23 Post-cholecystectomy Idiopathic BAD
0.6966056445461479
2586 P68 B31 Post-cholecystectomy Idiopathic BAD
0.8781464530892449
2587 P68 B35 Post-cholecystectomy Idiopathic BAD
0.8217009916094584
2588 P68 B39 Post-cholecystectomy Idiopathic BAD
0.8342868039664378
2589 P68 B43 Post-cholecystectomy Idiopathic BAD
0.891113653699466
2590 P68 B47 Post-cholecystectomy Idiopathic BAD
0.7827993897787948
2591 P68 B48 Post-cholecystectomy Idiopathic BAD
0.793859649122807
2592 P68 B49 Post-cholecystectomy Idiopathic BAD
0.7589626239511823
2593 P68 B53 Post-cholecystectomy Idiopathic BAD
0.7896643783371472
2594 P68 B54 Post-cholecystectomy Idiopathic BAD
0.9176201372997712
2595 P68 B55 Post-cholecystectomy Idiopathic BAD
0.8539282990083905
2596 P68 B59 Post-cholecystectomy Idiopathic BAD
0.9759725400457666
2597 P68 B70 Post-cholecystectomy Idiopathic BAD
0.6632341723874905
2598 P68 B74 Post-cholecystectomy Idiopathic BAD
0.8659420289855072
2599 P68 B77 Post-cholecystectomy Idiopathic BAD
0.7200610221205187
2600 P68 B81 Post-cholecystectomy Idiopathic BAD
0.8863463005339436
2601 P68 B84 Post-cholecystectomy Idiopathic BAD

0.831998474446987
2602 P68 B89 Post–cholecystectomy Idiopathic BAD
0.931350114416476
2603 P68 B92 Post–cholecystectomy Idiopathic BAD
0.8508771929824561
2604 P68 B95 Post–cholecystectomy Idiopathic BAD
0.919717772692601
2605 P68 B99 Post–cholecystectomy Idiopathic BAD
0.7273073989321129
2606 P68 B103 Post–cholecystectomy Idiopathic BAD
0.8880625476735317
2607 P68 B106 Post–cholecystectomy Idiopathic BAD
0.9506102212051869
2608 P68 B109 Post–cholecystectomy Idiopathic BAD
0.7801296720061022
2609 P68 B118 Post–cholecystectomy Idiopathic BAD
0.9378337147215866
2610 P68 B119 Post–cholecystectomy Idiopathic BAD
0.6668573607932876
2611 P70 B1 Post–cholecystectomy Idiopathic BAD
0.9136155606407322
2612 P70 B5 Post–cholecystectomy Idiopathic BAD
0.7868039664378337
2613 P70 B6 Post–cholecystectomy Idiopathic BAD
0.8796720061022121
2614 P70 B10 Post–cholecystectomy Idiopathic BAD
0.9610983981693364
2615 P70 B17 Post–cholecystectomy Idiopathic BAD
0.8743325705568269
2616 P70 B20 Post–cholecystectomy Idiopathic BAD
0.8281845919145691
2617 P70 B23 Post–cholecystectomy Idiopathic BAD
0.7151029748283753
2618 P70 B31 Post–cholecystectomy Idiopathic BAD
0.8710907704042715
2619 P70 B35 Post–cholecystectomy Idiopathic BAD
0.8577421815408085
2620 P70 B39 Post–cholecystectomy Idiopathic BAD
0.8878718535469108
2621 P70 B43 Post–cholecystectomy Idiopathic BAD
0.8880625476735317
2622 P70 B47 Post–cholecystectomy Idiopathic BAD
0.820747520976354
2623 P70 B48 Post–cholecystectomy Idiopathic BAD
0.8859649122807017
2624 P70 B49 Post–cholecystectomy Idiopathic BAD
0.9239130434782609
2625 P70 B53 Post–cholecystectomy Idiopathic BAD
0.8018688024408849
2626 P70 B54 Post–cholecystectomy Idiopathic BAD
0.8996948893974066
2627 P70 B55 Post–cholecystectomy Idiopathic BAD
0.8518306636155606
2628 P70 B59 Post–cholecystectomy Idiopathic BAD

0.9631960335621663
2629	P70	B70	Post-cholecystectomy	Idiopathic BAD
0.7215865751334859
2630	P70	B74	Post-cholecystectomy	Idiopathic BAD
0.9012204424103738
2631	P70	B77	Post-cholecystectomy	Idiopathic BAD
0.7669717772692601
2632	P70	B81	Post-cholecystectomy	Idiopathic BAD
0.8726163234172387
2633	P70	B84	Post-cholecystectomy	Idiopathic BAD
0.8308543096872616
2634	P70	B89	Post-cholecystectomy	Idiopathic BAD
0.8348588863463006
2635	P70	B92	Post-cholecystectomy	Idiopathic BAD
0.7894736842105263
2636	P70	B95	Post-cholecystectomy	Idiopathic BAD
0.8564073226544623
2637	P70	B99	Post-cholecystectomy	Idiopathic BAD
0.8192219679633868
2638	P70	B103	Post-cholecystectomy	Idiopathic BAD
0.9021739130434783
2639	P70	B106	Post-cholecystectomy	Idiopathic BAD
0.937070938215103
2640	P70	B109	Post-cholecystectomy	Idiopathic BAD
0.7438977879481312
2641	P70	B118	Post-cholecystectomy	Idiopathic BAD
0.8552631578947368
2642	P70	B119	Post-cholecystectomy	Idiopathic BAD
0.6842105263157895
2643	P71	B1	Post-cholecystectomy	Idiopathic BAD
0.9435545385202135
2644	P71	B5	Post-cholecystectomy	Idiopathic BAD
0.8196033562166285
2645	P71	B6	Post-cholecystectomy	Idiopathic BAD
0.9080854309687262
2646	P71	B10	Post-cholecystectomy	Idiopathic BAD
0.9612890922959573
2647	P71	B17	Post-cholecystectomy	Idiopathic BAD
0.6512204424103738
2648	P71	B20	Post-cholecystectomy	Idiopathic BAD
0.6577040427154843
2649	P71	B23	Post-cholecystectomy	Idiopathic BAD
0.8272311212814645
2650	P71	B31	Post-cholecystectomy	Idiopathic BAD
0.8745232646834478
2651	P71	B35	Post-cholecystectomy	Idiopathic BAD
0.9670099160945843
2652	P71	B39	Post-cholecystectomy	Idiopathic BAD
0.9048436308161708
2653	P71	B43	Post-cholecystectomy	Idiopathic BAD
0.7534324942791762
2654	P71	B47	Post-cholecystectomy	Idiopathic BAD
0.8373379099923722
2655	P71	B48	Post-cholecystectomy	Idiopathic BAD

0.9527078565980168
2656	P71	B49	Post-cholecystectomy	Idiopathic BAD
0.9094202898550725
2657	P71	B53	Post-cholecystectomy	Idiopathic BAD
0.864607170099161
2658	P71	B54	Post-cholecystectomy	Idiopathic BAD
0.7170099160945843
2659	P71	B55	Post-cholecystectomy	Idiopathic BAD
0.7768878718535469
2660	P71	B59	Post-cholecystectomy	Idiopathic BAD
0.9891304347826086
2661	P71	B70	Post-cholecystectomy	Idiopathic BAD
0.8960717009916095
2662	P71	B74	Post-cholecystectomy	Idiopathic BAD
0.9422196796338673
2663	P71	B77	Post-cholecystectomy	Idiopathic BAD
0.7948131197559115
2664	P71	B81	Post-cholecystectomy	Idiopathic BAD
0.8749046529366895
2665	P71	B84	Post-cholecystectomy	Idiopathic BAD
0.8323798627002288
2666	P71	B89	Post-cholecystectomy	Idiopathic BAD
0.8695652173913043
2667	P71	B92	Post-cholecystectomy	Idiopathic BAD
0.7299771167048055
2668	P71	B95	Post-cholecystectomy	Idiopathic BAD
0.8026315789473685
2669	P71	B99	Post-cholecystectomy	Idiopathic BAD
0.8419145690312738
2670	P71	B103	Post-cholecystectomy	Idiopathic BAD
0.7463768115942029
2671	P71	B106	Post-cholecystectomy	Idiopathic BAD
0.7520976353928299
2672	P71	B109	Post-cholecystectomy	Idiopathic BAD
0.8676582761250954
2673	P71	B118	Post-cholecystectomy	Idiopathic BAD
0.7265446224256293
2674	P71	B119	Post-cholecystectomy	Idiopathic BAD
0.8445842868039665
2675	P74	B1	Post-cholecystectomy	Idiopathic BAD
0.8905415713196033
2676	P74	B5	Post-cholecystectomy	Idiopathic BAD
0.7564836003051106
2677	P74	B6	Post-cholecystectomy	Idiopathic BAD
0.8117848970251716
2678	P74	B10	Post-cholecystectomy	Idiopathic BAD
0.8890160183066361
2679	P74	B17	Post-cholecystectomy	Idiopathic BAD
0.8176964149504196
2680	P74	B20	Post-cholecystectomy	Idiopathic BAD
0.8503051106025934
2681	P74	B23	Post-cholecystectomy	Idiopathic BAD
0.7658276125095347
2682	P74	B31	Post-cholecystectomy	Idiopathic BAD

0.8350495804729214
2683	P74	B35	Post-cholecystectomy	Idiopathic BAD
0.971205186880244
2684	P74	B39	Post-cholecystectomy	Idiopathic BAD
0.8785278413424866
2685	P74	B43	Post-cholecystectomy	Idiopathic BAD
0.8150266971777269
2686	P74	B47	Post-cholecystectomy	Idiopathic BAD
0.7572463768115942
2687	P74	B48	Post-cholecystectomy	Idiopathic BAD
0.9477498093058734
2688	P74	B49	Post-cholecystectomy	Idiopathic BAD
0.8384820747520977
2689	P74	B53	Post-cholecystectomy	Idiopathic BAD
0.7923340961098398
2690	P74	B54	Post-cholecystectomy	Idiopathic BAD
0.8813882532418001
2691	P74	B55	Post-cholecystectomy	Idiopathic BAD
0.9214340198321892
2692	P74	B59	Post-cholecystectomy	Idiopathic BAD
0.9628146453089245
2693	P74	B70	Post-cholecystectomy	Idiopathic BAD
0.7934782608695652
2694	P74	B74	Post-cholecystectomy	Idiopathic BAD
0.834096109839817
2695	P74	B77	Post-cholecystectomy	Idiopathic BAD
0.8285659801678108
2696	P74	B81	Post-cholecystectomy	Idiopathic BAD
0.8281845919145691
2697	P74	B84	Post-cholecystectomy	Idiopathic BAD
0.5636918382913806
2698	P74	B89	Post-cholecystectomy	Idiopathic BAD
0.8304729214340199
2699	P74	B92	Post-cholecystectomy	Idiopathic BAD
0.7192982456140351
2700	P74	B95	Post-cholecystectomy	Idiopathic BAD
0.8913043478260869
2701	P74	B99	Post-cholecystectomy	Idiopathic BAD
0.6948893974065599
2702	P74	B103	Post-cholecystectomy	Idiopathic BAD
0.8154080854309688
2703	P74	B106	Post-cholecystectomy	Idiopathic BAD
0.9549961861174676
2704	P74	B109	Post-cholecystectomy	Idiopathic BAD
0.7534324942791762
2705	P74	B118	Post-cholecystectomy	Idiopathic BAD
0.8728070175438597
2706	P74	B119	Post-cholecystectomy	Idiopathic BAD
0.7534324942791762
2707	P75	B1	Post-cholecystectomy	Idiopathic BAD
0.8554538520213577
2708	P75	B5	Post-cholecystectomy	Idiopathic BAD
0.738558352402746
2709	P75	B6	Post-cholecystectomy	Idiopathic BAD

0.8251334858886347
2710 P75 B10 Post−cholecystectomy Idiopathic BAD
0.831998474446987
2711 P75 B17 Post−cholecystectomy Idiopathic BAD
0.7467581998474447
2712 P75 B20 Post−cholecystectomy Idiopathic BAD
0.7906178489702517
2713 P75 B23 Post−cholecystectomy Idiopathic BAD
0.7479023646071701
2714 P75 B31 Post−cholecystectomy Idiopathic BAD
0.7873760488176964
2715 P75 B35 Post−cholecystectomy Idiopathic BAD
0.9521357742181541
2716 P75 B39 Post−cholecystectomy Idiopathic BAD
0.8222730739893211
2717 P75 B43 Post−cholecystectomy Idiopathic BAD
0.7364607170099161
2718 P75 B47 Post−cholecystectomy Idiopathic BAD
0.7051868802440885
2719 P75 B48 Post−cholecystectomy Idiopathic BAD
0.843440122044241
2720 P75 B49 Post−cholecystectomy Idiopathic BAD
0.8077803203661327
2721 P75 B53 Post−cholecystectomy Idiopathic BAD
0.8545003813882532
2722 P75 B54 Post−cholecystectomy Idiopathic BAD
0.8876811594202898
2723 P75 B55 Post−cholecystectomy Idiopathic BAD
0.9448893974065599
2724 P75 B59 Post−cholecystectomy Idiopathic BAD
0.9876048817696415
2725 P75 B70 Post−cholecystectomy Idiopathic BAD
0.8407704042715485
2726 P75 B74 Post−cholecystectomy Idiopathic BAD
0.8121662852784134
2727 P75 B77 Post−cholecystectomy Idiopathic BAD
0.729023646071701
2728 P75 B81 Post−cholecystectomy Idiopathic BAD
0.8096872616323417
2729 P75 B84 Post−cholecystectomy Idiopathic BAD
0.8628909229595728
2730 P75 B89 Post−cholecystectomy Idiopathic BAD
0.8146453089244852
2731 P75 B92 Post−cholecystectomy Idiopathic BAD
0.7734553775743707
2732 P75 B95 Post−cholecystectomy Idiopathic BAD
0.8350495804729214
2733 P75 B99 Post−cholecystectomy Idiopathic BAD
0.6643783371472158
2734 P75 B103 Post−cholecystectomy Idiopathic BAD
0.910373760488177
2735 P75 B106 Post−cholecystectomy Idiopathic BAD
0.9281083142639207
2736 P75 B109 Post−cholecystectomy Idiopathic BAD

0.8293287566742944
2737 P75 B118 Post-cholecystectomy Idiopathic BAD
0.8144546147978642
2738 P75 B119 Post-cholecystectomy Idiopathic BAD
0.7755530129672006
2739 P1 C1 Post-cholecystectomy Post-op CD
0.8636536994660564
2740 P1 C3 Post-cholecystectomy Post-op CD
0.784324942791762
2741 P1 C7 Post-cholecystectomy Post-op CD
0.9837909992372235
2742 P1 C8 Post-cholecystectomy Post-op CD
0.9279176201372997
2743 P1 C11 Post-cholecystectomy Post-op CD
0.9525171624713958
2744 P1 C15 Post-cholecystectomy Post-op CD
0.9677726926010679
2745 P1 C19 Post-cholecystectomy Post-op CD
0.9729214340198322
2746 P1 C22 Post-cholecystectomy Post-op CD
0.7751716247139588
2747 P1 C26 Post-cholecystectomy Post-op CD
0.9755911517925248
2748 P1 C28 Post-cholecystectomy Post-op CD
0.9645308924485125
2749 P1 C31 Post-cholecystectomy Post-op CD
0.9641495041952708
2750 P1 C35 Post-cholecystectomy Post-op CD
0.9536613272311213
2751 P1 C38 Post-cholecystectomy Post-op CD
0.9721586575133486
2752 P1 C40 Post-cholecystectomy Post-op CD
0.9605263157894737
2753 P1 C44 Post-cholecystectomy Post-op CD
0.9778794813119756
2754 P1 C47 Post-cholecystectomy Post-op CD
0.9816933638443935
2755 P1 C48 Post-cholecystectomy Post-op CD
0.8899694889397407
2756 P1 C49 Post-cholecystectomy Post-op CD
0.8920671243325705
2757 P1 C53 Post-cholecystectomy Post-op CD
0.9265827612509535
2758 P1 C56 Post-cholecystectomy Post-op CD
0.8977879481311976
2759 P1 C60 Post-cholecystectomy Post-op CD
0.8293287566742944
2760 P1 C62 Post-cholecystectomy Post-op CD
0.9303966437833715
2761 P1 C64 Post-cholecystectomy Post-op CD
0.902745995423341
2762 P1 C65 Post-cholecystectomy Post-op CD
0.8024408848207475
2763 P1 C69 Post-cholecystectomy Post-op CD

0.9521357742181541
2764	P1	C70	Post-cholecystectomy	Post-op CD
0.8895881006864989
2765	P1	C74	Post-cholecystectomy	Post-op CD
0.8270404271548436
2766	P1	C78	Post-cholecystectomy	Post-op CD
0.9448893974065599
2767	P2	C1	Post-cholecystectomy	Post-op CD
0.9410755148741419
2768	P2	C3	Post-cholecystectomy	Post-op CD
0.8808161708619374
2769	P2	C7	Post-cholecystectomy	Post-op CD
0.9157131960335622
2770	P2	C8	Post-cholecystectomy	Post-op CD
0.9210526315789473
2771	P2	C11	Post-cholecystectomy	Post-op CD
0.8977879481311976
2772	P2	C15	Post-cholecystectomy	Post-op CD
0.8424866514111365
2773	P2	C19	Post-cholecystectomy	Post-op CD
0.8560259344012204
2774	P2	C22	Post-cholecystectomy	Post-op CD
0.8790999237223494
2775	P2	C26	Post-cholecystectomy	Post-op CD
0.9353546910755148
2776	P2	C28	Post-cholecystectomy	Post-op CD
0.8756674294431731
2777	P2	C31	Post-cholecystectomy	Post-op CD
0.9679633867276888
2778	P2	C35	Post-cholecystectomy	Post-op CD
0.8735697940503433
2779	P2	C38	Post-cholecystectomy	Post-op CD
0.9654843630816171
2780	P2	C40	Post-cholecystectomy	Post-op CD
0.9569031273836766
2781	P2	C44	Post-cholecystectomy	Post-op CD
0.8813882532418001
2782	P2	C47	Post-cholecystectomy	Post-op CD
0.8924485125858124
2783	P2	C48	Post-cholecystectomy	Post-op CD
0.7858504958047292
2784	P2	C49	Post-cholecystectomy	Post-op CD
0.852974828375286
2785	P2	C53	Post-cholecystectomy	Post-op CD
0.801487414187643
2786	P2	C56	Post-cholecystectomy	Post-op CD
0.7406559877955758
2787	P2	C60	Post-cholecystectomy	Post-op CD
0.7713577421815409
2788	P2	C62	Post-cholecystectomy	Post-op CD
0.8485888634630053
2789	P2	C64	Post-cholecystectomy	Post-op CD
0.7990083905415714
2790	P2	C65	Post-cholecystectomy	Post-op CD

0.8150266971777269
2791	P2	C69	Post-cholecystectomy	Post-op CD
0.8215102974828375
2792	P2	C70	Post-cholecystectomy	Post-op CD
0.8400076277650649
2793	P2	C74	Post-cholecystectomy	Post-op CD
0.8424866514111365
2794	P2	C78	Post-cholecystectomy	Post-op CD
0.8291380625476735
2795	P4	C1	Post-cholecystectomy	Post-op CD
0.9475591151792525
2796	P4	C3	Post-cholecystectomy	Post-op CD
0.937070938215103
2797	P4	C7	Post-cholecystectomy	Post-op CD
0.9252479023646072
2798	P4	C8	Post-cholecystectomy	Post-op CD
0.9248665141113653
2799	P4	C11	Post-cholecystectomy	Post-op CD
0.9227688787185355
2800	P4	C15	Post-cholecystectomy	Post-op CD
0.8996948893974066
2801	P4	C19	Post-cholecystectomy	Post-op CD
0.8623188405797102
2802	P4	C22	Post-cholecystectomy	Post-op CD
0.9467963386727689
2803	P4	C26	Post-cholecystectomy	Post-op CD
0.8142639206712433
2804	P4	C28	Post-cholecystectomy	Post-op CD
0.9061784897025171
2805	P4	C31	Post-cholecystectomy	Post-op CD
0.9344012204424104
2806	P4	C35	Post-cholecystectomy	Post-op CD
0.9149504195270786
2807	P4	C38	Post-cholecystectomy	Post-op CD
0.9401220442410374
2808	P4	C40	Post-cholecystectomy	Post-op CD
0.7831807780320366
2809	P4	C44	Post-cholecystectomy	Post-op CD
0.8993135011441648
2810	P4	C47	Post-cholecystectomy	Post-op CD
0.8714721586575134
2811	P4	C48	Post-cholecystectomy	Post-op CD
0.9046529366895499
2812	P4	C49	Post-cholecystectomy	Post-op CD
0.8062547673531655
2813	P4	C53	Post-cholecystectomy	Post-op CD
0.9338291380625476
2814	P4	C56	Post-cholecystectomy	Post-op CD
0.8993135011441648
2815	P4	C60	Post-cholecystectomy	Post-op CD
0.8892067124332571
2816	P4	C62	Post-cholecystectomy	Post-op CD
0.9181922196796338
2817	P4	C64	Post-cholecystectomy	Post-op CD

0.9090389016018307
2818	P4	C65	Post-cholecystectomy	Post-op CD
0.9538520213577422
2819	P4	C69	Post-cholecystectomy	Post-op CD
0.7362700228832952
2820	P4	C70	Post-cholecystectomy	Post-op CD
0.9412662090007627
2821	P4	C74	Post-cholecystectomy	Post-op CD
0.9496567505720824
2822	P4	C78	Post-cholecystectomy	Post-op CD
0.7486651411136537
2823	P5	C1	Post-cholecystectomy	Post-op CD
0.9450800915331807
2824	P5	C3	Post-cholecystectomy	Post-op CD
0.9174294431731502
2825	P5	C7	Post-cholecystectomy	Post-op CD
0.8583142639206712
2826	P5	C8	Post-cholecystectomy	Post-op CD
0.9279176201372997
2827	P5	C11	Post-cholecystectomy	Post-op CD
0.8548817696414951
2828	P5	C15	Post-cholecystectomy	Post-op CD
0.8333333333333334
2829	P5	C19	Post-cholecystectomy	Post-op CD
0.858886346300534
2830	P5	C22	Post-cholecystectomy	Post-op CD
0.9420289855072463
2831	P5	C26	Post-cholecystectomy	Post-op CD
0.916094584286804
2832	P5	C28	Post-cholecystectomy	Post-op CD
0.8100686498855835
2833	P5	C31	Post-cholecystectomy	Post-op CD
0.9399313501144165
2834	P5	C35	Post-cholecystectomy	Post-op CD
0.8442028985507246
2835	P5	C38	Post-cholecystectomy	Post-op CD
0.9761632341723875
2836	P5	C40	Post-cholecystectomy	Post-op CD
0.8796720061022121
2837	P5	C44	Post-cholecystectomy	Post-op CD
0.8712814645308925
2838	P5	C47	Post-cholecystectomy	Post-op CD
0.8726163234172387
2839	P5	C48	Post-cholecystectomy	Post-op CD
0.8260869565217391
2840	P5	C49	Post-cholecystectomy	Post-op CD
0.8789092295957285
2841	P5	C53	Post-cholecystectomy	Post-op CD
0.8205568268497331
2842	P5	C56	Post-cholecystectomy	Post-op CD
0.7048054919908466
2843	P5	C60	Post-cholecystectomy	Post-op CD
0.7951945080091534
2844	P5	C62	Post-cholecystectomy	Post-op CD

0.8480167810831426
2845 P5 C64 Post-cholecystectomy Post-op CD
0.8598398169336384
2846 P5 C65 Post-cholecystectomy Post-op CD
0.8613653699466056
2847 P5 C69 Post-cholecystectomy Post-op CD
0.8188405797101449
2848 P5 C70 Post-cholecystectomy Post-op CD
0.8731884057971014
2849 P5 C74 Post-cholecystectomy Post-op CD
0.8964530892448512
2850 P5 C78 Post-cholecystectomy Post-op CD
0.8051106025934401
2851 P9 C1 Post-cholecystectomy Post-op CD
0.9570938215102975
2852 P9 C3 Post-cholecystectomy Post-op CD
0.9525171624713958
2853 P9 C7 Post-cholecystectomy Post-op CD
0.9054157131960335
2854 P9 C8 Post-cholecystectomy Post-op CD
0.9595728451563692
2855 P9 C11 Post-cholecystectomy Post-op CD
0.7414187643020596
2856 P9 C15 Post-cholecystectomy Post-op CD
0.7238749046529367
2857 P9 C19 Post-cholecystectomy Post-op CD
0.7364607170099161
2858 P9 C22 Post-cholecystectomy Post-op CD
0.9527078565980168
2859 P9 C26 Post-cholecystectomy Post-op CD
0.898741418764302
2860 P9 C28 Post-cholecystectomy Post-op CD
0.7105263157894737
2861 P9 C31 Post-cholecystectomy Post-op CD
0.8810068649885584
2862 P9 C35 Post-cholecystectomy Post-op CD
0.7398932112890922
2863 P9 C38 Post-cholecystectomy Post-op CD
0.9692982456140351
2864 P9 C40 Post-cholecystectomy Post-op CD
0.872234935163997
2865 P9 C44 Post-cholecystectomy Post-op CD
0.7793668954996186
2866 P9 C47 Post-cholecystectomy Post-op CD
0.8070175438596491
2867 P9 C48 Post-cholecystectomy Post-op CD
0.8867276887871853
2868 P9 C49 Post-cholecystectomy Post-op CD
0.9229595728451564
2869 P9 C53 Post-cholecystectomy Post-op CD
0.7898550724637681
2870 P9 C56 Post-cholecystectomy Post-op CD
0.7234935163996948
2871 P9 C60 Post-cholecystectomy Post-op CD

0.7702135774218154
2872   P9   C62   Post-cholecystectomy   Post-op CD
0.8981693363844394
2873   P9   C64   Post-cholecystectomy   Post-op CD
0.9016018306636155
2874   P9   C65   Post-cholecystectomy   Post-op CD
0.9517543859649122
2875   P9   C69   Post-cholecystectomy   Post-op CD
0.8714721586575134
2876   P9   C70   Post-cholecystectomy   Post-op CD
0.9340198321891686
2877   P9   C74   Post-cholecystectomy   Post-op CD
0.950419527078566
2878   P9   C78   Post-cholecystectomy   Post-op CD
0.7801296720061022
2879   P13   C1   Post-cholecystectomy   Post-op CD
0.9572845156369184
2880   P13   C3   Post-cholecystectomy   Post-op CD
0.9357360793287567
2881   P13   C7   Post-cholecystectomy   Post-op CD
0.9155225019069413
2882   P13   C8   Post-cholecystectomy   Post-op CD
0.969488939740656
2883   P13   C11   Post-cholecystectomy   Post-op CD
0.9277269260106789
2884   P13   C15   Post-cholecystectomy   Post-op CD
0.9077040427154843
2885   P13   C19   Post-cholecystectomy   Post-op CD
0.9189549961861174
2886   P13   C22   Post-cholecystectomy   Post-op CD
0.9603356216628528
2887   P13   C26   Post-cholecystectomy   Post-op CD
0.9296338672768879
2888   P13   C28   Post-cholecystectomy   Post-op CD
0.8768115942028986
2889   P13   C31   Post-cholecystectomy   Post-op CD
0.9723493516399695
2890   P13   C35   Post-cholecystectomy   Post-op CD
0.8575514874141876
2891   P13   C38   Post-cholecystectomy   Post-op CD
0.9788329519450801
2892   P13   C40   Post-cholecystectomy   Post-op CD
0.9101830663615561
2893   P13   C44   Post-cholecystectomy   Post-op CD
0.9563310450038138
2894   P13   C47   Post-cholecystectomy   Post-op CD
0.967391304347826
2895   P13   C48   Post-cholecystectomy   Post-op CD
0.8693745232646835
2896   P13   C49   Post-cholecystectomy   Post-op CD
0.8890160183066361
2897   P13   C53   Post-cholecystectomy   Post-op CD
0.8996948893974066
2898   P13   C56   Post-cholecystectomy   Post-op CD

0.8333333333333334
2899 P13 C60 Post-cholecystectomy Post-op CD
0.897025171624714
2900 P13 C62 Post-cholecystectomy Post-op CD
0.9016018306636155
2901 P13 C64 Post-cholecystectomy Post-op CD
0.8886346300533944
2902 P13 C65 Post-cholecystectomy Post-op CD
0.8956903127383676
2903 P13 C69 Post-cholecystectomy Post-op CD
0.86441647597254
2904 P13 C70 Post-cholecystectomy Post-op CD
0.8943554538520213
2905 P13 C74 Post-cholecystectomy Post-op CD
0.9099923722349351
2906 P13 C78 Post-cholecystectomy Post-op CD
0.8647978642257819
2907 P15 C1 Post-cholecystectomy Post-op CD
0.9244851258581236
2908 P15 C3 Post-cholecystectomy Post-op CD
0.851258581235698
2909 P15 C7 Post-cholecystectomy Post-op CD
0.8304729214340199
2910 P15 C8 Post-cholecystectomy Post-op CD
0.876048817696415
2911 P15 C11 Post-cholecystectomy Post-op CD
0.9473684210526315
2912 P15 C15 Post-cholecystectomy Post-op CD
0.9359267734553776
2913 P15 C19 Post-cholecystectomy Post-op CD
0.8157894736842105
2914 P15 C22 Post-cholecystectomy Post-op CD
0.9054157131960335
2915 P15 C26 Post-cholecystectomy Post-op CD
0.8766209000762777
2916 P15 C28 Post-cholecystectomy Post-op CD
0.9422196796338673
2917 P15 C31 Post-cholecystectomy Post-op CD
0.965675057208238
2918 P15 C35 Post-cholecystectomy Post-op CD
0.8832951945080092
2919 P15 C38 Post-cholecystectomy Post-op CD
0.9399313501144165
2920 P15 C40 Post-cholecystectomy Post-op CD
0.877765064836003
2921 P15 C44 Post-cholecystectomy Post-op CD
0.959954233409611
2922 P15 C47 Post-cholecystectomy Post-op CD
0.9595728451563692
2923 P15 C48 Post-cholecystectomy Post-op CD
0.7726926010678871
2924 P15 C49 Post-cholecystectomy Post-op CD
0.7818459191456903
2925 P15 C53 Post-cholecystectomy Post-op CD

0.8276125095347063
2926 P15 C56 Post-cholecystectomy Post-op CD
0.7885202135774219
2927 P15 C60 Post-cholecystectomy Post-op CD
0.7515255530129672
2928 P15 C62 Post-cholecystectomy Post-op CD
0.8159801678108314
2929 P15 C64 Post-cholecystectomy Post-op CD
0.7940503432494279
2930 P15 C65 Post-cholecystectomy Post-op CD
0.8125476735316552
2931 P15 C69 Post-cholecystectomy Post-op CD
0.734744469870328
2932 P15 C70 Post-cholecystectomy Post-op CD
0.8060640732265446
2933 P15 C74 Post-cholecystectomy Post-op CD
0.8291380625476735
2934 P15 C78 Post-cholecystectomy Post-op CD
0.8323798627002288
2935 P16 C1 Post-cholecystectomy Post-op CD
0.9117086193745233
2936 P16 C3 Post-cholecystectomy Post-op CD
0.9277269260106789
2937 P16 C7 Post-cholecystectomy Post-op CD
0.9090389016018307
2938 P16 C8 Post-cholecystectomy Post-op CD
0.9246758199847445
2939 P16 C11 Post-cholecystectomy Post-op CD
0.8762395118230358
2940 P16 C15 Post-cholecystectomy Post-op CD
0.8453470633104501
2941 P16 C19 Post-cholecystectomy Post-op CD
0.8914950419527079
2942 P16 C22 Post-cholecystectomy Post-op CD
0.914187643020595
2943 P16 C26 Post-cholecystectomy Post-op CD
0.8426773455377574
2944 P16 C28 Post-cholecystectomy Post-op CD
0.8613653699466056
2945 P16 C31 Post-cholecystectomy Post-op CD
0.8852021357742181
2946 P16 C35 Post-cholecystectomy Post-op CD
0.8466819221967964
2947 P16 C38 Post-cholecystectomy Post-op CD
0.8419145690312738
2948 P16 C40 Post-cholecystectomy Post-op CD
0.7745995423340961
2949 P16 C44 Post-cholecystectomy Post-op CD
0.8688024408848207
2950 P16 C47 Post-cholecystectomy Post-op CD
0.9187643020594966
2951 P16 C48 Post-cholecystectomy Post-op CD
0.8632723112128147
2952 P16 C49 Post-cholecystectomy Post-op CD

0.7644927536231884
2953	P16	C53	Post-cholecystectomy	Post-op CD
0.8846300533943554
2954	P16	C56	Post-cholecystectomy	Post-op CD
0.8367658276125095
2955	P16	C60	Post-cholecystectomy	Post-op CD
0.8327612509534706
2956	P16	C62	Post-cholecystectomy	Post-op CD
0.8846300533943554
2957	P16	C64	Post-cholecystectomy	Post-op CD
0.9096109839816934
2958	P16	C65	Post-cholecystectomy	Post-op CD
0.9170480549199085
2959	P16	C69	Post-cholecystectomy	Post-op CD
0.7845156369183829
2960	P16	C70	Post-cholecystectomy	Post-op CD
0.9155225019069413
2961	P16	C74	Post-cholecystectomy	Post-op CD
0.9361174675819984
2962	P16	C78	Post-cholecystectomy	Post-op CD
0.7069031273836766
2963	P17	C1	Post-cholecystectomy	Post-op CD
0.9799771167048055
2964	P17	C3	Post-cholecystectomy	Post-op CD
0.9183829138062548
2965	P17	C7	Post-cholecystectomy	Post-op CD
0.9069412662090007
2966	P17	C8	Post-cholecystectomy	Post-op CD
0.9662471395881007
2967	P17	C11	Post-cholecystectomy	Post-op CD
0.8194126620900076
2968	P17	C15	Post-cholecystectomy	Post-op CD
0.8503051106025934
2969	P17	C19	Post-cholecystectomy	Post-op CD
0.8228451563691839
2970	P17	C22	Post-cholecystectomy	Post-op CD
0.9628146453089245
2971	P17	C26	Post-cholecystectomy	Post-op CD
0.9734935163996948
2972	P17	C28	Post-cholecystectomy	Post-op CD
0.8821510297482837
2973	P17	C31	Post-cholecystectomy	Post-op CD
0.992372234935164
2974	P17	C35	Post-cholecystectomy	Post-op CD
0.8565980167810832
2975	P17	C38	Post-cholecystectomy	Post-op CD
0.9717772692601068
2976	P17	C40	Post-cholecystectomy	Post-op CD
0.9658657513348589
2977	P17	C44	Post-cholecystectomy	Post-op CD
0.8443935926773455
2978	P17	C47	Post-cholecystectomy	Post-op CD
0.8585049580472921
2979	P17	C48	Post-cholecystectomy	Post-op CD

0.8636536994660564
2980 P17 C49 Post-cholecystectomy Post-op CD
0.9193363844393593
2981 P17 C53 Post-cholecystectomy Post-op CD
0.8312356979405034
2982 P17 C56 Post-cholecystectomy Post-op CD
0.8348588863463006
2983 P17 C60 Post-cholecystectomy Post-op CD
0.8623188405797102
2984 P17 C62 Post-cholecystectomy Post-op CD
0.9218154080854309
2985 P17 C64 Post-cholecystectomy Post-op CD
0.8918764302059496
2986 P17 C65 Post-cholecystectomy Post-op CD
0.9036994660564455
2987 P17 C69 Post-cholecystectomy Post-op CD
0.8781464530892449
2988 P17 C70 Post-cholecystectomy Post-op CD
0.9080854309687262
2989 P17 C74 Post-cholecystectomy Post-op CD
0.9241037376048817
2990 P17 C78 Post-cholecystectomy Post-op CD
0.8401983218916858
2991 P20 C1 Post-cholecystectomy Post-op CD
0.8670861937452327
2992 P20 C3 Post-cholecystectomy Post-op CD
0.868230358504958
2993 P20 C7 Post-cholecystectomy Post-op CD
0.9303966437833715
2994 P20 C8 Post-cholecystectomy Post-op CD
0.948512585812357
2995 P20 C11 Post-cholecystectomy Post-op CD
0.9078947368421053
2996 P20 C15 Post-cholecystectomy Post-op CD
0.8182684973302822
2997 P20 C19 Post-cholecystectomy Post-op CD
0.8525934401220442
2998 P20 C22 Post-cholecystectomy Post-op CD
0.894927536231884
2999 P20 C26 Post-cholecystectomy Post-op CD
0.7240655987795576
3000 P20 C28 Post-cholecystectomy Post-op CD
0.858886346300534
3001 P20 C31 Post-cholecystectomy Post-op CD
0.9176201372997712
3002 P20 C35 Post-cholecystectomy Post-op CD
0.9033180778032036
3003 P20 C38 Post-cholecystectomy Post-op CD
0.9528985507246377
3004 P20 C40 Post-cholecystectomy Post-op CD
0.6601830663615561
3005 P20 C44 Post-cholecystectomy Post-op CD
0.9344012204424104
3006 P20 C47 Post-cholecystectomy Post-op CD

0.9702517162471396
3007	P20	C48	Post-cholecystectomy	Post-op CD
0.8945461479786423
3008	P20	C49	Post-cholecystectomy	Post-op CD
0.6924103737604882
3009	P20	C53	Post-cholecystectomy	Post-op CD
0.9225781845919145
3010	P20	C56	Post-cholecystectomy	Post-op CD
0.8222730739893211
3011	P20	C60	Post-cholecystectomy	Post-op CD
0.8247520976353928
3012	P20	C62	Post-cholecystectomy	Post-op CD
0.8724256292906178
3013	P20	C64	Post-cholecystectomy	Post-op CD
0.9050343249427918
3014	P20	C65	Post-cholecystectomy	Post-op CD
0.8667048054919908
3015	P20	C69	Post-cholecystectomy	Post-op CD
0.7740274599542334
3016	P20	C70	Post-cholecystectomy	Post-op CD
0.9080854309687262
3017	P20	C74	Post-cholecystectomy	Post-op CD
0.8632723112128147
3018	P20	C78	Post-cholecystectomy	Post-op CD
0.7702135774218154
3019	P21	C1	Post-cholecystectomy	Post-op CD
0.8562166285278413
3020	P21	C3	Post-cholecystectomy	Post-op CD
0.9364988558352403
3021	P21	C7	Post-cholecystectomy	Post-op CD
0.8792906178489702
3022	P21	C8	Post-cholecystectomy	Post-op CD
0.9487032799389779
3023	P21	C11	Post-cholecystectomy	Post-op CD
0.8058733790999237
3024	P21	C15	Post-cholecystectomy	Post-op CD
0.7726926010678871
3025	P21	C19	Post-cholecystectomy	Post-op CD
0.801487414187643
3026	P21	C22	Post-cholecystectomy	Post-op CD
0.938977879481312
3027	P21	C26	Post-cholecystectomy	Post-op CD
0.8642257818459191
3028	P21	C28	Post-cholecystectomy	Post-op CD
0.7280701754385965
3029	P21	C31	Post-cholecystectomy	Post-op CD
0.6796338672768879
3030	P21	C35	Post-cholecystectomy	Post-op CD
0.8272311212814645
3031	P21	C38	Post-cholecystectomy	Post-op CD
0.914187643020595
3032	P21	C40	Post-cholecystectomy	Post-op CD
0.8127383676582761
3033	P21	C44	Post-cholecystectomy	Post-op CD

0.8230358504958047
3034	P21	C47	Post-cholecystectomy	Post-op CD
0.8848207475209764
3035	P21	C48	Post-cholecystectomy	Post-op CD
0.8926392067124332
3036	P21	C49	Post-cholecystectomy	Post-op CD
0.9317315026697178
3037	P21	C53	Post-cholecystectomy	Post-op CD
0.8409610983981693
3038	P21	C56	Post-cholecystectomy	Post-op CD
0.729023646071701
3039	P21	C60	Post-cholecystectomy	Post-op CD
0.8424866514111365
3040	P21	C62	Post-cholecystectomy	Post-op CD
0.8754767353165522
3041	P21	C64	Post-cholecystectomy	Post-op CD
0.8789092295957285
3042	P21	C65	Post-cholecystectomy	Post-op CD
0.9229595728451564
3043	P21	C69	Post-cholecystectomy	Post-op CD
0.8548817696414951
3044	P21	C70	Post-cholecystectomy	Post-op CD
0.9189549961861174
3045	P21	C74	Post-cholecystectomy	Post-op CD
0.9441266209000763
3046	P21	C78	Post-cholecystectomy	Post-op CD
0.7599160945842868
3047	P24	C1	Post-cholecystectomy	Post-op CD
0.9807398932112891
3048	P24	C3	Post-cholecystectomy	Post-op CD
0.9185736079328757
3049	P24	C7	Post-cholecystectomy	Post-op CD
0.9641495041952708
3050	P24	C8	Post-cholecystectomy	Post-op CD
0.9487032799389779
3051	P24	C11	Post-cholecystectomy	Post-op CD
0.973302822273074
3052	P24	C15	Post-cholecystectomy	Post-op CD
0.9477498093058734
3053	P24	C19	Post-cholecystectomy	Post-op CD
0.9685354691075515
3054	P24	C22	Post-cholecystectomy	Post-op CD
0.8903508771929824
3055	P24	C26	Post-cholecystectomy	Post-op CD
0.9557589626239512
3056	P24	C28	Post-cholecystectomy	Post-op CD
0.9279176201372997
3057	P24	C31	Post-cholecystectomy	Post-op CD
0.9515636918382914
3058	P24	C35	Post-cholecystectomy	Post-op CD
0.9443173150266971
3059	P24	C38	Post-cholecystectomy	Post-op CD
0.9830282227307399
3060	P24	C40	Post-cholecystectomy	Post-op CD

0.9450800915331807
3061	P24	C44	Post-cholecystectomy	Post-op CD
0.9784515636918383
3062	P24	C47	Post-cholecystectomy	Post-op CD
0.9774980930587338
3063	P24	C48	Post-cholecystectomy	Post-op CD
0.8545003813882532
3064	P24	C49	Post-cholecystectomy	Post-op CD
0.8712814645308925
3065	P24	C53	Post-cholecystectomy	Post-op CD
0.8842486651411137
3066	P24	C56	Post-cholecystectomy	Post-op CD
0.8180778032036613
3067	P24	C60	Post-cholecystectomy	Post-op CD
0.8983600305110603
3068	P24	C62	Post-cholecystectomy	Post-op CD
0.8726163234172387
3069	P24	C64	Post-cholecystectomy	Post-op CD
0.851258581235698
3070	P24	C65	Post-cholecystectomy	Post-op CD
0.8541189931350115
3071	P24	C69	Post-cholecystectomy	Post-op CD
0.8482074752097636
3072	P24	C70	Post-cholecystectomy	Post-op CD
0.8520213577421816
3073	P24	C74	Post-cholecystectomy	Post-op CD
0.9012204424103738
3074	P24	C78	Post-cholecystectomy	Post-op CD
0.9422196796338673
3075	P26	C1	Post-cholecystectomy	Post-op CD
0.8697559115179252
3076	P26	C3	Post-cholecystectomy	Post-op CD
0.9765446224256293
3077	P26	C7	Post-cholecystectomy	Post-op CD
0.8872997711670481
3078	P26	C8	Post-cholecystectomy	Post-op CD
0.9593821510297483
3079	P26	C11	Post-cholecystectomy	Post-op CD
0.6941266209000763
3080	P26	C15	Post-cholecystectomy	Post-op CD
0.702326468344775
3081	P26	C19	Post-cholecystectomy	Post-op CD
0.7065217391304348
3082	P26	C22	Post-cholecystectomy	Post-op CD
0.9530892448512586
3083	P26	C26	Post-cholecystectomy	Post-op CD
0.8735697940503433
3084	P26	C28	Post-cholecystectomy	Post-op CD
0.5217391304347826
3085	P26	C31	Post-cholecystectomy	Post-op CD
0.780511060259344
3086	P26	C35	Post-cholecystectomy	Post-op CD
0.6773455377574371
3087	P26	C38	Post-cholecystectomy	Post-op CD

0.9710144927536232
3088 P26 C40 Post-cholecystectomy Post-op CD
0.8886346300533944
3089 P26 C44 Post-cholecystectomy Post-op CD
0.6939359267734554
3090 P26 C47 Post-cholecystectomy Post-op CD
0.7580091533180778
3091 P26 C48 Post-cholecystectomy Post-op CD
0.9164759725400458
3092 P26 C49 Post-cholecystectomy Post-op CD
0.925629290617849
3093 P26 C53 Post-cholecystectomy Post-op CD
0.7991990846681922
3094 P26 C56 Post-cholecystectomy Post-op CD
0.757627765064836
3095 P26 C60 Post-cholecystectomy Post-op CD
0.778604118993135
3096 P26 C62 Post-cholecystectomy Post-op CD
0.8886346300533944
3097 P26 C64 Post-cholecystectomy Post-op CD
0.8939740655987796
3098 P26 C65 Post-cholecystectomy Post-op CD
0.9513729977116705
3099 P26 C69 Post-cholecystectomy Post-op CD
0.8161708619374524
3100 P26 C70 Post-cholecystectomy Post-op CD
0.944698703279939
3101 P26 C74 Post-cholecystectomy Post-op CD
0.9570938215102975
3102 P26 C78 Post-cholecystectomy Post-op CD
0.7057589626239512
3103 P30 C1 Post-cholecystectomy Post-op CD
0.9227688787185355
3104 P30 C3 Post-cholecystectomy Post-op CD
0.9223874904652937
3105 P30 C7 Post-cholecystectomy Post-op CD
0.9795957284515637
3106 P30 C8 Post-cholecystectomy Post-op CD
0.9323035850495804
3107 P30 C11 Post-cholecystectomy Post-op CD
0.9727307398932112
3108 P30 C15 Post-cholecystectomy Post-op CD
0.9427917620137299
3109 P30 C19 Post-cholecystectomy Post-op CD
0.9725400457665904
3110 P30 C22 Post-cholecystectomy Post-op CD
0.9101830663615561
3111 P30 C26 Post-cholecystectomy Post-op CD
0.9382151029748284
3112 P30 C28 Post-cholecystectomy Post-op CD
0.950419527078566
3113 P30 C31 Post-cholecystectomy Post-op CD
0.9290617848970252
3114 P30 C35 Post-cholecystectomy Post-op CD

0.9515636918382914
3115 P30 C38 Post-cholecystectomy Post-op CD
0.8665141113653699
3116 P30 C40 Post-cholecystectomy Post-op CD
0.8878718535469108
3117 P30 C44 Post-cholecystectomy Post-op CD
0.9750190694126621
3118 P30 C47 Post-cholecystectomy Post-op CD
0.9780701754385965
3119 P30 C48 Post-cholecystectomy Post-op CD
0.9263920671243325
3120 P30 C49 Post-cholecystectomy Post-op CD
0.9029366895499619
3121 P30 C53 Post-cholecystectomy Post-op CD
0.9494660564454614
3122 P30 C56 Post-cholecystectomy Post-op CD
0.9193363844393593
3123 P30 C60 Post-cholecystectomy Post-op CD
0.8627002288329519
3124 P30 C62 Post-cholecystectomy Post-op CD
0.916094584286804
3125 P30 C64 Post-cholecystectomy Post-op CD
0.8832951945080092
3126 P30 C65 Post-cholecystectomy Post-op CD
0.8939740655987796
3127 P30 C69 Post-cholecystectomy Post-op CD
0.918001525553013
3128 P30 C70 Post-cholecystectomy Post-op CD
0.9429824561403509
3129 P30 C74 Post-cholecystectomy Post-op CD
0.9473684210526315
3130 P30 C78 Post-cholecystectomy Post-op CD
0.9555682684973302
3131 P33 C1 Post-cholecystectomy Post-op CD
0.9546147978642258
3132 P33 C3 Post-cholecystectomy Post-op CD
0.9439359267734554
3133 P33 C7 Post-cholecystectomy Post-op CD
0.8880625476735317
3134 P33 C8 Post-cholecystectomy Post-op CD
0.9233409610983981
3135 P33 C11 Post-cholecystectomy Post-op CD
0.8602212051868803
3136 P33 C15 Post-cholecystectomy Post-op CD
0.8135011441647597
3137 P33 C19 Post-cholecystectomy Post-op CD
0.8350495804729214
3138 P33 C22 Post-cholecystectomy Post-op CD
0.9328756674294432
3139 P33 C26 Post-cholecystectomy Post-op CD
0.9122807017543859
3140 P33 C28 Post-cholecystectomy Post-op CD
0.7967200610221206
3141 P33 C31 Post-cholecystectomy Post-op CD

0.9191456903127384
3142	P33	C35	Post-cholecystectomy	Post-op CD
0.8390541571319603
3143	P33	C38	Post-cholecystectomy	Post-op CD
0.935163996948894
3144	P33	C40	Post-cholecystectomy	Post-op CD
0.8710907704042715
3145	P33	C44	Post-cholecystectomy	Post-op CD
0.8062547673531655
3146	P33	C47	Post-cholecystectomy	Post-op CD
0.9117086193745233
3147	P33	C48	Post-cholecystectomy	Post-op CD
0.8667048054919908
3148	P33	C49	Post-cholecystectomy	Post-op CD
0.9040808543096872
3149	P33	C53	Post-cholecystectomy	Post-op CD
0.8403890160183066
3150	P33	C56	Post-cholecystectomy	Post-op CD
0.7911899313501144
3151	P33	C60	Post-cholecystectomy	Post-op CD
0.82627765064836
3152	P33	C62	Post-cholecystectomy	Post-op CD
0.8800533943554538
3153	P33	C64	Post-cholecystectomy	Post-op CD
0.8686117467581999
3154	P33	C65	Post-cholecystectomy	Post-op CD
0.8779557589626239
3155	P33	C69	Post-cholecystectomy	Post-op CD
0.8560259344012204
3156	P33	C70	Post-cholecystectomy	Post-op CD
0.9088482074752098
3157	P33	C74	Post-cholecystectomy	Post-op CD
0.9315408085430968
3158	P33	C78	Post-cholecystectomy	Post-op CD
0.7953852021357742
3159	P35	C1	Post-cholecystectomy	Post-op CD
0.9591914569031273
3160	P35	C3	Post-cholecystectomy	Post-op CD
0.9437452326468345
3161	P35	C7	Post-cholecystectomy	Post-op CD
0.8968344774980931
3162	P35	C8	Post-cholecystectomy	Post-op CD
0.9479405034324943
3163	P35	C11	Post-cholecystectomy	Post-op CD
0.7940503432494279
3164	P35	C15	Post-cholecystectomy	Post-op CD
0.8222730739893211
3165	P35	C19	Post-cholecystectomy	Post-op CD
0.7713577421815409
3166	P35	C22	Post-cholecystectomy	Post-op CD
0.9691075514874142
3167	P35	C26	Post-cholecystectomy	Post-op CD
0.897025171624714
3168	P35	C28	Post-cholecystectomy	Post-op CD

0.8104500381388253
3169 P35 C31 Post-cholecystectomy Post-op CD
0.8569794050343249
3170 P35 C35 Post-cholecystectomy Post-op CD
0.7717391304347826
3171 P35 C38 Post-cholecystectomy Post-op CD
0.9774980930587338
3172 P35 C40 Post-cholecystectomy Post-op CD
0.9225781845919145
3173 P35 C44 Post-cholecystectomy Post-op CD
0.8236079328756675
3174 P35 C47 Post-cholecystectomy Post-op CD
0.8636536994660564
3175 P35 C48 Post-cholecystectomy Post-op CD
0.8495423340961098
3176 P35 C49 Post-cholecystectomy Post-op CD
0.9300152555301296
3177 P35 C53 Post-cholecystectomy Post-op CD
0.8033943554538521
3178 P35 C56 Post-cholecystectomy Post-op CD
0.7873760488176964
3179 P35 C60 Post-cholecystectomy Post-op CD
0.8178871090770404
3180 P35 C62 Post-cholecystectomy Post-op CD
0.8850114416475973
3181 P35 C64 Post-cholecystectomy Post-op CD
0.8737604881769642
3182 P35 C65 Post-cholecystectomy Post-op CD
0.9302059496567505
3183 P35 C69 Post-cholecystectomy Post-op CD
0.8768115942028986
3184 P35 C70 Post-cholecystectomy Post-op CD
0.92372234935164
3185 P35 C74 Post-cholecystectomy Post-op CD
0.933066361556064
3186 P35 C78 Post-cholecystectomy Post-op CD
0.7505720823798627
3187 P38 C1 Post-cholecystectomy Post-op CD
0.9374523264683448
3188 P38 C3 Post-cholecystectomy Post-op CD
0.9122807017543859
3189 P38 C7 Post-cholecystectomy Post-op CD
0.9815026697177727
3190 P38 C8 Post-cholecystectomy Post-op CD
0.936880244088482
3191 P38 C11 Post-cholecystectomy Post-op CD
0.8749046529366895
3192 P38 C15 Post-cholecystectomy Post-op CD
0.9263920671243325
3193 P38 C19 Post-cholecystectomy Post-op CD
0.9698703279938978
3194 P38 C22 Post-cholecystectomy Post-op CD
0.9029366895499619
3195 P38 C26 Post-cholecystectomy Post-op CD

0.879862700228833
3196	P38	C28	Post-cholecystectomy	Post-op CD
0.9338291380625476
3197	P38	C31	Post-cholecystectomy	Post-op CD
0.8182684973302822
3198	P38	C35	Post-cholecystectomy	Post-op CD
0.9355453852021358
3199	P38	C38	Post-cholecystectomy	Post-op CD
0.8709000762776506
3200	P38	C40	Post-cholecystectomy	Post-op CD
0.8899694889397407
3201	P38	C44	Post-cholecystectomy	Post-op CD
0.973302822273074
3202	P38	C47	Post-cholecystectomy	Post-op CD
0.9794050343249427
3203	P38	C48	Post-cholecystectomy	Post-op CD
0.933257055682685
3204	P38	C49	Post-cholecystectomy	Post-op CD
0.9281083142639207
3205	P38	C53	Post-cholecystectomy	Post-op CD
0.9328756674294432
3206	P38	C56	Post-cholecystectomy	Post-op CD
0.9359267734553776
3207	P38	C60	Post-cholecystectomy	Post-op CD
0.9105644546147978
3208	P38	C62	Post-cholecystectomy	Post-op CD
0.795957284515637
3209	P38	C64	Post-cholecystectomy	Post-op CD
0.6024027459954233
3210	P38	C65	Post-cholecystectomy	Post-op CD
0.8794813119755912
3211	P38	C69	Post-cholecystectomy	Post-op CD
0.9019832189168574
3212	P38	C70	Post-cholecystectomy	Post-op CD
0.8975972540045767
3213	P38	C74	Post-cholecystectomy	Post-op CD
0.9595728451563692
3214	P38	C78	Post-cholecystectomy	Post-op CD
0.9610983981693364
3215	P39	C1	Post-cholecystectomy	Post-op CD
0.8775743707093822
3216	P39	C3	Post-cholecystectomy	Post-op CD
0.9445080091533181
3217	P39	C7	Post-cholecystectomy	Post-op CD
0.9317315026697178
3218	P39	C8	Post-cholecystectomy	Post-op CD
0.9170480549199085
3219	P39	C11	Post-cholecystectomy	Post-op CD
0.9168573607932876
3220	P39	C15	Post-cholecystectomy	Post-op CD
0.8813882532418001
3221	P39	C19	Post-cholecystectomy	Post-op CD
0.9221967963386728
3222	P39	C22	Post-cholecystectomy	Post-op CD

0.9288710907704043
3223	P39	C26	Post-cholecystectomy	Post-op CD
0.7816552250190694
3224	P39	C28	Post-cholecystectomy	Post-op CD
0.8840579710144928
3225	P39	C31	Post-cholecystectomy	Post-op CD
0.881769641495042
3226	P39	C35	Post-cholecystectomy	Post-op CD
0.947177269260107
3227	P39	C38	Post-cholecystectomy	Post-op CD
0.8836765827612509
3228	P39	C40	Post-cholecystectomy	Post-op CD
0.7807017543859649
3229	P39	C44	Post-cholecystectomy	Post-op CD
0.9506102212051869
3230	P39	C47	Post-cholecystectomy	Post-op CD
0.973302822273074
3231	P39	C48	Post-cholecystectomy	Post-op CD
0.897025171624714
3232	P39	C49	Post-cholecystectomy	Post-op CD
0.872234935163997
3233	P39	C53	Post-cholecystectomy	Post-op CD
0.9544241037376049
3234	P39	C56	Post-cholecystectomy	Post-op CD
0.8422959572845157
3235	P39	C60	Post-cholecystectomy	Post-op CD
0.8977879481311976
3236	P39	C62	Post-cholecystectomy	Post-op CD
0.8794813119755912
3237	P39	C64	Post-cholecystectomy	Post-op CD
0.9277269260106789
3238	P39	C65	Post-cholecystectomy	Post-op CD
0.9481311975591151
3239	P39	C69	Post-cholecystectomy	Post-op CD
0.8585049580472921
3240	P39	C70	Post-cholecystectomy	Post-op CD
0.9017925247902364
3241	P39	C74	Post-cholecystectomy	Post-op CD
0.9387871853546911
3242	P39	C78	Post-cholecystectomy	Post-op CD
0.8161708619374524
3243	P42	C1	Post-cholecystectomy	Post-op CD
0.9242944317315027
3244	P42	C3	Post-cholecystectomy	Post-op CD
0.9744469870327994
3245	P42	C7	Post-cholecystectomy	Post-op CD
0.9265827612509535
3246	P42	C8	Post-cholecystectomy	Post-op CD
0.9555682684973302
3247	P42	C11	Post-cholecystectomy	Post-op CD
0.5221205186880244
3248	P42	C15	Post-cholecystectomy	Post-op CD
0.6273836765827613
3249	P42	C19	Post-cholecystectomy	Post-op CD

0.6292906178489702
3250	P42	C22	Post-cholecystectomy	Post-op CD
0.9439359267734554
3251	P42	C26	Post-cholecystectomy	Post-op CD
0.9479405034324943
3252	P42	C28	Post-cholecystectomy	Post-op CD
0.7486651411136537
3253	P42	C31	Post-cholecystectomy	Post-op CD
0.9014111365369947
3254	P42	C35	Post-cholecystectomy	Post-op CD
0.6468344774980931
3255	P42	C38	Post-cholecystectomy	Post-op CD
0.9115179252479023
3256	P42	C40	Post-cholecystectomy	Post-op CD
0.9363081617086194
3257	P42	C44	Post-cholecystectomy	Post-op CD
0.5659801678108314
3258	P42	C47	Post-cholecystectomy	Post-op CD
0.5705568268497331
3259	P42	C48	Post-cholecystectomy	Post-op CD
0.9113272311212814
3260	P42	C49	Post-cholecystectomy	Post-op CD
0.9187643020594966
3261	P42	C53	Post-cholecystectomy	Post-op CD
0.7088100686498856
3262	P42	C56	Post-cholecystectomy	Post-op CD
0.8123569794050344
3263	P42	C60	Post-cholecystectomy	Post-op CD
0.7236842105263158
3264	P42	C62	Post-cholecystectomy	Post-op CD
0.8867276887871853
3265	P42	C64	Post-cholecystectomy	Post-op CD
0.9078947368421053
3266	P42	C65	Post-cholecystectomy	Post-op CD
0.9258199847444699
3267	P42	C69	Post-cholecystectomy	Post-op CD
0.8991228070175439
3268	P42	C70	Post-cholecystectomy	Post-op CD
0.9305873379099924
3269	P42	C74	Post-cholecystectomy	Post-op CD
0.9670099160945843
3270	P42	C78	Post-cholecystectomy	Post-op CD
0.8047292143401983
3271	P43	C1	Post-cholecystectomy	Post-op CD
0.9176201372997712
3272	P43	C3	Post-cholecystectomy	Post-op CD
0.9666285278413425
3273	P43	C7	Post-cholecystectomy	Post-op CD
0.9378337147215866
3274	P43	C8	Post-cholecystectomy	Post-op CD
0.9660564454614798
3275	P43	C11	Post-cholecystectomy	Post-op CD
0.8947368421052632
3276	P43	C15	Post-cholecystectomy	Post-op CD

0.8924485125858124
3277 P43 C19 Post-cholecystectomy Post-op CD
0.9195270785659801
3278 P43 C22 Post-cholecystectomy Post-op CD
0.9590007627765065
3279 P43 C26 Post-cholecystectomy Post-op CD
0.851258581235698
3280 P43 C28 Post-cholecystectomy Post-op CD
0.8630816170861938
3281 P43 C31 Post-cholecystectomy Post-op CD
0.9406941266209001
3282 P43 C35 Post-cholecystectomy Post-op CD
0.9090389016018307
3283 P43 C38 Post-cholecystectomy Post-op CD
0.937070938215103
3284 P43 C40 Post-cholecystectomy Post-op CD
0.7646834477498093
3285 P43 C44 Post-cholecystectomy Post-op CD
0.9509916094584286
3286 P43 C47 Post-cholecystectomy Post-op CD
0.9851258581235698
3287 P43 C48 Post-cholecystectomy Post-op CD
0.9088482074752098
3288 P43 C49 Post-cholecystectomy Post-op CD
0.8548817696414951
3289 P43 C53 Post-cholecystectomy Post-op CD
0.9315408085430968
3290 P43 C56 Post-cholecystectomy Post-op CD
0.8497330282227308
3291 P43 C60 Post-cholecystectomy Post-op CD
0.910373760488177
3292 P43 C62 Post-cholecystectomy Post-op CD
0.9181922196796338
3293 P43 C64 Post-cholecystectomy Post-op CD
0.9157131960335622
3294 P43 C65 Post-cholecystectomy Post-op CD
0.9328756674294432
3295 P43 C69 Post-cholecystectomy Post-op CD
0.8037757437070938
3296 P43 C70 Post-cholecystectomy Post-op CD
0.8941647597254004
3297 P43 C74 Post-cholecystectomy Post-op CD
0.9443173150266971
3298 P43 C78 Post-cholecystectomy Post-op CD
0.6821128909229596
3299 P46 C1 Post-cholecystectomy Post-op CD
0.9357360793287567
3300 P46 C3 Post-cholecystectomy Post-op CD
0.9391685736079328
3301 P46 C7 Post-cholecystectomy Post-op CD
0.6977498093058734
3302 P46 C8 Post-cholecystectomy Post-op CD
0.9494660564454614
3303 P46 C11 Post-cholecystectomy Post-op CD

0.8886346300533944
3304	P46	C15	Post-cholecystectomy	Post-op CD
0.8483981693363845
3305	P46	C19	Post-cholecystectomy	Post-op CD
0.7238749046529367
3306	P46	C22	Post-cholecystectomy	Post-op CD
0.9546147978642258
3307	P46	C26	Post-cholecystectomy	Post-op CD
0.8720442410373761
3308	P46	C28	Post-cholecystectomy	Post-op CD
0.7803203661327232
3309	P46	C31	Post-cholecystectomy	Post-op CD
0.8895881006864989
3310	P46	C35	Post-cholecystectomy	Post-op CD
0.759534706331045
3311	P46	C38	Post-cholecystectomy	Post-op CD
0.9372616323417239
3312	P46	C40	Post-cholecystectomy	Post-op CD
0.8697559115179252
3313	P46	C44	Post-cholecystectomy	Post-op CD
0.8625095347063311
3314	P46	C47	Post-cholecystectomy	Post-op CD
0.8663234172387491
3315	P46	C48	Post-cholecystectomy	Post-op CD
0.6899313501144165
3316	P46	C49	Post-cholecystectomy	Post-op CD
0.7915713196033562
3317	P46	C53	Post-cholecystectomy	Post-op CD
0.7873760488176964
3318	P46	C56	Post-cholecystectomy	Post-op CD
0.732837528604119
3319	P46	C60	Post-cholecystectomy	Post-op CD
0.7816552250190694
3320	P46	C62	Post-cholecystectomy	Post-op CD
0.7465675057208238
3321	P46	C64	Post-cholecystectomy	Post-op CD
0.7930968726163234
3322	P46	C65	Post-cholecystectomy	Post-op CD
0.7971014492753623
3323	P46	C69	Post-cholecystectomy	Post-op CD
0.6466437833714722
3324	P46	C70	Post-cholecystectomy	Post-op CD
0.7631578947368421
3325	P46	C74	Post-cholecystectomy	Post-op CD
0.834096109839817
3326	P46	C78	Post-cholecystectomy	Post-op CD
0.7164378337147216
3327	P47	C1	Post-cholecystectomy	Post-op CD
0.9479405034324943
3328	P47	C3	Post-cholecystectomy	Post-op CD
0.9391685736079328
3329	P47	C7	Post-cholecystectomy	Post-op CD
0.37090007627765065
3330	P47	C8	Post-cholecystectomy	Post-op CD

0.9551868802440885
3331 P47 C11 Post-cholecystectomy Post-op CD
0.9578565980167811
3332 P47 C15 Post-cholecystectomy Post-op CD
0.9782608695652174
3333 P47 C19 Post-cholecystectomy Post-op CD
0.5568268497330282
3334 P47 C22 Post-cholecystectomy Post-op CD
0.9366895499618612
3335 P47 C26 Post-cholecystectomy Post-op CD
0.9757818459191457
3336 P47 C28 Post-cholecystectomy Post-op CD
0.9450800915331807
3337 P47 C31 Post-cholecystectomy Post-op CD
0.9666285278413425
3338 P47 C35 Post-cholecystectomy Post-op CD
0.7562929061784897
3339 P47 C38 Post-cholecystectomy Post-op CD
0.9727307398932112
3340 P47 C40 Post-cholecystectomy Post-op CD
0.9578565980167811
3341 P47 C44 Post-cholecystectomy Post-op CD
0.9757818459191457
3342 P47 C47 Post-cholecystectomy Post-op CD
0.9706331045003814
3343 P47 C48 Post-cholecystectomy Post-op CD
0.6596109839816934
3344 P47 C49 Post-cholecystectomy Post-op CD
0.8545003813882532
3345 P47 C53 Post-cholecystectomy Post-op CD
0.816742944317315
3346 P47 C56 Post-cholecystectomy Post-op CD
0.8176964149504196
3347 P47 C60 Post-cholecystectomy Post-op CD
0.8165522501906941
3348 P47 C62 Post-cholecystectomy Post-op CD
0.8495423340961098
3349 P47 C64 Post-cholecystectomy Post-op CD
0.8247520976353928
3350 P47 C65 Post-cholecystectomy Post-op CD
0.8119755911517925
3351 P47 C69 Post-cholecystectomy Post-op CD
0.8173150266971777
3352 P47 C70 Post-cholecystectomy Post-op CD
0.8386727688787186
3353 P47 C74 Post-cholecystectomy Post-op CD
0.8504958047292144
3354 P47 C78 Post-cholecystectomy Post-op CD
0.9715865751334859
3355 P50 C1 Post-cholecystectomy Post-op CD
0.9364988558352403
3356 P50 C3 Post-cholecystectomy Post-op CD
0.8930205949656751
3357 P50 C7 Post-cholecystectomy Post-op CD

0.8583142639206712
3358 P50 C8 Post-cholecystectomy Post-op CD
0.9279176201372997
3359 P50 C11 Post-cholecystectomy Post-op CD
0.8283752860411899
3360 P50 C15 Post-cholecystectomy Post-op CD
0.8487795575896262
3361 P50 C19 Post-cholecystectomy Post-op CD
0.7869946605644547
3362 P50 C22 Post-cholecystectomy Post-op CD
0.9378337147215866
3363 P50 C26 Post-cholecystectomy Post-op CD
0.915903890160183
3364 P50 C28 Post-cholecystectomy Post-op CD
0.8390541571319603
3365 P50 C31 Post-cholecystectomy Post-op CD
0.9588100686498856
3366 P50 C35 Post-cholecystectomy Post-op CD
0.8226544622425629
3367 P50 C38 Post-cholecystectomy Post-op CD
0.9698703279938978
3368 P50 C40 Post-cholecystectomy Post-op CD
0.9321128909229596
3369 P50 C44 Post-cholecystectomy Post-op CD
0.8323798627002288
3370 P50 C47 Post-cholecystectomy Post-op CD
0.8800533943554538
3371 P50 C48 Post-cholecystectomy Post-op CD
0.8142639206712433
3372 P50 C49 Post-cholecystectomy Post-op CD
0.9046529366895499
3373 P50 C53 Post-cholecystectomy Post-op CD
0.8115942028985508
3374 P50 C56 Post-cholecystectomy Post-op CD
0.7526697177726926
3375 P50 C60 Post-cholecystectomy Post-op CD
0.7898550724637681
3376 P50 C62 Post-cholecystectomy Post-op CD
0.8838672768878718
3377 P50 C64 Post-cholecystectomy Post-op CD
0.8381006864988558
3378 P50 C65 Post-cholecystectomy Post-op CD
0.8766209000762777
3379 P50 C69 Post-cholecystectomy Post-op CD
0.8377192982456141
3380 P50 C70 Post-cholecystectomy Post-op CD
0.8899694889397407
3381 P50 C74 Post-cholecystectomy Post-op CD
0.9002669717772692
3382 P50 C78 Post-cholecystectomy Post-op CD
0.7145308924485125
3383 P55 C1 Post-cholecystectomy Post-op CD
0.9572845156369184
3384 P55 C3 Post-cholecystectomy Post-op CD

0.898932112890923
3385	P55	C7	Post-cholecystectomy	Post-op CD
0.918001525553013
3386	P55	C8	Post-cholecystectomy	Post-op CD
0.924866514111653
3387	P55	C11	Post-cholecystectomy	Post-op CD
0.788710907704042 7
3388	P55	C15	Post-cholecystectomy	Post-op CD
0.780511060259344
3389	P55	C19	Post-cholecystectomy	Post-op CD
0.772692601067887 1
3390	P55	C22	Post-cholecystectomy	Post-op CD
0.946987032799389 8
3391	P55	C26	Post-cholecystectomy	Post-op CD
0.956331045003813 8
3392	P55	C28	Post-cholecystectomy	Post-op CD
0.799771167048055
3393	P55	C31	Post-cholecystectomy	Post-op CD
0.980358504958047 3
3394	P55	C35	Post-cholecystectomy	Post-op CD
0.795003813882532 4
3395	P55	C38	Post-cholecystectomy	Post-op CD
0.961670480549199 1
3396	P55	C40	Post-cholecystectomy	Post-op CD
0.934210526315789 5
3397	P55	C44	Post-cholecystectomy	Post-op CD
0.802631578947368 5
3398	P55	C47	Post-cholecystectomy	Post-op CD
0.825324180015255 5
3399	P55	C48	Post-cholecystectomy	Post-op CD
0.876239511823035 8
3400	P55	C49	Post-cholecystectomy	Post-op CD
0.922006102212051 8
3401	P55	C53	Post-cholecystectomy	Post-op CD
0.794050343249427 9
3402	P55	C56	Post-cholecystectomy	Post-op CD
0.747520976353928 3
3403	P55	C60	Post-cholecystectomy	Post-op CD
0.737604881769641 5
3404	P55	C62	Post-cholecystectomy	Post-op CD
0.910755148741418 7
3405	P55	C64	Post-cholecystectomy	Post-op CD
0.894355453852021 3
3406	P55	C65	Post-cholecystectomy	Post-op CD
0.894927536231884
3407	P55	C69	Post-cholecystectomy	Post-op CD
0.868611746758199 9
3408	P55	C70	Post-cholecystectomy	Post-op CD
0.909038901601830 7
3409	P55	C74	Post-cholecystectomy	Post-op CD
0.915141113653699 4
3410	P55	C78	Post-cholecystectomy	Post-op CD
0.751525553012967 2
3411	P58	C1	Post-cholecystectomy	Post-op CD

0.9645308924485125
3412 P58 C3 Post-cholecystectomy Post-op CD
0.9452707856598017
3413 P58 C7 Post-cholecystectomy Post-op CD
0.9223874904652937
3414 P58 C8 Post-cholecystectomy Post-op CD
0.9492753623188406
3415 P58 C11 Post-cholecystectomy Post-op CD
0.7507627765064836
3416 P58 C15 Post-cholecystectomy Post-op CD
0.734744469870328
3417 P58 C19 Post-cholecystectomy Post-op CD
0.7065217391304348
3418 P58 C22 Post-cholecystectomy Post-op CD
0.9466056445461479
3419 P58 C26 Post-cholecystectomy Post-op CD
0.9433638443935927
3420 P58 C28 Post-cholecystectomy Post-op CD
0.7602974828375286
3421 P58 C31 Post-cholecystectomy Post-op CD
0.7345537757437071
3422 P58 C35 Post-cholecystectomy Post-op CD
0.7620137299771167
3423 P58 C38 Post-cholecystectomy Post-op CD
0.9763539282990084
3424 P58 C40 Post-cholecystectomy Post-op CD
0.9021739130434783
3425 P58 C44 Post-cholecystectomy Post-op CD
0.7240655987795576
3426 P58 C47 Post-cholecystectomy Post-op CD
0.6937452326468345
3427 P58 C48 Post-cholecystectomy Post-op CD
0.9046529366895499
3428 P58 C49 Post-cholecystectomy Post-op CD
0.950419527078566
3429 P58 C53 Post-cholecystectomy Post-op CD
0.8087337909992373
3430 P58 C56 Post-cholecystectomy Post-op CD
0.7257818459191457
3431 P58 C60 Post-cholecystectomy Post-op CD
0.7130053394355453
3432 P58 C62 Post-cholecystectomy Post-op CD
0.8321891685736079
3433 P58 C64 Post-cholecystectomy Post-op CD
0.910373760488177
3434 P58 C65 Post-cholecystectomy Post-op CD
0.9380244088482075
3435 P58 C69 Post-cholecystectomy Post-op CD
0.8432494279176201
3436 P58 C70 Post-cholecystectomy Post-op CD
0.9344012204424104
3437 P58 C74 Post-cholecystectomy Post-op CD
0.940884820747521
3438 P58 C78 Post-cholecystectomy Post-op CD

0.8100686498855835
3439	P60	C1	Post-cholecystectomy	Post-op CD
0.9183829138062548
3440	P60	C3	Post-cholecystectomy	Post-op CD
0.9620518688024409
3441	P60	C7	Post-cholecystectomy	Post-op CD
0.9662471395881007
3442	P60	C8	Post-cholecystectomy	Post-op CD
0.9603356216628528
3443	P60	C11	Post-cholecystectomy	Post-op CD
0.9344012204424104
3444	P60	C15	Post-cholecystectomy	Post-op CD
0.9090389016018307
3445	P60	C19	Post-cholecystectomy	Post-op CD
0.9290617848970252
3446	P60	C22	Post-cholecystectomy	Post-op CD
0.9593821510297483
3447	P60	C26	Post-cholecystectomy	Post-op CD
0.8548817696414951
3448	P60	C28	Post-cholecystectomy	Post-op CD
0.9212433257055682
3449	P60	C31	Post-cholecystectomy	Post-op CD
0.8766209000762777
3450	P60	C35	Post-cholecystectomy	Post-op CD
0.9588100686498856
3451	P60	C38	Post-cholecystectomy	Post-op CD
0.9492753623188406
3452	P60	C40	Post-cholecystectomy	Post-op CD
0.6853546910755148
3453	P60	C44	Post-cholecystectomy	Post-op CD
0.9258199847444699
3454	P60	C47	Post-cholecystectomy	Post-op CD
0.873951182303585
3455	P60	C48	Post-cholecystectomy	Post-op CD
0.9651029748283753
3456	P60	C49	Post-cholecystectomy	Post-op CD
0.7726926010678871
3457	P60	C53	Post-cholecystectomy	Post-op CD
0.9672006102212052
3458	P60	C56	Post-cholecystectomy	Post-op CD
0.9002669717772692
3459	P60	C60	Post-cholecystectomy	Post-op CD
0.9216247139588101
3460	P60	C62	Post-cholecystectomy	Post-op CD
0.8884439359267735
3461	P60	C64	Post-cholecystectomy	Post-op CD
0.9328756674294432
3462	P60	C65	Post-cholecystectomy	Post-op CD
0.9692982456140351
3463	P60	C69	Post-cholecystectomy	Post-op CD
0.7675438596491229
3464	P60	C70	Post-cholecystectomy	Post-op CD
0.9492753623188406
3465	P60	C74	Post-cholecystectomy	Post-op CD

0.9769260106788711
3466   P60   C78   Post-cholecystectomy   Post-op CD
0.8714721586575134
3467   P63   C1   Post-cholecystectomy   Post-op CD
0.9700610221205187
3468   P63   C3   Post-cholecystectomy   Post-op CD
0.9260106788710908
3469   P63   C7   Post-cholecystectomy   Post-op CD
0.9498474446987033
3470   P63   C8   Post-cholecystectomy   Post-op CD
0.9506102212051869
3471   P63   C11   Post-cholecystectomy   Post-op CD
0.9344012204424104
3472   P63   C15   Post-cholecystectomy   Post-op CD
0.950228832951945
3473   P63   C19   Post-cholecystectomy   Post-op CD
0.8968344774980931
3474   P63   C22   Post-cholecystectomy   Post-op CD
0.9528985507246377
3475   P63   C26   Post-cholecystectomy   Post-op CD
0.9401220442410374
3476   P63   C28   Post-cholecystectomy   Post-op CD
0.9525171624713958
3477   P63   C31   Post-cholecystectomy   Post-op CD
0.9248665141113653
3478   P63   C35   Post-cholecystectomy   Post-op CD
0.9595728451563692
3479   P63   C38   Post-cholecystectomy   Post-op CD
0.9750190694126621
3480   P63   C40   Post-cholecystectomy   Post-op CD
0.9492753623188406
3481   P63   C44   Post-cholecystectomy   Post-op CD
0.8096872616323417
3482   P63   C47   Post-cholecystectomy   Post-op CD
0.9422196796338673
3483   P63   C48   Post-cholecystectomy   Post-op CD
0.9002669717772692
3484   P63   C49   Post-cholecystectomy   Post-op CD
0.9282990083905416
3485   P63   C53   Post-cholecystectomy   Post-op CD
0.9231502669717773
3486   P63   C56   Post-cholecystectomy   Post-op CD
0.8747139588100686
3487   P63   C60   Post-cholecystectomy   Post-op CD
0.8993135011441648
3488   P63   C62   Post-cholecystectomy   Post-op CD
0.8909229595728452
3489   P63   C64   Post-cholecystectomy   Post-op CD
0.8972158657513348
3490   P63   C65   Post-cholecystectomy   Post-op CD
0.9254385964912281
3491   P63   C69   Post-cholecystectomy   Post-op CD
0.8691838291380626
3492   P63   C70   Post-cholecystectomy   Post-op CD

0.9277269260106789
3493	P63	C74	Post-cholecystectomy	Post-op CD
0.9250572082379863
3494	P63	C78	Post-cholecystectomy	Post-op CD
0.5949656750572082
3495	P65	C1	Post-cholecystectomy	Post-op CD
0.9723493516399695
3496	P65	C3	Post-cholecystectomy	Post-op CD
0.9506102212051869
3497	P65	C7	Post-cholecystectomy	Post-op CD
0.8892067124332571
3498	P65	C8	Post-cholecystectomy	Post-op CD
0.9448893974065599
3499	P65	C11	Post-cholecystectomy	Post-op CD
0.8089244851258581
3500	P65	C15	Post-cholecystectomy	Post-op CD
0.8165522501906941
3501	P65	C19	Post-cholecystectomy	Post-op CD
0.8024408848207475
3502	P65	C22	Post-cholecystectomy	Post-op CD
0.965675057208238
3503	P65	C26	Post-cholecystectomy	Post-op CD
0.9324942791762014
3504	P65	C28	Post-cholecystectomy	Post-op CD
0.8081617086193745
3505	P65	C31	Post-cholecystectomy	Post-op CD
0.9355453852021358
3506	P65	C35	Post-cholecystectomy	Post-op CD
0.813119755911518
3507	P65	C38	Post-cholecystectomy	Post-op CD
0.969488939740656
3508	P65	C40	Post-cholecystectomy	Post-op CD
0.8653699466056446
3509	P65	C44	Post-cholecystectomy	Post-op CD
0.8123569794050344
3510	P65	C47	Post-cholecystectomy	Post-op CD
0.8651792524790236
3511	P65	C48	Post-cholecystectomy	Post-op CD
0.8585049580472921
3512	P65	C49	Post-cholecystectomy	Post-op CD
0.9220061022120518
3513	P65	C53	Post-cholecystectomy	Post-op CD
0.8102593440122045
3514	P65	C56	Post-cholecystectomy	Post-op CD
0.761441647597254
3515	P65	C60	Post-cholecystectomy	Post-op CD
0.8201754385964912
3516	P65	C62	Post-cholecystectomy	Post-op CD
0.8607932875667429
3517	P65	C64	Post-cholecystectomy	Post-op CD
0.8810068649885584
3518	P65	C65	Post-cholecystectomy	Post-op CD
0.9038901601830663
3519	P65	C69	Post-cholecystectomy	Post-op CD

0.8401983218916858
3520 P65 C70 Post-cholecystectomy Post-op CD
0.9040808543096872
3521 P65 C74 Post-cholecystectomy Post-op CD
0.9227688787185355
3522 P65 C78 Post-cholecystectomy Post-op CD
0.7170099160945843
3523 P68 C1 Post-cholecystectomy Post-op CD
0.9761632341723875
3524 P68 C3 Post-cholecystectomy Post-op CD
0.9628146453089245
3525 P68 C7 Post-cholecystectomy Post-op CD
0.9250572082379863
3526 P68 C8 Post-cholecystectomy Post-op CD
0.944698703279939
3527 P68 C11 Post-cholecystectomy Post-op CD
0.59744469870328
3528 P68 C15 Post-cholecystectomy Post-op CD
0.5596872616323417
3529 P68 C19 Post-cholecystectomy Post-op CD
0.7204424103737604
3530 P68 C22 Post-cholecystectomy Post-op CD
0.9269641495041953
3531 P68 C26 Post-cholecystectomy Post-op CD
0.8152173913043478
3532 P68 C28 Post-cholecystectomy Post-op CD
0.7608695652173914
3533 P68 C31 Post-cholecystectomy Post-op CD
0.8678489702517163
3534 P68 C35 Post-cholecystectomy Post-op CD
0.7166285278413425
3535 P68 C38 Post-cholecystectomy Post-op CD
0.9759725400457666
3536 P68 C40 Post-cholecystectomy Post-op CD
0.9048436308161708
3537 P68 C44 Post-cholecystectomy Post-op CD
0.614607170099161
3538 P68 C47 Post-cholecystectomy Post-op CD
0.643211289092296
3539 P68 C48 Post-cholecystectomy Post-op CD
0.8829138062547673
3540 P68 C49 Post-cholecystectomy Post-op CD
0.9185736079328757
3541 P68 C53 Post-cholecystectomy Post-op CD
0.7862318840579711
3542 P68 C56 Post-cholecystectomy Post-op CD
0.7067124332570557
3543 P68 C60 Post-cholecystectomy Post-op CD
0.7200610221205187
3544 P68 C62 Post-cholecystectomy Post-op CD
0.8270404271548436
3545 P68 C64 Post-cholecystectomy Post-op CD
0.8968344774980931
3546 P68 C65 Post-cholecystectomy Post-op CD

0.900839054157132
3547	P68	C69	Post-cholecystectomy	Post-op CD
0.8960717009916095
3548	P68	C70	Post-cholecystectomy	Post-op CD
0.919908466819222
3549	P68	C74	Post-cholecystectomy	Post-op CD
0.9355453852021358
3550	P68	C78	Post-cholecystectomy	Post-op CD
0.725209763539283
3551	P70	C1	Post-cholecystectomy	Post-op CD
0.9136155606407322
3552	P70	C3	Post-cholecystectomy	Post-op CD
0.9569031273836766
3553	P70	C7	Post-cholecystectomy	Post-op CD
0.876048817696415
3554	P70	C8	Post-cholecystectomy	Post-op CD
0.915903890160183
3555	P70	C11	Post-cholecystectomy	Post-op CD
0.6657131960335622
3556	P70	C15	Post-cholecystectomy	Post-op CD
0.6559877955758963
3557	P70	C19	Post-cholecystectomy	Post-op CD
0.6752479023646072
3558	P70	C22	Post-cholecystectomy	Post-op CD
0.9490846681922197
3559	P70	C26	Post-cholecystectomy	Post-op CD
0.8941647597254004
3560	P70	C28	Post-cholecystectomy	Post-op CD
0.7839435545385202
3561	P70	C31	Post-cholecystectomy	Post-op CD
0.9515636918382914
3562	P70	C35	Post-cholecystectomy	Post-op CD
0.704042715484363
3563	P70	C38	Post-cholecystectomy	Post-op CD
0.9759725400457666
3564	P70	C40	Post-cholecystectomy	Post-op CD
0.8712814645308925
3565	P70	C44	Post-cholecystectomy	Post-op CD
0.6857360793287567
3566	P70	C47	Post-cholecystectomy	Post-op CD
0.7524790236460717
3567	P70	C48	Post-cholecystectomy	Post-op CD
0.8607932875667429
3568	P70	C49	Post-cholecystectomy	Post-op CD
0.9126620900076278
3569	P70	C53	Post-cholecystectomy	Post-op CD
0.7892829900839055
3570	P70	C56	Post-cholecystectomy	Post-op CD
0.7313119755911518
3571	P70	C60	Post-cholecystectomy	Post-op CD
0.7105263157894737
3572	P70	C62	Post-cholecystectomy	Post-op CD
0.8895881006864989
3573	P70	C64	Post-cholecystectomy	Post-op CD

0.8962623951182304
3574	P70	C65	Post-cholecystectomy	Post-op CD
0.9078947368421053
3575	P70	C69	Post-cholecystectomy	Post-op CD
0.8749046529366895
3576	P70	C70	Post-cholecystectomy	Post-op CD
0.8729977116704806
3577	P70	C74	Post-cholecystectomy	Post-op CD
0.9078947368421053
3578	P70	C78	Post-cholecystectomy	Post-op CD
0.4832189168573608
3579	P71	C1	Post-cholecystectomy	Post-op CD
0.9527078565980168
3580	P71	C3	Post-cholecystectomy	Post-op CD
0.9601449275362319
3581	P71	C7	Post-cholecystectomy	Post-op CD
0.7475209763539283
3582	P71	C8	Post-cholecystectomy	Post-op CD
0.967581998474447
3583	P71	C11	Post-cholecystectomy	Post-op CD
0.830282227307399
3584	P71	C15	Post-cholecystectomy	Post-op CD
0.8913043478260869
3585	P71	C19	Post-cholecystectomy	Post-op CD
0.6706712433257056
3586	P71	C22	Post-cholecystectomy	Post-op CD
0.8716628527841342
3587	P71	C26	Post-cholecystectomy	Post-op CD
0.9242944317315027
3588	P71	C28	Post-cholecystectomy	Post-op CD
0.8899694889397407
3589	P71	C31	Post-cholecystectomy	Post-op CD
0.9538520213577422
3590	P71	C35	Post-cholecystectomy	Post-op CD
0.6292906178489702
3591	P71	C38	Post-cholecystectomy	Post-op CD
0.9744469870327994
3592	P71	C40	Post-cholecystectomy	Post-op CD
0.9466056445461479
3593	P71	C44	Post-cholecystectomy	Post-op CD
0.8344774980930587
3594	P71	C47	Post-cholecystectomy	Post-op CD
0.7263539282990084
3595	P71	C48	Post-cholecystectomy	Post-op CD
0.6790617848970252
3596	P71	C49	Post-cholecystectomy	Post-op CD
0.8068268497330282
3597	P71	C53	Post-cholecystectomy	Post-op CD
0.5932494279176201
3598	P71	C56	Post-cholecystectomy	Post-op CD
0.6916475972540046
3599	P71	C60	Post-cholecystectomy	Post-op CD
0.7536231884057971
3600	P71	C62	Post-cholecystectomy	Post-op CD

0.7719298245614035
3601	P71	C64	Post-cholecystectomy	Post-op CD
0.7452326468344775
3602	P71	C65	Post-cholecystectomy	Post-op CD
0.7307398932112891
3603	P71	C69	Post-cholecystectomy	Post-op CD
0.7070938215102975
3604	P71	C70	Post-cholecystectomy	Post-op CD
0.7168192219679634
3605	P71	C74	Post-cholecystectomy	Post-op CD
0.8033943554538521
3606	P71	C78	Post-cholecystectomy	Post-op CD
0.8321891685736079
3607	P74	C1	Post-cholecystectomy	Post-op CD
0.9262013729977117
3608	P74	C3	Post-cholecystectomy	Post-op CD
0.9300152555301296
3609	P74	C7	Post-cholecystectomy	Post-op CD
0.8686117467581999
3610	P74	C8	Post-cholecystectomy	Post-op CD
0.9282990083905416
3611	P74	C11	Post-cholecystectomy	Post-op CD
0.872234935163997
3612	P74	C15	Post-cholecystectomy	Post-op CD
0.8237986270022883
3613	P74	C19	Post-cholecystectomy	Post-op CD
0.8794813119755912
3614	P74	C22	Post-cholecystectomy	Post-op CD
0.8874904652936689
3615	P74	C26	Post-cholecystectomy	Post-op CD
0.8943554538520213
3616	P74	C28	Post-cholecystectomy	Post-op CD
0.7988176964149504
3617	P74	C31	Post-cholecystectomy	Post-op CD
0.9338291380625476
3618	P74	C35	Post-cholecystectomy	Post-op CD
0.834096109839817
3619	P74	C38	Post-cholecystectomy	Post-op CD
0.9801678108314263
3620	P74	C40	Post-cholecystectomy	Post-op CD
0.8888253241800153
3621	P74	C44	Post-cholecystectomy	Post-op CD
0.8823417238749046
3622	P74	C47	Post-cholecystectomy	Post-op CD
0.9258199847444699
3623	P74	C48	Post-cholecystectomy	Post-op CD
0.8615560640732265
3624	P74	C49	Post-cholecystectomy	Post-op CD
0.92372234935164
3625	P74	C53	Post-cholecystectomy	Post-op CD
0.8768115942028986
3626	P74	C56	Post-cholecystectomy	Post-op CD
0.8098779557589626
3627	P74	C60	Post-cholecystectomy	Post-op CD

0.8525934401220442
3628 P74 C62 Post-cholecystectomy Post-op CD
0.8792906178489702
3629 P74 C64 Post-cholecystectomy Post-op CD
0.8796720061022121
3630 P74 C65 Post-cholecystectomy Post-op CD
0.8960717009916095
3631 P74 C69 Post-cholecystectomy Post-op CD
0.8495423340961098
3632 P74 C70 Post-cholecystectomy Post-op CD
0.9149504195270786
3633 P74 C74 Post-cholecystectomy Post-op CD
0.9096109839816934
3634 P74 C78 Post-cholecystectomy Post-op CD
0.8201754385964912
3635 P75 C1 Post-cholecystectomy Post-op CD
0.9483218916857361
3636 P75 C3 Post-cholecystectomy Post-op CD
0.9263920671243325
3637 P75 C7 Post-cholecystectomy Post-op CD
0.8703279938977879
3638 P75 C8 Post-cholecystectomy Post-op CD
0.9576659038901602
3639 P75 C11 Post-cholecystectomy Post-op CD
0.9221967963386728
3640 P75 C15 Post-cholecystectomy Post-op CD
0.8790999237223494
3641 P75 C19 Post-cholecystectomy Post-op CD
0.8844393592677345
3642 P75 C22 Post-cholecystectomy Post-op CD
0.8533562166285278
3643 P75 C26 Post-cholecystectomy Post-op CD
0.8663234172387491
3644 P75 C28 Post-cholecystectomy Post-op CD
0.8781464530892449
3645 P75 C31 Post-cholecystectomy Post-op CD
0.9242944317315027
3646 P75 C35 Post-cholecystectomy Post-op CD
0.8829138062547673
3647 P75 C38 Post-cholecystectomy Post-op CD
0.9759725400457666
3648 P75 C40 Post-cholecystectomy Post-op CD
0.8625095347063311
3649 P75 C44 Post-cholecystectomy Post-op CD
0.9387871853546911
3650 P75 C47 Post-cholecystectomy Post-op CD
0.9344012204424104
3651 P75 C48 Post-cholecystectomy Post-op CD
0.8234172387490465
3652 P75 C49 Post-cholecystectomy Post-op CD
0.8783371472158658
3653 P75 C53 Post-cholecystectomy Post-op CD
0.8964530892448512
3654 P75 C56 Post-cholecystectomy Post-op CD

0.7776506483600305
3655	P75	C60	Post-cholecystectomy	Post-op CD
0.8442028985507246
3656	P75	C62	Post-cholecystectomy	Post-op CD
0.8428680396643783
3657	P75	C64	Post-cholecystectomy	Post-op CD
0.8506864988558352
3658	P75	C65	Post-cholecystectomy	Post-op CD
0.8560259344012204
3659	P75	C69	Post-cholecystectomy	Post-op CD
0.8192219679633868
3660	P75	C70	Post-cholecystectomy	Post-op CD
0.8220823798627003
3661	P75	C74	Post-cholecystectomy	Post-op CD
0.8920671243325705
3662	P75	C78	Post-cholecystectomy	Post-op CD
0.8003432494279176
0	C3	C1	Post-op CD	Post-op CD
0.8792906178489702
1	C7	C1	Post-op CD	Post-op CD
0.9738749046529367
2	C7	C3	Post-op CD	Post-op CD
0.9691075514874142
3	C8	C1	Post-op CD	Post-op CD
0.9492753623188406
4	C8	C3	Post-op CD	Post-op CD
0.7397025171624714
5	C8	C7	Post-op CD	Post-op CD
0.9767353165522502
6	C11	C1	Post-op CD	Post-op CD
0.9263920671243325
7	C11	C3	Post-op CD	Post-op CD
0.9807398932112891
8	C11	C7	Post-op CD	Post-op CD
0.8916857360793288
9	C11	C8	Post-op CD	Post-op CD
0.9643401983218917
10	C15	C1	Post-op CD	Post-op CD
0.9460335621662853
11	C15	C3	Post-op CD	Post-op CD
0.9710144927536232
12	C15	C7	Post-op CD	Post-op CD
0.9138062547673532
13	C15	C8	Post-op CD	Post-op CD
0.9691075514874142
14	C15	C11	Post-op CD	Post-op CD
0.4548054919908467
15	C19	C1	Post-op CD	Post-op CD
0.9820747520976354
16	C19	C3	Post-op CD	Post-op CD
0.9662471395881007
17	C19	C7	Post-op CD	Post-op CD
0.5259344012204424
18	C19	C8	Post-op CD	Post-op CD

0.9412662090007627
19 C19 C11 Post-op CD Post-op CD
0.6003051106025934
20 C19 C15 Post-op CD Post-op CD
0.7427536231884058
21 C22 C1 Post-op CD Post-op CD
0.8192219679633868
22 C22 C3 Post-op CD Post-op CD
0.8546910755148741
23 C22 C7 Post-op CD Post-op CD
0.975209763539283
24 C22 C8 Post-op CD Post-op CD
0.9347826086956522
25 C22 C11 Post-op CD Post-op CD
0.9668192219679634
26 C22 C15 Post-op CD Post-op CD
0.9416475972540046
27 C22 C19 Post-op CD Post-op CD
0.9681540808543097
28 C26 C1 Post-op CD Post-op CD
0.9487032799389779
29 C26 C3 Post-op CD Post-op CD
0.9761632341723875
30 C26 C7 Post-op CD Post-op CD
0.9647215865751335
31 C26 C8 Post-op CD Post-op CD
0.9672006102212052
32 C26 C11 Post-op CD Post-op CD
0.9284897025171625
33 C26 C15 Post-op CD Post-op CD
0.7810831426392068
34 C26 C19 Post-op CD Post-op CD
0.9437452326468345
35 C26 C22 Post-op CD Post-op CD
0.9582379862700229
36 C28 C1 Post-op CD Post-op CD
0.8543096872616324
37 C28 C3 Post-op CD Post-op CD
0.9704424103737604
38 C28 C7 Post-op CD Post-op CD
0.9124713958810069
39 C28 C8 Post-op CD Post-op CD
0.9582379862700229
40 C28 C11 Post-op CD Post-op CD
0.7456140350877193
41 C28 C15 Post-op CD Post-op CD
0.7602974828375286
42 C28 C19 Post-op CD Post-op CD
0.7572463768115942
43 C28 C22 Post-op CD Post-op CD
0.9557589626239512
44 C28 C26 Post-op CD Post-op CD
0.9042715484363082
45 C31 C1 Post-op CD Post-op CD

0.8487795575896262
46 C31 C3 Post-op CD Post-op CD
0.9824561403508771
47 C31 C7 Post-op CD Post-op CD
0.9820747520976354
48 C31 C8 Post-op CD Post-op CD
0.9668192219679634
49 C31 C11 Post-op CD Post-op CD
0.9452707856598017
50 C31 C15 Post-op CD Post-op CD
0.9403127383676583
51 C31 C19 Post-op CD Post-op CD
0.9710144927536232
52 C31 C22 Post-op CD Post-op CD
0.9406941266209001
53 C31 C26 Post-op CD Post-op CD
0.8592677345537757
54 C31 C28 Post-op CD Post-op CD
0.8350495804729214
55 C35 C1 Post-op CD Post-op CD
0.9700610221205187
56 C35 C3 Post-op CD Post-op CD
0.9757818459191457
57 C35 C7 Post-op CD Post-op CD
0.7191075514874142
58 C35 C8 Post-op CD Post-op CD
0.9750190694126621
59 C35 C11 Post-op CD Post-op CD
0.6590389016018307
60 C35 C15 Post-op CD Post-op CD
0.7534324942791762
61 C35 C19 Post-op CD Post-op CD
0.6064073226544623
62 C35 C22 Post-op CD Post-op CD
0.9700610221205187
63 C35 C26 Post-op CD Post-op CD
0.9235316552250191
64 C35 C28 Post-op CD Post-op CD
0.652745995423341
65 C35 C31 Post-op CD Post-op CD
0.9416475972540046
66 C38 C1 Post-op CD Post-op CD
0.936880244088482
67 C38 C3 Post-op CD Post-op CD
0.9437452326468345
68 C38 C7 Post-op CD Post-op CD
0.988558352402746
69 C38 C8 Post-op CD Post-op CD
0.967581998474447
70 C38 C11 Post-op CD Post-op CD
0.9660564454614798
71 C38 C15 Post-op CD Post-op CD
0.9233409610983981
72 C38 C19 Post-op CD Post-op CD

0.9841723874904653
73 C38 C22 Post-op CD Post-op CD
0.9328756674294432
74 C38 C26 Post-op CD Post-op CD
0.9397406559877955
75 C38 C28 Post-op CD Post-op CD
0.9605263157894737
76 C38 C31 Post-op CD Post-op CD
0.8888253241800153
77 C38 C35 Post-op CD Post-op CD
0.988558352402746
78 C40 C1 Post-op CD Post-op CD
0.9122807017543859
79 C40 C3 Post-op CD Post-op CD
0.9685354691075515
80 C40 C7 Post-op CD Post-op CD
0.9609077040427155
81 C40 C8 Post-op CD Post-op CD
0.9178108314263921
82 C40 C11 Post-op CD Post-op CD
0.9405034324942791
83 C40 C15 Post-op CD Post-op CD
0.8943554538520213
84 C40 C19 Post-op CD Post-op CD
0.9496567505720824
85 C40 C22 Post-op CD Post-op CD
0.9578565980167811
86 C40 C26 Post-op CD Post-op CD
0.793859649122807
87 C40 C28 Post-op CD Post-op CD
0.9059877955758963
88 C40 C31 Post-op CD Post-op CD
0.870137299771167
89 C40 C35 Post-op CD Post-op CD
0.9530892448512586
90 C40 C38 Post-op CD Post-op CD
0.9214340198321892
91 C44 C1 Post-op CD Post-op CD
0.9305873379099924
92 C44 C3 Post-op CD Post-op CD
0.9776887871853547
93 C44 C7 Post-op CD Post-op CD
0.9067505720823799
94 C44 C8 Post-op CD Post-op CD
0.9626239511823036
95 C44 C11 Post-op CD Post-op CD
0.2885202135774218
96 C44 C15 Post-op CD Post-op CD
0.4712051868802441
97 C44 C19 Post-op CD Post-op CD
0.6064073226544623
98 C44 C22 Post-op CD Post-op CD
0.9708237986270023
99 C44 C26 Post-op CD Post-op CD

0.9710144927536232
100	C44	C28	Post-op CD	Post-op CD
0.782418001525553
101	C44	C31	Post-op CD	Post-op CD
0.9683447749809306
102	C44	C35	Post-op CD	Post-op CD
0.681350114416476
103	C44	C38	Post-op CD	Post-op CD
0.9807398932112891
104	C44	C40	Post-op CD	Post-op CD
0.9576659038901602
105	C47	C1	Post-op CD	Post-op CD
0.984744469870328
106	C47	C3	Post-op CD	Post-op CD
0.9755911517925248
107	C47	C7	Post-op CD	Post-op CD
0.9267734553775744
108	C47	C8	Post-op CD	Post-op CD
0.9853165522501907
109	C47	C11	Post-op CD	Post-op CD
0.545766590389016
110	C47	C15	Post-op CD	Post-op CD
0.6308161708619374
111	C47	C19	Post-op CD	Post-op CD
0.6500762776506483
112	C47	C22	Post-op CD	Post-op CD
0.9719679633867276
113	C47	C26	Post-op CD	Post-op CD
0.9708237986270023
114	C47	C28	Post-op CD	Post-op CD
0.7963386727688787
115	C47	C31	Post-op CD	Post-op CD
0.984744469870328
116	C47	C35	Post-op CD	Post-op CD
0.5829519450800915
117	C47	C38	Post-op CD	Post-op CD
0.9836003051106026
118	C47	C40	Post-op CD	Post-op CD
0.9853165522501907
119	C47	C44	Post-op CD	Post-op CD
0.5289855072463768
120	C48	C1	Post-op CD	Post-op CD
0.956140350877193
121	C48	C3	Post-op CD	Post-op CD
0.9311594202898551
122	C48	C7	Post-op CD	Post-op CD
0.6550343249427918
123	C48	C8	Post-op CD	Post-op CD
0.9553775743707094
124	C48	C11	Post-op CD	Post-op CD
0.92372234935164
125	C48	C15	Post-op CD	Post-op CD
0.9231502669717773
126	C48	C19	Post-op CD	Post-op CD

0.7185354691075515
127 C48 C22 Post-op CD Post-op CD
0.9410755148741419
128 C48 C26 Post-op CD Post-op CD
0.9145690312738368
129 C48 C28 Post-op CD Post-op CD
0.9035087719298246
130 C48 C31 Post-op CD Post-op CD
0.9654843630816171
131 C48 C35 Post-op CD Post-op CD
0.7410373760488177
132 C48 C38 Post-op CD Post-op CD
0.9744469870327994
133 C48 C40 Post-op CD Post-op CD
0.954042715484363
134 C48 C44 Post-op CD Post-op CD
0.9223874904652937
135 C48 C47 Post-op CD Post-op CD
0.8813882532418001
136 C49 C1 Post-op CD Post-op CD
0.9557589626239512
137 C49 C3 Post-op CD Post-op CD
0.9294431731502669
138 C49 C7 Post-op CD Post-op CD
0.8756674294431731
139 C49 C8 Post-op CD Post-op CD
0.9538520213577422
140 C49 C11 Post-op CD Post-op CD
0.9704424103737604
141 C49 C15 Post-op CD Post-op CD
0.9202898550724637
142 C49 C19 Post-op CD Post-op CD
0.8743325705568269
143 C49 C22 Post-op CD Post-op CD
0.9290617848970252
144 C49 C26 Post-op CD Post-op CD
0.8676582761250954
145 C49 C28 Post-op CD Post-op CD
0.9416475972540046
146 C49 C31 Post-op CD Post-op CD
0.9605263157894737
147 C49 C35 Post-op CD Post-op CD
0.868230358504958
148 C49 C38 Post-op CD Post-op CD
0.9696796338672768
149 C49 C40 Post-op CD Post-op CD
0.740465293668955
150 C49 C44 Post-op CD Post-op CD
0.9643401983218917
151 C49 C47 Post-op CD Post-op CD
0.9214340198321892
152 C49 C48 Post-op CD Post-op CD
0.5240274599542334
153 C53 C1 Post-op CD Post-op CD

0.9778794813119756
154	C53	C3	Post-op CD	Post-op CD
0.9483218916857361
155	C53	C7	Post-op CD	Post-op CD
0.8106407322654462
156	C53	C8	Post-op CD	Post-op CD
0.9652936689549961
157	C53	C11	Post-op CD	Post-op CD
0.7116704805491991
158	C53	C15	Post-op CD	Post-op CD
0.8234172387490465
159	C53	C19	Post-op CD	Post-op CD
0.6014492753623188
160	C53	C22	Post-op CD	Post-op CD
0.9536613272311213
161	C53	C26	Post-op CD	Post-op CD
0.9445080091533181
162	C53	C28	Post-op CD	Post-op CD
0.7578184591914569
163	C53	C31	Post-op CD	Post-op CD
0.9538520213577422
164	C53	C35	Post-op CD	Post-op CD
0.5333714721586575
165	C53	C38	Post-op CD	Post-op CD
0.9799771167048055
166	C53	C40	Post-op CD	Post-op CD
0.9546147978642258
167	C53	C44	Post-op CD	Post-op CD
0.7444698703279939
168	C53	C47	Post-op CD	Post-op CD
0.5749427917620137
169	C53	C48	Post-op CD	Post-op CD
0.40122044241037375
170	C53	C49	Post-op CD	Post-op CD
0.5684591914569032
171	C56	C1	Post-op CD	Post-op CD
0.9410755148741419
172	C56	C3	Post-op CD	Post-op CD
0.9269641495041953
173	C56	C7	Post-op CD	Post-op CD
0.778794813119756
174	C56	C8	Post-op CD	Post-op CD
0.950228832951945
175	C56	C11	Post-op CD	Post-op CD
0.7972921434019832
176	C56	C15	Post-op CD	Post-op CD
0.7625858123569794
177	C56	C19	Post-op CD	Post-op CD
0.6847826086956522
178	C56	C22	Post-op CD	Post-op CD
0.9391685736079328
179	C56	C26	Post-op CD	Post-op CD
0.9176201372997712
180	C56	C28	Post-op CD	Post-op CD

0.7726926010678871
181	C56	C31	Post-op CD	Post-op CD
0.8975972540045767
182	C56	C35	Post-op CD	Post-op CD
0.7274980930587338
183	C56	C38	Post-op CD	Post-op CD
0.9706331045003814
184	C56	C40	Post-op CD	Post-op CD
0.8735697940503433
185	C56	C44	Post-op CD	Post-op CD
0.7955758962623951
186	C56	C47	Post-op CD	Post-op CD
0.7810831426392068
187	C56	C48	Post-op CD	Post-op CD
0.324370709382151
188	C56	C49	Post-op CD	Post-op CD
0.5177345537757437
189	C56	C53	Post-op CD	Post-op CD
0.31369183829138064
190	C60	C1	Post-op CD	Post-op CD
0.8377192982456141
191	C60	C3	Post-op CD	Post-op CD
0.8211289092295957
192	C60	C7	Post-op CD	Post-op CD
0.831998474446987
193	C60	C8	Post-op CD	Post-op CD
0.8575514874141876
194	C60	C11	Post-op CD	Post-op CD
0.7568649885583524
195	C60	C15	Post-op CD	Post-op CD
0.7416094584286804
196	C60	C19	Post-op CD	Post-op CD
0.6578947368421053
197	C60	C22	Post-op CD	Post-op CD
0.8327612509534706
198	C60	C26	Post-op CD	Post-op CD
0.9590007627765065
199	C60	C28	Post-op CD	Post-op CD
0.7969107551487414
200	C60	C31	Post-op CD	Post-op CD
0.954233409610984
201	C60	C35	Post-op CD	Post-op CD
0.702326468344775
202	C60	C38	Post-op CD	Post-op CD
0.9239130434782609
203	C60	C40	Post-op CD	Post-op CD
0.9334477498093059
204	C60	C44	Post-op CD	Post-op CD
0.7669717772692601
205	C60	C47	Post-op CD	Post-op CD
0.7181540808543097
206	C60	C48	Post-op CD	Post-op CD
0.5594965675057209
207	C60	C49	Post-op CD	Post-op CD

0.6130816170861938
208	C60	C53	Post-op CD	Post-op CD
0.48264683447749807
209	C60	C56	Post-op CD	Post-op CD
0.448512585812357
210	C62	C1	Post-op CD	Post-op CD
0.9628146453089245
211	C62	C3	Post-op CD	Post-op CD
0.9364988558352403
212	C62	C7	Post-op CD	Post-op CD
0.8438215102974829
213	C62	C8	Post-op CD	Post-op CD
0.9492753623188406
214	C62	C11	Post-op CD	Post-op CD
0.8729977116704806
215	C62	C15	Post-op CD	Post-op CD
0.9084668192219679
216	C62	C19	Post-op CD	Post-op CD
0.8419145690312738
217	C62	C22	Post-op CD	Post-op CD
0.9374523264683448
218	C62	C26	Post-op CD	Post-op CD
0.868230358504958
219	C62	C28	Post-op CD	Post-op CD
0.9115179252479023
220	C62	C31	Post-op CD	Post-op CD
0.736651411136537
221	C62	C35	Post-op CD	Post-op CD
0.8695652173913043
222	C62	C38	Post-op CD	Post-op CD
0.9078947368421053
223	C62	C40	Post-op CD	Post-op CD
0.8745232646834478
224	C62	C44	Post-op CD	Post-op CD
0.9439359267734554
225	C62	C47	Post-op CD	Post-op CD
0.9094202898550725
226	C62	C48	Post-op CD	Post-op CD
0.5101067887109078
227	C62	C49	Post-op CD	Post-op CD
0.5701754385964912
228	C62	C53	Post-op CD	Post-op CD
0.5432875667429443
229	C62	C56	Post-op CD	Post-op CD
0.46281464530892447
230	C62	C60	Post-op CD	Post-op CD
0.5835240274599542
231	C64	C1	Post-op CD	Post-op CD
0.9490846681922197
232	C64	C3	Post-op CD	Post-op CD
0.9075133485888635
233	C64	C7	Post-op CD	Post-op CD
0.8360030511060259
234	C64	C8	Post-op CD	Post-op CD

0.9385964912280702
235	C64	C11	Post-op CD	Post-op CD
0.8852021357742181
236	C64	C15	Post-op CD	Post-op CD
0.9109458428680397
237	C64	C19	Post-op CD	Post-op CD
0.8295194508009154
238	C64	C22	Post-op CD	Post-op CD
0.9040808543096872
239	C64	C26	Post-op CD	Post-op CD
0.8749046529366895
240	C64	C28	Post-op CD	Post-op CD
0.9084668192219679
241	C64	C31	Post-op CD	Post-op CD
0.8482074752097636
242	C64	C35	Post-op CD	Post-op CD
0.8480167810831426
243	C64	C38	Post-op CD	Post-op CD
0.8800533943554538
244	C64	C40	Post-op CD	Post-op CD
0.9052250190694127
245	C64	C44	Post-op CD	Post-op CD
0.9395499618611747
246	C64	C47	Post-op CD	Post-op CD
0.897025171624714
247	C64	C48	Post-op CD	Post-op CD
0.41380625476735317
248	C64	C49	Post-op CD	Post-op CD
0.5581617086193745
249	C64	C53	Post-op CD	Post-op CD
0.4569031273836766
250	C64	C56	Post-op CD	Post-op CD
0.42200610221205187
251	C64	C60	Post-op CD	Post-op CD
0.5486270022883295
252	C64	C62	Post-op CD	Post-op CD
0.36384439359267734
253	C65	C1	Post-op CD	Post-op CD
0.8184591914569032
254	C65	C3	Post-op CD	Post-op CD
0.855072463768116
255	C65	C7	Post-op CD	Post-op CD
0.8281845919145691
256	C65	C8	Post-op CD	Post-op CD
0.9363081617086194
257	C65	C11	Post-op CD	Post-op CD
0.9551868802440885
258	C65	C15	Post-op CD	Post-op CD
0.9235316552250191
259	C65	C19	Post-op CD	Post-op CD
0.8287566742944318
260	C65	C22	Post-op CD	Post-op CD
0.7551487414187643
261	C65	C26	Post-op CD	Post-op CD

0.9443173150266971
262	C65	C28	Post-op CD	Post-op CD
0.9494660564454614
263	C65	C31	Post-op CD	Post-op CD
0.9166666666666666
264	C65	C35	Post-op CD	Post-op CD
0.8836765827612509
265	C65	C38	Post-op CD	Post-op CD
0.8909229595728452
266	C65	C40	Post-op CD	Post-op CD
0.9403127383676583
267	C65	C44	Post-op CD	Post-op CD
0.9475591151792525
268	C65	C47	Post-op CD	Post-op CD
0.9145690312738368
269	C65	C48	Post-op CD	Post-op CD
0.37623951182303583
270	C65	C49	Post-op CD	Post-op CD
0.5465293668954996
271	C65	C53	Post-op CD	Post-op CD
0.4666285278413425
272	C65	C56	Post-op CD	Post-op CD
0.39740655987795576
273	C65	C60	Post-op CD	Post-op CD
0.4759725400457666
274	C65	C62	Post-op CD	Post-op CD
0.4738749046529367
275	C65	C64	Post-op CD	Post-op CD
0.39282990083905417
276	C69	C1	Post-op CD	Post-op CD
0.9488939740655988
277	C69	C3	Post-op CD	Post-op CD
0.9488939740655988
278	C69	C7	Post-op CD	Post-op CD
0.784324942791762
279	C69	C8	Post-op CD	Post-op CD
0.9538520213577422
280	C69	C11	Post-op CD	Post-op CD
0.8913043478260869
281	C69	C15	Post-op CD	Post-op CD
0.8726163234172387
282	C69	C19	Post-op CD	Post-op CD
0.7780320366132724
283	C69	C22	Post-op CD	Post-op CD
0.9500381388253242
284	C69	C26	Post-op CD	Post-op CD
0.8133104500381388
285	C69	C28	Post-op CD	Post-op CD
0.8724256292906178
286	C69	C31	Post-op CD	Post-op CD
0.8924485125858124
287	C69	C35	Post-op CD	Post-op CD
0.8377192982456141
288	C69	C38	Post-op CD	Post-op CD

0.9473684210526315
289	C69	C40	Post-op CD	Post-op CD
0.8106407322654462
290	C69	C44	Post-op CD	Post-op CD
0.8789092295957285
291	C69	C47	Post-op CD	Post-op CD
0.8438215102974829
292	C69	C48	Post-op CD	Post-op CD
0.41914569031273835
293	C69	C49	Post-op CD	Post-op CD
0.4900839054157132
294	C69	C53	Post-op CD	Post-op CD
0.4754004576659039
295	C69	C56	Post-op CD	Post-op CD
0.4162852784134249
296	C69	C60	Post-op CD	Post-op CD
0.5964912280701754
297	C69	C62	Post-op CD	Post-op CD
0.5026697177726926
298	C69	C64	Post-op CD	Post-op CD
0.43592677345537756
299	C69	C65	Post-op CD	Post-op CD
0.444698703279939
300	C70	C1	Post-op CD	Post-op CD
0.9651029748283753
301	C70	C3	Post-op CD	Post-op CD
0.9506102212051869
302	C70	C7	Post-op CD	Post-op CD
0.835812356979405
303	C70	C8	Post-op CD	Post-op CD
0.9414569031273837
304	C70	C11	Post-op CD	Post-op CD
0.8802440884820748
305	C70	C15	Post-op CD	Post-op CD
0.9105644546147978
306	C70	C19	Post-op CD	Post-op CD
0.8047292143401983
307	C70	C22	Post-op CD	Post-op CD
0.9101830663615561
308	C70	C26	Post-op CD	Post-op CD
0.8975972540045767
309	C70	C28	Post-op CD	Post-op CD
0.9374523264683448
310	C70	C31	Post-op CD	Post-op CD
0.9393592677345538
311	C70	C35	Post-op CD	Post-op CD
0.8811975591151793
312	C70	C38	Post-op CD	Post-op CD
0.9519450800915332
313	C70	C40	Post-op CD	Post-op CD
0.8398169336384439
314	C70	C44	Post-op CD	Post-op CD
0.9578565980167811
315	C70	C47	Post-op CD	Post-op CD

0.9035087719298246
316 C70 C48 Post-op CD Post-op CD
0.41990846681922195
317 C70 C49 Post-op CD Post-op CD
0.5442410373760488
318 C70 C53 Post-op CD Post-op CD
0.4666285278413425
319 C70 C56 Post-op CD Post-op CD
0.42200610221205187
320 C70 C60 Post-op CD Post-op CD
0.5850495804729214
321 C70 C62 Post-op CD Post-op CD
0.4645308924485126
322 C70 C64 Post-op CD Post-op CD
0.40389016018306634
323 C70 C65 Post-op CD Post-op CD
0.41418764302059496
324 C70 C69 Post-op CD Post-op CD
0.4405034324942792
325 C74 C1 Post-op CD Post-op CD
0.8327612509534706
326 C74 C3 Post-op CD Post-op CD
0.8518306636155606
327 C74 C7 Post-op CD Post-op CD
0.8752860411899314
328 C74 C8 Post-op CD Post-op CD
0.9525171624713958
329 C74 C11 Post-op CD Post-op CD
0.9710144927536232
330 C74 C15 Post-op CD Post-op CD
0.9563310450038138
331 C74 C19 Post-op CD Post-op CD
0.8691838291380626
332 C74 C22 Post-op CD Post-op CD
0.8140732265446224
333 C74 C26 Post-op CD Post-op CD
0.9759725400457666
334 C74 C28 Post-op CD Post-op CD
0.959954233409611
335 C74 C31 Post-op CD Post-op CD
0.9856979405034325
336 C74 C35 Post-op CD Post-op CD
0.8880625476735317
337 C74 C38 Post-op CD Post-op CD
0.9759725400457666
338 C74 C40 Post-op CD Post-op CD
0.9769260106788711
339 C74 C44 Post-op CD Post-op CD
0.9660564454614798
340 C74 C47 Post-op CD Post-op CD
0.9181922196796338
341 C74 C48 Post-op CD Post-op CD
0.5779938977879482
342 C74 C49 Post-op CD Post-op CD

0.6197559115179252
343 C74 C53 Post-op CD Post-op CD
0.6203279938977879
344 C74 C56 Post-op CD Post-op CD
0.5814263920671243
345 C74 C60 Post-op CD Post-op CD
0.5436689549961862
346 C74 C62 Post-op CD Post-op CD
0.6220442410373761
347 C74 C64 Post-op CD Post-op CD
0.5980167810831426
348 C74 C65 Post-op CD Post-op CD
0.4551868802440885
349 C74 C69 Post-op CD Post-op CD
0.6228070175438597
350 C74 C70 Post-op CD Post-op CD
0.6060259344012204
351 C78 C1 Post-op CD Post-op CD
0.9334477498093059
352 C78 C3 Post-op CD Post-op CD
0.9670099160945843
353 C78 C7 Post-op CD Post-op CD
0.8848207475209764
354 C78 C8 Post-op CD Post-op CD
0.9670099160945843
355 C78 C11 Post-op CD Post-op CD
0.8022501906941266
356 C78 C15 Post-op CD Post-op CD
0.7494279176201373
357 C78 C19 Post-op CD Post-op CD
0.8348588863463006
358 C78 C22 Post-op CD Post-op CD
0.9609077040427155
359 C78 C26 Post-op CD Post-op CD
0.8133104500381388
360 C78 C28 Post-op CD Post-op CD
0.8483981693363845
361 C78 C31 Post-op CD Post-op CD
0.9118993135011442
362 C78 C35 Post-op CD Post-op CD
0.8258962623951183
363 C78 C38 Post-op CD Post-op CD
0.9475591151792525
364 C78 C40 Post-op CD Post-op CD
0.8632723112128147
365 C78 C44 Post-op CD Post-op CD
0.851258581235698
366 C78 C47 Post-op CD Post-op CD
0.8623188405797102
367 C78 C48 Post-op CD Post-op CD
0.8630816170861938
368 C78 C49 Post-op CD Post-op CD
0.8569794050343249
369 C78 C53 Post-op CD Post-op CD

0.8499237223493517
370	C78	C56	Post-op CD	Post-op CD
0.7969107551487414
371	C78	C60	Post-op CD	Post-op CD
0.8415331807780321
372	C78	C62	Post-op CD	Post-op CD
0.8607932875667429
373	C78	C64	Post-op CD	Post-op CD
0.893211289092296
374	C78	C65	Post-op CD	Post-op CD
0.9220061022120518
375	C78	C69	Post-op CD	Post-op CD
0.7892829900839055
376	C78	C70	Post-op CD	Post-op CD
0.8895881006864989
377	C78	C74	Post-op CD	Post-op CD
0.918001525553013
378	C1	B27	Post-op CD	Control group
0.9645308924485125
379	C1	B66	Post-op CD	Control group
0.9412662090007627
380	C1	B86	Post-op CD	Control group
0.9563310450038138
381	C1	B97	Post-op CD	Control group
0.9738749046529367
382	C1	B98	Post-op CD	Control group
0.9422196796338673
383	C1	B100	Post-op CD	Control group
0.9355453852021358
384	C1	B112	Post-op CD	Control group
0.9593821510297483
385	C1	B115	Post-op CD	Control group
0.9706331045003814
386	C1	I1	Post-op CD	Control group
0.9610983981693364
387	C1	I3	Post-op CD	Control group
0.8827231121281465
388	C1	I6	Post-op CD	Control group
0.9696796338672768
389	C1	I8	Post-op CD	Control group
0.9685354691075515
390	C1	I10	Post-op CD	Control group
0.9559496567505721
391	C1	I11	Post-op CD	Control group
0.9450800915331807
392	C1	I13	Post-op CD	Control group
0.9670099160945843
393	C1	I15	Post-op CD	Control group
0.8571700991609459
394	C1	I17	Post-op CD	Control group
0.9622425629290617
395	C1	I18	Post-op CD	Control group
0.9042715484363082
396	C1	I19	Post-op CD	Control group

0.9895118230358505
397	C1	I22	Post-op CD	Control group
0.8442028985507246
398	C1	I23	Post-op CD	Control group
0.9588100686498856
399	C1	I24	Post-op CD	Control group
0.9931350114416476
400	C1	I25	Post-op CD	Control group
0.952326468344775
401	C1	I26	Post-op CD	Control group
0.9387871853546911
402	C1	I27	Post-op CD	Control group
0.9099923722349351
403	C1	I28	Post-op CD	Control group
0.8381006864988558
404	C1	I29	Post-op CD	Control group
0.9570938215102975
405	C1	I30	Post-op CD	Control group
0.9218154080854309
406	C1	I31	Post-op CD	Control group
0.9633867276887872
407	C1	I32	Post-op CD	Control group
0.9691075514874142
408	C1	I33	Post-op CD	Control group
0.9744469870327994
409	C1	I34	Post-op CD	Control group
0.9605263157894737
410	C1	I35	Post-op CD	Control group
0.9565217391304348
411	C1	I36	Post-op CD	Control group
0.8196033562166285
412	C1	I37	Post-op CD	Control group
0.8154080854309688
413	C3	B27	Post-op CD	Control group
0.8968344774980931
414	C3	B66	Post-op CD	Control group
0.9412662090007627
415	C3	B86	Post-op CD	Control group
0.9345919145690312
416	C3	B97	Post-op CD	Control group
0.8920671243325705
417	C3	B98	Post-op CD	Control group
0.8363844393592678
418	C3	B100	Post-op CD	Control group
0.9176201372997712
419	C3	B112	Post-op CD	Control group
0.9427917620137299
420	C3	B115	Post-op CD	Control group
0.9361174675819984
421	C3	I1	Post-op CD	Control group
0.894355453852013
422	C3	I3	Post-op CD	Control group
0.940884820747521
423	C3	I6	Post-op CD	Control group

0.9057971014492754
424 C3 I8 Post-op CD Control group
0.9071319603356217
425 C3 I10 Post-op CD Control group
0.9185736079328757
426 C3 I11 Post-op CD Control group
0.8920671243325705
427 C3 I13 Post-op CD Control group
0.9094202898550725
428 C3 I15 Post-op CD Control group
0.8663234172387491
429 C3 I17 Post-op CD Control group
0.9469870327993898
430 C3 I18 Post-op CD Control group
0.8905415713196033
431 C3 I19 Post-op CD Control group
0.9406941266209001
432 C3 I22 Post-op CD Control group
0.8220823798627003
433 C3 I23 Post-op CD Control group
0.8924485125858124
434 C3 I24 Post-op CD Control group
0.9691075514874142
435 C3 I25 Post-op CD Control group
0.9422196796338673
436 C3 I26 Post-op CD Control group
0.9471777269260107
437 C3 I27 Post-op CD Control group
0.9300152555301296
438 C3 I28 Post-op CD Control group
0.8951182303585049
439 C3 I29 Post-op CD Control group
0.952326468344775
440 C3 I30 Post-op CD Control group
0.9576659038901602
441 C3 I31 Post-op CD Control group
0.9544241037376049
442 C3 I32 Post-op CD Control group
0.9725400457665904
443 C3 I33 Post-op CD Control group
0.9464149504195271
444 C3 I34 Post-op CD Control group
0.9294431731502669
445 C3 I35 Post-op CD Control group
0.8985507246376812
446 C3 I36 Post-op CD Control group
0.8995041952707856
447 C3 I37 Post-op CD Control group
0.631769641495042
448 C7 B27 Post-op CD Control group
0.715675057208238
449 C7 B66 Post-op CD Control group
0.8861556064073226
450 C7 B86 Post-op CD Control group

0.6561784897025171
451	C7	B97	Post-op CD	Control group
0.9145690312738368
452	C7	B98	Post-op CD	Control group
0.9183829138062548
453	C7	B100	Post-op CD	Control group
0.9856979405034325
454	C7	B112	Post-op CD	Control group
0.7299771167048055
455	C7	B115	Post-op CD	Control group
0.9288710907704043
456	C7	I1	Post-op CD	Control group
0.9080854309687262
457	C7	I3	Post-op CD	Control group
0.9290617848970252
458	C7	I6	Post-op CD	Control group
0.9403127383676583
459	C7	I8	Post-op CD	Control group
0.9241037376048817
460	C7	I10	Post-op CD	Control group
0.910373760488177
461	C7	I11	Post-op CD	Control group
0.9864607170099161
462	C7	I13	Post-op CD	Control group
0.8962623951182304
463	C7	I15	Post-op CD	Control group
0.9242944317315027
464	C7	I17	Post-op CD	Control group
0.7377955758962624
465	C7	I18	Post-op CD	Control group
0.8972158657513348
466	C7	I19	Post-op CD	Control group
0.7692601067887109
467	C7	I22	Post-op CD	Control group
0.9334477498093059
468	C7	I23	Post-op CD	Control group
0.8995041952707856
469	C7	I24	Post-op CD	Control group
0.5108695652173914
470	C7	I25	Post-op CD	Control group
0.9044622425629291
471	C7	I26	Post-op CD	Control group
0.8617467581998475
472	C7	I27	Post-op CD	Control group
0.8972158657513348
473	C7	I28	Post-op CD	Control group
0.9570938215102975
474	C7	I29	Post-op CD	Control group
0.9406941266209001
475	C7	I30	Post-op CD	Control group
0.8920671243325705
476	C7	I31	Post-op CD	Control group
0.9740655987795576
477	C7	I32	Post-op CD	Control group

0.9431731502669718
478	C7	I33	Post-op CD	Control group
0.5396643783371472
479	C7	I34	Post-op CD	Control group
0.7402745995423341
480	C7	I35	Post-op CD	Control group
0.9437452326468345
481	C7	I36	Post-op CD	Control group
0.9971395881006865
482	C7	I37	Post-op CD	Control group
0.9639588100686499
483	C8	B27	Post-op CD	Control group
0.9189549961861174
484	C8	B66	Post-op CD	Control group
0.92372234935164
485	C8	B86	Post-op CD	Control group
0.9416475972540046
486	C8	B97	Post-op CD	Control group
0.900839054157132
487	C8	B98	Post-op CD	Control group
0.8861556064073226
488	C8	B100	Post-op CD	Control group
0.9530892448512586
489	C8	B112	Post-op CD	Control group
0.938977879481312
490	C8	B115	Post-op CD	Control group
0.9654843630816171
491	C8	I1	Post-op CD	Control group
0.9109458428680397
492	C8	I3	Post-op CD	Control group
0.9830282227307399
493	C8	I6	Post-op CD	Control group
0.9162852784134249
494	C8	I8	Post-op CD	Control group
0.9206712433257056
495	C8	I10	Post-op CD	Control group
0.954042715484363
496	C8	I11	Post-op CD	Control group
0.9155225019069413
497	C8	I13	Post-op CD	Control group
0.9221967963386728
498	C8	I15	Post-op CD	Control group
0.933066361556064
499	C8	I17	Post-op CD	Control group
0.9715865751334859
500	C8	I18	Post-op CD	Control group
0.9406941266209001
501	C8	I19	Post-op CD	Control group
0.9719679633867276
502	C8	I22	Post-op CD	Control group
0.940884820747521
503	C8	I23	Post-op CD	Control group
0.933257055682685
504	C8	I24	Post-op CD	Control group

0.9689168573607932
505	C8	I25	Post-op CD	Control group
0.9534706331045004
506	C8	I26	Post-op CD	Control group
0.9294431731502669
507	C8	I27	Post-op CD	Control group
0.9412662090007627
508	C8	I28	Post-op CD	Control group
0.8954996186117468
509	C8	I29	Post-op CD	Control group
0.9494660564454614
510	C8	I30	Post-op CD	Control group
0.9378337147215866
511	C8	I31	Post-op CD	Control group
0.9658657513348589
512	C8	I32	Post-op CD	Control group
0.9681540808543097
513	C8	I33	Post-op CD	Control group
0.9679633867276888
514	C8	I34	Post-op CD	Control group
0.9441266209000763
515	C8	I35	Post-op CD	Control group
0.8800533943554538
516	C8	I36	Post-op CD	Control group
0.9525171624713958
517	C8	I37	Post-op CD	Control group
0.8411517925247902
518	C11	B27	Post-op CD	Control group
0.9326849733028223
519	C11	B66	Post-op CD	Control group
0.7972921434019832
520	C11	B86	Post-op CD	Control group
0.8813882532418001
521	C11	B97	Post-op CD	Control group
0.8825324180015256
522	C11	B98	Post-op CD	Control group
0.8907322654462243
523	C11	B100	Post-op CD	Control group
0.889397406559878
524	C11	B112	Post-op CD	Control group
0.9153318077803204
525	C11	B115	Post-op CD	Control group
0.8741418764302059
526	C11	I1	Post-op CD	Control group
0.9174294431731502
527	C11	I3	Post-op CD	Control group
0.9183829138062548
528	C11	I6	Post-op CD	Control group
0.8482074752097636
529	C11	I8	Post-op CD	Control group
0.9475591151792525
530	C11	I10	Post-op CD	Control group
0.8964530892448512
531	C11	I11	Post-op CD	Control group

0.9715865751334859
532	C11	I13	Post-op CD	Control group
0.8773836765827613
533	C11	I15	Post-op CD	Control group
0.8325705568268498
534	C11	I17	Post-op CD	Control group
0.9795957284515637
535	C11	I18	Post-op CD	Control group
0.8525934401220442
536	C11	I19	Post-op CD	Control group
0.9490846681922197
537	C11	I22	Post-op CD	Control group
0.9353546910755148
538	C11	I23	Post-op CD	Control group
0.8604118993135011
539	C11	I24	Post-op CD	Control group
0.9229595728451564
540	C11	I25	Post-op CD	Control group
0.8794813119755912
541	C11	I26	Post-op CD	Control group
0.8335240274599542
542	C11	I27	Post-op CD	Control group
0.8197940503432495
543	C11	I28	Post-op CD	Control group
0.8483981693363845
544	C11	I29	Post-op CD	Control group
0.912090007627765
545	C11	I30	Post-op CD	Control group
0.7818459191456903
546	C11	I31	Post-op CD	Control group
0.9591914569031273
547	C11	I32	Post-op CD	Control group
0.8958810068649885
548	C11	I33	Post-op CD	Control group
0.9191456903127384
549	C11	I34	Post-op CD	Control group
0.795766590389016
550	C11	I35	Post-op CD	Control group
0.9071319603356217
551	C11	I36	Post-op CD	Control group
0.9925629290617849
552	C11	I37	Post-op CD	Control group
0.9530892448512586
553	C15	B27	Post-op CD	Control group
0.9496567505720824
554	C15	B66	Post-op CD	Control group
0.8428680396643783
555	C15	B86	Post-op CD	Control group
0.8583142639206712
556	C15	B97	Post-op CD	Control group
0.8756674294431731
557	C15	B98	Post-op CD	Control group
0.8920671243325705
558	C15	B100	Post-op CD	Control group

0.9221967963386728
559 C15 B112 Post-op CD Control group
0.9342105263157895
560 C15 B115 Post-op CD Control group
0.8966437833714722
561 C15 I1 Post-op CD Control group
0.8972158657513348
562 C15 I3 Post-op CD Control group
0.8787185354691075
563 C15 I6 Post-op CD Control group
0.7837528604118993
564 C15 I8 Post-op CD Control group
0.9448893974065599
565 C15 I10 Post-op CD Control group
0.8422959572845157
566 C15 I11 Post-op CD Control group
0.931350114416476
567 C15 I13 Post-op CD Control group
0.8909229595728452
568 C15 I15 Post-op CD Control group
0.8190312738367659
569 C15 I17 Post-op CD Control group
0.9660564454614798
570 C15 I18 Post-op CD Control group
0.8299008390541571
571 C15 I19 Post-op CD Control group
0.9353546910755148
572 C15 I22 Post-op CD Control group
0.9126620900076278
573 C15 I23 Post-op CD Control group
0.8649885583524027
574 C15 I24 Post-op CD Control group
0.9220061022120518
575 C15 I25 Post-op CD Control group
0.8546910755148741
576 C15 I26 Post-op CD Control group
0.8209382151029748
577 C15 I27 Post-op CD Control group
0.7389397406559878
578 C15 I28 Post-op CD Control group
0.9101830663615561
579 C15 I29 Post-op CD Control group
0.9282990083905416
580 C15 I30 Post-op CD Control group
0.7648741418764302
581 C15 I31 Post-op CD Control group
0.9557589626239512
582 C15 I32 Post-op CD Control group
0.9347826086956522
583 C15 I33 Post-op CD Control group
0.8625095347063311
584 C15 I34 Post-op CD Control group
0.8295194508009154
585 C15 I35 Post-op CD Control group

0.9132341723874905
586 C15 I36 Post-op CD Control group
0.982837528604119
587 C15 I37 Post-op CD Control group
0.965675057208238
588 C19 B27 Post-op CD Control group
0.8295194508009154
589 C19 B66 Post-op CD Control group
0.8108314263920672
590 C19 B86 Post-op CD Control group
0.5932494279176201
591 C19 B97 Post-op CD Control group
0.8709000762776506
592 C19 B98 Post-op CD Control group
0.8392448512585813
593 C19 B100 Post-op CD Control group
0.9759725400457666
594 C19 B112 Post-op CD Control group
0.7469488939740656
595 C19 B115 Post-op CD Control group
0.8647978642257819
596 C19 I1 Post-op CD Control group
0.8916857360793288
597 C19 I3 Post-op CD Control group
0.9309687261632341
598 C19 I6 Post-op CD Control group
0.7990083905415714
599 C19 I8 Post-op CD Control group
0.9229595728451564
600 C19 I10 Post-op CD Control group
0.8857742181540809
601 C19 I11 Post-op CD Control group
0.950419527078566
602 C19 I13 Post-op CD Control group
0.8506864988558352
603 C19 I15 Post-op CD Control group
0.8278032036613272
604 C19 I17 Post-op CD Control group
0.7530511060259344
605 C19 I18 Post-op CD Control group
0.8600305110602593
606 C19 I19 Post-op CD Control group
0.7688787185354691
607 C19 I22 Post-op CD Control group
0.9010297482837528
608 C19 I23 Post-op CD Control group
0.8382913806254767
609 C19 I24 Post-op CD Control group
0.5921052631578947
610 C19 I25 Post-op CD Control group
0.8638443935926774
611 C19 I26 Post-op CD Control group
0.8266590389016019
612 C19 I27 Post-op CD Control group

0.8119755911517925
613	C19	I28	Post-op CD	Control group
0.9006483600305111
614	C19	I29	Post-op CD	Control group
0.8979786422578184
615	C19	I30	Post-op CD	Control group
0.7688787185354691
616	C19	I31	Post-op CD	Control group
0.915903890160183
617	C19	I32	Post-op CD	Control group
0.9210526315789473
618	C19	I33	Post-op CD	Control group
0.623951182303585
619	C19	I34	Post-op CD	Control group
0.6781083142639207
620	C19	I35	Post-op CD	Control group
0.873951182303585
621	C19	I36	Post-op CD	Control group
0.9858886346300534
622	C19	I37	Post-op CD	Control group
0.937070938215103
623	C22	B27	Post-op CD	Control group
0.9298245614035088
624	C22	B66	Post-op CD	Control group
0.9477498093058734
625	C22	B86	Post-op CD	Control group
0.9448893974065599
626	C22	B97	Post-op CD	Control group
0.9105644546147978
627	C22	B98	Post-op CD	Control group
0.9000762776506483
628	C22	B100	Post-op CD	Control group
0.9096109839816934
629	C22	B112	Post-op CD	Control group
0.9376430205949656
630	C22	B115	Post-op CD	Control group
0.9086575133485889
631	C22	I1	Post-op CD	Control group
0.933257055682685
632	C22	I3	Post-op CD	Control group
0.9557589626239512
633	C22	I6	Post-op CD	Control group
0.9347826086956522
634	C22	I8	Post-op CD	Control group
0.9475591151792525
635	C22	I10	Post-op CD	Control group
0.9605263157894737
636	C22	I11	Post-op CD	Control group
0.9452707856598017
637	C22	I13	Post-op CD	Control group
0.956140350877193
638	C22	I15	Post-op CD	Control group
0.8499237223493517
639	C22	I17	Post-op CD	Control group

0.971205186880244
640	C22	I18	Post-op CD	Control group
0.7580091533180778
641	C22	I19	Post-op CD	Control group
0.9641495041952708
642	C22	I22	Post-op CD	Control group
0.8098779557589626
643	C22	I23	Post-op CD	Control group
0.9448893974065599
644	C22	I24	Post-op CD	Control group
0.9588100686498856
645	C22	I25	Post-op CD	Control group
0.9527078565980168
646	C22	I26	Post-op CD	Control group
0.948512585812357
647	C22	I27	Post-op CD	Control group
0.8659420289855072
648	C22	I28	Post-op CD	Control group
0.9000762776506483
649	C22	I29	Post-op CD	Control group
0.9607170099160945
650	C22	I30	Post-op CD	Control group
0.9450800915331807
651	C22	I31	Post-op CD	Control group
0.9736842105263158
652	C22	I32	Post-op CD	Control group
0.9754004576659039
653	C22	I33	Post-op CD	Control group
0.9702517162471396
654	C22	I34	Post-op CD	Control group
0.9651029748283753
655	C22	I35	Post-op CD	Control group
0.9374523264683448
656	C22	I36	Post-op CD	Control group
0.7654462242562929
657	C22	I37	Post-op CD	Control group
0.7059496567505721
658	C26	B27	Post-op CD	Control group
0.9651029748283753
659	C26	B66	Post-op CD	Control group
0.9319221967963387
660	C26	B86	Post-op CD	Control group
0.9637681159420289
661	C26	B97	Post-op CD	Control group
0.90255530129672
662	C26	B98	Post-op CD	Control group
0.9157131960335622
663	C26	B100	Post-op CD	Control group
0.8832951945080092
664	C26	B112	Post-op CD	Control group
0.9601449275362319
665	C26	B115	Post-op CD	Control group
0.9586193745232647
666	C26	I1	Post-op CD	Control group

0.9477498093058734
667	C26	I3	Post-op CD	Control group
0.8735697940503433
668	C26	I6	Post-op CD	Control group
0.9244851258581236
669	C26	I8	Post-op CD	Control group
0.9710144927536232
670	C26	I10	Post-op CD	Control group
0.9040808543096872
671	C26	I11	Post-op CD	Control group
0.9414569031273837
672	C26	I13	Post-op CD	Control group
0.9170480549199085
673	C26	I15	Post-op CD	Control group
0.9448893974065599
674	C26	I17	Post-op CD	Control group
0.9593821510297483
675	C26	I18	Post-op CD	Control group
0.8909229595728452
676	C26	I19	Post-op CD	Control group
0.9818840579710145
677	C26	I22	Post-op CD	Control group
0.9549961861174676
678	C26	I23	Post-op CD	Control group
0.8968344774980931
679	C26	I24	Post-op CD	Control group
0.9679633867276888
680	C26	I25	Post-op CD	Control group
0.9172387490465294
681	C26	I26	Post-op CD	Control group
0.8972158657513348
682	C26	I27	Post-op CD	Control group
0.7990083905415714
683	C26	I28	Post-op CD	Control group
0.8573607932875668
684	C26	I29	Post-op CD	Control group
0.9727307398932112
685	C26	I30	Post-op CD	Control group
0.9191456903127384
686	C26	I31	Post-op CD	Control group
0.9570938215102975
687	C26	I32	Post-op CD	Control group
0.9559496567505721
688	C26	I33	Post-op CD	Control group
0.9326849733028223
689	C26	I34	Post-op CD	Control group
0.9212433257055682
690	C26	I35	Post-op CD	Control group
0.8743325705568269
691	C26	I36	Post-op CD	Control group
0.9860793287566743
692	C26	I37	Post-op CD	Control group
0.9666285278413425
693	C28	B27	Post-op CD	Control group

0.9475591151792525
694 C28 B66 Post-op CD Control group
0.8115942028985508
695 C28 B86 Post-op CD Control group
0.8954996186117468
696 C28 B97 Post-op CD Control group
0.8804347826086957
697 C28 B98 Post-op CD Control group
0.9113272311212814
698 C28 B100 Post-op CD Control group
0.9376430205949656
699 C28 B112 Post-op CD Control group
0.9443173150266971
700 C28 B115 Post-op CD Control group
0.8592677345537757
701 C28 I1 Post-op CD Control group
0.9088482074752098
702 C28 I3 Post-op CD Control group
0.8342868039664378
703 C28 I6 Post-op CD Control group
0.8154080854309688
704 C28 I8 Post-op CD Control group
0.9448893974065599
705 C28 I10 Post-op CD Control group
0.9164759725400458
706 C28 I11 Post-op CD Control group
0.9427917620137299
707 C28 I13 Post-op CD Control group
0.8661327231121282
708 C28 I15 Post-op CD Control group
0.7982456140350878
709 C28 I17 Post-op CD Control group
0.9584286803966438
710 C28 I18 Post-op CD Control group
0.8852021357742181
711 C28 I19 Post-op CD Control group
0.933257055682685
712 C28 I22 Post-op CD Control group
0.9042715484363082
713 C28 I23 Post-op CD Control group
0.855072463768116
714 C28 I24 Post-op CD Control group
0.9296338672768879
715 C28 I25 Post-op CD Control group
0.8800533943554538
716 C28 I26 Post-op CD Control group
0.8007246376811594
717 C28 I27 Post-op CD Control group
0.8232265446224256
718 C28 I28 Post-op CD Control group
0.9412662090007627
719 C28 I29 Post-op CD Control group
0.8709000762776506
720 C28 I30 Post-op CD Control group

0.7515255530129672
721 C28 I31 Post-op CD Control group
0.9239130434782609
722 C28 I32 Post-op CD Control group
0.9445080091533181
723 C28 I33 Post-op CD Control group
0.9323035850495804
724 C28 I34 Post-op CD Control group
0.8800533943554538
725 C28 I35 Post-op CD Control group
0.940884820747521
726 C28 I36 Post-op CD Control group
0.982837528604119
727 C28 I37 Post-op CD Control group
0.9603356216628528
728 C31 B27 Post-op CD Control group
0.9746376811594203
729 C31 B66 Post-op CD Control group
0.8781464530892449
730 C31 B86 Post-op CD Control group
0.9719679633867276
731 C31 B97 Post-op CD Control group
0.9099923722349351
732 C31 B98 Post-op CD Control group
0.9748283752860412
733 C31 B100 Post-op CD Control group
0.8436308161708619
734 C31 B112 Post-op CD Control group
0.9900839054157132
735 C31 B115 Post-op CD Control group
0.9679633867276888
736 C31 I1 Post-op CD Control group
0.9792143401983219
737 C31 I3 Post-op CD Control group
0.8430587337909993
738 C31 I6 Post-op CD Control group
0.9323035850495804
739 C31 I8 Post-op CD Control group
0.9889397406559878
740 C31 I10 Post-op CD Control group
0.9559496567505721
741 C31 I11 Post-op CD Control group
0.9677726926010679
742 C31 I13 Post-op CD Control group
0.9710144927536232
743 C31 I15 Post-op CD Control group
0.8287566742944318
744 C31 I17 Post-op CD Control group
0.9944698703279939
745 C31 I18 Post-op CD Control group
0.9345919145690312
746 C31 I19 Post-op CD Control group
0.9816933638443935
747 C31 I22 Post-op CD Control group

0.9469870327993898
748 C31 I23 Post-op CD Control group
0.958047292143402
749 C31 I24 Post-op CD Control group
0.9876048817696415
750 C31 I25 Post-op CD Control group
0.971395881006865
751 C31 I26 Post-op CD Control group
0.9405034324942791
752 C31 I27 Post-op CD Control group
0.8852021357742181
753 C31 I28 Post-op CD Control group
0.9628146453089245
754 C31 I29 Post-op CD Control group
0.9622425629290617
755 C31 I30 Post-op CD Control group
0.9220061022120518
756 C31 I31 Post-op CD Control group
0.9843630816170862
757 C31 I32 Post-op CD Control group
0.9685354691075515
758 C31 I33 Post-op CD Control group
0.9855072463768116
759 C31 I34 Post-op CD Control group
0.9803585049580473
760 C31 I35 Post-op CD Control group
0.9614797864225781
761 C31 I36 Post-op CD Control group
0.9590007627765065
762 C31 I37 Post-op CD Control group
0.9740655987795576
763 C35 B27 Post-op CD Control group
0.7501906941266209
764 C35 B66 Post-op CD Control group
0.8503051106025934
765 C35 B86 Post-op CD Control group
0.8350495804729214
766 C35 B97 Post-op CD Control group
0.88558352402746
767 C35 B98 Post-op CD Control group
0.88558352402746
768 C35 B100 Post-op CD Control group
0.9450800915331807
769 C35 B112 Post-op CD Control group
0.8632723112128147
770 C35 B115 Post-op CD Control group
0.8897787948131197
771 C35 I1 Post-op CD Control group
0.92372234935164
772 C35 I3 Post-op CD Control group
0.919908466819222
773 C35 I6 Post-op CD Control group
0.8354309687261632
774 C35 I8 Post-op CD Control group

0.9258199847444699
775	C35	I10	Post-op CD	Control group
0.8964530892448512
776	C35	I11	Post-op CD	Control group
0.9858886346300534
777	C35	I13	Post-op CD	Control group
0.8522120518688024
778	C35	I15	Post-op CD	Control group
0.801487414187643
779	C35	I17	Post-op CD	Control group
0.8127383676582761
780	C35	I18	Post-op CD	Control group
0.9200991609458429
781	C35	I19	Post-op CD	Control group
0.8688024408848207
782	C35	I22	Post-op CD	Control group
0.940884820747521
783	C35	I23	Post-op CD	Control group
0.8718535469107551
784	C35	I24	Post-op CD	Control group
0.7278794813119756
785	C35	I25	Post-op CD	Control group
0.9193363844393593
786	C35	I26	Post-op CD	Control group
0.8037757437070938
787	C35	I27	Post-op CD	Control group
0.8449656750572082
788	C35	I28	Post-op CD	Control group
0.9815026697177727
789	C35	I29	Post-op CD	Control group
0.9054157131960335
790	C35	I30	Post-op CD	Control group
0.8249427917620137
791	C35	I31	Post-op CD	Control group
0.9755911517925248
792	C35	I32	Post-op CD	Control group
0.9385964912280702
793	C35	I33	Post-op CD	Control group
0.7187261632341724
794	C35	I34	Post-op CD	Control group
0.6924103737604882
795	C35	I35	Post-op CD	Control group
0.9380244088482075
796	C35	I36	Post-op CD	Control group
0.9918001525553013
797	C35	I37	Post-op CD	Control group
0.9740655987795576
798	C38	B27	Post-op CD	Control group
0.9744469870327994
799	C38	B66	Post-op CD	Control group
0.9260106788710908
800	C38	B86	Post-op CD	Control group
0.9296338672768879
801	C38	B97	Post-op CD	Control group

0.9740655987795576
802 C38 B98 Post-op CD Control group
0.971205186880244
803 C38 B100 Post-op CD Control group
0.8094965675057209
804 C38 B112 Post-op CD Control group
0.9898932112890922
805 C38 B115 Post-op CD Control group
0.9527078565980168
806 C38 I1 Post-op CD Control group
0.9734935163996948
807 C38 I3 Post-op CD Control group
0.9660564454614798
808 C38 I6 Post-op CD Control group
0.9548054919908466
809 C38 I8 Post-op CD Control group
0.9755911517925248
810 C38 I10 Post-op CD Control group
0.9719679633867276
811 C38 I11 Post-op CD Control group
0.9565217391304348
812 C38 I13 Post-op CD Control group
0.9677726926010679
813 C38 I15 Post-op CD Control group
0.8501144164759725
814 C38 I17 Post-op CD Control group
0.9815026697177727
815 C38 I18 Post-op CD Control group
0.9740655987795576
816 C38 I19 Post-op CD Control group
0.9908466819221968
817 C38 I22 Post-op CD Control group
0.9628146453089245
818 C38 I23 Post-op CD Control group
0.975209763539283
819 C38 I24 Post-op CD Control group
0.9959954233409611
820 C38 I25 Post-op CD Control group
0.9742562929061785
821 C38 I26 Post-op CD Control group
0.9797864225781846
822 C38 I27 Post-op CD Control group
0.9738749046529367
823 C38 I28 Post-op CD Control group
0.9418382913806255
824 C38 I29 Post-op CD Control group
0.988558352402746
825 C38 I30 Post-op CD Control group
0.9170480549199085
826 C38 I31 Post-op CD Control group
0.9820747520976354
827 C38 I32 Post-op CD Control group
0.9971395881006865
828 C38 I33 Post-op CD Control group

0.9809305873379099
829 C38 I34 Post-op CD Control group
0.9755911517925248
830 C38 I35 Post-op CD Control group
0.9689168573607932
831 C38 I36 Post-op CD Control group
0.975209763539283
832 C38 I37 Post-op CD Control group
0.952326468344775
833 C40 B27 Post-op CD Control group
0.9567124332570557
834 C40 B66 Post-op CD Control group
0.9139969488939741
835 C40 B86 Post-op CD Control group
0.9364988558352403
836 C40 B97 Post-op CD Control group
0.885392829900839
837 C40 B98 Post-op CD Control group
0.9099923722349351
838 C40 B100 Post-op CD Control group
0.8878718535469108
839 C40 B112 Post-op CD Control group
0.9426010678871091
840 C40 B115 Post-op CD Control group
0.950419527078566
841 C40 I1 Post-op CD Control group
0.9248665141113653
842 C40 I3 Post-op CD Control group
0.7896643783371472
843 C40 I6 Post-op CD Control group
0.8905415713196033
844 C40 I8 Post-op CD Control group
0.952326468344775
845 C40 I10 Post-op CD Control group
0.8464912280701754
846 C40 I11 Post-op CD Control group
0.9002669717772692
847 C40 I13 Post-op CD Control group
0.9181922196796338
848 C40 I15 Post-op CD Control group
0.9574752097635393
849 C40 I17 Post-op CD Control group
0.9685354691075515
850 C40 I18 Post-op CD Control group
0.8627002288329519
851 C40 I19 Post-op CD Control group
0.9836003051106026
852 C40 I22 Post-op CD Control group
0.9212433257055682
853 C40 I23 Post-op CD Control group
0.9344012204424104
854 C40 I24 Post-op CD Control group
0.9345919145690312
855 C40 I25 Post-op CD Control group

0.8651792524790236
856 C40 I26 Post-op CD Control group
0.872234935163997
857 C40 I27 Post-op CD Control group
0.8335240274599542
858 C40 I28 Post-op CD Control group
0.6151792524790236
859 C40 I29 Post-op CD Control group
0.9221967963386728
860 C40 I30 Post-op CD Control group
0.8260869565217391
861 C40 I31 Post-op CD Control group
0.9044622425629291
862 C40 I32 Post-op CD Control group
0.9046529366895499
863 C40 I33 Post-op CD Control group
0.9473684210526315
864 C40 I34 Post-op CD Control group
0.9361174675819984
865 C40 I35 Post-op CD Control group
0.8373379099923722
866 C40 I36 Post-op CD Control group
0.9652936689549961
867 C40 I37 Post-op CD Control group
0.9098016781083142
868 C44 B27 Post-op CD Control group
0.9620518688024409
869 C44 B66 Post-op CD Control group
0.8447749809305873
870 C44 B86 Post-op CD Control group
0.8762395118230358
871 C44 B97 Post-op CD Control group
0.8880625476735317
872 C44 B98 Post-op CD Control group
0.8913043478260869
873 C44 B100 Post-op CD Control group
0.9769260106788711
874 C44 B112 Post-op CD Control group
0.92372234935164
875 C44 B115 Post-op CD Control group
0.9166666666666666
876 C44 I1 Post-op CD Control group
0.9265827612509535
877 C44 I3 Post-op CD Control group
0.9610983981693364
878 C44 I6 Post-op CD Control group
0.8432494279176201
879 C44 I8 Post-op CD Control group
0.9570938215102975
880 C44 I10 Post-op CD Control group
0.918001525553013
881 C44 I11 Post-op CD Control group
0.992372234935164
882 C44 I13 Post-op CD Control group

0.9118993135011442
883 C44 I15 Post-op CD Control group
0.7955758962623951
884 C44 I17 Post-op CD Control group
0.9822654462242563
885 C44 I18 Post-op CD Control group
0.9212433257055682
886 C44 I19 Post-op CD Control group
0.9488939740655988
887 C44 I22 Post-op CD Control group
0.9530892448512586
888 C44 I23 Post-op CD Control group
0.8935926773455377
889 C44 I24 Post-op CD Control group
0.9242944317315027
890 C44 I25 Post-op CD Control group
0.9092295957284515
891 C44 I26 Post-op CD Control group
0.8522120518688024
892 C44 I27 Post-op CD Control group
0.8531655225019069
893 C44 I28 Post-op CD Control group
0.9384057971014492
894 C44 I29 Post-op CD Control group
0.9231502669717773
895 C44 I30 Post-op CD Control group
0.6891685736079328
896 C44 I31 Post-op CD Control group
0.9950419527078566
897 C44 I32 Post-op CD Control group
0.9532799389778794
898 C44 I33 Post-op CD Control group
0.9118993135011442
899 C44 I34 Post-op CD Control group
0.8327612509534706
900 C44 I35 Post-op CD Control group
0.9527078565980168
901 C44 I36 Post-op CD Control group
0.9855072463768116
902 C44 I37 Post-op CD Control group
0.9729214340198322
903 C47 B27 Post-op CD Control group
0.9464149504195271
904 C47 B66 Post-op CD Control group
0.8995041952707856
905 C47 B86 Post-op CD Control group
0.8964530892448512
906 C47 B97 Post-op CD Control group
0.9164759725400458
907 C47 B98 Post-op CD Control group
0.9210526315789473
908 C47 B100 Post-op CD Control group
0.9836003051106026
909 C47 B112 Post-op CD Control group

0.9380244088482075
910 C47 B115 Post-op CD Control group
0.906559877955759
911 C47 I1 Post-op CD Control group
0.9424103737604882
912 C47 I3 Post-op CD Control group
0.9900839054157132
913 C47 I6 Post-op CD Control group
0.860602593440122
914 C47 I8 Post-op CD Control group
0.9563310450038138
915 C47 I10 Post-op CD Control group
0.9597635392829901
916 C47 I11 Post-op CD Control group
0.9910373760488177
917 C47 I13 Post-op CD Control group
0.9254385964912281
918 C47 I15 Post-op CD Control group
0.8680396643783371
919 C47 I17 Post-op CD Control group
0.971395881006865
920 C47 I18 Post-op CD Control group
0.9311594202898551
921 C47 I19 Post-op CD Control group
0.9036994660564455
922 C47 I22 Post-op CD Control group
0.952326468344775
923 C47 I23 Post-op CD Control group
0.9115179252479023
924 C47 I24 Post-op CD Control group
0.9307780320366132
925 C47 I25 Post-op CD Control group
0.9336384439359268
926 C47 I26 Post-op CD Control group
0.8676582761250954
927 C47 I27 Post-op CD Control group
0.8974065598779558
928 C47 I28 Post-op CD Control group
0.982837528604119
929 C47 I29 Post-op CD Control group
0.9511823035850496
930 C47 I30 Post-op CD Control group
0.8436308161708619
931 C47 I31 Post-op CD Control group
0.9902745995423341
932 C47 I32 Post-op CD Control group
0.9952326468344775
933 C47 I33 Post-op CD Control group
0.9252479023646072
934 C47 I34 Post-op CD Control group
0.8331426392067124
935 C47 I35 Post-op CD Control group
0.9826468344774981
936 C47 I36 Post-op CD Control group

0.9954233409610984
937 C47 I37 Post-op CD Control group
0.9826468344774981
938 C48 B27 Post-op CD Control group
0.7229214340198322
939 C48 B66 Post-op CD Control group
0.9044622425629291
940 C48 B86 Post-op CD Control group
0.6287185354691075
941 C48 B97 Post-op CD Control group
0.8485888634630053
942 C48 B98 Post-op CD Control group
0.8871090770404272
943 C48 B100 Post-op CD Control group
0.9567124332570557
944 C48 B112 Post-op CD Control group
0.677536231884058
945 C48 B115 Post-op CD Control group
0.8640350877192983
946 C48 I1 Post-op CD Control group
0.8659420289855072
947 C48 I3 Post-op CD Control group
0.9139969488939741
948 C48 I6 Post-op CD Control group
0.8575514874141876
949 C48 I8 Post-op CD Control group
0.8545003813882532
950 C48 I10 Post-op CD Control group
0.8937833714721587
951 C48 I11 Post-op CD Control group
0.9317315026697178
952 C48 I13 Post-op CD Control group
0.837909992372235
953 C48 I15 Post-op CD Control group
0.9063691838291381
954 C48 I17 Post-op CD Control group
0.6584668192219679
955 C48 I18 Post-op CD Control group
0.8489702517162472
956 C48 I19 Post-op CD Control group
0.3489702517162471
957 C48 I22 Post-op CD Control group
0.8329519450800915
958 C48 I23 Post-op CD Control group
0.8464912280701754
959 C48 I24 Post-op CD Control group
0.6128909229595728
960 C48 I25 Post-op CD Control group
0.8382913806254767
961 C48 I26 Post-op CD Control group
0.8808161708619374
962 C48 I27 Post-op CD Control group
0.835812356979405
963 C48 I28 Post-op CD Control group

0.9429824561403509
964	C48	I29	Post-op CD	Control group
0.8954996186117468
965	C48	I30	Post-op CD	Control group
0.8979786422578184
966	C48	I31	Post-op CD	Control group
0.9336384439359268
967	C48	I32	Post-op CD	Control group
0.9454614797864226
968	C48	I33	Post-op CD	Control group
0.5692219679633868
969	C48	I34	Post-op CD	Control group
0.7395118230358505
970	C48	I35	Post-op CD	Control group
0.900839054157132
971	C48	I36	Post-op CD	Control group
0.9778794813119756
972	C48	I37	Post-op CD	Control group
0.9382151029748284
973	C49	B27	Post-op CD	Control group
0.8373379099923722
974	C49	B66	Post-op CD	Control group
0.950228832951945
975	C49	B86	Post-op CD	Control group
0.8140732265446224
976	C49	B97	Post-op CD	Control group
0.8495423340961098
977	C49	B98	Post-op CD	Control group
0.9002669717772692
978	C49	B100	Post-op CD	Control group
0.940884820747521
979	C49	B112	Post-op CD	Control group
0.8274218154080855
980	C49	B115	Post-op CD	Control group
0.881769641495042
981	C49	I1	Post-op CD	Control group
0.9202898550724637
982	C49	I3	Post-op CD	Control group
0.8728070175438597
983	C49	I6	Post-op CD	Control group
0.9073226544622426
984	C49	I8	Post-op CD	Control group
0.9090389016018307
985	C49	I10	Post-op CD	Control group
0.9286803966437833
986	C49	I11	Post-op CD	Control group
0.8943554538520213
987	C49	I13	Post-op CD	Control group
0.8823417238749046
988	C49	I15	Post-op CD	Control group
0.9372616323417239
989	C49	I17	Post-op CD	Control group
0.8470633104500381
990	C49	I18	Post-op CD	Control group

0.8537376048817696
991	C49	I19	Post-op CD	Control group
0.6062166285278413
992	C49	I22	Post-op CD	Control group
0.8823417238749046
993	C49	I23	Post-op CD	Control group
0.92372234935164
994	C49	I24	Post-op CD	Control group
0.8487795575896262
995	C49	I25	Post-op CD	Control group
0.839626239511823
996	C49	I26	Post-op CD	Control group
0.9528985507246377
997	C49	I27	Post-op CD	Control group
0.9244851258581236
998	C49	I28	Post-op CD	Control group
0.7065217391304348
999	C49	I29	Post-op CD	Control group
0.9361174675819984
1000	C49	I30	Post-op CD	Control group
0.9317315026697178
1001	C49	I31	Post-op CD	Control group
0.9433638443935927
1002	C49	I32	Post-op CD	Control group
0.9441266209000763
1003	C49	I33	Post-op CD	Control group
0.7915713196033562
1004	C49	I34	Post-op CD	Control group
0.8562166285278413
1005	C49	I35	Post-op CD	Control group
0.8720442410373761
1006	C49	I36	Post-op CD	Control group
0.9670099160945843
1007	C49	I37	Post-op CD	Control group
0.9395499618611747
1008	C53	B27	Post-op CD	Control group
0.8222730739893211
1009	C53	B66	Post-op CD	Control group
0.8575514874141876
1010	C53	B86	Post-op CD	Control group
0.7206331045003814
1011	C53	B97	Post-op CD	Control group
0.8537376048817696
1012	C53	B98	Post-op CD	Control group
0.8790999237223494
1013	C53	B100	Post-op CD	Control group
0.9452707856598017
1014	C53	B112	Post-op CD	Control group
0.7694508009153318
1015	C53	B115	Post-op CD	Control group
0.8478260869565217
1016	C53	I1	Post-op CD	Control group
0.9021739130434783
1017	C53	I3	Post-op CD	Control group

0.9338291380625476
1018	C53	I6	Post-op CD	Control group
0.7848970251716247
1019	C53	I8	Post-op CD	Control group
0.8857742181540809
1020	C53	I10	Post-op CD	Control group
0.9210526315789473
1021	C53	I11	Post-op CD	Control group
0.9488939740655988
1022	C53	I13	Post-op CD	Control group
0.8436308161708619
1023	C53	I15	Post-op CD	Control group
0.858886346300534
1024	C53	I17	Post-op CD	Control group
0.7961479786422578
1025	C53	I18	Post-op CD	Control group
0.8781464530892449
1026	C53	I19	Post-op CD	Control group
0.4973302822273074
1027	C53	I22	Post-op CD	Control group
0.8764302059496567
1028	C53	I23	Post-op CD	Control group
0.8451563691838292
1029	C53	I24	Post-op CD	Control group
0.7715484363081617
1030	C53	I25	Post-op CD	Control group
0.8543096872616324
1031	C53	I26	Post-op CD	Control group
0.8501144164759725
1032	C53	I27	Post-op CD	Control group
0.8527841342486652
1033	C53	I28	Post-op CD	Control group
0.9528985507246377
1034	C53	I29	Post-op CD	Control group
0.9231502669717773
1035	C53	I30	Post-op CD	Control group
0.8171243325705568
1036	C53	I31	Post-op CD	Control group
0.9435545385202135
1037	C53	I32	Post-op CD	Control group
0.9635774218154081
1038	C53	I33	Post-op CD	Control group
0.7374141876430206
1039	C53	I34	Post-op CD	Control group
0.7276887871853547
1040	C53	I35	Post-op CD	Control group
0.9172387490465294
1041	C53	I36	Post-op CD	Control group
0.9876048817696415
1042	C53	I37	Post-op CD	Control group
0.9555682684973302
1043	C56	B27	Post-op CD	Control group
0.7850877192982456
1044	C56	B66	Post-op CD	Control group

0.8171243325705568
1045 C56 B86 Post-op CD Control group
0.679252479023646
1046 C56 B97 Post-op CD Control group
0.8054919908466819
1047 C56 B98 Post-op CD Control group
0.8756674294431731
1048 C56 B100 Post-op CD Control group
0.9460335621662853
1049 C56 B112 Post-op CD Control group
0.7145308924485125
1050 C56 B115 Post-op CD Control group
0.8032036613272311
1051 C56 I1 Post-op CD Control group
0.8352402745995423
1052 C56 I3 Post-op CD Control group
0.8136918382913806
1053 C56 I6 Post-op CD Control group
0.6983218916857361
1054 C56 I8 Post-op CD Control group
0.8430587337909993
1055 C56 I10 Post-op CD Control group
0.8676582761250954
1056 C56 I11 Post-op CD Control group
0.9166666666666666
1057 C56 I13 Post-op CD Control group
0.8205568268497331
1058 C56 I15 Post-op CD Control group
0.8310450038138826
1059 C56 I17 Post-op CD Control group
0.746186117467582
1060 C56 I18 Post-op CD Control group
0.7822273073989321
1061 C56 I19 Post-op CD Control group
0.4334477498093059
1062 C56 I22 Post-op CD Control group
0.8047292143401983
1063 C56 I23 Post-op CD Control group
0.8199847444698704
1064 C56 I24 Post-op CD Control group
0.7545766590389016
1065 C56 I25 Post-op CD Control group
0.7929061784897025
1066 C56 I26 Post-op CD Control group
0.811022120518688
1067 C56 I27 Post-op CD Control group
0.721205186880244
1068 C56 I28 Post-op CD Control group
0.8993135011441648
1069 C56 I29 Post-op CD Control group
0.8728070175438597
1070 C56 I30 Post-op CD Control group
0.6939359267734554
1071 C56 I31 Post-op CD Control group

0.8951182303585049
1072 C56 I32 Post-op CD Control group
0.910373760488177
1073 C56 I33 Post-op CD Control group
0.6952707856598017
1074 C56 I34 Post-op CD Control group
0.7276887871853547
1075 C56 I35 Post-op CD Control group
0.8995041952707856
1076 C56 I36 Post-op CD Control group
0.9788329519450801
1077 C56 I37 Post-op CD Control group
0.9427917620137299
1078 C60 B27 Post-op CD Control group
0.7618230358504958
1079 C60 B66 Post-op CD Control group
0.8363844393592678
1080 C60 B86 Post-op CD Control group
0.7429443173150267
1081 C60 B97 Post-op CD Control group
0.8440122044241037
1082 C60 B98 Post-op CD Control group
0.8621281464530892
1083 C60 B100 Post-op CD Control group
0.9088482074752098
1084 C60 B112 Post-op CD Control group
0.7759344012204424
1085 C60 B115 Post-op CD Control group
0.8213196033562167
1086 C60 I1 Post-op CD Control group
0.830091533180778
1087 C60 I3 Post-op CD Control group
0.9050343249427918
1088 C60 I6 Post-op CD Control group
0.7673531655225019
1089 C60 I8 Post-op CD Control group
0.868230358504958
1090 C60 I10 Post-op CD Control group
0.9088482074752098
1091 C60 I11 Post-op CD Control group
0.8733790999237223
1092 C60 I13 Post-op CD Control group
0.8348588863463006
1093 C60 I15 Post-op CD Control group
0.7116704805491991
1094 C60 I17 Post-op CD Control group
0.8043478260869565
1095 C60 I18 Post-op CD Control group
0.818649885583524
1096 C60 I19 Post-op CD Control group
0.587909992372235
1097 C60 I22 Post-op CD Control group
0.7663996948893974
1098 C60 I23 Post-op CD Control group

0.8360030511060259
1099	C60	I24	Post-op CD	Control group
0.8056826849733029
1100	C60	I25	Post-op CD	Control group
0.843440122044241
1101	C60	I26	Post-op CD	Control group
0.8546910755148741
1102	C60	I27	Post-op CD	Control group
0.8413424866514111
1103	C60	I28	Post-op CD	Control group
0.8630816170861938
1104	C60	I29	Post-op CD	Control group
0.9246758199847445
1105	C60	I30	Post-op CD	Control group
0.7864225781845919
1106	C60	I31	Post-op CD	Control group
0.9107551487414187
1107	C60	I32	Post-op CD	Control group
0.9553775743707094
1108	C60	I33	Post-op CD	Control group
0.7561022120518688
1109	C60	I34	Post-op CD	Control group
0.7273073989321129
1110	C60	I35	Post-op CD	Control group
0.9080854309687262
1111	C60	I36	Post-op CD	Control group
0.8693745232646835
1112	C60	I37	Post-op CD	Control group
0.8011060259344012
1113	C62	B27	Post-op CD	Control group
0.8440122044241037
1114	C62	B66	Post-op CD	Control group
0.8840579710144928
1115	C62	B86	Post-op CD	Control group
0.7694508009153318
1116	C62	B97	Post-op CD	Control group
0.8790999237223494
1117	C62	B98	Post-op CD	Control group
0.9122807017543859
1118	C62	B100	Post-op CD	Control group
0.782608695652174
1119	C62	B112	Post-op CD	Control group
0.8056826849733029
1120	C62	B115	Post-op CD	Control group
0.9139969488939741
1121	C62	I1	Post-op CD	Control group
0.910373760488177
1122	C62	I3	Post-op CD	Control group
0.8846300533943554
1123	C62	I6	Post-op CD	Control group
0.8432494279176201
1124	C62	I8	Post-op CD	Control group
0.9050343249427918
1125	C62	I10	Post-op CD	Control group

0.9088482074752098
1126 C62 I11 Post-op CD Control group
0.9231502669717773
1127 C62 I13 Post-op CD Control group
0.8781464530892449
1128 C62 I15 Post-op CD Control group
0.8888253241800153
1129 C62 I17 Post-op CD Control group
0.8251334858886347
1130 C62 I18 Post-op CD Control group
0.8667048054919908
1131 C62 I19 Post-op CD Control group
0.5734172387490465
1132 C62 I22 Post-op CD Control group
0.8686117467581999
1133 C62 I23 Post-op CD Control group
0.912090007627765
1134 C62 I24 Post-op CD Control group
0.8142639206712433
1135 C62 I25 Post-op CD Control group
0.8909229595728452
1136 C62 I26 Post-op CD Control group
0.929252479023646
1137 C62 I27 Post-op CD Control group
0.8579328756674295
1138 C62 I28 Post-op CD Control group
0.9290617848970252
1139 C62 I29 Post-op CD Control group
0.9214340198321892
1140 C62 I30 Post-op CD Control group
0.8872997711670481
1141 C62 I31 Post-op CD Control group
0.9374523264683448
1142 C62 I32 Post-op CD Control group
0.9315408085430968
1143 C62 I33 Post-op CD Control group
0.7835621662852784
1144 C62 I34 Post-op CD Control group
0.8436308161708619
1145 C62 I35 Post-op CD Control group
0.9288710907704043
1146 C62 I36 Post-op CD Control group
0.8464912280701754
1147 C62 I37 Post-op CD Control group
0.9412662090007627
1148 C64 B27 Post-op CD Control group
0.811022120518688
1149 C64 B66 Post-op CD Control group
0.8251334858886347
1150 C64 B86 Post-op CD Control group
0.7418001525553013
1151 C64 B97 Post-op CD Control group
0.8625095347063311
1152 C64 B98 Post-op CD Control group

0.893211289092296
1153 C64 B100 Post-op CD Control group
0.5947749809305873
1154 C64 B112 Post-op CD Control group
0.7726926010678871
1155 C64 B115 Post-op CD Control group
0.8716628527841342
1156 C64 I1 Post-op CD Control group
0.847254004576659
1157 C64 I3 Post-op CD Control group
0.8941647597254004
1158 C64 I6 Post-op CD Control group
0.8661327231121282
1159 C64 I8 Post-op CD Control group
0.8594584286803967
1160 C64 I10 Post-op CD Control group
0.8943554538520213
1161 C64 I11 Post-op CD Control group
0.9096109839816934
1162 C64 I13 Post-op CD Control group
0.8691838291380626
1163 C64 I15 Post-op CD Control group
0.9126620900076278
1164 C64 I17 Post-op CD Control group
0.7974828375286042
1165 C64 I18 Post-op CD Control group
0.8741418764302059
1166 C64 I19 Post-op CD Control group
0.4977116704805492
1167 C64 I22 Post-op CD Control group
0.8623188405797102
1168 C64 I23 Post-op CD Control group
0.8514492753623188
1169 C64 I24 Post-op CD Control group
0.7902364607170099
1170 C64 I25 Post-op CD Control group
0.8546910755148741
1171 C64 I26 Post-op CD Control group
0.8811975591151793
1172 C64 I27 Post-op CD Control group
0.8869183829138062
1173 C64 I28 Post-op CD Control group
0.916094584286804
1174 C64 I29 Post-op CD Control group
0.8981693363844394
1175 C64 I30 Post-op CD Control group
0.8939740655987796
1176 C64 I31 Post-op CD Control group
0.9397406559877955
1177 C64 I32 Post-op CD Control group
0.954042715484363
1178 C64 I33 Post-op CD Control group
0.7520976353928299
1179 C64 I34 Post-op CD Control group

0.7816552250190694
1180 C64 I35 Post-op CD Control group
0.9006483600305111
1181 C64 I36 Post-op CD Control group
0.9063691838291381
1182 C64 I37 Post-op CD Control group
0.9122807017543859
1183 C65 B27 Post-op CD Control group
0.8094965675057209
1184 C65 B66 Post-op CD Control group
0.9246758199847445
1185 C65 B86 Post-op CD Control group
0.7027078565980168
1186 C65 B97 Post-op CD Control group
0.8857742181540809
1187 C65 B98 Post-op CD Control group
0.9130434782608695
1188 C65 B100 Post-op CD Control group
0.8808161708619374
1189 C65 B112 Post-op CD Control group
0.7433257055682685
1190 C65 B115 Post-op CD Control group
0.8718535469107551
1191 C65 I1 Post-op CD Control group
0.8945461479786423
1192 C65 I3 Post-op CD Control group
0.9307780320366132
1193 C65 I6 Post-op CD Control group
0.8790999237223494
1194 C65 I8 Post-op CD Control group
0.8787185354691075
1195 C65 I10 Post-op CD Control group
0.9147597254004577
1196 C65 I11 Post-op CD Control group
0.9366895499618612
1197 C65 I13 Post-op CD Control group
0.8731884057971014
1198 C65 I15 Post-op CD Control group
0.8155987795575896
1199 C65 I17 Post-op CD Control group
0.7723112128146453
1200 C65 I18 Post-op CD Control group
0.813119755911518
1201 C65 I19 Post-op CD Control group
0.4342105263157895
1202 C65 I22 Post-op CD Control group
0.7673531655225019
1203 C65 I23 Post-op CD Control group
0.9056064073226545
1204 C65 I24 Post-op CD Control group
0.7704042715484363
1205 C65 I25 Post-op CD Control group
0.8598398169336384
1206 C65 I26 Post-op CD Control group

0.9248665141113653
1207	C65	I27	Post-op CD	Control group
0.8972158657513348
1208	C65	I28	Post-op CD	Control group
0.9147597254004577
1209	C65	I29	Post-op CD	Control group
0.9012204424103738
1210	C65	I30	Post-op CD	Control group
0.9124713958810069
1211	C65	I31	Post-op CD	Control group
0.9374523264683448
1212	C65	I32	Post-op CD	Control group
0.9498474446987033
1213	C65	I33	Post-op CD	Control group
0.7402745995423341
1214	C65	I34	Post-op CD	Control group
0.8463005339435545
1215	C65	I35	Post-op CD	Control group
0.9277269260106789
1216	C65	I36	Post-op CD	Control group
0.7986270022883295
1217	C65	I37	Post-op CD	Control group
0.784324942791762
1218	C69	B27	Post-op CD	Control group
0.8239893211289092
1219	C69	B66	Post-op CD	Control group
0.8476353928299009
1220	C69	B86	Post-op CD	Control group
0.685163996948894
1221	C69	B97	Post-op CD	Control group
0.8255148741418764
1222	C69	B98	Post-op CD	Control group
0.8539282990083905
1223	C69	B100	Post-op CD	Control group
0.9086575133485889
1224	C69	B112	Post-op CD	Control group
0.7410373760488177
1225	C69	B115	Post-op CD	Control group
0.8789092295957285
1226	C69	I1	Post-op CD	Control group
0.8790999237223494
1227	C69	I3	Post-op CD	Control group
0.8165522501906941
1228	C69	I6	Post-op CD	Control group
0.86441647597254
1229	C69	I8	Post-op CD	Control group
0.8787185354691075
1230	C69	I10	Post-op CD	Control group
0.8665141113653699
1231	C69	I11	Post-op CD	Control group
0.8914950419527079
1232	C69	I13	Post-op CD	Control group
0.8325705568268498
1233	C69	I15	Post-op CD	Control group

0.9155225019069413
1234	C69	I17	Post-op CD	Control group
0.7677345537757437
1235	C69	I18	Post-op CD	Control group
0.8232265446224256
1236	C69	I19	Post-op CD	Control group
0.4652936689549962
1237	C69	I22	Post-op CD	Control group
0.855072463768116
1238	C69	I23	Post-op CD	Control group
0.8546910755148741
1239	C69	I24	Post-op CD	Control group
0.7561022120518688
1240	C69	I25	Post-op CD	Control group
0.8024408848207475
1241	C69	I26	Post-op CD	Control group
0.8733790999237223
1242	C69	I27	Post-op CD	Control group
0.8733790999237223
1243	C69	I28	Post-op CD	Control group
0.8226544622425629
1244	C69	I29	Post-op CD	Control group
0.8804347826086957
1245	C69	I30	Post-op CD	Control group
0.9014111365369947
1246	C69	I31	Post-op CD	Control group
0.9258199847444699
1247	C69	I32	Post-op CD	Control group
0.9029366895499619
1248	C69	I33	Post-op CD	Control group
0.7049961861174676
1249	C69	I34	Post-op CD	Control group
0.8310450038138826
1250	C69	I35	Post-op CD	Control group
0.8140732265446224
1251	C69	I36	Post-op CD	Control group
0.9822654462242563
1252	C69	I37	Post-op CD	Control group
0.9344012204424104
1253	C70	B27	Post-op CD	Control group
0.8213196033562167
1254	C70	B66	Post-op CD	Control group
0.9143783371472158
1255	C70	B86	Post-op CD	Control group
0.7494279176201373
1256	C70	B97	Post-op CD	Control group
0.8872997711670481
1257	C70	B98	Post-op CD	Control group
0.898932112890923
1258	C70	B100	Post-op CD	Control group
0.9069412662090007
1259	C70	B112	Post-op CD	Control group
0.7662090007627765
1260	C70	B115	Post-op CD	Control group

0.8686117467581999
1261	C70	I1	Post-op CD	Control group
0.9033180778032036
1262	C70	I3	Post-op CD	Control group
0.9113272311212814
1263	C70	I6	Post-op CD	Control group
0.879862700228833
1264	C70	I8	Post-op CD	Control group
0.8838672768878718
1265	C70	I10	Post-op CD	Control group
0.9067505720823799
1266	C70	I11	Post-op CD	Control group
0.9262013729977117
1267	C70	I13	Post-op CD	Control group
0.8308543096872616
1268	C70	I15	Post-op CD	Control group
0.9500381388253242
1269	C70	I17	Post-op CD	Control group
0.7833714721586575
1270	C70	I18	Post-op CD	Control group
0.8220823798627003
1271	C70	I19	Post-op CD	Control group
0.4687261632341724
1272	C70	I22	Post-op CD	Control group
0.8686117467581999
1273	C70	I23	Post-op CD	Control group
0.9071319603356217
1274	C70	I24	Post-op CD	Control group
0.7852784134248665
1275	C70	I25	Post-op CD	Control group
0.8522120518688024
1276	C70	I26	Post-op CD	Control group
0.9176201372997712
1277	C70	I27	Post-op CD	Control group
0.86441647597254
1278	C70	I28	Post-op CD	Control group
0.8703279938977879
1279	C70	I29	Post-op CD	Control group
0.9098016781083142
1280	C70	I30	Post-op CD	Control group
0.9427917620137299
1281	C70	I31	Post-op CD	Control group
0.9324942791762014
1282	C70	I32	Post-op CD	Control group
0.9277269260106789
1283	C70	I33	Post-op CD	Control group
0.7374141876430206
1284	C70	I34	Post-op CD	Control group
0.8407704042715485
1285	C70	I35	Post-op CD	Control group
0.8792906178489702
1286	C70	I36	Post-op CD	Control group
0.9326849733028223
1287	C70	I37	Post-op CD	Control group

0.9136155606407322
1288	C74	B27	Post-op CD	Control group
0.8413424866514111
1289	C74	B66	Post-op CD	Control group
0.9509916094584286
1290	C74	B86	Post-op CD	Control group
0.8081617086193745
1291	C74	B97	Post-op CD	Control group
0.916094584286804
1292	C74	B98	Post-op CD	Control group
0.927536231884058
1293	C74	B100	Post-op CD	Control group
0.9727307398932112
1294	C74	B112	Post-op CD	Control group
0.8178871090770404
1295	C74	B115	Post-op CD	Control group
0.9227688787185355
1296	C74	I1	Post-op CD	Control group
0.9059877955758963
1297	C74	I3	Post-op CD	Control group
0.9380244088482075
1298	C74	I6	Post-op CD	Control group
0.9061784897025171
1299	C74	I8	Post-op CD	Control group
0.9029366895499619
1300	C74	I10	Post-op CD	Control group
0.9242944317315027
1301	C74	I11	Post-op CD	Control group
0.9315408085430968
1302	C74	I13	Post-op CD	Control group
0.8852021357742181
1303	C74	I15	Post-op CD	Control group
0.8485888634630053
1304	C74	I17	Post-op CD	Control group
0.841723874904653
1305	C74	I18	Post-op CD	Control group
0.8539282990083905
1306	C74	I19	Post-op CD	Control group
0.6254767353165522
1307	C74	I22	Post-op CD	Control group
0.7965293668954996
1308	C74	I23	Post-op CD	Control group
0.9172387490465294
1309	C74	I24	Post-op CD	Control group
0.8360030511060259
1310	C74	I25	Post-op CD	Control group
0.8981693363844394
1311	C74	I26	Post-op CD	Control group
0.9357360793287567
1312	C74	I27	Post-op CD	Control group
0.9170480549199085
1313	C74	I28	Post-op CD	Control group
0.9241037376048817
1314	C74	I29	Post-op CD	Control group

0.9302059496567505
1315 C74 I30 Post-op CD Control group
0.9534706331045004
1316 C74 I31 Post-op CD Control group
0.9418382913806255
1317 C74 I32 Post-op CD Control group
0.9557589626239512
1318 C74 I33 Post-op CD Control group
0.7999618611746758
1319 C74 I34 Post-op CD Control group
0.8499237223493517
1320 C74 I35 Post-op CD Control group
0.9347826086956522
1321 C74 I36 Post-op CD Control group
0.8390541571319603
1322 C74 I37 Post-op CD Control group
0.7993897787948131
1323 C78 B27 Post-op CD Control group
0.9193363844393593
1324 C78 B66 Post-op CD Control group
0.7057589626239512
1325 C78 B86 Post-op CD Control group
0.8064454614797865
1326 C78 B97 Post-op CD Control group
0.7503813882532419
1327 C78 B98 Post-op CD Control group
0.8506864988558352
1328 C78 B100 Post-op CD Control group
0.9658657513348589
1329 C78 B112 Post-op CD Control group
0.8934019832189168
1330 C78 B115 Post-op CD Control group
0.8483981693363845
1331 C78 I1 Post-op CD Control group
0.877765064836003
1332 C78 I3 Post-op CD Control group
0.7797482837528604
1333 C78 I6 Post-op CD Control group
0.8356216628527842
1334 C78 I8 Post-op CD Control group
0.8972158657513348
1335 C78 I10 Post-op CD Control group
0.7675438596491229
1336 C78 I11 Post-op CD Control group
0.9355453852021358
1337 C78 I13 Post-op CD Control group
0.8422959572845157
1338 C78 I15 Post-op CD Control group
0.7473302822273074
1339 C78 I17 Post-op CD Control group
0.9149504195270786
1340 C78 I18 Post-op CD Control group
0.8136918382913806
1341 C78 I19 Post-op CD Control group

0.9094202898550725
1342 C78 I22 Post-op CD Control group
0.8901601830663616
1343 C78 I23 Post-op CD Control group
0.825896262395118З
1344 C78 I24 Post-op CD Control group
0.8705186880244088
1345 C78 I25 Post-op CD Control group
0.7887109077040427
1346 C78 I26 Post-op CD Control group
0.7604881769641495
1347 C78 I27 Post-op CD Control group
0.6611365369946606
1348 C78 I28 Post-op CD Control group
0.8239893211289092
1349 C78 I29 Post-op CD Control group
0.8409610983981693
1350 C78 I30 Post-op CD Control group
0.7803203661327232
1351 C78 I31 Post-op CD Control group
0.9174294431731502
1352 C78 I32 Post-op CD Control group
0.8306636155606407
1353 C78 I33 Post-op CD Control group
0.811022120518688
1354 C78 I34 Post-op CD Control group
0.8281845919145691
1355 C78 I35 Post-op CD Control group
0.8030129672006102
1356 C78 I36 Post-op CD Control group
0.9963768115942029
1357 C78 I37 Post-op CD Control group
0.9469870327993898
1358 C1 B1 Post-op CD Idiopathic BAD
0.830282227307399
1359 C1 B5 Post-op CD Idiopathic BAD
0.8716628527841342
1360 C1 B6 Post-op CD Idiopathic BAD
0.9860793287566743
1361 C1 B10 Post-op CD Idiopathic BAD
0.8096872616323417
1362 C1 B17 Post-op CD Idiopathic BAD
0.9525171624713958
1363 C1 B20 Post-op CD Idiopathic BAD
0.9759725400457666
1364 C1 B23 Post-op CD Idiopathic BAD
0.9454614797864226
1365 C1 B31 Post-op CD Idiopathic BAD
0.7240655987795576
1366 C1 B35 Post-op CD Idiopathic BAD
0.7738367658276125
1367 C1 B39 Post-op CD Idiopathic BAD
0.8838672768878718
1368 C1 B43 Post-op CD Idiopathic BAD

0.984744469870328
1369 C1 B47 Post-op CD Idiopathic BAD
0.9481311975591151
1370 C1 B48 Post-op CD Idiopathic BAD
0.8283752860411899
1371 C1 B49 Post-op CD Idiopathic BAD
0.9584286803966438
1372 C1 B53 Post-op CD Idiopathic BAD
0.9439359267734554
1373 C1 B54 Post-op CD Idiopathic BAD
0.967391304347826
1374 C1 B55 Post-op CD Idiopathic BAD
0.9938977879481312
1375 C1 B59 Post-op CD Idiopathic BAD
0.8316170861937452
1376 C1 B70 Post-op CD Idiopathic BAD
0.9746376811594203
1377 C1 B74 Post-op CD Idiopathic BAD
0.9773073989321129
1378 C1 B77 Post-op CD Idiopathic BAD
0.952326468344775
1379 C1 B81 Post-op CD Idiopathic BAD
0.8596491228070176
1380 C1 B84 Post-op CD Idiopathic BAD
0.9105644546147978
1381 C1 B89 Post-op CD Idiopathic BAD
0.9717772692601068
1382 C1 B92 Post-op CD Idiopathic BAD
0.9355453852021358
1383 C1 B95 Post-op CD Idiopathic BAD
0.9374523264683448
1384 C1 B99 Post-op CD Idiopathic BAD
0.9698703279938978
1385 C1 B103 Post-op CD Idiopathic BAD
0.9643401983218917
1386 C1 B106 Post-op CD Idiopathic BAD
0.9830282227307399
1387 C1 B109 Post-op CD Idiopathic BAD
0.9433638443935927
1388 C1 B118 Post-op CD Idiopathic BAD
0.9794050343249427
1389 C1 B119 Post-op CD Idiopathic BAD
0.9464149504195271
1390 C3 B1 Post-op CD Idiopathic BAD
0.8155987795575896
1391 C3 B5 Post-op CD Idiopathic BAD
0.8996948893974066
1392 C3 B6 Post-op CD Idiopathic BAD
0.9212433257055682
1393 C3 B10 Post-op CD Idiopathic BAD
0.8173150266971777
1394 C3 B17 Post-op CD Idiopathic BAD
0.9233409610983981
1395 C3 B20 Post-op CD Idiopathic BAD

0.9456521739130435
1396 C3 B23 Post-op CD Idiopathic BAD
0.8503051106025934
1397 C3 B31 Post-op CD Idiopathic BAD
0.8144546147978642
1398 C3 B35 Post-op CD Idiopathic BAD
0.9761632341723875
1399 C3 B39 Post-op CD Idiopathic BAD
0.833905415713196
1400 C3 B43 Post-op CD Idiopathic BAD
0.9467963386727689
1401 C3 B47 Post-op CD Idiopathic BAD
0.9340198321891686
1402 C3 B48 Post-op CD Idiopathic BAD
0.971395881006865
1403 C3 B49 Post-op CD Idiopathic BAD
0.935163996948894
1404 C3 B53 Post-op CD Idiopathic BAD
0.9155225019069413
1405 C3 B54 Post-op CD Idiopathic BAD
0.9565217391304348
1406 C3 B55 Post-op CD Idiopathic BAD
0.9532799389778794
1407 C3 B59 Post-op CD Idiopathic BAD
0.8089244851258581
1408 C3 B70 Post-op CD Idiopathic BAD
0.9631960335621663
1409 C3 B74 Post-op CD Idiopathic BAD
0.7889016018306636
1410 C3 B77 Post-op CD Idiopathic BAD
0.9014111365369947
1411 C3 B81 Post-op CD Idiopathic BAD
0.9565217391304348
1412 C3 B84 Post-op CD Idiopathic BAD
0.8859649122807017
1413 C3 B89 Post-op CD Idiopathic BAD
0.9477498093058734
1414 C3 B92 Post-op CD Idiopathic BAD
0.877765064836003
1415 C3 B95 Post-op CD Idiopathic BAD
0.9586193745232647
1416 C3 B99 Post-op CD Idiopathic BAD
0.9439359267734554
1417 C3 B103 Post-op CD Idiopathic BAD
0.9816933638443935
1418 C3 B106 Post-op CD Idiopathic BAD
0.9723493516399695
1419 C3 B109 Post-op CD Idiopathic BAD
0.9677726926010679
1420 C3 B118 Post-op CD Idiopathic BAD
0.9452707856598017
1421 C3 B119 Post-op CD Idiopathic BAD
0.9286803966437833
1422 C7 B1 Post-op CD Idiopathic BAD

0.9414569031273837
1423	C7	B5	Post-op CD	Idiopathic BAD
0.8785278413424866
1424	C7	B6	Post-op CD	Idiopathic BAD
0.9603356216628528
1425	C7	B10	Post-op CD	Idiopathic BAD
0.9906559877955758
1426	C7	B17	Post-op CD	Idiopathic BAD
0.6624713958810069
1427	C7	B20	Post-op CD	Idiopathic BAD
0.5922959572845157
1428	C7	B23	Post-op CD	Idiopathic BAD
0.8758581235697941
1429	C7	B31	Post-op CD	Idiopathic BAD
0.950419527078566
1430	C7	B35	Post-op CD	Idiopathic BAD
0.9630053394355453
1431	C7	B39	Post-op CD	Idiopathic BAD
0.9443173150266971
1432	C7	B43	Post-op CD	Idiopathic BAD
0.748093058733791
1433	C7	B47	Post-op CD	Idiopathic BAD
0.8918764302059496
1434	C7	B48	Post-op CD	Idiopathic BAD
0.9464149504195271
1435	C7	B49	Post-op CD	Idiopathic BAD
0.9815026697177727
1436	C7	B53	Post-op CD	Idiopathic BAD
0.8947368421052632
1437	C7	B54	Post-op CD	Idiopathic BAD
0.6437833714721587
1438	C7	B55	Post-op CD	Idiopathic BAD
0.5671243325705568
1439	C7	B59	Post-op CD	Idiopathic BAD
0.9900839054157132
1440	C7	B70	Post-op CD	Idiopathic BAD
0.915903890160183
1441	C7	B74	Post-op CD	Idiopathic BAD
0.9487032799389779
1442	C7	B77	Post-op CD	Idiopathic BAD
0.9061784897025171
1443	C7	B81	Post-op CD	Idiopathic BAD
0.9385964912280702
1444	C7	B84	Post-op CD	Idiopathic BAD
0.8956903127383676
1445	C7	B89	Post-op CD	Idiopathic BAD
0.910373760488177
1446	C7	B92	Post-op CD	Idiopathic BAD
0.5797101449275363
1447	C7	B95	Post-op CD	Idiopathic BAD
0.7210144927536232
1448	C7	B99	Post-op CD	Idiopathic BAD
0.9016018306636155
1449	C7	B103	Post-op CD	Idiopathic BAD

0.912090007627765
1450	C7	B106	Post-op CD	Idiopathic BAD
0.5240274599542334
1451	C7	B109	Post-op CD	Idiopathic BAD
0.9088482074752098
1452	C7	B118	Post-op CD	Idiopathic BAD
0.6462623951182304
1453	C7	B119	Post-op CD	Idiopathic BAD
0.9042715484363082
1454	C8	B1	Post-op CD	Idiopathic BAD
0.9084668192219679
1455	C8	B5	Post-op CD	Idiopathic BAD
0.9429824561403509
1456	C8	B6	Post-op CD	Idiopathic BAD
0.8878718535469108
1457	C8	B10	Post-op CD	Idiopathic BAD
0.8996948893974066
1458	C8	B17	Post-op CD	Idiopathic BAD
0.9252479023646072
1459	C8	B20	Post-op CD	Idiopathic BAD
0.9740655987795576
1460	C8	B23	Post-op CD	Idiopathic BAD
0.8827231121281465
1461	C8	B31	Post-op CD	Idiopathic BAD
0.9052250190694127
1462	C8	B35	Post-op CD	Idiopathic BAD
0.9557589626239512
1463	C8	B39	Post-op CD	Idiopathic BAD
0.8836765827612509
1464	C8	B43	Post-op CD	Idiopathic BAD
0.9759725400457666
1465	C8	B47	Post-op CD	Idiopathic BAD
0.9414569031273837
1466	C8	B48	Post-op CD	Idiopathic BAD
0.9464149504195271
1467	C8	B49	Post-op CD	Idiopathic BAD
0.965675057208238
1468	C8	B53	Post-op CD	Idiopathic BAD
0.9719679633867276
1469	C8	B54	Post-op CD	Idiopathic BAD
0.9778794813119756
1470	C8	B55	Post-op CD	Idiopathic BAD
0.9645308924485125
1471	C8	B59	Post-op CD	Idiopathic BAD
0.9225781845919145
1472	C8	B70	Post-op CD	Idiopathic BAD
0.9666285278413425
1473	C8	B74	Post-op CD	Idiopathic BAD
0.7236842105263158
1474	C8	B77	Post-op CD	Idiopathic BAD
0.938977879481312
1475	C8	B81	Post-op CD	Idiopathic BAD
0.9195270785659801
1476	C8	B84	Post-op CD	Idiopathic BAD

0.9214340198321892
1477 C8 B89 Post-op CD Idiopathic BAD
0.9519450800915332
1478 C8 B92 Post-op CD Idiopathic BAD
0.908276125095347
1479 C8 B95 Post-op CD Idiopathic BAD
0.9836003051106026
1480 C8 B99 Post-op CD Idiopathic BAD
0.9557589626239512
1481 C8 B103 Post-op CD Idiopathic BAD
0.9437452326468345
1482 C8 B106 Post-op CD Idiopathic BAD
0.9780701754385965
1483 C8 B109 Post-op CD Idiopathic BAD
0.9647215865751335
1484 C8 B118 Post-op CD Idiopathic BAD
0.9811212814645309
1485 C8 B119 Post-op CD Idiopathic BAD
0.8688024408848207
1486 C11 B1 Post-op CD Idiopathic BAD
0.9231502669717773
1487 C11 B5 Post-op CD Idiopathic BAD
0.8638443935926774
1488 C11 B6 Post-op CD Idiopathic BAD
0.954233409610984
1489 C11 B10 Post-op CD Idiopathic BAD
0.9950419527078566
1490 C11 B17 Post-op CD Idiopathic BAD
0.8890160183066361
1491 C11 B20 Post-op CD Idiopathic BAD
0.9229595728451564
1492 C11 B23 Post-op CD Idiopathic BAD
0.7210144927536232
1493 C11 B31 Post-op CD Idiopathic BAD
0.8838672768878718
1494 C11 B35 Post-op CD Idiopathic BAD
0.8525934401220442
1495 C11 B39 Post-op CD Idiopathic BAD
0.9050343249427918
1496 C11 B43 Post-op CD Idiopathic BAD
0.9668192219679634
1497 C11 B47 Post-op CD Idiopathic BAD
0.8918764302059496
1498 C11 B48 Post-op CD Idiopathic BAD
0.9016018306636155
1499 C11 B49 Post-op CD Idiopathic BAD
0.915903890160183
1500 C11 B53 Post-op CD Idiopathic BAD
0.795766590389016
1501 C11 B54 Post-op CD Idiopathic BAD
0.9355453852021358
1502 C11 B55 Post-op CD Idiopathic BAD
0.8581235697940504
1503 C11 B59 Post-op CD Idiopathic BAD

0.9910373760488177
1504 C11 B70 Post-op CD Idiopathic BAD
0.7082379862700229
1505 C11 B74 Post-op CD Idiopathic BAD
0.9460335621662853
1506 C11 B77 Post-op CD Idiopathic BAD
0.8516399694889397
1507 C11 B81 Post-op CD Idiopathic BAD
0.8653699466056446
1508 C11 B84 Post-op CD Idiopathic BAD
0.88558352402746
1509 C11 B89 Post-op CD Idiopathic BAD
0.8954996186117468
1510 C11 B92 Post-op CD Idiopathic BAD
0.8691838291380626
1511 C11 B95 Post-op CD Idiopathic BAD
0.9479405034324943
1512 C11 B99 Post-op CD Idiopathic BAD
0.86441647597254
1513 C11 B103 Post-op CD Idiopathic BAD
0.7734553775743707
1514 C11 B106 Post-op CD Idiopathic BAD
0.9849351639969489
1515 C11 B109 Post-op CD Idiopathic BAD
0.7869946605644547
1516 C11 B118 Post-op CD Idiopathic BAD
0.9376430205949656
1517 C11 B119 Post-op CD Idiopathic BAD
0.7572463768115942
1518 C15 B1 Post-op CD Idiopathic BAD
0.9050343249427918
1519 C15 B5 Post-op CD Idiopathic BAD
0.7692601067887109
1520 C15 B6 Post-op CD Idiopathic BAD
0.9241037376048817
1521 C15 B10 Post-op CD Idiopathic BAD
0.977116704805492
1522 C15 B17 Post-op CD Idiopathic BAD
0.9239130434782609
1523 C15 B20 Post-op CD Idiopathic BAD
0.9023646071700991
1524 C15 B23 Post-op CD Idiopathic BAD
0.7456140350877193
1525 C15 B31 Post-op CD Idiopathic BAD
0.952326468344775
1526 C15 B35 Post-op CD Idiopathic BAD
0.8573607932875668
1527 C15 B39 Post-op CD Idiopathic BAD
0.9016018306636155
1528 C15 B43 Post-op CD Idiopathic BAD
0.9626239511823036
1529 C15 B47 Post-op CD Idiopathic BAD
0.8056826849733029
1530 C15 B48 Post-op CD Idiopathic BAD

0.8138825324180016
1531 C15 B49 Post-op CD Idiopathic BAD
0.8922578184591915
1532 C15 B53 Post-op CD Idiopathic BAD
0.8028222730739893
1533 C15 B54 Post-op CD Idiopathic BAD
0.9122807017543859
1534 C15 B55 Post-op CD Idiopathic BAD
0.8899694889397407
1535 C15 B59 Post-op CD Idiopathic BAD
0.9456521739130435
1536 C15 B70 Post-op CD Idiopathic BAD
0.6361556064073226
1537 C15 B74 Post-op CD Idiopathic BAD
0.9452707856598017
1538 C15 B77 Post-op CD Idiopathic BAD
0.8173150266971777
1539 C15 B81 Post-op CD Idiopathic BAD
0.9479405034324943
1540 C15 B84 Post-op CD Idiopathic BAD
0.8188405797101449
1541 C15 B89 Post-op CD Idiopathic BAD
0.8375286041189931
1542 C15 B92 Post-op CD Idiopathic BAD
0.8508771929824561
1543 C15 B95 Post-op CD Idiopathic BAD
0.864607170099161
1544 C15 B99 Post-op CD Idiopathic BAD
0.8026315789473685
1545 C15 B103 Post-op CD Idiopathic BAD
0.9576659038901602
1546 C15 B106 Post-op CD Idiopathic BAD
0.9754004576659039
1547 C15 B109 Post-op CD Idiopathic BAD
0.8415331807780321
1548 C15 B118 Post-op CD Idiopathic BAD
0.9303966437833715
1549 C15 B119 Post-op CD Idiopathic BAD
0.7183447749809306
1550 C19 B1 Post-op CD Idiopathic BAD
0.9269641495041953
1551 C19 B5 Post-op CD Idiopathic BAD
0.8680396643783371
1552 C19 B6 Post-op CD Idiopathic BAD
0.9145690312738368
1553 C19 B10 Post-op CD Idiopathic BAD
0.9788329519450801
1554 C19 B17 Post-op CD Idiopathic BAD
0.6289092295957285
1555 C19 B20 Post-op CD Idiopathic BAD
0.7091914569031273
1556 C19 B23 Post-op CD Idiopathic BAD
0.719488939740656
1557 C19 B31 Post-op CD Idiopathic BAD

0.912090007627765
1558 C19 B35 Post-op CD Idiopathic BAD
0.9765446224256293
1559 C19 B39 Post-op CD Idiopathic BAD
0.8937833714721587
1560 C19 B43 Post-op CD Idiopathic BAD
0.8276125095347063
1561 C19 B47 Post-op CD Idiopathic BAD
0.8556445461479787
1562 C19 B48 Post-op CD Idiopathic BAD
0.9862700228832952
1563 C19 B49 Post-op CD Idiopathic BAD
0.9376430205949656
1564 C19 B53 Post-op CD Idiopathic BAD
0.7627765064836003
1565 C19 B54 Post-op CD Idiopathic BAD
0.5606407322654462
1566 C19 B55 Post-op CD Idiopathic BAD
0.4744469870327994
1567 C19 B59 Post-op CD Idiopathic BAD
0.9832189168573608
1568 C19 B70 Post-op CD Idiopathic BAD
0.7200610221205187
1569 C19 B74 Post-op CD Idiopathic BAD
0.9191456903127384
1570 C19 B77 Post-op CD Idiopathic BAD
0.8237986270022883
1571 C19 B81 Post-op CD Idiopathic BAD
0.9078947368421053
1572 C19 B84 Post-op CD Idiopathic BAD
0.8676582761250954
1573 C19 B89 Post-op CD Idiopathic BAD
0.8741418764302059
1574 C19 B92 Post-op CD Idiopathic BAD
0.6401601830663616
1575 C19 B95 Post-op CD Idiopathic BAD
0.8442028985507246
1576 C19 B99 Post-op CD Idiopathic BAD
0.8230358504958047
1577 C19 B103 Post-op CD Idiopathic BAD
0.8098779557589626
1578 C19 B106 Post-op CD Idiopathic BAD
0.5909610983981693
1579 C19 B109 Post-op CD Idiopathic BAD
0.8272311212814645
1580 C19 B118 Post-op CD Idiopathic BAD
0.6485507246376812
1581 C19 B119 Post-op CD Idiopathic BAD
0.7587719298245614
1582 C22 B1 Post-op CD Idiopathic BAD
0.8607932875667429
1583 C22 B5 Post-op CD Idiopathic BAD
0.8832951945080092
1584 C22 B6 Post-op CD Idiopathic BAD

0.9576659038901602
1585	C22	B10	Post-op CD	Idiopathic BAD
0.6836384439359268
1586	C22	B17	Post-op CD	Idiopathic BAD
0.9130434782608695
1587	C22	B20	Post-op CD	Idiopathic BAD
0.9433638443935927
1588	C22	B23	Post-op CD	Idiopathic BAD
0.9454614797864226
1589	C22	B31	Post-op CD	Idiopathic BAD
0.8285659801678108
1590	C22	B35	Post-op CD	Idiopathic BAD
0.9738749046529367
1591	C22	B39	Post-op CD	Idiopathic BAD
0.9012204424103738
1592	C22	B43	Post-op CD	Idiopathic BAD
0.9578565980167811
1593	C22	B47	Post-op CD	Idiopathic BAD
0.9467963386727689
1594	C22	B48	Post-op CD	Idiopathic BAD
0.9628146453089245
1595	C22	B49	Post-op CD	Idiopathic BAD
0.96186117467582
1596	C22	B53	Post-op CD	Idiopathic BAD
0.96186117467582
1597	C22	B54	Post-op CD	Idiopathic BAD
0.9729214340198322
1598	C22	B55	Post-op CD	Idiopathic BAD
0.973302822273074
1599	C22	B59	Post-op CD	Idiopathic BAD
0.7492372234935164
1600	C22	B70	Post-op CD	Idiopathic BAD
0.9689168573607932
1601	C22	B74	Post-op CD	Idiopathic BAD
0.8937833714721587
1602	C22	B77	Post-op CD	Idiopathic BAD
0.8625095347063311
1603	C22	B81	Post-op CD	Idiopathic BAD
0.9666285278413425
1604	C22	B84	Post-op CD	Idiopathic BAD
0.9056064073226545
1605	C22	B89	Post-op CD	Idiopathic BAD
0.9746376811594203
1606	C22	B92	Post-op CD	Idiopathic BAD
0.9269641495041953
1607	C22	B95	Post-op CD	Idiopathic BAD
0.9778794813119756
1608	C22	B99	Post-op CD	Idiopathic BAD
0.9391685736079328
1609	C22	B103	Post-op CD	Idiopathic BAD
0.9696796338672768
1610	C22	B106	Post-op CD	Idiopathic BAD
0.9803585049580473
1611	C22	B109	Post-op CD	Idiopathic BAD

0.9692982456140351
1612 C22 B118 Post-op CD Idiopathic BAD
0.9698703279938978
1613 C22 B119 Post-op CD Idiopathic BAD
0.9620518688024409
1614 C26 B1 Post-op CD Idiopathic BAD
0.900839054157132
1615 C26 B5 Post-op CD Idiopathic BAD
0.7179633867276888
1616 C26 B6 Post-op CD Idiopathic BAD
0.9155225019069413
1617 C26 B10 Post-op CD Idiopathic BAD
0.9893211289092296
1618 C26 B17 Post-op CD Idiopathic BAD
0.938977879481312
1619 C26 B20 Post-op CD Idiopathic BAD
0.9662471395881007
1620 C26 B23 Post-op CD Idiopathic BAD
0.8714721586575134
1621 C26 B31 Post-op CD Idiopathic BAD
0.9258199847444699
1622 C26 B35 Post-op CD Idiopathic BAD
0.6992753623188406
1623 C26 B39 Post-op CD Idiopathic BAD
0.9023646071700991
1624 C26 B43 Post-op CD Idiopathic BAD
0.975209763539283
1625 C26 B47 Post-op CD Idiopathic BAD
0.9050343249427918
1626 C26 B48 Post-op CD Idiopathic BAD
0.7301678108314263
1627 C26 B49 Post-op CD Idiopathic BAD
0.8352402745995423
1628 C26 B53 Post-op CD Idiopathic BAD
0.9551868802440885
1629 C26 B54 Post-op CD Idiopathic BAD
0.9300152555301296
1630 C26 B55 Post-op CD Idiopathic BAD
0.9616704805491991
1631 C26 B59 Post-op CD Idiopathic BAD
0.9897025171624714
1632 C26 B70 Post-op CD Idiopathic BAD
0.9010297482837528
1633 C26 B74 Post-op CD Idiopathic BAD
0.9487032799389779
1634 C26 B77 Post-op CD Idiopathic BAD
0.898741418764302
1635 C26 B81 Post-op CD Idiopathic BAD
0.9344012204424104
1636 C26 B84 Post-op CD Idiopathic BAD
0.9061784897025171
1637 C26 B89 Post-op CD Idiopathic BAD
0.858886346300534
1638 C26 B92 Post-op CD Idiopathic BAD

0.8598398169336384
1639 C26 B95 Post-op CD Idiopathic BAD
0.8709000762776506
1640 C26 B99 Post-op CD Idiopathic BAD
0.8741418764302059
1641 C26 B103 Post-op CD Idiopathic BAD
0.9553775743707094
1642 C26 B106 Post-op CD Idiopathic BAD
0.9851258581235698
1643 C26 B109 Post-op CD Idiopathic BAD
0.9511823035850496
1644 C26 B118 Post-op CD Idiopathic BAD
0.9654843630816171
1645 C26 B119 Post-op CD Idiopathic BAD
0.851067887109077
1646 C28 B1 Post-op CD Idiopathic BAD
0.9134248665141114
1647 C28 B5 Post-op CD Idiopathic BAD
0.8655606407322655
1648 C28 B6 Post-op CD Idiopathic BAD
0.8975972540045767
1649 C28 B10 Post-op CD Idiopathic BAD
0.9799771167048055
1650 C28 B17 Post-op CD Idiopathic BAD
0.9094202898550725
1651 C28 B20 Post-op CD Idiopathic BAD
0.9406941266209001
1652 C28 B23 Post-op CD Idiopathic BAD
0.7540045766590389
1653 C28 B31 Post-op CD Idiopathic BAD
0.9525171624713958
1654 C28 B35 Post-op CD Idiopathic BAD
0.8848207475209764
1655 C28 B39 Post-op CD Idiopathic BAD
0.8888253241800153
1656 C28 B43 Post-op CD Idiopathic BAD
0.9221967963386728
1657 C28 B47 Post-op CD Idiopathic BAD
0.8630816170861938
1658 C28 B48 Post-op CD Idiopathic BAD
0.9122807017543859
1659 C28 B49 Post-op CD Idiopathic BAD
0.9307780320366132
1660 C28 B53 Post-op CD Idiopathic BAD
0.7185354691075515
1661 C28 B54 Post-op CD Idiopathic BAD
0.9212433257055682
1662 C28 B55 Post-op CD Idiopathic BAD
0.8972158657513348
1663 C28 B59 Post-op CD Idiopathic BAD
0.9849351639969489
1664 C28 B70 Post-op CD Idiopathic BAD
0.7170099160945843
1665 C28 B74 Post-op CD Idiopathic BAD

0.944698703279939
1666 C28 B77 Post-op CD Idiopathic BAD
0.8331426392067124
1667 C28 B81 Post-op CD Idiopathic BAD
0.9281083142639207
1668 C28 B84 Post-op CD Idiopathic BAD
0.8236079328756675
1669 C28 B89 Post-op CD Idiopathic BAD
0.9218154080854309
1670 C28 B92 Post-op CD Idiopathic BAD
0.8581235697940504
1671 C28 B95 Post-op CD Idiopathic BAD
0.9530892448512586
1672 C28 B99 Post-op CD Idiopathic BAD
0.8495423340961098
1673 C28 B103 Post-op CD Idiopathic BAD
0.8306636155606407
1674 C28 B106 Post-op CD Idiopathic BAD
0.9782608695652174
1675 C28 B109 Post-op CD Idiopathic BAD
0.7419908466819222
1676 C28 B118 Post-op CD Idiopathic BAD
0.9395499618611747
1677 C28 B119 Post-op CD Idiopathic BAD
0.7229214340198322
1678 C31 B1 Post-op CD Idiopathic BAD
0.9797864225781846
1679 C31 B5 Post-op CD Idiopathic BAD
0.8571700991609459
1680 C31 B6 Post-op CD Idiopathic BAD
0.9622425629290617
1681 C31 B10 Post-op CD Idiopathic BAD
0.9836003051106026
1682 C31 B17 Post-op CD Idiopathic BAD
0.9681540808543097
1683 C31 B20 Post-op CD Idiopathic BAD
0.9853165522501907
1684 C31 B23 Post-op CD Idiopathic BAD
0.9422196796338673
1685 C31 B31 Post-op CD Idiopathic BAD
0.9652936689549961
1686 C31 B35 Post-op CD Idiopathic BAD
0.8461098398169337
1687 C31 B39 Post-op CD Idiopathic BAD
0.9191456903127384
1688 C31 B43 Post-op CD Idiopathic BAD
0.9834096109839817
1689 C31 B47 Post-op CD Idiopathic BAD
0.9536613272311213
1690 C31 B48 Post-op CD Idiopathic BAD
0.8146453089244852
1691 C31 B49 Post-op CD Idiopathic BAD
0.8266590389016019
1692 C31 B53 Post-op CD Idiopathic BAD

0.969488939740656
1693	C31	B54	Post-op CD	Idiopathic BAD
0.9185736079328757
1694	C31	B55	Post-op CD	Idiopathic BAD
0.9959954233409611
1695	C31	B59	Post-op CD	Idiopathic BAD
0.9839816933638444
1696	C31	B70	Post-op CD	Idiopathic BAD
0.9063691838291381
1697	C31	B74	Post-op CD	Idiopathic BAD
0.9944698703279939
1698	C31	B77	Post-op CD	Idiopathic BAD
0.9044622425629291
1699	C31	B81	Post-op CD	Idiopathic BAD
0.9837909992372235
1700	C31	B84	Post-op CD	Idiopathic BAD
0.9616704805491991
1701	C31	B89	Post-op CD	Idiopathic BAD
0.9851258581235698
1702	C31	B92	Post-op CD	Idiopathic BAD
0.9385964912280702
1703	C31	B95	Post-op CD	Idiopathic BAD
0.9805491990846682
1704	C31	B99	Post-op CD	Idiopathic BAD
0.86441647597254
1705	C31	B103	Post-op CD	Idiopathic BAD
0.9273455377574371
1706	C31	B106	Post-op CD	Idiopathic BAD
0.9937070938215103
1707	C31	B109	Post-op CD	Idiopathic BAD
0.9239130434782609
1708	C31	B118	Post-op CD	Idiopathic BAD
0.9877955758962624
1709	C31	B119	Post-op CD	Idiopathic BAD
0.9105644546147978
1710	C35	B1	Post-op CD	Idiopathic BAD
0.9622425629290617
1711	C35	B5	Post-op CD	Idiopathic BAD
0.9086575133485889
1712	C35	B6	Post-op CD	Idiopathic BAD
0.9071319603356217
1713	C35	B10	Post-op CD	Idiopathic BAD
0.9918001525553013
1714	C35	B17	Post-op CD	Idiopathic BAD
0.8030129672006102
1715	C35	B20	Post-op CD	Idiopathic BAD
0.721205186880244
1716	C35	B23	Post-op CD	Idiopathic BAD
0.7334096109839817
1717	C35	B31	Post-op CD	Idiopathic BAD
0.9528985507246377
1718	C35	B35	Post-op CD	Idiopathic BAD
0.9769260106788711
1719	C35	B39	Post-op CD	Idiopathic BAD

0.9311594202898551
1720	C35	B43	Post-op CD	Idiopathic BAD
0.7656369183829138
1721	C35	B47	Post-op CD	Idiopathic BAD
0.8716628527841342
1722	C35	B48	Post-op CD	Idiopathic BAD
0.9683447749809306
1723	C35	B49	Post-op CD	Idiopathic BAD
0.9185736079328757
1724	C35	B53	Post-op CD	Idiopathic BAD
0.7984363081617086
1725	C35	B54	Post-op CD	Idiopathic BAD
0.8394355453852022
1726	C35	B55	Post-op CD	Idiopathic BAD
0.8222730739893211
1727	C35	B59	Post-op CD	Idiopathic BAD
0.9849351639969489
1728	C35	B70	Post-op CD	Idiopathic BAD	0.75
1729	C35	B74	Post-op CD	Idiopathic BAD
0.9427917620137299
1730	C35	B77	Post-op CD	Idiopathic BAD
0.8072082379862701
1731	C35	B81	Post-op CD	Idiopathic BAD
0.9628146453089245
1732	C35	B84	Post-op CD	Idiopathic BAD
0.8173150266971777
1733	C35	B89	Post-op CD	Idiopathic BAD
0.9397406559877955
1734	C35	B92	Post-op CD	Idiopathic BAD
0.6838291380625476
1735	C35	B95	Post-op CD	Idiopathic BAD
0.7387490465293669
1736	C35	B99	Post-op CD	Idiopathic BAD
0.8661327231121282
1737	C35	B103	Post-op CD	Idiopathic BAD
0.6842105263157895
1738	C35	B106	Post-op CD	Idiopathic BAD
0.9040808543096872
1739	C35	B109	Post-op CD	Idiopathic BAD
0.8535469107551488
1740	C35	B118	Post-op CD	Idiopathic BAD
0.8468726163234173
1741	C35	B119	Post-op CD	Idiopathic BAD
0.7587719298245614
1742	C38	B1	Post-op CD	Idiopathic BAD
0.9567124332570557
1743	C38	B5	Post-op CD	Idiopathic BAD
0.9551868802440885
1744	C38	B6	Post-op CD	Idiopathic BAD
0.9862700228832952
1745	C38	B10	Post-op CD	Idiopathic BAD
0.9475591151792525
1746	C38	B17	Post-op CD	Idiopathic BAD
0.9786422578184591

1747	C38	B20	Post-op CD	Idiopathic BAD	0.9774980930587338
1748	C38	B23	Post-op CD	Idiopathic BAD	0.9782608695652174
1749	C38	B31	Post-op CD	Idiopathic BAD	0.9570938215102975
1750	C38	B35	Post-op CD	Idiopathic BAD	0.9380244088482075
1751	C38	B39	Post-op CD	Idiopathic BAD	0.9528985507246377
1752	C38	B43	Post-op CD	Idiopathic BAD	0.9876048817696415
1753	C38	B47	Post-op CD	Idiopathic BAD	0.9692982456140351
1754	C38	B48	Post-op CD	Idiopathic BAD	0.8199847444698704
1755	C38	B49	Post-op CD	Idiopathic BAD	0.9803585049580473
1756	C38	B53	Post-op CD	Idiopathic BAD	0.9620518688024409
1757	C38	B54	Post-op CD	Idiopathic BAD	0.982837528604119
1758	C38	B55	Post-op CD	Idiopathic BAD	0.9938977879481312
1759	C38	B59	Post-op CD	Idiopathic BAD	0.9338291380625476
1760	C38	B70	Post-op CD	Idiopathic BAD	0.977116704805492
1761	C38	B74	Post-op CD	Idiopathic BAD	0.9860793287566743
1762	C38	B77	Post-op CD	Idiopathic BAD	0.9405034324942791
1763	C38	B81	Post-op CD	Idiopathic BAD	0.9448893974065599
1764	C38	B84	Post-op CD	Idiopathic BAD	0.9549961861174676
1765	C38	B89	Post-op CD	Idiopathic BAD	0.9807398932112891
1766	C38	B92	Post-op CD	Idiopathic BAD	0.9786422578184591
1767	C38	B95	Post-op CD	Idiopathic BAD	0.9839816933638444
1768	C38	B99	Post-op CD	Idiopathic BAD	0.9631960335621663
1769	C38	B103	Post-op CD	Idiopathic BAD	0.9824561403508771
1770	C38	B106	Post-op CD	Idiopathic BAD	0.9916094584286804
1771	C38	B109	Post-op CD	Idiopathic BAD	0.9940884820747521
1772	C38	B118	Post-op CD	Idiopathic BAD	0.9900839054157132
1773	C38	B119	Post-op CD	Idiopathic BAD	0.9225781845919145

1774	C40	B1	Post-op CD	Idiopathic BAD
0.8670861937452327
1775	C40	B5	Post-op CD	Idiopathic BAD
0.7816552250190694
1776	C40	B6	Post-op CD	Idiopathic BAD
0.9029366895499619
1777	C40	B10	Post-op CD	Idiopathic BAD
0.9530892448512586
1778	C40	B17	Post-op CD	Idiopathic BAD
0.9473684210526315
1779	C40	B20	Post-op CD	Idiopathic BAD
0.9364988558352403
1780	C40	B23	Post-op CD	Idiopathic BAD
0.9549961861174676
1781	C40	B31	Post-op CD	Idiopathic BAD
0.9242944317315027
1782	C40	B35	Post-op CD	Idiopathic BAD
0.6332951945080092
1783	C40	B39	Post-op CD	Idiopathic BAD
0.9023646071700991
1784	C40	B43	Post-op CD	Idiopathic BAD
0.9231502669717773
1785	C40	B47	Post-op CD	Idiopathic BAD
0.9315408085430968
1786	C40	B48	Post-op CD	Idiopathic BAD
0.669717772692601
1787	C40	B49	Post-op CD	Idiopathic BAD
0.8480167810831426
1788	C40	B53	Post-op CD	Idiopathic BAD
0.9288710907704043
1789	C40	B54	Post-op CD	Idiopathic BAD
0.9685354691075515
1790	C40	B55	Post-op CD	Idiopathic BAD
0.9879862700228833
1791	C40	B59	Post-op CD	Idiopathic BAD
0.9765446224256293
1792	C40	B70	Post-op CD	Idiopathic BAD
0.9098016781083142
1793	C40	B74	Post-op CD	Idiopathic BAD
0.9429824561403509
1794	C40	B77	Post-op CD	Idiopathic BAD
0.9326849733028223
1795	C40	B81	Post-op CD	Idiopathic BAD
0.8287566742944318
1796	C40	B84	Post-op CD	Idiopathic BAD
0.9305873379099924
1797	C40	B89	Post-op CD	Idiopathic BAD
0.8878718535469108
1798	C40	B92	Post-op CD	Idiopathic BAD
0.9300152555301296
1799	C40	B95	Post-op CD	Idiopathic BAD
0.847254004576659
1800	C40	B99	Post-op CD	Idiopathic BAD
0.881769641495042

1801 C40 B103 Post-op CD Idiopathic BAD
0.919908466819222
1802 C40 B106 Post-op CD Idiopathic BAD
0.9773073989321129
1803 C40 B109 Post-op CD Idiopathic BAD
0.9019832189168574
1804 C40 B118 Post-op CD Idiopathic BAD
0.9624332570556827
1805 C40 B119 Post-op CD Idiopathic BAD
0.8257055682684973
1806 C44 B1 Post-op CD Idiopathic BAD
0.8895881006864989
1807 C44 B5 Post-op CD Idiopathic BAD
0.8728070175438597
1808 C44 B6 Post-op CD Idiopathic BAD
0.9773073989321129
1809 C44 B10 Post-op CD Idiopathic BAD
0.9834096109839817
1810 C44 B17 Post-op CD Idiopathic BAD
0.9021739130434783
1811 C44 B20 Post-op CD Idiopathic BAD
0.9218154080854309
1812 C44 B23 Post-op CD Idiopathic BAD
0.7679252479023646
1813 C44 B31 Post-op CD Idiopathic BAD
0.9418382913806255
1814 C44 B35 Post-op CD Idiopathic BAD
0.950228832951945
1815 C44 B39 Post-op CD Idiopathic BAD
0.9439359267734554
1816 C44 B43 Post-op CD Idiopathic BAD
0.9809305873379099
1817 C44 B47 Post-op CD Idiopathic BAD
0.8869183829138062
1818 C44 B48 Post-op CD Idiopathic BAD
0.937070938215103
1819 C44 B49 Post-op CD Idiopathic BAD
0.9626239511823036
1820 C44 B53 Post-op CD Idiopathic BAD
0.7782227307398932
1821 C44 B54 Post-op CD Idiopathic BAD
0.9063691838291381
1822 C44 B55 Post-op CD Idiopathic BAD
0.9021739130434783
1823 C44 B59 Post-op CD Idiopathic BAD
0.9816933638443935
1824 C44 B70 Post-op CD Idiopathic BAD
0.6632341723874905
1825 C44 B74 Post-op CD Idiopathic BAD
0.9509916094584286
1826 C44 B77 Post-op CD Idiopathic BAD
0.8352402745995423
1827 C44 B81 Post-op CD Idiopathic BAD
0.8752860411899314

1828 C44 B84 Post-op CD Idiopathic BAD
0.8832951945080092
1829 C44 B89 Post-op CD Idiopathic BAD
0.9164759725400458
1830 C44 B92 Post-op CD Idiopathic BAD
0.8897787948131197
1831 C44 B95 Post-op CD Idiopathic BAD
0.937070938215103
1832 C44 B99 Post-op CD Idiopathic BAD
0.8794813119755912
1833 C44 B103 Post-op CD Idiopathic BAD
0.8676582761250954
1834 C44 B106 Post-op CD Idiopathic BAD
0.9795957284515637
1835 C44 B109 Post-op CD Idiopathic BAD
0.7915713196033562
1836 C44 B118 Post-op CD Idiopathic BAD
0.9437452326468345
1837 C44 B119 Post-op CD Idiopathic BAD
0.7292143401983219
1838 C47 B1 Post-op CD Idiopathic BAD
0.931350114416476
1839 C47 B5 Post-op CD Idiopathic BAD
0.9052250190694127
1840 C47 B6 Post-op CD Idiopathic BAD
0.979023646071701
1841 C47 B10 Post-op CD Idiopathic BAD
0.9889397406559878
1842 C47 B17 Post-op CD Idiopathic BAD
0.910373760488177
1843 C47 B20 Post-op CD Idiopathic BAD
0.9342105263157895
1844 C47 B23 Post-op CD Idiopathic BAD
0.7879481311975591
1845 C47 B31 Post-op CD Idiopathic BAD
0.9929443173150267
1846 C47 B35 Post-op CD Idiopathic BAD
0.9406941266209001
1847 C47 B39 Post-op CD Idiopathic BAD
0.9271548436308161
1848 C47 B43 Post-op CD Idiopathic BAD
0.9546147978642258
1849 C47 B47 Post-op CD Idiopathic BAD
0.8663234172387491
1850 C47 B48 Post-op CD Idiopathic BAD
0.9832189168573608
1851 C47 B49 Post-op CD Idiopathic BAD
0.9740655987795576
1852 C47 B53 Post-op CD Idiopathic BAD
0.7669717772692601
1853 C47 B54 Post-op CD Idiopathic BAD
0.9374523264683448
1854 C47 B55 Post-op CD Idiopathic BAD
0.8859649122807017

1855	C47	B59	Post-op CD	Idiopathic BAD	0.9839816933638444
1856	C47	B70	Post-op CD	Idiopathic BAD	0.7011823035850496
1857	C47	B74	Post-op CD	Idiopathic BAD	0.948512585812357
1858	C47	B77	Post-op CD	Idiopathic BAD	0.8609839816933639
1859	C47	B81	Post-op CD	Idiopathic BAD	0.9490846681922197
1860	C47	B84	Post-op CD	Idiopathic BAD	0.8914950419527079
1861	C47	B89	Post-op CD	Idiopathic BAD	0.9643401983218917
1862	C47	B92	Post-op CD	Idiopathic BAD	0.9016018306636155
1863	C47	B95	Post-op CD	Idiopathic BAD	0.971395881006865
1864	C47	B99	Post-op CD	Idiopathic BAD	0.877765064836003
1865	C47	B103	Post-op CD	Idiopathic BAD	0.811022120518688
1866	C47	B106	Post-op CD	Idiopathic BAD	0.9672006102212052
1867	C47	B109	Post-op CD	Idiopathic BAD	0.8565980167810832
1868	C47	B118	Post-op CD	Idiopathic BAD	0.9481311975591151
1869	C47	B119	Post-op CD	Idiopathic BAD	0.8175057208237986
1870	C48	B1	Post-op CD	Idiopathic BAD	0.9324942791762014
1871	C48	B5	Post-op CD	Idiopathic BAD	0.8632723112128147
1872	C48	B6	Post-op CD	Idiopathic BAD	0.9269641495041953
1873	C48	B10	Post-op CD	Idiopathic BAD	0.92372234935164
1874	C48	B17	Post-op CD	Idiopathic BAD	0.700419527078566
1875	C48	B20	Post-op CD	Idiopathic BAD	0.6338672768878718
1876	C48	B23	Post-op CD	Idiopathic BAD	0.8447749809305873
1877	C48	B31	Post-op CD	Idiopathic BAD	0.9105644546147978
1878	C48	B35	Post-op CD	Idiopathic BAD	0.9822654462242563
1879	C48	B39	Post-op CD	Idiopathic BAD	0.8968344774980931
1880	C48	B43	Post-op CD	Idiopathic BAD	0.6956521739130435
1881	C48	B47	Post-op CD	Idiopathic BAD	0.8360030511060259

1882	C48	B48	Post-op CD	Idiopathic BAD	0.9750190694126621
1883	C48	B49	Post-op CD	Idiopathic BAD	0.8831045003813882
1884	C48	B53	Post-op CD	Idiopathic BAD	0.8617467581998475
1885	C48	B54	Post-op CD	Idiopathic BAD	0.6969870327993898
1886	C48	B55	Post-op CD	Idiopathic BAD	0.7248283752860412
1887	C48	B59	Post-op CD	Idiopathic BAD	0.9521357742181541
1888	C48	B70	Post-op CD	Idiopathic BAD	0.9157131960335622
1889	C48	B74	Post-op CD	Idiopathic BAD	0.9010297482837528
1890	C48	B77	Post-op CD	Idiopathic BAD	0.8180778032036613
1891	C48	B81	Post-op CD	Idiopathic BAD	0.8802440884820748
1892	C48	B84	Post-op CD	Idiopathic BAD	0.8810068649885584
1893	C48	B89	Post-op CD	Idiopathic BAD	0.9157131960335622
1894	C48	B92	Post-op CD	Idiopathic BAD	0.5968726163234173
1895	C48	B95	Post-op CD	Idiopathic BAD	0.7358886346300534
1896	C48	B99	Post-op CD	Idiopathic BAD	0.8583142639206712
1897	C48	B103	Post-op CD	Idiopathic BAD	0.9385964912280702
1898	C48	B106	Post-op CD	Idiopathic BAD	0.7093821510297483
1899	C48	B109	Post-op CD	Idiopathic BAD	0.8953089244851259
1900	C48	B118	Post-op CD	Idiopathic BAD	0.6887871853546911
1901	C48	B119	Post-op CD	Idiopathic BAD	0.8768115942028986
1902	C49	B1	Post-op CD	Idiopathic BAD	0.8724256292906178
1903	C49	B5	Post-op CD	Idiopathic BAD	0.8640350877192983
1904	C49	B6	Post-op CD	Idiopathic BAD	0.9183829138062548
1905	C49	B10	Post-op CD	Idiopathic BAD	0.9303966437833715
1906	C49	B17	Post-op CD	Idiopathic BAD	0.8548817696414951
1907	C49	B20	Post-op CD	Idiopathic BAD	0.8415331807780321
1908	C49	B23	Post-op CD	Idiopathic BAD	0.935163996948894

1909	C49	B31	Post-op CD	Idiopathic BAD	0.9403127383676583
1910	C49	B35	Post-op CD	Idiopathic BAD	0.7440884820747521
1911	C49	B39	Post-op CD	Idiopathic BAD	0.9088482074752098
1912	C49	B43	Post-op CD	Idiopathic BAD	0.820747520976354
1913	C49	B47	Post-op CD	Idiopathic BAD	0.9174294431731502
1914	C49	B48	Post-op CD	Idiopathic BAD	0.7913806254767353
1915	C49	B49	Post-op CD	Idiopathic BAD	0.7942410373760488
1916	C49	B53	Post-op CD	Idiopathic BAD	0.9113272311212814
1917	C49	B54	Post-op CD	Idiopathic BAD	0.864607170099161
1918	C49	B55	Post-op CD	Idiopathic BAD	0.8657513348588863
1919	C49	B59	Post-op CD	Idiopathic BAD	0.9366895499618612
1920	C49	B70	Post-op CD	Idiopathic BAD	0.9414569031273837
1921	C49	B74	Post-op CD	Idiopathic BAD	0.9445080091533181
1922	C49	B77	Post-op CD	Idiopathic BAD	0.8852021357742181
1923	C49	B81	Post-op CD	Idiopathic BAD	0.9218154080854309
1924	C49	B84	Post-op CD	Idiopathic BAD	0.916094584286804
1925	C49	B89	Post-op CD	Idiopathic BAD	0.8924485125858124
1926	C49	B92	Post-op CD	Idiopathic BAD	0.818649885583524
1927	C49	B95	Post-op CD	Idiopathic BAD	0.801487414187643
1928	C49	B99	Post-op CD	Idiopathic BAD	0.8688024408848207
1929	C49	B103	Post-op CD	Idiopathic BAD	0.9677726926010679
1930	C49	B106	Post-op CD	Idiopathic BAD	0.8367658276125095
1931	C49	B109	Post-op CD	Idiopathic BAD	0.9420289855072463
1932	C49	B118	Post-op CD	Idiopathic BAD	0.8470633104500381
1933	C49	B119	Post-op CD	Idiopathic BAD	0.9164759725400458
1934	C53	B1	Post-op CD	Idiopathic BAD	0.9521357742181541
1935	C53	B5	Post-op CD	Idiopathic BAD	0.8956903127383676

1936	C53	B6	Post-op CD	Idiopathic BAD	0.9321128909229596
1937	C53	B10	Post-op CD	Idiopathic BAD	0.9546147978642258
1938	C53	B17	Post-op CD	Idiopathic BAD	0.7448512585812357
1939	C53	B20	Post-op CD	Idiopathic BAD	0.7776506483600305
1940	C53	B23	Post-op CD	Idiopathic BAD	0.7782227307398932
1941	C53	B31	Post-op CD	Idiopathic BAD	0.9517543859649122
1942	C53	B35	Post-op CD	Idiopathic BAD	0.9363081617086194
1943	C53	B39	Post-op CD	Idiopathic BAD	0.9328756674294432
1944	C53	B43	Post-op CD	Idiopathic BAD	0.7965293668954996
1945	C53	B47	Post-op CD	Idiopathic BAD	0.84744469870328
1946	C53	B48	Post-op CD	Idiopathic BAD	0.9757818459191457
1947	C53	B49	Post-op CD	Idiopathic BAD	0.8909229595728452
1948	C53	B53	Post-op CD	Idiopathic BAD	0.7721205186880244
1949	C53	B54	Post-op CD	Idiopathic BAD	0.7757437070938215
1950	C53	B55	Post-op CD	Idiopathic BAD	0.7494279176201373
1951	C53	B59	Post-op CD	Idiopathic BAD	0.9643401983218917
1952	C53	B70	Post-op CD	Idiopathic BAD	0.8011060259344012
1953	C53	B74	Post-op CD	Idiopathic BAD	0.9189549961861174
1954	C53	B77	Post-op CD	Idiopathic BAD	0.7742181540808543
1955	C53	B81	Post-op CD	Idiopathic BAD	0.9214340198321892
1956	C53	B84	Post-op CD	Idiopathic BAD	0.8466819221967964
1957	C53	B89	Post-op CD	Idiopathic BAD	0.9256292906178489
1958	C53	B92	Post-op CD	Idiopathic BAD	0.740465293668955
1959	C53	B95	Post-op CD	Idiopathic BAD	0.837909992372235
1960	C53	B99	Post-op CD	Idiopathic BAD	0.8861556064073226
1961	C53	B103	Post-op CD	Idiopathic BAD	0.7606788710907704
1962	C53	B106	Post-op CD	Idiopathic BAD	0.8041571319603357

1963 C53 B109 Post-op CD Idiopathic BAD
0.8569794050343249
1964 C53 B118 Post-op CD Idiopathic BAD
0.8114035087719298
1965 C53 B119 Post-op CD Idiopathic BAD
0.7990083905415714
1966 C56 B1 Post-op CD Idiopathic BAD
0.9078947368421053
1967 C56 B5 Post-op CD Idiopathic BAD
0.770976353928299
1968 C56 B6 Post-op CD Idiopathic BAD
0.8819603356216629
1969 C56 B10 Post-op CD Idiopathic BAD
0.9204805491990846
1970 C56 B17 Post-op CD Idiopathic BAD
0.7496186117467581
1971 C56 B20 Post-op CD Idiopathic BAD
0.7358886346300534
1972 C56 B23 Post-op CD Idiopathic BAD
0.7307398932112891
1973 C56 B31 Post-op CD Idiopathic BAD
0.9042715484363082
1974 C56 B35 Post-op CD Idiopathic BAD
0.9525171624713958
1975 C56 B39 Post-op CD Idiopathic BAD
0.864607170099161
1976 C56 B43 Post-op CD Idiopathic BAD
0.7549580472921434
1977 C56 B47 Post-op CD Idiopathic BAD
0.8011060259344012
1978 C56 B48 Post-op CD Idiopathic BAD
0.8775743707093822
1979 C56 B49 Post-op CD Idiopathic BAD
0.8714721586575134
1980 C56 B53 Post-op CD Idiopathic BAD
0.7398932112890922
1981 C56 B54 Post-op CD Idiopathic BAD
0.7753623188405797
1982 C56 B55 Post-op CD Idiopathic BAD
0.7336003051106026
1983 C56 B59 Post-op CD Idiopathic BAD
0.9530892448512586
1984 C56 B70 Post-op CD Idiopathic BAD
0.7524790236460717
1985 C56 B74 Post-op CD Idiopathic BAD
0.8928299008390541
1986 C56 B77 Post-op CD Idiopathic BAD
0.7416094584286804
1987 C56 B81 Post-op CD Idiopathic BAD
0.864607170099161
1988 C56 B84 Post-op CD Idiopathic BAD
0.8218916857360793
1989 C56 B89 Post-op CD Idiopathic BAD
0.8729977116704806

1990	C56	B92	Post-op CD	Idiopathic BAD
0.719488939740656				
1991	C56	B95	Post-op CD	Idiopathic BAD
0.7763157894736842				
1992	C56	B99	Post-op CD	Idiopathic BAD
0.7898550724637681				
1993	C56	B103	Post-op CD	Idiopathic BAD
0.9267734553775744				
1994	C56	B106	Post-op CD	Idiopathic BAD
0.7721205186880244				
1995	C56	B109	Post-op CD	Idiopathic BAD
0.7753623188405797				
1996	C56	B118	Post-op CD	Idiopathic BAD
0.7753623188405797				
1997	C56	B119	Post-op CD	Idiopathic BAD
0.6857360793287567				
1998	C60	B1	Post-op CD	Idiopathic BAD
0.805301296720061				
1999	C60	B5	Post-op CD	Idiopathic BAD
0.8220823798627003				
2000	C60	B6	Post-op CD	Idiopathic BAD
0.908276125095347				
2001	C60	B10	Post-op CD	Idiopathic BAD
0.7927154843630816				
2002	C60	B17	Post-op CD	Idiopathic BAD
0.7433257055682685				
2003	C60	B20	Post-op CD	Idiopathic BAD
0.7848970251716247				
2004	C60	B23	Post-op CD	Idiopathic BAD
0.7326468344774981				
2005	C60	B31	Post-op CD	Idiopathic BAD
0.8005339435545386				
2006	C60	B35	Post-op CD	Idiopathic BAD
0.9170480549199085				
2007	C60	B39	Post-op CD	Idiopathic BAD
0.8686117467581999				
2008	C60	B43	Post-op CD	Idiopathic BAD
0.8049199084668193				
2009	C60	B47	Post-op CD	Idiopathic BAD
0.7950038138825324				
2010	C60	B48	Post-op CD	Idiopathic BAD
0.8981693363844394				
2011	C60	B49	Post-op CD	Idiopathic BAD
0.9092295957284515				
2012	C60	B53	Post-op CD	Idiopathic BAD
0.6966056445461479				
2013	C60	B54	Post-op CD	Idiopathic BAD
0.7831807780320366				
2014	C60	B55	Post-op CD	Idiopathic BAD
0.7583905415713196				
2015	C60	B59	Post-op CD	Idiopathic BAD
0.8525934401220442				
2016	C60	B70	Post-op CD	Idiopathic BAD
0.7265446224256293				

2017	C60	B74	Post-op CD	Idiopathic BAD	0.8579328756674295
2018	C60	B77	Post-op CD	Idiopathic BAD	0.7692601067887109
2019	C60	B81	Post-op CD	Idiopathic BAD	0.9134248665141114
2020	C60	B84	Post-op CD	Idiopathic BAD	0.7660183066361556
2021	C60	B89	Post-op CD	Idiopathic BAD	0.9410755148741419
2022	C60	B92	Post-op CD	Idiopathic BAD	0.7475209763539283
2023	C60	B95	Post-op CD	Idiopathic BAD	0.8333333333333334
2024	C60	B99	Post-op CD	Idiopathic BAD	0.8602212051868803
2025	C60	B103	Post-op CD	Idiopathic BAD	0.9450800915331807
2026	C60	B106	Post-op CD	Idiopathic BAD	0.822463768115942
2027	C60	B109	Post-op CD	Idiopathic BAD	0.8459191456903128
2028	C60	B118	Post-op CD	Idiopathic BAD	0.8089244851258581
2029	C60	B119	Post-op CD	Idiopathic BAD	0.7845156369183829
2030	C62	B1	Post-op CD	Idiopathic BAD	0.9401220442410374
2031	C62	B5	Post-op CD	Idiopathic BAD	0.8346681922196796
2032	C62	B6	Post-op CD	Idiopathic BAD	0.9162852784134249
2033	C62	B10	Post-op CD	Idiopathic BAD	0.9429824561403509
2034	C62	B17	Post-op CD	Idiopathic BAD	0.831998474446987
2035	C62	B20	Post-op CD	Idiopathic BAD	0.8068268497330282
2036	C62	B23	Post-op CD	Idiopathic BAD	0.9061784897025171
2037	C62	B31	Post-op CD	Idiopathic BAD	0.9563310450038138
2038	C62	B35	Post-op CD	Idiopathic BAD	0.9265827612509535
2039	C62	B39	Post-op CD	Idiopathic BAD	0.8770022883295194
2040	C62	B43	Post-op CD	Idiopathic BAD	0.8102593440122045
2041	C62	B47	Post-op CD	Idiopathic BAD	0.8972158657513348
2042	C62	B48	Post-op CD	Idiopathic BAD	0.8872997711670481
2043	C62	B49	Post-op CD	Idiopathic BAD	0.8749046529366895

2044	C62	B53	Post-op CD	Idiopathic BAD
0.9174294431731502
2045	C62	B54	Post-op CD	Idiopathic BAD
0.8451563691838292
2046	C62	B55	Post-op CD	Idiopathic BAD
0.8482074752097636
2047	C62	B59	Post-op CD	Idiopathic BAD
0.9565217391304348
2048	C62	B70	Post-op CD	Idiopathic BAD
0.9016018306636155
2049	C62	B74	Post-op CD	Idiopathic BAD
0.9517543859649122
2050	C62	B77	Post-op CD	Idiopathic BAD
0.8432494279176201
2051	C62	B81	Post-op CD	Idiopathic BAD
0.9397406559877955
2052	C62	B84	Post-op CD	Idiopathic BAD
0.9040808543096872
2053	C62	B89	Post-op CD	Idiopathic BAD
0.9242944317315027
2054	C62	B92	Post-op CD	Idiopathic BAD
0.8041571319603357
2055	C62	B95	Post-op CD	Idiopathic BAD
0.8476353928299009
2056	C62	B99	Post-op CD	Idiopathic BAD
0.8272311212814645
2057	C62	B103	Post-op CD	Idiopathic BAD
0.8958810068649885
2058	C62	B106	Post-op CD	Idiopathic BAD
0.8390541571319603
2059	C62	B109	Post-op CD	Idiopathic BAD
0.8779557589626239
2060	C62	B118	Post-op CD	Idiopathic BAD
0.830091533180778
2061	C62	B119	Post-op CD	Idiopathic BAD
0.8737604881769642
2062	C64	B1	Post-op CD	Idiopathic BAD
0.9309687261632341
2063	C64	B5	Post-op CD	Idiopathic BAD
0.8707093821510298
2064	C64	B6	Post-op CD	Idiopathic BAD
0.9218154080854309
2065	C64	B10	Post-op CD	Idiopathic BAD
0.8779557589626239
2066	C64	B17	Post-op CD	Idiopathic BAD
0.7683066361556065
2067	C64	B20	Post-op CD	Idiopathic BAD
0.7908085430968727
2068	C64	B23	Post-op CD	Idiopathic BAD
0.833905415713196
2069	C64	B31	Post-op CD	Idiopathic BAD
0.90255530129672
2070	C64	B35	Post-op CD	Idiopathic BAD
0.9208619374523265

2071	C64	B39	Post-op CD	Idiopathic BAD
0.9077040427154843
2072	C64	B43	Post-op CD	Idiopathic BAD
0.7847063310450039
2073	C64	B47	Post-op CD	Idiopathic BAD
0.879862700228833
2074	C64	B48	Post-op CD	Idiopathic BAD
0.9263920671243325
2075	C64	B49	Post-op CD	Idiopathic BAD
0.8859649122807017
2076	C64	B53	Post-op CD	Idiopathic BAD
0.8520213577421816
2077	C64	B54	Post-op CD	Idiopathic BAD
0.7889016018306636
2078	C64	B55	Post-op CD	Idiopathic BAD
0.8312356979405034
2079	C64	B59	Post-op CD	Idiopathic BAD
0.9437452326468345
2080	C64	B70	Post-op CD	Idiopathic BAD
0.8670861937452327
2081	C64	B74	Post-op CD	Idiopathic BAD
0.8983600305110603
2082	C64	B77	Post-op CD	Idiopathic BAD
0.8346681922196796
2083	C64	B81	Post-op CD	Idiopathic BAD
0.9044622425629291
2084	C64	B84	Post-op CD	Idiopathic BAD
0.8775743707093822
2085	C64	B89	Post-op CD	Idiopathic BAD
0.9265827612509535
2086	C64	B92	Post-op CD	Idiopathic BAD
0.7610602593440122
2087	C64	B95	Post-op CD	Idiopathic BAD
0.8440122044241037
2088	C64	B99	Post-op CD	Idiopathic BAD
0.8865369946605645
2089	C64	B103	Post-op CD	Idiopathic BAD
0.9363081617086194
2090	C64	B106	Post-op CD	Idiopathic BAD
0.8070175438596491
2091	C64	B109	Post-op CD	Idiopathic BAD
0.9134248665141114
2092	C64	B118	Post-op CD	Idiopathic BAD
0.7948131197559115
2093	C64	B119	Post-op CD	Idiopathic BAD
0.9099923722349351
2094	C65	B1	Post-op CD	Idiopathic BAD
0.858886346300534
2095	C65	B5	Post-op CD	Idiopathic BAD
0.8518306636155606
2096	C65	B6	Post-op CD	Idiopathic BAD
0.9345919145690312
2097	C65	B10	Post-op CD	Idiopathic BAD
0.753813882532418

2098 C65 B17 Post-op CD Idiopathic BAD
0.7984363081617086
2099 C65 B20 Post-op CD Idiopathic BAD
0.7765064836003052
2100 C65 B23 Post-op CD Idiopathic BAD
0.9138062547673532
2101 C65 B31 Post-op CD Idiopathic BAD
0.8571700991609459
2102 C65 B35 Post-op CD Idiopathic BAD
0.9643401983218917
2103 C65 B39 Post-op CD Idiopathic BAD
0.9231502669717773
2104 C65 B43 Post-op CD Idiopathic BAD
0.7833714721586575
2105 C65 B47 Post-op CD Idiopathic BAD
0.8874904652936689
2106 C65 B48 Post-op CD Idiopathic BAD
0.9479405034324943
2107 C65 B49 Post-op CD Idiopathic BAD
0.8947368421052632
2108 C65 B53 Post-op CD Idiopathic BAD
0.8991228070175439
2109 C65 B54 Post-op CD Idiopathic BAD
0.8133104500381388
2110 C65 B55 Post-op CD Idiopathic BAD
0.8205568268497331
2111 C65 B59 Post-op CD Idiopathic BAD
0.7532418001525553
2112 C65 B70 Post-op CD Idiopathic BAD
0.9361174675819984
2113 C65 B74 Post-op CD Idiopathic BAD
0.9242944317315027
2114 C65 B77 Post-op CD Idiopathic BAD
0.8255148741418764
2115 C65 B81 Post-op CD Idiopathic BAD
0.9170480549199085
2116 C65 B84 Post-op CD Idiopathic BAD
0.8562166285278413
2117 C65 B89 Post-op CD Idiopathic BAD
0.9328756674294432
2118 C65 B92 Post-op CD Idiopathic BAD
0.7713577421815409
2119 C65 B95 Post-op CD Idiopathic BAD
0.8411517925247902
2120 C65 B99 Post-op CD Idiopathic BAD
0.8928299008390541
2121 C65 B103 Post-op CD Idiopathic BAD
0.9551868802440885
2122 C65 B106 Post-op CD Idiopathic BAD
0.7929061784897025
2123 C65 B109 Post-op CD Idiopathic BAD
0.9195270785659801
2124 C65 B118 Post-op CD Idiopathic BAD
0.8049199084668193

2125	C65	B119	Post-op CD	Idiopathic BAD
0.929252479023646
2126	C69	B1	Post-op CD	Idiopathic BAD
0.8115942028985508
2127	C69	B5	Post-op CD	Idiopathic BAD
0.7953852021357742
2128	C69	B6	Post-op CD	Idiopathic BAD
0.9069412662090007
2129	C69	B10	Post-op CD	Idiopathic BAD
0.9475591151792525
2130	C69	B17	Post-op CD	Idiopathic BAD
0.7658276125095347
2131	C69	B20	Post-op CD	Idiopathic BAD
0.7721205186880244
2132	C69	B23	Post-op CD	Idiopathic BAD
0.8731884057971014
2133	C69	B31	Post-op CD	Idiopathic BAD
0.9143783371472158
2134	C69	B35	Post-op CD	Idiopathic BAD
0.8554538520213577
2135	C69	B39	Post-op CD	Idiopathic BAD
0.9269641495041953
2136	C69	B43	Post-op CD	Idiopathic BAD
0.7822273073989321
2137	C69	B47	Post-op CD	Idiopathic BAD
0.8647978642257819
2138	C69	B48	Post-op CD	Idiopathic BAD
0.8241800152555301
2139	C69	B49	Post-op CD	Idiopathic BAD
0.7793668954996186
2140	C69	B53	Post-op CD	Idiopathic BAD
0.8371472158657514
2141	C69	B54	Post-op CD	Idiopathic BAD
0.7807017543859649
2142	C69	B55	Post-op CD	Idiopathic BAD
0.8213196033562167
2143	C69	B59	Post-op CD	Idiopathic BAD
0.9626239511823036
2144	C69	B70	Post-op CD	Idiopathic BAD
0.881578947368421
2145	C69	B74	Post-op CD	Idiopathic BAD
0.9405034324942791
2146	C69	B77	Post-op CD	Idiopathic BAD
0.86441647597254
2147	C69	B81	Post-op CD	Idiopathic BAD
0.8533562166285278
2148	C69	B84	Post-op CD	Idiopathic BAD
0.8956903127383676
2149	C69	B89	Post-op CD	Idiopathic BAD
0.8285659801678108
2150	C69	B92	Post-op CD	Idiopathic BAD
0.7086193745232647
2151	C69	B95	Post-op CD	Idiopathic BAD
0.761632341723875

2152	C69	B99	Post-op CD	Idiopathic BAD	0.7871853546910755
2153	C69	B103	Post-op CD	Idiopathic BAD	0.897025171624714
2154	C69	B106	Post-op CD	Idiopathic BAD	0.78813882532418
2155	C69	B109	Post-op CD	Idiopathic BAD	0.8560259344012204
2156	C69	B118	Post-op CD	Idiopathic BAD	0.7839435545385202
2157	C69	B119	Post-op CD	Idiopathic BAD	0.8527841342486652
2158	C70	B1	Post-op CD	Idiopathic BAD	0.9317315026697178
2159	C70	B5	Post-op CD	Idiopathic BAD	0.8890160183066361
2160	C70	B6	Post-op CD	Idiopathic BAD	0.8996948893974066
2161	C70	B10	Post-op CD	Idiopathic BAD	0.952326468344775
2162	C70	B17	Post-op CD	Idiopathic BAD	0.8135011441647597
2163	C70	B20	Post-op CD	Idiopathic BAD	0.7925247902364607
2164	C70	B23	Post-op CD	Idiopathic BAD	0.9250572082379863
2165	C70	B31	Post-op CD	Idiopathic BAD	0.9609077040427155
2166	C70	B35	Post-op CD	Idiopathic BAD	0.7902364607170099
2167	C70	B39	Post-op CD	Idiopathic BAD	0.9246758199847445
2168	C70	B43	Post-op CD	Idiopathic BAD	0.7848970251716247
2169	C70	B47	Post-op CD	Idiopathic BAD	0.8958810068649885
2170	C70	B48	Post-op CD	Idiopathic BAD	0.9326849733028223
2171	C70	B49	Post-op CD	Idiopathic BAD	0.885392829900839
2172	C70	B53	Post-op CD	Idiopathic BAD	0.9038901601830663
2173	C70	B54	Post-op CD	Idiopathic BAD	0.8201754385964912
2174	C70	B55	Post-op CD	Idiopathic BAD	0.8161708619374524
2175	C70	B59	Post-op CD	Idiopathic BAD	0.9639588100686499
2176	C70	B70	Post-op CD	Idiopathic BAD	0.9448893974065599
2177	C70	B74	Post-op CD	Idiopathic BAD	0.8979786422578184
2178	C70	B77	Post-op CD	Idiopathic BAD	0.8407704042715485

2179	C70	B81	Post-op CD	Idiopathic BAD
0.7738367658276125
2180	C70	B84	Post-op CD	Idiopathic BAD
0.9052250190694127
2181	C70	B89	Post-op CD	Idiopathic BAD
0.8884439359267735
2182	C70	B92	Post-op CD	Idiopathic BAD
0.7807017543859649
2183	C70	B95	Post-op CD	Idiopathic BAD
0.8228451563691839
2184	C70	B99	Post-op CD	Idiopathic BAD
0.8695652173913043
2185	C70	B103	Post-op CD	Idiopathic BAD
0.9132341723874905
2186	C70	B106	Post-op CD	Idiopathic BAD
0.7999618611746758
2187	C70	B109	Post-op CD	Idiopathic BAD
0.9260106788710908
2188	C70	B118	Post-op CD	Idiopathic BAD
0.8121662852784134
2189	C70	B119	Post-op CD	Idiopathic BAD
0.9059877955758963
2190	C74	B1	Post-op CD	Idiopathic BAD
0.8506864988558352
2191	C74	B5	Post-op CD	Idiopathic BAD
0.8714721586575134
2192	C74	B6	Post-op CD	Idiopathic BAD
0.9347826086956522
2193	C74	B10	Post-op CD	Idiopathic BAD
0.8012967200610221
2194	C74	B17	Post-op CD	Idiopathic BAD
0.8468726163234173
2195	C74	B20	Post-op CD	Idiopathic BAD
0.8211289092295957
2196	C74	B23	Post-op CD	Idiopathic BAD
0.933257055682685
2197	C74	B31	Post-op CD	Idiopathic BAD
0.8737604881769642
2198	C74	B35	Post-op CD	Idiopathic BAD
0.9935163996948894
2199	C74	B39	Post-op CD	Idiopathic BAD
0.9214340198321892
2200	C74	B43	Post-op CD	Idiopathic BAD
0.8199847444698704
2201	C74	B47	Post-op CD	Idiopathic BAD
0.9084668192219679
2202	C74	B48	Post-op CD	Idiopathic BAD
0.9723493516399695
2203	C74	B49	Post-op CD	Idiopathic BAD
0.9223874904652937
2204	C74	B53	Post-op CD	Idiopathic BAD
0.9262013729977117
2205	C74	B54	Post-op CD	Idiopathic BAD
0.849351639969488

2206	C74	B55	Post-op CD	Idiopathic BAD
0.8651792524790236
2207	C74	B59	Post-op CD	Idiopathic BAD
0.8150266971777269
2208	C74	B70	Post-op CD	Idiopathic BAD
0.9578565980167811
2209	C74	B74	Post-op CD	Idiopathic BAD
0.9435545385202135
2210	C74	B77	Post-op CD	Idiopathic BAD
0.9002669717772692
2211	C74	B81	Post-op CD	Idiopathic BAD
0.9374523264683448
2212	C74	B84	Post-op CD	Idiopathic BAD
0.8857742181540809
2213	C74	B89	Post-op CD	Idiopathic BAD
0.9458428680396643
2214	C74	B92	Post-op CD	Idiopathic BAD
0.8157894736842105
2215	C74	B95	Post-op CD	Idiopathic BAD
0.8745232646834478
2216	C74	B99	Post-op CD	Idiopathic BAD
0.9050343249427918
2217	C74	B103	Post-op CD	Idiopathic BAD
0.9727307398932112
2218	C74	B106	Post-op CD	Idiopathic BAD
0.8369565217391305
2219	C74	B109	Post-op CD	Idiopathic BAD
0.9420289855072463
2220	C74	B118	Post-op CD	Idiopathic BAD
0.8365751334858886
2221	C74	B119	Post-op CD	Idiopathic BAD
0.9321128909229596
2222	C78	B1	Post-op CD	Idiopathic BAD
0.8640350877192983
2223	C78	B5	Post-op CD	Idiopathic BAD
0.7822273073989321
2224	C78	B6	Post-op CD	Idiopathic BAD
0.9052250190694127
2225	C78	B10	Post-op CD	Idiopathic BAD
0.9672006102212052
2226	C78	B17	Post-op CD	Idiopathic BAD
0.8668954996186118
2227	C78	B20	Post-op CD	Idiopathic BAD
0.8565980167810832
2228	C78	B23	Post-op CD	Idiopathic BAD
0.7568649885583524
2229	C78	B31	Post-op CD	Idiopathic BAD
0.9057971014492754
2230	C78	B35	Post-op CD	Idiopathic BAD
0.839626239511823
2231	C78	B39	Post-op CD	Idiopathic BAD
0.8802440884820748
2232	C78	B43	Post-op CD	Idiopathic BAD
0.9134248665141114

2233	C78	B47	Post-op CD	Idiopathic BAD
0.8072082379862701
2234	C78	B48	Post-op CD	Idiopathic BAD
0.7980549199084668
2235	C78	B49	Post-op CD	Idiopathic BAD
0.765255530129672
2236	C78	B53	Post-op CD	Idiopathic BAD
0.8560259344012204
2237	C78	B54	Post-op CD	Idiopathic BAD
0.8916857360793288
2238	C78	B55	Post-op CD	Idiopathic BAD
0.8794813119755912
2239	C78	B59	Post-op CD	Idiopathic BAD
0.9830282227307399
2240	C78	B70	Post-op CD	Idiopathic BAD
0.8558352402745996
2241	C78	B74	Post-op CD	Idiopathic BAD
0.9174294431731502
2242	C78	B77	Post-op CD	Idiopathic BAD
0.8073989321128909
2243	C78	B81	Post-op CD	Idiopathic BAD
0.8670861937452327
2244	C78	B84	Post-op CD	Idiopathic BAD
0.8401983218916858
2245	C78	B89	Post-op CD	Idiopathic BAD
0.7698321891685737
2246	C78	B92	Post-op CD	Idiopathic BAD
0.8064454614797865
2247	C78	B95	Post-op CD	Idiopathic BAD
0.843440122044241
2248	C78	B99	Post-op CD	Idiopathic BAD
0.7044241037376049
2249	C78	B103	Post-op CD	Idiopathic BAD
0.8741418764302059
2250	C78	B106	Post-op CD	Idiopathic BAD
0.9513729977116705
2251	C78	B109	Post-op CD	Idiopathic BAD
0.7379862700228833
2252	C78	B118	Post-op CD	Idiopathic BAD
0.9098016781083142
2253	C78	B119	Post-op CD	Idiopathic BAD
0.7149122807017544
2254	C1	P1	Post-op CD	Post-cholecystectomy
0.8636536994660564
2255	C1	P2	Post-op CD	Post-cholecystectomy
0.9410755148741419
2256	C1	P4	Post-op CD	Post-cholecystectomy
0.9475591151792525
2257	C1	P5	Post-op CD	Post-cholecystectomy
0.9450800915331807
2258	C1	P9	Post-op CD	Post-cholecystectomy
0.9570938215102975
2259	C1	P13	Post-op CD	Post-cholecystectomy
0.9572845156369184

2260	C1	P15	Post-op CD	Post-cholecystectomy	0.9244851258581236
2261	C1	P16	Post-op CD	Post-cholecystectomy	0.9117086193745233
2262	C1	P17	Post-op CD	Post-cholecystectomy	0.9799771167048055
2263	C1	P20	Post-op CD	Post-cholecystectomy	0.8670861937452327
2264	C1	P21	Post-op CD	Post-cholecystectomy	0.8562166285278413
2265	C1	P24	Post-op CD	Post-cholecystectomy	0.9807398932112891
2266	C1	P26	Post-op CD	Post-cholecystectomy	0.8697559115179252
2267	C1	P30	Post-op CD	Post-cholecystectomy	0.9227688787185355
2268	C1	P33	Post-op CD	Post-cholecystectomy	0.9546147978642258
2269	C1	P35	Post-op CD	Post-cholecystectomy	0.9591914569031273
2270	C1	P38	Post-op CD	Post-cholecystectomy	0.9374523264683448
2271	C1	P39	Post-op CD	Post-cholecystectomy	0.8775743707093822
2272	C1	P42	Post-op CD	Post-cholecystectomy	0.9242944317315027
2273	C1	P43	Post-op CD	Post-cholecystectomy	0.9176201372997712
2274	C1	P46	Post-op CD	Post-cholecystectomy	0.9357360793287567
2275	C1	P47	Post-op CD	Post-cholecystectomy	0.9479405034324943
2276	C1	P50	Post-op CD	Post-cholecystectomy	0.9364988558352403
2277	C1	P55	Post-op CD	Post-cholecystectomy	0.9572845156369184
2278	C1	P58	Post-op CD	Post-cholecystectomy	0.9645308924485125
2279	C1	P60	Post-op CD	Post-cholecystectomy	0.9183829138062548
2280	C1	P63	Post-op CD	Post-cholecystectomy	0.9700610221205187
2281	C1	P65	Post-op CD	Post-cholecystectomy	0.9723493516399695
2282	C1	P68	Post-op CD	Post-cholecystectomy	0.9761632341723875
2283	C1	P70	Post-op CD	Post-cholecystectomy	0.9136155606407322
2284	C1	P71	Post-op CD	Post-cholecystectomy	0.9527078565980168
2285	C1	P74	Post-op CD	Post-cholecystectomy	0.9262013729977117
2286	C1	P75	Post-op CD	Post-cholecystectomy	0.9483218916857361

2287	C3	P1	Post-op CD	Post-cholecystectomy
0.784324942791762
2288	C3	P2	Post-op CD	Post-cholecystectomy
0.8808161708619374
2289	C3	P4	Post-op CD	Post-cholecystectomy
0.937070938215103
2290	C3	P5	Post-op CD	Post-cholecystectomy
0.9174294431731502
2291	C3	P9	Post-op CD	Post-cholecystectomy
0.9525171624713958
2292	C3	P13	Post-op CD	Post-cholecystectomy
0.9357360793287567
2293	C3	P15	Post-op CD	Post-cholecystectomy
0.851258581235698
2294	C3	P16	Post-op CD	Post-cholecystectomy
0.9277269260106789
2295	C3	P17	Post-op CD	Post-cholecystectomy
0.9183829138062548
2296	C3	P20	Post-op CD	Post-cholecystectomy
0.868230358504958
2297	C3	P21	Post-op CD	Post-cholecystectomy
0.9364988558352403
2298	C3	P24	Post-op CD	Post-cholecystectomy
0.9185736079328757
2299	C3	P26	Post-op CD	Post-cholecystectomy
0.9765446224256293
2300	C3	P30	Post-op CD	Post-cholecystectomy
0.9223874904652937
2301	C3	P33	Post-op CD	Post-cholecystectomy
0.9439359267734554
2302	C3	P35	Post-op CD	Post-cholecystectomy
0.9437452326468345
2303	C3	P38	Post-op CD	Post-cholecystectomy
0.9122807017543859
2304	C3	P39	Post-op CD	Post-cholecystectomy
0.9445080091533181
2305	C3	P42	Post-op CD	Post-cholecystectomy
0.9744469870327994
2306	C3	P43	Post-op CD	Post-cholecystectomy
0.9666285278413425
2307	C3	P46	Post-op CD	Post-cholecystectomy
0.9391685736079328
2308	C3	P47	Post-op CD	Post-cholecystectomy
0.9391685736079328
2309	C3	P50	Post-op CD	Post-cholecystectomy
0.8930205949656751
2310	C3	P55	Post-op CD	Post-cholecystectomy
0.898932112890923
2311	C3	P58	Post-op CD	Post-cholecystectomy
0.9452707856598017
2312	C3	P60	Post-op CD	Post-cholecystectomy
0.9620518688024409
2313	C3	P63	Post-op CD	Post-cholecystectomy
0.9260106788710908

2314	C3	P65	Post-op CD	Post-cholecystectomy
0.9506102212051869
2315	C3	P68	Post-op CD	Post-cholecystectomy
0.9628146453089245
2316	C3	P70	Post-op CD	Post-cholecystectomy
0.9569031273836766
2317	C3	P71	Post-op CD	Post-cholecystectomy
0.9601449275362319
2318	C3	P74	Post-op CD	Post-cholecystectomy
0.9300152555301296
2319	C3	P75	Post-op CD	Post-cholecystectomy
0.9263920671243325
2320	C7	P1	Post-op CD	Post-cholecystectomy
0.9837909992372235
2321	C7	P2	Post-op CD	Post-cholecystectomy
0.9157131960335622
2322	C7	P4	Post-op CD	Post-cholecystectomy
0.9252479023646072
2323	C7	P5	Post-op CD	Post-cholecystectomy
0.8583142639206712
2324	C7	P9	Post-op CD	Post-cholecystectomy
0.9054157131960335
2325	C7	P13	Post-op CD	Post-cholecystectomy
0.9155225019069413
2326	C7	P15	Post-op CD	Post-cholecystectomy
0.8304729214340199
2327	C7	P16	Post-op CD	Post-cholecystectomy
0.9090389016018307
2328	C7	P17	Post-op CD	Post-cholecystectomy
0.9069412662090007
2329	C7	P20	Post-op CD	Post-cholecystectomy
0.9303966437833715
2330	C7	P21	Post-op CD	Post-cholecystectomy
0.8792906178489702
2331	C7	P24	Post-op CD	Post-cholecystectomy
0.9641495041952708
2332	C7	P26	Post-op CD	Post-cholecystectomy
0.8872997711670481
2333	C7	P30	Post-op CD	Post-cholecystectomy
0.9795957284515637
2334	C7	P33	Post-op CD	Post-cholecystectomy
0.8880625476735317
2335	C7	P35	Post-op CD	Post-cholecystectomy
0.8968344774980931
2336	C7	P38	Post-op CD	Post-cholecystectomy
0.9815026697177727
2337	C7	P39	Post-op CD	Post-cholecystectomy
0.9317315026697178
2338	C7	P42	Post-op CD	Post-cholecystectomy
0.9265827612509535
2339	C7	P43	Post-op CD	Post-cholecystectomy
0.9378337147215866
2340	C7	P46	Post-op CD	Post-cholecystectomy
0.6977498093058734

2341	C7	P47	Post-op CD	Post-cholecystectomy	0.37090007627765065
2342	C7	P50	Post-op CD	Post-cholecystectomy	0.8583142639206712
2343	C7	P55	Post-op CD	Post-cholecystectomy	0.918001525553013
2344	C7	P58	Post-op CD	Post-cholecystectomy	0.9223874904652937
2345	C7	P60	Post-op CD	Post-cholecystectomy	0.9662471395881007
2346	C7	P63	Post-op CD	Post-cholecystectomy	0.9498474446987033
2347	C7	P65	Post-op CD	Post-cholecystectomy	0.8892067124332571
2348	C7	P68	Post-op CD	Post-cholecystectomy	0.9250572082379863
2349	C7	P70	Post-op CD	Post-cholecystectomy	0.876048817696415
2350	C7	P71	Post-op CD	Post-cholecystectomy	0.7475209763539283
2351	C7	P74	Post-op CD	Post-cholecystectomy	0.8686117467581999
2352	C7	P75	Post-op CD	Post-cholecystectomy	0.8703279938977879
2353	C8	P1	Post-op CD	Post-cholecystectomy	0.9279176201372997
2354	C8	P2	Post-op CD	Post-cholecystectomy	0.9210526315789473
2355	C8	P4	Post-op CD	Post-cholecystectomy	0.9248665141113653
2356	C8	P5	Post-op CD	Post-cholecystectomy	0.9279176201372997
2357	C8	P9	Post-op CD	Post-cholecystectomy	0.9595728451563692
2358	C8	P13	Post-op CD	Post-cholecystectomy	0.969488939740656
2359	C8	P15	Post-op CD	Post-cholecystectomy	0.876048817696415
2360	C8	P16	Post-op CD	Post-cholecystectomy	0.9246758199847445
2361	C8	P17	Post-op CD	Post-cholecystectomy	0.9662471395881007
2362	C8	P20	Post-op CD	Post-cholecystectomy	0.948512585812357
2363	C8	P21	Post-op CD	Post-cholecystectomy	0.9487032799389779
2364	C8	P24	Post-op CD	Post-cholecystectomy	0.9487032799389779
2365	C8	P26	Post-op CD	Post-cholecystectomy	0.9593821510297483
2366	C8	P30	Post-op CD	Post-cholecystectomy	0.9323035850495804
2367	C8	P33	Post-op CD	Post-cholecystectomy	0.9233409610983981

2368	C8	P35	Post-op CD	Post-cholecystectomy	0.9479405034324943
2369	C8	P38	Post-op CD	Post-cholecystectomy	0.936880244088482
2370	C8	P39	Post-op CD	Post-cholecystectomy	0.9170480549199085
2371	C8	P42	Post-op CD	Post-cholecystectomy	0.9555682684973302
2372	C8	P43	Post-op CD	Post-cholecystectomy	0.9660564454614798
2373	C8	P46	Post-op CD	Post-cholecystectomy	0.9494660564454614
2374	C8	P47	Post-op CD	Post-cholecystectomy	0.9551868802440885
2375	C8	P50	Post-op CD	Post-cholecystectomy	0.9279176201372997
2376	C8	P55	Post-op CD	Post-cholecystectomy	0.9248665141113653
2377	C8	P58	Post-op CD	Post-cholecystectomy	0.9492753623188406
2378	C8	P60	Post-op CD	Post-cholecystectomy	0.9603356216628528
2379	C8	P63	Post-op CD	Post-cholecystectomy	0.9506102212051869
2380	C8	P65	Post-op CD	Post-cholecystectomy	0.9448893974065599
2381	C8	P68	Post-op CD	Post-cholecystectomy	0.944698703279939
2382	C8	P70	Post-op CD	Post-cholecystectomy	0.915903890160183
2383	C8	P71	Post-op CD	Post-cholecystectomy	0.967581998474447
2384	C8	P74	Post-op CD	Post-cholecystectomy	0.9282990083905416
2385	C8	P75	Post-op CD	Post-cholecystectomy	0.9576659038901602
2386	C11	P1	Post-op CD	Post-cholecystectomy	0.9525171624713958
2387	C11	P2	Post-op CD	Post-cholecystectomy	0.8977879481311976
2388	C11	P4	Post-op CD	Post-cholecystectomy	0.9227688787185355
2389	C11	P5	Post-op CD	Post-cholecystectomy	0.8548817696414951
2390	C11	P9	Post-op CD	Post-cholecystectomy	0.7414187643020596
2391	C11	P13	Post-op CD	Post-cholecystectomy	0.9277269260106789
2392	C11	P15	Post-op CD	Post-cholecystectomy	0.947368421052631 5
2393	C11	P16	Post-op CD	Post-cholecystectomy	0.8762395118230358
2394	C11	P17	Post-op CD	Post-cholecystectomy	0.8194126620900076

2395	C11	P20	Post-op CD	Post-cholecystectomy
0.9078947368421053
2396	C11	P21	Post-op CD	Post-cholecystectomy
0.8058733790999237
2397	C11	P24	Post-op CD	Post-cholecystectomy
0.973302822273074
2398	C11	P26	Post-op CD	Post-cholecystectomy
0.6941266209000763
2399	C11	P30	Post-op CD	Post-cholecystectomy
0.9727307398932112
2400	C11	P33	Post-op CD	Post-cholecystectomy
0.8602212051868803
2401	C11	P35	Post-op CD	Post-cholecystectomy
0.7940503432494279
2402	C11	P38	Post-op CD	Post-cholecystectomy
0.8749046529366895
2403	C11	P39	Post-op CD	Post-cholecystectomy
0.9168573607932876
2404	C11	P42	Post-op CD	Post-cholecystectomy
0.5221205186880244
2405	C11	P43	Post-op CD	Post-cholecystectomy
0.8947368421052632
2406	C11	P46	Post-op CD	Post-cholecystectomy
0.8886346300533944
2407	C11	P47	Post-op CD	Post-cholecystectomy
0.9578565980167811
2408	C11	P50	Post-op CD	Post-cholecystectomy
0.8283752860411899
2409	C11	P55	Post-op CD	Post-cholecystectomy
0.7887109077040427
2410	C11	P58	Post-op CD	Post-cholecystectomy
0.7507627765064836
2411	C11	P60	Post-op CD	Post-cholecystectomy
0.9344012204424104
2412	C11	P63	Post-op CD	Post-cholecystectomy
0.9344012204424104
2413	C11	P65	Post-op CD	Post-cholecystectomy
0.8089244851258581
2414	C11	P68	Post-op CD	Post-cholecystectomy
0.59744469870328
2415	C11	P70	Post-op CD	Post-cholecystectomy
0.6657131960335622
2416	C11	P71	Post-op CD	Post-cholecystectomy
0.830282227307399
2417	C11	P74	Post-op CD	Post-cholecystectomy
0.872234935163997
2418	C11	P75	Post-op CD	Post-cholecystectomy
0.9221967963386728
2419	C15	P1	Post-op CD	Post-cholecystectomy
0.9677726926010679
2420	C15	P2	Post-op CD	Post-cholecystectomy
0.8424866514111365
2421	C15	P4	Post-op CD	Post-cholecystectomy
0.8996948893974066

2422	C15	P5	Post-op CD	Post-cholecystectomy	0.8333333333333334
2423	C15	P9	Post-op CD	Post-cholecystectomy	0.7238749046529367
2424	C15	P13	Post-op CD	Post-cholecystectomy	0.9077040427154843
2425	C15	P15	Post-op CD	Post-cholecystectomy	0.9359267734553776
2426	C15	P16	Post-op CD	Post-cholecystectomy	0.8453470633104501
2427	C15	P17	Post-op CD	Post-cholecystectomy	0.8503051106025934
2428	C15	P20	Post-op CD	Post-cholecystectomy	0.8182684973302822
2429	C15	P21	Post-op CD	Post-cholecystectomy	0.7726926010678871
2430	C15	P24	Post-op CD	Post-cholecystectomy	0.9477498093058734
2431	C15	P26	Post-op CD	Post-cholecystectomy	0.702326468344775
2432	C15	P30	Post-op CD	Post-cholecystectomy	0.9427917620137299
2433	C15	P33	Post-op CD	Post-cholecystectomy	0.8135011441647597
2434	C15	P35	Post-op CD	Post-cholecystectomy	0.8222730739893211
2435	C15	P38	Post-op CD	Post-cholecystectomy	0.9263920671243325
2436	C15	P39	Post-op CD	Post-cholecystectomy	0.8813882532418001
2437	C15	P42	Post-op CD	Post-cholecystectomy	0.6273836765827613
2438	C15	P43	Post-op CD	Post-cholecystectomy	0.8924485125858124
2439	C15	P46	Post-op CD	Post-cholecystectomy	0.8483981693363845
2440	C15	P47	Post-op CD	Post-cholecystectomy	0.9782608695652174
2441	C15	P50	Post-op CD	Post-cholecystectomy	0.8487795575896262
2442	C15	P55	Post-op CD	Post-cholecystectomy	0.780511060259344
2443	C15	P58	Post-op CD	Post-cholecystectomy	0.734744469870328
2444	C15	P60	Post-op CD	Post-cholecystectomy	0.9090389016018307
2445	C15	P63	Post-op CD	Post-cholecystectomy	0.950228832951945
2446	C15	P65	Post-op CD	Post-cholecystectomy	0.8165522501906941
2447	C15	P68	Post-op CD	Post-cholecystectomy	0.5596872616323417
2448	C15	P70	Post-op CD	Post-cholecystectomy	0.6559877955758963

2449	C15	P71	Post-op CD	Post-cholecystectomy	0.8913043478260869
2450	C15	P74	Post-op CD	Post-cholecystectomy	0.8237986270022883
2451	C15	P75	Post-op CD	Post-cholecystectomy	0.8790999237223494
2452	C19	P1	Post-op CD	Post-cholecystectomy	0.9729214340198322
2453	C19	P2	Post-op CD	Post-cholecystectomy	0.8560259344012204
2454	C19	P4	Post-op CD	Post-cholecystectomy	0.8623188405797102
2455	C19	P5	Post-op CD	Post-cholecystectomy	0.858886346300534
2456	C19	P9	Post-op CD	Post-cholecystectomy	0.7364607170099161
2457	C19	P13	Post-op CD	Post-cholecystectomy	0.9189549961861174
2458	C19	P15	Post-op CD	Post-cholecystectomy	0.8157894736842105
2459	C19	P16	Post-op CD	Post-cholecystectomy	0.8914950419527079
2460	C19	P17	Post-op CD	Post-cholecystectomy	0.8228451563691839
2461	C19	P20	Post-op CD	Post-cholecystectomy	0.8525934401220442
2462	C19	P21	Post-op CD	Post-cholecystectomy	0.801487414187643
2463	C19	P24	Post-op CD	Post-cholecystectomy	0.9685354691075515
2464	C19	P26	Post-op CD	Post-cholecystectomy	0.7065217391304348
2465	C19	P30	Post-op CD	Post-cholecystectomy	0.9725400457665904
2466	C19	P33	Post-op CD	Post-cholecystectomy	0.8350495804729214
2467	C19	P35	Post-op CD	Post-cholecystectomy	0.7713577421815409
2468	C19	P38	Post-op CD	Post-cholecystectomy	0.9698703279938978
2469	C19	P39	Post-op CD	Post-cholecystectomy	0.9221967963386728
2470	C19	P42	Post-op CD	Post-cholecystectomy	0.6292906178489702
2471	C19	P43	Post-op CD	Post-cholecystectomy	0.9195270785659801
2472	C19	P46	Post-op CD	Post-cholecystectomy	0.7238749046529367
2473	C19	P47	Post-op CD	Post-cholecystectomy	0.5568268497330282
2474	C19	P50	Post-op CD	Post-cholecystectomy	0.7869946605644547
2475	C19	P55	Post-op CD	Post-cholecystectomy	0.772692601067887 1

2476	C19	P58	Post-op CD	Post-cholecystectomy	0.7065217391304348
2477	C19	P60	Post-op CD	Post-cholecystectomy	0.9290617848970252
2478	C19	P63	Post-op CD	Post-cholecystectomy	0.8968344774980931
2479	C19	P65	Post-op CD	Post-cholecystectomy	0.8024408848207475
2480	C19	P68	Post-op CD	Post-cholecystectomy	0.7204424103737604
2481	C19	P70	Post-op CD	Post-cholecystectomy	0.6752479023646072
2482	C19	P71	Post-op CD	Post-cholecystectomy	0.6706712433257056
2483	C19	P74	Post-op CD	Post-cholecystectomy	0.8794813119755912
2484	C19	P75	Post-op CD	Post-cholecystectomy	0.8844393592677345
2485	C22	P1	Post-op CD	Post-cholecystectomy	0.7751716247139588
2486	C22	P2	Post-op CD	Post-cholecystectomy	0.8790999237223494
2487	C22	P4	Post-op CD	Post-cholecystectomy	0.9467963386727689
2488	C22	P5	Post-op CD	Post-cholecystectomy	0.9420289855072463
2489	C22	P9	Post-op CD	Post-cholecystectomy	0.9527078565980168
2490	C22	P13	Post-op CD	Post-cholecystectomy	0.9603356216628528
2491	C22	P15	Post-op CD	Post-cholecystectomy	0.9054157131960335
2492	C22	P16	Post-op CD	Post-cholecystectomy	0.914187643020595
2493	C22	P17	Post-op CD	Post-cholecystectomy	0.9628146453089245
2494	C22	P20	Post-op CD	Post-cholecystectomy	0.894927536231884
2495	C22	P21	Post-op CD	Post-cholecystectomy	0.938977879481312
2496	C22	P24	Post-op CD	Post-cholecystectomy	0.8903508771929824
2497	C22	P26	Post-op CD	Post-cholecystectomy	0.9530892448512586
2498	C22	P30	Post-op CD	Post-cholecystectomy	0.9101830663615561
2499	C22	P33	Post-op CD	Post-cholecystectomy	0.9328756674294432
2500	C22	P35	Post-op CD	Post-cholecystectomy	0.9691075514874142
2501	C22	P38	Post-op CD	Post-cholecystectomy	0.9029366895499619
2502	C22	P39	Post-op CD	Post-cholecystectomy	0.9288710907704043

2503 C22 P42 Post-op CD Post-cholecystectomy
0.9439359267734554
2504 C22 P43 Post-op CD Post-cholecystectomy
0.9590007627765065
2505 C22 P46 Post-op CD Post-cholecystectomy
0.9546147978642258
2506 C22 P47 Post-op CD Post-cholecystectomy
0.9366895499618612
2507 C22 P50 Post-op CD Post-cholecystectomy
0.9378337147215866
2508 C22 P55 Post-op CD Post-cholecystectomy
0.9469870327993898
2509 C22 P58 Post-op CD Post-cholecystectomy
0.9466056445461479
2510 C22 P60 Post-op CD Post-cholecystectomy
0.9593821510297483
2511 C22 P63 Post-op CD Post-cholecystectomy
0.9528985507246377
2512 C22 P65 Post-op CD Post-cholecystectomy
0.965675057208238
2513 C22 P68 Post-op CD Post-cholecystectomy
0.9269641495041953
2514 C22 P70 Post-op CD Post-cholecystectomy
0.9490846681922197
2515 C22 P71 Post-op CD Post-cholecystectomy
0.8716628527841342
2516 C22 P74 Post-op CD Post-cholecystectomy
0.8874904652936689
2517 C22 P75 Post-op CD Post-cholecystectomy
0.8533562166285278
2518 C26 P1 Post-op CD Post-cholecystectomy
0.9755911517925248
2519 C26 P2 Post-op CD Post-cholecystectomy
0.9353546910755148
2520 C26 P4 Post-op CD Post-cholecystectomy
0.8142639206712433
2521 C26 P5 Post-op CD Post-cholecystectomy
0.916094584286804
2522 C26 P9 Post-op CD Post-cholecystectomy
0.898741418764302
2523 C26 P13 Post-op CD Post-cholecystectomy
0.9296338672768879
2524 C26 P15 Post-op CD Post-cholecystectomy
0.8766209000762777
2525 C26 P16 Post-op CD Post-cholecystectomy
0.8426773455377574
2526 C26 P17 Post-op CD Post-cholecystectomy
0.9734935163996948
2527 C26 P20 Post-op CD Post-cholecystectomy
0.7240655987795576
2528 C26 P21 Post-op CD Post-cholecystectomy
0.8642257818459191
2529 C26 P24 Post-op CD Post-cholecystectomy
0.9557589626239512

2530	C26	P26	Post-op CD	Post-cholecystectomy	0.8735697940503433
2531	C26	P30	Post-op CD	Post-cholecystectomy	0.9382151029748284
2532	C26	P33	Post-op CD	Post-cholecystectomy	0.9122807017543859
2533	C26	P35	Post-op CD	Post-cholecystectomy	0.897025171624714
2534	C26	P38	Post-op CD	Post-cholecystectomy	0.879862700228833
2535	C26	P39	Post-op CD	Post-cholecystectomy	0.7816552250190694
2536	C26	P42	Post-op CD	Post-cholecystectomy	0.9479405034324943
2537	C26	P43	Post-op CD	Post-cholecystectomy	0.851258581235698
2538	C26	P46	Post-op CD	Post-cholecystectomy	0.8720442410373761
2539	C26	P47	Post-op CD	Post-cholecystectomy	0.9757818459191457
2540	C26	P50	Post-op CD	Post-cholecystectomy	0.915903890160183
2541	C26	P55	Post-op CD	Post-cholecystectomy	0.9563310450038138
2542	C26	P58	Post-op CD	Post-cholecystectomy	0.9433638443935927
2543	C26	P60	Post-op CD	Post-cholecystectomy	0.8548817696414951
2544	C26	P63	Post-op CD	Post-cholecystectomy	0.9401220442410374
2545	C26	P65	Post-op CD	Post-cholecystectomy	0.9324942791762014
2546	C26	P68	Post-op CD	Post-cholecystectomy	0.8152173913043478
2547	C26	P70	Post-op CD	Post-cholecystectomy	0.8941647597254004
2548	C26	P71	Post-op CD	Post-cholecystectomy	0.9242944317315027
2549	C26	P74	Post-op CD	Post-cholecystectomy	0.8943554538520213
2550	C26	P75	Post-op CD	Post-cholecystectomy	0.8663234172387491
2551	C28	P1	Post-op CD	Post-cholecystectomy	0.9645308924485125
2552	C28	P2	Post-op CD	Post-cholecystectomy	0.8756674294431731
2553	C28	P4	Post-op CD	Post-cholecystectomy	0.9061784897025171
2554	C28	P5	Post-op CD	Post-cholecystectomy	0.8100686498855835
2555	C28	P9	Post-op CD	Post-cholecystectomy	0.7105263157894737
2556	C28	P13	Post-op CD	Post-cholecystectomy	0.8768115942028986

2557	C28	P15	Post-op CD	Post-cholecystectomy	0.9422196796338673
2558	C28	P16	Post-op CD	Post-cholecystectomy	0.8613653699466056
2559	C28	P17	Post-op CD	Post-cholecystectomy	0.8821510297482837
2560	C28	P20	Post-op CD	Post-cholecystectomy	0.858886346300534
2561	C28	P21	Post-op CD	Post-cholecystectomy	0.7280701754385965
2562	C28	P24	Post-op CD	Post-cholecystectomy	0.9279176201372997
2563	C28	P26	Post-op CD	Post-cholecystectomy	0.5217391304347826
2564	C28	P30	Post-op CD	Post-cholecystectomy	0.950419527078566
2565	C28	P33	Post-op CD	Post-cholecystectomy	0.7967200610221206
2566	C28	P35	Post-op CD	Post-cholecystectomy	0.8104500381388253
2567	C28	P38	Post-op CD	Post-cholecystectomy	0.9338291380625476
2568	C28	P39	Post-op CD	Post-cholecystectomy	0.8840579710144928
2569	C28	P42	Post-op CD	Post-cholecystectomy	0.7486651411136537
2570	C28	P43	Post-op CD	Post-cholecystectomy	0.8630816170861938
2571	C28	P46	Post-op CD	Post-cholecystectomy	0.7803203661327232
2572	C28	P47	Post-op CD	Post-cholecystectomy	0.9450800915331807
2573	C28	P50	Post-op CD	Post-cholecystectomy	0.8390541571319603
2574	C28	P55	Post-op CD	Post-cholecystectomy	0.799771167048055
2575	C28	P58	Post-op CD	Post-cholecystectomy	0.7602974828375286
2576	C28	P60	Post-op CD	Post-cholecystectomy	0.9212433257055682
2577	C28	P63	Post-op CD	Post-cholecystectomy	0.9525171624713958
2578	C28	P65	Post-op CD	Post-cholecystectomy	0.8081617086193745
2579	C28	P68	Post-op CD	Post-cholecystectomy	0.7608695652173914
2580	C28	P70	Post-op CD	Post-cholecystectomy	0.7839435545385202
2581	C28	P71	Post-op CD	Post-cholecystectomy	0.8899694889397407
2582	C28	P74	Post-op CD	Post-cholecystectomy	0.7988176964149504
2583	C28	P75	Post-op CD	Post-cholecystectomy	0.8781464530892449

2584	C31	P1	Post-op CD	Post-cholecystectomy	0.9641495041952708
2585	C31	P2	Post-op CD	Post-cholecystectomy	0.9679633867276888
2586	C31	P4	Post-op CD	Post-cholecystectomy	0.9344012204424104
2587	C31	P5	Post-op CD	Post-cholecystectomy	0.9399313501144165
2588	C31	P9	Post-op CD	Post-cholecystectomy	0.8810068649885584
2589	C31	P13	Post-op CD	Post-cholecystectomy	0.9723493516399695
2590	C31	P15	Post-op CD	Post-cholecystectomy	0.965675057208238
2591	C31	P16	Post-op CD	Post-cholecystectomy	0.8852021357742181
2592	C31	P17	Post-op CD	Post-cholecystectomy	0.992372234935164
2593	C31	P20	Post-op CD	Post-cholecystectomy	0.9176201372997712
2594	C31	P21	Post-op CD	Post-cholecystectomy	0.6796338672768879
2595	C31	P24	Post-op CD	Post-cholecystectomy	0.9515636918382914
2596	C31	P26	Post-op CD	Post-cholecystectomy	0.780511060259344
2597	C31	P30	Post-op CD	Post-cholecystectomy	0.9290617848970252
2598	C31	P33	Post-op CD	Post-cholecystectomy	0.9191456903127384
2599	C31	P35	Post-op CD	Post-cholecystectomy	0.8569794050343249
2600	C31	P38	Post-op CD	Post-cholecystectomy	0.8182684973302822
2601	C31	P39	Post-op CD	Post-cholecystectomy	0.881769641495042
2602	C31	P42	Post-op CD	Post-cholecystectomy	0.9014111365369947
2603	C31	P43	Post-op CD	Post-cholecystectomy	0.9406941266209001
2604	C31	P46	Post-op CD	Post-cholecystectomy	0.8895881006864989
2605	C31	P47	Post-op CD	Post-cholecystectomy	0.9666285278413425
2606	C31	P50	Post-op CD	Post-cholecystectomy	0.9588100686498856
2607	C31	P55	Post-op CD	Post-cholecystectomy	0.9803585049580473
2608	C31	P58	Post-op CD	Post-cholecystectomy	0.7345537757437071
2609	C31	P60	Post-op CD	Post-cholecystectomy	0.8766209000762777
2610	C31	P63	Post-op CD	Post-cholecystectomy	0.9248665141113653

2611	C31	P65	Post-op CD	Post-cholecystectomy
0.9355453852021358
2612	C31	P68	Post-op CD	Post-cholecystectomy
0.8678489702517163
2613	C31	P70	Post-op CD	Post-cholecystectomy
0.9515636918382914
2614	C31	P71	Post-op CD	Post-cholecystectomy
0.9538520213577422
2615	C31	P74	Post-op CD	Post-cholecystectomy
0.9338291380625476
2616	C31	P75	Post-op CD	Post-cholecystectomy
0.9242944317315027
2617	C35	P1	Post-op CD	Post-cholecystectomy
0.9536613272311213
2618	C35	P2	Post-op CD	Post-cholecystectomy
0.8735697940503433
2619	C35	P4	Post-op CD	Post-cholecystectomy
0.9149504195270786
2620	C35	P5	Post-op CD	Post-cholecystectomy
0.8442028985507246
2621	C35	P9	Post-op CD	Post-cholecystectomy
0.7398932112890922
2622	C35	P13	Post-op CD	Post-cholecystectomy
0.8575514874141876
2623	C35	P15	Post-op CD	Post-cholecystectomy
0.8832951945080092
2624	C35	P16	Post-op CD	Post-cholecystectomy
0.8466819221967964
2625	C35	P17	Post-op CD	Post-cholecystectomy
0.8565980167810832
2626	C35	P20	Post-op CD	Post-cholecystectomy
0.9033180778032036
2627	C35	P21	Post-op CD	Post-cholecystectomy
0.8272311212814645
2628	C35	P24	Post-op CD	Post-cholecystectomy
0.9443173150266971
2629	C35	P26	Post-op CD	Post-cholecystectomy
0.6773455377574371
2630	C35	P30	Post-op CD	Post-cholecystectomy
0.9515636918382914
2631	C35	P33	Post-op CD	Post-cholecystectomy
0.8390541571319603
2632	C35	P35	Post-op CD	Post-cholecystectomy
0.7717391304347826
2633	C35	P38	Post-op CD	Post-cholecystectomy
0.9355453852021358
2634	C35	P39	Post-op CD	Post-cholecystectomy
0.9471777269260107
2635	C35	P42	Post-op CD	Post-cholecystectomy
0.6468344774980931
2636	C35	P43	Post-op CD	Post-cholecystectomy
0.9090389016018307
2637	C35	P46	Post-op CD	Post-cholecystectomy
0.759534706331045

2638	C35	P47	Post-op CD	Post-cholecystectomy	0.7562929061784897
2639	C35	P50	Post-op CD	Post-cholecystectomy	0.8226544622425629
2640	C35	P55	Post-op CD	Post-cholecystectomy	0.7950038138825324
2641	C35	P58	Post-op CD	Post-cholecystectomy	0.7620137299771167
2642	C35	P60	Post-op CD	Post-cholecystectomy	0.9588100686498856
2643	C35	P63	Post-op CD	Post-cholecystectomy	0.9595728451563692
2644	C35	P65	Post-op CD	Post-cholecystectomy	0.813119755911518
2645	C35	P68	Post-op CD	Post-cholecystectomy	0.7166285278413425
2646	C35	P70	Post-op CD	Post-cholecystectomy	0.704042715484363
2647	C35	P71	Post-op CD	Post-cholecystectomy	0.6292906178489702
2648	C35	P74	Post-op CD	Post-cholecystectomy	0.834096109839817
2649	C35	P75	Post-op CD	Post-cholecystectomy	0.8829138062547673
2650	C38	P1	Post-op CD	Post-cholecystectomy	0.9721586575133486
2651	C38	P2	Post-op CD	Post-cholecystectomy	0.9654843630816171
2652	C38	P4	Post-op CD	Post-cholecystectomy	0.9401220442410374
2653	C38	P5	Post-op CD	Post-cholecystectomy	0.9761632341723875
2654	C38	P9	Post-op CD	Post-cholecystectomy	0.9692982456140351
2655	C38	P13	Post-op CD	Post-cholecystectomy	0.9788329519450801
2656	C38	P15	Post-op CD	Post-cholecystectomy	0.9399313501144165
2657	C38	P16	Post-op CD	Post-cholecystectomy	0.8419145690312738
2658	C38	P17	Post-op CD	Post-cholecystectomy	0.9717772692601068
2659	C38	P20	Post-op CD	Post-cholecystectomy	0.9528985507246377
2660	C38	P21	Post-op CD	Post-cholecystectomy	0.914187643020595
2661	C38	P24	Post-op CD	Post-cholecystectomy	0.9830282227307399
2662	C38	P26	Post-op CD	Post-cholecystectomy	0.9710144927536232
2663	C38	P30	Post-op CD	Post-cholecystectomy	0.8665141113653699
2664	C38	P33	Post-op CD	Post-cholecystectomy	0.935163996948894

2665	C38	P35	Post-op CD	Post-cholecystectomy	0.9774980930587338
2666	C38	P38	Post-op CD	Post-cholecystectomy	0.8709000762776506
2667	C38	P39	Post-op CD	Post-cholecystectomy	0.8836765827612509
2668	C38	P42	Post-op CD	Post-cholecystectomy	0.9115179252479023
2669	C38	P43	Post-op CD	Post-cholecystectomy	0.937070938215103
2670	C38	P46	Post-op CD	Post-cholecystectomy	0.9372616323417239
2671	C38	P47	Post-op CD	Post-cholecystectomy	0.9727307398932112
2672	C38	P50	Post-op CD	Post-cholecystectomy	0.9698703279938978
2673	C38	P55	Post-op CD	Post-cholecystectomy	0.9616704805491991
2674	C38	P58	Post-op CD	Post-cholecystectomy	0.9763539282990084
2675	C38	P60	Post-op CD	Post-cholecystectomy	0.9492753623188406
2676	C38	P63	Post-op CD	Post-cholecystectomy	0.9750190694126621
2677	C38	P65	Post-op CD	Post-cholecystectomy	0.969488939740656
2678	C38	P68	Post-op CD	Post-cholecystectomy	0.9759725400457666
2679	C38	P70	Post-op CD	Post-cholecystectomy	0.9759725400457666
2680	C38	P71	Post-op CD	Post-cholecystectomy	0.9744469870327994
2681	C38	P74	Post-op CD	Post-cholecystectomy	0.9801678108314263
2682	C38	P75	Post-op CD	Post-cholecystectomy	0.9759725400457666
2683	C40	P1	Post-op CD	Post-cholecystectomy	0.9605263157894737
2684	C40	P2	Post-op CD	Post-cholecystectomy	0.9569031273836766
2685	C40	P4	Post-op CD	Post-cholecystectomy	0.7831807780320366
2686	C40	P5	Post-op CD	Post-cholecystectomy	0.8796720061022121
2687	C40	P9	Post-op CD	Post-cholecystectomy	0.872234935163997
2688	C40	P13	Post-op CD	Post-cholecystectomy	0.9101830663615561
2689	C40	P15	Post-op CD	Post-cholecystectomy	0.877765064836003
2690	C40	P16	Post-op CD	Post-cholecystectomy	0.7745995423340961
2691	C40	P17	Post-op CD	Post-cholecystectomy	0.9658657513348589

2692	C40	P20	Post-op CD	Post-cholecystectomy	0.6601830663615561
2693	C40	P21	Post-op CD	Post-cholecystectomy	0.8127383676582761
2694	C40	P24	Post-op CD	Post-cholecystectomy	0.9450800915331807
2695	C40	P26	Post-op CD	Post-cholecystectomy	0.8886346300533944
2696	C40	P30	Post-op CD	Post-cholecystectomy	0.8878718535469108
2697	C40	P33	Post-op CD	Post-cholecystectomy	0.8710907704042715
2698	C40	P35	Post-op CD	Post-cholecystectomy	0.9225781845919145
2699	C40	P38	Post-op CD	Post-cholecystectomy	0.8899694889397407
2700	C40	P39	Post-op CD	Post-cholecystectomy	0.7807017543859649
2701	C40	P42	Post-op CD	Post-cholecystectomy	0.9363081617086194
2702	C40	P43	Post-op CD	Post-cholecystectomy	0.7646834477498093
2703	C40	P46	Post-op CD	Post-cholecystectomy	0.8697559115179252
2704	C40	P47	Post-op CD	Post-cholecystectomy	0.9578565980167811
2705	C40	P50	Post-op CD	Post-cholecystectomy	0.9321128909229596
2706	C40	P55	Post-op CD	Post-cholecystectomy	0.9342105263157895
2707	C40	P58	Post-op CD	Post-cholecystectomy	0.9021739130434783
2708	C40	P60	Post-op CD	Post-cholecystectomy	0.6853546910755148
2709	C40	P63	Post-op CD	Post-cholecystectomy	0.9492753623188406
2710	C40	P65	Post-op CD	Post-cholecystectomy	0.8653699466056446
2711	C40	P68	Post-op CD	Post-cholecystectomy	0.9048436308161708
2712	C40	P70	Post-op CD	Post-cholecystectomy	0.8712814645308925
2713	C40	P71	Post-op CD	Post-cholecystectomy	0.9466056445461479
2714	C40	P74	Post-op CD	Post-cholecystectomy	0.8888253241800153
2715	C40	P75	Post-op CD	Post-cholecystectomy	0.8625095347063311
2716	C44	P1	Post-op CD	Post-cholecystectomy	0.9778794813119756
2717	C44	P2	Post-op CD	Post-cholecystectomy	0.8813882532418001
2718	C44	P4	Post-op CD	Post-cholecystectomy	0.8993135011441648

2719	C44	P5	Post-op CD	Post-cholecystectomy	0.8712814645308925
2720	C44	P9	Post-op CD	Post-cholecystectomy	0.7793668954996186
2721	C44	P13	Post-op CD	Post-cholecystectomy	0.9563310450038138
2722	C44	P15	Post-op CD	Post-cholecystectomy	0.959954233409611
2723	C44	P16	Post-op CD	Post-cholecystectomy	0.8688024408848207
2724	C44	P17	Post-op CD	Post-cholecystectomy	0.8443935926773455
2725	C44	P20	Post-op CD	Post-cholecystectomy	0.9344012204424104
2726	C44	P21	Post-op CD	Post-cholecystectomy	0.8230358504958047
2727	C44	P24	Post-op CD	Post-cholecystectomy	0.9784515636918383
2728	C44	P26	Post-op CD	Post-cholecystectomy	0.6939359267734554
2729	C44	P30	Post-op CD	Post-cholecystectomy	0.9750190694126621
2730	C44	P33	Post-op CD	Post-cholecystectomy	0.8062547673531655
2731	C44	P35	Post-op CD	Post-cholecystectomy	0.8236079328756675
2732	C44	P38	Post-op CD	Post-cholecystectomy	0.973302822273074
2733	C44	P39	Post-op CD	Post-cholecystectomy	0.9506102212051869
2734	C44	P42	Post-op CD	Post-cholecystectomy	0.5659801678108314
2735	C44	P43	Post-op CD	Post-cholecystectomy	0.9509916094584286
2736	C44	P46	Post-op CD	Post-cholecystectomy	0.8625095347063311
2737	C44	P47	Post-op CD	Post-cholecystectomy	0.9757818459191457
2738	C44	P50	Post-op CD	Post-cholecystectomy	0.8323798627002288
2739	C44	P55	Post-op CD	Post-cholecystectomy	0.8026315789473685
2740	C44	P58	Post-op CD	Post-cholecystectomy	0.7240655987795576
2741	C44	P60	Post-op CD	Post-cholecystectomy	0.9258199847444699
2742	C44	P63	Post-op CD	Post-cholecystectomy	0.8096872616323417
2743	C44	P65	Post-op CD	Post-cholecystectomy	0.8123569794050344
2744	C44	P68	Post-op CD	Post-cholecystectomy	0.614607170099161
2745	C44	P70	Post-op CD	Post-cholecystectomy	0.6857360793287567

2746	C44	P71	Post-op CD	Post-cholecystectomy	0.8344774980930587
2747	C44	P74	Post-op CD	Post-cholecystectomy	0.8823417238749046
2748	C44	P75	Post-op CD	Post-cholecystectomy	0.9387871853546911
2749	C47	P1	Post-op CD	Post-cholecystectomy	0.9816933638443935
2750	C47	P2	Post-op CD	Post-cholecystectomy	0.8924485125858124
2751	C47	P4	Post-op CD	Post-cholecystectomy	0.8714721586575134
2752	C47	P5	Post-op CD	Post-cholecystectomy	0.8726163234172387
2753	C47	P9	Post-op CD	Post-cholecystectomy	0.8070175438596491
2754	C47	P13	Post-op CD	Post-cholecystectomy	0.967391304347826
2755	C47	P15	Post-op CD	Post-cholecystectomy	0.9595728451563692
2756	C47	P16	Post-op CD	Post-cholecystectomy	0.9187643020594966
2757	C47	P17	Post-op CD	Post-cholecystectomy	0.8585049580472921
2758	C47	P20	Post-op CD	Post-cholecystectomy	0.9702517162471396
2759	C47	P21	Post-op CD	Post-cholecystectomy	0.8848207475209764
2760	C47	P24	Post-op CD	Post-cholecystectomy	0.9774980930587338
2761	C47	P26	Post-op CD	Post-cholecystectomy	0.7580091533180778
2762	C47	P30	Post-op CD	Post-cholecystectomy	0.9780701754385965
2763	C47	P33	Post-op CD	Post-cholecystectomy	0.9117086193745233
2764	C47	P35	Post-op CD	Post-cholecystectomy	0.8636536994660564
2765	C47	P38	Post-op CD	Post-cholecystectomy	0.9794050343249427
2766	C47	P39	Post-op CD	Post-cholecystectomy	0.973302822273074
2767	C47	P42	Post-op CD	Post-cholecystectomy	0.5705568268497331
2768	C47	P43	Post-op CD	Post-cholecystectomy	0.9851258581235698
2769	C47	P46	Post-op CD	Post-cholecystectomy	0.8663234172387491
2770	C47	P47	Post-op CD	Post-cholecystectomy	0.9706331045003814
2771	C47	P50	Post-op CD	Post-cholecystectomy	0.8800533943554538
2772	C47	P55	Post-op CD	Post-cholecystectomy	0.825324180015255

2773 C47 P58 Post-op CD Post-cholecystectomy
0.6937452326468345
2774 C47 P60 Post-op CD Post-cholecystectomy
0.873951182303585
2775 C47 P63 Post-op CD Post-cholecystectomy
0.9422196796338673
2776 C47 P65 Post-op CD Post-cholecystectomy
0.8651792524790236
2777 C47 P68 Post-op CD Post-cholecystectomy
0.643211289092296
2778 C47 P70 Post-op CD Post-cholecystectomy
0.7524790236460717
2779 C47 P71 Post-op CD Post-cholecystectomy
0.7263539282990084
2780 C47 P74 Post-op CD Post-cholecystectomy
0.9258199847444699
2781 C47 P75 Post-op CD Post-cholecystectomy
0.9344012204424104
2782 C48 P1 Post-op CD Post-cholecystectomy
0.8899694889397407
2783 C48 P2 Post-op CD Post-cholecystectomy
0.7858504958047292
2784 C48 P4 Post-op CD Post-cholecystectomy
0.9046529366895499
2785 C48 P5 Post-op CD Post-cholecystectomy
0.8260869565217391
2786 C48 P9 Post-op CD Post-cholecystectomy
0.8867276887871853
2787 C48 P13 Post-op CD Post-cholecystectomy
0.8693745232646835
2788 C48 P15 Post-op CD Post-cholecystectomy
0.7726926010678871
2789 C48 P16 Post-op CD Post-cholecystectomy
0.8632723112128147
2790 C48 P17 Post-op CD Post-cholecystectomy
0.8636536994660564
2791 C48 P20 Post-op CD Post-cholecystectomy
0.8945461479786423
2792 C48 P21 Post-op CD Post-cholecystectomy
0.8926392067124332
2793 C48 P24 Post-op CD Post-cholecystectomy
0.8545003813882532
2794 C48 P26 Post-op CD Post-cholecystectomy
0.9164759725400458
2795 C48 P30 Post-op CD Post-cholecystectomy
0.9263920671243325
2796 C48 P33 Post-op CD Post-cholecystectomy
0.8667048054919908
2797 C48 P35 Post-op CD Post-cholecystectomy
0.8495423340961098
2798 C48 P38 Post-op CD Post-cholecystectomy
0.933257055682685
2799 C48 P39 Post-op CD Post-cholecystectomy
0.897025171624714

2800	C48	P42	Post-op CD	Post-cholecystectomy
0.9113272311212814
2801	C48	P43	Post-op CD	Post-cholecystectomy
0.9088482074752098
2802	C48	P46	Post-op CD	Post-cholecystectomy
0.6899313501144165
2803	C48	P47	Post-op CD	Post-cholecystectomy
0.6596109839816934
2804	C48	P50	Post-op CD	Post-cholecystectomy
0.8142639206712433
2805	C48	P55	Post-op CD	Post-cholecystectomy
0.8762395118230358
2806	C48	P58	Post-op CD	Post-cholecystectomy
0.9046529366895499
2807	C48	P60	Post-op CD	Post-cholecystectomy
0.9651029748283753
2808	C48	P63	Post-op CD	Post-cholecystectomy
0.9002669717772692
2809	C48	P65	Post-op CD	Post-cholecystectomy
0.8585049580472921
2810	C48	P68	Post-op CD	Post-cholecystectomy
0.8829138062547673
2811	C48	P70	Post-op CD	Post-cholecystectomy
0.8607932875667429
2812	C48	P71	Post-op CD	Post-cholecystectomy
0.6790617848970252
2813	C48	P74	Post-op CD	Post-cholecystectomy
0.8615560640732265
2814	C48	P75	Post-op CD	Post-cholecystectomy
0.8234172387490465
2815	C49	P1	Post-op CD	Post-cholecystectomy
0.8920671243325705
2816	C49	P2	Post-op CD	Post-cholecystectomy
0.852974828375286
2817	C49	P4	Post-op CD	Post-cholecystectomy
0.8062547673531655
2818	C49	P5	Post-op CD	Post-cholecystectomy
0.8789092295957285
2819	C49	P9	Post-op CD	Post-cholecystectomy
0.9229595728451564
2820	C49	P13	Post-op CD	Post-cholecystectomy
0.8890160183066361
2821	C49	P15	Post-op CD	Post-cholecystectomy
0.7818459191456903
2822	C49	P16	Post-op CD	Post-cholecystectomy
0.7644927536231884
2823	C49	P17	Post-op CD	Post-cholecystectomy
0.9193363844393593
2824	C49	P20	Post-op CD	Post-cholecystectomy
0.6924103737604882
2825	C49	P21	Post-op CD	Post-cholecystectomy
0.9317315026697178
2826	C49	P24	Post-op CD	Post-cholecystectomy
0.8712814645308925

2827	C49	P26	Post-op CD	Post-cholecystectomy
0.9256292906178489
2828	C49	P30	Post-op CD	Post-cholecystectomy
0.9029366895499619
2829	C49	P33	Post-op CD	Post-cholecystectomy
0.9040808543096872
2830	C49	P35	Post-op CD	Post-cholecystectomy
0.9300152555301296
2831	C49	P38	Post-op CD	Post-cholecystectomy
0.9281083142639207
2832	C49	P39	Post-op CD	Post-cholecystectomy
0.872234935163997
2833	C49	P42	Post-op CD	Post-cholecystectomy
0.9187643020594966
2834	C49	P43	Post-op CD	Post-cholecystectomy
0.8548817696414951
2835	C49	P46	Post-op CD	Post-cholecystectomy
0.7915713196033562
2836	C49	P47	Post-op CD	Post-cholecystectomy
0.8545003813882532
2837	C49	P50	Post-op CD	Post-cholecystectomy
0.9046529366895499
2838	C49	P55	Post-op CD	Post-cholecystectomy
0.9220061022120518
2839	C49	P58	Post-op CD	Post-cholecystectomy
0.950419527078566
2840	C49	P60	Post-op CD	Post-cholecystectomy
0.7726926010678871
2841	C49	P63	Post-op CD	Post-cholecystectomy
0.9282990083905416
2842	C49	P65	Post-op CD	Post-cholecystectomy
0.9220061022120518
2843	C49	P68	Post-op CD	Post-cholecystectomy
0.9185736079328757
2844	C49	P70	Post-op CD	Post-cholecystectomy
0.9126620900076278
2845	C49	P71	Post-op CD	Post-cholecystectomy
0.8068268497330282
2846	C49	P74	Post-op CD	Post-cholecystectomy
0.92372234935164
2847	C49	P75	Post-op CD	Post-cholecystectomy
0.8783371472158658
2848	C53	P1	Post-op CD	Post-cholecystectomy
0.9265827612509535
2849	C53	P2	Post-op CD	Post-cholecystectomy
0.801487414187643
2850	C53	P4	Post-op CD	Post-cholecystectomy
0.9338291380625476
2851	C53	P5	Post-op CD	Post-cholecystectomy
0.8205568268497331
2852	C53	P9	Post-op CD	Post-cholecystectomy
0.7898550724637681
2853	C53	P13	Post-op CD	Post-cholecystectomy
0.8996948893974066

2854	C53	P15	Post-op CD	Post-cholecystectomy
0.8276125095347063
2855	C53	P16	Post-op CD	Post-cholecystectomy
0.8846300533943554
2856	C53	P17	Post-op CD	Post-cholecystectomy
0.8312356979405034
2857	C53	P20	Post-op CD	Post-cholecystectomy
0.9225781845919145
2858	C53	P21	Post-op CD	Post-cholecystectomy
0.8409610983981693
2859	C53	P24	Post-op CD	Post-cholecystectomy
0.8842486651411137
2860	C53	P26	Post-op CD	Post-cholecystectomy
0.7991990846681922
2861	C53	P30	Post-op CD	Post-cholecystectomy
0.9494660564454614
2862	C53	P33	Post-op CD	Post-cholecystectomy
0.8403890160183066
2863	C53	P35	Post-op CD	Post-cholecystectomy
0.8033943554538521
2864	C53	P38	Post-op CD	Post-cholecystectomy
0.9328756674294432
2865	C53	P39	Post-op CD	Post-cholecystectomy
0.9544241037376049
2866	C53	P42	Post-op CD	Post-cholecystectomy
0.7088100686498856
2867	C53	P43	Post-op CD	Post-cholecystectomy
0.9315408085430968
2868	C53	P46	Post-op CD	Post-cholecystectomy
0.7873760488176964
2869	C53	P47	Post-op CD	Post-cholecystectomy
0.816742944317315
2870	C53	P50	Post-op CD	Post-cholecystectomy
0.8115942028985508
2871	C53	P55	Post-op CD	Post-cholecystectomy
0.7940503432494279
2872	C53	P58	Post-op CD	Post-cholecystectomy
0.8087337909992373
2873	C53	P60	Post-op CD	Post-cholecystectomy
0.9672006102212052
2874	C53	P63	Post-op CD	Post-cholecystectomy
0.9231502669717773
2875	C53	P65	Post-op CD	Post-cholecystectomy
0.8102593440122045
2876	C53	P68	Post-op CD	Post-cholecystectomy
0.7862318840579711
2877	C53	P70	Post-op CD	Post-cholecystectomy
0.7892829900839055
2878	C53	P71	Post-op CD	Post-cholecystectomy
0.5932494279176201
2879	C53	P74	Post-op CD	Post-cholecystectomy
0.8768115942028986
2880	C53	P75	Post-op CD	Post-cholecystectomy
0.8964530892448512

2881	C56	P1	Post-op CD	Post-cholecystectomy	0.8977879481311976
2882	C56	P2	Post-op CD	Post-cholecystectomy	0.7406559877955758
2883	C56	P4	Post-op CD	Post-cholecystectomy	0.8993135011441648
2884	C56	P5	Post-op CD	Post-cholecystectomy	0.7048054919908466
2885	C56	P9	Post-op CD	Post-cholecystectomy	0.7234935163996948
2886	C56	P13	Post-op CD	Post-cholecystectomy	0.8333333333333334
2887	C56	P15	Post-op CD	Post-cholecystectomy	0.7885202135774219
2888	C56	P16	Post-op CD	Post-cholecystectomy	0.8367658276125095
2889	C56	P17	Post-op CD	Post-cholecystectomy	0.8348588863463006
2890	C56	P20	Post-op CD	Post-cholecystectomy	0.8222730739893211
2891	C56	P21	Post-op CD	Post-cholecystectomy	0.729023646071701
2892	C56	P24	Post-op CD	Post-cholecystectomy	0.8180778032036613
2893	C56	P26	Post-op CD	Post-cholecystectomy	0.757627765064836
2894	C56	P30	Post-op CD	Post-cholecystectomy	0.9193363844393593
2895	C56	P33	Post-op CD	Post-cholecystectomy	0.7911899313501144
2896	C56	P35	Post-op CD	Post-cholecystectomy	0.7873760488176964
2897	C56	P38	Post-op CD	Post-cholecystectomy	0.9359267734553776
2898	C56	P39	Post-op CD	Post-cholecystectomy	0.8422959572845157
2899	C56	P42	Post-op CD	Post-cholecystectomy	0.8123569794050344
2900	C56	P43	Post-op CD	Post-cholecystectomy	0.8497330282227308
2901	C56	P46	Post-op CD	Post-cholecystectomy	0.732837528604119
2902	C56	P47	Post-op CD	Post-cholecystectomy	0.8176964149504196
2903	C56	P50	Post-op CD	Post-cholecystectomy	0.7526697177726926
2904	C56	P55	Post-op CD	Post-cholecystectomy	0.7475209763539283
2905	C56	P58	Post-op CD	Post-cholecystectomy	0.7257818459191457
2906	C56	P60	Post-op CD	Post-cholecystectomy	0.9002669717772692
2907	C56	P63	Post-op CD	Post-cholecystectomy	0.8747139588100686

2908	C56	P65	Post-op CD	Post-cholecystectomy	0.761441647597254
2909	C56	P68	Post-op CD	Post-cholecystectomy	0.7067124332570557
2910	C56	P70	Post-op CD	Post-cholecystectomy	0.7313119755911518
2911	C56	P71	Post-op CD	Post-cholecystectomy	0.6916475972540046
2912	C56	P74	Post-op CD	Post-cholecystectomy	0.8098779557589626
2913	C56	P75	Post-op CD	Post-cholecystectomy	0.7776506483600305
2914	C60	P1	Post-op CD	Post-cholecystectomy	0.8293287566742944
2915	C60	P2	Post-op CD	Post-cholecystectomy	0.7713577421815409
2916	C60	P4	Post-op CD	Post-cholecystectomy	0.8892067124332571
2917	C60	P5	Post-op CD	Post-cholecystectomy	0.7951945080091534
2918	C60	P9	Post-op CD	Post-cholecystectomy	0.7702135774218154
2919	C60	P13	Post-op CD	Post-cholecystectomy	0.897025171624714
2920	C60	P15	Post-op CD	Post-cholecystectomy	0.7515255530129672
2921	C60	P16	Post-op CD	Post-cholecystectomy	0.8327612509534706
2922	C60	P17	Post-op CD	Post-cholecystectomy	0.8623188405797102
2923	C60	P20	Post-op CD	Post-cholecystectomy	0.8247520976353928
2924	C60	P21	Post-op CD	Post-cholecystectomy	0.8424866514111365
2925	C60	P24	Post-op CD	Post-cholecystectomy	0.8983600305110603
2926	C60	P26	Post-op CD	Post-cholecystectomy	0.778604118993135
2927	C60	P30	Post-op CD	Post-cholecystectomy	0.8627002288329519
2928	C60	P33	Post-op CD	Post-cholecystectomy	0.82627765064836
2929	C60	P35	Post-op CD	Post-cholecystectomy	0.8178871090770404
2930	C60	P38	Post-op CD	Post-cholecystectomy	0.9105644546147978
2931	C60	P39	Post-op CD	Post-cholecystectomy	0.8977879481311976
2932	C60	P42	Post-op CD	Post-cholecystectomy	0.7236842105263158
2933	C60	P43	Post-op CD	Post-cholecystectomy	0.910373760488177
2934	C60	P46	Post-op CD	Post-cholecystectomy	0.7816552250190694

2935	C60	P47	Post-op CD	Post-cholecystectomy
0.8165522501906941
2936	C60	P50	Post-op CD	Post-cholecystectomy
0.7898550724637681
2937	C60	P55	Post-op CD	Post-cholecystectomy
0.7376048817696415
2938	C60	P58	Post-op CD	Post-cholecystectomy
0.7130053394355453
2939	C60	P60	Post-op CD	Post-cholecystectomy
0.9216247139588101
2940	C60	P63	Post-op CD	Post-cholecystectomy
0.8993135011441648
2941	C60	P65	Post-op CD	Post-cholecystectomy
0.8201754385964912
2942	C60	P68	Post-op CD	Post-cholecystectomy
0.7200610221205187
2943	C60	P70	Post-op CD	Post-cholecystectomy
0.7105263157894737
2944	C60	P71	Post-op CD	Post-cholecystectomy
0.7536231884057971
2945	C60	P74	Post-op CD	Post-cholecystectomy
0.8525934401220442
2946	C60	P75	Post-op CD	Post-cholecystectomy
0.8442028985507246
2947	C62	P1	Post-op CD	Post-cholecystectomy
0.9303966437833715
2948	C62	P2	Post-op CD	Post-cholecystectomy
0.8485888634630053
2949	C62	P4	Post-op CD	Post-cholecystectomy
0.9181922196796338
2950	C62	P5	Post-op CD	Post-cholecystectomy
0.8480167810831426
2951	C62	P9	Post-op CD	Post-cholecystectomy
0.8981693363844394
2952	C62	P13	Post-op CD	Post-cholecystectomy
0.9016018306636155
2953	C62	P15	Post-op CD	Post-cholecystectomy
0.8159801678108314
2954	C62	P16	Post-op CD	Post-cholecystectomy
0.8846300533943554
2955	C62	P17	Post-op CD	Post-cholecystectomy
0.9218154080854309
2956	C62	P20	Post-op CD	Post-cholecystectomy
0.8724256292906178
2957	C62	P21	Post-op CD	Post-cholecystectomy
0.8754767353165522
2958	C62	P24	Post-op CD	Post-cholecystectomy
0.8726163234172387
2959	C62	P26	Post-op CD	Post-cholecystectomy
0.8886346300533944
2960	C62	P30	Post-op CD	Post-cholecystectomy
0.916094584286804
2961	C62	P33	Post-op CD	Post-cholecystectomy
0.8800533943554538

2962	C62	P35	Post-op CD	Post-cholecystectomy	0.8850114416475973
2963	C62	P38	Post-op CD	Post-cholecystectomy	0.795957284515637
2964	C62	P39	Post-op CD	Post-cholecystectomy	0.8794813119755912
2965	C62	P42	Post-op CD	Post-cholecystectomy	0.8867276887871853
2966	C62	P43	Post-op CD	Post-cholecystectomy	0.9181922196796338
2967	C62	P46	Post-op CD	Post-cholecystectomy	0.7465675057208238
2968	C62	P47	Post-op CD	Post-cholecystectomy	0.8495423340961098
2969	C62	P50	Post-op CD	Post-cholecystectomy	0.8838672768878718
2970	C62	P55	Post-op CD	Post-cholecystectomy	0.9107551487414187
2971	C62	P58	Post-op CD	Post-cholecystectomy	0.8321891685736079
2972	C62	P60	Post-op CD	Post-cholecystectomy	0.8884439359267735
2973	C62	P63	Post-op CD	Post-cholecystectomy	0.8909229595728452
2974	C62	P65	Post-op CD	Post-cholecystectomy	0.8607932875667429
2975	C62	P68	Post-op CD	Post-cholecystectomy	0.8270404271548436
2976	C62	P70	Post-op CD	Post-cholecystectomy	0.8895881006864989
2977	C62	P71	Post-op CD	Post-cholecystectomy	0.7719298245614035
2978	C62	P74	Post-op CD	Post-cholecystectomy	0.8792906178489702
2979	C62	P75	Post-op CD	Post-cholecystectomy	0.8428680396643783
2980	C64	P1	Post-op CD	Post-cholecystectomy	0.902745995423341
2981	C64	P2	Post-op CD	Post-cholecystectomy	0.7990083905415714
2982	C64	P4	Post-op CD	Post-cholecystectomy	0.9090389016018307
2983	C64	P5	Post-op CD	Post-cholecystectomy	0.8598398169336384
2984	C64	P9	Post-op CD	Post-cholecystectomy	0.9016018306636155
2985	C64	P13	Post-op CD	Post-cholecystectomy	0.8886346300533944
2986	C64	P15	Post-op CD	Post-cholecystectomy	0.7940503432494279
2987	C64	P16	Post-op CD	Post-cholecystectomy	0.9096109839816934
2988	C64	P17	Post-op CD	Post-cholecystectomy	0.8918764302059496

2989	C64	P20	Post-op CD	Post-cholecystectomy	0.9050343249427918
2990	C64	P21	Post-op CD	Post-cholecystectomy	0.8789092295957285
2991	C64	P24	Post-op CD	Post-cholecystectomy	0.851258581235698
2992	C64	P26	Post-op CD	Post-cholecystectomy	0.8939740655987796
2993	C64	P30	Post-op CD	Post-cholecystectomy	0.8832951945080092
2994	C64	P33	Post-op CD	Post-cholecystectomy	0.8686117467581999
2995	C64	P35	Post-op CD	Post-cholecystectomy	0.8737604881769642
2996	C64	P38	Post-op CD	Post-cholecystectomy	0.6024027459954233
2997	C64	P39	Post-op CD	Post-cholecystectomy	0.9277269260106789
2998	C64	P42	Post-op CD	Post-cholecystectomy	0.9078947368421053
2999	C64	P43	Post-op CD	Post-cholecystectomy	0.9157131960335622
3000	C64	P46	Post-op CD	Post-cholecystectomy	0.7930968726163234
3001	C64	P47	Post-op CD	Post-cholecystectomy	0.8247520976353928
3002	C64	P50	Post-op CD	Post-cholecystectomy	0.8381006864988558
3003	C64	P55	Post-op CD	Post-cholecystectomy	0.8943554538520213
3004	C64	P58	Post-op CD	Post-cholecystectomy	0.910373760488177
3005	C64	P60	Post-op CD	Post-cholecystectomy	0.9328756674294432
3006	C64	P63	Post-op CD	Post-cholecystectomy	0.8972158657513348
3007	C64	P65	Post-op CD	Post-cholecystectomy	0.8810068649885584
3008	C64	P68	Post-op CD	Post-cholecystectomy	0.8968344774980931
3009	C64	P70	Post-op CD	Post-cholecystectomy	0.8962623951182304
3010	C64	P71	Post-op CD	Post-cholecystectomy	0.7452326468344775
3011	C64	P74	Post-op CD	Post-cholecystectomy	0.8796720061022121
3012	C64	P75	Post-op CD	Post-cholecystectomy	0.8506864988558352
3013	C65	P1	Post-op CD	Post-cholecystectomy	0.8024408848207475
3014	C65	P2	Post-op CD	Post-cholecystectomy	0.8150266971777269
3015	C65	P4	Post-op CD	Post-cholecystectomy	0.9538520213577422

3016	C65	P5	Post-op CD	Post-cholecystectomy	0.8613653699466056
3017	C65	P9	Post-op CD	Post-cholecystectomy	0.9517543859649122
3018	C65	P13	Post-op CD	Post-cholecystectomy	0.8956903127383676
3019	C65	P15	Post-op CD	Post-cholecystectomy	0.8125476735316552
3020	C65	P16	Post-op CD	Post-cholecystectomy	0.9170480549199085
3021	C65	P17	Post-op CD	Post-cholecystectomy	0.9036994660564455
3022	C65	P20	Post-op CD	Post-cholecystectomy	0.8667048054919908
3023	C65	P21	Post-op CD	Post-cholecystectomy	0.9229595728451564
3024	C65	P24	Post-op CD	Post-cholecystectomy	0.8541189931350115
3025	C65	P26	Post-op CD	Post-cholecystectomy	0.9513729977116705
3026	C65	P30	Post-op CD	Post-cholecystectomy	0.8939740655987796
3027	C65	P33	Post-op CD	Post-cholecystectomy	0.8779557589626239
3028	C65	P35	Post-op CD	Post-cholecystectomy	0.9302059496567505
3029	C65	P38	Post-op CD	Post-cholecystectomy	0.8794813119755912
3030	C65	P39	Post-op CD	Post-cholecystectomy	0.9481311975591151
3031	C65	P42	Post-op CD	Post-cholecystectomy	0.9258199847444699
3032	C65	P43	Post-op CD	Post-cholecystectomy	0.9328756674294432
3033	C65	P46	Post-op CD	Post-cholecystectomy	0.7971014492753623
3034	C65	P47	Post-op CD	Post-cholecystectomy	0.8119755911517925
3035	C65	P50	Post-op CD	Post-cholecystectomy	0.8766209000762777
3036	C65	P55	Post-op CD	Post-cholecystectomy	0.894927536231884
3037	C65	P58	Post-op CD	Post-cholecystectomy	0.9380244088482075
3038	C65	P60	Post-op CD	Post-cholecystectomy	0.9692982456140351
3039	C65	P63	Post-op CD	Post-cholecystectomy	0.9254385964912281
3040	C65	P65	Post-op CD	Post-cholecystectomy	0.9038901601830663
3041	C65	P68	Post-op CD	Post-cholecystectomy	0.900839054157132
3042	C65	P70	Post-op CD	Post-cholecystectomy	0.9078947368421053

3043	C65	P71	Post-op CD	Post-cholecystectomy	0.7307398932112891
3044	C65	P74	Post-op CD	Post-cholecystectomy	0.8960717009916095
3045	C65	P75	Post-op CD	Post-cholecystectomy	0.8560259344012204
3046	C69	P1	Post-op CD	Post-cholecystectomy	0.9521357742181541
3047	C69	P2	Post-op CD	Post-cholecystectomy	0.8215102974828375
3048	C69	P4	Post-op CD	Post-cholecystectomy	0.7362700228832952
3049	C69	P5	Post-op CD	Post-cholecystectomy	0.8188405797101449
3050	C69	P9	Post-op CD	Post-cholecystectomy	0.8714721586575134
3051	C69	P13	Post-op CD	Post-cholecystectomy	0.86441647597254
3052	C69	P15	Post-op CD	Post-cholecystectomy	0.734744469870328
3053	C69	P16	Post-op CD	Post-cholecystectomy	0.7845156369183829
3054	C69	P17	Post-op CD	Post-cholecystectomy	0.8781464530892449
3055	C69	P20	Post-op CD	Post-cholecystectomy	0.7740274599542334
3056	C69	P21	Post-op CD	Post-cholecystectomy	0.8548817696414951
3057	C69	P24	Post-op CD	Post-cholecystectomy	0.8482074752097636
3058	C69	P26	Post-op CD	Post-cholecystectomy	0.8161708619374524
3059	C69	P30	Post-op CD	Post-cholecystectomy	0.918001525553013
3060	C69	P33	Post-op CD	Post-cholecystectomy	0.8560259344012204
3061	C69	P35	Post-op CD	Post-cholecystectomy	0.8768115942028986
3062	C69	P38	Post-op CD	Post-cholecystectomy	0.9019832189168574
3063	C69	P39	Post-op CD	Post-cholecystectomy	0.8585049580472921
3064	C69	P42	Post-op CD	Post-cholecystectomy	0.8991228070175439
3065	C69	P43	Post-op CD	Post-cholecystectomy	0.8037757437070938
3066	C69	P46	Post-op CD	Post-cholecystectomy	0.6466437833714722
3067	C69	P47	Post-op CD	Post-cholecystectomy	0.8173150266971777
3068	C69	P50	Post-op CD	Post-cholecystectomy	0.8377192982456141
3069	C69	P55	Post-op CD	Post-cholecystectomy	0.8686117467581999

3070	C69	P58	Post-op CD	Post-cholecystectomy	0.8432494279176201
3071	C69	P60	Post-op CD	Post-cholecystectomy	0.7675438596491229
3072	C69	P63	Post-op CD	Post-cholecystectomy	0.8691838291380626
3073	C69	P65	Post-op CD	Post-cholecystectomy	0.8401983218916858
3074	C69	P68	Post-op CD	Post-cholecystectomy	0.8960717009916095
3075	C69	P70	Post-op CD	Post-cholecystectomy	0.8749046529366895
3076	C69	P71	Post-op CD	Post-cholecystectomy	0.7070938215102975
3077	C69	P74	Post-op CD	Post-cholecystectomy	0.8495423340961098
3078	C69	P75	Post-op CD	Post-cholecystectomy	0.8192219679633868
3079	C70	P1	Post-op CD	Post-cholecystectomy	0.8895881006864989
3080	C70	P2	Post-op CD	Post-cholecystectomy	0.8400076277650649
3081	C70	P4	Post-op CD	Post-cholecystectomy	0.9412662090007627
3082	C70	P5	Post-op CD	Post-cholecystectomy	0.8731884057971014
3083	C70	P9	Post-op CD	Post-cholecystectomy	0.9340198321891686
3084	C70	P13	Post-op CD	Post-cholecystectomy	0.8943554538520213
3085	C70	P15	Post-op CD	Post-cholecystectomy	0.8060640732265446
3086	C70	P16	Post-op CD	Post-cholecystectomy	0.9155225019069413
3087	C70	P17	Post-op CD	Post-cholecystectomy	0.9080854309687262
3088	C70	P20	Post-op CD	Post-cholecystectomy	0.9080854309687262
3089	C70	P21	Post-op CD	Post-cholecystectomy	0.9189549961861174
3090	C70	P24	Post-op CD	Post-cholecystectomy	0.8520213577421816
3091	C70	P26	Post-op CD	Post-cholecystectomy	0.944698703279939
3092	C70	P30	Post-op CD	Post-cholecystectomy	0.9429824561403509
3093	C70	P33	Post-op CD	Post-cholecystectomy	0.9088482074752098
3094	C70	P35	Post-op CD	Post-cholecystectomy	0.92372234935164
3095	C70	P38	Post-op CD	Post-cholecystectomy	0.8975972540045767
3096	C70	P39	Post-op CD	Post-cholecystectomy	0.9017925247902364

3097 C70 P42 Post-op CD Post-cholecystectomy
0.9305873379099924
3098 C70 P43 Post-op CD Post-cholecystectomy
0.8941647597254004
3099 C70 P46 Post-op CD Post-cholecystectomy
0.7631578947368421
3100 C70 P47 Post-op CD Post-cholecystectomy
0.8386727688787186
3101 C70 P50 Post-op CD Post-cholecystectomy
0.8899694889397407
3102 C70 P55 Post-op CD Post-cholecystectomy
0.9090389016018307
3103 C70 P58 Post-op CD Post-cholecystectomy
0.9344012204424104
3104 C70 P60 Post-op CD Post-cholecystectomy
0.9492753623188406
3105 C70 P63 Post-op CD Post-cholecystectomy
0.9277269260106789
3106 C70 P65 Post-op CD Post-cholecystectomy
0.9040808543096872
3107 C70 P68 Post-op CD Post-cholecystectomy
0.919908466819222
3108 C70 P70 Post-op CD Post-cholecystectomy
0.8729977116704806
3109 C70 P71 Post-op CD Post-cholecystectomy
0.7168192219679634
3110 C70 P74 Post-op CD Post-cholecystectomy
0.9149504195270786
3111 C70 P75 Post-op CD Post-cholecystectomy
0.8220823798627003
3112 C74 P1 Post-op CD Post-cholecystectomy
0.8270404271548436
3113 C74 P2 Post-op CD Post-cholecystectomy
0.8424866514111365
3114 C74 P4 Post-op CD Post-cholecystectomy
0.9496567505720824
3115 C74 P5 Post-op CD Post-cholecystectomy
0.8964530892448512
3116 C74 P9 Post-op CD Post-cholecystectomy
0.950419527078566
3117 C74 P13 Post-op CD Post-cholecystectomy
0.9099923722349351
3118 C74 P15 Post-op CD Post-cholecystectomy
0.8291380625476735
3119 C74 P16 Post-op CD Post-cholecystectomy
0.9361174675819984
3120 C74 P17 Post-op CD Post-cholecystectomy
0.9241037376048817
3121 C74 P20 Post-op CD Post-cholecystectomy
0.8632723112128147
3122 C74 P21 Post-op CD Post-cholecystectomy
0.9441266209000763
3123 C74 P24 Post-op CD Post-cholecystectomy
0.9012204424103738

3124	C74	P26	Post-op CD	Post-cholecystectomy	0.9570938215102975
3125	C74	P30	Post-op CD	Post-cholecystectomy	0.9473684210526315
3126	C74	P33	Post-op CD	Post-cholecystectomy	0.9315408085430968
3127	C74	P35	Post-op CD	Post-cholecystectomy	0.933066361556064
3128	C74	P38	Post-op CD	Post-cholecystectomy	0.9595728451563692
3129	C74	P39	Post-op CD	Post-cholecystectomy	0.9387871853546911
3130	C74	P42	Post-op CD	Post-cholecystectomy	0.9670099160945843
3131	C74	P43	Post-op CD	Post-cholecystectomy	0.9443173150266971
3132	C74	P46	Post-op CD	Post-cholecystectomy	0.834096109839817
3133	C74	P47	Post-op CD	Post-cholecystectomy	0.8504958047292144
3134	C74	P50	Post-op CD	Post-cholecystectomy	0.9002669717772692
3135	C74	P55	Post-op CD	Post-cholecystectomy	0.9151411136536994
3136	C74	P58	Post-op CD	Post-cholecystectomy	0.940884820747521
3137	C74	P60	Post-op CD	Post-cholecystectomy	0.9769260106788711
3138	C74	P63	Post-op CD	Post-cholecystectomy	0.9250572082379863
3139	C74	P65	Post-op CD	Post-cholecystectomy	0.9227688787185355
3140	C74	P68	Post-op CD	Post-cholecystectomy	0.9355453852021358
3141	C74	P70	Post-op CD	Post-cholecystectomy	0.9078947368421053
3142	C74	P71	Post-op CD	Post-cholecystectomy	0.8033943554538521
3143	C74	P74	Post-op CD	Post-cholecystectomy	0.9096109839816934
3144	C74	P75	Post-op CD	Post-cholecystectomy	0.8920671243325705
3145	C78	P1	Post-op CD	Post-cholecystectomy	0.9448893974065599
3146	C78	P2	Post-op CD	Post-cholecystectomy	0.8291380625476735
3147	C78	P4	Post-op CD	Post-cholecystectomy	0.7486651411136537
3148	C78	P5	Post-op CD	Post-cholecystectomy	0.8051106025934401
3149	C78	P9	Post-op CD	Post-cholecystectomy	0.7801296720061022
3150	C78	P13	Post-op CD	Post-cholecystectomy	0.8647978642257819

3151	C78	P15	Post-op CD	Post-cholecystectomy	0.8323798627002288
3152	C78	P16	Post-op CD	Post-cholecystectomy	0.7069031273836766
3153	C78	P17	Post-op CD	Post-cholecystectomy	0.8401983218916858
3154	C78	P20	Post-op CD	Post-cholecystectomy	0.7702135774218154
3155	C78	P21	Post-op CD	Post-cholecystectomy	0.7599160945842868
3156	C78	P24	Post-op CD	Post-cholecystectomy	0.9422196796338673
3157	C78	P26	Post-op CD	Post-cholecystectomy	0.7057589626239512
3158	C78	P30	Post-op CD	Post-cholecystectomy	0.9555682684973302
3159	C78	P33	Post-op CD	Post-cholecystectomy	0.7953852021357742
3160	C78	P35	Post-op CD	Post-cholecystectomy	0.7505720823798627
3161	C78	P38	Post-op CD	Post-cholecystectomy	0.9610983981693364
3162	C78	P39	Post-op CD	Post-cholecystectomy	0.8161708619374524
3163	C78	P42	Post-op CD	Post-cholecystectomy	0.8047292143401983
3164	C78	P43	Post-op CD	Post-cholecystectomy	0.6821128909229596
3165	C78	P46	Post-op CD	Post-cholecystectomy	0.7164378337147216
3166	C78	P47	Post-op CD	Post-cholecystectomy	0.9715865751334859
3167	C78	P50	Post-op CD	Post-cholecystectomy	0.7145308924485125
3168	C78	P55	Post-op CD	Post-cholecystectomy	0.7515255530129672
3169	C78	P58	Post-op CD	Post-cholecystectomy	0.8100686498855835
3170	C78	P60	Post-op CD	Post-cholecystectomy	0.8714721586575134
3171	C78	P63	Post-op CD	Post-cholecystectomy	0.5949656750572082
3172	C78	P65	Post-op CD	Post-cholecystectomy	0.7170099160945843
3173	C78	P68	Post-op CD	Post-cholecystectomy	0.725209763539283
3174	C78	P70	Post-op CD	Post-cholecystectomy	0.4832189168573608
3175	C78	P71	Post-op CD	Post-cholecystectomy	0.8321891685736079
3176	C78	P74	Post-op CD	Post-cholecystectomy	0.8201754385964912
3177	C78	P75	Post-op CD	Post-cholecystectomy	0.8003432494279176
