# Supplementary material for: The analysis of gut microbiota in patients with bile acid diarrhoea treated with colesevelam
Source: Front Microbiol. 2023 Mar 17;14:1134105. doi: 10.3389/fmicb.2023.1134105 (PMC10063896; doi:10.3389/fmicb.2023.1134105)
Supplement: Supplementary file 5 [file Table_1.DOCX]

*Supplementary table 1: Faecal samples collected from each patient cohort. Patients with a positive diagnosis of BAD were given a trial of treatment with the bile acid sequestrant colesevelam. Reasons for exclusion and study withdrawal listed. CG: ^75^SeHCAT negative control group, BAD: idiopathic bile acid diarrhoea cohort, PC: post-cholecystectomy cohort, CD: post-terminal ileal resected Crohn’s disease cohort. DNA: did not attend outpatient clinic appointment and could not obtain sample at required study timepoint. COVID: Due to SARS-COV-2 virus restrictions, unable to collect samples at required study timepoints. *Of the post-operative Crohn’s disease cohort, 14 patients had a primary terminal ileal resection that were eligible to participate in sub-study cohort.*

|  | | | ***CG*** | ***BAD*** | ***PC*** | ***CD*** |
| --- | --- | --- | --- | --- | --- | --- |
| *Baseline* | | | *38* | *35* | *34* | *28* |
| *4-weeks post-treatment* | | | *-* | *23* | *16* | *18* |
|  | *Excluded*  *Study withdrawal* | |  | *0*  *2 (adverse effects)*  *3 (DNA)*  *1 (COVID)*  *6 (withdrawal, unknown cause)* | *9 (negative ^75^SeHCAT)*  *1 (adverse effects)*  *1 (DNA)*  *7 (withdrawal, unknown cause)* | *4 (negative ^75^SeHCAT)*  *2 (DNA)*  *4 (withdrawal, unknown cause)* |
| *8-weeks post-treatment* | | | *-* | *24* | *12* | *18* |
|  | | *Excluded*  *Study withdrawal* |  | *0*  *4 (adverse effects)*  *1 (COVID)*  *6 (withdrawal, unknown cause)* | *9 (negative ^75^SeHCAT)*  *1 (adverse effects)*  *5 (DNA)*  *7 (withdrawal, unknown cause)* | *4 (negative ^75^SeHCAT)*  *1 (DNA)*  *1 (COVID)*  *4 (withdrawal, unknown cause)* |
| *6-12 months post-treatment* | | | *-* | *-* | *-* | *11** |
|  | | *Study withdrawal* | *-* | *-* | *-* | *3 (COVID)* |

*Supplementary table 2: Patient demographics in total and per group cohort. [a- Includes hypertension, cerebrovascular disease, asthma or chronic obstructive pulmonary disease, hypercholesterolaemia, epilepsy, and hypothyroid disease. b- Although generally prescribed for depression and/or anxiety, some patients were prescribed this for analgesic relief. c- Includes co-codamol or other opioid derivative, non-steroidal anti-inflammatory agents, pregabalin or gabapentin. d- Includes hypoglycaemics, anti-hypertensives, anti-epileptics, statins]*

|  | | | **Total**  **(n=134)** | **CG**  **(n=29)** | **BAD**  **(n=43)** | **PC**  **(n=34)** | **CD**  **(n=28)** |
| --- | --- | --- | --- | --- | --- | --- | --- |
| **Age (years)** | | | 49.8 | 54.3 | 44.5 | 57.4 | 44.2 |
| **Gender** | | | | | | | |
|  | Male | | 51  (38.1%) | 8  (27.6%) | 20  (46.5%) | 10  (29.4%) | 13  (46.4%) |
|  | Female | | 82  (61.2%) | 21  (93.1%) | 23  (53.5%) | 23  (67.6%) | 15  (53.6%) |
|  | Unknown | | 1  (0.7%) | 0 | 0 | 1  (2.9%) | 0 |
| **Ethnicity** | | | | | | | |
|  | White British | | 113 (84.3%) | 26  (89.7%) | 34  (79%) | 29  (85.3%) | 24  (85.7%) |
|  | Indian Asian | | 7  (5.2%) | 0 | 5  (11.6%) | 1  (2.9%) | 1  (3.6%) |
|  | Black Caribbean | | 3  (2.2%) | 1  (3.4%) | 0 | 1  (2.9%) | 1  (3.6%) |
|  | Unknown | | 11  (8.2%) | 2  (6.9%) | 4  (9.3%) | 3  (8.8%) | 2  (7.1%) |
| **Co-morbidities** | | | | | | | |
|  | IHD | | 8  (6.0%) | 2  (6.9%) | 1  (2.3%) | 4  (11.8%) | 1  (3.6%) |
|  | Diabetes mellitus | | 9  (6.7%) | 2  (6.9%) | 2  (4.7%) | 5  (14.7%) | 0 |
|  | Dyspepsia/ Gastro-oesophageal reflux disease | | 12  (9.0%) | 4  (13.8%) | 3  (7%) | 5  (14.7%) | 0 |
|  | Malignancy | | 13  (9.7%) | 4  (13.8%) | 5  (11.6%) | 3  (8.8%) | 1  (3.6%) |
|  | Ulcerative colitis | | 4  (3.0%) | 0 | 3  (7%) | 1  (2.9%) | 0 |
|  | Crohn’s disease | | 31  (23.1%) | 0 | 2  (4.7%) | 1  (2.9%) | 28  (100%) |
|  | Anxiety/ Depression | | 16  (11.9%) | 7  (24.1%) | 5  (11.6%) | 3  (8.8%) | 1  (3.6%) |
|  | Chronic pain/ Fibromyalgia | | 10  (7.5%) | 6  (20.1%) | 1  (2.3%) | 3  (8.8%) | 0 |
|  | Others^a^ | | 47  (35.1%) | 14  (48.3%) | 13  (30.2%) | 15  (44.1%) | 5  (17.9%) |
|  | Nil | | 32  (23.9%) | 8  (27.6%) | 19  (44.2%) | 5  (14.7%) | 0 |
|  | Unknown | | 6  (4.5%) | 0 | 1  (2.3%) | 5  (14.7%) | 0 |
| **Medications** | | | | | | | |
|  | | Anti-depressants^b^ | 27  (20.1%) | 9  (31.0%) | 10  (23.3%) | 6  (17.6%) | 2  (7.1%) |
|  | | PPI | 38  (28.4%) | 6  (20.1%) | 14  (32.6%) | 16  (57.1%) | 2  (7.1%) |
|  | | Anti-spasmodics | 21  (15.7%) | 8  (27.6%) | 5  (11.6%) | 7  (20.6%) | 1  (3.6%) |
|  | | Analgesics^c^ | 14  (10.4%) | 5  (17.2%) | 4  (9.3%) | 5  (14.7%) | 0 |
|  | | Biologics | 8  (6.0%) | 1  (3.4%) | 2  (4.7%) | 0 | 5  (17.9%) |
|  | | Immunosuppressants | 8  (6.0%) | 0 | 1  (2.3%) | 0 | 7  (17.9%) |
|  | | Others^d^ | 51  (38.1%) | 15  (51.7%) | 13  (30.2%) | 17  (50%) | 6  (21.4%) |
|  | | Nil | 45  (33.6%) | 7  (24.1%) | 20  (46.5%) | 4  (11.8%) | 14  (50%) |
|  | | Unknown | 10  (7.5%) | 0 | 2  (4.7%) | 7  (20.6%) | 1  (3.6%) |

Supplementary Table 3: Mean Stool frequency and consistency with standard deviation pre- and post-treatment with bile acid sequestrants in the different patient cohorts with diagnosed bile acid diarrhoea. *CG = SeHCAT negative control group, BAD = idiopathic bile acid diarrhoea, PC = post-cholecystectomy, CD = post-operative terminal ileal resected Crohn’s disease. (Table used with permission, already published by Kumar et al 2022)^1^*

| **Treatment cohort** | **Mean bowel movements per day** | |  | **Mean consistency based on Bristol stool chart** | |  |
| --- | --- | --- | --- | --- | --- | --- |
|  | **Before Treatment** | **After treatment** | **p-value** | **Before treatment** | **After treatment** | **p-value** |
| CG | 4.4 (2.5) |  |  | 5.8 (0.9) |  |  |
| BAD | 5.6 (2.7) | 3.7 (1.7) | 0.0004 | 5.7 (1.2) | 4.2 (1.8) | 0.02 |
| PC | 6.0 (5.3) | 2.8 (2.2) | 0.07 | 4.6 (1.8) | 3.6 (0.9) | 0.30 |
| CD | 5.6 (2.0) | 2.0 (1.0) | <0.0001 | 5.5 (0.7) | 4.3 (1.7) | 0.39 |

**References**

1. Kumar A, Galbraith N, Al-Hassi HO, et al. The impact of treatment with bile acid sequestrants on quality of life in patients with bile acid diarrhoea. *BMC Gastroenterol*. Jul 2 2022;22(1):325. doi:10.1186/s12876-022-02404-9
